# A systematic revision of *Baconia* Lewis (Coleoptera, Histeridae, Exosternini)

**DOI:** 10.3897/zookeys.343.5744

**Published:** 2013-10-15

**Authors:** Michael S. Caterino, Alexey K. Tishechkin

**Affiliations:** 1 Department of Invertebrate Zoology, Santa Barbara Museum of Natural History, 2559 Puesta del Sol, Santa Barbara, CA 93105 USA; 2 Louisiana State Arthropod Museum, Department of Entomology, Louisiana State University, 404 Life Sciences Building, Baton Rouge, LA 70803 USA

**Keywords:** Histeridae, Histerinae, Exosternini, taxonomy, subcortical, Neotropical region, Nearctic region, Palaearctic region, Oriental region

## Abstract

Here we present a complete revision of the species of *Baconia*. Up until now there have been 27 species assigned to the genus (Mazur, 2011), in two subgenera (*Binhister* Cooman and *Baconia* s. str.), with species in the Neotropical, Nearctic, Palaearctic, and Oriental regions. We recognize all these species as valid and correctly assigned to the genus, and redescribe all of them. We synonymize *Binhister*, previously used for a polyphyletic assemblage of species with varied relationships in the genus. We move four species into *Baconia* from other genera, and describe 85 species as new, bringing the total for the genus to 116 species. We divide these into 12 informal species groups, leaving 13 species unplaced to group. We present keys and diagnoses for all species, as well as habitus photos and illustrations of male genitalia for nearly all. The genus now contains the following species and species groups: *Baconia loricata* group [*Baconia loricata* Lewis, 1885,* B. patula* Lewis, 1885, *Baconia gounellei* (Marseul, 1887a), *Baconia jubaris* (Lewis, 1901), *Baconia festiva* (Lewis, 1891), *Baconia foliosoma* **sp. n.**, *Baconia sapphirina* **sp. n.**, *Baconia furtiva* **sp. n.**, *Baconia pernix* **sp. n.**, *Baconia applanatis* **sp. n.**, *Baconia disciformis* **sp. n.**, *Baconia nebulosa* **sp. n.**, *Baconia brunnea* **sp. n.**], *Baconia godmani* group [*Baconia godmani* (Lewis, 1888), *Baconia venusta* (J. E. LeConte, 1845), *Baconia riehli* (Marseul, 1862), **comb. n.**, *Baconia scintillans* **sp. n.**, *Baconia isthmia* **sp. n.**, *Baconia rossi* **sp. n.**, *Baconia navarretei* **sp. n.**, *Baconia maculata* **sp. n.**, *Baconia deliberata* **sp. n.**, *Baconia excelsa* **sp. n.**, *Baconia violacea* (Marseul, 1853), *Baconia varicolor* (Marseul, 1887b), *Baconia dives* (Marseul, 1862), *Baconia eximia* (Lewis, 1888), *Baconia splendida* **sp. n.**, *Baconia jacinta* **sp. n.**, *Baconia prasina* **sp. n.**, *Baconia opulenta* **sp. n.**, *Baconia illustris* (Lewis, 1900), *Baconia choaspites* (Lewis, 1901), *Baconia lewisi* Mazur, 1984], *Baconia salobrus* group [*Baconia salobrus* (Marseul, 1887b), *Baconia turgifrons* **sp. n.**, *Baconia crassa* **sp. n.**, *Baconia anthracina* **sp. n.**, *Baconia emarginata* **sp. n.**, *Baconia obsoleta* **sp. n.**], *Baconia ruficauda* group [*Baconia ruficauda* **sp. n.**, *Baconia repens* **sp. n.**], *Baconia angusta* group [*Baconia angusta* Schmidt, 1893a, *Baconia incognita* **sp. n.**, *Baconia guartela* **sp. n.**, *Baconia bullifrons* **sp. n.**, *Baconia cavei* **sp. n.**, *Baconia subtilis* **sp. n.**, *Baconia dentipes* **sp. n.**, *Baconia rubripennis* **sp. n.**, *Baconia lunatifrons* **sp. n.**], *Baconia aeneomicans* group [*Baconia aeneomicans* (Horn, 1873), *Baconia pulchella* **sp. n.**, *Baconia quercea* **sp. n.**, *Baconia stephani* **sp. n.**, *Baconia irinae* **sp. n.**, *Baconia fornix* **sp. n.**, *Baconia slipinskii* Mazur, 1981, *Baconia submetallica* **sp. n.**, *Baconia diminua* **sp. n.**, *Baconia rufescens* **sp. n.**, *Baconia punctiventer* **sp. n.**, *Baconia aulaea* **sp. n.**, *Baconia mustax* **sp. n.**, *Baconia plebeia* **sp. n.**, *Baconia castanea* **sp. n.**, *Baconia lescheni* **sp. n.**, *Baconia oblonga* **sp. n.**, *Baconia animata* **sp. n.**, *Baconia teredina* **sp. n.**, *Baconia chujoi* (Cooman, 1941), *Baconia barbarus* (Cooman, 1934), *Baconia reposita* **sp. n.**, *Baconia kubani* **sp. n.**, *Baconia wallacea* **sp. n.**, *Baconia bigemina* **sp. n.**, *Baconia adebratti* **sp. n.**, *Baconia silvestris* **sp. n.**], *Baconia cylindrica* group [*Baconia cylindrica* **sp. n.**, *Baconia chatzimanolisi* **sp. n.**], *Baconia gibbifer* group [*Baconia gibbifer* **sp. n.**,* B. piluliformis* **sp. n.**, *Baconia maquipucunae* **sp. n.**, *Baconia tenuipes* **sp. n.**, *Baconia tuberculifer* **sp. n.**, *Baconia globosa* **sp. n.**], *Baconia insolita* group [*Baconia insolita* (Schmidt, 1893a), **comb. n.**, *Baconia burmeisteri* (Marseul, 1870), *Baconia tricolor* **sp. n.**, *Baconia pilicauda* **sp. n.**], *Baconia riouka* group [*Baconia riouka* (Marseul, 1861), *Baconia azuripennis* **sp. n.**], *Baconia famelica* group [*Baconia famelica* **sp. n.**, *Baconia grossii* **sp. n.**, *Baconia redemptor* **sp. n.**, *Baconia fortis* **sp. n.**, *Baconia longipes* **sp. n.**, *Baconia katieae* **sp. n.**, *Baconia cavifrons* (Lewis, 1893), **comb. n.**, *Baconia haeterioides* **sp. n.**], *Baconia micans* group [*Baconia micans* (Schmidt, 1889a), *Baconia carinifrons* **sp. n.**, *Baconia fulgida* (Schmidt, 1889c)], *Baconia* incertae sedis [*Baconia chilense* (Redtenbacher, 1867), *Baconia glauca* (Marseul, 1884), *Baconia coerulea* (Bickhardt, 1917), *Baconia angulifrons* **sp. n.**, *Baconia sanguinea* **sp. n.**, *Baconia viridimicans* (Schmidt, 1893b), *Baconia nayarita* **sp. n.**, *Baconia viridis* **sp. n.**, *Baconia purpurata* **sp. n.**, *Baconia aenea* **sp. n.**, *Baconia clemens* **sp. n.**, *Baconia leivasi* **sp. n.**, *Baconia atricolor* **sp. n.**]. We designate lectotypes for the following species: *Baconia loricata* Lewis, 1885,*Phelister gounellei* Marseul, 1887, *Baconia jubaris* Lewis, 1901, *Baconia festiva* Lewis, 1891, *Platysoma venustum* J.E. LeConte, 1845, *Phelister riehli* Marseul, 1862, *Phelister violaceus* Marseul, 1853, *Phelister varicolor* Marseul, 1887b, *Phelister illustris* Lewis, 1900, *Baconia choaspites* Lewis, 1901, *Epierus festivus* Lewis, 1898, *Phelister salobrus* Marseul, 1887, *Baconia angusta* Schmidt, 1893a, *Phelister insolitus* Schmidt, 1893a, *Pachycraerus burmeisteri* Marseul, 1870, *Phelister riouka* Marseul, 1861, *Homalopygus cavifrons* Lewis, 1893, *Phelister micans* Schmidt, 1889a, *Phelister coeruleus* Bickhardt, 1917, and *Phelister viridimicans* Schmidt, 1893b. We designate neotypes for *Baconia patula* Lewis, 1885 and *Hister aeneomicans* Horn, 1873, whose type specimens are lost.

## Introduction

The genus *Baconia* Lewis includes some of the most beautiful and remarkable of all histerids. Many of the species are brilliantly metallic blue, green, or violet, and may rarely have various maculations. Many also exhibit extremely flattened body forms, easily rivalling the better-known Hololeptini for flatness. The genus was described by Lewis (1885) to accommodate one of the most extreme of these, the very large (~5mm) and highly flattened *Baconia loricata* Lewis. Many subsequently described species were recognized for their distinctive coloration, and the group is generally considered among the more easily recognizeable and distinctive elements of the New World Exosternini fauna.

Unfortunately, the reality of *Baconia* is much more complex. In fact the genus has suffered from numerous taxonomic and systematic problems. Initially, Lewis himself apparently regarded only the most extremely flattened species to properly belong to *Baconia*, and he described most of the species now included here in *Phelister*, despite what appears to modern taxonomists to be obvious similarities in structure. There was also a period during which its status as an independent genus or as a subgenus of *Phelister* was in flux (e.g. Schmidt 1889a, 1889b), and even its higher level placement was questioned by its author (Lewis 1891). There has been a question as to the status of the genus *Binhister* Cooman, which has contained two Asian and one Neotropical species, whether it should be included as a subgenus of *Baconia* (as in Mazur 1997, 2011) or as an independent genus (Mazur 1984; Ôhara 1989, 1994). Worst of all, the taxonomic history of *Baconia* became further intertwined with that of *Phelister*, when Lewis (1889) offhandedly designated the type of *Phelister* to be a species that is now considered an unquestioned member of *Baconia*, *Baconia venusta* (LeConte). This and other problems have been overlooked or ignored by most subsequent authors. A proposal to the ICZN to suppress the potentially disruptive type designation for *Phelister* has been published, though not yet ruled upon (Caterino and Tishechkin 2013b). In addition to the taxonomic problems, there is also a tremendous wealth of undescribed diversity of *Baconia*, many of which species significantly expand the conventional conception of the genus.

The original definition of the genus was rather vague as to defining characters. Of those provided, only a few might be construed as unique to the genus ‘*corpus depressum...prosternum parum angustatum…mesosternum latissimum antice rectum…tibiae extus unidentatae vel inermes*’ (Lewis 1885: 462), with the remainder of the description listing characters common to Histerinae or even Histeridae as a whole. Of these Lewis clearly considered the flattened body and correspondingly wide prosternum to be the most significant, as convex species sharing most of these characters were shortly thereafter described in *Phelister* (e.g., *Baconia godmani* Lewis, 1888; *Baconia eximia* Lewis, 1888). Lewis’s tight circumscription of *Baconia* was soon challenged. When describing the convex *‘Phelister’ micans*, Schmidt (1889a) suggested that it might be better placed in *Baconia*. Schmidt (1889b) provided a longer list of *Phelister* spp. that might be grouped under *Baconia*. And Schmidt (1889c) treated his broader *Baconia*, first as a subgenus of *Phelister*, and then as a distinct genus (Schmidt 1893a). Schmidt’s broader conception rested on the arrangement of marginal spines of the protibia, the anterior depressions of the pronotum, the weak to absent spines of the posterior tibiae, and the possession of punctures near the apices of the elytra (Schmidt 1889b). Lewis (1901) subsequently began describing a few additional species in *Baconia*, but explicitly disagreed with Schmidt’s assignment of *micans, fulgidus*, and *angustus* to the genus, citing their bisinuate mesosternum. He never addressed Schmidt’s conception of the genus directly, and kept the genera widely separated in his 1905 catalog (*Baconia* among what we now consider to be Platysomatini genera, *Phelister* among the other Exosternini). Bickhardt (1917) followed Lewis’s delimitations of *Baconia* and *Phelister*, and assigned them to Histerini and Exosternini, respectively. It was not until Mazur (1981) described *Baconia slipinskii*, comparing it to Schmidt’s *Baconia angusta* that the issue was, however obliquely, addressed again. Finally, in 1984, Mazur enlarged the concept of *Baconia*, essentially to that espoused by Schmidt nearly 100 years prior.

**Figure 1.**
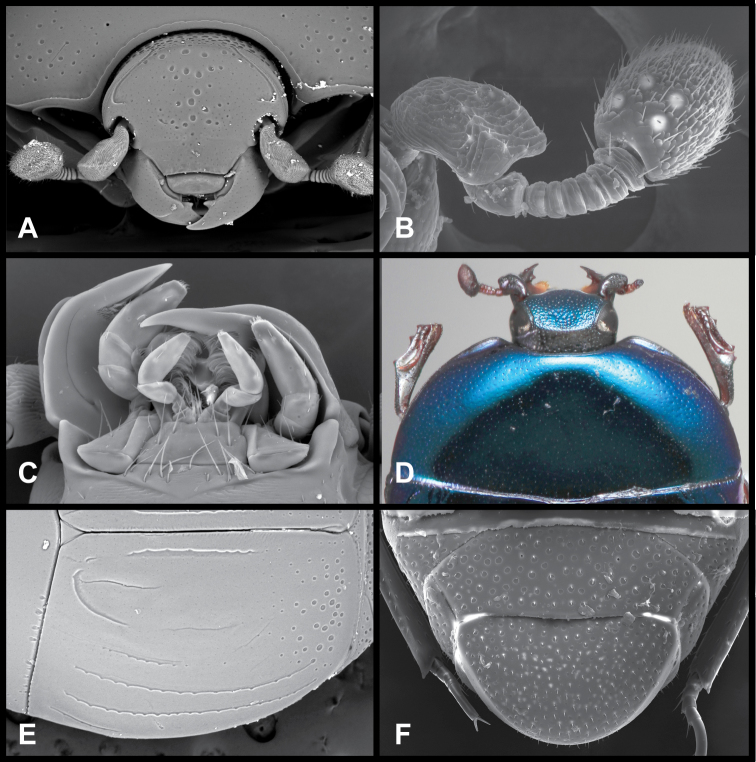
*Baconia* generic characters. **A** Frons of *Baconia anthracina* **B** Antenna of *Baconia tricolor* **C** Mouthparts of *Baconia gibbifer* **D** Pronotum of *Baconia disciformis* **E** Elytron of *Baconia anthracina* **F** Pygidia of *Baconia tricolor*.

Our definition of *Baconia* relies primarily on one unambiguous morphological character. The fundamental synapomorphy of the group is a very distinctive antennal club, which has four sensory openings on each upper and lower surface (Figs 1B, 54E, 64C). These apparently occur along what in other Histeridae would be the annuli dividing the three subsections of the club. In most *Baconia* species the club is entirely tomentose, and these sensoria are surrounded by small glabrous rims. However, in a few taxa (the *Baconia famelica* group), much of the surface of the club is glabrous. This character unites a diversity of general body forms, some of which might be considered distinctive taxa of their own. The most extreme of these is an apparent myrmecophile with spectacular pygidial trichomes. Given the strong antennal character, however, we prefer to keep all these together within the genus. There remains one small lineage of uncertain relationships, which we exclude from *Baconia* for the present. This lineage comprises *Phelister simoni* and one undescribed close relative from the southern USA. These species superficially look nothing like *Baconia*, and lack this synapomorphic antennal club. However, phylogenetic analyses, which include DNA for the undescribed species, place these either at the base of, or even within, *Baconia* (Caterino and Tishechkin in prep.) Lacking any clear morphological character support for inclusion, we consider the *Baconia simoni* lineage most likely to be a sister lineage to *Baconia*, and will be describing a new genus for it in a separate paper (Caterino, Tishechkin and Proudfoot in prep.)

Below we present a complete revision of the species of *Baconia*. There are presently 27 species assigned to the genus (Mazur 2011), in two subgenera (*Binhister* Cooman and *Baconia* s. str.). We fully synonymize the subgenus *Binhister* with *Baconia*, as it has previously referred to an artificial group of species. We recognize all 27 described species as valid and correctly assigned, and redescribe all of them. We move four species into *Baconia* from other genera, and describe 85 species as new, bringing the total for the genus to 116 species. We present keys and diagnoses for all species, establish a series of informal species groups to facilitate identification, and provide habitus photos and illustrations of male genitalia for nearly all species.

## Materials and methods

Type material of all New World species was examined by one or both of the authors. We have not not seen type material of two of the three Asian species, as the primary emphasis of the study was on the New World fauna. Nonetheless we have established identities of the Asian species with reasonable confidence through reference to other authoritatively determined specimens.

Conventional imaging was done using a Visionary Digital’s ‘Passport’ portable imaging system, which incorporates a Canon D7 with MP-E 65mm 1–5×macro zoom lens. Images were stacked using Helicon Focus software. SEM imaging was done on a Zeiss EVO 40 scope, most specimens sputter coated with gold, or uncoated specimens examined in ‘variable pressure’ mode. We present only selected images as necessary to identify the species in this paper. However, multiple photographs of all species have been archived in MorphBank (www.morphbank.net), and are also available through the Encyclopedia of Life (www.eol.org). Following histerid conventions, total body length is measured from the anterior margin of the pronotum to the posterior margin of the elytra (to exclude preservation variability in head and pygidial extension), while width is taken at the widest point, generally near the elytral humeri.

Much of the morphological terminology used is based on Wenzel and Dybas (1941), but modified to follow more recent treatments by Helava et al. (1985), Ôhara (1994), Kanaar (1997) and Lawrence et al. (2011). We have presented an extensive discussion of Exosternini-specific morphological terminology in Caterino and Tishechkin (2013a), and refer the reader to the labeled illustrations there.

We present extensive descriptions for the majority of species. At the same time, we term these ‘diagnostic descriptions’, to emphasize the fact that they focus on those character systems in which differences among species are typically found. They are not intended to be exhaustive descriptions of each species’ morphology. We have attempted to make most of them consistent in character content and order, facilitating comparison as well as their reuse of descriptions in other contexts, such as in species pages and other media, which we encourage. The ‘remarks’ sections highlight the few most important key characters of each species.

Material examined lists provide verbatim data only for holotypes and lectotypes, and summary data for all other material, whether paratypes or non-type locality.

**Type material.** Within verbatim records, data are enclosed in double quotes, with data on separate labels separated by a slash ‘/’. The summary data generally avoids excessive repetition. Each record begins with the number of specimens exhibiting identical data. Records separated by commas are largely identical, differing only in the datum presented, most frequently distinct dates or collectors. Distinct localities are separated by semicolons, and records from distinct countries are separated by periods (full-stops).

### Specimens from the following collections were examined:

AKTC Alexey Tishechkin Collection, Santa Barbara, USA

BDGC Bruce Gill Collection, Ottawa, Canada

BMNH Natural History Museum, London, UK

CASC California Academy of Sciences Collection, San Francisco, USA

CDFA California State Collection of Arthropods, Sacramento, USA

CEMT Coleção de Entomologia, Universidade Federal do Mato Grosso, Cuiabá, Brazil

CHJG Jeffrey P. Gruber Collection, Madison, USA

CHND Nicolas Degallier Collection, Paris

CHPWK Peter Kovarik Collection, Columbus, USA

CHSM Slawomir Mazur Collection, Warsaw, Poland

CMNC Canadian Museum of Nature, Ottawa, Canada

CMNH Carnegie Museum of Natural History, Pittsburg, USA

FMNH Field Museum, Chicago, USA

FSCA Florida State Collection of Arthropods, Gainesville, USA

GBFM Museo Fairchild, Universidad de Panama, Panama City, Panama

IAVH Instituto Alexander von Humboldt, Villa de Leyva, Colombia

INBIO Instituto Nacional de Biodiversidad, San Jose, Costa Rica

LSAM Louisiana State Arthropod Museum, Baton Rouge, USA

LUND Entomological Museum of Lund University, Lund, Sweden

MCZC Museum of Comparative Zoology, Harvard University, Cambridge, USA

MHNG Museum d’Histoire Naturelle, Geneva, Switzerland

MNHN Museum National d’Histoire Naturelle, Paris, France

MSCC Michael Caterino Collection, Santa Barbara, USA

MUSM Museo de Historia Natural, Universidad Nacional Mayor de San Marcos, Lima, Peru.

NCSU North Carolina State University Collection, Raleigh, USA

NHRS Natur Historiska Riksmuseet, Stockholm, Sweden

SBMNH Santa Barbara Museum of Natural History, Santa Barbara, USA

SEMC Snow Entomology Museum, University of Kansas, Lawrence, USA

TAMU Texas A&M University Collection, College Station, USA

UDG Universidad de Guadalajara, Guadalajara, Mexico

UFPR Universidade Federal do Paraná, Curitiba, Brazil

UNESP Universdade Estadual Paulista, Faculdade de Engenharia de Ilha Solteira, Ilha Solteira, Brazil

USFQ Universidad San Francisco de Quito, Ecuador

USNM National Museum of Natural History, Washington, USA

WSUC Washington State University Insect Collection, Pullman, USA

ZMHB Zoological Museum of Humboldt University, Berlin, Germany

## Taxonomy

### Baconia

Lewis, 1885: 462

http://species-id.net/wiki/Baconia

#### Type species:

*Baconia loricata* Lewis, 1885: 463, designated by Bickhardt 1917: 163.

*Binhister* Cooman, 1934: 122 (type species *Binhister barbarus* Cooman, by original designation); previously recognized as a subgenus, it is here fully synonymized; **comb. n.**

#### Description.

**Figure 2.**
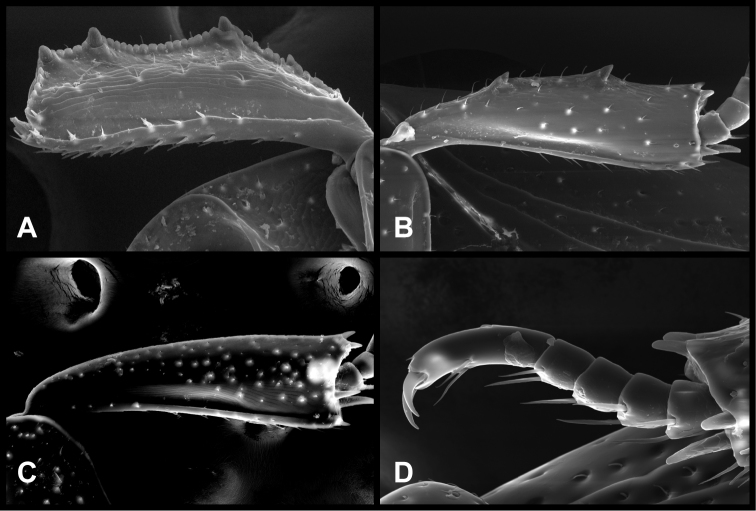
*Baconia* generic characters. **A** Protibia of *Baconia tricolor* **B** Mesotibia of *Baconia tricolor* **C** Metatibia of *Baconia anthracina* **D** Mesotarsus of *Baconia insolita*.

**Figure 3.**
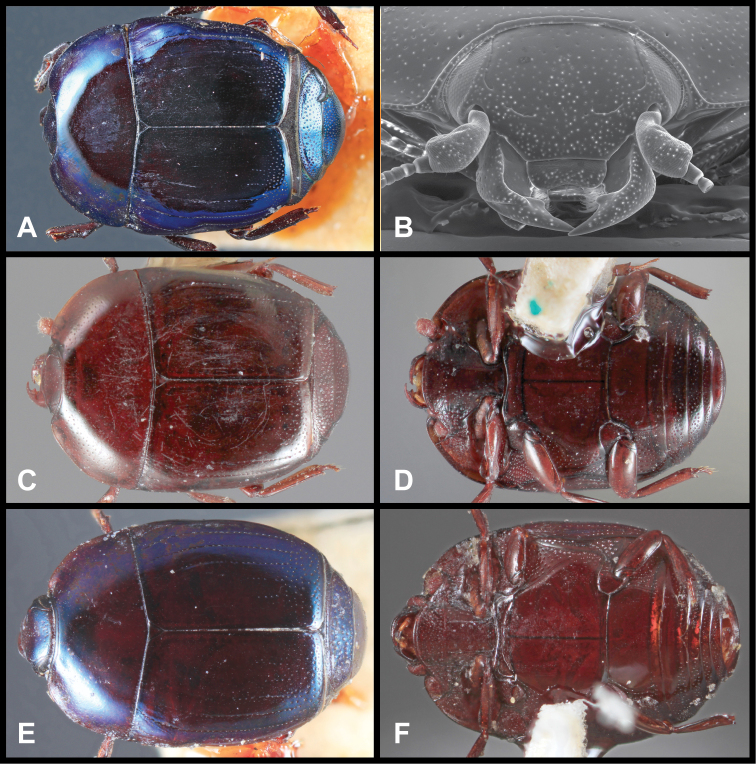
*Baconia loricata* group. **A** Dorsal habitus of lectotype of *Baconia loricata* **B** Frons of *Baconia patula* **C **Dorsal habitus of *Baconia patula* **D** Ventral habitus of *Baconia patula* **E** Dorsal habitus of lectotypeof *Baconia gounellei* **F** Ventral habitus of lectotypeof *Baconia gounellei*.

***Size range***: Length 1.0–5.0mm; width 0.6–4.0mm; ***Body***: ovoid to elongate, sides broadly rounded to sub- or fully parallel, convex to very strongly flattened; color rufescent to frequently piceous or metallic; glabrous or rarely finely setose. ***Head***: frons convex, flat, or deeply depressed, frontal stria usually present along inner margin of eyes, variably interrupted or obsolete across front, frons and epistoma frequently separated by weak to strong transverse carina; supraorbital stria present or absent; epistoma depressed to flat or convex, frequently swollen along apical margin, apical margin usually straight; labrum usually much wider than long, up to 4× or more, usually emarginate apically, but may be straight, bisinuate, or weakly produced; antennal scape usually short, stout, only weakly expanded to apex (Fig. 1A), may be longer, and/or expanded apically; antennal club generally completely tomentose, though rarely glabrous basally, annuli absent, but with 4 characteristic sensory slits on upper and lower surfaces (Fig. 1B), rarely with additional subapical sensorial patch (Fig. 54E); submentum angulate at base, truncate to projecting along distal margin, with few simple setae; gular sutures finely impressed, extending anterolaterad, uninterrupted to basal corner of buccal cavity; mentum subquadrate, sides weakly convergent, apical margin truncate to weakly emarginate, bearing few simple setae; labium with palpifers prominent, palpi with three palpomeres, the basalmost very short, the distal two with short, scattered setae; maxilla with cardo short, transverse, glabrous, stipes triangular, bearing few simple setae, palpi with four palpomeres, the basalmost very short; mandibles (Figs 1A, 3A, 28C) generally each with basal tooth, may be blunt or strong and acute, mandible frequently furrowed along lower, outer edge, may have ventral (mesal) pore and associated (presumed) secretory channel (Fig. 1C). ***Pronotum***: sides parallel to convergent apically; marginal stria usually present and continuous around lateral and anterior margins; lateral submarginal stria present or absent; anterior corners nearly always weakly depressed (Fig. 1D); prescutellar impression absent; disk with single pair of anterior marginal gland openings, usually located close to anterior margin, behind eye on each side, discal punctation highly varied. ***Elytra***: with 2–3 epipleural striae, outer subhumeral stria rarely present, inner subhumeral stria frequently present, often restricted to short basal fragment, dorsal striae 1-5 and sutural stria highly varied, variously abbreviated from base or apex or entirely absent; elytral disk nearly always with distinct secondary punctures in apical half or less (Fig. 1E). ***Prosternum***: prosternal keel varied in width, often quite broad in depressed species, basal margin varied from emarginate to truncate, rarely outwardly arcuate; carinal striae generally present, usually complete, free, rarely abbreviated anteriorly, united or obsolete; prosternal lobe short to moderate in length, apical margin subtruncate to broadly arcuate, rarely bisinuate; marginal stria of prosternal lobe usually distinct across middle, variably obsolete at sides. ***Mesoventrite***: anterior mesoventral margin ranging from distinctly emarginate to distinctly projecting, marginal stria complete to absent, rarely with secondary submarginal stria; mesometaventral stria usually present, most frequently arched forward onto mesoventrite, may in some cases partially displace or completely replace marginal mesoventral stria. ***Metaventrite***: Anterior margin, i.e., mesometaventral suture, frequently arched forward (mirrored in most, but not all cases, in mesometaventral stria), inner lateral metaventral stria generally present, extending from near inner corner of mesocoxa toward metacoxa, or toward posterior corner of metepisternum in the most depressed species, variably sinuate or abbreviated apically; outer lateral metaventral stria present or absent; metaventral disk usually coarsely punctate at sides, impunctate at middle. ***Abdomen***: 1^st^ abdominal ventrite with one or two lateral striae along inner edge of metacoxa, disk usually impunctate at middle, but with conspicuous median punctures in various species; abdominal ventrites 2-5 usually punctate at sides, rarely with dense punctures extending across middle of disk (Fig. 43F). Propygidium often with basal transverse stria (Fig. 5E), disk variably punctate sexually dimorphic in one species; single pair of propygidial gland openings usually conspicuous (Fig. 1F), situated on each side variably near basal margin; pygidium never with apical marginal stria, usually densely punctate, very rarely sexually dimorphic, with male’s setose or otherwise modified; both sexes bearing pygidial trichomes in one species.***Legs***: Protibia usually rather narrow, with 0-5 unevenly spaced marginal teeth (Fig. 2A), the outer margin nearly always finely serrulate along entire length (Fig. 2A); protibial spurs present, usually short, weakly curved; mesotibia usually with 1-2 weak marginal spines (Fig. 2B), rarely lacking spines; metatibia rarely with any marginal spines (Fig. 2C), generally smooth, with outer apical corner slightly prolonged; tarsi not obviously dimorphic, tarsomeres 1-4 short, usually bearing only single pair of apical setae, tarsomere 5 about as long as 2-4 together, usually weakly dorsoventrally curved (Fig. 2D); tarsal claws simple, separate. ***Male genitalia***:Accessory sclerites absent. T8 generally short, broad, with basal rim strongly sclerotized, basolateral edge extending beneath to inner corner of ventrolateral apodeme; ventrolateral apodeme usually acute, with distal portion strongly reduced, T8 usually broadly open beneath; basal emargination very shallow to moderately deep; basal membrane attachment line rarely evident, usually intersecting basal emargination; distal margin weakly sclerotized, poorly defined, usually vaguely emarginate. S8 articulated at basal corners with ventrolateral apodemes of T8, apical guides weakly developed, halves separate or fused; if separate, halves usually strongly divergent from base, with apices narrow, often rounded, bare to conspicuously setose; if halves of S8 fused, apical margin usually weakly emarginate, frequently with apicoventral velar membrane, bare to conspicuously setose. T9 usually divided, rarely united, with basal apodemes long and slender to short and broad; ventrolateral apodemes weak to strong, opposing or recurved basad, very rarely fused beneath; distal apices usually weakly opposed, subacute to truncate; subapical seta often present on sides. T10 entire, weakly sclerotized, apical margin rounded to weakly emarginate. S9 usually desclerotized along midline, rarely entirely divided; stem very narrow to moderately broad, frequently with ventral keel along much of length; stem rarely absent, with entire T9 short, subcordate; head of S9 usually broad, acute apicolaterally, with apical margin shallowly emarginate to sinuate; tegmen relatively simple, shallowly incised apically, parallel-sided to tapered apically, lacking medioventral tooth or process; median lobe narrow, simple, in a few species associated with small, articulated apical denticulate plates (Fig. 41O); basal piece usually short, with superficial membrane attachment line and oval, asymmetrical basal foramen. ***Female genitalia***: T8 forming a single plate, apical margin usually emarginate; S8 entire or with median plate isolated, with basal baculi detached, articulated with sternites, basally subparallel; S9 usually present, elongate; median coxite articulation present; valviferae paddle-shaped; coxites varied in shape, subquadrate to elongate, with 2-5 apical marginal teeth, with distinct, articulated apical stylus; bursa copulatrix usually completely membraneous, rarely with small sclerotizations of bursal wall; generally with single, bulbous, weakly sclerotized spermatheca, inserted near or at apex of bursa copulatrix; single, basically thin and elongate spermathecal gland present, generally attached near midpoint of spermatheca.

#### Diagnosis.

Although initially characterized on the basis of extreme flattening of the body, the species of *Baconia* in fact span a broad range from convex to very flat. They are frequently metallic in appearance, although many species exibit no hint of metallic coloration. They are best recognized by their unique antennal club sensoria – in all species the annuli are reduced to a set of 4 distinct sensory slits on both upper and lower surfaces (Figs 1B, 54E, 64C). These are found in no other Histeridae. Additional characters that can help to diagnose the genus if the antennal club is not visible: antennal scape usually short (Fig. 1A), no more than twice as long as wide, its apex obliquely truncate; frontal stria rarely complete; inner margins of eyes often strongly convergent dorsally (Fig. 1A); both mandibles usually with distinct basal tooth (Fig. 1A); anterior corners of pronotum concave to depressed (Fig. 1D); antescutellar fovea absent; lateral pronotal stria, when present, close to margin and often carinate; elytral disk with secondary punctures near apex (or, if generally punctate, punctures becoming larger and denser toward apex; Fig. 58A); prosternal striae usually present, rarely meeting anteriorly; protibia usually finely serrulate between major marginal spines; metatibia (and often mesotibia) with few or no marginal spines, and frequently prolonged at outer apex (Fig. 2C); and apical tarsomere long and somewhat curved dorsoventrally (Fig. 2D). In the Neotropical region, the depressed anterior pronotal corners, minimially spinose posterior tibiae, and presence of apical elytral secondary punctures will distinguish them from all other histerids in the region. *Hypobletus* spp. may show comparable flatness to some *Baconia*, but are always rufescent in color and parallel-sided in body form. The most flattened *Baconia* spp. are nearly all metallic and/or rounded at the sides. In the Oriental region, where all the *Baconia* spp. are moderately to strongly depressed in body shape, there may be some Platysomatini that are generally similar in body form, but these will always exhibit S-shaped protarsal grooves and complete antennal annuli.

#### Checklist of the species of *Baconia*

We establish here a series of informal species groups. These generally correspond to groups that we feel reflect monophyletic groups within the genus (and are supported as such in preliminary analyses). However, as much as anything they are intended to facilitate identification and description, uniting species that are similar and appear related. Ordering of species within species groups is also intended to reflect phylogeny to a certain degree, facilitating comparisons among closely related species and their diagnoses.

***Baconia loricata* group**

*Baconia loricata* Lewis, 1885

*Baconia patula* Lewis, 1885

*Baconia gounellei* (Marseul, 1887a)

*Baconia jubaris* (Lewis, 1901)

*Baconia festiva* (Lewis, 1891)

*Baconia foliosoma* sp. n.

*Baconia sapphirina* sp. n.

*Baconia furtiva* sp. n.

*Baconia pernix* sp. n.

*Baconia applanatis* sp. n.

*Baconia disciformis* sp. n.

*Baconia nebulosa* sp. n.

*Baconia brunnea* sp. n.

***Baconia godmani* group**

*Baconia godmani* (Lewis, 1888)

*Baconia venusta* (J. E. LeConte, 1845)

*Baconia riehli* (Marseul, 1862), comb. n.

*Baconia scintillans* sp. n.

*Baconia isthmia* sp. n.

*Baconia rossi* sp. n.

*Baconia navarretei* sp. n.

*Baconia maculata* sp. n.

*Baconia deliberata* sp. n.

*Baconia excelsa* sp. n.

*Baconia violacea* (Marseul, 1853)

*Baconia varicolor* (Marseul, 1887b)

*Baconia dives* (Marseul, 1862)

*Baconia eximia* (Lewis, 1888)

*Baconia splendida* sp. n.

*Baconia jacinta* sp. n.

*Baconia prasina* sp. n.

*Baconia opulenta* sp. n.

*Baconia illustris* (Lewis, 1900)

*Baconia choaspites* (Lewis, 1901)

*Baconia lewisi* Mazur, 1984

***Baconia salobrus* group**

*Baconia salobrus* (Marseul, 1887b)

*Baconia turgifrons* sp. n.

*Baconia crassa* sp. n.

*Baconia anthracina* sp. n.

*Baconia emarginata* sp. n.

*Baconia obsoleta* sp. n.

***Baconia ruficauda* group**

*Baconia ruficauda* sp. n.

*Baconia repens* sp. n.

***Baconia angusta* group**

*Baconia angusta* Schmidt, 1893a

*Baconia incognita* sp. n.

*Baconia guartela* sp. n.

*Baconia bullifrons* sp. n.

*Baconia cavei* sp. n.

*Baconia subtilis* sp. n.

*Baconia dentipes* sp. n.

*Baconia rubripennis* sp. n.

*Baconia lunatifrons* sp. n.

***Baconia aeneomicans* group**

*Baconia aeneomicans* (Horn, 1873)

*Baconia pulchella* sp. n.

*Baconia quercea* sp. n.

*Baconia stephani* sp. n.

*Baconia irinae* sp. n.

*Baconia fornix* sp. n.

*Baconia slipinskii* Mazur, 1981

*Baconia submetallica* sp. n.

*Baconia diminua* sp. n.

*Baconia rufescens* sp. n.

*Baconia punctiventer* sp. n.

*Baconia aulaea* sp. n.

*Baconia mustax* sp. n.

*Baconia plebeia* sp. n.

*Baconia castanea* sp. n.

*Baconia lescheni* sp. n.

*Baconia oblonga* sp. n.

*Baconia animata* sp. n.

*Baconia teredina* sp. n.

*Baconia chujoi* (Cooman, 1941)

*Baconia barbarus* (Cooman, 1934)

*Baconia reposita* sp. n.

*Baconia kubani* sp. n.

*Baconia wallacea* sp. n.

*Baconia bigemina* sp. n.

*Baconia adebratti* sp. n.

*Baconia silvestris* sp. n.

***Baconia cylindrica* group**

*Baconia cylindrica* sp. n.

*Baconia chatzimanolisi* sp. n.

***Baconia gibbifer* group**

*Baconia gibbifer* sp. n.

*Baconia piluliformis* sp. n.

*Baconia maquipucunae* sp. n.

*Baconia tenuipes* sp. n.

*Baconia tuberculifer* sp. n.

*Baconia globosa* sp. n.

***Baconia insolita* group**

*Baconia insolita* (Schmidt, 1893a), comb. n.

*Baconia burmeisteri* (Marseul, 1870)

*Baconia tricolor* sp. n.

*Baconia pilicauda* sp. n.

***Baconia riouka* group**

*Baconia riouka* (Marseul, 1861)

*Baconia azuripennis* sp. n.

***Baconia famelica* group**

*Baconia famelica* sp. n.

*Baconia grossii* sp. n.

*Baconia redemptor* sp. n.

*Baconia fortis* sp. n.

*Baconia longipes* sp. n.

*Baconia katieae* sp. n.

*Baconia cavifrons* (Lewis, 1893), comb. n.

*Baconia haeterioides* sp. n.

***Baconia micans* group**

*Baconia micans* (Schmidt, 1889a)

*Baconia carinifrons* sp. n.

*Baconia fulgida* (Schmidt, 1889c)

***Baconia* incertae sedis**

*Baconia chilense* (Redtenbacher, 1867)

*Baconia glauca* (Marseul, 1884)

*Baconia coerulea* (Bickhardt, 1917)

*Baconia angulifrons* sp. n.

*Baconia sanguinea* sp. n.

*Baconia viridimicans* (Schmidt, 1893b)

*Baconia nayarita* sp. n.

*Baconia viridis* sp. n.

*Baconia purpurata* sp. n.

*Baconia aenea* sp. n.

*Baconia clemens* sp. n.

*Baconia leivasi* sp. n.

*Baconia atricolor* sp. n.

**Key to species of *Baconia***

**Table:** 

1	Asian species	2
–	American species	10
2	Metaventrite with two parallel lateral striae, the outer variably complete (Figs 49H, 50B); pronotum with coarse punctures sparser across central part of disk (Figs 49E, 50A); widely distributed in SE Asia outside of Japan	3
–	Metaventrite with single lateral stria; pronotum coarsely punctate across entire disk; Japan	*Baconia chujoi* (Cooman)
3	Body metallic	4
–	Body nonmetallic, rufotestaceus to piceous	5
4	Frontal stria absent from central part of frons; body more parallel-sided (Fig. 49E)	*Baconia reposita* sp. n.
–	Frontal stria complete; body broad and subdepressed (Fig. 73C)	*Baconia glauca* (Marseul)
5	Prosternal carinal striae united around prosternal midpoint (Fig. 49H)	6
–	Prosternal carinal striae separate throughout lengths	7
6	Inner lateral metaventral stria straight to apex; 1^st^ abdominal ventrite with 2 lateral striae	*Baconia kubani* sp. n.
–	Inner lateral metaventral stria curved strongly mediad posteriorly (Fig. 50E); 1^st^ abdominal ventrite with single lateral stria	*Baconia adebratti* sp. n.
7	Mesometaventral stria strongly arched forward, interrupting marginal mesoventral stria at middle, non-crenulate (Fig. 50G); mainland SE Asia	8
–	Mesometaventral stria not strongly arched forward, crenulate; Indomalayan region	9
8	First abdominal ventrite with two lateral striae	*Baconia barbarus* (Cooman)
–	First abdominal ventrite with a single lateral stria	*Baconia silvestris* sp. n.
9	Marginal mesoventral stria complete at middle; Indonesia: Sulawesi and Malaysia: Sabah	*Baconia wallacea* sp. n.
–	Marginal mesoventral stria interrupted at middle; Malaysia: Sabah	*Baconia bigemina* sp. n.
10	Lateral mesotibial margin with >5 marginal teeth; metatibia with 2 marginal spines; body elongate, parallel-sided, moderately flattened (Fig. 69A)	*Baconia cavifrons* (Lewis)
–	Lateral mesotibial margin with no more than 3 marginal spines; metatibia lacking marginal spines	11
11	Pygidium with large, round trichomes (Fig. 69F)	*Baconia haeterioides* sp. n.
–	Pygidium lacking trichomes	. 12
12	Body strongly flattened; sutural, and usually 5^th^ elytral striae completely absent	13 (*Baconia loricata* group, in part)
–	Body flattened or not; at least fragments of sutural stria present; 5^th^ elytral stria present or not	20
13	Propygidium with complete stria along basal margin (Fig. 5E)	14
–	Propygidium lacking basal marginal stria	18
14	Body color metallic	15
–	Body not metallic, entirely rufotestaceus (Fig. 3C)	*Baconia patula* Lewis
15	Fifth elytral stria present	16
–	Fifth elytral stria absent	17
16	Frontal stria present (though interrupted) across middle; 4^th^ dorsal stria strongly abbreviated from base (Fig. 3E)	*Baconia gounellei* (Marseul)
–	Frontal stria absent across middle, labrum very short, rounded; 4^th^ dorsal stria more or less complete	*Baconia nebulosa* sp. n.
17	Body very large (>5mm); known only from southern Brazil	*Baconia loricata* Lewis
–	Body smaller (<4mm); known from Costa Rica	*Baconia applanatis* sp. n.
18	Body metallic	19
–	Body rufopiceous to piceous	*Baconia brunnea* sp. n.
19	Larger (>4mm), broader, flatter species (Fig. 9A-B); prosternal carinal striae separated by about the width of profemur, free basally; sides of frontal stria present along inner edge of eye	*Baconia disciformis*sp. n.
–	Smaller species (~3mm) (Fig. 5A); prosternal carinal striae separated by less than width of profemur, united basally; frontal stria completely absent	*Baconia jubaris* (Lewis)
20	Protibia narrow, parallel-sided, with apical and sometimes basal marginal teeth, but rest of margin lacking teeth (Fig. 54F)	21 (*Baconia gibbifer* group)
–	Protibia wider, margin with at least one distinct tooth along middle portion of margin	26
21	Central portion of mesometaventral stria absent (Fig. 54C); lateral striae of 1^st^ abdominal ventrite curving mediad along anterior margin of ventrite, occasionally meeting at midline	*Baconia gibbifer* sp. n.
–	Central portion of mesometaventral stria present; lateral striae of abdominal ventrite 1 not curving mediad along anterior margin of ventrite	22
22	Prosternal keel narrowed anterad (Fig. 54G), becoming subcarinate in anterior one-fourth	23
–	Prosternal keel more or less evenly convex throughout length (Fig. 54H)	24
23	Short anterior fragment of 5^th^ elytral stria present; body more nearly parallel-sided (Fig. 56A), width at elytral apices about two-thirds humeral width	*Baconia tenuipes* sp. n.
–	Fifth elytral stria absent; body strongly narrowed posterad (Fig. 54G), width at elytral apices little more than half humeral width	*Baconia piluliformis* sp. n.
24	Elytral striae abbreviated, none extending into posterior half of elytra; body strongly convex, prosternal lobe densely punctate at sides (Fig. 54H)	*Baconia maquipucunae* sp. n.
–	At least 2^nd^ elytral stria extending into posterior half of elytra; body subdepressed; prosternal lobe not densely punctate at sides	25
25	Basal fragment of 4^th^ elytral stria very short, not extending posterad past basal one-fourth; prescutellar area of pronotal disk impunctate (Fig. 56C); epistoma often with small median tubercle (Fig. 56B); Central America into northwestern South America	*Baconia tuberculifer* sp. n.
–	Basal fragment of 4^th^ elytral stria extending to elytral midpoint (Fig. 56D); prescutellar area of pronotal disk with a few coarse punctures; epistoma lacking median tubercle; South America	*Baconia globosa* sp. n.
26	Body small, elongate and parallel-sided to strongly cylindrical (e.g. Figs 30, 52); body color rufotestaceus to metallic; 5^th^ and sutural striae nearly or fully meeting in narrow anterior arch	27 (*Baconia cylindrica* & *ruficauda* groups, in part)
–	Body larger, rarely subcylindrical; body color varied; 5^th^ and sutural striae not joined in anterior arch (4^th^ and sutural may be)	30
27	Most of dorsum metallic, with rufescent pygidia (Fig. 30A); base of 5^th^ dorsal stria not meeting sutural stria	*Baconia ruficauda* sp. n.
–	Entire dorsum uniformly rufotestaceus; bases of 5^th^ dorsal stria and sutural stria united	28
28	Anterior margin of frons produced as blunt, transverse ridge; epistoma concave beneath; 4^th^ dorsal stria complete	*Baconia teredina* sp. n.
–	Frons and epistoma more or less coplanar, anterior margin of frons not produced; 4^th^ dorsal stria frequently interrupted or obsolete	29
29	Body larger (~1.5mm); 4^th^ elytral stria more or less obsolete; Central America	*Baconia chatzimanolisi* sp. n.
–	Body smaller (~1.3mm); 4^th^ elytral stria present, but may be broadly interrupted; South America	*Baconia cylindrica* sp. n.
30	Body small (<1.8mm), elongate, subquadrate, subdepressed (Figs 32A-D, E, G, 35C); body color rufescent, not metallic, not piceous; 4^th^ dorsal stria meeting sutural stria at subangulate anterior arch	31 (*Baconia angusta* group)
–	Body size varied, but typically larger; body color darker, piceous or metallic; elytral striae varied, but 4^th^ stria rarely joined to sutural stria	39
31	Frons obliquely subcarinate over each antennal base (Fig. 32H)	*Baconia bullifrons* sp. n.
–	Frons more or less flat, not subcarinate over antennal bases	32
32	Dorsum distinctly bicolored, with pronotum darker than rufescent elytra (Fig. 35C); pronotum with coarse punctures sparsely scattered over most of disk, with only very narrow median band impunctate	*Baconia rubripennis* sp. n.
–	Dorsum unicolored; pronotum with coarse punctures, if present, limited to sides, median impunctate band distinctly broader, nearly as wide or wider than head	33
33	Mesofemur with dentate process at posteroapical corner (Fig. 35B)	*Baconia dentipes* sp. n.
–	Mesofemur at most weakly produced at posteroapical corner	34
34	Aedeagus very short and broad, tegmen no longer than twice maximum width (Figs 33E, K); 8^th^ sternite with conspicuous fringe of long setae	35
–	Aedeagus longer, tegmen length generally 3 or more times maximum width; setae of 8^th^ sternite much less conspicuous, may be absent	36
35	Basal piece of aedeagus about two-thirds as long as tegmen (Fig. 33E); spiculum gastrale with digitiform, laterally directed apical processes (Fig. 33D)	*Baconia angusta* Schmidt
–	Basal piece of aedeagus shorter, no more than half tegmen length (Fig. 33K); spiculum gastrale with fine, setiform apical processes (Fig. 33J)	*Baconia incognita* sp. n.
36	Spiculum gastrale with apical emargination relatively shallow, less than half total length of segment (Figs 36E-F)	37
–	Spiculum gastrale with apical emargination relatively deep, more than half total length of segment (Fig. 33N)	38
37	Apex of 9^th^ tergite appearing truncate (Fig. 37C); apex of aedeagus narrower in dorsal view, less strongly curved ventrad (Figs 37E-F)	*Baconia lunatifrons* sp. n.
–	Apex of 9^th^ tergite rounded (Fig. 36C); apex of aedeagus more evenly rounded in dorsal view, strongly curved ventrad (Figs 36I-J)	*Baconia subtilis* sp. n.
38	Basal piece of aedeagus long, meeting tegmen at a nearly right angle (Figs 33O-P); tegmen with little ventral curvature	*Baconia guartela* sp. n.
–	Basal piece of aedeagus short, in line with tegmen; tegmen with significant ventral curvature (Figs 34M-N)	*Baconia cavei* sp. n.
39	Body elongate, parallel-sided, subdepressed, coarsely punctate above (Figs 58, 60); pronotum uniformly punctate; frons strongly punctate, convex; all punctures (especially evident on pygidia) finely setigerous	40 (*Baconia insolita* group)
–	Body shape varied, may be coarsely punctate on various dorsal surfaces, but if so, punctures not setigerous; frons, if densely punctate, then variously depressed at middle	43
40	Pronotum lacking any suggestion of lateral submarginal stria	41
–	Pronotum with lateral submarginal stria composed of series of punctures	42
41	Body color metallic (Fig. 58C); South America	*Baconia burmeisteri* (Marseul)
–	Body not metallic (Fig. 58A); Mexico	*Baconia insolita* (Schmidt)
42	Frontal and especially epistomal punctures coarse; male genitalia with S9 broad (Fig. 61E)	*Baconia pilicauda* sp. n.
–	Frontal and especially epistomal punctures sparser and finer (Fig. 60B); male genitalia with S9 narrower (Fig. 61D)	*Baconia tricolor* sp. n.
43	Body metallic above, may be blue, violet, green, or bronzy	44
–	Body piceous or rufotestaceous, without any hint of metallic coloration	93
44	Dorsal metallic coloration disrupted by rufescent elytral maculations	45
–	Metallic coloration not interrupted by rufescent maculations	46
45	Body larger (~3mm), rounded; red maculations reaching elytral base (Fig. 68A); epistoma produced to form broad transverse ridge above labrum (Fig. 68B)	*Baconia katieae* sp. n.
–	Body smaller (<2mm), elongate; elytra metallic blue at base, red maculations more medial (Fig. 16A); epistoma not produced over labrum	*Baconia maculata* sp. n.
46	Lateral submarginal pronotal stria present (in rare cases may have linear depression with series of punctures weakly coalesced into striae)	47
–	Lateral submarginal pronotal stria absent; pronotal disk not depressed or elevated laterally	63
47	Lateral pronotal bead elevated into distinct submarginal ridge, depressed along its inner edge (e.g. Figs 64A, E, G); body relatively large, elongate, sides subparallel; frons usually depressed at middle	48 (*Baconia famelica* group, in part)
–	Lateral pronotal bead not elevated into submarginal ridge, pronotal disk at most weakly impressed along submarginal stria; body smaller and/or rounder; frons varied	51
48	Central part of frons very deeply impressed, separated from epistoma by distinct carina (Fig. 66C); dorsum conspicuously and more or less uniformly punctate (Fig. 66A); known only from Amazonas, Brazil	*Baconia longipes* sp. n.
–	Central part of frons no more than shallowly impressed, separated from epistoma by only very fine carina or stria; body generally less conspicuously punctate, punctures distinctly finer on central part of pronotal and elytral disks; SE Brazil	49
49	Pronotum and pygidia more or less non-metallic, elytra rather faintly metallic (Fig. 64E); apices of elytral disks obliquely punctatorugose	*Baconia redemptor* sp. n.
–	Pronotum, elytra, and pygidia more or less uniformly metallic	50
50	Frons and epistoma uniformly and continuously punctate (Fig. 64B); epistoma lacking apical transverse microsculpture; western Paraná, Brazil	*Baconia famelica* sp. n.
–	Epistoma distinctly less punctate than frons; epistoma (and labrum) with conspicuous transverse microsculpture along sides and apical margin; eastern Paraná, Brazil	*Baconia grossii* sp. n.
51	Distinct lateral submarginal pronotal stria present	52
–	Lateral submarginal pronotal ‘stria’ comprising only a series of submarginal punctures	61
52	Frontal stria complete	53
–	Frontal stria interrupted, narrowly to broadly	54
53	Propygidium of male depressed on either side of elevated, setose median ridge; pronotum black, with numerous fine lateral punctures (Fig. 62F); elytra metallic blue	*Baconia azuripennis* sp. n.
–	Propygidium of male unmodified; pronotum darker blue than elytra, but not black (Fig. 73D); lateral pronotal punctures fewer, coarser	*Baconia coerulea* (Bickhardt)
54	Frontal stria narrowly interrupted at middle; frons weakly depressed, with few punctures at middle	*Baconia violacea* (Marseul)
–	Frontal stria broadly interrupted at middle; frontal shape varied	55
55	Body large (~3mm), broadly rounded, bright metallic blue; frons depressed at middle	56
–	Body smaller (<2.5mm), elongate, metallic coloration indistinct, barely evident without strong light or magnification; frons flat, frontal stria obsolete between antennal bases	57
56	Frons with strong, well-defined oblique ridges descending onto epistoma, nearly meeting at middle (Fig. 71D)	*Baconia carinifrons* sp. n.
–	Frons with only weakly defined ridges anterad median frontal depression	*Baconia micans* (Schmidt)
57	Violet elytra contrasting distinctly with blue pronotum (Fig. 38C); Cuba	*Baconia pulchella* sp. n.
–	Elytra not violet, though coloration of elytra and pronotum may differ slightly; North and Central America	58
58	Coloration distinct; pronotum blue, elytra grading from blue-green anteriorly to blue posteriorly (Fig. 38A); elytral striae 1-5 more or less complete; USA	*Baconia aeneomicans* (Horn)
–	Coloration very subtle; pronotum blue-black; elytral faintly blue to blue-green	59
59	Marginal mesoventral stria obsolete from most of anterior mesoventral margin, displaced by mesometaventral stria; aedeagus slightly broader, rounded apically	*Baconia quercea* sp. n.
–	Marginal mesoventral stria complete or nearly complete, mesometaventral stria not strongly arched anterad	60
60	Anterior marginal pronotal stria interrupted above eyes; marginal mesoventral stria complete; dorsal punctation sparser (Fig. 40F); prosternum wider, striae subparallel to front; apices of S8 truncate (Fig. 41F)	*Baconia submetallica* sp. n.
–	Anterior marginal pronotal stria continuous along anterior margin (Fig. 40B); marginal mesoventral stria interrupted at middle; dorsal punctation denser (Fig. 40A); prosternal striae weakly convergent to front; apices of S8 subacute (Fig. 39P)	*Baconia irinae* sp. n.
61	Marginal mesoventral stria interrupted at middle; mesometaventral stria arched strongly forward (Fig. 16C)	*Baconia deliberata* sp. n.
–	Marginal mesoventral stria complete (may be fine at middle); mesometaventral stria not so strongly arched forward	62
62	Sutural stria present in posterior one-half elytral length (Fig. 11A); frontal stria narrowly interrupted for about one-half labrum width; known from Central America and extreme northwestern South America	*Baconia godmani* (Lewis)
–	Sutural stria longer, present in posterior two-thirds elytral length (Fig. 16E); frontal stria generally more broadly interrupted, for about labrum width; known from western Amazonia	*Baconia excelsa* sp. n.
63	Propygidium with transverse basal stria	64
–	Propygidium without transverse basal stria	74
64	Coloration bronzy, only faintly blue (Fig. 82C); head large, frons broad, with frontal stria obsolete between antennal bases (Fig. 82D); labrum large, faintly bilobed, with conspicuous transverse microsculpture	*Baconia aenea* sp. n.
–	Coloration not appearing bronzy, blue to violet-blue	65
65	Body large (>2.4mm), rounded, convex (Fig. 71E); male pygidium with elevated setose process in basal half (Fig. 71F); sutural stria complete, connected to 5^th^ stria by basal arch	*Baconia fulgida* (Schmidt)
–	Body size and shape varied; male pygidium unmodified; sutural stria abbreviated to obsolete, not connected to any other stria	66
66	Metallic coloration conspicuous dorsally and ventrally (Figs 11G–H)	*Baconia riehli* (Marseul)
–	Metallic coloration limited to dorsal surface	67
67	Body strongly flattened	68
–	Body moderately to distinctly convex	73
68	At least elytral stria 4 strongly abbreviated from base	69
–	Elytral striae 1-4 more or less complete	70
69	Fourth and fifth elytral striae short but distinctly impressed, 5^th^ stria longer than 4^th^ (Fig. 6D); lateral pronotal punctures shallower and sparser; Panama	*Baconia pernix* sp. n.
–	Fourth and fifth striae little more than apical series of punctures (Fig. 6C); lateral pronotal punctures deeper and denser; French Guiana	*Baconia furtiva*sp. n.
70	Frons irregularly and rather sparsely punctate; northern South America to Central America	71
–	Frons coarsely and uniformly punctate (Fig. 5G); known only from southern Brazil	*Baconia foliosoma* sp. n.
71	Sutural stria well impressed in posterior half; frons depressed at middle, with few coarse punctures between ends of interrupted frontal stria (e.g. Fig. 11F); pygidial punctures distinctly finer in apical half	*Baconia isthmia* sp. n.
–	Sutural stria barely impressed near apex; frons more or less flat; pygidial punctures only barely finer toward apex	72
72	Body strongly depressed (Figs 6A–B), propygidial width about 4×midline length; pygidial punctures fine	*Baconia sapphirina* sp. n.
–	Body subdepressed (Figs 5C–D), propygidial width about 3×midline length; pygidial punctures coarser	*Baconia festiva* (Lewis)
73	Mesoventrite (Fig. 22D) with doubled marginal and mesometaventral striae, the anterior three complete, the posterior-most interrupted at middle; French Guiana, northern Brazil	*Baconia lewisi* Mazur
–	Mesoventrite (Fig. 11D) with single marginal and mesometaventral striae, the marginal mesoventral stria interrupted at middle; USA	*Baconia venusta* (LeConte)
74	Mesometaventral stria present (at least as median fragments), in addition to marginal mesoventral stria (which may be interrupted at middle)	80
–	Mesometaventral stria absent	75
75	Fourth and 5th dorsal striae absent (Fig. 18G), sutural stria largely obsolete	*Baconia jacinta* sp. n.
–	Fourth and/or sutural stria present and distinct	76
76	Fifth and sutural striae absent (Fig. 80C)	*Baconia viridis* sp. n.
–	Sutural, and sometimes 5^th^ striae, present	77
77	Sutural stria complete, well impressed; elytra violet in color, contrasting with blue pronotum and pygidia (Fig. 21E)	*Baconia illustris* (Lewis)
–	Sutural stria obsolete in basal one-fourth to one-half; elytra and pronotum concolorous, generally blue to blue-green, not violet	78
78	Fourth stria variably present in apical half only	*Baconia navarretei* sp. n.
–	Fourth stria variably present in basal half only	79
79	Frons and epistoma both depressed, each with few median punctures (Fig. 80B); frontal stria weakly interrupted between them	*Baconia nayarita* sp. n.
–	Frons depressed, epistoma flat to slightly elevated relative to frons, both impunctate; frontal stria complete (Fig. 18D)	*Baconia eximia* (Lewis)
80	Median portion of mesometaventral stria detached from sides, maybe be reduced or fragmented	81
–	Median portion of mesometaventral stria connected to lateral metaventral stria at sides	85
81	Elytral striae 1-5 distinct, present at elytral base	82
–	At least 5^th^ stria, usually also 4^th^, abbreviated to some degree from base	83
82	Sutural stria more or less complete, inner subhumeral stria strongly abbreviated (Fig. 30B); frons convex, median portion of frontal stria absent	*Baconia repens* sp. n.
–	Sutural stria abbreviated from base (Fig. 18E); inner subhumeral stria more or less complete; frons depressed at middle, median portion of frontal stria fragmented but present (Fig. 18F)	*Baconia splendida* sp. n.
83	Inner subhumeral stria weak, interrupted at middle; mesometaventral stria well developed across middle two-thirds of mesoventral disk	84
–	Inner subhumeral stria more or less complete; mesometaventral stria weak, fragmented, present only at middle of disk (Fig. 14C)	*Baconia rossi* sp. n.
84	Labrum strongly emarginate (Fig. 22A); sutural stria absent	*Baconia choaspites* (Lewis)
–	Labrum weakly emarginate; sutural stria present in apical half	*Baconia scintillans* sp. n.
85	Elytral stria 5 complete to base, may be slightly abbreviated apically (Fig. 82E); frons flat, labrum strongly emarginate (Fig. 82F)	*Baconia clemens* sp. n.
–	Elytral stria 5, if present, strongly abbreviated from base; frons at least slightly depressed at middle; labrum varied, but usually not more than weakly emarginate	86
86	Sutural and 4^th^ elytral striae connected by distinct basal arch (Fig. 21A)	*Baconia prasina* sp. n.
–	4^th^ elytral stria may be arched toward suture, but not connected to sutural stria	87
87	Marginal mesoventral stria interrupted; central portion of frontal stria complete (may be detached at sides), subangulate at middle (Fig. 77B)	*Baconia angulifrons* sp. n.
–	Marginal mesoventral stria complete; central portion of frontal stria complete or interrupted at middle, but not subangulate at middle	88
88	Dorsum and pygidium concolorous (blue, blue-green or violet); body moderately convex	89
–	Pygidia black or testaceus, contrasting with dorsum; body subdepressed	91
89	Sutural stria complete; body color contrasting with elytra violet (Fig. 16F), pronotum and pygidia blueish	*Baconia violacea* (Marseul)
–	Sutural stria abbreviated basally; body more or less unicolorous, varied in color	90
90	Body color blue (Fig. 18B); male S8 weakly widened apically (Fig. 19D)	*Baconia dives* (Marseul)
–	Body color generally more violet (Fig. 18A); male S8 broadly widened apically (Fig. 19B)	*Baconia varicolor* (Marseul)
91	Epistoma not markedly convex, depressed at middle; pygidia and venter black	92
–	Epistoma transversely convex along apical margin, not depressed at middle; pygidia (Figs 77F-G) and venter testaceus	*Baconia viridimicans* (Schmidt)
92	Pronotum and elytra uniformly violet (Fig. 82A); Guianas	*Baconia purpurata* sp. n.
–	Pronotum and elytra blue (Fig. 21D); known only from Costa Rica	*Baconia opulenta* sp. n.
93	Lateral submarginal pronotal stria present	94
–	Lateral submarginal pronotal stria absent	111
94	Frons and epistoma divided by raised carina (Fig. 64H); frons, pronotum, and elytra conspicuously punctate (Fig. 64G); body large (>4mm)	*Baconia fortis* sp. n.
–	Frons and epistoma not divided by raised carina; body with punctures much more restricted in distribution, predominantly impunctate	95
95	Frons with central portion of frontal stria present, may be abbreviated; frons depressed at middle, or deflexed with respect to epistoma	96
–	Central part of frontal stria absent; frons flat, rarely depressed at middle	97
96	Elytra lacking 5^th^ and sutural striae; body strongly convex (Fig. 73A); known only from Chile	*Baconia chilense* (Redtenbacher)
–	Elytra with sutural stria present; body broadly rounded, subdepressed (Fig. 62A); known from southern Brazil, Paraguay	*Baconia riouka* (Marseul)
97	Pronotum conspicuously, almost uniformly punctate (Fig. 38F); known only from south-central to southeastern USA	*Baconia stephani* sp. n.
–	Pronotum punctate at sides only or entirely impunctate; neotropical	98
98	Elytral 5^th^ and sutural striae joined in basal arch (Fig. 84A); striae 1-4 nearly complete, only barely abbreviated apically	*Baconia leivasi* sp. n.
–	Fifth and sutural striae not joined in basal arch; striae 2-4 usually distinctly abbreviated apically	99
99	Fourth dorsal stria bent inward at basal one-third, curved mediad along basal elytral margin (e.g., Figs 40A, C); 5^th^ and sutural striae rather distinctly converging basad; body generally piceous, sides rounded	104
–	Fourth and 5^th^ dorsal striae subparallel to sutural, bending only very weakly mediad at base; body rufescent to rufo-testaceus, generally parallel-sided	100
100	Mesometaventral stria strongly arched anterad, displacing central portion of marginal mesoventral stria (e.g., Fig. 47C); prosternal striae more or less complete, separate throughout their lengths; body more or less unicolorous	101
–	Marginal mesoventral stria complete, not displaced by mesometaventral stria; prosternal striae abbreviated anteriorly, united near prosternal midpoint; pronotum distinctly darker than rufescent elytra (Fig. 77C)	*Baconia sanguinea* sp. n.
101	First abdominal ventrite with two lateral striae	102
–	First abdominal ventrite with single lateral stria	103
102	Body subdepressed, but not strongly flattened; prosternal carinal striae separated by less than width of profemur (Fig. 47C)	*Baconia lescheni* sp. n.
–	Body rather strongly flattened; prosternum broad, the carinal striae separated by more than the width of profemur (Fig. 47F)	*Baconia animata* sp. n.
103	Inner subhumeral elytral stria more or less complete; frons densely and uniformly punctate (Fig. 47D)	*Baconia oblonga* sp. n.
–	Inner subhumeral elytral stria present at extreme base only; frons with dense punctures somewhat irregularly spaced	*Baconia rufescens* sp. n.
104	First abdominal ventrite with at least a few distinct, coarse punctures across anterior part of disk (e.g., Figs 40B, 43F)	106
–	First abdominal ventrite with no more than fine ground punctation on disk	105
105	Fourth abdominal ventrite punctatorugose at center (e.g., Fig. 43F); marginal mesoventral stria interrupted for most of central one-third	*Baconia castanea* sp. n.
–	Fourth abdominal ventrite with only sparse punctures at center (Fig. 40H); marginal mesoventral stria more broadly interrupted, with strial remnants only in extreme corners	*Baconia diminua* sp. n.
106	Body larger (~2.5mm); frontal/epistomal disk distinctly prolonged, total midline length approximately equal to greatest width between eyes; first abdominal ventrite with dense punctures throughout basal one-half to two-thirds (Fig. 40D)	*Baconia fornix* sp. n.
–	Body smaller (<2mm); frontal/epistomal disk not prolonged, midline length distinctly less than maximum interocular width; punctures of first abdominal ventrite generally sparser	107
107	Anterior marginal pronotal stria interrupted behind eyes, ends recurved slightly	108
–	Anterior marginal pronotal stria continuous along anterior margin	111
108	Epistoma densely microsculptured along anterior margin (Fig. 43E)	*Baconia mustax* sp. n.
–	Epistoma without microsculpture along anterior margin	109
109	Protibial margin with distinct median and basal marginal teeth; Central America	*Baconia submetallica* sp. n.
–	Protibial median and basal marginal teeth strongly reduced, margin more or less straight, with only very fine denticles produced beyond the marginal serrulation; South America	110
110	Prosternum relatively narrow, carinal striae separated by less than width of profemur	*Baconia plebeia* sp. n.
–	Prosternum relatively broad, carinal striae separated by more than width of profemur	*Baconia slipinskii* Mazur
111	Body very small (<1.5mm); basal piece longer, about twice as long as wide (Fig. 42M)	*Baconia aulaea* sp. n.
–	Body moderately small (~1.8-2mm); basal piece shorter, about 1.5× as long as wide (Fig. 42P)	*Baconia punctiventer* sp. n.
112	Base of 4^th^ elytral stria present, arched to sutural stria (to which it may or may not be connected; Fig. 24A)	113
–	Base of 4^th^ elytral stria absent	116
113	Body rather strongly depressed; sutural elytral stria strongly reduced, not extending into basal half of elytron (Fig. 84C)	*Baconia atricolor* sp. n.
–	Body more distinctly convex; sutural stria more nearly complete, extending clearly into basal half of elytron, often reaching basal arch of 4^th^ stria	114
114	Epistoma deflexed relative to frons	115
–	Epistoma and frons more or less coplanar (sharing a common median depression; Fig. 24C)	*Baconia salobrus* (Marseul)
115	Labrum deeply emarginate (Fig. 28C); mesometaventral stria transverse, strongly crenulate, coincident with mesometaventral suture; epistoma lacking central fovea	*Baconia emarginata* sp. n.
–	Labrum at most weakly emarginate (Fig. 28A); mesometaventral stria at least slightly arched anterad at middle, departing from mesometaventral suture, less strongly crenulate; epistoma frequently with median fovea	*Baconia anthracina* sp. n.
116	Epistoma no more strongly punctate than frons	*Baconia obsoleta* sp. n.
–	Epistoma distinctly punctate to punctato-rugose (e.g., Fig. 26A)	117
117	Central portion of frontal stria present, complete across middle; epistoma convex, but strongly deflexed; labrum reduced, narrowly rounded apically (Fig. 26A)	*Baconia turgifrons* sp. n.
–	Central portion of frontal stria fragmented to absent, not complete across middle; epistoma less strongly deflexed; labrum not markedly reduced, transverse to weakly emarginate (Fig. 26C)	*Baconia crassa* sp. n.

### Species treatments

#### *Baconia loricata* group

**Figure 4.**
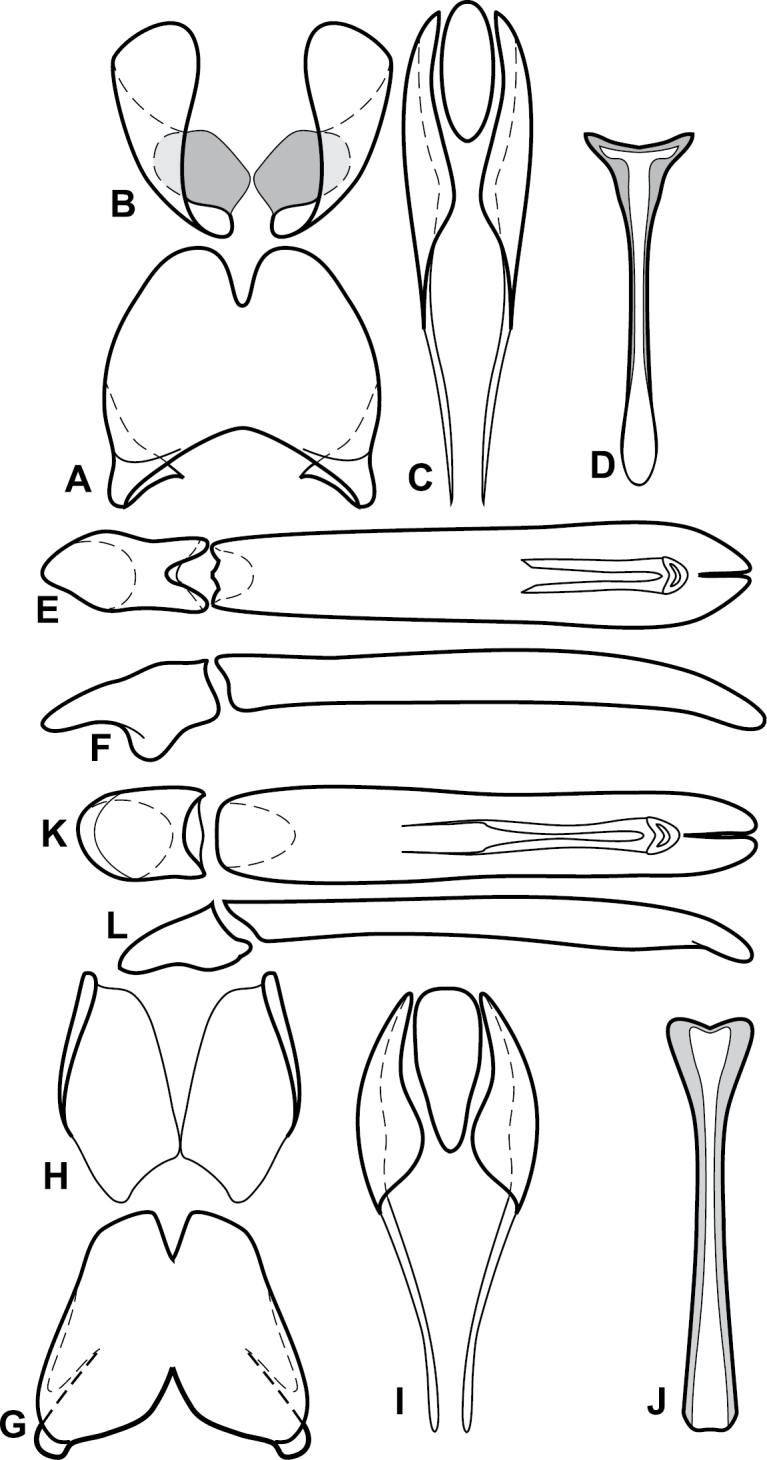
Male genitalia of *Baconia loricata* group. **A–F** *Baconia patula* **A** T8 **B** S8 **C** T9 & T10 **D** S9 **E** Aedeagus, dorsal view **F** Aedeagus, lateral view **G–L** *Baconia foliosoma* **G** T8 **H** S8 **I** T9 & T10 **J** S9 **K** Aedeagus, dorsal view **L** Aedeagus, lateral view.

Species in the *Baconia loricata* group are among the most consistently and strongly flattened species in the genus, and most may be placed here on that basis. However, they also share a few more substantial characters that might suggest that it is somewhat more than a group of convenience, including: apices of male S8 narrowed and extended (Fig. 4H), male S8 usually longer than T8; T9 with long, thin basal apodemes (Fig. 4C). Members of the generally similar *Baconia godmani* group are not only more convex, but also have a male S8 with a conspicuous fringe of apical setae (e.g., Fig. 12B).

##### Baconia loricata

Lewis, 1885

http://species-id.net/wiki/Baconia_loricata

Fig. 3A Map 1

Baconia loricata Lewis, 1885: 463.

###### Type locality.

 BRAZIL: Santa Catarina: Blumenau [26.9°S, 49.0°W].

###### Type material.

**Lectotype,** of undetermined sex, here designated (BMNH): “Blumenau. Amer. mer.” / “*Baconia loricata* Lewis Type” / “George Lewis Coll. B.M.1926-369” / “LECTOTYPE *Baconia loricata* Lewis, M.S.Caterino & A.K.Tishechkin des. 2010”. This species was described from an unspecified number of specimens, and the lectotype designation fixes primary type status on the only known specimen.

###### Diagnostic description.

Length: 4.8mm, width: 4.6mm; body broadly subquadrate, sides weakly rounded, widest at humeri, very strongly flattened, glabrous; dorsum entirely metallic blue, pygidia slightly greenish-blue; frons broad, shallowly depressed at middle, interocular margins weakly convergent dorsad, disk uniformly punctate, punctures separated by about their diameters, frontal stria present along inner margin of eye, bent mediad at front but broadly interrupted medially; epistoma with apical margin distinctly emarginate; labrum about 3×wider than long, distinctly and narrowly emarginate; both mandibles with basal tooth; pronotal sides strongly converging, arcuate to apex, weakly explanate at sides, lateral marginal stria complete around lateral and anterior margins, very fine and close to margin, submarginal stria absent; pronotal disk with only fine ground punctation over median three-fourths of disk, with small, shallowly impressed secondary punctures sparsely interspersed at sides; elytra with single complete epipleural stria, outer subhumeral stria absent, inner subhumeral stria more or less complete, dorsal stria 1 and 2 complete, 2^nd^ stria very fine basally, 3^rd^ stria present in basal half and as a short series of apical punctures, 4^th^ stria represented by few apical fragments, 5^th^ and sutural striae absent; elytral disk with small secondary punctures in apical fifth; prosternal keel broad, weakly convex, base weakly produced, carinal striae more or less complete, separate, subparallel; prosternal lobe about half keel length, apical margin broadly rounded, marginal stria obsolete at sides; mesoventrite broadly and shallowly emarginate, marginal stria interrupted at middle; mesometaventral stria present at middle, detached laterally, inner lateral metaventral stria extending obliquely posterolaterad toward outer corner of metacoxa, abbreviated apically, outer lateral metaventral stria absent, metaventral disk impunctate at middle; protibia narrow, elongate, with three weak marginal teeth, outer margin very finely serrulate between; meso- and metatibiae narrow, each with a single marginal spine about 3/4 of the way towards the apex; pygidia short and wide, propygidium with basal transverse stria present at middle, obsolete at sides; propygidium and pygidium both with medium sized, ocellate punctures, those of propygidium separated by slightly more than their diameters, those of the pygidium becoming smaller and denser, particularly toward apex. Male genitalia: not known.

**Map 1.**
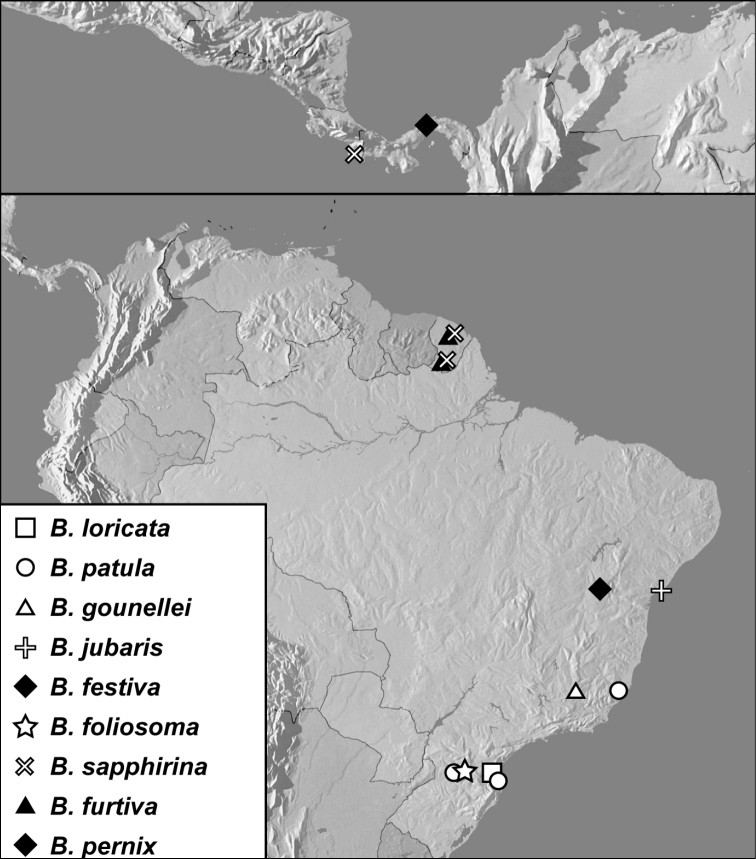
*Baconia loricata* group records. Record for *Baconia festiva* in Bahia, Brazil is a state record only.

###### Remarks.

The type of the genus, *Baconia loricata*, is unfortunately still known only from the type specimen, which appeared too delicate to dissect. It is an unmistakeable species, being nearly twice the size of any other known species, as well as being among the most strongly flattened (Fig. 3A). It appears closely related to *Baconia patula*, sharing many subtle characters, but its size and metallic coloration will distinguish it immediately.

##### Baconia patula

Lewis, 1885

http://species-id.net/wiki/Baconia_patula

Figs 3C–D 4A–F Map 1

Baconia patula Lewis, 1885: 463.

###### Type locality.

BRAZIL: Santa Catarina: Blumenau [26.9°S, 49.0°W].

###### Type material.

**Neotype male** (ZMHB): “Blumenau Bras.” / “*Baconia patula*” / “Caterino/Tishechkin Exosternini Voucher EXO-00442” / “NEOTYPE *Baconia patula* Lewis Desg. M.Caterino & A.Tishechkin, 2011”.

###### Other material.

**BRAZIL**: 1: **Espirito Santo**: Venda Nova do Imigrante, 20°16'S, 41°25'W, xii.2000, FIT, forest, F. Vaz-de-Mello (AKTC); 1: **Santa Catarina**: Blumenau (ZMHB); 1: Nova Teutonia, 10.iv.1957, *Bambusa*, F. Plaumann (FMNH), 3: 10.vi.1960, 5: 11.vi.1960, 2: 11.vii.1957, 3: 12.v.1960, 5: 12.vi.1960, 4: 13.vi.1960, 5: 19.v.1960, 4: 2.vi.1960, 1: 2.vii.1959, 5: 20.v.1960, 2: 20.vi.1960, 8: 21.v.1960, 7: 22.vii.1960, 4: 23.v.1960, 5: 24.v.1960, 5: 25.v.1950, 2: 26.v.1960, 3: 27.v.1960, 4: 28.v.1960, 5: 3.vi.1960, 2: 4.vi.1960, 6: 5.vi.1960, 11: 6.vi.1960, 6: 7.vi.1960, 5: 8.vi.1960, 4: 9.vi.1960 (all: *Bambusa taquara*, F. Plaumann leg., FMNH).

###### Diagnostic description.

Length: 2.0–2.7mm, width: 1.8–2.3mm; body broadly subquadrate, slightly but distinctly widening toward the front, very strongly depressed, glabrous; color rufo-brunneus throughout; frons broad, shallowly depressed at middle, interocular margins weakly convergent dorsad, disk with few sparse median punctures, frontal stria fine, present along inner margin of eye, bent mediad at front, but broadly interrupted medially, supraorbital stria absent; antennal club distinctly elongate, sides subparallel; epistoma with apical margin straight; labrum about 4×wider than long, apically emarginate; both mandibles with basal tooth; pronotal sides almost evenly arcuate to apex, subexplanate at sides, lateral marginal stria complete around lateral and anterior margins, very fine and close to margin, submarginal stria absent; pronotal disk with only fine ground punctation over median three-fourths of disk, with small, shallowly impressed secondary punctures sparsely interspersed at sides; elytra with two complete epipleural striae and fragments of a third, outer subhumeral stria absent, inner subhumeral stria variably impressed in basal half, dorsal stria 1 complete, 2^nd^ stria abbreviated at extreme base, 3^rd^ stria very fine, scratchlike, present in basal half only, 4^th^, 5^th^ and sutural striae absent, elytral disk with very small secondary punctures in apical fifth; prosternal keel broad, weakly convex, base weakly produced, carinal striae more or less complete, may be slightly abbreviated anteriorly, separate, subparallel; prosternal lobe about half keel length, apical margin broadly rounded, marginal stria present, obsolete at sides; mesoventrite broadly and distinctly emarginate, marginal stria complete; mesometaventral stria present at middle, detached laterally, inner lateral metaventral stria continuing from apex of marginal mesoventral stria, extending obliquely posterolaterad toward outer corner of metacoxa, abbreviated apically, outer lateral metaventral stria absent, metaventral disk impunctate at middle; abdominal ventrite 1 with single, complete lateral stria, disk impunctate between; protibia narrow, elongate, with three marginal teeth, outer margin very finely serrulate between; mesotibia with single, inconspicuous subapical spine, and weak, oblique, submarginal carina near midpoint of anterior face; outer metatibial margin smooth; pygidia short and wide, propygidium with transverse basal stria, with moderately large ocellate punctures separated by about their diameters at middle, denser toward sides; propygidial gland openings very small, visible posterad ends of transverse basal stria; pygidium with sparse ground punctation and small secondary punctures evenly interspersed, separated by about their diameters. Male genitalia (Figs 4A-F): T8 broad, sides rounded to apex, basal rim slightly widened, basal emargination shallow, subangulate, apical emargination deep, narrow, ventrolateral apodemes weakly sclerotized, short, opposing, separated by about one-half tegmen width; S8 short, divided, with distinct, stronger ventromedial subsclerotizations, inner edges strongly divergent in apical half, outer margins weakly divergent, apical guides widening to broadly rounded apices, without conspicuous setae; T9 with basal apodemes long, thin, about one-half total length, T9 apices very narrowly rounded, glabrous, ventrolateral apodemes very poorly developed; T10 entire; S9 weakly widened to rounded base, head abruptly widened, sides attenuate, sclerotized along lateral and distal margins, not apically divided; tegmen narrowest near base, widening weakly to near apex, tegmen in lateral aspect rather thick throughout, weakly curved ventrad just at apex; median lobe simple, about one-third tegmen length; basal piece about one-fifth tegmen length, apical emarginations deep.

###### Remarks.

The original type of *Baconia patula* is unfortunately lost. There is an empty point in the BMNH, with labels that unambiguously associate the mount with the original type specimen. However, the specimen has been sought on multiple occasions, by the senior author and by BMNH personnel, and no corresponding specimen could be found. Because of the strong similarity among *Baconia* species the designation of an unambiguous Neotype seemed appropriate. The Neotype bears the same data as the original type, and may in fact be a syntype, although this can only be speculated.

This species is very distinct in its strongly flattened (Fig. 3C), non-metallic, rufobrunneus appearance, its emarginate labrum (Fig. 3B), and weakly convex prosternal keel (Fig. 3D). The association with ‘Bambusa taquara’, is not as specific as it appears, as no species of *Bambusa* currently bears this name, and taquara is generally used as a common name for various bamboos.

##### Baconia gounellei

(Marseul, 1887)

http://species-id.net/wiki/Baconia_gounellei

Figs 3E–F Map 1

Phelister gounellei Marseul, 1887: cxviii; *Baconia gounellei*: Lewis 1901: 372.

###### Type locality.

BRAZIL: Minas Gerais: Caraça [exact locality uncertain].

###### Type material.

**Lectotype, probably female**, here designated (BMNH): “Caraça(Minas Geraez) Bresil E.Gounelle 1.2.1885” / “*Phelister Gounellei* n.sp.” / “Marseul’s Type.” / “George Lewis Coll. B.M.1926-369” / “LECTOTYPE *Phelister gounellei* Marseul, M.S.Caterino & A.K.Tishechkin des. 2010”. This species was described from an unspecified number of specimens, and the lectotype designation fixes primary type status on the only known specimen.

###### Diagnostic description.

Length: [not measured, ~2.5mm], width: [not measured, ~1.5mm]; body broadly subquadrate, sides weakly rounded, widest at humeri, strongly flattened, glabrous; dorsum entirely metallic blue, pronotum and pygidia slightly more greenish-blue; frons slightly depressed at middle, interocular margins weakly convergent dorsad, disk with few coarse punctures at middle, frontal stria present along inner margin of eye, bent mediad at front but broadly interrupted medially; epistoma with apical margin weakly emarginate; labrum about 3×wider than long; pronotal sides converging, arcuate to apex, weakly explanate at sides, lateral marginal stria complete around lateral and anterior margins, fine and close to margin, submarginal stria absent; pronotal disk with only fine ground punctation over median three-fourths of disk, with small, shallowly impressed secondary punctures sparsely interspersed at sides and front; elytra with single complete epipleural stria, outer subhumeral stria absent, inner subhumeral stria present as separate basal and median fragments, dorsal striae 1-2 more or less complete, 3^rd^ stria slightly abbreviated at both ends, 4^th^ and 5^th^ striae represented by few apical fragments, sutural stria absent; elytral disk with small secondary punctures in apical fourth; prosternal keel broad, flat, base weakly produced, carinal striae more or less complete, separate, subparallel; prosternal lobe about two-thirds keel length, apical margin broadly rounded, marginal stria well impressed at middle, obsolete at sides; mesoventrite broadly and shallowly emarginate, marginal stria broadly interrupted at middle, reduced to lateral strioles; mesometaventral stria present at middle, arched strongly forward to near margin, inner lateral metaventral stria sinuately curving posterolaterally toward mesepisternum, abbreviated apically, outer lateral metaventral stria absent, metaventral disk impunctate at middle; protibia rather narrow, elongate, with three weak marginal teeth, outer margin very finely serrulate between; meso- and metatibiae narrow, mesotibia with a single marginal spine, metatibial margin smooth; pygidia short and wide, propygidium with complete basal transverse stria, discal punctures ocellate, medium-sized, separated by a little more than their diameters; pygidial punctures smaller, sparser toward apex. Male genitalia: not known.

###### Remarks.

Because *Baconia gounellei* is known only from the type specimen, which is in relatively poor condition, it is hard to adequately characterize. It appears very close to *Baconia loricata*, but is much smaller and not as flat. Among the highly flattened, metallic species, it is unusual in the presence of the 5^th^ elytral stria, and further distinguished by the presence of fragments of the frontal stria across the anterior margin of the frons. The only other species in this group with the 5^th^ elytral stria present, *Baconia nebulosa*, is completely lacking anterior fragments of the frontal stria, and has very short, unusual mandibles.

##### Baconia jubaris

Lewis, 1901

http://species-id.net/wiki/Baconia_jubaris

Figs 5A–B Map 1

Baconia jubaris Lewis, 1901: 371.

###### Type locality.

BRAZIL: Bahia: San Antonio da Barra [13.0°S, 38.5°W].

###### Type material.

**Lectotype**, sex undetermined, here designated (BMNH): “S.Antonio da Barra, Pr. de Bahia, Gounelle, 11-12.88”/ “*Baconia jubaris* Lewis Type” / “George Lewis Coll. B.M.1926-369.” / “LECTOTYPE *Baconia jubaris* Lewis, M.S.Caterino & A.K.Tishechkin des. 2010”. This species was described from an unspecified number of specimens, and the lectotype designation fixes primary type status on the only known original specimen.

###### Diagnostic description.

Length: [not measured, ~2.5mm], width: [not measured, ~1.5mm]; body broadly subquadrate, sides weakly rounded, widest just behind humeri, strongly flattened, glabrous; dorsum entirely metallic blue, pronotum and pygidia slightly more greenish-blue; frons very weakly depressed at middle, interocular margins weakly convergent dorsad, disk with scattered, mostly fine punctures, few coarser punctures intermingled at middle; frontal stria absent, lacking from inner edge of eyes; epistoma very weakly emarginate; labrum about 3×wider than long, distinctly emarginate apically; each mandible with acute basal tooth on inner margin; pronotal sides converging, arcuate to apex, weakly explanate at sides, lateral marginal stria complete around lateral and anterior margins, fine and close to margin; pronotum very finely and very sparsely punctate throughout, with slightly larger punctures interspersed in lateral sixth; elytra with outer subhumeral absent, inner subhumeral stria more or less complete, 1^st^ dorsal stria complete, 2^nd^ dorsal stria slightly abbreviated basally, 3^rd^ dorsal stria present as fine basal scratch and represented by punctures in apical third, 4^th^, 5^th^ and sutural striae absent; elytral disk with conspicuous secondary punctures in apical fourth; prosternal keel broad, weakly convex, base bisinuate, weakly produced on either side, emarginate medially, carinal striae complete, united along basal margin, subparallel; prosternal lobe about two-thirds keel length, apical margin broadly rounded, marginal stria well impressed at middle; mesoventrite sinuate, broadly emarginate but weakly produced at middle, marginal stria broadly interrupted at middle; mesometaventral stria present at middle, arched strongly forward to near margin; inner lateral metaventral stria sinuately curving posterolaterad toward outer third of metacoxa, nearly complete, outer lateral metaventral stria weakly indicated in anterior third, metaventral disk impunctate at middle; abdominal ventrite 1 with complete inner lateral stria and posterior half of outer lateral stria, disk impunctate at middle; protibia rather narrow, elongate, with five weak marginal teeth, outer margin very finely serrulate between; meso- and metatibiae narrow, mesotibia with two marginal spines; propygidium lacking basal transverse stria, discal punctures ocellate, rather deep, separated by about their diameters; pygidial punctures smaller, sparser toward apex. Male genitalia: not known.

**Figure 5.**
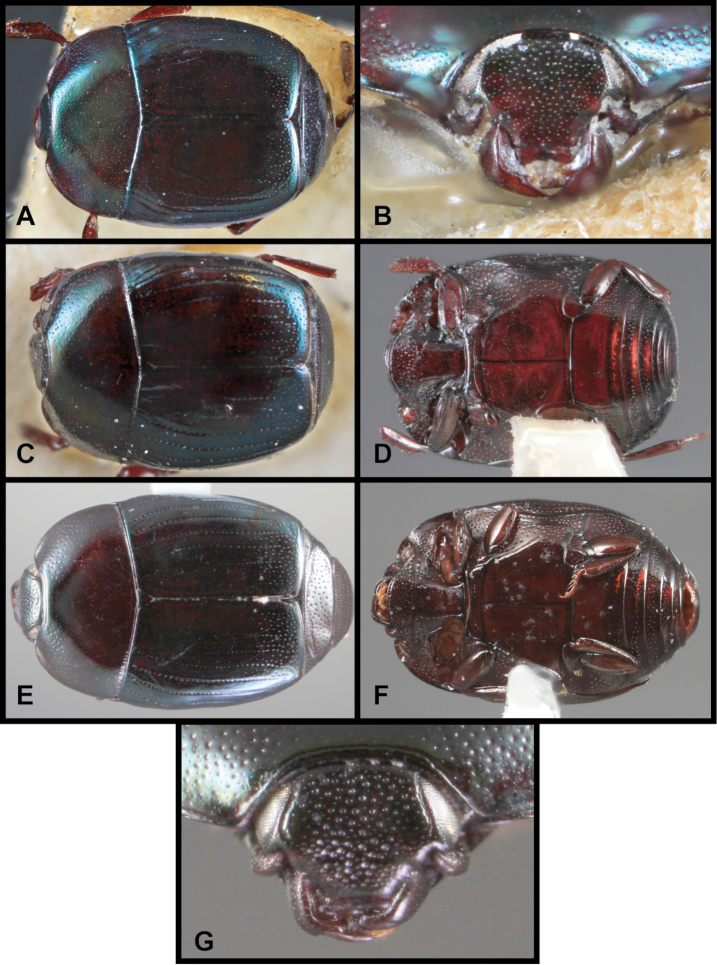
*Baconia loricata* group. **A** Dorsal habitus of lectotype of *Baconia jubaris* **B** Frons of lectotype of *Baconia jubaris* **C** Dorsal habitus oflectotype of *Baconia festiva* **D** Ventral habitus oflectotype of *Baconia festiva* **E** Dorsal habitus of *Baconia foliosoma* **F** Ventral habitus of *Baconia foliosoma* **G** Frons of *Baconia foliosoma*.

###### Remarks.

This species is very similar to the preceding, but can be distinguished by the complete lack of frontal stria (Fig. 5B), even from the inner margins of the eyes, absence of 4^th^ and 5^th^ elytral striae (Fig. 5A), and more nearly complete inner subhumeral stria. It is known only from the type specimen.

##### Baconia festiva

Lewis, 1891

http://species-id.net/wiki/Baconia_festiva

Figs 5C–D Map 1

Baconia festiva Lewis, 1891: 389.

###### Type locality.

BRAZIL: Bahia [exact locality uncertain].

###### Type material.

**Lectotype**, sex undetermined, here designated (BMNH): “Bahia” / “Bahia AG” / “*Baconia festiva* Lewis Type” / “LECTOTYPE *Baconia festiva* Lewis, M.S.Caterino & A.K.Tishechkin des. 2010”. This species was described from an unspecified number of specimens, and the lectotype designation fixes primary type status on the only known original specimen.

###### Diagnostic description.

Length: [not measured, ~2.5mm], width: [not measured, ~1.5mm]; body broadly elongate oval, strongly depressed, glabrous; head, pronotum and pygidia dully metallic greenish-blue, elytra slightly more blue in color, venter rufo-brunneus; frons rather flat, interocular margins weakly convergent dorsad, disk with numerous coarse punctures at middle, frontal stria present along inner margin of eye, curved across middle, interrupted for about half epistomal width; epistoma weakly emarginate apically; labrum about 3×wider than long, apex weakly arcuate; mandibles short; pronotal sides weakly, more or less evenly, curved to anterior corners, disk depressed along anterior fourth of lateral margin, marginal stria complete around lateral and anterior margins, submarginal stria absent, disk with coarse punctures at extreme sides separated by about twice their diameters; elytra with outer subhumeral stria absent, inner subhumeral stria present in about basal one-fifth and for short distance at middle, dorsal striae 1-4 complete, 5^th^ stria present in apical half, sutural stria present as very short apical fragment, elytral disk with scattered secondary punctures in apical fourth; prosternal keel broad, weakly convex, base broadly, weakly produced, carinal striae complete, separate, diverging slightly to front; prosternal lobe about two-thirds keel length, apical margin bluntly rounded, marginal stria obsolete at sides; mesoventrite broadly, shallowly emarginate, marginal stria interrupted for width of emargination; mesometaventral stria arched strongly forward, weakly crenulate, narrowly detached at sides, inner lateral metaventral stria originating close to mesocoxa, extending obliquely posterolaterad toward posterior corner of metepisternum, abbreviated apically, outer lateral metaventral stria briefly indicated at base, metaventral and 1^st^ abdominal disks impunctate at middle; abdominal ventrite 1 with single, complete lateral stria; protibia with four marginal denticles, the basalmost weak, outer margin serrulate between; mesotibia with single fine marginal spine; outer metatibial margin smooth; pygidia short and wide, propygidium with complete transverse basal stria, secondary punctures rather deep, separated by about their diameters or slightly more; pygidium with fine ground punctation and small, sparse secondary punctures more or less uniformly interspersed. Male genitalia: not known.

###### Remarks.

This species is very closely related to several that follow (*Baconia foliosoma*, *Baconia sapphirina*, *Baconia furtiva*, *Baconia pernix*, and *Baconia applanatis*). All are similar in size (~2.5mm), broad, roughly parallel-sided, strongly depressed, and have a basal transverse stria on the propygidium. Most of them are represented by very little material in collections. For example *Baconia festiva* is known only from the type specimen, which does not exactly match anything else studied. It is possible that discovery of more specimens would justify the synonymization of a few of these. However, at present they are distinguishable and mostly allopatric. *Baconia festiva* can be distinguished from the others by the combination of a complete 4^th^ dorsal stria, 5^th^ stria in the apical half of the elytra (Fig. 5C), rather coarse, sparse lateral pronotal punctation, and the generally arcuate mesometaventral stria (Fig. 5D).

##### Baconia foliosoma sp. n.

http://zoobank.org/45C257F1-2963-4C57-84D8-D187007CD0A3

http://species-id.net/wiki/Baconia_foliosoma

Figs 4G–L 5E–G Map 1

###### Type locality.

BRAZIL: Santa Catarina: Nova Teutonia [27.18°S, 52.38°W].

###### Type material.

**Holotype male**: “Brasilien, Nova Teutonia, 27°11'B, 52°23'L Fritz Plaumann, I:2:1949, 3-500 m” / “*Baconia* sp. det.R.Wenzel 19” / “FMNH-INS 0000 069 302” (FMNH).

###### Diagnostic description.

Length: 2.0mm, width: 1.7mm; body elongate oval, strongly depressed, glabrous; head, pronotum and pygidia dully metallic blue, elytra more distinctly colored, venter rufo-brunneus; frons rather flat, weakly depressed at middle, interocular margins weakly convergent dorsad, disk rather coarsely punctate, frontal stria present along inner margin of eye, only faintly indicated, fragmented across middle, supraorbital stria absent; epistoma flat, weakly emarginate apically; labrum about 4×wider than long, apex weakly bisinuate, surface with distinct reticulate microsculpture; mandibles short, only very bluntly dentate; pronotal sides weakly convergent in basal half, arcuate to apex, marginal stria complete around lateral and anterior margins, submarginal stria absent; pronotal disk depressed along anterior fourth of lateral margin, with only fine ground punctation over median two-thirds, with small secondary punctures interspersed at sides; elytra with two complete epipleural striae, outer subhumeral stria absent, inner subhumeral stria impressed, fragmented over most of length, dorsal striae 1-4 complete, 5^th^ stria present in apical two-thirds, sutural stria absent though with few punctures subserially arranged in apical half, elytral disk with scattered secondary punctures in apical fourth; prosternal keel broad, weakly convex, base broadly, weakly produced, carinal striae complete, separate, diverging slightly to front, slightly sinuate; prosternal lobe about two-thirds keel length, apical margin bluntly rounded, marginal stria obsolete at sides; mesoventrite broadly, shallowly emarginate, marginal stria interrupted for width of emargination; mesometaventral stria arched strongly forward, weakly crenulate, detached at sides, inner lateral metaventral stria originating close to mesocoxa, extending obliquely posterolaterad toward posterior corner of metepisternum, abbreviated apically, outer lateral metaventral stria absent, metaventral and 1^st^ abdominal disks impunctate at middle; abdominal ventrite 1 with single, complete lateral stria; protibia with four marginal denticles, the basalmost weak, outer margin serrulate between; mesotibia with single marginal spine; outer metatibial margin smooth; pygidia short and wide, propygidium with complete transverse basal stria, small secondary punctures more or less uniformly separated by slightly more than their diameters; propygidial gland openings present behind transverse stria, about one-fourth from each side; pygidium with fine ground punctation and small, sparse secondary punctures more or less uniformly scattered. Male genitalia (Figs 4G–L): T8 about as long as broad, sides weakly divergent apically, basal emargination abrupt, narrow, subacute, apical emargination narrow, equilateral, with ventrolateral apodemes narrow, separated by about one-half maximum T8 width, extending about midway distad beneath, obsolete in apical half; S8 divided, inner margins evenly divergent to broad, glabrous apices, outer margins subparallel to weakly convergent, apical guides only weakly developed in apical half; T9 with basal apodemes thin, just over half total length, T9 apices narrow, subacute, weakly opposed, glabrous, ventrolateral apodemes very weak, not projecting beneath; S9 weakly widened at base, head only weakly widened, with no development of apicolateral points, desclerotized along midline; tegmen with sides weakly narrowed to apex, undulating slightly, apex bluntly rounded, tegmen more or less straight in lateral aspect; median lobe about two-thirds tegmen length; basal piece about one-fifth tegmen length.

###### Remarks.

*Baconia foliosoma* can be distinguished from others in this complex by its dense frontal punctation (Fig. 5G), absence of sutural stria (Fig. 5E), subquadrate mesometaventral stria (Fig. 5F), and rather small propygidial punctures. It is most similar to *Baconia sapphirina*, which always has at least a small, distinct fragment of the sutural stria present. It is known only from the type.

###### Etymology.

This species’ name refers to its flattened, leaf-like body.

##### Baconia sapphirina sp. n.

http://zoobank.org/9759A345-0424-4627-9C1C-8A5B02301565

http://species-id.net/wiki/Baconia_sapphirina

Figs 6A–B 7 Map 1

###### Type locality.

COSTA RICA: Puntarenas: Osa Peninsula [8.7°N, 83.6°W].

###### Type material.

**Holotype male**: “Rancho Quemado, 200 m, Península de Osa, Prov. Punt., COSTA RICA. 12 a 24 may 1993. A. Gutiérrez. L-S 292500, 511000” / “INBIO CRI001189233” (INBIO). **Paratypes** (3): **FRENCH GUIANA**: 1: Montagne des Chevaux, 4°43'N, 52°24'W, FIT, 13.vi.2009, SEAG [Société entomologique Antilles-Guyane] (MNHN), 1: Belvèdére de Saül, 3°1'22"N, 53°12'34"W, FIT, 17.i.2011, SEAG (CHND), 1: 4.i.2011, FIT, SEAG (MSCC).

###### Diagnostic description.

Length: 1.7–1.9mm, width: 1.5–1.6mm; body subquadrate, strongly depressed, glabrous; head and pronotum metallic greenish-blue, very slightly contrasting with blue elytra and pygidia, venter rufo-brunneus; frons wide, weakly depressed at middle, interocular margins convergent dorsad, ground punctation fine, with coarser punctures at middle and toward vertex, frontal stria present along inner margin of eye, variably fragmented across middle, never complete, supraorbital stria absent; antennal scape short, club elongate, ovoid; epistoma flat, weakly emarginate apically; labrum about 4×wider than long, apex weakly bisinuate; mandibles short, only very bluntly dentate; pronotal sides subparallel in basal half, arcuate to apex, marginal stria complete around lateral and anterior margins, submarginal stria absent; pronotal disk with only fine ground punctation over median two-thirds, with small secondary punctures interspersed at sides; elytra with two complete epipleural striae, outer subhumeral stria absent, inner subhumeral stria impressed at base and often with isolated median fragment, dorsal striae 1-4 complete, 5^th^ stria present in apical two-thirds, sutural stria very short, present in less than apical half, elytral disk with scattered secondary punctures along apical margin; prosternal keel broad, very weakly convex, base broadly produced, carinal striae complete, separate, subparallel, with bases just curved mediad; prosternal lobe about two-thirds keel length, apical margin bluntly rounded, marginal stria slightly fragmented at sides; mesoventrite broadly emarginate, marginal stria interrupted for width of emargination; mesometaventral stria arched forward, slightly sinuate, crenulate, narrowly detached at sides, inner lateral metaventral stria originating close to mesocoxa, extending obliquely posterolaterad toward posterior corner of metepisternum, outer lateral metaventral stria absent, metaventral and 1^st^ abdominal disks impunctate at middle; abdominal ventrite 1 with single, complete lateral stria; protibia with four marginal denticles, the basal-most weak, outer margin serrulate between; mesotibia with single marginal spine; outer metatibial margin smooth; pygidia short and wide, propygidium with complete transverse basal stria, with moderately large, ocellate punctures more or less uniformly separated by slightly less than their diameters; propygidial gland openings present behind transverse stria, about one-fourth from each side; pygidium with rather dense ground punctation in apical half, small secondary punctures more conspicuous in basal half. Male genitalia (Fig. 7): T8 about as long as broad, sides straight to near apex, basal emargination deep, broad, subangulate, apical emargination shallow, narrowly rounded, ventrolateral apodemes weakly sclerotized, basal, opposing, separated by about tegmen width; S8 short, divided, inner edges approximate nearly to apex, outer margins divergent, apical guides widening to broadly rounded apices, without conspicuous setae; T9 with basal apodemes thin, about one-third total length, T9 apices narrowly rounded, glabrous, ventrolateral apodemes poorly developed; T10 entire; stem of S9 very weakly widened to rounded base, widening from midpoint toward apex, apices curving and acuminate, sclerotized along lateral margins, not apically divided; tegmen narrow in basal half, with sides subparallel, widening to spoon-shaped apex, tegmen weakly dorsoventrally flattened, curved ventrad in apical fourth; median lobe simple, about one-third tegmen length; basal piece about one-third tegmen length, apical emarginations deep.

**Figure 6.**
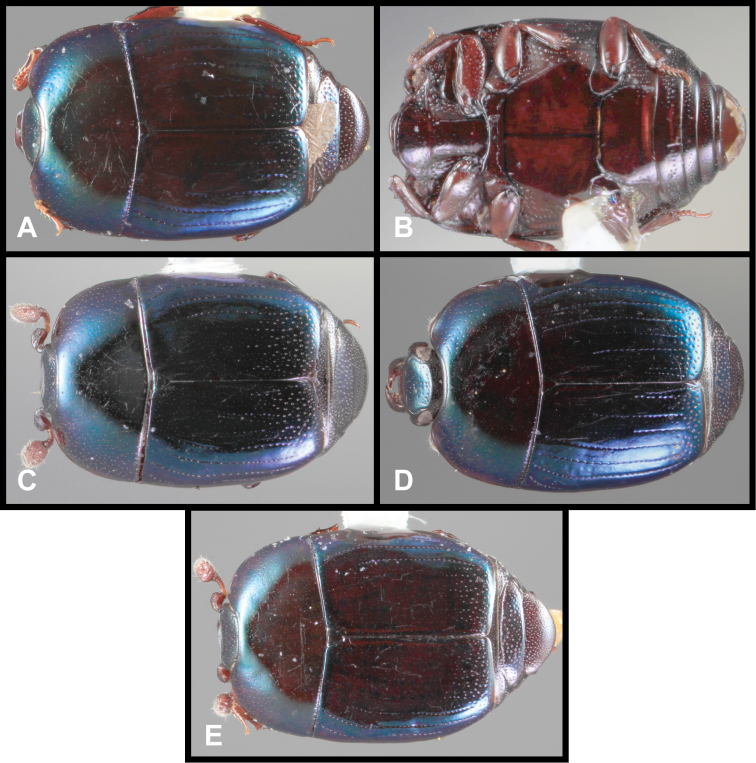
*Baconia loricata* group. **A** Dorsal habitus of *Baconia sapphirina* **B** Ventral habitus of *Baconia sapphirina* **C **Dorsal habitus of *Baconia furtiva* **D** Dorsal habitus of *Baconia pernix* **E** Dorsal habitus of *Baconia applanatis*.

**Figure 7.**
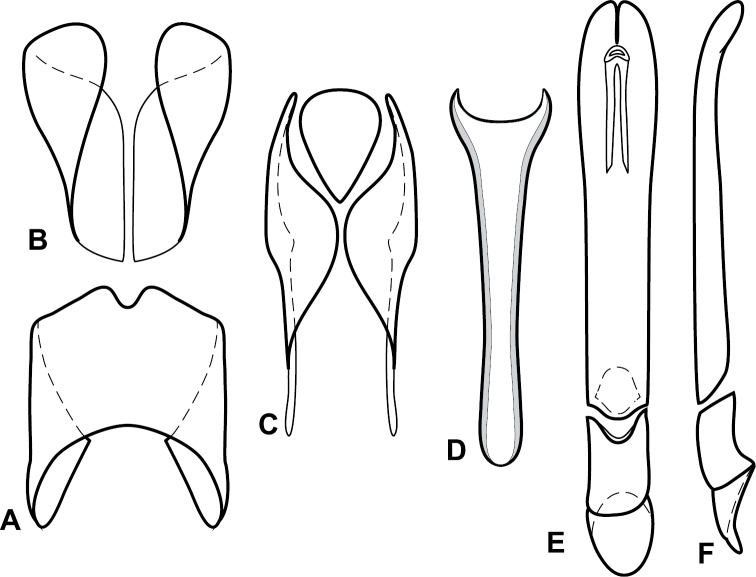
Male genitalia of *Baconia sapphirina*. **A** T8 **B** S8 **C** T9 & T10 **D** S9 **E** Aedeagus, dorsal view **F** Aedeagus, lateral view.

###### Remarks.

Among the species related to *Baconia festiva*, this one is most easily recognized by its very shallow, sparse lateral pronotal punctation (Fig. 6A). In addition its inner subhumeral stria is more nearly complete than that of *Baconia festiva*, and its prosternal carinal striae tend to converge (Fig. 6B) rather than diverging slightly to the front.

###### Etymology.

This species is named for its brilliant, sapphire-like coloration.

##### Baconia furtiva sp. n.

http://zoobank.org/2C070A94-820E-47F4-95EF-4FED7F42CD9B

http://species-id.net/wiki/Baconia_furtiva

Figs 6C 8A–B, G Map 1

###### Type locality.

FRENCH GUIANA: Montagne des Chevaux [4.72°N, 52.40°W].

###### Type material.

**Holotype male**: “**GUYANE FRANÇAISE**:Montagne des Chevaux 4°43'N, 52°24'W Piège d’interception 1 Aou 2009. SEAG leg.” / “Caterino/Tishechkin Exosternini Voucher EXO-00500” (MNHN). **Paratypes** (28): **FRENCH GUIANA**: 1:Montagne des Chevaux, 4°43'N, 52°24'W, 1.viii.2009, FIT, SEAG, 2:11.vii.2009, 4:13.vi.2009, 1:16.v.2009, 2:19.vii.2009, 1:2.v.2009, 3:27.vi.2009, 2:27.vii.2009, FIT, SEAG; 5:6.vi.2009, 4:9.viii.2009, 1: 15.iii.2009; 1: Belvèdére de Saül, 3°1'22"N, 53°12'34"W, 17.i.2011, 1: 20.xii.2010 (all FIT, SEAG leg.; CHND, MSCC, AKTC, FMNH).

###### Diagnostic description.

**Figure 8.**
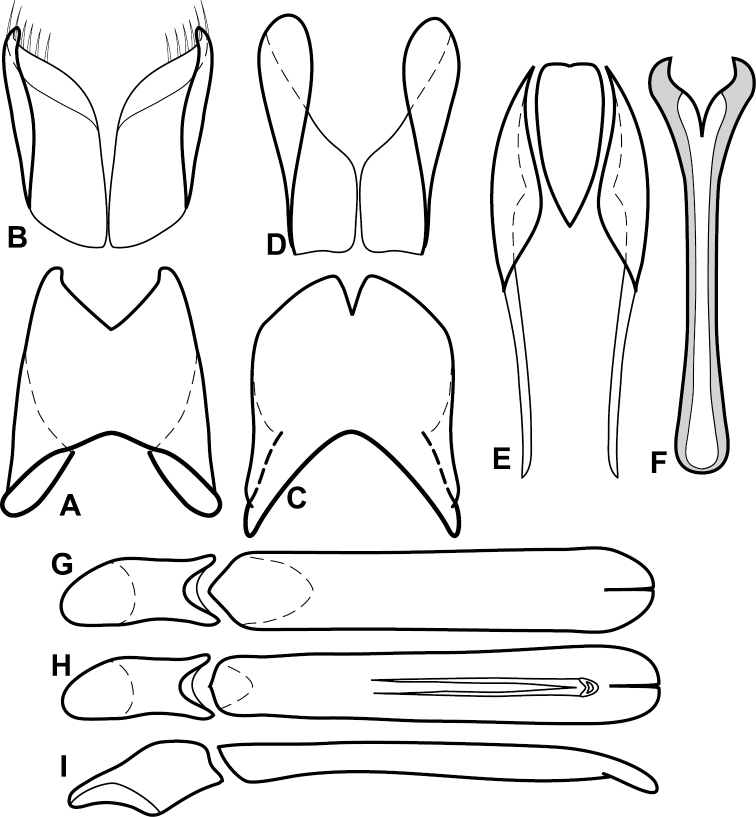
Male genitalia of *Baconia loricata* group. **A** T8 of *Baconia furtiva* **B** S8 of *Baconia furtiva* **C** T8 of *Baconia pernix* **D** S8 of *Baconia pernix* **E** T9 & T10 of *Baconia pernix* **F** S9 of *Baconia pernix* **G** Aedeagus, dorsal view of *Baconia furtiva* **H **Aedeagus, dorsal view of *Baconia pernix* **I** Aedeagus, lateral view of *Baconia pernix*.

Length: 1.9–2.2mm, width: 1.5–1.8mm; body broadly elongate oval, strongly depressed, glabrous; head and pronotum metallic greenish-blue, contrasting slightly with metallic blue elytra and pygidia, venter piceous; frons convex over antennal bases, depressed along midline, ground punctation inconspicuous, with few coarse punctures on epistoma, middle of frontal disk, and toward vertex, frontal stria present along inner margin of eye, curving inward at front, usually interrupted over antennal bases and at middle, supraorbital stria absent; antennal scape short, club asymmetrically oblong; epistoma weakly emarginate apically; labrum about 3×wider than long, weakly emarginate apically; both mandibles with acute basal tooth; pronotal sides increasingly arcuate to apex, marginal stria complete along lateral and anterior margins, lateral submarginal stria absent, pronotal disk narrowly depressed very close to anterior corners, ground punctation of pronotal disk fine, inconspicuous at middle, slightly coarser secondary punctures present in lateral thirds; elytra with two complete epipleural striae, outer subhumeral stria absent, inner subhumeral stria present in basal two-thirds, may be interrupted, dorsal striae 1-3 complete, 3^rd^ stria may be abbreviated apically, 4^th^, 5^th^ and sutural striae only faintly indicated, generally only by serial punctures in apical third, elytral disk with few coarse punctures across apical fourth; prosternum broad, weakly convex, keel truncate to weakly sinuate at base, carinal striae complete, bent mediad at base, rarely united, subparallel to divergent anterad; prosternal lobe about two-thirds keel length, apical margin broadly rounded, marginal stria obsolete at sides; mesoventrite broadly, shallowly emarginate at middle, marginal stria complete; mesometaventral stria absent, inner lateral metaventral stria extending from end of marginal mesoventral stria posterolaterad toward middle of metacoxa, sinuate apically, outer lateral metaventral stria present, parallel to basal two-thirds of inner stria, metaventral disk impunctate at middle; abdominal ventrite 1 with complete inner lateral stria and posterior fragments of outer stria, middle portion of disk lacking coarse punctures; protibia 4-5 dentate, the basal denticles weak, outer margin serrulate between teeth; mesotibia with single marginal spine, subtended by submarginal carina diminishing to base; outer metatibial margin smooth; propygidium with complete transverse basal stria, discal punctures small, ocellate, separated by 1–2× their diameters basally; propygidial gland openings evident behind ends of transverse basal stria, about one-fourth from each lateral margin; pygidium with ground punctation moderately dense, secondary punctation increasingly evident toward base. Male genitalia (Figs 8A–B, G): T8 slightly longer than broad, sides subparallel to weakly convergent apically, basal emargination broad, shallow, weakly acute at middle, basal rim slightly explanate, apical emargination broad, deep, with ventrolateral apodemes separated by about one-half maximum T8 width, extending about one-third distad beneath, tapering to near apex; S8 divided, inner margins approximate at base, weakly divergent to near apex, markedly desclerotized in apical third, bearing conspicuous fringe of setae near apex, outer margins weakly convergent, apical guides narrow but evenly developed along most of sides, narrowly rounded apically; T9 with basal apodemes thin, about half total length, T9 apices narrow, acute, weakly opposed, glabrous, ventrolateral apodemes very weakly projecting beneath; S9 weakly widened at base, head only slightly widened, with apicolateral points curved, horn-like, desclerotized along midline, with narrow apicomedial division; tegmen with sides subparallel to near apex, apical one-fourth weakly bulbous, broadly rounded at apex, dorsobasal edge projecting, tegmen more or less straight in lateral aspect; median lobe about two-thirds tegmen length; basal piece about one-fourth tegmen length.

###### Remarks.

Among the species closely related to *Baconia festiva*, this species can be easily recognized by the presence of only apical fragments of the 4^th^, 5^th^, and sutural elytral striae (Fig. 6C).

###### Etymology.

The name of this species means ‘stealthy’ or ‘furtive’, referring to its presumed habit of stalking subcortical prey.

##### Baconia pernix sp. n.

http://zoobank.org/AA0C1D49-5741-48CB-95D0-ADF4A38B520D

http://species-id.net/wiki/Baconia_pernix

Figs 6D 8C–F, H–I Map 1

###### Type locality.

PANAMA: Canal Zone: Paraiso [9.03°N, 79.62°W]

###### Type material.

**Holotype male**: “Paraiso CZ Pan Feb 9.11 EASchwarz” / “ex. Colln. USNM” / “FMNH-INS 0000 069 303” (FMNH). **Paratype** (1): **PANAMA**: **Colón**: 2 km S Sabanitas, 9°19'19"N, 79°47'54"W, 120 m, 15–19.vii.1999, A. Gillogly & J.B. Woolley (TAMU).

###### Diagnostic description.

Length: 1.8–2.0mm, width: 1.5–1.7mm; body broadly elongate, strongly depressed, glabrous; head and pronotum metallic greenish-blue, contrasting slightly with metallic blue elytra and pygidia, venter rufo-brunneus; frons weakly convex over antennal bases, shallowly depressed along midline, ground punctation fine, few coarse punctures at middle of frontal disk and toward vertex, frontal stria present along inner margin of eye, curving inward at front, interrupted over antennal bases and at middle, supraorbital stria absent; antennal scape short, club oblong; epistoma weakly emarginate apically; labrum about 3×wider than long, weakly bisinuate apically; both mandibles with small basal tooth; pronotal sides subparallel basally, increasingly arcuate to apex, marginal stria complete along lateral and anterior margins, lateral submarginal stria absent, pronotal disk narrowly depressed in anterior corners, ground punctation of pronotal disk fine, inconspicuous at middle, slightly coarser secondary punctures present in lateral fourths; elytra with two complete epipleural striae, outer subhumeral stria absent, inner subhumeral stria present at base, dorsal striae 1-3 complete, 4^th^ stria present in apical fourth, 5^th^ stria longer, present in most of apical half, sutural stria shorter, may be absent, elytral disk with few coarse punctures in apical fifth; prosternum broad, weakly convex, keel outwardly arcuate at base, carinal striae well separated, complete, subparallel anterad; prosternal lobe about two-thirds keel length, apical margin broadly rounded, marginal stria obsolete at sides; mesoventrite broadly, shallowly emarginate at middle, marginal stria broadly interrupted; mesometaventral stria anteriorly arcuate, sinuate at middle, crenulate, narrowly detached at sides, inner lateral metaventral stria extending from inner corner of mesocoxa posterolaterad toward outer third of metacoxa, slightly abbreviated apically, outer lateral metaventral stria absent, metaventral disk impunctate at middle; abdominal ventrite 1 with single, complete lateral stria, middle portion of disk lacking coarse punctures; protibia narrow, 4-dentate, outer margin serrulate between teeth; mesotibia with single marginal spine, outer metatibial margin smooth; propygidium with complete transverse basal stria, discal punctures small, ocellate, separated by 1–2× their diameters; propygidial gland openings inconspicuous; pygidium with ground punctation moderately dense, secondary punctation increasingly evident toward base. Male genitalia (Figs 8C–F, H–I): T8 slightly longer than broad, sides subparallel, basal emargination broad, deep, apical emargination small, narrow, with ventrolateral apodemes short, separated by about two-thirds maximum T8 width, extending about midway distad beneath, obsolete in apical half; S8 divided, inner margins subparallel in basal one-third, evenly divergent to apices, outer margins weakly divergent, apical guides well developed in apical half, broadly rounded apically; T9 with basal apodemes thin, about half total length, T9 apices narrow, acute, weakly opposed, glabrous, ventrolateral apodemes very weakly projecting beneath; S9 weakly widened at base, head only slightly widened, with apicolateral points curved, horn-like, desclerotized along midline, with narrow apicomedial division; tegmen with sides weakly widened to apex, undulating slightly, apex broadly rounded, dorsobasal edge weakly arcuate, tegmen more or less straight in lateral aspect; median lobe about two-thirds tegmen length; basal piece about one-fourth tegmen length.

###### Remarks.

This species can be distinguished from the close relatives above by the short but distinctly impressed 4^th^ and 5^th^ elytral striae (Fig. 6D), with the 5^th^ extending further anterad than the 4^th^. It is very similar to *Baconia furtiva*, above, but in that species these striae are little more than apical series of punctures. The lateral pronotal punctures of *Baconia pernix* are also shallower and sparser.

###### Etymology.

This species name means ‘active’.

##### Baconia applanatis sp. n.

http://zoobank.org/act:93F8D5E8-DCEB-4FBB-AB84-A5234C378214

http://species-id.net/wiki/Baconia_applanatis

Fig. 6E Map 2

###### Type locality.

COSTA RICA: Guanacaste: Sta. Rosa National Park [10.3°N, 85.62°W].

###### Type material.

**Holotype female**: “Est. Sta. Rosa, 300m, P.N. Sta. Rosa, Prov. Guanacaste, Costa Rica, 3 a 12 jun 1992, III curso Parataxon. L-N 313000,359800” / “INBIO CRI000427988” (INBIO). **Paratype female** (1): **COSTA RICA: Guanacaste:** 4 km SSW Guayabo, 1600 ft, 4.vii.1993, M.S. Caterino (MSCC).

###### Other material.

1: **FRENCH GUIANA:** Montagne des Chevaux, 4°43'N, 52°24'W, FIT, SEAG (CHND).

###### Diagnostic description.

Length: 2.1–2.2mm, width: 1.7–1.8mm; body broadly subquadrate, strongly depressed, glabrous; head and pronotum metallic greenish-blue, elytra and pygidia metallic blue, contrasting slightly with pronotum dorsally, venter rufo-brunneus; frons wide, very weakly depressed at middle, interocular margins convergent dorsad, ground punctation fine, with few coarser punctures at middle and toward vertex, frontal stria present along inner margin of eye, narrowly interrupted over antennal bases and broadly interrupted across middle, median fragments may be very weak or absent, supraorbital stria absent; antennal scape short, apex obliquely truncate, club asymmetrically oblong; epistoma truncate apically; labrum about 3×wider than long, apex weakly bisinuate; mandibles short, each with acute basal tooth; pronotal sides almost evenly arcuate to apex, lateral marginal stria complete around lateral and anterior margins, submarginal stria absent, pronotal disk with only fine ground punctation over median three-fourths of disk, with small secondary punctures interspersed only at sides; elytra with two complete epipleural striae, outer subhumeral stria absent, inner subhumeral stria may be impressed over much of its length, but generally fine, fragmented, rarely absent, dorsal striae 1-2 more or less complete, 2^nd^ may be slightly abbreviated basally, 3^rd^ stria very fine, scratchlike, present in basal half only, 4^th^, 5^th^ and sutural striae absent, elytral disk with scattered secondary punctures in apical one-third; prosternal keel broad, flat, very weakly emarginate at base, carinal striae complete, separate, sinuate between coxae, subparallel anterad; prosternal lobe about one-half keel length, apical margin rounded, marginal stria present at middle, fragmented to sides; mesoventrite weakly produced at middle, marginal stria complete; mesometaventral stria absent, inner lateral metaventral stria originating close to mesocoxa, curving posterolaterad toward outer third of metacoxa, outer lateral metaventral stria absent, metaventral and 1^st^ abdominal disks impunctate at middle; abdominal ventrite 1 with complete inner lateral stria and posterior fragment of outer stria; protibia with three marginal denticles, plus a very small basal spine, outer margin serrulate between spines; mesotibia with single marginal spine, a subcarinate ridge extending to it from base; outer metatibial margin smooth; pygidia short and wide, propygidium with complete transverse basal stria, coarse secondary punctures irregularly separated by their diameters or less; propygidial gland openings inconspicuous; pygidium with sparse ground punctation and small secondary punctures densely interspersed. Male: not known.

**Map 2.**
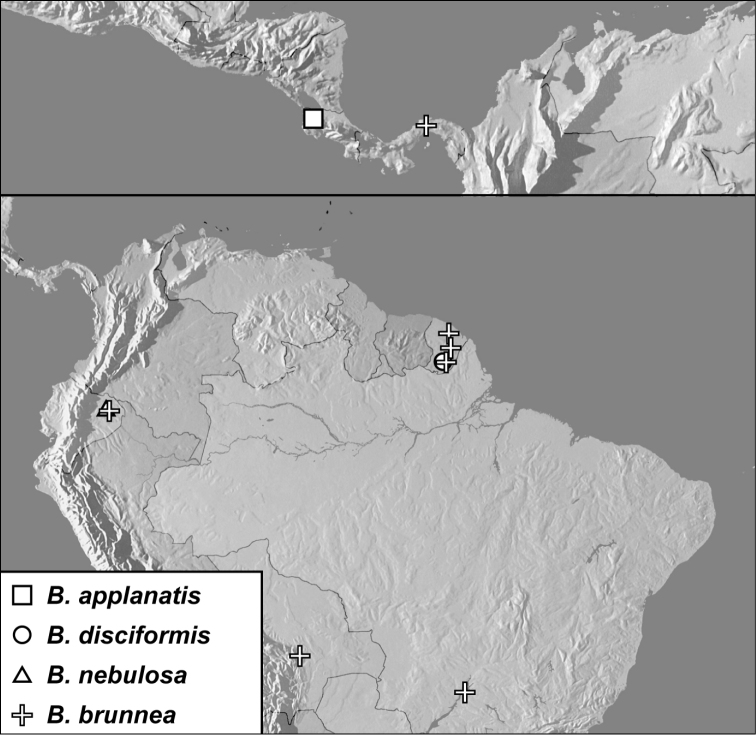
*Baconia loricata* group records.

###### Remarks.

This strongly depressed species is similar in size to the preceding several species, but may be easily distinguished by the complete absence of the 4^th^, 5^th^, and sutural elytral striae (Fig. 6E). The 3^rd^ stria is also strongly abbreviated posteriorly, represented by only a fine stria in the basal half.

###### Etymology.

This species’ name refers to its strongly flattened body form.

##### Baconia disciformis sp. n.

http://zoobank.org/574F16EB-2EB1-4AA6-BE86-04C10353ABC9

http://species-id.net/wiki/Baconia_disciformis

Figs 9A–D Map 2

###### Type locality.

FRENCH GUIANA: Belvèdére de Saül [3.01°N, 53.21°W].

###### Type material.

**Holotype female**: “**GUYANE FRANÇAISE**: Belvèdére de Saül, point de vue. 3°1'22"N, 53°12'34"W. Piège vitre 20.xii.2010. SEAG leg.” / “Caterino/Tishechkin Exosternini Voucher EXO-01292” (MNHN).

###### Diagnostic description.

Length: 2.6mm, width: 2.5mm; body broadly subquadrate, strongly depressed, glabrous; dorsum uniformly metallic blue, venter piceous; frons depressed at middle, interocular margins weakly convergent dorsad, coarsely punctate throughout, more densely toward vertex, frontal stria present along inner margin of eye, absent across front, supraorbital stria absent; antennal scape short, club distinctly elongate, sides subparallel; epistoma transversely elevated along apical margin; labrum about 3×wider than long, apically emarginate; both mandibles with basal tooth, that on right mandible rather weak; pronotal sides almost evenly arcuate to apex, lateral marginal stria complete around lateral and anterior margins, close to margin and non-crenulate, submarginal stria absent; pronotal disk rather linearly depressed along anterior third of lateral margin, with only fine ground punctation over median three-fourths of disk, with small, shallowly impressed secondary punctures interspersed at sides; elytra with two complete epipleural striae, outer subhumeral stria absent, inner nearly complete, only slightly abbreviated apically, dorsal stria 1 complete, 2^nd^ stria obsolete in basal third, 3^rd^ stria very fine, scratchlike, present in basal half only, 4^th^, 5^th^ and sutural striae absent, elytral disk with very small secondary punctures just along apical margin; prosternal keel broad, flat, base more or less truncate, carinal striae complete, separate, weakly convergent anterad; prosternal lobe about two-thirds keel length, apical margin narrowly rounded, marginal stria present obsolete at sides; mesoventrite broadly and weakly emarginate, marginal stria interrupted for width of prosternal keel; mesometaventral stria absent, though suture is evident, arched anterad, inner lateral metaventral stria originating close to mesocoxa, curving posterolaterad toward outer corner of metacoxa, recurved mediad at apex, outer lateral metaventral stria present, close and parallel to inner stria for about two-thirds its length, metaventral disk impunctate at middle; abdominal ventrite 1 with complete inner lateral stria and posterior fragment of outer stria, disk impunctate between; protibia narrow, elongate, with three well developed marginal teeth, outer margin very finely serrulate between; mesotibia with single, inconspicuous submarginal spine; outer metatibial margin smooth; pygidia short and wide, propygidium without transverse basal stria, small secondary punctures concentrated along base and at sides; propygidial gland openings very small, located about one-third from anterior margin, and one-fifth from lateral corner; pygidium with sparse ground punctation and small secondary punctures evenly, sparsely interspersed. Male: not known.

**Figure 9.**
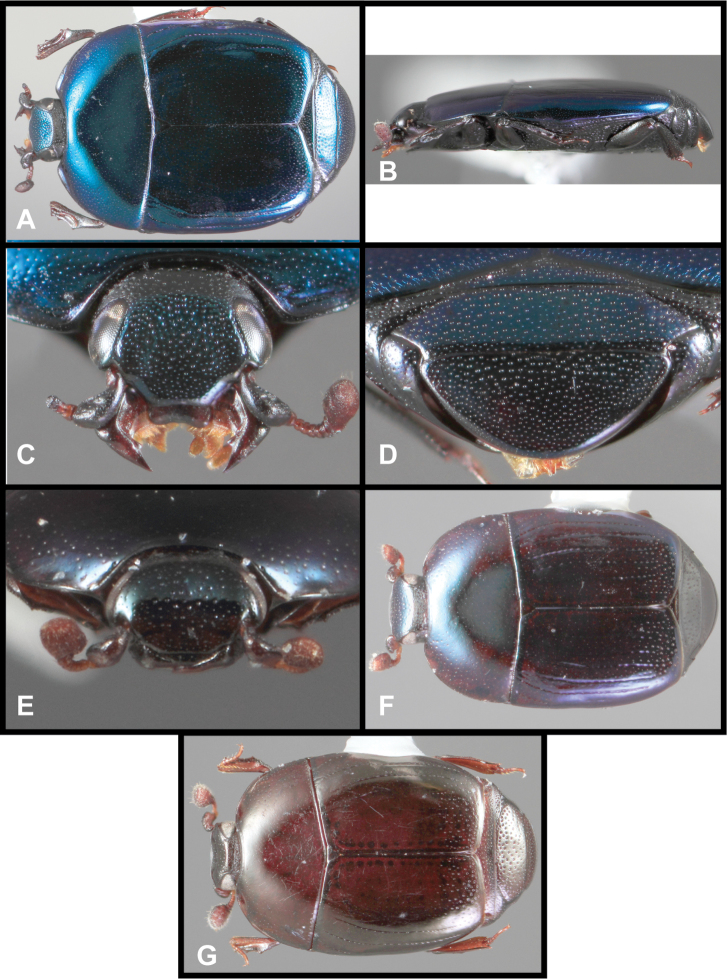
*Baconia loricata* group. **A** Dorsal habitus of *Baconia disciformis* **B** Lateral habitus of *Baconia disciformis* **C **Frons of *Baconia disciformis* **D** Pygidia of *Baconia disciformis* **E** Frons of *Baconia nebulosa* **F** Dorsal habitus of *Baconia nebulosa* **G** Dorsal habitus of *Baconia brunnea*.

###### Remarks.

This species is second in size only to *Baconia loricata*, but is still significantly smaller than that species. It may be further distinguished by its lack of transverse propygidial stria, densely punctate frons, and elongate legs, particularly the protibia (Figs 9A–D).

###### Etymology.

This species’ name refers to its large, flattened disc-like body.

##### Baconia nebulosa sp. n.

http://zoobank.org/187396B9-BB55-4FE1-9CFD-DEC90D13E188

http://species-id.net/wiki/Baconia_nebulosa

Figs 9E–F 10A–D, I–J Map 2

###### Type locality.

ECUADOR: Orellana: Tiputini Biodiversity Station [0.635°S, 76.150°W].

###### Type material.

**Holotype male**: “**ECUADOR: Depto.** **Orellana**, Tiputini Biodiversity Station, 0°37'55"S, 76°08'39"W, 220-250m, 30 June 1998, T.L.Erwin et al. collectors” / “fogging, bare green leaves, some with covering of lichenous or bryophytic plants in terra firme forest, **Lot 1821 Trans. 3 Sta. 2**” / “Caterino/Tishechkin Exosternini Voucher EXO-00436” (USNM).

###### Diagnostic description.

**Figure 10.**
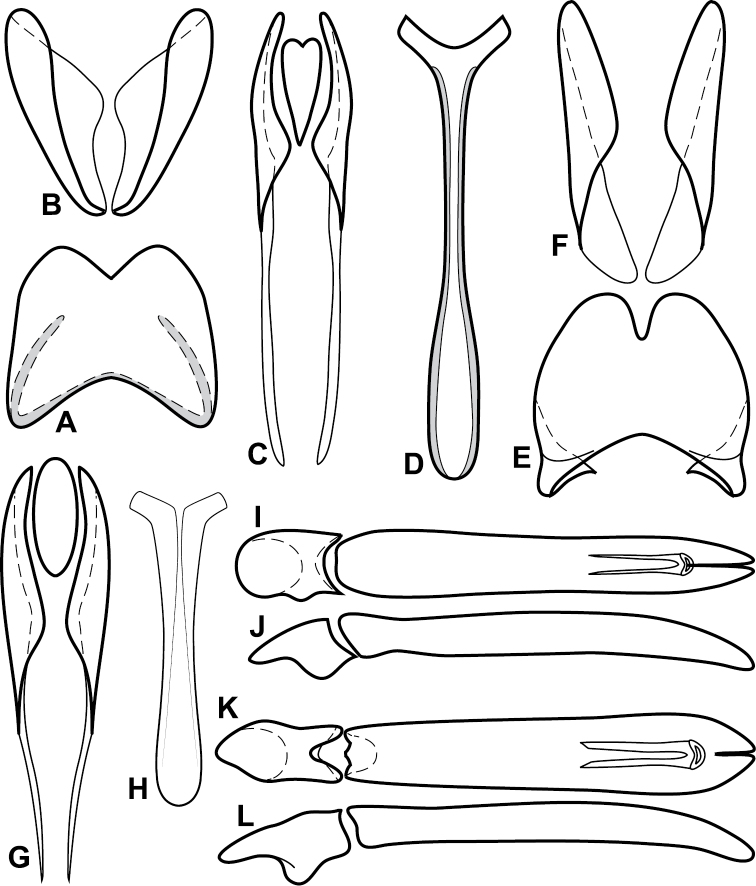
Male genitalia of *Baconia loricata* group. **A** T8 of *Baconia nebulosa* **B** S8 of *Baconia nebulosa* **C** T9 & T10 of *Baconia nebulosa* **D** S9 of *Baconia nebulosa* **E** T8 of *Baconia brunnea* **F** S8 of *Baconia brunnea* **G** T9 & T10 of *Baconia brunnea* **H **S9 of *Baconia brunnea* **I** Aedeagus, dorsal view of *Baconia nebulosa* **J** Aedeagus, dorsal view of *Baconia nebulosa* **K **Aedeagus, dorsal view of *Baconia brunnea* **L** Aedeagus, lateral view of *Baconia brunnea*.

Length: 1.5mm, width: 1.2mm; body broadly subquadrate, moderately strongly depressed, glabrous; head, pronotum and elytra metallic blue, venter and pygidia rufo-brunneus; frons very wide, short, very weakly depressed across middle, interocular margins convergent dorsad, frontal disk with numerous coarser punctures at middle, frontal and supraorbital striae absent; antennal scape short, apex obliquely truncate, club asymmetrically oblong; epistoma truncate apically; labrum very short, about 4×wider than long, apex outwardly arcuate; mandibles extremely short, mostly concealed in repose, dentation not observed in type; pronotal sides subparallel in basal third, evenly arcuate to apices, lateral marginal stria complete around lateral and anterior margins, submarginal stria absent, pronotal disk with only fine ground punctation over median third of disk, with small secondary punctures interspersed at sides; elytra with two complete epipleural striae and fragments of a third, outer subhumeral stria absent, inner subhumeral stria present in basal fourth, dorsal striae 1-4 complete, 5^th^ stria present in apical three-fourths, sutural stria absent, elytral disk with scattered secondary punctures in apical fifth; prosternal keel very broad, weakly convex, slightly outwardly produced at base, carinal striae complete, diverging from base to apex; prosternal lobe about one-half keel length, apical margin bluntly rounded, marginal stria present at middle, fragmented to sides; mesoventrite shallowly and broadly emarginate, marginal stria broadly interrupted; mesometaventral stria isolated at middle, arched strongly forward; inner lateral metaventral stria originating close to mesocoxa, extending posterolaterad toward outer corner of metacoxa, abbreviated apically, outer lateral metaventral stria absent; metaventral and 1^st^ abdominal disks impunctate at middle, median metaventral suture rather deeply impressed; abdominal ventrite 1 with complete inner lateral stria and posterior fragment of outer stria; protibia with three weak marginal denticles, outer margin serrulate between spines; mesotibia with single marginal spine; outer metatibial margin smooth; pygidia short and wide, propygidium with complete transverse basal stria, coarse secondary punctures sparse at middle, slightly denser laterad; propygidial gland openings inconspicuous; pygidium with rather dense ground punctation in apical half, small coarse puncture more evident in basal half. Male genitalia (Figs 10A–D, I–J): T8 about as long as broad, sides weakly convergent to apex, basal emargination evenly arcuate, apical emargination deep, acute, ventrolateral apodemes well sclerotized, extending just beyond midline beneath, separated by about one-half tegmen width; S8 short, divided, inner edges sinuate, approximate beyond midpoint, outer margins weakly divergent, apical guides widening to rounded apices, without conspicuous setae; T9 with basal apodemes long, thin, about two-thirds total length, T9 apices very narrowly rounded, glabrous, ventrolateral apodemes very poorly developed; T10 entire; S9 widened to rounded base, head abruptly widened, sides subquadrate, sclerotized along lateral margins, not apically divided; tegmen narrowest near base, weakly constricted at middle, apices narrow, subacute, tegmen in lateral aspect rather thick throughout, only very weakly curved dorsoventrally; median lobe simple, about one-fourth tegmen length; basal piece about one-fifth tegmen length.

###### Remarks.

Among the strongly depressed, metallic species lacking a sutural stria, *Baconia nebulosa* can be distinguished by its small size, and by its short, wide head and peculiar, mostly concealed mandibles (Fig. 9E).

###### Etymology.

The name of this species means ‘foggy’, referring mainly to the fact that it was collected by fogging the canopy of lowland forest.

##### Baconia brunnea sp. n.

http://zoobank.org/5DCE41DD-1BB3-47C3-BC0C-CE619C22C431

http://species-id.net/wiki/Baconia_brunnea

Figs 9G 10E–H, K–L Map 2

###### Type locality.

FRENCH GUIANA: Montagne des Chevaux [4.72°N, 52.40°W].

###### Type material.

**Holotype male**: **GUYANE FR.,** Montagne des Chevaux, 4°43'N, 52°24'W Piège d’interception 16 May 2009. SEAG leg.” / “Caterino/Tishechkin Exosternini Voucher EXO-00441” (MNHN). **Paratypes** (8): 1: **FRENCH GUIANA**: Montagne des Chevaux, 4°43'N, 52°24'W, Piège d’interception, 26.xii.2008 (CHND), 1: 4.i.2009 (MSCC), 1: 23.ii.2009 (AKTC), 1: 25.iv.2009 (FMNH); 1:Rés. des Nouragues, Régina, 4°2.27'N, 52°40.35'W, 28.i.2010, FIT, SEAG (CHND); 1: Belvèdére de Saül, 3°1'22"N, 53°12'34"W, 20.xii.2010, SEAG (CHND), 1: 21.iv.2011, SEAG (FMNH); 1:Route Nac. 1., P.k. 2, 4°53.5'N, 52°21'W, 20.ix.2008, FIT, J. Touroult (MNHN).

###### Other material.

**BOLIVIA**, 1: **Santa Cruz**:Amboro National Park, Los Volcanes, 18°06'S, 63°36'W, 1000 m, 20.xi–12.xii.2004, FIT, H. Mendel & M. Barclay (BMNH). **BRAZIL**: 1: **Mato Grosso do Sul**: cerradão fragment nr. Selviria, 20°20'10"S, 51°24'36"W, 1.5 m, 11.xii.2010, FIT, C. Flechtmann, 1: 1.5 m, 21.xii.2010, FIT, C. Flechtmann, 1: 28.i.2011, FIT, ground level trail, C. Flechtmann, 1: 30.x.2010, FIT, ground level trail, C. Flechtmann (MEFEIS, FMNH). **ECUADOR**:1: **Orellana**: Est. Biodiv. Tiputini, 0.6376°N, 76.1499°W, 2–9.vi.2011, under bark, M. Caterino & A. Tishechkin, DNA Extract MSC-2128, EXO-00630 (MSCC). **PANAMA**:1: **Colón**: 14 km N jct. Escobal & Pina Rds., 2–11.vi.1996, FIT, J. Ashe & R. Brooks (SEMC); 1: **Panamá**: Barro Colorado Island, 9°11'N, 79°51'W, 23–27.vii.2000, FIT, S. Chatzimanolis (SEMC).

###### Diagnostic description.

Length: 1.9–2.3mm, width: 1.–1.9mm; body broadly subquadrate, strongly depressed, glabrous; color rufo-brunneus to rufo-piceous; frons broad, weakly depressed along midline, interocular margins convergent dorsad, ground punctation fine, with few coarser punctures at middle and toward vertex, frontal and supraorbital striae absent; antennal scape short, apex obliquely truncate, club asymmetrically oblong; epistoma truncate apically; labrum about 3×wider than long, apex weakly bisinuate; both mandibles with acute basal tooth; pronotum weakly convergent in basal two-thirds, rounded to apex, lateral marginal stria complete around lateral and anterior margins, submarginal stria absent, pronotal disk with only fine ground punctation over median three-fourths of disk, with small secondary punctures interspersed only at sides; elytra with two complete epipleural striae, outer subhumeral stria absent, inner subhumeral stria finely impressed at base, rarely also at middle, dorsal striae 1–2 more or less complete, 2^nd^ may be slightly abbreviated basally, 3^rd^ stria very fine, scratchlike, present in basal half only, 4^th^, 5^th^ and sutural striae absent, elytral disk with scattered secondary punctures in apical one-third; prosternal keel moderately broad, weakly convex, weakly emarginate at base, carinal striae complete, separate, subparallel; prosternal lobe about two-thirds keel length, apical margin rounded, with marginal stria present at middle; mesoventrite weakly produced at middle, marginal stria interrupted for width of prosternal keel; mesometaventral stria strongly arched forward, slightly detached at sides from lateral metaventral stria, which extends sinuately and obliquely toward outer third of metacoxa, metaventral and 1^st^ abdominal disks impunctate at middle; abdominal ventrite 1 with complete inner lateral stria, and posterior fragment of outer; protibia 4-dentate, outer margin serrulate between marginal spines; mesofemur with transverse apical series of punctures, subcontinuous with posterior marginal stria; mesotibia with single marginal spine, a subcarinate series of setigerous punctures extending from tibial base to base of marginal spine; outer metatibial margin smooth; propygidium lacking basal stria, coarse secondary punctures rather dense along base and sides, sparser at middle, propygidial gland openings evident, located about midway behind anterior margin, about one-fourth width from each lateral margin; pygidium with sparse ground punctation and small secondary punctures evenly but sparsely interspersed. Male genitalia (Figs 10E-H, K-L): T8 broad, sides rounded to apex, basal rim slightly widened, basal emargination shallow, subangulate, apical emargination deep, narrow, ventrolateral apodemes weakly sclerotized, basal, opposing, separated by about one-half tegmen width; S8 elongate, divided, approximate at base, inner margins strongly and evenly divergent toward apex, outer margins divergent, apical guides widest near middle, narrowing to rounded apices, without conspicuous setae; T9 with basal apodemes long, thin, about one-half total length, T9 apices very narrowly rounded, glabrous, ventrolateral apodemes very poorly developed; T10 entire; S9 weakly widened to rounded base, head abruptly widened, sides obliquely subquadrate, deeply divided apically, nearly full length of sclerite; tegmen narrowest near base, widening weakly to near apex, tegmen in lateral aspect rather thick throughout, weakly curved ventrad just at apex; median lobe simple, about one-third tegmen length; basal piece about one-fifth tegmen length, apical emarginations deep.

###### Remarks.

As a strongly depressed, non-metallic species lacking 4^th^, 5^th^ and sutural elytral striae (Fig. 9G), this species could only be confused with *Baconia patula*. The latter is, however, even more strongly flattened, and the body widens toward the front, whereas in *Baconia brunnea*, the body is clearly widest at the humeri. *Baconia brunnea* also lacks a basal propygidial stria. Due to some variation in elytral striation we restrict the type series to those specimens from French Guiana.

###### Etymology.

The name of this species refers to its non-metallic coloration.

#### *Baconia godmani* group

**Figure 11.**
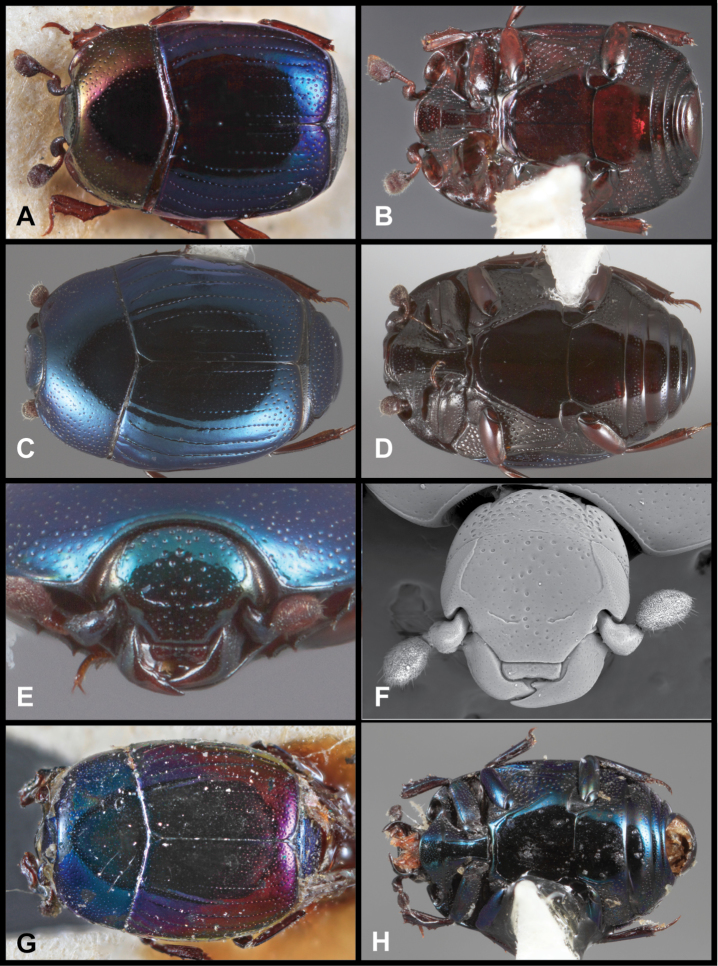
*Baconia godmani* group. **A** Dorsal habitus of lectotype of *Baconia godmani* **B** Ventral habitus of lectotype of *Baconia godmani* **C** Dorsal habitus of *Baconia venusta* **D** Ventral habitus of *Baconia venusta* **E** Frons of *Baconia venusta* **F** Frons (SEM) of *Baconia venusta* **G** Dorsal habitus oflectotype of *Baconia riehli* **H** Ventral habitus of *Baconia riehli*.

**Figure 12.**
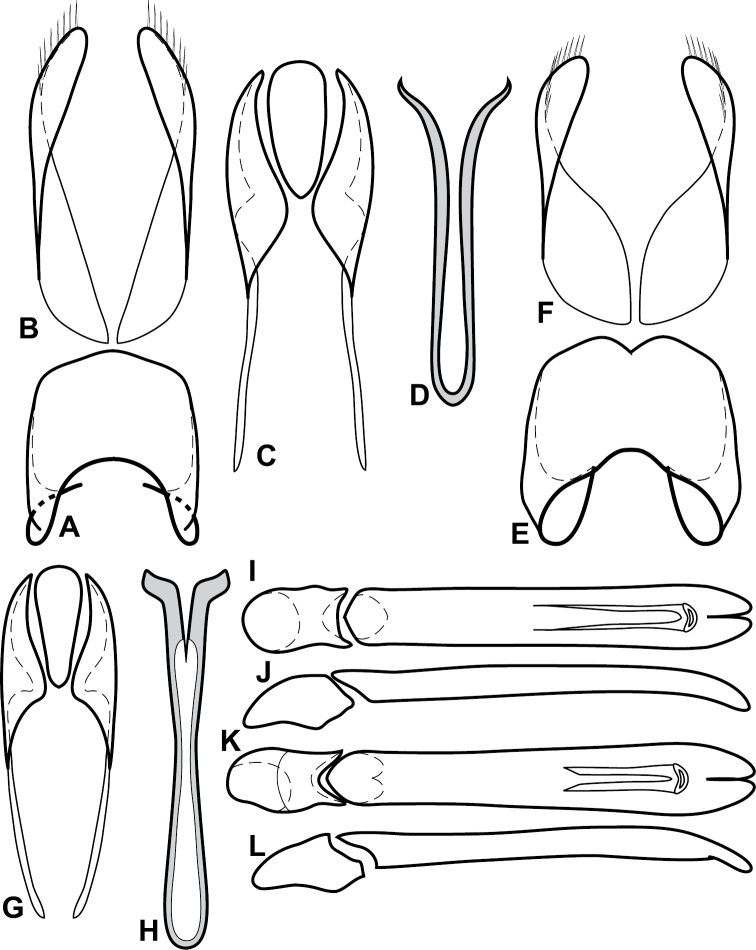
Male genitalia of *Baconia godmani* group. **A** T8 of *Baconia godmani* **B** S8 of *Baconia godmani* **C** T9 & T10 of *Baconia godmani* **D** S9 of *Baconia godmani* **E** T8 of *Baconia venusta* **F** S8 of *Baconia venusta* **G** T9 & T10 of *Baconia venusta* **H **S9 of *Baconia venusta* **I** Aedeagus, dorsal view of *Baconia godmani* **J** Aedeagus, lateral view of *Baconia godmani* **K** Aedeagus, dorsal view of *Baconia venusta* **L** Aedeagus, lateral view of *Baconia venusta*.

The *Baconia godmani* group predominantly comprises species that are metallic-colored and only moderately depressed. Most of the species also have the frons weakly depressed and punctate medially (Fig. 11F), have the prosternal keel at least weakly emarginate basally (Fig. 11D), the labrum bisinuate with a slight median projection (Fig. 11F), and have a conspicuous setal fringe along the apical margin of a very elongate male 8^th^ sternite (Fig. 12B). A number of species here are represented by female specimens only, and their placement should be considered tentative, pending confirmation of male characters: *Baconia splendida*, *Baconia prasina*, *Baconia opulenta*, *Baconia choaspites*, and *Baconia illustris*. *Baconia riehli* is placed here on the basis of external characters, although its male does not have the setal fringe on the 8^th^ sternite. This species may bridge this group and the preceding, representing an early diverging lineage. In general, species in this group are very similar externally, and can only be diagnosed by combinations of relatively subtle external characteristics with male genitalia.

##### Baconia godmani

(Lewis, 1888)

http://species-id.net/wiki/Baconia_godmani

**Map 3.**
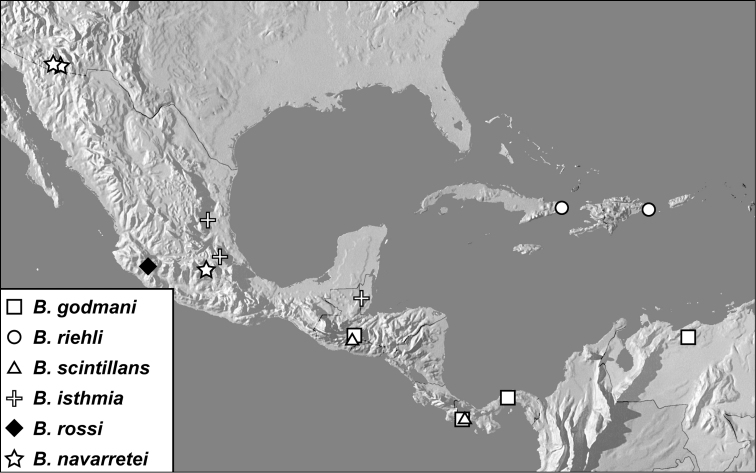
*Baconia godmani* group records. Record for *Baconia godmani* in Venezuela is a country record only.

Figs 11A–B 12A–D, I–J Map 3

Phelister godmani Lewis, 1888: 191; *Baconia godmani*: Mazur 1984: 280

###### Type locality.

PANAMA: Chiriqui: Bugaba [8.49°N, 82.62°W].

###### Type material.

**Holotype**, sex undetermined(BMNH): “Bugaba, 800–1500 ft. Champion” / “B.C.A.,Col.,II,(1). *Phelister*” / “*Phelister godmani* Lewis Type” / “Sp. figured.”.

###### Other material.

**GUATEMALA**: 1: **Zacapa**: Santa Clara, in interior valley of Sierra de las Minas (N. of Cabanas), 5500 ft, 9.viii.1948, under bark, R. Mitchell (FMNH). **PANAMA**:2: **Colón**: P. N. San Lorenzo, STRI Crane Site, 9°17'N, 79°58'W, 28 m, 25.iv–5.v.2004, FIT, M. Gonzales (AKTC, GBFM). **VENEZUELA**: 1:Moritz (ZMHB).

###### Diagnostic description.

Length: 1.9–2.2mm, width: 1.6–1.8mm; body elongate oval, depressed, glabrous; head and pronotum metallic greenish to violet-blue, contrasting with metallic blue elytra, pygidia more subtly metallic blue, venter rufopiceous; frons elevated over antennal bases, depressed at middle, ground punctation fine, with few coarse punctures at middle and near vertex, frontal stria present along inner margin of eye, curving inward at front, interrupted at middle, supraorbital stria more or less complete; antennal scape short, club broadly rounded; epistoma truncate apically; labrum about 4×wider than long, weakly bisinuate along apical margin; both mandibles with acute basal tooth; pronotum with sides weakly arcuate to apex, marginal stria complete along lateral and anterior margins, lateral submarginal stria absent, but generally represented by distinct series of submarginal punctures along side, ground punctation of pronotal disk rather conspicuous, coarse secondary punctures present in lateral thirds; elytra with two complete epipleural striae, outer subhumeral stria absent, inner subhumeral stria largely complete, but usually interrupted in basal half, dorsal striae 1–4 complete, 5^th^ stria complete or abbreviated from base with basal puncture, sutural stria present in about apical half, elytral disk with few coarse punctures in apical fourth; prosternum moderately broad, weakly convex, keel weakly emarginate at base, carinal striae complete, subparallel to divergent anterad, separate or united along basal margin; prosternal lobe about one-half keel length, apical margin broadly rounded, marginal stria obsolete at sides; mesoventrite weakly produced at middle, marginal stria complete; mesometaventral stria arched forward at middle, crenulate, detached at sides, inner lateral metaventral stria extending from end of marginal mesoventral stria obliquely posterolaterad toward middle of metacoxa, outer lateral metaventral stria absent, metaventral disk impunctate at middle; abdominal ventrite 1 with single, complete lateral stria, middle portion of disk lacking coarse punctures; protibia 4–5 dentate, the basal one or two denticles weak, outer margin serrulate between teeth; mesotibia with two weak marginal spines; outer metatibial margin smooth; propygidium without transverse basal stria, discal punctures moderately large, ocellate, separated by about half their diameters basally, slightly smaller and sparser posterad; propygidial gland openings evident about one-third from anterior margin, about one-fourth from each lateral margin; pygidium with ground punctation very fine but rather dense in apical half, secondary punctation evident mainly along basal margin. Male genitalia (Figs 12A–D, I–J): T8 about as long as broad, sides subparallel, basal rim weakly explanate, basal emargination broadly rounded, apical emargination inconspicuous, ventrolateral apodemes separated by about one-third maximum T8 width, extending about one-half distad beneath, obsolete in apical half; S8 much longer than T8, divided, inner margins approximate at base, strongly divergent apically, bearing conspicuous fringe of setae along apical one-third, outer margins subparallel to weakly divergent, apical guides well developed in apical two-thirds, narrowly rounded apically; T9 with basal apodemes thin, about one-half total length, T9 apices narrowly subacute, glabrous, ventrolateral apodemes weakly projecting beneath; S9 stem parallel-sided, weakly widened in basal half, head wide, thin, apicolateral points poorly developed, strongly desclerotized along midline, weakly divided apicomedially; tegmen with sides subparallel throughout, very weakly widened near apex, dorsobasal edge projecting, tegmen in lateral aspect more or less straight, weakly curved ventrad at apex; median lobe about one-third tegmen length; basal piece about one-fifth tegmen length.

###### Remarks.

This species is best distinguished by its lack of basal propygidial stria, presence of sublateral pronotal stria, and presence of the apical half of the sutural elytral stria.

##### Baconia venusta

(J.E. LeConte, 1845)

http://species-id.net/wiki/Baconia_venusta

**Map 4.**
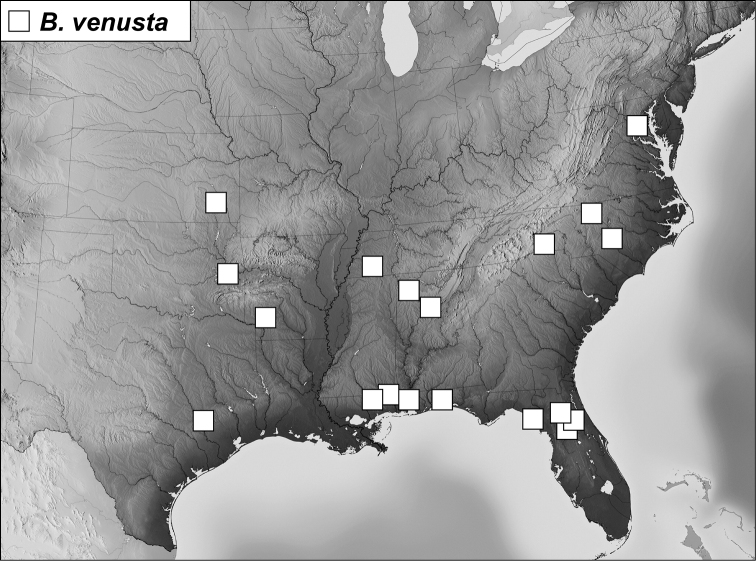
*Baconia venusta* records.

Figs 11C–F 12E–H, K–L Map 4

Platysoma venustum Dejean, 1837 (nom. nud.)
Platysoma venustum J.E. LeConte, 1845: 86; *Hister venustus*: J.L. LeConte 1851: 163; *Phelister venustus*: Marseul 1853: 468; *Phelister venustulus*Marseul 1862: 706 (emend.); *Baconia venusta*: Mazur 1984: 281.
Phelister venustus chalybaeus Casey, 1916: 234; *Baconia venusta chalybaea*: Mazur 1984: 281 (as valid subsp.); Wenzel in Mazur 1997: 26 (synonymized).

###### Type locality.

UNITED STATES: ‘Southern states’ [exact locality uncertain].

###### Type material.

**Lectotype**, here designated (MCZC): [orange locality disk indicating collection in ‘southern states’ (Carolina and Georgia as per original description)] / “7126” / “Type 6896” / “*H. venustus* Lec.” / “LECTOTYPE *Platysoma venustum* J.E.LeConte, M.S.Caterino & A.K.Tishechkin des. 2010”. This species was described from an unspecified number of specimens, and the lectotype designation fixes primary type status on the only known specimen.

###### Other material.

**USA**: **Alabama**: 2: Baldwin Co., Daphne, 8.viii.1958, under bark gum tree, B.K. Dozier (FSCA); 1: Colbert Co., 3mi. W Tuscumbia, 21.vi.1959, under bark, H. Steeves (FMNH); 1: Jefferson Co., Birmingham, Rocky Ridge, 14.vi.1983, at light, W. Suter (FMNH); 1: Mobile Co., Mobile, 16.xi.1924, H. Loding (FMNH), 2: no date (SEMC, FMNH); **Arkansas**: 2: southwest Arkansas (AMNH); 2: Arkansas, state record only (FMNH, AMNH); **Florida**: 1: Alachua Co., Newnan’s Lake, 14.vi.1965, C.W.O’Brien (MHNG); 1: Levy Do., 4 mi SW Archer, 4.vi.1994, at light, R. Aalbu (CDFA); 1: Santa Rosa Co., Milton, Lindgren trap baited with *Persea borbonia*, 21.vi.2007, R. Robinson(FSCA); 1: Marion Co., Ocala, 17.viii.1977, M.C. Thomas (FSCA); 1: Dixie Co. 6 mi N Old Town, 23.vii.1978, M.C. Thomas (FSCA); 1: Putnam Co., 2.iii.1960, under bark dead *Quercus laevis*, H.V. Weems (FSCA), 1: same data but 18.vi.1960 (FSCA); 1: A. Slosson (AMNH); **Kansas**: 1: Woodson Co., Cross Timbers St. Pk., E Spillway Access , 37.73918°N, 95.91898°W, 10.vi.2010, under bark, Z. Falin, DNA Extract MSC-2231, EXO-00944; 1: **Louisiana**: 1: East Baton Rouge Par., Baton Rouge, 12.ii.1982, under bark, S.M. Strother (LSAM); 1: Natchitoches Par., 1 mi NNE Lotus, 31°30'N, 93°7.5'W, 12.iv-3.vii.1996, FIT, A. Cline, S. Dash & M. Seymour (LSAM); 1: W Feliciana Par., Feliciana Pres., nr. Freeland, 30°47'N, 91°15'W, FIT, 29.v–12.vi.2005, A. Tishechkin & S. Gil (LSAM); **Maryland**: 4: Pr. Georges Co., 18.vii.1948, under bark (thin) tree, fire-killed 3 1/2 mos prior, G. Vogt (USNM), 5: 19.vi.1949, tree, fire-killed 14 1/2 mos. ago, red or black oak, G. Vogt (USNM), 1: 3.vii.1948, under thin bark red or black oak, fire-killed 3 mos. prior, G. Vogt (USNM); **Mississippi**: 4: George Co., Lucedale, 16.i.1931, H. Dietrich (FMNH), 1: 18.iv.1930, H. Dietrich (FMNH); **North Carolina**: 1: Guilford Co., Greensboro, 21.vi.1956, P. Ashlock (SEMC); 1: Southern Pines, 24.xi.1911, A.H. Manee (FMNH), 3: 25.iii.1911, 1: 25.ii.1911, 1: 16.i.1911, 1: 8.i.1915 (NCSU); 1: Cleveland Co., 20.v.1972, J. Ashe; **Oklahoma**: 2: Latimer Co., 5 mi. W Red Oak, x.1980, K. Stephan, 1: vi.1981, 5: vi.1982, 1: xi.1982, 3: v.1983, FIT, 1: vi.1983, 1: vi.1984, FIT, 1: v.1984, FIT, 1: v.1984, tree hole oak + rodent, 1: iv.1985, 2: vi.1985, 1: vii.1985, 1: v.1986, 3: v.1987, 2: iv.1991, 2: v.1991, 1: v.1993, 1: vi.1993, 1: iv.1994, 1: vii.1995 (all K. Stephan; FMNH, TAMU, FSCA); **Tennessee**: Hardeman Co., 5 mi S Bolivar on Union RD(8232), 200m, 28.vii.1972 (MHNG); 1: **Texas**: Brazos Co., 9.xi.1935, J. Robinson (FMNH).

###### Diagnostic description.

Length: 2.0–2.3mm, width: 1.7–2.0mm; body elongate oval, depressed, glabrous; dorsum metallic blue to greenish-blue, venter rufopiceous; frons elevated over antennal bases, depressed at middle, ground punctation fine, with few coarse punctures at middle and near vertex, frontal stria present along inner margin of eye, curving inward at front, interrupted over antennal bases, at middle, or both, supraorbital stria vaguely represented by series of punctures; antennal scape short, club broadly rounded; epistoma truncate apically; labrum about 4×wider than long, weakly bisinuate along apical margin; both mandibles with acute basal tooth; pronotum with sides increasingly arcuate to apex, marginal stria complete along lateral and anterior margins, lateral submarginal stria absent, ground punctation of pronotal disk rather conspicuous, interspersed with coarser secondary punctures at sides, nearly to midline anteriorly; elytra with two complete epipleural striae, outer subhumeral stria absent, inner subhumeral stria present as basal and frequently median fragments, dorsal striae 1–4 complete, 5^th^ stria present in apical two-thirds and frequently with basal puncture, sutural stria present in apical half or slightly more, elytral disk with few coarse punctures in apical fourth; prosternum moderately broad, weakly convex, keel emarginate at base, carinal striae complete, subparallel to divergent anterad, separate or united along basal margin; prosternal lobe about two-thirds keel length, apical margin rounded, marginal stria obsolete at sides; mesoventrite produced at middle, marginal stria narrowly interrupted at middle; mesometaventral stria arched forward, crenulate, narrowly detached from lateral metaventral stria, which curves posterolaterad toward middle of metacoxa, outer lateral metaventral stria absent, metaventral disk impunctate at middle; abdominal ventrite 1 with single, complete lateral stria, middle portion of disk lacking coarse punctures; protibia 4–5 dentate, the basal one or two denticles weak, outer margin serrulate between teeth; mesotibia with two weak marginal spines; outer metatibial margin smooth; propygidium with complete transverse basal stria, discal punctures ocellate, separated more or less uniformly by about their diameters; propygidial gland openings evident behind ends of transverse basal stria, about one-fourth from each lateral margin; pygidium with ground punctation rather dense in apical half, secondary punctation denser toward base. Male genitalia (Figs 12E–H, K–L): T8 about as long as broad, sides subparallel, narrowed to base, basal emargination broadly, unevenly rounded, apical emargination very shallow, ventrolateral apodemes separated by about one-half maximum T8 width, extending about one-third distad beneath, strongly narrowed in apical half; S8 longer than T8, divided, inner margins approximate along basal one-fourth, strongly divergent apically, bearing conspicuous fringe of setae along apical one-third, outer margins subparallel to weakly divergent, apical guides well developed in apical half, broadly rounded apically; T9 with basal apodemes thin, about one-half total length, T9 apices narrowly rounded, glabrous, ventrolateral apodemes moderately strongly projecting beneath; S9 widened in basal half, head similar in width, subangulate to apicolateral points, desclerotized along midline, with narrow apicomedial division; tegmen with sides subparallel in basal half, weakly widened to apex, dorsobasal edge projecting, tegmen in lateral aspect more or less straight, slightly curved ventrad at apex; median lobe about one-fourth tegmen length; basal piece about one-fifth tegmen length.

###### Remarks.

This is one of only three species of *Baconia* occurring in the eastern US, and only shares metallic coloration with one, *Baconia aeneomicans*. It is easily distinguished from this species (see Fig. 38A) by its larger size, more broadly rounded body form, and relatively uniform coloration (Fig. 11C).One additional species occurs in the American southwest, *Baconia navarretei*, which is more similar. It is not likely that the ranges of these species will overlap, but *Baconia venusta* is nonetheless easily distinguished by the presence of a basal propygidial stria, and the presence of the median portion of the mesometaventral stria (Fig. 11D), both striae lacking in *Baconia navarretei*.

Mazur (2011) reported this species from Mexico. However, we have studied those specimens and assign them to *Baconia eximia*, below.

##### Baconia riehli

(Marseul, 1862) comb. n.

http://species-id.net/wiki/Baconia_riehli

Figs 11G–H 13 Map 3

Phelister riehli Marseul, 1862: 697.

###### Type locality.

CUBA [exact locality uncertain].

###### Type material.

**Lectotype**, sex undetermined, here designated (MNHN): “*Phelister riehli* M. Cuba [rest illegible]” / “[green heart-shaped label]” / “Museum Paris Coll. De Marseul 2842-90” / “Type” / “LECTOTYPE *Phelister riehli* Marseul, 1862, M.S.Caterino & A.K.Tishechkin des. 2010”. This species was described from an unspecified number of specimens, and the lectotype designation fixes primary type status on the only known specimen.

###### Other material.

**CUBA**: 1:Upper Ovando R. eastern Oriente, 1000-2000 ft, 17-20.vii.1936, P. Darlington (FMNH); 1: country record only (ZMHB). **DOMINICAN REPUBLIC**: 1: **La Altagracia**: P. N. del Este, Boca de Yuma, 18°21.508'N, 68°36.956'W, 20 m, 19.vii.2004, S. Lingafelter (AKTC).

###### Diagnostic description.

**Figure 13.**
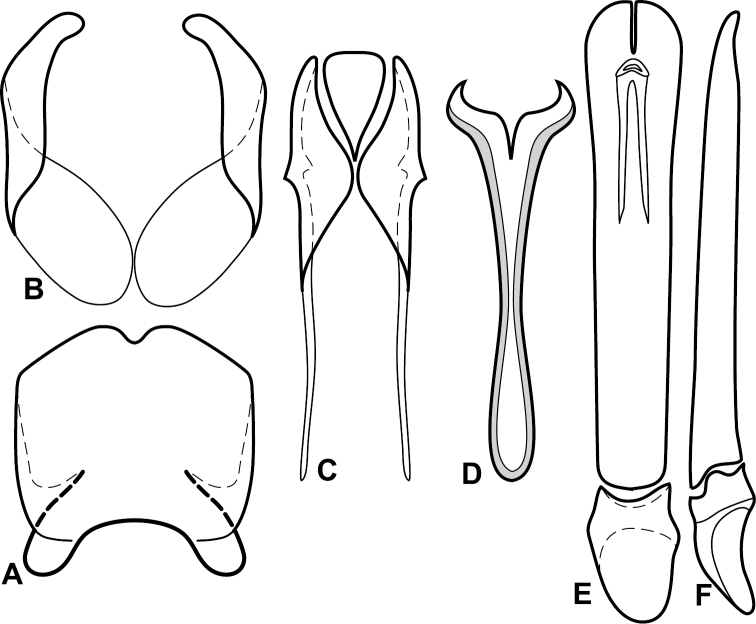
Male genitalia of *Baconia riehli*. **A** T8 **B** S8 **C** T9 & T10 **D** S9 **E** Aedeagus, dorsal view **F **Aedeagus, lateral view.

Length: 2.2–2.3mm, width: 1.7–2.0mm; body elongate oval, almost parallel-sided, moderately depressed, glabrous; most of body, including venter, metallic blue to greenish-blue, elytra moderately to strongly violet; frons elevated over antennal bases, depressed at middle, ground punctation rather conspicuous, with small secondary punctures on epistoma, slightly larger punctures in frontal depression, frontal stria present along inner margin of eye, fragmented to absent across front, supraorbital stria vaguely represented by series of punctures; antennal scape short, club slightly asymmetrically oblong; epistoma slightly convex along apical margin, truncate; labrum about 3×wider than long, weakly emarginate to bisinuate along apical margin; both mandibles with acute basal tooth; pronotal sides weakly convergent in basal half, arcuate to apex, marginal stria complete along lateral and anterior margins, lateral submarginal stria absent, ground punctation of pronotal disk rather conspicuous, interspersed with small secondary punctures nearly throughout, only lacking in posteromedial region; elytra with two complete epipleural striae, outer subhumeral stria absent, inner subhumeral stria present as basal and frequently median fragments, dorsal striae 1–3 complete, 4^th^ stria variably abbreviated from base, 5^th^ and sutural striae absent, elytral disk with few coarse punctures in apical fourth; prosternum narrowed between procoxae, weakly convex, keel truncate to very weakly emarginate at base, carinal striae complete, divergent anterad and posterad, separate throughout; prosternal lobe about two-thirds keel length, apical margin subtruncate, marginal stria obsolete; mesoventrite weakly emarginate, slightly sinuate at middle, with a complete stria slightly removed from margin which is probably the mesometaventral stria, strongly displaced anterad; lateral metaventral stria originating freely near mesocoxa, curving posterolaterad toward middle of metacoxa, slightly abbreviated apically, outer lateral metaventral stria absent, metaventral disk impunctate at middle; abdominal ventrite 1 with single, complete lateral stria, middle portion of disk with few small punctures along apical margin; protibia 4–5 dentate, the basal one or two denticles weak, outer margin serrulate between teeth; meso- and metatibiae rather elongate, narrow, mesotibia with two weak marginal spines; outer metatibial margin smooth; propygidium with complete transverse basal stria, discal punctures rather small, ocellate, separated more or less uniformly by about their diameters; propygidial gland openings conspicuous behind ends of transverse basal stria, about one-fourth from each lateral margin; pygidium with sparse ground punctation interspersed with small secondary punctures throughout, these slightly larger and denser toward base. Male genitalia (Fig. 13): T8 slightly longer than broad, sides subparallel to weakly rounded, basal emargination broad, shallow, apical emargination very shallow, ventrolateral apodemes separated by about one-half maximum T8 width, extending about one-third distad beneath, strongly narrowed in apical half; S8 about as long as T8, divided, inner margins approximate along basal one-fourth, strongly divergent apically, outer margins subparallel, convergent to apex, apical guides well developed in apical apical two-thirds, elongate, narrowly rounded apically, lacking apical setae; T9 with basal apodemes thin, almost two-thirds total length, T9 apices narrowly subacute, glabrous, weakly opposing, ventrolateral apodemes weakly projecting beneath; S9 stem thin near basal one-third, weakly bulbous at base, head wide, curved to horn-like apicolateral points, desclerotized along midline, with narrow apicomedial division; tegmen with sides subparallel in basal half, weakly widened to near apex, slightly bulbous apically, dorsobasal edge weakly arcuate, tegmen in lateral aspect more or less straight; median lobe about one-third tegmen length; basal piece about one-fourth tegmen length.

###### Remarks.

At present, *Baconia riehli* is one of only two species in the genus known to occur in the West Indies. The other is *Baconia pulchella*, in the *Baconia aeneomicans* group. It is interesting to note that both of these exhibit more distinctly violet coloration than do nearly any mainland species, although they are not closely related. The two are easily distinguished by the larger, more broadly body form of *Baconia riehli* (Fig. 11G vs. 38C). From mainland species in the *Baconia godmani* group, this species is unique in lacking a marginal stria on the prosternal lobe.

##### Baconia scintillans sp. n.

http://zoobank.org/486DCFAB-F1F1-4C25-A5A5-58DA6FF2D383

http://species-id.net/wiki/Baconia_scintillans

Figs 14A–B Map 3

###### Type locality.

PANAMA: Chiriqui: Hornito [8.68°N, 82.23°W].

###### Type material.

**Holotype male**: “PANAMA: Chiriquí Prov., Hornito, Finca La Suiza, 1220m 1.VI.2000 H. & A. Howden, FIT” / “Caterino/Tishechkin Exosternini Voucher EXO-00445” (CMNC). **Paratype** (1): same data as type, except: 3.vi.2000, FIT, H. & A. Howden (CMNC).

###### Other material.

**GUATEMALA: Zacapa**, Santa Clara, interior valley of Sierra de las Minas, under bark, 9.viii.1948, 5500 ft., R.D.Mitchell leg. (FMNH).

###### Diagnostic description.

Length: 2.2–2.3mm, width: 1.9–2.0mm; body broadly elongate oval, depressed, glabrous; color metallic greenish-blue over most of dorsum, venter rufobrunneus; frons elevated over antennal bases, depressed at middle, ground punctation fine, with few coarse punctures at middle and near vertex, frontal stria present along inner margin of eye, curving inward at front, interrupted over antennal bases, at middle, or both, supraorbital stria weakly impressed but complete; antennal scape short, club nearly circular; epistoma truncate apically; labrum about 3×wider than long, weakly emarginate apically; both mandibles with acute basal tooth; pronotum with sides increasingly arcuate to apex, marginal stria complete along lateral and anterior margins, lateral submarginal stria absent, ground punctation of pronotal disk very fine, interspersed with coarser secondary punctures in lateral fourths; elytra with two complete epipleural striae, outer subhumeral stria absent, inner subhumeral stria present as basal and median fragments, dorsal striae 1–3 complete, 4^th^ and 5^th^ striae weakly impressed in apical fourth, sutural stria present in apical third, elytral disk with few coarse punctures along apical margin; prosternum moderately broad, weakly convex, keel weakly emarginate at base, carinal striae complete, slightly sinuate, separate throughout; prosternal lobe about two-thirds keel length, apical margin rounded, marginal stria present along most of margin; mesoventrite weakly produced at middle, marginal stria interrupted for median one-third; mesometaventral stria arched forward, weakly crenulate, detached from lateral metaventral stria, which extends from inner corner of mesocoxa posterolaterad toward outer third of metacoxa, abbreviated apically, outer lateral metaventral stria absent, metaventral disk impunctate at middle; abdominal ventrite 1 with single, complete lateral stria, middle portion of disk lacking coarse punctures; protibia 4–5 dentate, the basal one or two denticles weak, outer margin serrulate between teeth; mesotibia with two weak marginal spines; outer metatibial margin smooth; propygidium lacking basal stria, with coarse punctures subserially arranged along basal margin, separated by 1–2× their diameters elsewhere; propygidial gland openings evident about one-third behind anterior margin, about one-fourth width from each lateral margin; pygidium with ground punctation rather dense in apical half, secondary punctation denser toward base. Male genitalia extremely similar that of *Baconia godmani* (see Figs 12A–D, I–J), differing as follows: T8 slightly more elongate, S8 with apical guides slightly more broadly expanded toward apex, tegmen with sides slightly more undulate, apex slightly more broadly rounded.

**Figure 14.**
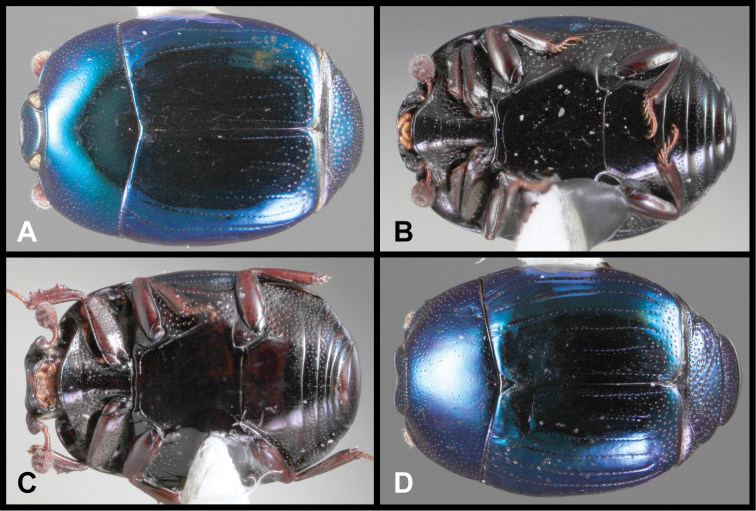
*Baconia godmani* group. **A** Dorsal habitus of *Baconia scintillans* **B** Ventral habitus of *Baconia scintillans* **C** Ventral habitus of *Baconia rossi* **D** Dorsal habitus of *Baconia navarretei*.

###### Remarks.

This species lacks submarginal pronotal and basal propygidial striae, and has an isolated median fragment of the mesometaventral stria (Fig. 14B). It is similar in all these respects only to *Baconia choaspites*, which lacks a sutural elytral stria and has a deeply emarginate labrum. In *Baconia scintillans*, the labrum is weakly bisinuate and the sutural stria is present in the apical half. The two types, collected at the same locality, differ substantially in color, with the paratype distinctly bluer, not greenish-blue as the holotype. Some of the coloration of the latter, however, may result from a slightly oily surface.

###### Etymology.

This species’ name refers to its shiny, metallic appearance.

##### Baconia isthmia sp. n.

http://zoobank.org/EFFF9258-3D7B-4943-8089-7A8A63064176

http://species-id.net/wiki/Baconia_isthmia

**Figure 15.**
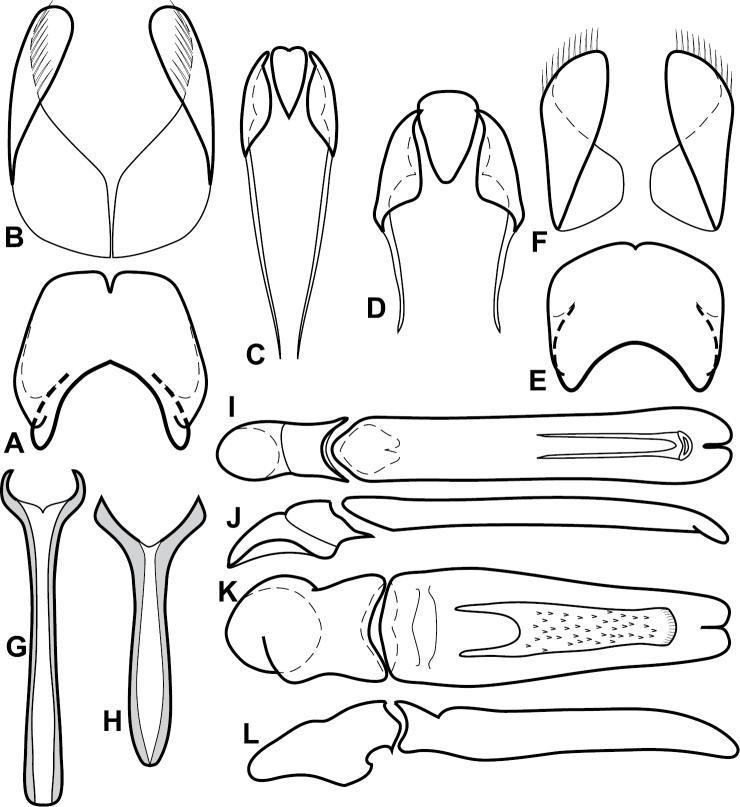
Male genitalia of *Baconia godmani* group. **A** T8 of *Baconia isthmia* **B** S8 of *Baconia isthmia* **C** T9 & T10 of *Baconia isthmia* **D** T9 & T10 of *Baconia maculata* **E** T8 of *Baconia maculata* **F** S8 of *Baconia maculata* **G** S9 of *Baconia isthmia* **H **S9 of *Baconia maculata* **I** Aedeagus, dorsal view of *Baconia isthmia* **J** Aedeagus, lateral view of *Baconia isthmia* **K** Aedeagus, dorsal view of *Baconia maculata* **L** Aedeagus, lateral view of *Baconia maculata*.

Figs 15A–C, G, I–J Map 3

###### Type locality.

**MEXICO**: **San Luis Potosí**: El Salto Falls [22.58°N, 99.37°W].

###### Type material.

**Holotype male**: “MEXICO: San Luis Potosí: El Salto Falls, 12 km NW El Naranjo, 26 July 1990, 400 m, J.S.Ashe, K.-J.Ahn, R.Leschen #246 ex.fungusy log” / “SEMC0903644” (SEMC). **Paratypes** (3): 1: **BELIZE**: **Cayo**: Las Cuevas, 8.v.1994 (BMNH). 2: **MEXICO: Puebla**: 4.7 mi. SW La Cumbre, 5200 ft., 23.vii.1987, Kovarik & Schaffner (CHPWK).

###### Diagnostic description.

Length: 2.1–2.2mm, width: 1.8–1.9mm; body broadly elongate oval, depressed, glabrous; color metallic blue to greenish-blue over most of dorsum, pronotum may be more distinctly greenish (not in type), venter rufobrunneus; frons elevated over antennal bases, depressed at middle, ground punctation fine, with few coarse punctures at middle and near vertex, frontal stria present along inner margin of eye, curving inward at front, interrupted over antennal bases and at middle; antennal scape short, club nearly circular; epistoma truncate apically; labrum about 4×wider than long, weakly bisinuate apically; both mandibles with acute basal tooth; pronotum with sides increasingly arcuate to apex, marginal stria complete along lateral and anterior margins, lateral submarginal stria absent, disk depressed along anterior fifth of lateral margin, ground punctation of pronotal disk very fine, coarser secondary punctures limited to narrow lateral region; elytra with two complete epipleural striae, outer subhumeral stria absent, inner subhumeral stria present as basal and median fragments, dorsal striae 1-3 complete, 4^th^ stria complete or interrupted in basal half, 5^th^ stria weakly impressed in apical half to two-thirds, abbreviated from apex, sutural stria shorter than 5^th^ anteriorly, but extending further to apex, elytral disk with few coarse punctures in apical sixth; prosternum moderately broad, weakly convex, keel subacutely emarginate at base, carinal striae complete, depressed at bases, slightly sinuate, weakly divergent anterad; prosternal lobe about two-thirds keel length, apical margin rounded, marginal stria obsolete at sides; mesoventrite subacutely produced at middle, marginal stria interrupted for width of prosternal keel; mesometaventral stria strongly arched forward, weakly crenulate, detached from lateral metaventral stria, which extends from inner corner of mesocoxa posterolaterad toward outer corner of metacoxa, abbreviated apically, outer lateral metaventral stria absent, metaventral disk impunctate at middle; abdominal ventrite 1 with single, complete lateral stria, middle portion of disk lacking coarse punctures; protibia 4–5 dentate, the basal denticles weak, outer margin serrulate between teeth; mesotibia with two weak marginal spines; outer metatibial margin smooth; transverse basal propygidial stria varied, complete or represented only by subserially arranged punctures, punctures otherwise rather small and sparse, separated by 1–2× their diameters; propygidial gland openings evident about one-third behind anterior margin, about one-fourth width from each lateral margin; pygidium with ground punctation rather dense in apical half, secondary punctation evident only along basal margin. Male genitalia (Figs 15A–C, G, I–J): T8 slightly shorter than broad, sides weakly widened in basal one-third, convergent to apex, basal emargination broad, deep, weakly acute at middle, basal rim slightly explanate, apical emargination narrow, shallow, with ventrolateral apodemes separated by about one-half maximum T8 width, extending about one-half distad beneath, obsolete in apical half; S8 divided, inner margins approximate at base, weakly divergent in basal half, strongly divergent to apex, bearing conspicuous fringe of setae in apical one-third, outer margins subparallel, apical guides well developed in apical half, narrowly lobate apically; T9 with basal apodemes thin, about two-thirds total length, T9 apices narrow, bluntly subacute, weakly opposed, glabrous, ventrolateral apodemes weakly projecting beneath; S9 weakly widened at base, head broad, with apicolateral points curved, horn-like, desclerotized along midline, with narrow apicomedial division; tegmen with sides subparallel to near apex, apical one-fourth weakly bulbous, broadly rounded at apex, dorsobasal edge projecting, tegmen more or less straight in lateral aspect; median lobe about one-half tegmen length; basal piece about one-fourth tegmen length.

###### Remarks.

*Baconia isthmia* is very closely related to *Baconia scintillans*, above, both lacking a submarginal lateral pronotal stria, and exhibiting an isolated median fragment of the mesometaventral stria. *Baconia isthmia* is slightly smaller, a little more distinctly flattened, has a distinct transverse propygidial stria, has the 4^th^ elytral stria reaching the base (may be interrupted), and lacks a median fragment of the inner subhumeral stria. There are slight genitalic differences as well, with the tegmen of *Baconia isthmia* slightly shorter and more spatulate apically, and the 8^th^ sternite of the male less elongate, than those of *Baconia scintillans* or *Baconia godmani*.

###### Etymology.

This species is named for Isthmian region (de Tehuantepec) that spans the region inhabited by this species.

##### Baconia rossi sp. n.

http://zoobank.org/E4EC553A-1827-4520-BD65-4D138625106A

http://species-id.net/wiki/Baconia_rossi

Fig. 14C Map 3

###### Type locality.

MEXICO: Jalisco: 6 km S Tecalitlan [19.4°N, 103.3°W].

###### Type material.

**Holotype male**: “MEXICO: Jalisco: 6 km S Tecalitlan, 1219 m, 14-VIII-1978, Edward S. Ross, Cal.Acad.Sci.Coll.” / “Caterino/Tishechkin Exosternini Voucher EXO-00497” (CASC). **Paratypes** (2): 1: same data as type (CASC), 1: same locality, 9.xi.1980, E. Ross (CASC).

###### Diagnostic description.

Length: 2.1–2.3mm, width: 1.7–1.9mm; body elongate oval, subparallel-sided, moderately depressed, glabrous; dorsum uniformly metallic blue, venter rufopiceous; frons elevated over antennal bases, depressed at middle, ground punctation fine, with coarse punctures on epistoma, at middle and near vertex, frontal stria present along inner margin of eye, curving inward at front, interrupted at middle, rarely also interrupted over antennal bases, supraorbital stria absent; antennal scape short, club rounded; epistoma truncate apically; labrum about 4×wider than long, weakly bisinuate along apical margin; both mandibles with acute basal tooth; pronotal sides weakly convergent in basal half, arcuate to apex, marginal stria complete along lateral and anterior margins, lateral submarginal stria absent, ground punctation of pronotal disk fine, coarse secondary punctures extending across anterior margin and conspicuous in lateral thirds; elytra with two complete epipleural striae, outer subhumeral stria absent, inner subhumeral stria largely complete, may be weakly interrupted in middle, dorsal striae 1-3 complete, 4^th^ stria weakened or interrupted near base, present in apical half or more, 5^th^ stria present in apical third, sutural stria slightly longer, elytral disk with few coarse punctures in apical fifth; prosternum moderately broad, weakly convex, keel truncate to very weakly emarginate at base, carinal striae complete, united or nearly united along basal margin, subparallel anterad; prosternal lobe about one-half keel length, apical margin broadly rounded, marginal stria weak, obsolete at sides; mesoventrite weakly sinuate at middle, marginal stria interrupted; mesometaventral stria absent represented by short, weak median fragment, inner lateral metaventral stria extending from near mesocoxa obliquely posterolaterad toward outer third of metacoxa, abbreviated apically, outer lateral metaventral stria absent, metaventral disk impunctate at middle; abdominal ventrite 1 with single, complete lateral stria, middle portion of disk lacking coarse punctures; protibia 4-dentate, the basal denticle weak, outer margin serrulate between teeth; mesotibia with two weak marginal spines; outer metatibial margin smooth; propygidium without transverse basal stria, discal punctures moderately large, ocellate, separated by about half their diameters, smaller posterad; propygidial gland openings evident about one-third from anterior margin, about one-fourth from each lateral margin; pygidium with ground punctation very fine but rather dense in apical half, secondary punctation evident mainly in basal third. Male genitalia essentially indistinguishable from that of *Baconia godmani* (though aedeagus was missing from type and not available for study).

###### Remarks.

This species is closely related to *Baconia godmani*, *Baconia isthmia*, and *Baconia scintillans*, as revealed by male genitalia, but it lacks the lateral submarginal pronotal stria of *Baconia godmani*, and differs from all three in its complete inner subhumeral stria. Additionally, in *Baconia rossi* the prosternal striae tend to be united basally or at least curved medially at the base (Fig. 14C), and the isolated fragment of the mesometaventral stria is very weak.

###### Etymology.

This species is named to honor Dr. Edward Ross, collector of the types and histerid aficionado going back many decades.

##### Baconia navarretei sp. n.

http://zoobank.org/B036FFE7-8BF6-4101-BC5F-ADA38A0B2C92

http://species-id.net/wiki/Baconia_navarretei

Fig. 14D Map 3

###### Type locality.

UNITED STATES: Arizona: Huachuca Mts. [31.42°N, 110.27°W].

###### Type material.

**Holotype male**: “**ARIZONA**: Cochise Co., Huachuca Mts. Miller Canyon Rec. Ar., 31°25'N, 110°16.5'W, Oak, u/bark 17 July 2001, A.Tishechkin” / “Caterino/Tishechkin Exosternini Voucher EXO-01120” (FMNH). **Paratypes** (3): **USA**: **Arizona:** 1: Cochise Co., Huachuca Mts. Sunnyside Canyon, 5500 ft, 18.vii.1972, R. Curtis (FMNH); 1: Santa Cruz Co., Santa Rita Mts. Madera Cyn, 25.vii.1966, K. Stephan (USNM); 1: Pima Co., Madera Cyn 31.72694°N, 110.88039°W, 20.vii.2012, E.G.Riley (TAMU)

###### Other material.

**MEXICO**: 2: **Morelos**: Tlayacapan, San Jose de los Laureles, BMM, 1768 m, 14.viii.1993, rotting log, G. Quiroz y J. Navarrete (UDGC, MSCC); 1: Morelos: Tlayacapan, San Jose de los Laureles, BMM, 1768 m, 14.viii.1993, rotting log, G. Quiroz y J. Navarrete (AKTC).

###### Diagnostic description.

Length: 2.4–2.6mm, width: 2.0–2.2mm; body elongate oval, depressed, glabrous; dorsum metallic blue, venter rufopiceous; frons elevated over antennal bases, depressed at middle, ground punctation fine, with few coarse punctures at middle and near vertex, frontal stria present along inner margin of eye, curving inward at front, interrupted over antennal bases, at middle, or both, supraorbital stria vaguely represented by series of punctures; antennal scape short, club broadly rounded; epistoma truncate apically; labrum about 4×wider than long, weakly bisinuate along apical margin; both mandibles with acute basal tooth; pronotum with sides weakly arcuate to apex, marginal stria complete along lateral and anterior margins, lateral submarginal stria absent, ground punctation of pronotal disk rather conspicuous, coarse secondary punctures extending across anterior half, becoming more widespread and dense along sides; elytra with two complete epipleural striae, outer subhumeral stria absent, inner subhumeral stria largely complete, but usually interrupted in basal half, dorsal striae 1–3 complete, 4^th^ stria abbreviated from base, present in apical half or more, 5^th^ stria present in apical third, sutural stria present in about apical half, elytral disk with few coarse punctures in apical fourth; prosternum moderately broad, weakly convex, keel weakly emarginate at base, carinal striae complete, subparallel to divergent anterad, separate or united along basal margin; prosternal lobe about one-half keel length, apical margin broadly rounded, marginal stria obsolete at sides; mesoventrite weakly produced at middle, marginal stria complete; mesometaventral stria absent, inner lateral metaventral stria extending from end of marginal mesoventral stria obliquely posterolaterad toward outer third of metacoxa, slightly abbreviated apically, outer lateral metaventral stria absent, metaventral disk impunctate at middle; abdominal ventrite 1 with single, complete lateral stria, middle portion of disk lacking coarse punctures; protibia 4–5 dentate, the basal one or two denticles weak, outer margin serrulate between teeth; mesotibia with two weak marginal spines; outer metatibial margin smooth; propygidium without transverse basal stria, discal punctures moderately large, ocellate, separated by about half their diameters basally and laterally, sparser medioapically; propygidial gland openings evident about one-third from anterior margin, about one-fourth from each lateral margin; pygidium with ground punctation very fine but rather dense in apical half, secondary punctation evident mainly along basal margin. Male genitalia indistinguishable in shape from those of *Baconia venusta* (see Figs 12E–H, K–L), although somewhat larger in absolute size.

###### Remarks.

*Baconia navarretei* is a relatively large species, completely lacking mesometaventral and basal propygidial striae. Although rather superficial, the most consistently distinguishing character of this species is the fact that the 5^th^ elytral stria is shorter than either the 4^th^ or the sutural, all of them being largely restricted to the posterior half of the elytron. In addition the lateral pronotal punctures tend to be very dense near the margin (Fig. 14D), moreso than in most similar species. Due to variation in pygidial sculpturing, and the large gap between localities for available material we restrict the type series to those specimens from Arizona, USA.

###### Etymology.

We name this species for Dr. Jose Luis Navarrete Heredia, of the University of Guadalajara, collector of several specimens of this species, in recognition of his valuable contributions to the knowledge of beetles of western Mexico, and recognizing his considerable contributions to many of our studies.

##### Baconia maculata sp. n.

http://zoobank.org/845B8BBC-5DFA-4C64-9A51-AABCCD05F691

http://species-id.net/wiki/Baconia_maculata

**Map 5.**
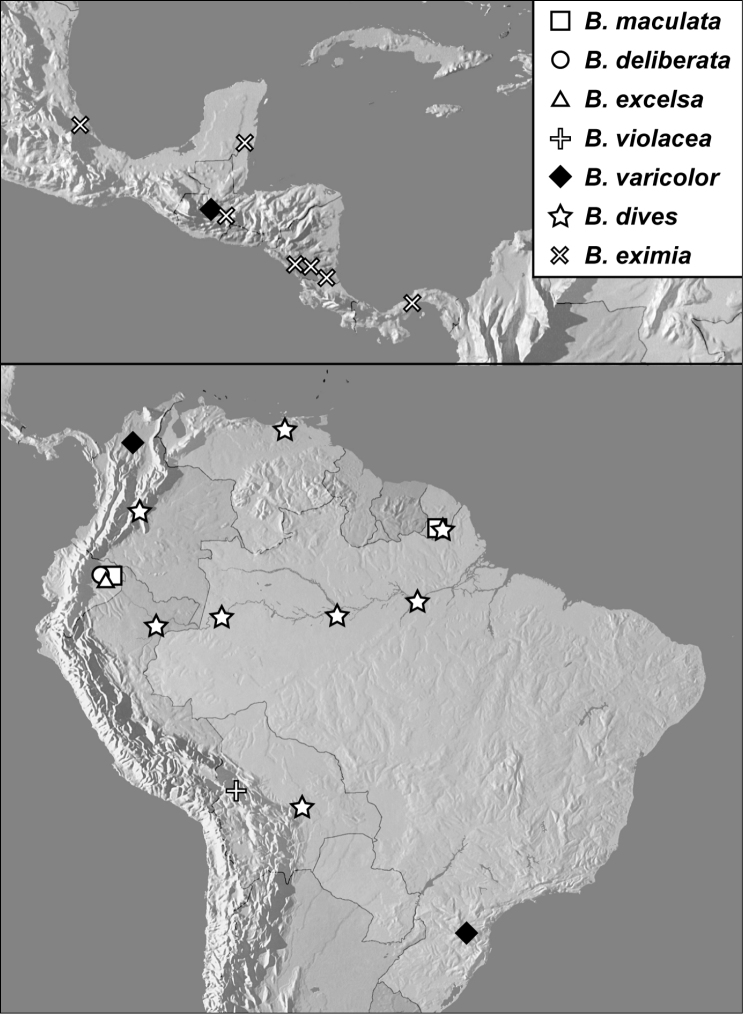
*Baconia godmani* group records. Record for *Baconia varicolor* in Colombia is a country record only.

Figs 15D–F, H, K–L 16A Map 5

###### Type locality.

ECUADOR: Orellana:Res. Ethnica Waorani [0.67°N, 76.43°W].

###### Type material.

**Holotype male**: “**ECUADOR: Depto. Orellana**:Res. Ethnica Waorani, 1km S Onkone Gare Camp, Trans. Ent., 0°39'10"S, 76°26'W, 220m, 25 June 1996, T.L. Erwin et al. collectors” / “Insecticidal fogging of mostly bare green leaves, some with covering of lichenous or bryophytic plants in terra firme forest. Project MAXUS **Lot 1536 Trans. 2 Sta. 6**” / “Caterino/Tishechkin Exosternini Voucher EXO-00448” (USNM). **Paratype** (1): **FRENCH GUIANA**: Bélvédère de Saül, point de vue. 3°1'22"N, 53°12'34"W, FIT, 17.i.2011. SEAG leg. (CHND).

###### Diagnostic description.

Length: 1.7–1.8mm, width: 1.2–1.3mm; body elongate oval, subdepressed, glabrous; head and pronotum metallic greenish-blue, elytra metallic blue with distinct rufescent maculations extending across middle of disc from approximately sutural stria laterad to margin; frons together with epistoma strongly convex, weakly depressed at middle, uniformly coarsely punctate, interocular margins very weakly convergent dorsad, frontal stria present along inner margin of eyes, interrupted above antennal bases and at middle; antennal scape short, apex obliquely truncate, club elongate, subquadrate; epistoma convex along apical margin, weakly emarginate; labrum about 4×wider than long, apex broadly, shallowly emarginate; mandibles short, each with small, acute basal tooth; palpomeres stout, flattened; pronotal sides subparallel in basal half, weakly arcuate to apex, marginal stria complete around lateral and anterior margins, submarginal stria present close to lateral marginal, ending about one-fifth from anterior corner; pronotal disk with fine ground punctation and slightly coarser secondary punctures more or less uniformly dispersed throughout; elytra with two complete epipleural striae, outer subhumeral stria absent, inner subhumeral stria present in basal two-thirds, but interrupted in middle, dorsal striae 1–5 complete to base, progressively more abbreviated apically mediad, with 5^th^ stria obsolete in apical fourth, sutural stria present in apical two-thirds, obsolete at base; elytral disk with secondary punctures in apical fourth; prosternal keel narrow, weakly convex, base truncate, carinal striae complete, bent mediad at apices, but ending freely; prosternal lobe about two-thirds keel length, broadly rounded, marginal stria slightly fragmented at sides; anterior edge of mesoventrite weakly sinuate, marginal stria complete; mesometaventral stria arched forward, crenulate, detached at sides, inner lateral metaventral stria originating close to mesocoxa, extending posterolaterad toward middle of metacoxa, outer lateral metaventral stria short, oblique, metaventral and 1^st^ abdominal disks impunctate at middle; abdominal ventrite 1 with complete inner lateral stria and posterior fragments of outer stria; protibia with five marginal denticles, the basal two weak, outer margin serrulate between; mesotibia with 2–3 marginal spines; outer metatibial margin smooth; propygidium without transverse basal stria, with moderately large, ocellate punctures uniformly separated by about their diameters; propygidial gland openings evident about one-third from anterior and one-fourth from lateral margins; pygidium with fine, sparse ground punctation denser in apical half, with sparse secondary punctures principally in basal half. Male genitalia (Figs 15D–F, H, K–L): T8 distinctly shorter than broad, sides subparallel, basal emargination broad, apical emargination narrow, shallow, with ventrolateral apodemes separated by about two-thirds maximum T8 width, extending about two-thirds distad beneath; S8 divided, short, inner margins approximate at base, strongly divergent to apex, bearing conspicuous fringe of setae along apical one-third, outer margins subparallel, to weakly divergent, apical guides well developed in apical half, apices broadly rounded; T9 with basal apodemes thin, about one-half total length, T9 apices narrow, bluntly subacute, weakly opposed, glabrous, ventrolateral apodemes moderately strongly projecting beneath; S9 very weakly widened in basal two-thirds, desclerotized along midline, head broad, with apicolateral points short, subparallel; tegmen with sides broadly rounded in basal half, weakly narrowed to apex, dorsal surface near base narrowly, transversely impressed, dorsobasal edge weakly arcuate, tegmen weakly sinuate in lateral aspect; median lobe broad, about two-thirds tegmen length, bearing conspicuous fine denticles on dorsal surface; basal piece nearly one-half tegmen length.

**Figure 16.**
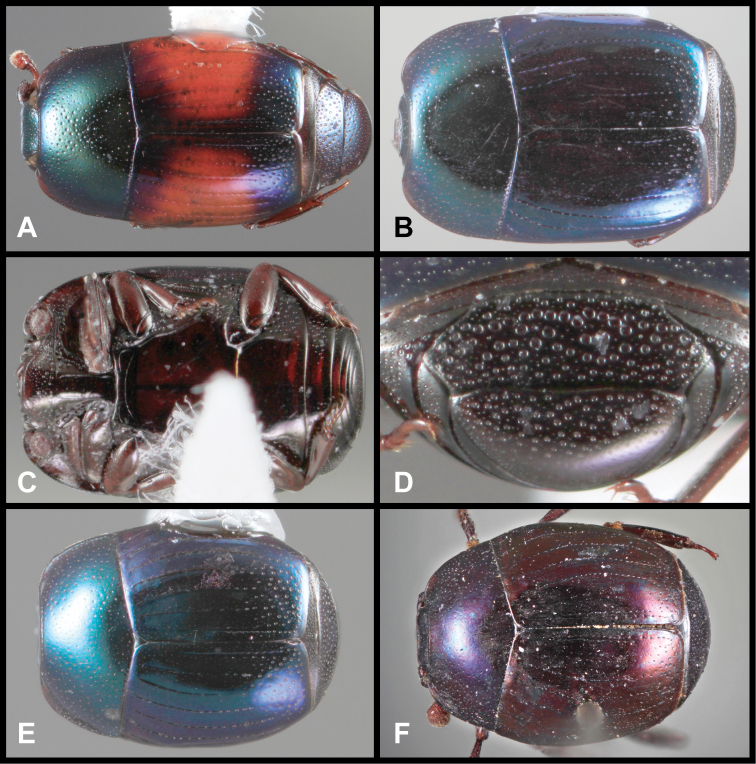
*Baconia godmani* group. **A** Dorsal habitus of *Baconia maculata* **B** Dorsal habitus of *Baconia deliberata* **C **Ventral habitus of *Baconia deliberata* **D** Pygidia of *Baconia deliberata* **E** Dorsal habitus of *Baconia excelsa* **F** Dorsal habitus of lectotype of *Baconia violacea*.

###### Remarks.

While this species’ male genitalia associates it clearly with the *Baconia godmani* group, in external characters it is highly distinctive, with its narrow elongate body form and red-maculate elytra (Fig. 16A).

###### Etymology.

This species is named for its distinctive elytral maculations.

##### Baconia deliberata sp. n.

http://zoobank.org/9AA5A279-ED81-4A7D-9C2C-52A50D66CA26

http://species-id.net/wiki/Baconia_deliberata

Figs 16B–D 17A–C, G, I–J Map 5

###### Type locality.

ECUADOR: Orellana:Res. Ethnica Waorani [0.67°N, 76.43°W].

###### Type material.

**Holotype male**: “**ECUADOR: Depto. Orellana**:Res. Ethnica Waorani, 1km S Onkone Gare Camp, Trans. Ent., 0°39'10"S, 76°26'W, 220m, 26 June 1996, T.L. Erwin et al. collectors” / “Insecticidal fogging of mostly bare green leaves, some with covering of lichenous or bryophytic plants in terra firme forest. Project MAXUS **Lot 1582 Trans. 7 Sta. 2**” / “Caterino/Tishechkin Exosternini Voucher EXO-00438” (USNM). **Paratype** (1): same locality as type, 21.i.2006, Lot 3115, Trans. 2, Sta. 6 (USNM).

###### Diagnostic description.

**Figure 17.**
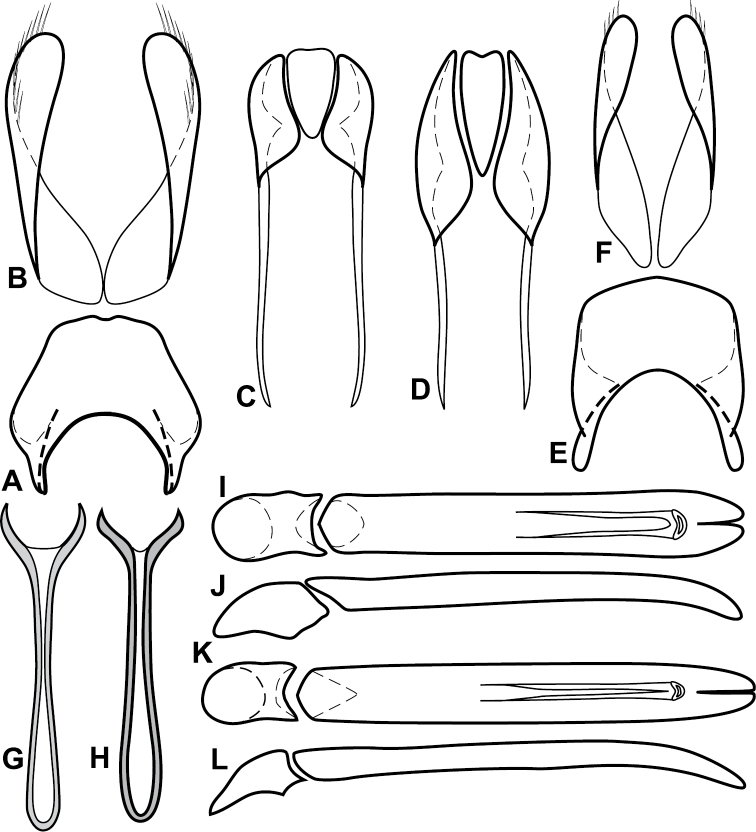
Male genitalia of *Baconia godmani* group. **A** T8 of *Baconia deliberata* **B** S8 of *Baconia deliberata* **C** T9 & T10 of *Baconia deliberata* **D** T9 & T10 of *Baconia excelsa* **E** T8 of *Baconia excelsa* **F** S8 of *Baconia excelsa* **G** S9 of *Baconia deliberata* **H** S9 of *Baconia excelsa* **I** Aedeagus, dorsal view of *Baconia deliberata* **J** Aedeagus, lateral view of *Baconia deliberata* **K **Aedeagus, dorsal view of *Baconia excelsa* **L** Aedeagus, lateral view of *Baconia excelsa*.

Length: 1.7–1.9mm, width: 1.3–1.5mm; body elongate oval, subparallel-sided, depressed, glabrous; head and pronotum metallic greenish-blue, elytra blue, pygidia and venter rufopiceous; frons elevated over antennal bases, depressed at middle, ground punctation fine, with few coarse punctures at middle and near vertex, frontal stria present along inner margin of eye, curving inward at front, interrupted at middle; antennal scape short, club asymmetrically oblong; epistoma truncate apically; labrum about 4×wider than long, weakly bisinuate along apical margin; both mandibles with acute basal tooth; pronotal sides weakly arcuate to apex, marginal stria complete along lateral and anterior margins, lateral submarginal stria absent, ground punctation of pronotal disk fine, with coarse secondary punctures in lateral thirds; elytra with two complete epipleural striae, outer subhumeral stria absent, inner subhumeral stria present in basal third and as weak fragment behind middle, dorsal striae 1–5 more or less complete, 5^th^ stria may be weakly abbreviated from base, sutural stria present in about apical third, elytral disk with few coarse punctures in apical fifth; prosternum moderately broad, weakly convex, keel truncate at base, carinal striae complete, bent inward at base, weakly impressed along basal margin, subparallel anterad; prosternal lobe about two-thirds keel length, apical margin rounded, marginal stria obsolete at sides; mesoventrite emarginate at middle, marginal stria broadly interrupted; mesometaventral stria arched strongly forward, not crenulate, detached at sides, inner lateral metaventral stria extending from near mesocoxa obliquely posterolaterad toward middle of metacoxa, slightly sinuate apically, outer lateral metaventral stria absent, metaventral disk impunctate at middle; abdominal ventrite 1 with single, complete lateral stria, middle portion of disk lacking coarse punctures; protibia 4-dentate, the basal denticle weak, outer margin serrulate between teeth; mesotibia with two weak marginal spines; outer metatibial margin smooth; propygidium without transverse basal stria but with rather distinct basal series of large, close, ocellate punctures, discal punctures otherwise more or less uniformly separated by about 0.5–1× their diameters; propygidial gland openings evident about one-third from anterior margin, and almost one-third from each lateral margin; pygidium with ground punctation very fine but rather dense in apical half, secondary punctation conspicuous in basal third. Male genitalia (Figs 17A–C, G, I–J): T8 slightly shorter than broad, sides widened in basal one-third, convergent to apex, basal emargination broadly rounded, apical emargination inconspicuous, ventrolateral apodemes separated by about one-half maximum T8 width, extending about one-half distad beneath, obsolete in apical half; S8 much longer than T8, divided, inner margins approximate at base, strongly divergent apically, bearing conspicuous fringe of setae along apical one-third, outer margins subparallel to weakly divergent, apical guides well developed from base, broadly rounded apically; T9 with basal apodemes thin, almost two-thirds total length, T9 apices narrowly rounded, glabrous, ventrolateral apodemes moderately strongly projecting beneath; S9 stem thin at middle, widened in basal half, head broad, broadly curved to apicolateral points, desclerotized along midline; tegmen with sides subparallel throughout, very weakly widened near apex, dorsobasal edge projecting, tegmen in lateral aspect more or less straight, weakly curved ventrad at apex; median lobe about one-third tegmen length; basal piece about one-fifth tegmen length.

###### Remarks.

This species shares male genitalic characters with several others in this group (*Baconia godmani*, *Baconia rossi*, *Baconia scintillans*), but is distinctive in its small size, parallel-sided form (Fig. 16B), presence of complete elytra striae 1-5, noncrenulate mesometaventral stria (Fig. 16C), and series of punctures in place of both the basal propygidial stria (Fig. 16D) and the lateral submarginal pronotal stria.

###### Etymology.

This species’ name means determined or resolved.

##### Baconia excelsa sp. n.

http://zoobank.org/4D865F98-0FE3-45B5-B75F-AB839B2FAD95

http://species-id.net/wiki/Baconia_excelsa

Figs 16E 17D–F, H, K–L Map 5

###### Type locality.

ECUADOR: Orellana: Tiputini Biodiversity Station [0.635°S, 76.150°W].

###### Type material.

**Holotype male**: “**ECUADOR: Depto.** **Orellana**: Tiputini Biodiversity Station, 0°37'55"S, 76°08'39"W, 220-250m, 6 February 1999 T.L.Erwin *et. al*. collectors” / “insecticidal fogging of mostly bare green leaves, some with covering of lichenous or bryophytic plants **Lot 2075 Trans. 8 Sta. 6**” / “Caterino/Tishechkin Exosternini Voucher EXO-00432” (USNM). **Paratypes** (2): 1: **ECUADOR: Orellana:** Res. Ethnica Waorani, 1 km S Onkone Gare Camp, Trans. Ent., 0°39'10"S, 76°26'W, 220 m, 26.vi.1996, fogging, T. Erwin (USNM), 1: 30.ix.1996, fogging, T. Erwin (USNM).

###### Diagnostic description.

Length: 1.6–1.7mm, width: 1.3–1.4mm; body elongate oval, weakly depressed, glabrous; head and pronotum metallic greenish-blue, elytra and pygidia blue, venter rufopiceous; frons weakly elevated over antennal bases, depressed at middle, ground punctation rather coarse, with numerous coarser punctures at middle and near vertex, frontal stria present along inner margin of eye, curving inward at front, interrupted over antennal bases and at middle; antennal scape short, club rounded; epistoma truncate apically; labrum about 4×wider than long, weakly emarginate apically; both mandibles with acute basal tooth; pronotal sides weakly arcuate to near apex, somewhat abruptly bent inward in apical fifth, marginal stria complete along lateral and anterior margins, lateral submarginal stria represented by semiregular series of deeply impressed punctures, continuous with sublinear depression in anterior corner, ground punctation of pronotal disk fine, with slightly coarser sparsely present nearly throughout, though less conspicuous at middle; elytra with two complete epipleural striae, outer subhumeral stria absent, inner subhumeral stria present in basal fourth and as median fragment, dorsal striae 1–5 more or less complete, progressively abbreviated from apex, 5^th^ stria obsolete in about apical third, sutural stria present in apical two-thirds, elytral disk with few coarse punctures in apical third; prosternum weakly convex, keel shallowly emarginate at base, carinal striae complete, divergent anterad and posterad; prosternal lobe about two-thirds keel length, apical margin narrowly rounded, marginal stria obsolete at sides; mesoventrite weakly projecting at middle, marginal stria complete; mesometaventral stria arched forward, crenulate, narrowly detached at sides, inner lateral metaventral stria curving from near mesocoxa posterolaterad toward outer third of metacoxa, sinuate medially, outer lateral metaventral stria may be vaguely indicated by short series of connected punctures, metaventral disk impunctate at middle; abdominal ventrite 1 with single, complete lateral stria, middle portion of disk lacking coarse punctures, series of fine punctures more or less conspicuous along apical margin; protibia 4-5 dentate, basal denticles weak, outer margin serrulate between teeth; mesotibia with two weak marginal spines; outer metatibial margin smooth; propygidium without transverse basal stria, discal punctures moderately large, ocellate, mostly separated by about 0.5–1× their diameters; propygidial gland openings evident about one-third from anterior margin, and almost one-third from each lateral margin; pygidium with ground punctation fine, conspicuous in apical half, secondary punctation limited to basal half. Male genitalia (Figs 17D–F, H, K–L): T8 slightly longer than broad, sides subparallel, basal emargination broadly rounded, apical emargination inconspicuous, ventrolateral apodemes separated by about one-half maximum T8 width, extending about one-half distad beneath, strongly narrowed in apical half; S8 longer than T8, divided, inner margins approximate at base, strongly divergent apically, bearing fine, rather inconspicuous fringe of setae along apical one-fifth, outer margins subparallel to weakly divergent, apical guides well developed from just beyond base, narrowly rounded apically; T9 with basal apodemes thin, about one-half total length, T9 apices narrowly rounded, glabrous, ventrolateral apodemes weakly projecting beneath; S9 stem thin at middle, widened in basal half, head wide but weak, with weakly curved apicolateral points, strongly desclerotized along midline; tegmen narrow, with sides subparallel throughout, dorsobasal edge arcuate, tegmen in lateral aspect more or less straight, weakly curved ventrad at apex; median lobe about one-half tegmen length; basal piece about one-sixth tegmen length.

###### Remarks.

*Baconia excelsa* can be distinguished by its pattern of elytral striation (Fig. 16E), with striae 1–5 present to the base, but with 3–5 variably abbreviated from their apices, and the sutural stria deeply impressed in the apical two-thirds (also slightly abbreviated from the apex). Unusual characters also include the series of lateral submarginal pronotal punctures, not forming a distinct stria, but lying within a slightly linear depression.

###### Etymology.

This species’ name means high or lofty, as in the canopy from which it was collected.

##### Baconia violacea

(Marseul, 1853)

http://species-id.net/wiki/Baconia_violacea

Fig 16F Map 5

Phelister violaceus Marseul, 1853: 469; *Phelister violaris*Marseul 1857: 457 (emend.). *Baconia violacea*: Mazur 1984: 281.

###### Type locality.

NOUVELLE GRENADE [including parts of Colombia and Venzuela; exact locality uncertain].

###### Type material.

**Lectotype**, sex undetermined, here designated (MNHN): “*Phelister violaris* M., N. Gren. … [illegible]” / “TYPE” / “Museum Paris Coll. de Marseul 2842-90” / “LECTOTYPE *Phelister violaceus* Marseul, 1853, M.S.Caterino & A.K.Tishechkin des. 2010”.

Marseul’s poor labeling and inconsistent writings leave considerable room for question about the original identities of this and the following species. The specimen we designate as lectotype of *Phelister violaceus* is not labeled with that name anywhere, but only with *‘Phelister violaris’*, a nomen nudum that Marseul accidentally used in subsequent publications, referring evidently to this species. However, the specimen we designate as lectotype of *Phelister varicolor* has both *violaris* and *violaceus* on the original data label, and *‘varicolor’* only on a secondary label, which does not look exactly like Marseul’s handwriting. The type localities for the two are identical (though vague), and no other identifying data is given in the publication (though the labels are not otherwise identical). In addition the species are extremely similar and not adequately distinguished by the original descriptions. Our interpretation is that all the specimens were originally designated as *violaris* (at the time presumably only a manuscript name), that for the publication Marseul emended that to *violaceus*, and that several years later he decided that one of the original *violaceus* specimens was distinct and was named as *varicolor* (with a separate label applied at that time.) We have no strong evidence for this, however, and must admit that we cannot be certain which specimens went with which name. Our lectotype designations will help to alleviate this ambiguity.

###### Diagnostic description.

Length: [not measured, ~2.3mm], width: [not measured, ~1.5mm]; body elongate oval, weakly depressed, glabrous; dorsum metallic, nonuniformly in lectotype, with head, pronotum and pygidia metallic blue, elytra contrastingly bronzy-violet, venter rufobrunneus to faintly metallic; frons elevated over antennal bases, rather strongly depressed along antero-posterior midline, ground punctation rather conspicuous, with moderately large secondary punctures within frontal depression, frontal stria present along inner margin of eye, complete across front; antennal scape short, club rounded; epistoma slightly convex along apical margin, truncate to weakly emarginate; labrum about 3×wider than long, weakly emarginate along apical margin; pronotal sides rather strongly convergently arcuate to apex, depressed in anterior corners, marginal stria complete along lateral and anterior margins, lateral submarginal stria absent, ground punctation of pronotal disk rather conspicuous, interspersed with small secondary punctures across front and toward sides; elytra with three complete epipleural striae, outer subhumeral stria absent, inner subhumeral stria present at base, dorsal striae 1–4 complete, 5^th^ stria absent, sutural stria more or less complete, elytral disk with very few coarse punctures in apical fifth; prosternum rather narrow, weakly convex, keel shallowly emarginate at base, carinal striae complete, divergent anterad and posterad, separate throughout; prosternal lobe about one-half keel length, apical margin bluntly rounded, marginal stria nearly complete; mesoventrite produced at middle, marginal stria complete, mesometaventral stria complete, transverse, finely crenulate, meeting lateral metaventral stria, which curves posterolaterad toward middle of metacoxa, outer lateral metaventral stria short, oblique, metaventral disk impunctate at middle; abdominal ventrite 1 with single lateral stria, slightly abbreviated apically, middle portion of disk impunctate; protibia 4 dentate, basal denticle weak, outer margin serrulate between teeth; mesotibia with two marginal spines; outer metatibial margin smooth; propygidium without basal stria, discal punctures rather small, ocellate, denser in basal two-thirds; propygidial gland openings inconspicuous; pygidium with ground punctation interspersed with small secondary punctures throughout, denser basad. Male genitalia: not known.

###### Remarks.

As delineated here, the only distinctive characteristics of *Baconia violacea* are the pattern of elytral striae, with 1-4 and the sutural striae complete (Fig. 16F), the complete frontal stria, and the presence of a fine, transverse mesometaventral stria. In *Baconia varicolor*, the sutural stria is abbreviated from the base, the frontal stria is interrupted at the middle, and the mesometaventral stria is subangulately arched forward at the middle. The coloration of the lectotype of *Baconia violacea* is distinctive, but it is impossible to know how consistent that might be. Lewis (1888) reported this species from Guatemala in the BCA. However, we assign those specimens to *Baconia varicolor*.

##### Baconia varicolor

(Marseul, 1887)

http://species-id.net/wiki/Baconia_varicolor

**Figure 18.**
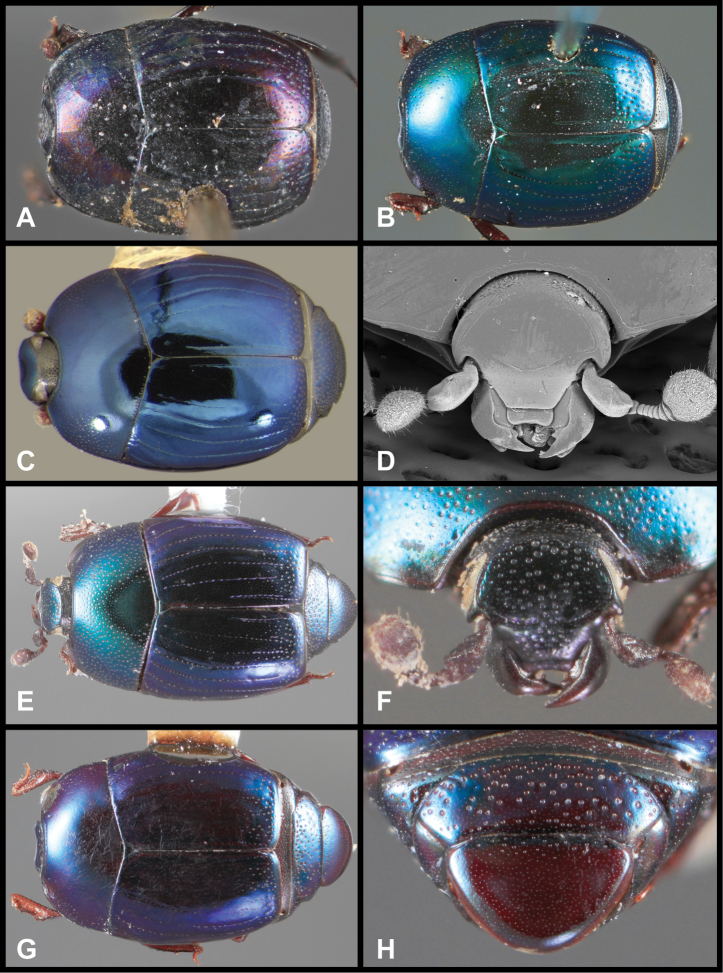
*Baconia godmani* group. **A** Dorsal habitus of lectotype of *Baconia varicolor* **B** Dorsal habitus of lectotype of *Baconia dives* **C** Dorsal habitus of *Baconia eximia* **D** Frons of *Baconia eximia* **E** Dorsal habitus of *Baconia splendida* **F** Frons of *Baconia splendida* **G** Dorsal habitus of *Baconia jacinta* **H** Pygidia of *Baconia jacinta*.

Figs 18A 19A–B, G, I, L–M

Phelister varicolor Marseul, 1887b: cxlvii; *Baconia varicolor*Mazur 1984: 281.

###### Type locality.

NOUVELLE GRENADE [exact locality uncertain].

###### Type material.

**Lectotype male**, here designated (MNHN): “*Phelister violaris* M violaceus, N.Gren…[illegible]” / “*Phelister violaceus* ….[illegible]” / “*varicolor*” / “TYPE” / “LECTOTYPE *Phelister varicolor* Marseul, 1887, M.S.Caterino & A.K.Tishechkin des. 2010”. See discussion under the preceding species for information on this lectotype.

###### Other material.

2: **BRAZIL**: **Santa Catarina**: 14.v.1988 (BMNH), 3: no date (BMNH). 1: **COLOMBIA** (BMNH). 3: **GUATEMALA**: S. Geronimo, G. Champion (BMNH).

###### Diagnostic description.

**Figure 19.**
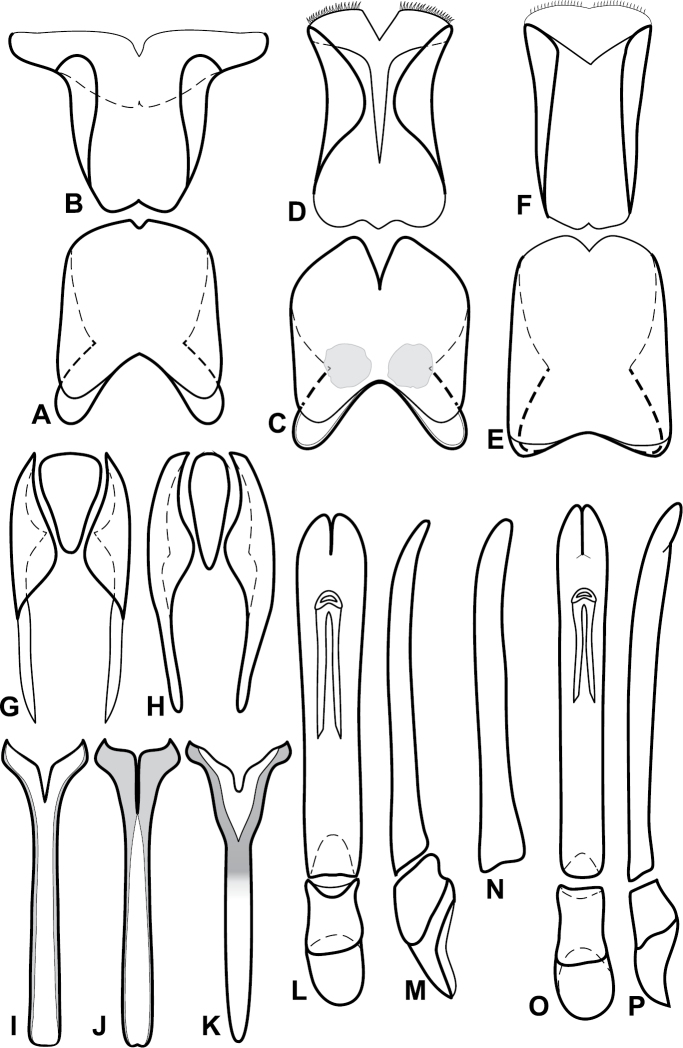
Male genitalia of *Baconia godmani* group. **A** T8 of *Baconia varicolor* **B** S8 of *Baconia varicolor* **C** T8 of *Baconia dives* **D** S8 of *Baconia dives* **E** T8 of *Baconia eximia* **F** S8 of *Baconia eximia* **G** T9 & T10 of *Baconia varicolor* **H** T9 & T10 of *Baconia eximia* **I** S9 of *Baconia varicolor* **J** S9 of *Baconia dives* **K** S9 of *Baconia eximia* **L** Aedeagus, dorsal view of *Baconia varicolor* **M** Aedeagus, lateral view of *Baconia varicolor* **N** Aedeagus, lateral view of *Baconia dives* **O** Aedeagus, dorsal view of *Baconia eximia* **P** Aedeagus, lateral view of *Baconia eximia*.

Length: 2.2–2.5mm, width: 1.9–2.1mm; body elongate oval, weakly depressed, glabrous; dorsum metallic, varied in color from bronzy to greenish-blue to blue or blue-violet; frons elevated over antennal bases, rather strongly depressed along antero-posterior midline, ground punctation rather conspicuous, with moderately large secondary punctures within frontal depression, frontal stria present along inner margin of eye, may be complete, but usually fragmented across front, supraorbital stria absent; antennal scape short, club slightly asymmetrically oblong; epistoma slightly convex along apical margin, truncate to weakly emarginate; labrum about 3×wider than long, weakly emarginate to bisinuate along apical margin; both mandibles with acute basal tooth; pronotal sides weakly convergent in basal half, arcuate to apex, depressed in anterior corners, marginal stria complete along lateral and anterior margins, lateral submarginal stria absent, ground punctation of pronotal disk rather conspicuous, interspersed with small secondary punctures toward sides, especially dense, subserially arranged near margin; elytra with two complete epipleural striae, outer subhumeral stria absent, inner subhumeral stria present as basal and rarely median fragments, dorsal striae 1–4 complete, 4^th^ stria arched mediad at base, variably, slightly abbreviated from apex, 5^th^ stria absent, sutural stria present in apical half or more, elytral disk with few coarse punctures in apical fourth; prosternum weakly convex, keel weakly emarginate at base, carinal striae complete, divergent anterad and posterad, separate throughout; prosternal lobe about one-half keel length, weakly deflexed, apical margin rather narrowly rounded, marginal stria obsolete at sides; mesoventrite weakly produced, marginal stria complete, mesometaventral stria subangulately arched forward, narrowly detached at sides; lateral metaventral stria extending from near mesocoxa posterolaterad toward inner third of metacoxa, sinuate apically, outer lateral metaventral stria short, oblique, metaventral disk impunctate at middle; abdominal ventrite 1 with single, complete lateral stria, middle portion of disk impunctate; protibia 4–5 dentate, basal denticles weak, outer margin serrulate between teeth; mesotibia with two weak marginal spines; outer metatibial margin smooth; propygidium without basal stria, discal punctures rather small, ocellate, denser in basal half; propygidial gland openings inconspicuous; pygidium with ground punctation rather dense in apical half, interspersed with small secondary punctures in basal third only. Male genitalia (Figs 19A–B, G, I, L–M): T8 slightly longer than broad, sides weakly convergent, more or less straight, basal emargination narrowly rounded, apical emargination inconspicuous, ventrolateral apodemes separated by about one-half maximum T8 width, extending beneath to about longitudinal midpoint, narrowed in apical half; S8 halves fused along midline, with broadly expanded, membraneous apical velum, not obviously setose, outer margins subparallel to weakly divergent, apical guides moderately well developed from just beyond base, narrowly rounded apically; T9 with basal apodemes thin, about one-half total length, apices acute, glabrous, ventrolateral apodemes acutely projecting beneath; S9 stem narrowest about two-thirds from base, very weakly expanded to base, strongly desclerotized along midline; tegmen with sides widest near apex, sinuately narrowed toward base, tegmen in lateral aspect weakly curved ventrad in apical fourth; median lobe nearly one-half tegmen length; basal piece about one-third tegmen length.

###### Remarks.

This species is very hard to characterize. The description above is based on the lectotype (Fig. 18A), but other specimens considered to be this species vary in a number of significant characters. The name alone posits a variable species, but whether this variation is real or if it has been confused with close relatives is very hard to say. Male genitalia of the type are unique, with a broadly apically widened S8, but genitalia are not available for most other purported specimens of the species. So even our own non-type identifications are tentative. The species is most closely related to *Baconia eximia* and *Baconia dives*, all of which share a united male 8^th^ sternite.

##### Baconia dives

(Marseul, 1861)

http://species-id.net/wiki/Baconia_dives

Figs 18B 19C–D, J, N Map 5

Phelister dives Marseul, 1861: 157 (cited in error as ‘Marseul, 1862: 706’ by Mazur 1997); *Baconia dives*: Mazur 1984: 280.

###### Type locality.

BRAZIL: Rio de Janeiro: [22.9°S, 43.2°W].

###### Type material.

**Lectotype**, sex undetermined, here designated (NHRS): “Rio Jan.” / “F. Sahlb.” / “Type” / “Typus” / “NHRS-VKBS 000000004” / “6903 E91 +” / “LECTOTYPE *Phelister dives* Marseul, M.S.Caterino & A.K.Tishechkin des. 2010”. **Paralectotype:** same data as type, NHRS-VKBS000000005. This species was described from an unspecified number of specimens, and the lectotype designation fixes primary type status on one of the original specimens.

###### Other material.

**BOLIVIA**, 1: **Santa Cruz**:Hotel Flora y Fauna, 4-5 km SSE Buena Vista, 17°29.925'S, 63°39.128'W, 440 m, 6–15.xii.2003, FIT, forest, S. & J. Peck (CMNC). **BRAZIL**: 1: **Amazonas**: Igarape Belem, nr. Rio Solimoes, 70 km E, of Leticia, 18–28.v.1970, under bark, B. Malkin (FMNH); 1: Rio Janauaca, 40 km SW Manaus, 03°20'S, 060°17'W, 10.iii.1979, fogging white water inundation forest canopy, T. Erwin (USNM); 1: **Pará**: Santarém (CMNH). **COLOMBIA**, 2: **Meta**: Villavicencio, 11.vii.1938, under bark, H. Dybas (FMNH); 1: 13.vii.1938, C. Seevers (USNM), 1: 25.vii.1938, under bark, H. Dybas (FMNH). **FRENCH GUIANA**:Belvèdére de Saül, 3°1'22"N, 53°12'34"W, 10.xii.2010, FIT, SEAG (CHND). **MEXICO**: 1: **San Luis Potosí**:intercepted in route to Texas, orchid del maiz (USNM). **VENEZUELA**, 2: **Anzoátegui**: Los Naranjos, Rio Neveri (+ or -) 900 m, 25.viii.1966, L. Joly (MHNLS). **PERU: Loreto**: Iquitos, Rio Nanay, 4–6.ii.1984, L. Huggert (MHNG).

###### Diagnostic description.

Length: 2.2–2.6mm, width: 1.9–2.2mm; body elongate oval, weakly depressed, glabrous; dorsum metallic, more or less uniformly blue to blue-violet; frons elevated over antennal bases, rather strongly depressed along antero-posterior midline, ground punctation rather conspicuous, with few moderately large secondary punctures within frontal depression, frontal stria present along inner margin of eye, curving mediad, interrupted at middle, supraorbital stria absent; antennal scape short; epistoma depressed in middle, slightly convex along distal margin, truncate to weakly emarginate; labrum about 3×wider than long, weakly emarginate to bisinuate along apical margin; both mandibles with acute basal tooth; pronotal sides increasingly arcuate to apex, depressed in extreme anterior corners, marginal stria complete along lateral and anterior margins, crenulate in front, lateral submarginal stria absent, ground punctation of pronotal disk rather conspicuous, interspersed with small secondary punctures along front and toward sides; elytra with three complete epipleural striae, outer subhumeral stria absent, inner subhumeral stria present as basal and median fragments, dorsal striae 1–4 more or less complete, inner striae weakly abbreviated from apices, 4^th^ stria arched mediad at base, 5^th^ stria mostly absent, may be represented by basal puncture, sutural stria present in apical two-thirds, elytral disk with coarse punctures in apical fourth; prosternum weakly convex, keel weakly emarginate at base, carinal striae complete, divergent anterad and posterad, separate throughout; prosternal lobe slightly over one-half keel length, weakly deflexed, apical margin rather narrowly rounded, marginal stria obsolete at sides; mesoventrite weakly produced at middle, marginal stria complete, mesometaventral stria arched forward, crenulate, interrupted at middle, laterally meeting lateral metaventral stria, which extends posterolaterad toward middle of metacoxa, sinuate apically, outer lateral metaventral stria very short, oblique, metaventral disk impunctate at middle; abdominal ventrite 1 with single lateral stria, abbreviated apically, middle portion of disk impunctate; protibia 4–5 dentate, basal denticles weak, outer margin serrulate between teeth; mesotibia with one prominent and one weak marginal spine; outer metatibial margin smooth; propygidium without basal stria, discal punctures rather small, ocellate, separated by 1–2× their diameters, sparser apically; propygidial gland openings rather conspicuous one-third from basal and lateral margins; pygidium with ground punctation very fine, interspersed with small secondary punctures mainly in basal third. Male genitalia (Figs 19C–D, J, N): T8 slightly longer than broad, sides subparallel, more or less straight, with superficial dorsal sclerotizations in basal half, basal emargination narrowly rounded, apical emargination deep, subacute, basal rim slightly explanate, ventrolateral apodemes separated by about one-half maximum T8 width, extending beneath to about one-third from base; S8 elongate, halves fused along midline, with weakly expanded, membraneous apical velum bearing a dense setal fringe, apical guides moderately well developed, widest near middle, narrowly rounded apically; T9 with basal apodemes moderately thick, about one-half total T9 length, apices subacute, glabrous, ventrolateral apodemes weakly produced beneath; S9 stem parallel-sided, strongly desclerotized along midline; tegmen with sides widest near apex, sinuately narrowed toward base (as in *Baconia varicolor*), in lateral aspect slightly thickened near middle, very weakly curved toward apex; median lobe nearly one-half tegmen length; basal piece about one-third tegmen length.

###### Remarks.

*Baconia dives* is a relatively large, convex, laterally rounded species (Fig. 18B). It is best recognized by the elytral striation, with the inner subhumeral and striae 1–4 largely complete (though they may be weakly abbreviated from their apices), with the base of the 4^th^ stria arched slightly mediad, the 5^th^ stria represented by only a basal puncture, and the sutural stria present in the apical two-thirds. It lacks submarginal pronotal or basal propygidial striae, and has its mesometaventral stria weakly arched forward and finely crenulate across the middle (rarely interrupted). In this species, as well as in *Baconia eximia*, *Baconia varicolor* (and perhaps a few others of the above whose males are not known), the halves of the 8^th^ sternite are fused along part of the midline.

##### Baconia eximia

(Lewis, 1888)

http://species-id.net/wiki/Baconia_eximia

Figs 18C–D 19E–F, H, K, O–P Map 5

Phelister eximius Lewis, 1888: 191; *Baconia eximia*: Mazur 1984: 280.

###### Type locality.

NICARAGUA: Chontales [exact locality uncertain].

###### Type material.

**Holotype**, sex undetermined (BMNH): “Chontales. Janson” / “Sp. figured” / “*eximius* Lewis Type” / “B.C.A.,Col.,II,(1). *Phelister*”. This species was explicitly described from a single specimen.

###### Other material.

**BELIZE**, 1: **Cayo**: Las Cuevas Res. Sta., 16°44.33'N, 88°59.07'W, 550 m, 28.v.2000, FIT, M. Caterino, DNA Extract MSC-0065, EXO-01142; 1: **Orange Walk**: Rio Bravo Cons. Area, 25–30.iv.1996, FIT, C.E. Carlton (CHPWK). **GUATEMALA**: 2: **Zacapa**: Santa Clara, in interior valley of Sierra de las Minas (N. of Cabanas), 5500 ft, 9.viii.1948, under bark, R. Mitchell (FMNH); 1: Santa Cruz, Marble Quarry rd, NE Teculutan, 15°04.454'N, 89°41.074'W, 1539m, 17.v.2006, R.S. Zack (WSUC). **MEXICO**: 1: **Veracruz**: 15 mi W Tlapacoyan, 28.ii.1972, F. Parker & D. Miller (CHND); 1: **Quintana Roo**: Chetumal, 24.x.2004, M. Sawoniewicz (MHNG), 1: 30.x.2004, M. Sawoniewicz (MHNG). **NICARAGUA**, 1: **Granada**: Res. Nat. Volcan Mombacho, entrance rd, 11°50.05'N, 85°58.83'W, 910 m, 1–5.vi.2002, FIT, R. Brooks, Z. Falin & S. Chatzimanolis (SEMC); 1: **Rio San Juan**: Ref. Bartola, 8 km SE El Castillo , 10°58.6'N, 84°20.4'W, 30 m, 25-31.v.2002, FIT, rainforest, S. Peck (CMNC). **PANAMA**:1: **Colón**: P. N. San Lorenzo, Achiote, Cafetal A Dist. , 9°12'N, 79°58'W, 10 m, 12-26.v.2008, FIT, A. Mercado (AKTC); 1: P. N. San Lorenzo, Achiote, Pastizal B Dist., 9°12'N, 79°59'W, 0 m, 12-27.v.2008, FIT, A. Mercado (GBFM); 1: Barro Colorado Island, 9°10'N, 79°50'W, 15–27.v.1972, T. & L. Erwin (CHND); 1: Barro Colorado Island, 9°11'N, 79°51'W, 15.vii.1994, FIT, D. Banks (SEMC), 1: 1-10.viii.2005, J. McHugh, DNA Extract MSC-1901, EXO-00431, 1: 1–10.viii.2005, J. McHugh, N. Nguyen & C. Rodriguez (MSCC), 1: 14.vii.1969, in rotting fruit, W. Overal (FMNH).

###### Diagnostic description.

Length: 2.2–2.6mm, width: 2.2–2.3mm; body elongate oval, weakly depressed, glabrous; dorsum metallic blue, head and pronotum greenish-blue, contrasting slightly with elytra; frons transversely elevated between antennal bases, weakly depressed behind, interocular margins strongly convergent dorsad, ground punctation fine, with few secondary punctures near vertex, frontal stria present along inner margin of eye, interrupted over antennal bases, but complete across middle, supraorbital stria absent; antennal scape short, club broadly rounded; epistoma weakly concave below frontal ridge, apical margin truncate to weakly emarginate; labrum about 3×wider than long, weakly emarginate apically; both mandibles with acute basal tooth; pronotal sides weakly convergent in basal half, somewhat abruptly arcuate to apex, weakly depressed in anterior corners, marginal stria complete along lateral and anterior margins, lateral submarginal stria absent, ground punctation of pronotal disk very fine, interspersed with small secondary punctures in lateral third; elytra with three complete epipleural striae, outer subhumeral stria absent, inner subhumeral stria present in basal fourth, dorsal striae 1-3 complete, 4^th^ stria variably abbreviated from apex, occasionally absent, 5^th^ stria absent, sutural stria present in about apical three-fourths, elytral disk with few coarse punctures in apical fourth; prosternum moderately broad, weakly convex, keel very weakly emarginate at base, carinal striae complete, divergent anterad and posterad, separate throughout; prosternal lobe rather short, about one-half keel length, apical margin broadly rounded, marginal stria deeply impressed at middle, fragmented at sides; mesoventrite weakly produced at middle, marginal stria interrupted, mesometaventral stria absent; lateral metaventral stria extending from near mesocoxa posterolaterad toward middle of metacoxa, slightly abbreviated, sinuate apically, outer lateral metaventral stria short, oblique, metaventral disk impunctate at middle; abdominal ventrite 1 with single lateral stria abbreviated apically, middle portion of disk impunctate; protibia weakly 4–5 dentate, outer margin serrulate between teeth; mesotibia with two weak marginal spines; outer metatibial margin smooth; propygidium without basal stria, discal punctures rather small, ocellate, denser in basal half; propygidial gland openings evident about one-fourth from anterior margin, one-third from lateral margins; pygidium with fine ground punctation uniformly interspersed with small secondary punctures. Male genitalia (Figs 19E–F, H, K, O–P): T8 slightly longer than broad, sides subparallel, more or less straight, basal emargination shallowly rounded, apical emargination shallow, narrow, subacute, ventrolateral apodemes separated by about one-half maximum T8 width, extending beneath to about one-third from base; S8 elongate, halves fused along midline, with membraneous apical velum bearing a fine but dense setal fringe, apical guides weakly developed, widest near apex; T9 with basal apodemes moderately thick, about one-third total T9 length, apices bent mediad, narrowly rounded, glabrous, ventrolateral apodemes weakly produced beneath; S9 stem parallel-sided in basal two-thirds, expanded abruptly, then more gradually to narrow, subquadrate, divergent apices, more strongly sclerotized in apical half, apical margin shallowly divided; tegmen long, narrow, widest near apex, in lateral aspect slightly thickened in apical half, very weakly curved over entire length; median lobe about one-third tegmen length; basal piece about one-third tegmen length.

###### Remarks.

Among those few *Baconia godmani* group species having the halves of the male 8^th^ sternite fused, *Baconia eximia* can be recognized by its distinctive, more or less complete transverse frontal elevation (Fig. 18D). Other helpful characters include the 4^th^ elytral stria being abbreviated from apex (Fig. 18C) and the absence of the mesometaventral stria. There is some evident variation among known specimens, with those from Nicaragua showing much finer propygidial and pygidial punctation.

##### Baconia splendida sp. n.

http://zoobank.org/A5CE09DD-04EC-443D-832B-EB59D342958A

http://species-id.net/wiki/Baconia_splendida

Figs 18E–F Map 6

###### Type locality.

ECUADOR: Orellana:Res. Ethnica Waorani [0.67°N, 76.43°W].

###### Type material.

**Holotype female**: “**ECUADOR: Depto. Orellana**:Res. Ethnica Waorani, 1km S Onkone Gare Camp, Trans. Ent., 0°39'10"S, 76°26'W, 220m, 22 January 1994, T.L. Erwin *et al*. collectors” / “Insecticidal fogging of mostly bare green leaves, some with covering of lichenous or bryophytic plants in terra firme forest. Project MAXUS **Lot 624 Trans. 5 Sta. 2**” / “Caterino/Tishechkin Exosternini Voucher EXO-00435” (USNM).

###### Diagnostic description.

Length: 2.3mm, width: 1.9mm; body elongate oval, subparallel-sided, moderately strongly depressed, glabrous; head and pronotum metallic greenish-blue, elytra brightly metallic blue, pygidia intermediately (in color) greenish-blue, venter rufo-brunneus; frons weakly elevated over antennal bases, weakly depressed along midline, ground punctation rather conspicuous, with numerous coarser punctures on epistoma, within frontal depression, and toward vertex; frontal stria present along inner margin of eye and across front, but interrupted at middle, frontal-vertical margin sharp, supraorbital stria absent; antennal scape short, apex obliquely truncate, club elongate; epistoma apex truncate; labrum about 4×wider than long, weakly emarginate along apical margin; both mandibles with acute basal tooth; pronotal sides weakly convergent in basal three-fourths, arcuate to apex, depressed in anterior corners, marginal stria complete along lateral and anterior margins, slightly crenulate in front, lateral submarginal stria absent, ground punctation of pronotal disk fine, uniform, densely and almost uniformly interspersed with conspicuous secondary punctures, relatively impunctate only in basomedial area; elytra with two complete epipleural striae, outer subhumeral stria absent, inner subhumeral stria complete, dorsal striae 1-5 complete, sutural stria present in apical half, elytral disk with few coarse punctures in apical fifth; prosternum weakly convex, keel weakly truncate at base, carinal striae complete, convergent between coxae, separate throughout; prosternal lobe about two-thirds keel length, apical margin bluntly rounded, marginal stria weak, obsolete at sides; mesoventrite weakly sinuate, marginal stria complete, mesometaventral stria arched forward at middle, weakly crenulate, detached at sides; lateral metaventral stria extending from near mesocoxa posterolaterad toward middle of metacoxa, outer lateral metaventral stria absent, metaventral disk impunctate at middle; abdominal ventrite 1 with single, complete lateral stria, middle portion of disk impunctate; protibia 4 dentate, rather deeply emarginate between middle pair of denticles, outer margin serrulate between; mesotibia with two weak marginal spines; outer metatibial margin smooth; propygidium without basal stria, discal punctures ocellate, large at middle, separated by less than their diameters, smaller and sparser to sides and apex; propygidial gland openings evident about one-third from anterior margin and one-fourth from lateral margins; pygidium with ground punctation fine and rather dense, secondary punctures interspersed throughout, becoming smaller toward apex. Male: not known.

**Map 6.**
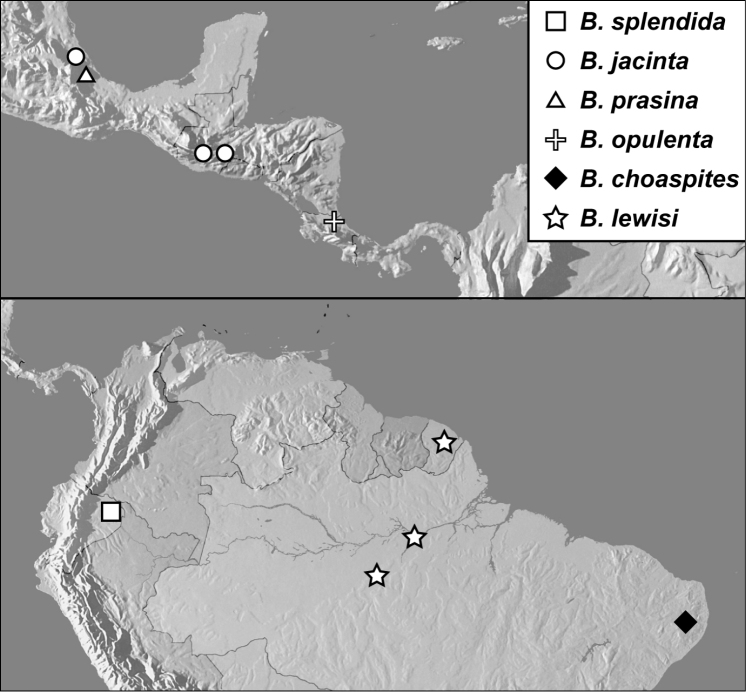
*Baconia godmani* group records.

###### Remarks.

This species is quite distinct in its complete elytral striae 1–5 (as well as the inner subhumeral), more or less uniformly punctate pronotum (Fig. 18E), and weakly depressed, strongly punctate frons and epistoma (Fig. 18F). The contrast between the greenish-blue head and pronotum and the deep blue elytra is particularly strong in this species.

###### Etymology.

This species is named for its splendid metallic coloration.

##### Baconia jacinta sp. n.

http://zoobank.org/76A52EC9-C39B-443D-918F-11B28ADA0818

http://species-id.net/wiki/Baconia_jacinta

Figs 18G–H 20 Map 6

###### Type locality.

**GUATEMALA:** Sacatepequez [14.61°N, 90.72°W].

###### Type material.

**Holotype male**: “Finca San Rafael, Sacatepequez, VII:1:48 GUAT. Elev. 6900 ft.” / “CNHM Guatemala Zool.Exped.(1948) R.D.Mitchell let.” / “under bark” / “FMNH-INS 0000 069 301” (FMNH). **Paratypes** (2): **GUATEMALA: Zacapa**: Santa Clara, in interior valley of Sierra de las Minas (N. of Cabañas), 5500 ft, 9.viii.1948, under bark, R. Mitchell (FMNH, MSCC).

###### Other material.

1: **MEXICO: Veracruz**: 15 mi W Tlapacoyan, 28.ii.1972, F. Parker & D. Miller (CHSM).

###### Diagnostic description.

**Figure 20.**
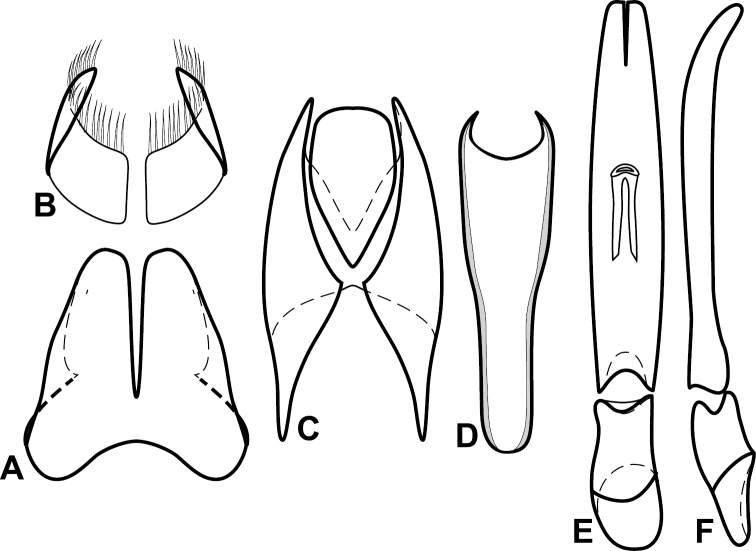
Male genitalia of *Baconia jacinta*. **A** T8 **B** S8 **C** T9 & T10 **D** S9 **E** Aedeagus, dorsal view **F** Aedeagus, lateral view.

Length: 2.3–2.4mm, width: 1.9–2.1mm; body elongate oval, moderately depressed, glabrous; head and pronotum metallic blue, usually faintly greener than deep blue metallic elytra and pygidia, venter rufo-brunneus with faint metallic tinge; frons elevated over antennal bases, depressed along antero-posterior midline, ground punctation rather conspicuous, with few secondary punctures within frontal depression, frontal stria present along inner margin of eye, curving mediad, but usually interrupted above antennal bases, at middle, or both, supraorbital stria absent; antennal scape short, club rounded; epistoma truncate to weakly emarginate; labrum about 3×wider than long, weakly emarginate along apical margin; both mandibles with acute basal tooth; pronotal sides weakly convergent in basal half, arcuate to apex, depressed in anterior corners, marginal stria complete along lateral and anterior margins, slightly crenulate in front, lateral submarginal stria absent, ground punctation of pronotal disk fine, inconspicuous across middle, sparsely interspersed with small secondary punctures toward sides; elytra with two complete epipleural striae, outer subhumeral stria absent, inner subhumeral stria present in basal fifth, dorsal striae 1–3 usually complete, 3^rd^ stria may be abbreviated apically, 4^th^ stria present in basal half only, slightly abbreviated from apex, 5^th^ stria absent, sutural stria present in up to apical half, may be very weak, mostly obsolete, elytral disk with few coarse punctures in apical fifth; prosternum moderately broad, weakly convex, keel weakly emarginate at base, carinal striae complete, divergent anterad and posterad, separate throughout; prosternal lobe about one-half keel length, apical margin bluntly rounded, marginal stria weak, fragmented; mesoventrite weakly produced, marginal stria complete, mesometaventral stria absent; lateral metaventral stria extending from near mesocoxa posterolaterad toward outer third of metacoxa, sinuate apically, outer lateral metaventral stria absent, metaventral disk impunctate at middle; abdominal ventrite 1 with lateral stria abbreviated, middle portion of disk impunctate; protibia 4-5 dentate, basal denticles weak, outer margin serrulate between teeth; mesotibia with one marginal spine and short series of weak submarginal spines toward base; outer metatibial margin smooth; propygidium without basal stria, but with several small discal punctures subserially arranged near basal margin, discal punctures otherwise small, ocellate, irregularly separated by 1–3× their diameters, sparser in apical half; propygidial gland openings evident about one-third from anterior and lateral margins; pygidium with ground punctation conspicuous, secondary punctures very small and restricted to near basal margin. Male genitalia (Fig. 20): T8 slightly longer than broad, sides weakly rounded, convergent to apex, basal emargination shallowly rounded, basal rim weakly explanate, apical emargination deep, narrow, ventrolateral apodemes separated by about three-fourths maximum T8 width, extending beneath to about one-third from base; S8 very short, halves separate, with dense setal fringe along entire apical margin, apical guides moderately well developed, similar in length throughout; T9 with basal apodemes moderately thin, short, about one-fourth total T9 length, apices long, gradually narrowed, ventrolateral apodemes fused for about one-fourth total T9 length beneath; S9 wide throughout, slightly narrowed in basal half, sides otherwise subparallel, lateral margins more strongly sclerotized, apical margin broadly emarginate; tegmen widest just basad midpoint, narrowed to base and apex, in lateral aspect slightly thickened in apical half, curved ventrad near apex; median lobe and basal piece each about one-third tegmen length.

###### Remarks.

This species is moderately distinctive externally, with its 4^th^ stria abbreviated from apex, 5^th^ absent (Fig. 18G), mesometaventral stria absent, pygidium with very few secondary punctures (Fig. 18H), no basal propygidial stria, relatively few lateral pronotal punctures, and hints of metallic ventrally. In addition it has highly distinctive male genitalia, with a very short, strongly fringed 8^th^ sternite, and a ventral fusion of the halves of the 9^th^ tergite. The specimen from Veracruz, Mexico is excluded from the types series as it shows a much more strongly impressed elytral sutural stria.

###### Etymology.

This species’ name refers to its blue coloration, ‘jacinta’ being Spanish for hyacinth.

##### Baconia prasina sp. n.

http://zoobank.org/070EB3AC-8F4E-4C45-B2B5-FD0CD50F38F7

http://species-id.net/wiki/Baconia_prasina

Figs 21A–C Map 6

###### Type locality.

**MEXICO: Veracruz:** Motzorongo [18.64°N, 96.73°W].

###### Type material.

**Holotype female**: “Motzorongo. 6” / “[handwritten, mostly unreadable] Schmidt!” / “MEXICO coll. J.Flohr” / “Caterino/Tishechkin Exosternini Voucher EXO-00488” (ZMHB).

###### Diagnostic description.

Length: 2.2mm, width: 1.9mm; body slightly elongate oval, rather strongly convex, glabrous; head, pronotum and elytra dark metallic green, pygidia only faintly metallic, venter rufo-piceous; frons elevated over antennal bases and along sides of frontal stria, slightly depressed behind, ground punctation rather conspicuous, with few secondary punctures on epistoma and within frontal depression, frontal stria present along inner margin of eye, bent mediad, complete, subangulate mediad; epistoma weakly convex along apical margin, truncate; labrum about 3×wider than long, transversely subcarinate, weakly bisinuate along apical margin; antennal scape short; pronotal sides evenly convergent in basal two-thirds, abruptly arcuate to apex, weakly depressed in anterior corners, marginal stria complete along lateral and anterior margins, slightly crenulate in front, lateral submarginal stria absent, ground punctation of pronotal disk conspicuous, increasingly interspersed with small secondary punctures in lateral thirds, punctures forming a more or less continuous submarginal series; elytra with three complete epipleural striae, outer subhumeral stria absent, inner subhumeral stria present in basal fifth and as faint median fragment, dorsal striae 1-3 usually complete, 3^rd^ stria faintly abbreviated apically, 4^th^ stria present in basal half, arched to connect to base of complete sutural stria, 5^th^ stria absent, elytral disk with few coarse punctures in apical sixth; prosternum convex, keel emarginate at base, carinal striae complete, divergent anterad and posterad, separate throughout; prosternal lobe about two-thirds keel length, apical margin bluntly rounded, marginal stria well impressed at middle, obsolete at sides; mesoventrite distinctly produced, marginal stria complete, mesometaventral stria arched forward, narrowly interrupted at middle, continuous at sides with lateral metaventral stria, extending obliquely posterolaterad toward inner third of metacoxa, outer lateral metaventral stria present, parallel to basal half of inner stria, metaventral disk impunctate at middle; abdominal ventrite 1 with single, complete lateral stria, middle portion of disk impunctate; protibia 4 dentate, basal pair of denticles very close together, outer margin serrulate between teeth; mesotibia with one marginal spine; outer metatibial margin smooth; propygidium without basal stria, discal punctures ocellate, very irregularly separated by 1-3× their diameters, smaller and sparser apically and laterally; propygidial gland openings evident about one-third from anterior and one-fourth from lateral margins; pygidium with ground punctation conspicuous, secondary punctures rather coarse and dense in basal half, smaller and sparser apically. Male: not known.

**Figure 21.**
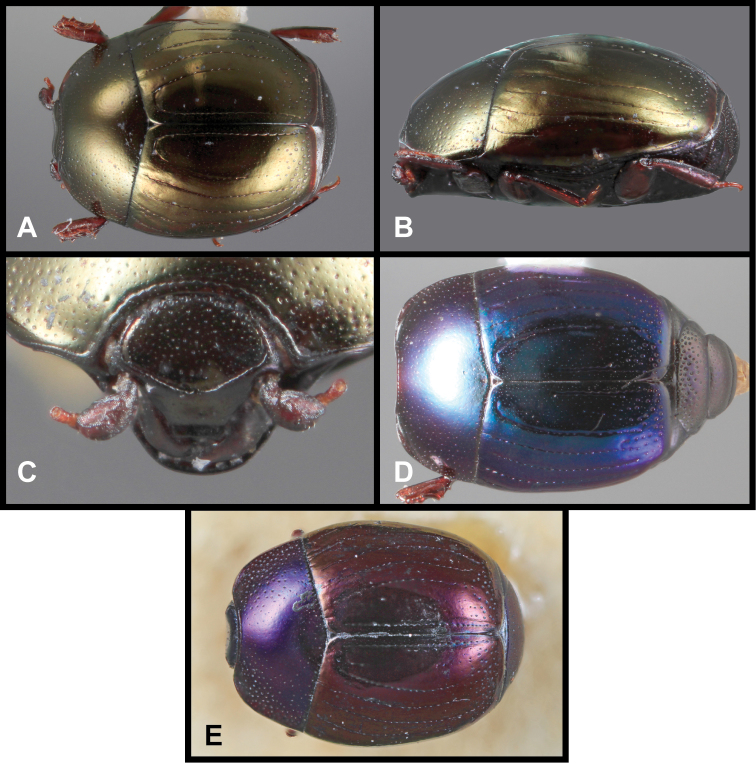
*Baconia godmani* group. **A** Dorsal habitus of *Baconia prasina* **B** Lateral habitus of *Baconia prasina* **C **Frons of *Baconia prasina* **D** Dorsal habitus of *Baconia opulenta* **E** Dorsal habitus of lectotype of *Baconia illustris*.

###### Remarks.

This is a highly distinctive species, in its relatively convex body form and dull green coloration (Fig. 21A), complete frontal stria (Fig. 21C), and basally connected 4^th^ and sutural elytral striae.

###### Etymology.

This species’ name refers to its distinctly green coloration.

##### Baconia opulenta sp. n.

http://zoobank.org/C384A7DB-0D86-43E7-82AD-4474A86CB3CA

http://species-id.net/wiki/Baconia_opulenta

Fig. 21D Map 6

###### Type locality.

COSTA RICA: Limon: Sardinas [10.7°N, 83.7°W].

###### Type material.

**Holotype female** (head mounted separately on point): “Sardinas, Barra del Colorado, Prov. Limon. COSTA RICA. 15m, 26 ABR-3MAY 1995. F. Araya, L N 291900 565900 #4639 / “INBIOCRI002170110” (INBIO).

###### Diagnostic description.

Length: 2.0mm, width: 1.7mm; body elongate oval, sides weakly rounded, moderately depressed, glabrous; head, pronotum and elytra uniformly metallic blue, with violet and greenish hints, pygidia and venter rufo-piceous; frons broad, weakly elevated above antennal bases, slightly depressed at middle, interocular margins strongly convergent dorsad, ground punctation fine, with a few secondary punctures at middle and near vertex, frontal stria fine, close to inner margin of eye, curving mediad but interrupted over antennal bases and in middle; supraorbital stria absent; epistoma weakly convex along apical and lateral margins; labrum about 3×wider than long, apically depressed, upper edge of depression arcuate and subcarinate; each mandible with acute basal tooth; antennal scape short, club asymmetrically oblong; pronotal sides weakly convergent in basal two-thirds, abruptly arcuate to apex, distinctly depressed in anterior corners, marginal stria complete along lateral and anterior margins, slightly crenulate in front, lateral submarginal stria absent, ground punctation of pronotal disk very fine, with small secondary punctures sparsely interspersed in lateral thirds; elytra with 2 complete epipleural striae, outer subhumeral stria absent, inner subhumeral stria present as short basal fragment, dorsal striae 1-4 complete, 4^th^ stria arched toward suture at base, 5^th^ stria absent, sutural stria present in apical two-thirds, elytral disk with few coarse punctures in apical fifth; prosternum moderately broad, weakly convex, keel very shallowly emarginate at base, carinal striae complete, slightly convergent at middle but separate throughout; prosternal lobe slightly less than half keel length, apical margin bluntly rounded, marginal stria more or less complete (becoming series of connected punctures at sides); mesoventrite sinuate, weakly produced at middle, marginal stria narrowly interrupted, mesometaventral stria weakly arched forward, crenulate, continuous at sides with lateral metaventral stria, which extends posterolaterad toward inner third of metacoxa, outer lateral metaventral stria absent, metaventral disk impunctate at middle; abdominal ventrite 1 with single lateral stria slightly abbreviated, middle portion of disk impunctate; protibia 4 dentate, basal denticle weak, outer margin only faintly serrulate; mesotibia with two weak marginal spines; outer metatibial margin smooth; propygidium without basal stria, ground punctation very fine, discal punctures large and rather deep mediobasally, separated by slightly less than their diameters, smaller and sparser to sides and apical margin; propygidial gland openings conspicuous nearly one-half from anterior and one-fourth from lateral margins; pygidium with ground punctation fine, sparse, secondary punctures small, uniformly separated by about 4× their diameters. Male: not known.

###### Remarks.

This species’ rather depressed body form, in combination with non-metallic pygidium, elytral striae 1-4 complete with the 4^th^ arched basally (Fig. 21D), and the triply interrupted frontal stria will distinguish it from all others.

###### Etymology.

This species’ name refers to its rich, deep blue coloration.

##### Baconia illustris

(Lewis, 1900)

http://species-id.net/wiki/Baconia_illustris

Fig. 21E

Phelister illustris Lewis, 1900: 226; *Baconia illustris*: Mazur 1984: 280.

###### Type locality.

BRAZIL [exact locality uncertain].

###### Type material.

**Lectotype**, sex undetermined, here designated (BMNH): “Brazil” / “Barton” / “*Phelister illustris* Lewis Type” / “G.Lewis Coll. B.M.1926-369” / “LECTOTYPE *Phelister illustris* Lewis, M.S.Caterino & A.K.Tishechkin des. 2010”. This species was described from an unspecified number of specimens, and the lectotype designation fixes primary type status on the only known original specimen.

###### Diagnostic description.

Length: [not measured, ~2.5mm], width: [not measured, ~1.5mm]; body slightly elongate oval, rather strongly convex, glabrous; head slightly greenish-blue, pronotum and pygidia metallic violet-blue, elytra strongly violet, venter rufo-brunneus, but with distinct metallic tinge; frons weakly elevated over antennal bases, slightly depressed in middle, with few secondary punctures within frontal depression, frontal stria present along inner margin of eye, bent mediad, obsolete across middle; labrum about 3×wider than long, apical margin straight; antennal scape short; pronotal sides weakly rounded, convergent, weakly depressed in anterior corners, marginal stria complete along lateral and anterior margins, slightly crenulate in front, lateral submarginal stria absent, ground punctation of pronotal disk conspicuous, increasingly interspersed with coarse secondary punctures in lateral thirds; elytra with two complete epipleural striae, outer subhumeral stria absent, inner subhumeral stria present only as short basal fragment, dorsal striae 1-3 complete, 4^th^ stria nearly complete, becoming fragmented/obsolete in apical fourth, 5^th^ stria absent, sutural stria complete, elytral disk with coarse punctures in apical fourth; prosternum convex, keel emarginate at base, carinal striae complete, divergent anterad and posterad, separate throughout; prosternal lobe about one-half keel length, apical margin bluntly rounded, marginal stria well impressed at middle, obsolete at sides; mesoventrite distinctly produced, marginal stria complete, mesometaventral stria absent, lateral metaventral stria extending obliquely posterolaterad toward middle of metacoxa, outer lateral metaventral stria present as short basal striole, metaventral disk impunctate at middle; abdominal ventrite 1 with single lateral stria slightly abbreviated, curved mediad at apex, middle portion of disk impunctate; protibia tridentate, with 2 additional minute basal denticles, outer margin serrulate between teeth; mesotibia with one marginal spine; outer metatibial margin smooth; propygidium without basal stria, discal punctures rather deep, separated by their diameters in basal half, much smaller and sparser in apical half; pygidium with ground punctation conspicuous, secondary punctures few and restricted to basal margin. Male genitalia: not known.

###### Remarks.

The color pattern, strongly rounded and convex body form (Fig. 21E), and complete sutural stria are adequate to recognize this species. This species’ type locality is too vague to pinpoint.

##### Baconia choaspites

Lewis, 1901

http://species-id.net/wiki/Baconia_choaspites

Figs 22A–B Map 6

Baconia choaspites Lewis, 1901: 372.

###### Type locality.

BRAZIL: Pernambuco: Serra de Communaty [exact locality unknown].

###### Type material.

**Lectotype**, sex undetermined, here designated (BMNH): “Serra de Communaty (Pernambuco) Gounelle 1.2.3.1893” / “*Baconia choaspites* Lewis Type” / “G.Lewis Coll. B.M.1926-369” / “LECTOTYPE *Baconia choaspites* Lewis, M.S.Caterino & A.K.Tishechkin des. 2010”. This species was described from an unspecified number of specimens, and the lectotype designation fixes primary type status on the only known original specimen.

###### Diagnostic description.

Length: [not measured, ~2.2mm], width: [not measured, ~1.3mm]; body elongate oval, moderately strongly flattened, glabrous; head, pronotum and pygidia slightly greenish-blue, elytra metallic blue, venter rufo-piceous; frons rather broad, very weakly depressed in middle, with few secondary medial punctures, frontal stria weak, present along inner margin of eye, obsolete across front; labrum about 3×wider than long, very deeply emarginate apically; antennal scape short, club rounded; pronotal sides weakly but evenly arcuate, convergent to front, depressed in anterior corners, marginal stria complete along lateral and anterior margins, slightly crenulate in front, lateral submarginal stria absent, ground punctation of pronotal disk conspicuous, increasingly interspersed with coarse secondary punctures in lateral thirds; elytra with two complete epipleural striae, outer subhumeral stria absent, inner subhumeral stria present only as short basal fragment, dorsal striae 1–2 complete, 1^st^ stria arched mediad at base, 3^rd^ stria more or less complete but becoming fragmented toward apex, 4^th^ stria slightly shorter than third, similarly fragmented apically, arched toward suture at base, 5^th^ and sutural striae absent, elytral disk with coarse punctures in apical fourth; prosternum moderately broad, weakly convex, keel very shallowly emarginate at base, carinal striae complete, subparallel, separate throughout; prosternal lobe about one-half keel length, apical margin bluntly rounded, marginal stria well impressed at middle, obsolete at sides; mesoventrite faintly produced, marginal stria narrowly interrupted at middle, mesometaventral stria arched strongly forward, lateral metaventral stria slightly sinuate, extending obliquely posterolaterad toward outer third of metacoxa, abbreviated apically, outer lateral metaventral stria short, fragmented, metaventral disk impunctate at middle; abdominal ventrite 1 with single lateral stria slightly abbreviated, curved mediad at apex, middle portion of disk impunctate; protibia tridentate, with 2 additional minute basal denticles; mesotibia with one marginal spine; outer metatibial margin smooth; propygidium without basal stria, discal punctures rather deep, somewhat irregularly separated by about their diameters throughout; pygidium with ground punctation conspicuous, secondary punctures small and sparse, mainly limited to basal half. Male genitalia: not known.

**Figure 22.**
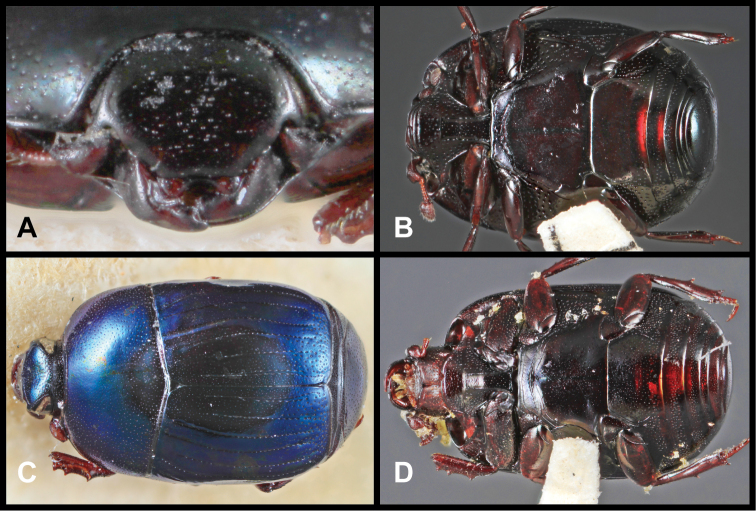
*Baconia godmani* group. **A** Frons of lectotype of *Baconia choaspites* **B** Ventral habitus of lectotype of *Baconia choaspites* **C** Dorsal habitus of lectotype of *Baconia lewisi* **D** Ventral habitus of lectotype of *Baconia lewisi*.

###### Remarks.

This species is placed in the *Baconia godmani* group due to overall similarity, but the deeply emarginate labrum (Fig. 22A) is unusual in the group. This feature, as well as the widely separated, parallel prosternal carinal striae (Fig. 22B) will help identify it. The basal curvature of the 1^st^ dorsal elytral stria is unique, but it seems somewhat questionable that it represents more than an individual aberration. Confirmation of these characters would help better define this apparently distinctive species. Discovery of a male specimen to allow study of male genitalia would also help confirm its placement in the *Baconia godmani* group.

##### Baconia lewisi

Mazur, 1984

http://species-id.net/wiki/Baconia_lewisi

Figs 22C–D 23 Map 6

Epierus festivus Lewis, 1898: 171; *Phelister festivus*: Lewis 1900: 226; *Baconia festiva* (Lewis, 1898: 171) Mazur 1984: 280; not *Baconia festiva*Lewis 1891: 389.
Baconia lewisi : Mazur, 1984: 280, replacement name.

###### Type locality.

BRAZIL: Pará: Santarém [2.44°S, 54.70°W].

###### Type material.

**Lectotype**, sex undetermined, here designated (BMNH): “Santarem” / “H.H. Smith 1898” / “*Phelister (Epierus) festivus* Lewis Type” / “G.Lewis Coll. B.M.1926-369” / “ LECTOTYPE *Phelister festivus* Lewis 1898, M.S.Caterino & A.K.Tishechkin des. 2010”. This species was described from an unspecified number of specimens, and the lectotype designation fixes primary type status on the only known original specimen.

###### Other material.

**BRAZIL**: 1: **Pará**: Jacareacanga, xii.1968, M. Alvarenga (UFPR). **FRENCH GUIANA**: 1:Montagne des Chevaux, 4°43'N, 52°24'W, 1.viii.2009, FIT, SEAG (CHND), 1:19.ix.2009, FIT, SEAG (MSCC), 1:22.xii.2009, FIT, SEAG (CHND), 1:9.v.2009, FIT, SEAG (FMNH), 1: 31.v.2009, FIT, SEAG (AKTC).

###### Diagnostic description.

**Figure 23.**
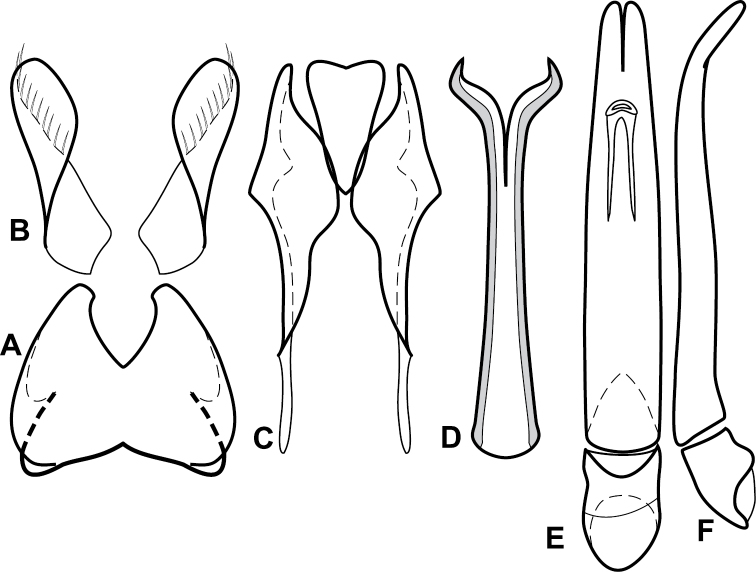
Male genitalia of *Baconia lewisi*. **A** T8 **B** S8 **C** T9 & T10 **D** S9 **E** Aedeagus, dorsal view **F **Aedeagus, lateral view.

Length: 2.5–2.6mm, width: 2.0–2.1mm; body elongate oval, sides subparallel, only weakly depressed, glabrous; dorsum rich metallic blue, pronotum rarely subtly more greenish, venter piceous; frons weakly elevated over antennal bases, depressed at middle, ground punctation rather coarse, with numerous larger punctures on epistoma, at middle of frontal disk and toward vertex, frontal stria complete, fine across front, subangulate at middle, supraorbital stria absent; antennal scape short, club asymmetrically oblong; epistoma weakly emarginate apically; labrum about 3×wider than long, distinctly bisinuate along apical margin, projecting at middle; both mandibles with acute basal tooth; pronotal sides weakly arcuate to apex, marginal stria complete along lateral and anterior margins, slightly crenulate, removed from margin behind head, lateral submarginal stria absent, pronotal disk not impressed in anterior corners, ground punctation of disk rather conspicuous throughout, with quite small secondary punctures increasing in size and density toward sides; elytra with two complete epipleural striae, outer subhumeral stria absent, inner subhumeral stria may be complete, but usually interrupted in middle and slightly abbreviated at apex, dorsal striae 1–4 complete, 5^th^ stria slightly abbreviated from base, sutural stria complete or slightly abbreviated basally, elytral disk with coarse punctures in apical fourth; prosternum rather narrow, flat at base, more convex anterad, keel very weakly emarginate at base, carinal striae slightly shortened anteriorly, may end freely or unite in anterior arch, usually free basally; prosternal lobe about one-half keel length, apical margin subtruncate, marginal stria obsolete at sides; mesoventrite very weakly produced at middle, with complete marginal and submarginal striae; mesometaventral stria arched forward at middle, crenulate, and with secondary mesometaventral stria behind it, interrupted at middle; inner lateral metaventral stria extending from end of secondary mesometaventral stria obliquely posterolaterad toward middle of metacoxa, slightly abbreviated apically, outer lateral metaventral stria parallel to inner stria for most or all of its length; metaventral disk with ground punctation distinct, but lacking secondary punctures; abdominal ventrite 1 with complete inner lateral stria and posterior fragments of outer stria, middle portion of disk with very small but distinct punctures decreasing in density anteromediad; protibia rather narrow, elongate, with 3 well developed marginal teeth, outer margin serrulate between teeth; meso- and metatibiae each with distinct marginal spine and few weak basal submarginal denticles; propygidium with complete transverse basal stria, discal punctures rather small, separated by about their diameters throughout; propygidial gland openings evident behind ends of transverse stria, about one-sixth from each lateral margin; pygidium with ground punctation dense throughout, with only slightly larger secondary punctures interspersed in basal half. Male genitalia (Fig. 23): T8 slightly shorter than broad, sides weakly widened in basal one-fourth, convergent to apex, basal emargination shallow, weakly acute at middle, apical emargination deep, elongate, with ventrolateral apodemes separated by about two-thirds maximum T8 width, extending about one-half distad beneath, obsolete in apical half; S8 divided, inner margins approximate at base, strongly divergent in apical three-fourths, bearing conspicuous fringe of setae in apical one-half, outer margins subparallel to weakly divergent, apical guides well developed in apical half, broadly rounded; T9 with basal apodemes thin, about one-half total length, T9 apices narrowly rounded, glabrous, ventrolateral apodemes weakly projecting beneath; S9 weakly widened at base, head broad, subangulate to apicolateral points, desclerotized along midline, with narrow apicomedial division; tegmen with sides subparallel in basal two-thirds, weakly narrowed to apex, dorsobasal edge weakly arcuate, tegmen in lateral aspect more or less straight in basal three-fourths, abruptly curved ventrad at apex; median lobe about one-fourth tegmen length; basal piece about one-fifth tegmen length.

###### Remarks.

The elongate, strongly convex body shape (Fig. 22C), and unique arrangment of extra meso- and metaventral striae (Fig. 22D) immediately identify this distinctive species.

#### *Baconia salobrus* group

**Figure 24.**
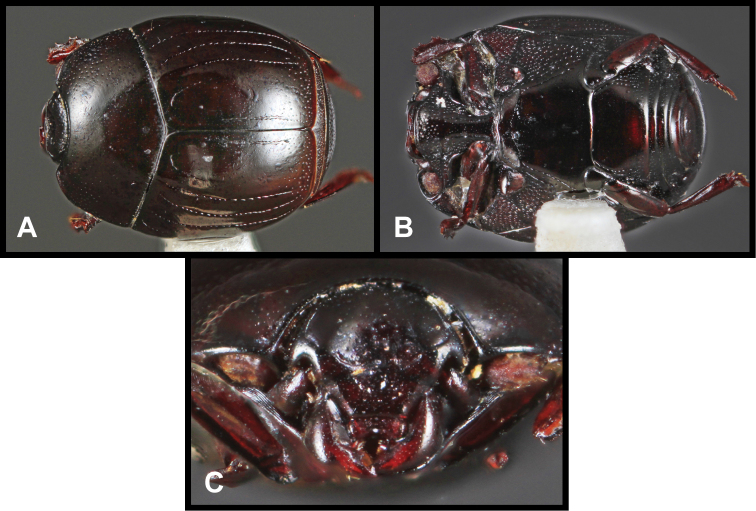
*Baconia salobrus*. **A** Dorsal habitus **B** Ventral habitus **C** Frons.

The *Baconia salobrus* group comprises six mostly convex, black species that otherwise bear considerable similarity to species in the *Baconia godmani* group, to the extent they generally have a weakly depressed, variably punctate frons (Fig. 24C), and weakly produced anterior mesoventral margin. The black body color is more distinctive than it might appear, in that most other non-metallic species of *Baconia* are lighter in color, usually distinctly rufopiceous if dark. When the 4^th^ dorsal stria is present (in about half the species), it forms a basal arch to the elytral suture (Fig. 24A). Most of the species have some form of labral or epistomal modification, in sculpturing principally, and this may help substantiate a close relationship among them.

##### Baconia salobrus

(Marseul, 1887)

http://species-id.net/wiki/Baconia_salobrus

Figs 24 25 Map 7

Phelister salobrus Marseul, 1887: cxlviii; *Baconia salobrus*: Dégallier et al. 2012: 36.

###### Type locality.

BRAZIL: Bahia: Salobro [exact locality uncertain].

###### Type material.

**Lectotype**, sex undetermined, here designated (BMNH): “Salobro prov de Bahia, Bresil, E.Gounelle 6.7.1885” / “Marseul’s Type” / “*Phelister salobrus* n.sp.” / “G.Lewis Coll. B.M.1926-369” / “Cotype or Type of *Phel salobrus* Mars. RLW ‘54” / “LECTOTYPE *Phelister salobrus* Marseul, 1887, M.S.Caterino & A.K.Tishechkin des. 2010”. **Paralectotypes** (4): same data as type(BMNH, MNHN). This species was described from an unspecified number of specimens, and the lectotype designation fixes primary type status on one of the known original specimens.

###### Other material.

**ARGENTINA**: 1: **Jujuy**: P. N. Calilegua, Estaca El Cero, 900 m, 18–28.xii.1987, forest malaise-FIT, S. & J. Peck (CHSM). **BELIZE**, 1: **Cayo**: Las Cuevas Res. Sta., 16°44.00'N, 88°58.24'W, 550 m, 23.v.2000, FIT, M. Caterino; 1: v.1997, D. Inward; 1: **Orange Walk**: Rio Bravo Cons. Area, La Milpa Field Station, 15–25.v.1997, FIT, C.E. Carlton (CHPWK). **BOLIVIA** 1: **Santa Cruz**:Hotel Flora y Fauna, 4–5 km SSE Buena Vista, 17°29.9'S, 63°39.1'W, 440 m, 24–31.xii.2003, FIT, S. & J. Peck (AKTC); 1:3–9.xi.2002, FIT, R. Leschen (AKTC). **BRAZIL**: 3: **Bahia**: 1885, E. Gounelle (MNHN); 1: **Mato Grosso do Sul**: cerradão fragment nr. Selviria, 20.3354°S, 51.4095°W, 30.xi–3.xii.2011, FIT, M. Caterino & A. Tishechkin, DNA Extract MSC-2241, EXO-00856 (MSCC). **FRENCH GUIANA**: 1:Belvèdére de Saül, 3°1'22"N, 53°12'34"W, 31.xi.2010, FIT, SEAG. **PANAMA**:1: **Colón**: P. N. San Lorenzo, Achiote, Cafetal A Dist., 09°12'N, 79°58'W, 0 m, 7-21.v.2007, FIT, A. Mercado (GBFM).

###### Diagnostic description.

**Figure 25.**
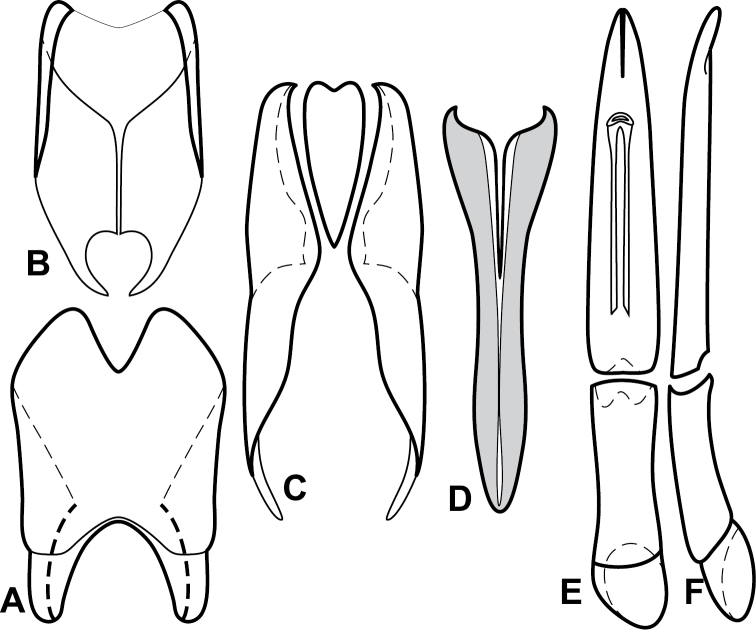
Male genitalia of *Baconia salobrus*. **A** T8 **B** S8 **C** T9 & T10 **D** S9 **E** Aedeagus, dorsal view **F** Aedeagus, lateral view.

Length: 2.2–2.4mm, width: 1.9–2.2mm; body elongate oval, weakly depressed, glabrous; color piceous, shining; frons depressed along midline, with few coarse punctures in depression, interocular margins convergent dorsad, frontal stria interrupted above antennal bases and at middle; supraorbital stria absent; antennal scape short, club slightly oblong; epistoma straight across apex; labrum about 3×wider than long, broadly, shallowly emarginate; mandibles each with small basal tooth; pronotal sides rather evenly narrowed in basal two-thirds, thence abruptly narrowed to apices, lateral marginal striae continuous around sides and front, submarginal stria absent, pronotal disk weakly depressed in anterior corners, disk largely impunctate at middle, with sparse coarse punctures near sides; elytra with two complete and a third partial epipleural stria, outer subhumeral stria absent, inner subhumeral stria fragmentarily present at base, dorsal striae 1-3 more or less complete, 4^th^ stria present in basal third, arched mediad at base toward suture, rarely meeting sutural stria, 5^th^ stria absent, sutural stria present in apical half to two-thirds, rarely complete, elytral disk sparsely punctate in apical fourth; prosternal keel moderately broad, weakly convex, very shallowly emarginate at base, with more or less complete, subparallel carinal striae; prosternal lobe about two-thirds keel length, apical margin rather narrowly rounded, marginal stria present only at middle; mesoventrite weakly produced at middle, marginal stria narrowly interrupted at middle, rarely complete; mesometaventral stria arched forward, continued laterally by inner lateral metaventral stria, which is rather widely separated at base from mesocoxa, curving posterolaterad toward middle of metacoxa, outer lateral metaventral stria parallel to basal half or slightly more of inner metaventral stria; metaventral and abdominal disks impunctate at middle; abdominal ventrite 1 with complete inner lateral striae, outer lateral stria present as short fragment behind metacoxa; protibiae 4-dentate, with basal two spines close together, outer margin finely serrulate; mesotibia with one marginal spine; outer metatibial margin smooth; propygidium lacking basal stria, with small secondary punctures separated by about 1.5× their diameters near base, smaller and sparser toward apex; propygidial glands evident about one-fourth from anterior and lateral margins; pygidium with fine ground punctation uniformly interspersed with small secondary punctures, separated by 2× their diameters. Male genitalia (Fig. 25): T8 about one-third longer than broad, sides subparallel, basal emargination deep, narrow, basal rim well sclerotized, basal membrane attachment line conspicuous, apical emargination moderately deep, ventrolateral apodemes separated by about two-thirds maximum T8 width, extending about one-third distad beneath, narrowed to apex; S8 divided, inner margins approximate along much of midline, divergent at base and apex, apical margin with membraneous velum, without conspicuous setae, outer margins subparallel to weakly convergent, apical guides narrow, consistent in width throughout, narrowly rounded apically; T9 with basal apodemes rather thick, short, with dorsal surface extending nearly to base, T9 apices broad, finely acute at inner corner, glabrous, ventrolateral apodemes prominent but blunt beneath; S9 with sides of stem subparallel in basal half, evenly widening to apex, with deep, narrow apical emargination, very narrowly desclerotized along most of midline; tegmen widest near base, sides very weakly rounded, convergent to narrow apices, tegmen in lateral aspect more or less straight, just slightly bent ventrad at apex; median lobe simple, two-thirds tegmen length; basal piece long, nearly two-thirds tegmen length.

**Map 7.**
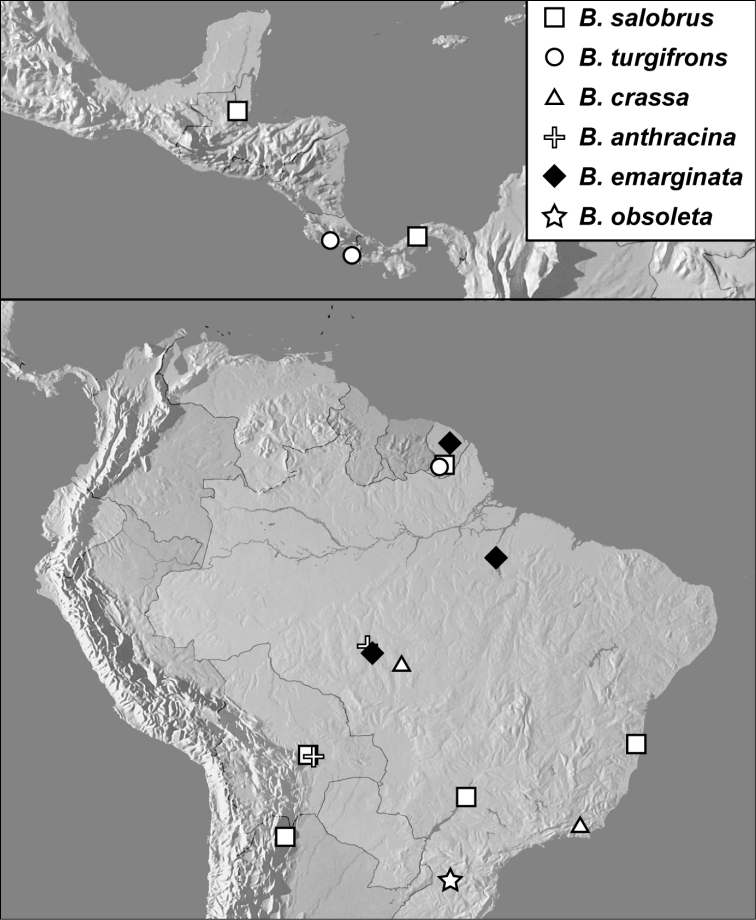
*Baconia salobrus* group records.

###### Remarks.

In this widespread species, the 4^th^ elytral stria is represented by a basal arch that does not meet the sutural stria (Fig. 24A), the marginal mesoventral and mesometaventral striae are both complete (Fig. 24B), the labrum is evenly emarginate (Fig. 24C), and the frontal stria is interrupted over both antennal bases and at the middle. These characteristics are adequate to distinguish it from other convex black *Baconia* spp. In addition, the elongate basal piece of the aedeagus is unique in the group (and nearly in the genus).

This is a difficult type locality to pinpoint, but there appears to have been a diamond mine associated with a perhaps now-extinct town of Salobro along the Pardo River, in southern Bahia, approximately 70km upriver of the coastal town of Canavieiras.

##### Baconia turgifrons sp. n.

http://zoobank.org/A3024542-153C-4408-9C36-5DF685C70D9F

http://species-id.net/wiki/Baconia_turgifrons

**Figure 26.**
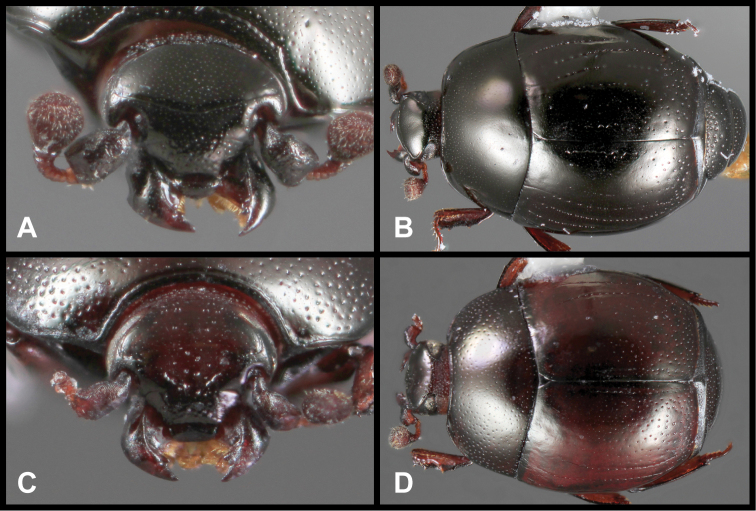
*Baconia salobrus* group. **A** Frons of *Baconia turgifrons* **B** Dorsal habitus of *Baconia turgifrons* **C** Frons of *Baconia crassa* **D** Dorsal habitus of *Baconia crassa*.

Figs 26A–B 27A–C, G, I–J Map 7

###### Type locality.

COSTA RICA: Puntarenas: Osa Peninsula [8.68°N, 83.52°W].

###### Type material.

**Holotype male**: “**COSTA RICA**: Punta Prov. Rincon de Osa, 150 m, 8°41.141'N, 83°31.117'W, 23-26-VI-2001, S. & J. Peck, 01-14, ex FIT, CR1P01 006” / “SM0563778 KUNHM-ENT” (SEMC). **Paratype** (1): **Puntarenas**: Sector Laguna Meandrica, R.B. Carara, 100 m, vi.1990, R. Zuniga (INBI).

###### Other material.

**FRENCH GUIANA**: Mont tabulaire Itoupé, 3°1.82'N, 53°6.40'W, 400m, FIT, 31.iii.2010, SEAG (MNHN).

###### Diagnostic description.

**Figure 27.**
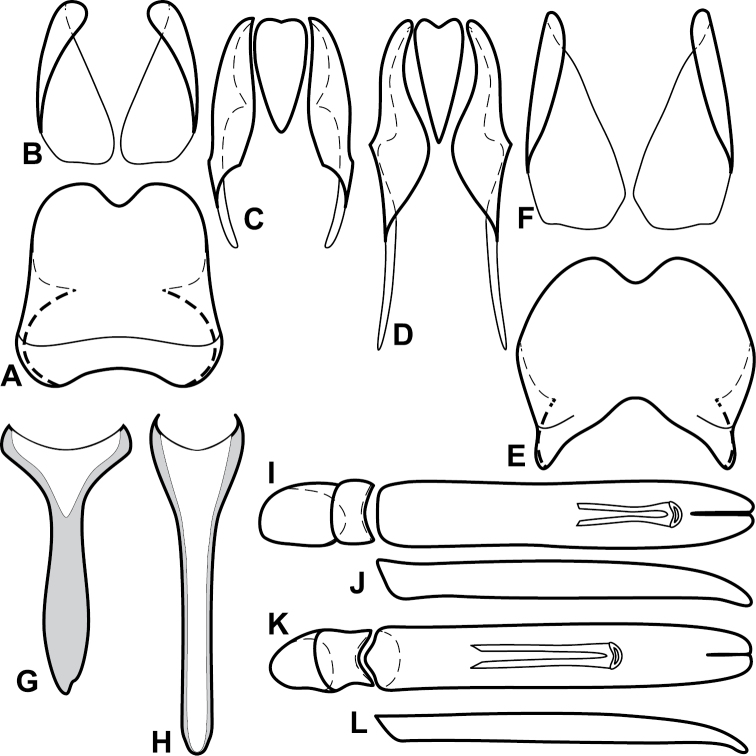
Male genitalia of *Baconia salobrus* group. **A** T8 of *Baconia turgifrons* **B** S8 of *Baconia turgifrons* **C** T9 & T10 of *Baconia turgifrons* **D** T9 & T10 of *Baconia crassa* **E** T8 of *Baconia crassa* **F** S8 of *Baconia crassa* **G** S9 of *Baconia turgifrons* **H **S9 of *Baconia crassa* **I** Aedeagus, dorsal view of *Baconia turgifrons* **J** Aedeagus, lateral view of *Baconia turgifrons* **K** Aedeagus, dorsal view of *Baconia crassa* **L** Aedeagus, lateral view of *Baconia crassa*.

Length: 1.9–2.1mm, width: 1.7–1.8mm; body elongate oval, convex, glabrous; color piceous, shining; frons very strongly convex, epistoma receding beneath and very finely, densely punctate, frontal disk with only sparse, fine ground punctation, frontal stria interrupted above antennal bases, present at middle (may rarely be complete); supraorbital stria absent; antennal scape short, thick, club oblong, distinctly widened toward apex; labrum reduced, transversely carinate, strongly narrowed to apex, subtriangular; mandibles rather short, convex dorsally, each with small basal tooth; pronotal sides rather evenly narrowed in basal three-fourths, thence abruptly narrowed to apices, lateral marginal striae continuous around sides and front, submarginal stria absent, anterior corners of pronotal disk deflexed, disk with only fine ground punctation at middle, with few coarser secondary punctures near anterolateral corners; elytra with two complete epipleural striae, outer subhumeral stria absent, inner subhumeral stria fragmentarily present at base, dorsal stria 1 scratchlike in apical half, variably abbreviated apically, 2^nd^ stria more or less complete, may be weakly abbreviated basally, 3^rd^ stria present in basal half only, 4^th^ and 5^th^ striae absent, sutural stria present in apical half to two-thirds, elytral disk with few rather coarse punctures in apical fourth; prosternal keel moderately broad, depressed across midline, shallowly emarginate at base, carinal striae convergent between coxae, complete, free; prosternal lobe short, about one-half keel length, apical margin rounded, marginal stria obsolete at sides; mesoventrite weakly produced at middle, marginal stria complete; mesometaventral stria simple, transverse or slightly angulate forward at middle, continued laterally by inner lateral metaventral stria, which is oblique, short, barely reaching middle of metaventrite, outer lateral metaventral stria slightly shorter; metaventral and abdominal disks impunctate at middle; abdominal ventrite 1 with inner lateral striae abbreviated apically, outer lateral stria absent; protibiae 4-dentate, middle pair of spines widely separated, outer margin finely serrulate; meso- and metatibiae rather broad, expanded apically, mesotibia with two marginal spines; outer metatibial margin smooth; propygidium lacking basal stria, with coarse secondary punctures separated by slightly less than their diameters, concentrated in basal two-thirds, propygidial glands inconspicuous; pygidium with fine ground punctation very sparsely interspersed with small secondary punctures, separated by 4–5× their diameters. Male genitalia (Figs 27A–C, G, I–J): T8 slightly longer than broad, sides straight, weakly convergent, basal emargination shallow, basal rim well sclerotized, explanate, basal membrane attachment line conspicuous, apex shallowly emarginate, ventrolateral apodemes projecting deeply beneath, to about midpoint of segment, separated by about one-third maximum T8 width, rapidly narrowed apically; S8 divided, inner margins approximate only at base, divergent apically, outer margins weakly rounded, convergent, apical guides widening distally, narrowly rounded at apices, apical velar membrane absent, apex lacking conspicuous setae; T9 with basal apodemes short, only about one-fourth total length, T9 apices narrowed, subacute, glabrous, ventrolateral apodemes prominent beneath; S9 with stem narrowest near midpoint, widened to base and more strongly to apex, apical emargination broadly arcuate, apex broadly desclerotized at middle; tegmen with sides uneven, narrowest just basad midpoint, weakly widened basally and apically, apices bluntly rounded, tegmen in lateral aspect more or less straight; median lobe simple, about one-third tegmen length; basal piece short, about one-fourth tegmen length.

###### Remarks.

The strongly recessed and microsculptured epistoma (Fig. 26A) and reduced labrum, in combination with the strongly convex frons is unique to this species, as is the pattern of elytral striae, with only the 2^nd^ stria complete, the 1^st^ and 3^rd^ striae abbreviated from the apex, and the 4^th^ and 5^th^ striae absent (Fig. 26B). The following species, *Baconia crassa*, is very similar, having a microsculptured epistoma, but has the frons depressed in the middle, a complete 1^st^ elytral stria, and a more nearly complete inner metaventral stria.

###### Etymology.

This species is named for its swollen frons.

##### Baconia crassa sp. n.

http://zoobank.org/C8251AD0-16D6-4258-8731-21CF3648970F

http://species-id.net/wiki/Baconia_crassa

Figs 26C–D 27D–F, H, K–L Map 7

###### Type locality.

BRAZIL: Rio de Janeiro: Nova Friburgo [22.26°S, 42.53°W].

###### Type material.

**Holotype male**: “**BRAZIL: Rio de Janeiro**, Nova Friburgo, 22°16'S, 42°32'W, piege d’interception 26-31 Oct 2009” / “Caterino/Tishechkin Exosternini Voucher EXO-00484” (UFPR). **Paratype** (1): same data as type (UFPR).

###### Other material.

1: **Mato Grosso**:Mpio. Claudia, 11°24.5'S, 55°19.5'W, 17–27.x.2010, FIT, A.F. Oliveira (CEMT).

###### Diagnostic description.

Length: 1.9–2.2mm, width: 1.7–2.0mm; body rather broadly elongate oval, convex, glabrous; faintly bicolored, with elytra rufopiceous, rest of body piceous, shining; frons elevated over antennal bases, depressed in middle, interocular margins convergent dorsad, epistoma convex and finely, densely punctate, frontal disk with few coarse punctures in median depression, frontal stria present along inner edges of eyes, largely absent between antennal bases, may be represented by few median fragments; supraorbital stria fine, rudimentary; antennal scape short, thick, club oblong, widened toward apex; labrum with upper edge weakly carinate, emarginate, lower margin narrowed, recessed between mandibles; mandibles rather short, each with small basal tooth; pronotal sides weakly arcuate in basal two-thirds, more strongly arcuate to apices, lateral marginal striae continuous around sides and front, submarginal stria absent, anterior corners of pronotal disk weakly depressed, coarser secondary punctures of pronotal disk extending across anterior half, sides, and along basal margin, relatively impunctate only in small area posteromedially; elytra with two complete epipleural striae and fragments of third (outermost), outer subhumeral stria absent, inner subhumeral stria present as short basal fragment, dorsal striae 1–2 more or less complete, 2^nd^ stria may be weakly abbreviated basally, 3^rd^ stria in basal half only, scratchlike, 4^th^ and 5^th^ striae absent, sutural stria present for short distance at middle, elytral disk with coarse punctures diminishing from apex nearly to midline; prosternal keel moderately broad, weakly convex, shallowly emarginate at base, carinal striae convergent between coxae, complete, free; prosternal lobe short, about one-half keel length, apical margin rounded, marginal stria well impressed at middle, obsolete at sides; mesoventrite weakly produced at middle, marginal stria complete; mesometaventral stria weakly arched forward at middle, crenulate, continued laterally by inner lateral metaventral stria toward middle of metacoxa, slightly abbreviated at apex, outer lateral metaventral stria subparallel, about half as long; metaventral and abdominal disks impunctate at middle; abdominal ventrite 1 with inner lateral stria abbreviated apically, outer lateral stria absent; protibiae 4-dentate, middle pair of spines more widely separated, outer margin finely serrulate; meso- and metatibiae moderately expanded apically, mesotibia with one marginal spine; outer metatibial margin smooth; propygidium lacking basal stria, with coarse secondary punctures separated by slightly less than their diameters near basal margin, much sparser posterad, propygidial glands inconspicuous; pygidium with fine ground punctation very sparsely interspersed with small secondary punctures mainly in basal half. Male genitalia (Figs 27D–F, H, K–L): T8 shorter than broad, sides broadly arcuate, basal emargination broad, moderately deep, basal rim well sclerotized, explanate, basal membrane attachment line visible at sides, apex shallowly emarginate, ventrolateral apodemes projecting weakly beneath, about one-third from base, separated by about two-thirds maximum T8 width, rapidly narrowed apically; S8 divided, inner margins approximate only at base, divergent apically, outer margins weakly rounded, convergent, apical guides slightly widened distally, narrowly rounded at apices, apical velar membrane absent, apex lacking conspicuous setae; T9 with basal apodemes rather thin and elongate, just over one-third total length, T9 apices narrowly rounded, glabrous, ventrolateral apodemes weakly projecting beneath; S9 with stem narrowest near midpoint, faintly widened to base, more strongly to apex, apical emargination arcuate, apex broadly desclerotized at middle, intermediate in sclerotization along entire midline; tegmen with sides subparallel, weakly narrowed to blunt apices, tegmen in lateral aspect more or less straight, weakly curved ventrad at tip; median lobe simple, about one-half tegmen length; basal piece short, about one-fourth tegmen length.

###### Remarks.

In addition to the diagnostic characters pertaining to the frons of this species (Fig. 26C), given under *Baconia turgifrons* above, the more widespread secondary punctures of the pronotum (Fig. 26D) will distinguish *Baconia crassa*. The type specimens are somewhat more rufopiceous than strictly piceous in color. The single individual from Mato Grosso exhibits a lower density of epistomal punctation, and is piceous, and so is excluded from type series. More material might support separation, but in any case it is clearly closely related.

###### Etymology.

This species’ name refers to its wide-bodied shape, *crassa* meaning literally fat or plump.

##### Baconia anthracina sp. n.

http://zoobank.org/5B6E690E-EAFA-47FD-B089-4EB26654B12E

http://species-id.net/wiki/Baconia_anthracina

Figs 1A, E 2C 28A–B 29A–F Map 7

###### Type locality.

BOLIVIA: Santa Cruz: Flora y Fauna Hotel [17.49°S, 63.55°W].

###### Type material.

**Holotype male**: “**BOLIVIA**: Santa Cruz Dep. 3.7 km SSE Buena Vista, Flora y Fauna Hotel, 17°29.9'S, 63°33.2'W, 400–440m. F.I.T. 3-9 Nov 2002. R. Leschen # 054” / “Caterino/Tishechkin Exosternini Voucher EXO-00514” (SEMC). **Paratypes** (26): **BOLIVIA**: 22:Hotel Flora y Fauna, 5 km SSE Buena Vista, 17°29.9'S, 63°39.1'W, 14–24.xii.2003, FIT, S. & J. Peck (CMNC, FMNH, MSCC, AKTC), 2:24–31.xii.2003, FIT, S. & J. Peck (CMNC); 2: **Santa Cruz**:Amboro National Park, Los Volcanes, 18°06'S, 63°36'W, 1000 m, 20.xi-12.xii.2004, FIT, H. Mendel & M. Barclay (BMNH).

###### Other material.

1: **BRAZIL**: **Mato Grosso**:Mpio. Cotriguaçu, Fazenda São Nicolau, Matinha, 9°50.3'S, 58°15.05'W, 3.iv.2009, FIT, F. Vaz-de-Mello (CEMT).

###### Diagnostic description.

Length: 1.9–2.1mm, width: 1.7–2.0mm; body rather broadly elongate oval, convex, glabrous; piceous, shining; frons elevated over antennal bases, depressed in middle, interocular margins convergent dorsad, frontal disk with few coarse punctures in median depression, epistoma with fairly descrete median fovea, otherwise impunctate, frontal stria present along inner edges of eyes, largely absent between antennal bases, may be represented by few median fragments; supraorbital stria absent; antennal scape short, club broadly rounded, slightly asymmetrical; labrum with upper edge weakly carinate, emarginate, distal margin narrowed, recessed between mandibles; mandibles rather short, each with small basal tooth; pronotal sides evenly narrowed in basal two-thirds, more strongly arcuate to apices, lateral marginal striae continuous around sides and front, submarginal stria absent, anterior corners of pronotal disk weakly depressed, coarser secondary punctures of pronotal disk present only in lateral fourths; elytra with two complete epipleural striae, outer subhumeral stria absent, inner subhumeral stria present as short basal fragment, dorsal striae 1-2 more or less complete, 3^rd^ stria vaguely present in basal half, fragmented and scratchlike, 4^th^ stria present as very short basal arch, 5^th^ stria absent, sutural stria present for short distance at middle, not meeting basal arch of 4^th^, elytral disk with small, sparse punctures in apical fourth; prosternal keel moderately broad, weakly convex, emarginate at base, carinal striae convergent between coxae, diverging anterad and posterad, complete, free; prosternal lobe short, about one-third keel length, apical margin rounded, marginal stria well impressed at middle, obsolete at sides; mesoventrite weakly produced at middle, marginal stria complete; mesometaventral stria weakly arched at middle, crenulate; base of inner lateral metaventral stria displaced slightly mediad, curving obliquely posterolaterad toward middle of hind coxa, slightly abbreviated at apex, outer lateral metaventral stria subparallel, about half as long as inner; metaventral and abdominal disks impunctate at middle; abdominal ventrite 1 with inner lateral stria nearly complete, outer lateral stria absent; protibiae 4-dentate, outer margin finely serrulate; mesotibia with one marginal spine; outer metatibial margin smooth; propygidium lacking basal stria, with coarse secondary punctures more or less uniformly separated by about their diameters, smaller and denser along basal margin, propygidial glands visible about one-fifth from basal margin, one-fourth from lateral margin; pygidium with fine ground punctation very sparsely interspersed with small secondary punctures, predominantly along basal margin. Male genitalia (Figs 29A–F): T8 about as long as broad, widest at middle, sides outwardly arcuate, basal emargination deep, subacute at middle, basal rim well sclerotized, apex narrowly emarginate, ventrolateral apodemes projecting weakly beneath, about one-third from base, separated by about two-thirds maximum T8 width, rapidly narrowed apically; S8 divided, inner margins approximate only at base, divergent apically, outer margins weakly rounded, slightly convergent, apical guides slightly widened distally, narrowly rounded at apices, apical velar membrane absent, apex lacking conspicuous setae; T9 with basal apodemes rather thin and elongate, nearly one-half total length, T9 apices narrowly rounded, glabrous, ventrolateral apodemes weakly projecting beneath; S9 rather broad, with stem weakly narrowed near midpoint, base rounded, apex expanded, apical emargination broadly subtriangular, broadly desclerotized along midline; tegmen with sides subparallel, weakly narrowed to blunt apices, tegmen in lateral aspect more or less straight, weakly curved ventrad at tip; median lobe simple, about one-half tegmen length; basal piece short, about one-fourth tegmen length.

**Figure 28.**
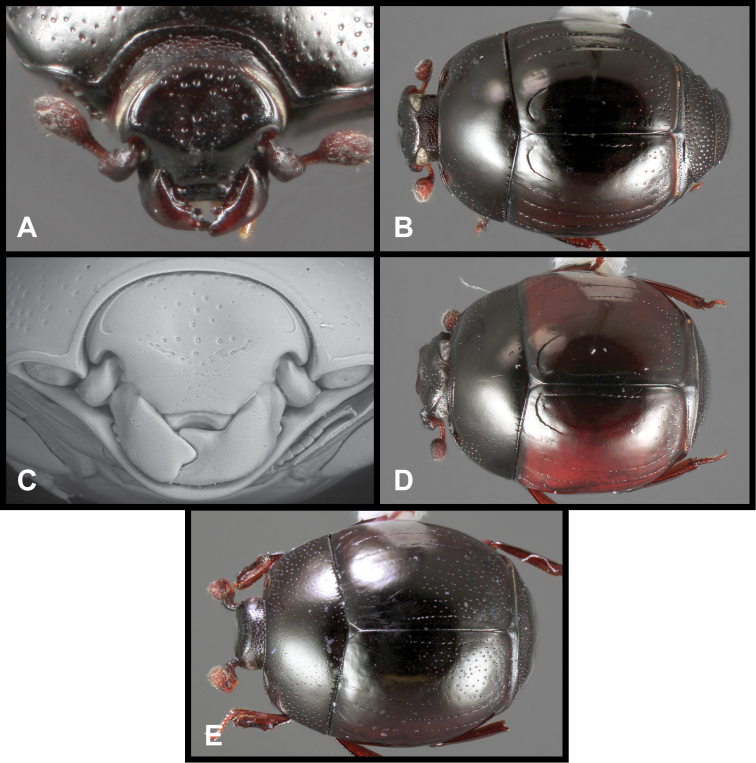
*Baconia salobrus* group. **A** Frons of *Baconia anthracina* **B** Dorsal habitus of *Baconia anthracina* **C** Frons of *Baconia emarginata* **D** Dorsal habitus of *Baconia emarginata* **E** Dorsal habitus of *Baconia obsoleta*.

**Figure 29.**
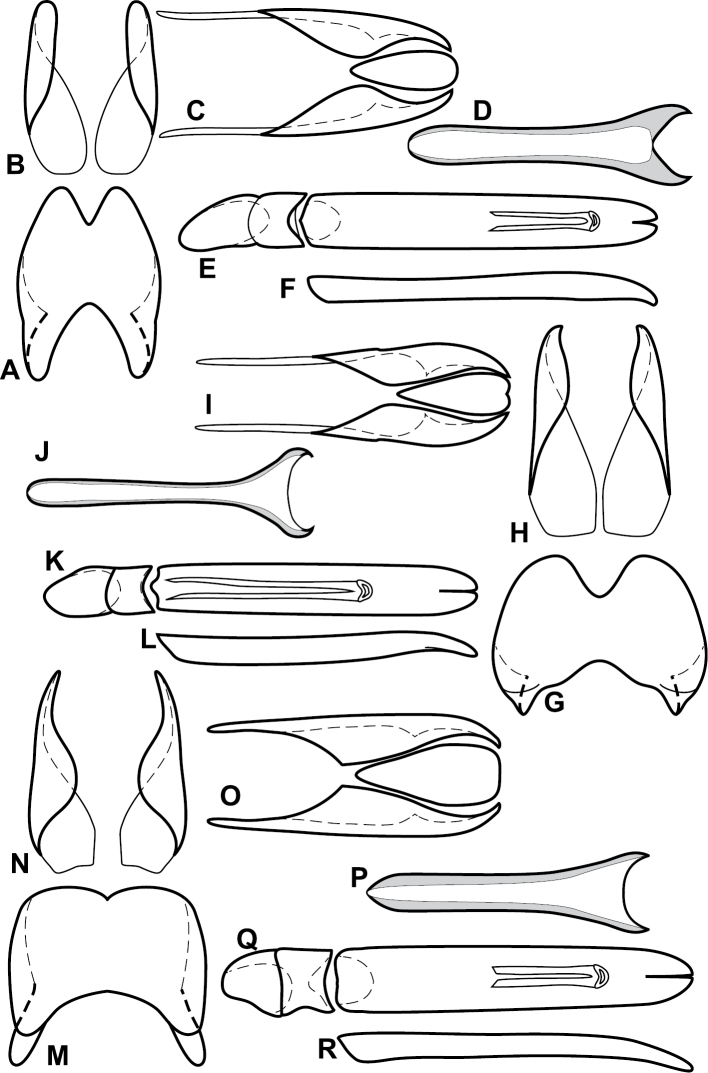
**A–F** Male genitalia of *Baconia anthracina*. **A** T8 **B** S8 **C** T9 & T10 **D** S9 **E** Aedeagus, dorsal view **F** Aedeagus, lateral view **G–L** Male genitalia of *Baconia emarginata* **G** T8 **H** S8 **I** T9 & T10 **J** S9 **K **Aedeagus, dorsal view **L** Aedeagus, lateral view **M–R** Male genitalia of *Baconia obsoleta* **M** T8 **N **S8 **O **T9 & T10 **P** S9 **Q** Aedeagus, dorsal view **R** Aedeagus, lateral view.

###### Remarks.

This species can be distinguished by its unique epistomal fovea (Figs 1A, 28A), a discrete depression immediately anterad, and sometimes continuing, the frontal depression. It is otherwise very similar to *Baconia salobrus*, having an isolated basal arch of the 4^th^ stria (Fig. 28B). The following species, *Baconia emarginata*, is also similar to both of these, but is easily recognized by its deeply emarginate labrum and generally large head and mandibles.

###### Etymology.

This species’ name refers to its black coloration.

##### Baconia emarginata sp. n.

http://zoobank.org/DA838A4C-3A46-4FE1-84A4-20CA6EFA3A31

http://species-id.net/wiki/Baconia_emarginata

Figs 28C–D 29G–L Map 7

###### Type locality.

BRAZIL: Pará: Tucuruí [3.75°S, 49.67°W].

###### Type material.

**Holotype male**: “**BRASIL: Pará**, Tucuruí. 3°45'S, 49°40'W, 27.x.–9.xi.1985, FIT” / “Caterino/Tishechkin Exosternini Voucher EXO-00486” (UFPR). **Paratypes** (2): 1: **BRAZIL**: **Mato Grosso**:Mpio. Cotriguaçu, Fazenda São Nicolau, Prainha, 9°51.6'S, 58°12.9'W, ix.2009, FIT, R. Nunes (CEMT). 1: **FRENCH GUIANA**: Montagne des Chevaux, 4°43'N, 52°24'W, 16.xii.2008, FIT, SEAG (CHND).

###### Other material.

**PERU**:1: **Junín**: 11 km NE Puerto Ocopa, Los Olivos, 11°3.00'S, 74°15.52'W, 1200 m, 29-30.iii.2009, FIT, A. Tishechkin, DNA Extract MSC-2148, EXO-00675 (AKTC).

###### Diagnostic description.

Length: 2.2–2.3mm, width: 2.0–2.1mm; body rather broadly elongate oval, convex, glabrous; piceous, shining; frons elevated over antennal bases, depressed in middle, interocular margins convergent dorsad, frontal disk with few coarse punctures in median depression, epistoma with fairly discrete median fovea, otherwise impunctate, frontal stria present along inner edges of eyes, interrupted above antennal bases and at middle; supraorbital stria absent; antennal scape short, club broadly rounded, slightly asymmetrical; labrum with distal edge weakly carinate, deeply emarginate; mandibles rather short, stout, each with basal tooth; pronotal sides evenly narrowed in basal two-thirds, more strongly arcuate to apices, lateral marginal striae continuous around sides and front, submarginal stria absent, anterior corners of pronotal disk depressed, coarser secondary punctures of pronotal disk present only in lateral thirds; elytra with complete inner epipleural stria and fragments of an additional outer epipleural stria, outer subhumeral stria absent, inner subhumeral stria present as short basal and median fragments, dorsal striae 1-2 more or less complete, either or both variably obsolete anteriorly, 3^rd^ stria present in basal half, very fine, scratchlike, 4^th^ stria present as very short basal arch, obsolete in apical three-fourths or more, 5^th^ stria absent, sutural stria present for short distance at middle, narrowly separated from basal arch of 4^th^, elytral disk with small, sparse punctures in apical fourth; prosternal keel moderately broad, weakly convex, emarginate at base, carinal striae convergent between coxae, diverging anterad and posterad, complete, free; prosternal lobe short, less than one-half keel length, apical margin rounded, marginal stria well impressed at middle, obsolete at sides; mesoventrite weakly produced at middle, marginal stria interrupted medially; mesometaventral stria transverse, crenulate, meeting base of inner lateral metaventral stria, which is displaced slightly mediad, curving obliquely posterolaterad toward middle of hind coxa, slightly abbreviated at apex, outer lateral metaventral stria subparallel, about half as long as inner; metaventral and abdominal disks impunctate at middle; abdominal ventrite 1 with inner lateral stria present in basal half only, outer lateral stria absent; protibiae 4-dentate, marginal spines fine, not prominent, outer margin finely serrulate between; mesotibia with one marginal spine; outer metatibial margin smooth; propygidium lacking basal stria, with coarse secondary punctures separated by about their diameters in basal third, smaller and much sparser posterad, propygidial glands visible about one-fourth from basal and anterolateral margins; pygidium with fine ground punctation very sparsely interspersed with small secondary punctures, predominantly in basal third. Male genitalia (Figs 29G–L): T8 shorter than broad, widest at middle, sides outwardly arcuate, basal emargination shallow, sinuate, basal rim not strongly sclerotized, apex narrowly emarginate, ventrolateral apodemes projecting weakly beneath, about one-fourth from base, separated by about three-fourths maximum T8 width, rapidly narrowed before longitudinal midpoint; S8 divided, longer than T8, inner margins approximate only in basal fourth, divergent apically, outer margins straight, subparallel, apical guides slightly widened distally, narrowly rounded at apices, apical velar membrane absent, apex lacking conspicuous setae; T9 with basal apodemes rather thin and elongate, about one-half total length, T9 apices narrowly rounded, glabrous, ventrolateral apodemes moderately strongly projecting beneath; S9 with stem weakly narrowed near midpoint, base rounded, apex abruptly expanded, apical emargination broadly, shallowly emarginate, broadly desclerotized along midline; tegmen with sides subparallel, weakly narrowed to rounded apex, tegmen in lateral aspect sinuate, dorsal surface somewhat depressed in basal two-thirds, weakly curved ventrad at tip; median lobe large, about three-fourths tegmen length; basal piece about one-third tegmen length.

###### Remarks.

As discussed under *Baconia anthracina*, above, *Baconia emarginata* is very similar to both it and *Baconia salobrus*, but it lacks the epistomal fovea of the former, and has a uniquely deep labral emargination (Fig. 28C), as well as an unusually large head with very strong mandibles. The lone specimen from Peru has more rufescent elytra and has the 4^th^ and sutural elytral striae connected basally, and we exclude it from the type series because of this variation.

###### Etymology.

This species’ name refers to its deeply emarginate labrum.

##### Baconia obsoleta sp. n.

http://zoobank.org/CDC1BA67-D7A3-41AC-8418-DDDF79F6D64B

http://species-id.net/wiki/Baconia_obsoleta

Figs 28E 29M–R Map 7

###### Type locality.

BRAZIL: Santa Catarina: Nova Teutonia [27.18°S, 52.38°W].

###### Type material.

**Holotype male**: “BRÉSIL X-77 Santa Catarina Nova Teutonia F. Plaumann” / “Caterino/Tishechkin Exosternini Voucher EXO-00651” (FMNH). **Paratype** (1): same data as type but collected xii.1969 (FMNH).

###### Diagnostic description.

Length: 2.2–2.3mm, width: 2.0–2.1mm; body ovoid, convex, glabrous; piceous, shining; frons weakly elevated over antennal bases, depressed in middle, interocular margins convergent dorsad, frontal disk with few coarse punctures in median depression and few across vertex, frontal stria present along inner edges of eyes, just bent mediad at sides, otherwise absent across middle; supraorbital stria absent; antennal scape short, club broadly rounded, subtruncate apically; epistoma convex across apex, apical margin straight; labrum with distal edge weakly carinate, emarginate; mandibles rather short, stout, each with basal tooth; pronotal sides convergent from base, more strongly arcuate to apices, lateral marginal striae continuous around sides and front, submarginal stria absent; pronotal disk with anterior corners depressed, with series of large, coarse punctures along posterior margin, smaller punctures present in lateral thirds, only fine ground punctures present at middle; elytra with two complete epipleural striae and fragments of an additional one further laterad, outer subhumeral stria absent, inner subhumeral stria present as short basal fragment, dorsal striae 1-2 more or less complete, 3^rd^ stria present in basal half, 4^th^ and 5^th^ striae absent, sutural stria present for short distance at middle, obsolete in basal and apical thirds, elytral disk with small, sparse punctures in apical third; prosternal keel weakly convex, emarginate at base, carinal striae subparallel basally, diverging anterad, complete, free; prosternal lobe short, about one-half keel length, apical margin rounded, marginal stria well impressed at middle, obsolete at sides; mesoventrite produced at middle, marginal stria complete; mesometaventral stria sightly arched forward at middle, crenulate, slightly detached from inner lateral metaventral stria (at least in types), which curves obliquely posterolaterad toward middle of hind coxa, outer lateral metaventral stria subparallel, about half as long as inner; metaventral disk impunctate at middle; abdominal ventrite 1 with inner lateral stria present in basal half only, outer lateral stria absent, disk with few punctures across middle of posterior margin; protibiae 4-dentate, outer margin finely serrulate between spines; mesotibia with one marginal spine; outer metatibial margin smooth; propygidium lacking basal stria, with coarse secondary punctures separated by slightly more than their diameters in basal half, smaller and much sparser posterad, propygidial glands inconspicuous; pygidium with fine ground punctation very sparsely interspersed with small secondary punctures, predominantly along basal margin. Male genitalia (Figs 29M–R): T8 shorter than broad, widest at middle, sides outwardly arcuate, basal emargination appearing weakly triangulate, basal rim well-sclerotized, apex shallowly, acutely emarginate, ventrolateral apodemes projecting very weakly beneath, extending nearly halfway along length, but inner apices widely separated; S8 divided, slightly longer than T8, inner margins rather well separated in basal fourth, divergent apically, outer margins weakly rounded, convergent, apical guides most strongly developed at their bases, narrowed strongly to apices, apical velar membrane absent, apex lacking conspicuous setae; T9 with basal apodemes rather thin, about one-third total length, T9 apices narrowly rounded, glabrous, ventrolateral apodemes weakly projecting beneath; S9 rather broad, sides subparallel in basal half, base rounded, apex gradually expanded, apical emargination broadly, shallowly emarginate, broadly desclerotized along midline, moreso in apical half; tegmen with sides subparallel, weakly narrowed in apical third, tegmen more or less straight in lateral aspect, weakly curved ventrad in apical fifth; median lobe broad, almost one-half tegmen length; basal piece short, about one-fourth tegmen length.

###### Remarks.

This species is strongly convex, with the pronotal disk narrowed rapidly from base, and pronotal punctures relatively widespread (Fig. 28E). Like *Baconia crassa* and *Baconia turgifrons*, this species completely lacks the 4^th^ and 5^th^ elytral striae, but *Baconia obsoleta* does not have the peculiar epistomal modifications of either of these species.

###### Etymology.

This species’ name refers to the extensively effaced elytral striae.

#### *Baconia ruficauda* group

This group contains only two distinctive species that share an elongate, more or less parallel-sided, subdepressed body form, a strongly convex frons lacking at least the central portion of the frontal stria, nearly complete elytral striation, though with various striae abbreviated from the apices, and a detached, strongly arched mesometaventral stria. Only *Baconia ruficauda* is represented by a male in the material available for study, so it is impossible to characterize the group on genitalic characters. However, what is available is not obviously different from males of the *Baconia loricata* group, with thin, elongate proximal apodemes on the 9^th^ tergite, and a relatively simple, narrow aedeagus.

##### Baconia ruficauda sp. n.

http://zoobank.org/666DB6CC-BCDC-49F8-B2CF-FE88AF997BAE

http://species-id.net/wiki/Baconia_ruficauda

**Figure 30.**
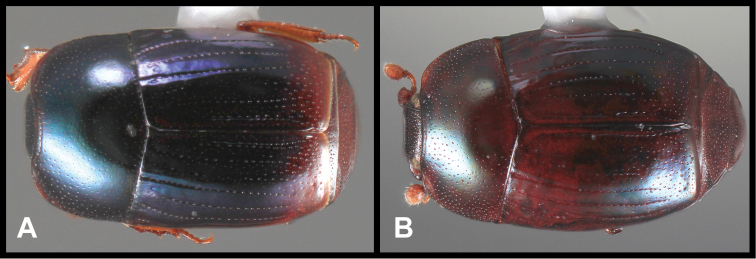
*Baconia ruficauda* group. **A** Dorsal habitus of *Baconia ruficauda* **B** Dorsal habitus of *Baconia repens*.

**Map 8.**
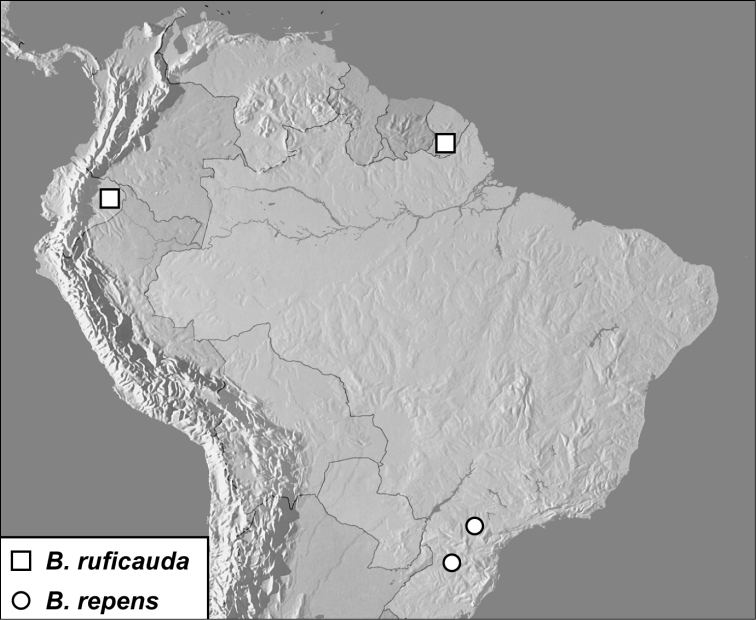
*Baconia ruficauda* group records.

Figs 30A 31A–C Map 8

###### Type locality.

ECUADOR: Orellana: Tiputini Biodiversity Station [0.635°S, 76.150°W].

**Type material. Holotype male**: “**ECUADOR: Depto. Orellana,** Tiputini Biodiversity Station 0°37'55"S, 76°08'39"W, 220–250m. 2 October 1996 T.L.Erwin *et al*. collectors” / “insecticidal fogging of mostly bare green leaves, some with covering of lichenous or bryophytic plants **Lot 1718 Trans. 6 Sta. 8**” / “Caterino/Tishechkin Exosternini Voucher EXO-00469” (USNM). **Paratypes** (3):same locality and data as type except as noted: 1: 21.vi.1996 (USNM), 1: 23.i.2006 (USNM), 1: 9.vii.2006, DNA Extract MSC-2135, EXO-00666 (MSCC).

###### Other material.

1: **FRENCH GUIANA:** Belvèdére de Saül, 3°1'22"N, 53°12'34"W, FIT, 17.i.2011, SEAG (CHND).

###### Diagnostic description.

**Figure 31.**
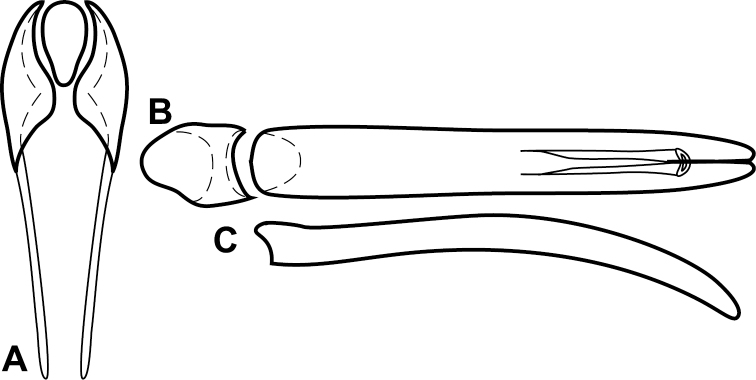
Male genitalia of *Baconia ruficauda*. **A** T9 & T10 **B** Aedeagus, dorsal view **C** Aedeagus, lateral view.

Length: 1.5–1.6mm, width: 1.0–1.1mm; body elongate, parallel-sided, weakly depressed, glabrous; head and pronotum metallic greenish-blue, very slightly contrasting with blue elytra, apical margin of elytra, pygidia, and venter rufescent; frons broad, together with epistoma strongly convex, interocular margins strongly convergent dorsad, few coarse punctures present throughout, especially at middle and toward vertex, frontal and supraorbital striae absent; antennal scape short, club rounded; epistoma weakly emarginate apically; labrum about 3×wider than long, apex weakly emarginate; mandibles short, left mandible with small acute tooth, right mandible only bluntly produced at base; pronotal sides subparallel in basal two-thirds, arcuate to apex, marginal stria complete around lateral and anterior margins, slightly removed from anterior margin, submarginal stria absent; pronotal disk with fine ground punctation conspicuous throughout, with small secondary punctures interspersed in lateral thirds; elytra with two complete epipleural striae, outer subhumeral stria absent, inner subhumeral stria faintly impressed at base, dorsal striae 1-3 complete, 4^th^ stria usually interrupted to obsolete in apical half, 5^th^ stria present in basal two-thirds, sutural stria nearly complete, weakly abbreviated basally and apically, elytral disk with very sparse secondary punctures in apical third; prosternal keel narrow, convex, base truncate, carinal striae complete to fragmented basally and apically; prosternal lobe about two-thirds keel length, deflexed, apically rounded, marginal stria slightly fragmented at sides; mesoventrite subacutely produced at middle (inconsistent with truncate prosternal keel), marginal stria narrowly interrupted at middle; mesometaventral stria narrowly arched forward, crenulate, meeting inner lateral metaventral stria close to mesocoxa, weakly curving obliquely posterolaterad toward outer third of metacoxa, outer lateral metaventral stria absent, metaventral and 1^st^ abdominal disks impunctate at middle; abdominal ventrite 1 with complete inner lateral stria and posterior fragments of outer stria; protibia with four marginal denticles, the basalmost weak, outer margin serrulate between; mesotibia with two distinct marginal spines; outer metatibial margin smooth; propygidium without transverse basal stria, with moderately large, ocellate punctures sparsely scattered at middle, smaller and denser toward anterior margin; propygidial gland openings inconspicuous; pygidium with fine, sparse ground punctation throughout, with small, sparse secondary punctures more conspicuous in basal half. Male genitalia (Figs 31A-C): T8, S8, S9 weakly sclerotized and poorly preserved, few details discernable: basal rim slightly sclerotized, basal emargination shallow, subangulate, ventrolateral apodemes extending beneath about one-half T8 length; T9 with basal apodemes long, thin, almost two-thirds total length, T9 apices narrow, acute, lacking setae, ventrolateral apodemes weak; T10 small, entire; tegmen widest one-fourth from base, weakly and unevenly narrowed to apex, tegmen in lateral aspect rather thick throughout, evenly curved in apical two-thirds; median lobe simple, about one-third tegmen length; basal piece short, about one-fifth tegmen length.

**Figure 32.**
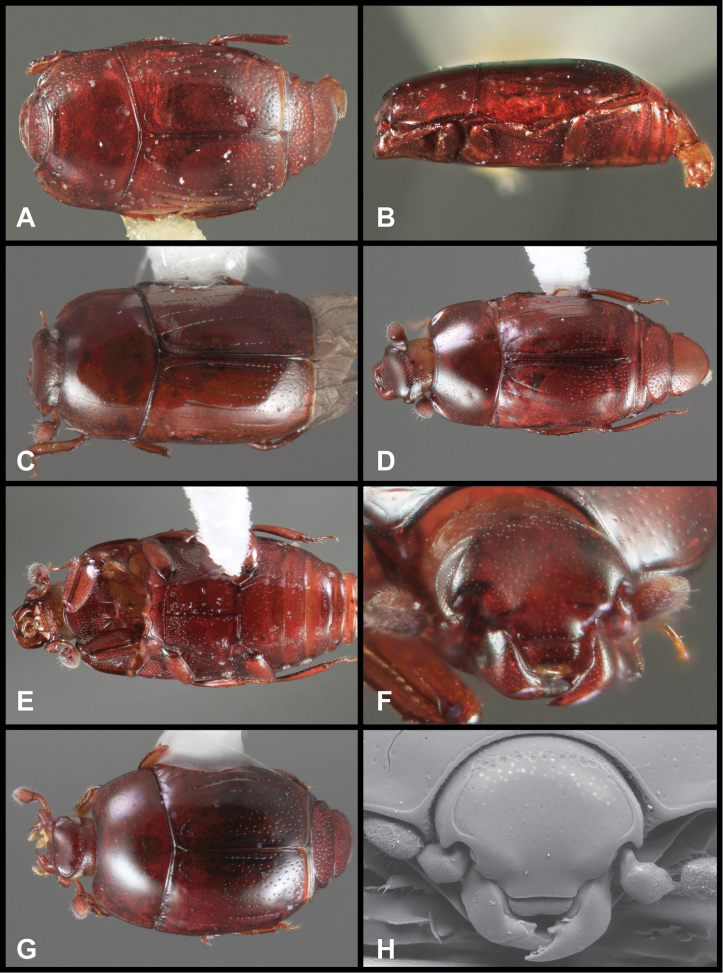
*Baconia angusta* group. **A** Dorsal habitus of paralectotype of *Baconia angusta* **B** Ventral habitus of paralectotype of *Baconia angusta* **C** Dorsal habitus of *Baconia incognita* **D** Dorsal habitus of *Baconia guartela* **E** Ventral habitus of *Baconia guartela* **F** Frons of *Baconia guartela* **G** Dorsal habitus of *Baconia bullifrons* **H** Frons of *Baconia bullifrons*.

###### Remarks.

Within this group, this species is by far the smaller of the two. Its distinctive color pattern (Fig. 21A) is largely sufficient to distinguish it, with the metallic blue elytra becoming apically rufescent to match the pygidia and venter. In addition, the 4^th^ elytral stria is significantly abbreviated from the apex in most individuals. It is superficially similar to *Baconia tricolor*, in the *Baconia insolitus* group, in body shape and coloration. But the two differ greatly in genitalic characters, and *Baconia ruficauda* is not externally setose.

###### Etymology.

This species is named for its rufescent posterior end.

##### Baconia repens sp. n.

http://zoobank.org/A0D6D638-84AD-4E5F-8584-D71663A30D77

http://species-id.net/wiki/Baconia_repens

Fig 30B Map 8

###### Type locality.

BRAZIL: Santa Catarina: Nova Teutonia [27.18°S, 52.38°W].

###### Type material.

**Holotype female**: “Nova Teutonia, Sta Catharina, BRAZ. I:8:1958 Fritz Plaumann leg.” / “FMNH-INS 0000 069 307” (FMNH). **Paratype** (1): **BRAZIL: Santa Catarina**: Nova Teutonia, i.1963, F. Plaumann (FMNH).

###### Other material.

1: **BRAZIL**: **Paraná**: Telêmaco Borba Klabin S.A., 24°18.9'S, 50°30.6'W, 22.ix.2006, baited FIT, *Pinus taeda* stand, C. Flechtmann (UNESP).

###### Diagnostic description.

Length: 1.9–2.0mm, width: 1.4–1.6mm; body elongate, sides weakly rounded, subdepressed, glabrous; body rufobrunneus with strong metallic tinge; frons together with epistoma strongly convex, interocular margins moderately convergent dorsad, few coarse punctures present throughout, especially toward vertex, frontal and supraorbital striae absent, marginal ocular punctures subserially arranged; antennal scape short, club rounded; epistoma straight apically; labrum about 3×wider than long, apex weakly emarginate; mandibles short, both with small acute tooth; pronotal sides weakly convergent from base, marginal stria complete around lateral and anterior margins, submarginal stria absent; pronotal disk weakly depressed in anterior corners, convex along rest of lateral margin, disk with fine ground punctation and conspicuous secondary punctures almost throughout, relatively impunctate only in small area posterad center of disk; elytra with two to three complete epipleural striae, the outermost may be fragmented, outer subhumeral stria absent, inner subhumeral stria faintly impressed at base, dorsal striae 1-5 present to base, increasingly abbreviated apically, with 1^st^ complete and 5^th^ present in basal two-thirds only, sutural stria nearly complete, but variably, slightly abbreviated basally and apically, elytral disk with very sparse secondary punctures in apical fourth; prosternal keel narrow, weakly convex, base narrowly, shallowly emarginate, carinal striae complete may unite apically; prosternal lobe about two-thirds keel length, weakly deflexed, apical margin rounded, marginal stria slightly fragmented at sides; mesoventrite narrowly, subacutely produced at middle, marginal stria complete; mesometaventral stria broadly arched forward, crenulate, separated from inner lateral metaventral stria, which extends from apex of marginal mesoventral stria, curving obliquely posterolaterad toward outer third of metacoxa, outer lateral metaventral stria present along basal half of inner stria, metaventral and 1^st^ abdominal disks impunctate at middle; abdominal ventrite 1 with complete inner lateral stria, outer stria complete or nearly so; protibia with four rather conspicuous marginal denticles, outer margin very finely serrulate between; mesotibia with two distinct marginal spines; outer metatibial margin smooth; propygidium without transverse basal stria, with ocellate punctures more or less uniformly separated by slightly less than their diameters; propygidial gland openings evident about one-third from anterior margin and almost one-third from lateral margins; pygidium flat to weakly depressed along lateral margins, with fine, sparse ground punctation throughout, with small, sparse secondary punctures more conspicuous in basal half. Male genitalia: not known.

###### Remarks.

This species shares many characters with the preceding, but is distinctly larger, has the body shape a bit more oval, less purely parallel-sided (Fig. 30B), and is much less distinctly bicolored, with the elytra only faintly metallic to their apices. The specimen from Paraná is more distinctly metallic in coloration, and exhibits some slight differences in elytral striae, and is therefore excluded from the type series.

###### Etymology.

This species’ name means crawling or creeping, also new or unexpected.

#### *Baconia angusta* group

The *Baconia angusta* group comprises nine very similar species, all of them best separated by reference to highly divergent and varied male genitalia. Externally, the group is easily characterized by their elongate, parallel-sided, convex to subdepressed, and rufescent body form. All of the species lack a frontal stria, possess a lateral submarginal pronotal stria, have the base of the pronotal keel distinctly and subangulately emarginate, and have the 4^th^ elytral stria curved mediad at the base, usually meeting the sutural stria in a narrow to subangulate basal arch. The male genitalia of the group is characterized by a broad aedeagus, 8^th^ sternite usually setose apically, with poorly developed or closed apical guides, and short, stemless spiculum gastrale (S9). Some characters suggest that this group may be close to or sister to the *Baconia aeneomicans* group, particularly the closed apical guides. However, the absence of a stem of the spiculum gastrale strongly supports the monophyly of the group.

##### Baconia angusta

Schmidt, 1893

http://species-id.net/wiki/Baconia_angusta

**Map 9.**
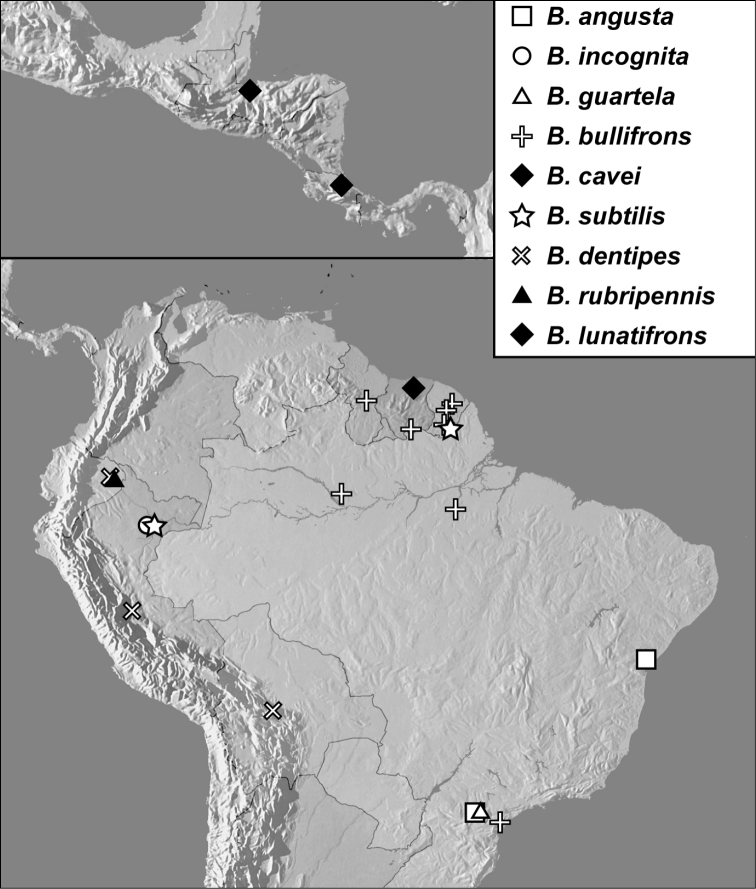
*Baconia angusta* group records. Record for *Baconia angusta* in Bahia, Brazil is a state record only.

Figs 32A–B 33A–F Map 9

Baconia angusta Schmidt, 1893a: 11; *Phelister angustus*: Lewis 1901: 372; *Baconia angusta*: Mazur 1997: 25.

###### Type locality.

BRAZIL [exact locality unknown].

###### Type material.

**Lectotype male**, here designated (ZMHB): “Brasilia” / “Type” / “*Baconia angusta* Schmidt, 1893. ex. Coll. Schmidt-Bickhardt” / “LECTOTYPE *Baconia angusta* Schmidt, 1893, M.S.Caterino & A.K.Tishechkin des. 2010” (ZMHB). **Paralectotype**: “Havana” / “Type” / “*angusta* M.” / “*angustus* Schm” / “Coll. J. Schmidt” / “*Baconia angusta* Schmidt, 1893. ex. Coll. Schmidt-Bickhardt”/ “PARALECTOTYPE *Baconia angusta* Schmidt, 1893, M.S.Caterino & A.K.Tishechkin des. 2010” (ZMHB). This species was described from an unspecified number of specimens, and the lectotype designation fixes primary type status on one of two original specimens.

###### Other material.

**BRAZIL**: 6: **Bahia** (BMNH); 1: **Paraná**: Parque Estad. Guartelá, Mpio. Tibagi, 24.5663°S, 50.2570°W, 12–15.xii.2011, FIT, forest, M. Caterino & A. Tishechkin, DNA Extract MSC-2277, EXO-00931 (MSCC).

###### Diagnostic description.

**Figure 33.**
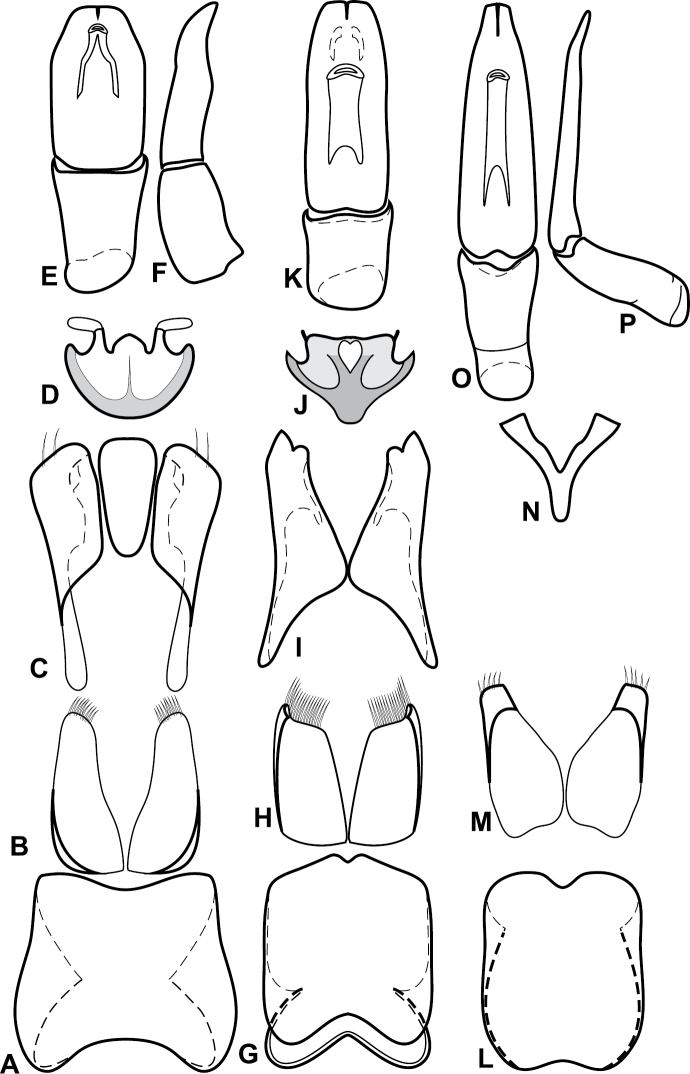
**A–F** Male genitalia of *Baconia angusta*. **A** T8 **B** S8 **C** T9 & T10 **D** S9 **E** Aedeagus, dorsal view **F** Aedeagus, lateral view **G–K** Male genitalia of *Baconia incognita*. **G** T8 **H** S8 **I** T9 & T10 **J** S9 **K **Aedeagus, dorsal view **L–P** Male genitalia of *Baconia guartela*. **L** T8 **M** S8 **N** S9 **O** Aedeagus, dorsal view **P** Aedeagus, lateral view.

Length: 1.3–1.8mm, width: 0.9–1.3mm; body elongate, parallel-sided, depressed, glabrous; color rufescent, shining; head with frons elevated over antennal bases, slightly depressed at middle, interocular margins convergent dorsad, frontal punctation sparse, diminished in anterior half, frontal stria absent, supraorbital stria vaguely indicated by serial punctures; antennal scape short, club rounded, slightly expanded apically; epistoma flat, apex weakly emarginate; labrum about 3× wider than long, apical margin weakly emarginate; mandibles short, each with median tooth; pronotum with sides subparallel in basal half, rounded to apex, lateral marginal and lateral submarginal striae very close throughout, the submarginal stria ending freely near anterior corner, marginal stria continuous along anterior margin; pronotal disk narrowly depressed along anterolateral margin, ground punctation fine, small secondary punctures rather well-impressed in lateral fourth; elytra with two more or less complete epipleural striae, outer and inner subhumeral striae absent, dorsal stria 1 nearly complete, fine, scratchlike in apical third, striae 2-4 progressively more abbreviated apically, basal arch of 4^th^ subangulate, meeting base of sutural stria, 5^th^ stria absent, sutural stria obsolete in apical fourth, elytral disk with small, shallow secondary punctures in apical fourth; prosternal keel weakly convex between striae, shallowly emarginate at base, carinal striae weakly divergent basally and apically; prosternal lobe about one-half keel length, apical margin rounded, marginal stria obsolete at sides; mesoventrite produced at middle, marginal stria complete; mesometaventral stria weakly arched forward, crenulate, continued by inner lateral metaventral stria obliquely posterad toward middle of metacoxa, outer lateral metaventral stria very short to absent; metaventral disk impunctate at middle; abdominal ventrite 1 with inner lateral stria complete, outer lateral stria present as short postcoxal fragment, disk impunctate at middle, ventrites 2-5 finely, sparsely punctate across middle; protibia with four weak marginal denticles more or less evenly spaced, margin serrulate between; mesofemur with posterior marginal stria continuous around distal margin; mesotibia with two distinct marginal spines; outer metatibial margin smooth; propygidium lacking basal stria, with moderately large ocellate punctures separated by slightly less than their diameters, propygidial gland openings present nearly one-half behind anterior margin, surrounded by small impuncate area; pygidium with fine ground punctation rather dense, small secondary punctures throughout, slightly sparser toward apex. Male genitalia (Figs 33A–F): T8 subquadrate, about as long as broad, weakly narrowed to apex, basal emargination shallow, basal rim moderately well-sclerotized, apex very shallowly, broadly emarginate, ventrolateral apodemes extending nearly to longitudinal midline, inner apices separated by one-eighth T8 width; S8 divided, slightly shorter than T8, flat, inner margins approximate at base, divergent apically, outer margins weakly rounded, convergent, apical guides undeveloped, apical velar membrane absent, apex with numerous conspicuous setae; T9 with basal apodemes stout, nearly one-half total length, dorsal lobe of T9 broad, subquadrate, bearing few (~2) conspicuous setae, ventrolateral apodemes sinuate along inner edge, with blunt subapical tooth; S9 broad, stem absent, basal margin semicircular, sclerotized along basal margin and extending distad along midline, apex with blunt central process, and complex, curving digitform processes on either side; tegmen very short and broad, sides straight and subparallel in basal two-thirds, convergent to wide, truncate apex, with inner apical sclerotizations of uncertain function, tegmen in lateral aspect widest at middle, apical half broadly excavate beneath; median lobe with diverging proximal apodemes, about one-third tegmen length; basal piece long, wide, about two-thirds tegmen length.

###### Remarks.

Externally, *Baconia angusta* is moderately distinctive in this group, being the smallest and most distinctly depressed (Figs 32A–B) yet known. However, its genitalia are highly distinct, with a very short, broad aedeagus with a long basal piece, an almost semicircular spiculum gastrale with unique digitiform apical processes, and a 9^th^ tergite with complex, bluntly dentate ventromedial lobe

The type locality of this species is unclear. In the original description Schmidt mentions “Hab. Havana, Brasilia”. First of all it is apparent that this corresponds to two distinct localities (corresponding to the two syntypes we found.) Although Mazur (1984, 1997) cites only Brazil for the species’ distribution, we can find no record of a Havana in Brazil. It conceivably refers to Havana, Cuba, but this would be well outside the known distribution for this entire group of species. Because of this doubt, and the fact that the Havana specimen is female, we selected the Brasilia specimen as the lectotype. At the same time, Brasilia probably refers only to the country in general, as the city of Brasilia had not been founded at the time of description.

##### Baconia incognita sp. n.

http://zoobank.org/454B443A-DB5A-49C3-BD72-85EE24B388DB

http://species-id.net/wiki/Baconia_incognita

Figs 32C 33G–K Map 9

###### Type locality.

PERU: Loreto: Rio Nanay [3.90°S, 73.55°W].

###### Type material.

**Holotype male**: “**PERU: Loreto** 45 km W Iquitos, Rio Nanay, Porvenir 3°54'S, 73°33'W White sand, piège vitre. 20.vi.2011 G.Lamarre. P3 V1 WS” / “Caterino/Tishechkin Exosternini Voucher EXO-02503” (MNHN).

###### Diagnostic description.

Length: 1.4mm, width: 0.9mm; body elongate, parallel-sided, weakly depressed, glabrous; color rufescent, shining; head with frons slightly elevated over antennal bases, weakly depressed at middle, interocular margins convergent dorsad, frontal punctation moderately coarse, finer and sparser toward epistoma, frontal stria absent, supraorbital stria vaguely represented by few confluent punctures; antennal scape short, club short, rounded; epistoma truncate; labrum about 3× wider than long, apical margin emarginate; mandibles short, each with median tooth; pronotum with sides subparallel in basal two-thirds, rounded to apex, lateral marginal and lateral submarginal striae merging behind anterior corner, narrowly detached from median part of anterior marginal stria; pronotal disk narrowly depressed along anterolateral margin, ground punctation extremely fine, very sparse, with small secondary punctures only in lateral fourths; elytra with inner epipleural stria complete, outer epipleural stria present in basal half, outer and inner subhumeral striae absent, dorsal stria 1 present in basal half, stria 2 nearly complete, 3^rd^ stria present in basal two-thirds, 4^th^ stria slightly shorter, arched to meet base of sutural stria, 5^th^ stria absent, sutural stria obsolete in apical fourth, elytral disk with small, shallow secondary punctures in apical third, mediad stria 3; prosternal keel narrow, flat, emarginate at base, carinal striae convergent at middle, diverging slightly, fragmented toward front; prosternal lobe about two-thirds keel length, apical margin rounded, marginal stria obsolete at sides; mesoventrite produced at middle, marginal stria complete; mesometaventral stria arched forward at middle, crenulate, detached from base of inner lateral metaventral stria, which extends obliquely posterad toward inner third of metacoxa, outer lateral metaventral stria very short, present as postmesocoxal fragment; metaventral disk impunctate at middle; abdominal ventrite 1 with complete inner lateral stria, outer lateral stria in apical half, disk impunctate at middle, ventrites 2–5 very finely, sparsely punctate across middle; protibia narrow, with four marginal denticles, the middle pair distant, margin serrulate between; mesofemur with posterior marginal stria weakly curving anterad along apical margin; mesotibia with two distinct marginal spines; outer metatibial margin smooth; propygidium lacking basal stria, with dense, ocellate secondary punctures separated by less than their diameters, propygidial gland openings inconspicuous; pygidium with fine ground punctation, small secondary punctures sparsely scattered, slightly smaller and sparser at middle of disk. Male genitalia (Figs 33G–K): very similar to that of *Baconia angustus*, but dorsal lobes of T9 more distinctly triangular, ventrolateral tooth better developed; S9 quite different in overall shape, with base subangulate, with only very fine and weakly developed apical processes; aedeagus slightly longer, relatively narrower, basal piece less than one-half tegmen length.

###### Remarks.

This species is very closely related to *Baconia angusta*, but is relatively easily separated by distinctive features of the male genitalia. Externally, the overall body shape is more elongate (Fig. 32C) and slightly less depressed, its frons is more distinctly convex, the submarginal impression of the pronotum is weaker, and the secondary punctures of both pronotal sides and elytral apices are finer and shallower. The aedeagus of *Baconia incognita* is not quite as short, and the basal piece is proportionally shorter. The spiculum gastrale has very thin apical processes, but nothing like the prominent processes of *Baconia angusta*.

###### Etymology.

This species’ name refers to the fact that it was only discovered to be distinct very late in the process of assembling this manuscript.

##### Baconia guartela sp. n.

http://zoobank.org/33858360-BAB0-4A4E-8052-61F3951CCC07

http://species-id.net/wiki/Baconia_guartela

Figs 32D–F 33L–P Map 9

###### Type locality.

BRASIL: Paraná: Parque Estad. Guartelá [24.5663°S, 50.2570°W].

###### Type material.

**Holotype male**: “**BRASIL: Paraná**, Mpio. Tibagi, Parque Estad. Guartelá 24.5663°S, 50.2570°W. F.I.T., forest. 12–15.xii.2011. AT196 M.S.Caterino & A.K.Tishechkin” / “Caterino DNA Voucher Extraction: MSC-2278, sp.: Baconia ~angusta2, Extraction Date: i.24.2012” / “Caterino/Tishechkin Exosternini Voucher EXO-00934” (UFPR).

###### Diagnostic description.

Length: 1.6mm, width: 1.1mm; body elongate, parallel-sided, subdepressed, glabrous; color rufescent, shining; frons broad, weakly elevated over antennal bases, more or less flat across middle, interocular margins convergent dorsad, frontal punctation fine, sparse, only few larger punctures near vertex, frontal stria absent, supraorbital stria well impressed; antennal scape short, club large, rounded; epistoma flat, apex weakly emarginate; labrum about 3× wider than long, slightly narrowed to apex, apical margin faintly arcuate; mandibles short, each with median tooth; pronotum with sides subparallel in basal two-thirds, rounded to apex, lateral marginal and lateral submarginal striae very close throughout, submarginal stria ending freely near anterior corner, marginal stria continuous along anterior margin, slightly removed from margin, weakly crenulate; pronotal disk narrowly depressed along anterolateral margin, ground punctation fine, lacking secondary punctures along middle third, sparsely impressed at sides; elytra with two more or less complete epipleural striae, outer and inner subhumeral striae absent, dorsal stria 1 present in basal half only, stria 2 nearly complete, striae 3–4 progressively abbreviated apically, basal arch of 4^th^ stria subangulate, meeting base of sutural stria, 5^th^ stria absent, sutural stria obsolete in apical fourth, elytral disk with small, shallow secondary punctures in apical fourth, particularly toward suture; prosternal keel weakly convex between striae, emarginate at base, carinal striae weakly divergent basally and apically; prosternal lobe about one-half keel length, apical margin rounded, marginal stria obsolete at sides; mesoventrite produced at middle, marginal stria interrupted for width of median process; mesometaventral stria weakly arched forward, crenulate, continued by inner lateral metaventral stria obliquely posterad toward middle of metacoxa, outer lateral metaventral stria present as very short postcoxal fragment; metaventral disk impunctate at middle; abdominal ventrite 1 with inner and outer lateral striae nearly complete, slightly abbreviated apically, disk impunctate at middle, ventrites 2-5 nearly impunctate across middle; protibia narrow, with three weak marginal denticles, the distal two relatively close, margin serrulate between; mesofemur with posterior marginal stria largely confined to posterior margin; mesotibia with two distinct marginal spines; outer metatibial margin smooth; propygidium lacking basal stria, with moderately large ocellate punctures more or less uniformly separated by about their diameters, propygidial gland openings inconspicuous; pygidium with fine ground punctation sparse, small secondary punctures diminishing from base to apex. Male genitalia (Figs 33L–P): T8 subquadrate, slightly longer than broad, sides straight, parallel, basal emargination very shallow, basal rim not strongly sclerotized, apex shallowly emarginate, ventrolateral apodemes poorly developed, inner apices separated by three-fourths T8 width; S8 divided, slightly shorter than T8, inner margins approximate in basal third, divergent apically, outer margins diverging apically, apical guides closed, apical velar membrane absent, apex with 5–6 conspicuous setae; [T9–10 poorly preserved and prepared]; S9 forming a rather shallow ‘V’, stem absent, base bluntly rounded, apices divergent and truncate; tegmen moderately broad, widest near base, narrowed distally, apices obliquely truncate, tegmen in lateral aspect more or less flat, apex very slightly bent ventrad; median lobe long, about two-thirds tegmen length; basal piece long, over one-half tegmen length, strongly bent relative to tegmen.

###### Remarks.

The simple, ‘V’-shaped spiculum gastrale is unique to this species, as is the distinct angle between the basal piece and the tegmen of the aedeagus. Externally, its relatively smooth, impunctate frons (Fig. 32F), and complete, transverse, crenulate mesometaventral stria (Fig. 32E) will help to characterize it.

###### Etymology.

This species is named for the Cânion Guartelá, where the authors collected the type during a memorable 2011 expedition. The name is a noun in apposition.

##### Baconia bullifrons sp. n.

http://zoobank.org/51423784-5445-4116-AB7F-0FE2D70F742D

http://species-id.net/wiki/Baconia_bullifrons

Figs 32G–H 34A–H Map 9

###### Type locality.

GUYANA: Region 8: Iwokrama Forest [4.28°N, 58.51°W].

**Type material. Holotype male**: “**GUYANA: Region 8**, Iwokrama Forest, Kabocalli Field Stn., 60 m 4°17'4"N, 58°30'35"W, 3–5 JUN 2001; R.Brooks,Z.Falin, GUY1BF01 146, ex: flight intercept trap” / “SM0567606” (SEMC). **Paratypes** (7): **FRENCH GUIANA**: 1:Rés. des Nouragues, Camp Inselberg, 4°05'N, 52°41'W, 25.i.2011, FIT, SEAG; 1:Rés. des Nouragues, Saut-Parare, 4°02'16.1"N, 52°40'21.1"W, 20.x.2009, FIT, S. Brule; 3:Belvèdére de Saül, 3°1'22"N, 53°12'34"W, 7.ii.2011, FIT, SEAG; 1:Montagne des Chevaux, 4°43'N, 52°24'W, 1.viii.2009, FIT, SEAG (CHND). 1: **SURINAME**: **Sipaliwini**: CI-RAP Survey camp 1, upper Palumeu, 225m. 2.47700°N, 55.62941°W, FIT, 10-16.iii.2012, A.E.Z. Short. SR12-0310-TN1 (SEMC).

###### Other material.

(5): **BRAZIL**: 1: **Manaus**: INPA, 2°25'S, 59°50'W, i.1994, Winklered leaf litter, terra firme forest, R. Didham; 2: **Pará**: Monte Alegre, 3°09'S, 52°03'W, 17.vi-3.vii.1992, FIT; **Santa Catarina**: Nova Teutonia, ii.77, F. Plaumann (MHNG); 1: **Paraná**: Piraquara, Mananciais de Serra, 25°29.77'S, 48°58.90'W, 1000 m, 17–31.x.2007, FIT, P. Grossi & D.Parizotto (UFPR).

###### Diagnostic description.

**Figure 34.**
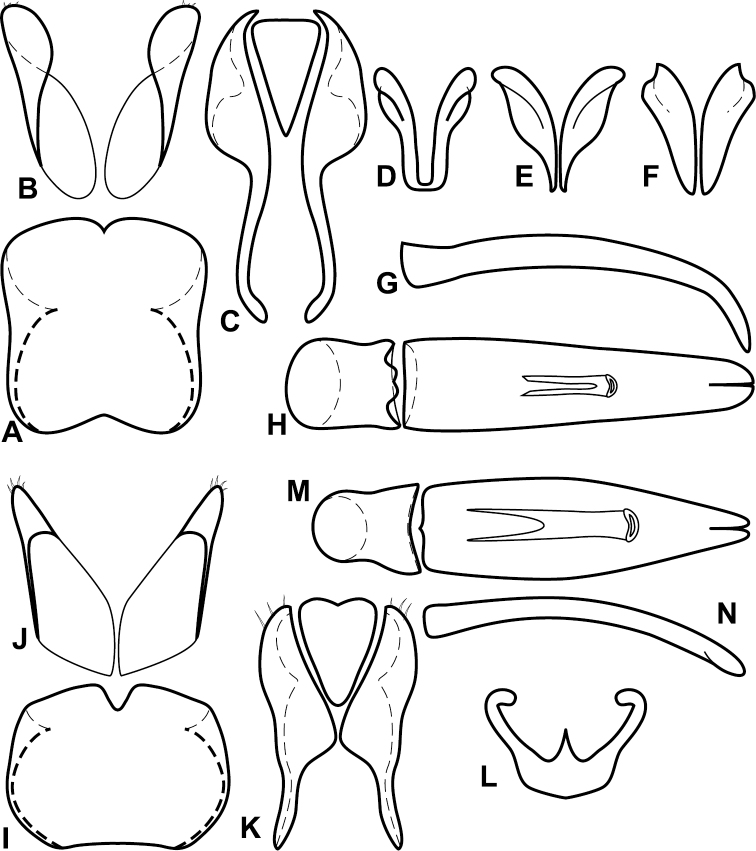
**A–H** Male genitalia of *Baconia bullifrons*. **A** T8 **B** S8 **C** T9 & T10 **D** S9 **E** S9 (variant) **F **S9 (variant) **G** Aedeagus, lateral view **H** Aedeagus, dorsal view **I–N** Male genitalia of *Baconia cavei* **I** T8 **J** S8 **K** T9 & T10 **L** S9 **M** Aedeagus, dorsal view. **N** Aedeagus, lateral view.

Length: 1.0–1.2mm, width: 0.9–1.0mm; body elongate oval, moderately depressed, glabrous; color rufescent, shining; head with frons strongly elevated over antennal bases as oblique carinae, interrupted, depressed at middle, interocular margins convergent dorsad, frontal punctation very fine and sparse, few punctures near vertex, frontal stria present along inner margins of eyes and along outer half of each oblique carina, supraorbital stria vaguely represented by few confluent punctures; antennal scape short, club short, rounded; epistoma weakly emarginate; labrum about 3× wider than long, apical margin truncate to weakly produced; mandibles short, each with median tooth; pronotum with sides weakly convergent in basal two-thirds, abruptly narrowed to apex, marginal stria complete along lateral and anterior margins, lateral submarginal stria present in basal two-thirds, ending freely; pronotal disk not depressed in anterior corners, ground punctation extremely fine, small secondary punctures present only in middle of lateral fourths; elytra with single complete epipleural stria, outer and inner subhumeral striae absent, dorsal stria 1 present in slightly less than basal half, stria 2 nearly complete, 3^rd^ stria present in basal half or slightly more, 4^th^ stria shorter, present in basal third and arched to meet base of sutural stria, 5^th^ stria absent, sutural stria obsolete in apical fifth, elytral disk with small, shallow secondary punctures in apical half, mediad stria 4, extending further laterad at apex; prosternal keel moderately broad, weakly convex, emarginate at base, carinal striae slightly convergent at middle, divergent anterad and posterad; prosternal lobe about one-half keel length, apical margin rounded, marginal stria complete; mesoventrite produced at middle, marginal stria complete or narrowly interrupted at middle; mesometaventral stria angulate forward at middle, fine, smooth, meeting inner lateral metaventral stria at mesocoxa, continuing obliquely posterad, curving toward outer corner of metacoxa, abbreviated apically, outer lateral metaventral stria short, present as postmesocoxal fragment; metaventral disk impunctate at middle; abdominal ventrite 1 with single, complete lateral stria, disk impunctate at middle, ventrites 2–5 finely, sparsely punctate across middle; protibia narrow, with three or four marginal denticles, margin serrulate between; mesofemur with posterior marginal stria not extending onto apical margin; mesotibia with two distinct marginal spines; outer metatibial margin smooth; propygidium lacking basal stria, with fine, sparse ground punctation and rather small secondary punctures separated by 1–1.5× their diameters, propygidial gland openings inconspicuous; pygidium with fine ground punctation, small secondary punctures present only along basal margin. Male genitalia (Figs 34A–H): T8 subquadrate, about as long as broad, sides subparallel, basal emargination shallow, basal rim well-sclerotized, apex very shallowly, narrowly emarginate, ventrolateral apodemes extending nearly to longitudinal midline, inner apices separated by about one-half T8 width; S8 divided, about as long as T8, inner margins approximate at base, divergent apically, outer margins weakly divergent, apical guides present, widened to near apex, narrowly rounded apically, apical velar membrane absent, apex with few inconspicuous setae; T9 with basal apodemes elongate, sinuous, nearly one-half total length, dorsal lobe of T9 elongate subtriangular, ventrolateral apodemes bluntly pointed at inner apices; S9 varied, may be completely divided, stem absent, with thin or absent basal bridge between the halves, apices divergent, with deep crease along lateral margin dividing distinct dorsal and ventral lobes; tegmen widest near base, evenly narrowed to apex, in lateral aspect rather thick throughout, abruptly bent ventrad in apical fourth; median lobe simple, about one-third tegmen length; basal piece about one-third tegmen length.

###### Remarks.

This is the most externally recognizable species in the *Baconia angusta* group. The oblique frontal carinae are unique (Fig. 32H), as are the slightly rounded body form (Fig. 32G) and the submarginal pronotal stria distinctly removed from the margin. The male genitalia of this species are perhaps relatively plesiomorphic in some respects, in having relatively well-developed apical guides on the 9^th^ tergite. The spiculum gastrale is nearly or fully divided, and has a distinctive apicolateral crease. We have limited the type series to specimens from the Guianas due in part to this genitalic variation.

###### Etymology.

This species is named for its distinctive frontal swellings.

##### Baconia cavei sp. n.

http://zoobank.org/86747A75-7C71-4F39-A924-61E404C0FC52

http://species-id.net/wiki/Baconia_cavei

Figs 34I–N 35A Map 9

###### Type locality.

Honduras: Cortés: Parque Nacional Cusuco [15.48°N, 88.22°W].

###### Type material.

**Holotype male**: “Honduras: Cortés, Parque Nacional Cusuco, 5 km N Buenos Aires, 15°29'N, 88°13'W, 30.VIII.1995, leg. R. Cave” / “Malaise trap in oak/pine cloud forest” / “Caterino/Tishechkin Exosternini Voucher EXO-00475” (FMNH).

###### Other material.

(2) **COSTA RICA**:1: **Heredia**: Est. Biol. La Selva, 10°26'N, 84°01'W, 50–150 m, 14.x.1994, *Pentaclethra macroloba* (INBI). 1: **Limón**: Sector Cerro Cocori, Finca de E. Rojas, 150 m, ix.1993, E. Rojas (INBI).

###### Diagnostic description.

Length: 1.7mm, width: 1.3mm; body elongate, parallel-sided, weakly depressed, glabrous; color rufescent, shining; head with frons swollen over antennal bases, slightly depressed at middle, interocular margins convergent dorsad, frontal disk smooth at center, few small punctures dorsad, frontal stria absent; antennal scape short, club rounded, rather large; epistoma flat, apex weakly emarginate; labrum about 2.5× wider than long, apical margin weakly arcuate; mandibles short, each with median tooth; pronotum with sides subparallel in basal half, weakly arcuately narrowed to apex, lateral marginal and lateral submarginal striae merging near anterior corner, submarginal stria very close to marginal throughout, continued around anterior margin, slightly removed from margin above head, crenulate; pronotal disk narrowly depressed along anterolateral margin, ground punctation fine, very sparse, with small, sparse secondary punctures present in lateral thirds, few along basal margin; elytra with single complete epipleural stria, outer and inner subhumeral striae absent, dorsal stria 1 shortened apically, may be fine and scratchlike beyond apical half, stria 2 nearly complete, striae 3-4 progressively apically abbreviated mediad, 4^th^ stria arched to meet base of sutural stria, 5^th^ stria absent, sutural stria obsolete in apical third, elytral disk with small, shallow secondary punctures in apical half, fewer towards sides; prosternal keel moderately broad, weakly convex between striae, emarginate at base, carinal striae subparallel throughout, diverging only slightly posterad; prosternal lobe about one-half keel length, apical margin rounded, marginal stria obsolete at sides; mesoventrite produced at middle, marginal stria complete; mesometaventral stria arched slightly forward, distinctly crenulate, meeting or nearly meeting anterior ends of inner lateral metaventral stria, which extends posterad toward inner third of metacoxa, outer lateral metaventral stria present as short, oblique postmesocoxal fragment; metaventral disk impunctate at middle; abdominal ventrite 1 with inner lateral stria slightly abbreviated, curved mediad apically, outer lateral stria absent, disk impunctate at middle, ventrites 2-5 finely punctate across middle; protibia narrow, with three weak marginal denticles, the distal pair relatively close together, margin serrulate between; mesofemur with posterior marginal stria weakly impressed around distal margin; mesotibia with two marginal spines, basalmost may be weak; outer metatibial margin smooth; propygidium lacking basal stria, ocellate secondary punctures sparse, slightly denser toward basal margin, propygidial gland openings inconspicuous; pygidium with fine ground punctation and very small, sparse secondary punctures. Male genitalia (Figs 34I–N): T8 broad, short, sides weakly convergent to apex, basal emargination very shallow, basal rim moderately well sclerotized, apex narrowly, acutely emarginate, ventrolateral apodemes small, inner apices separated by three-fourths T8 width; S8 divided, slightly longer than T8, inner margins approximate in basal third, divergent apically, outer margins diverging apically, apical guides closed, apical velar membrane absent, apex with few conspicuous setae; T9 with proximal apodemes short, about one-third total length, dorsal lobe large, subtriangular, narrowed to slightly curved, rounded apex, ventrolateral apodeme bluntly dentate beneath; S9 wide, shallow, stem absent, basal edge wide, distal arms divergent, with apices bent abruptly inward, inner basal margin with acute median tooth; tegmen moderately broad, sides rounded, widest basad middle, narrowed distally, apices narrowly rounded, tegmen in lateral aspect rather evenly rounded from base to apex; median lobe about two-thirds tegmen length; basal piece about one-third tegmen length.

**Figure 35.**
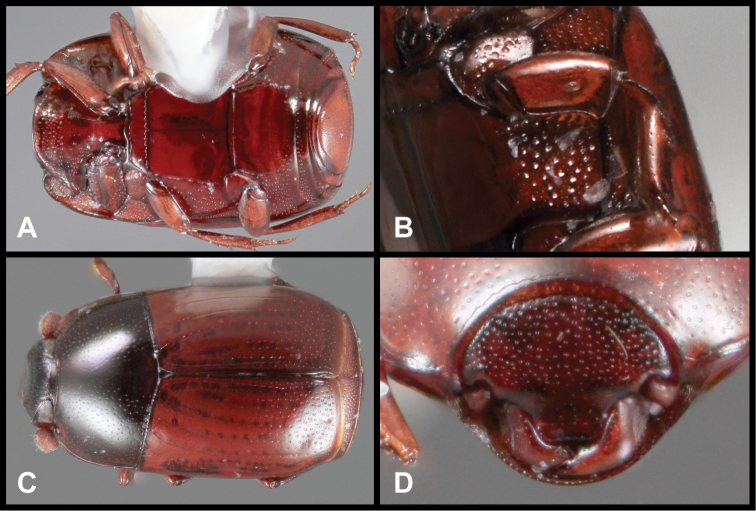
*Baconia angusta* group. **A** Ventral habitus of *Baconia cavei* **B** Mesofemur and tibia of *Baconia dentipes* **C** Dorsal habitus of *Baconia rubripennis* **D** Frons of *Baconia lunatifrons*.

###### Remarks.

Compared to other species in this group, *Baconia cavei* has a relatively broad prosternum (Fig. 35A), elongate, slender legs, and a large head. The male genitalia have the apices of the 8^th^ sternite narrowed and only finely setose, the aedeagus narrowed basally and apically, and the spiculum gastrale deeply apically emarginate, the entire sclerite more or less forming a ‘W’ shape.

There are some evident strial differences among all three available specimens, therefore we limit the type series to the single male from Honduras. Discovery of a male from Costa Rica may well support these differences as specific.

###### Etymology.

We are pleased to name this species for Dr. Ron Cave, collector of the holotype specimen.

##### Baconia subtilis sp. n.

http://zoobank.org/A0A307FE-DDD4-4DFC-BEC8-27CC4E9F65EF

http://species-id.net/wiki/Baconia_subtilis

**Figure 36.**
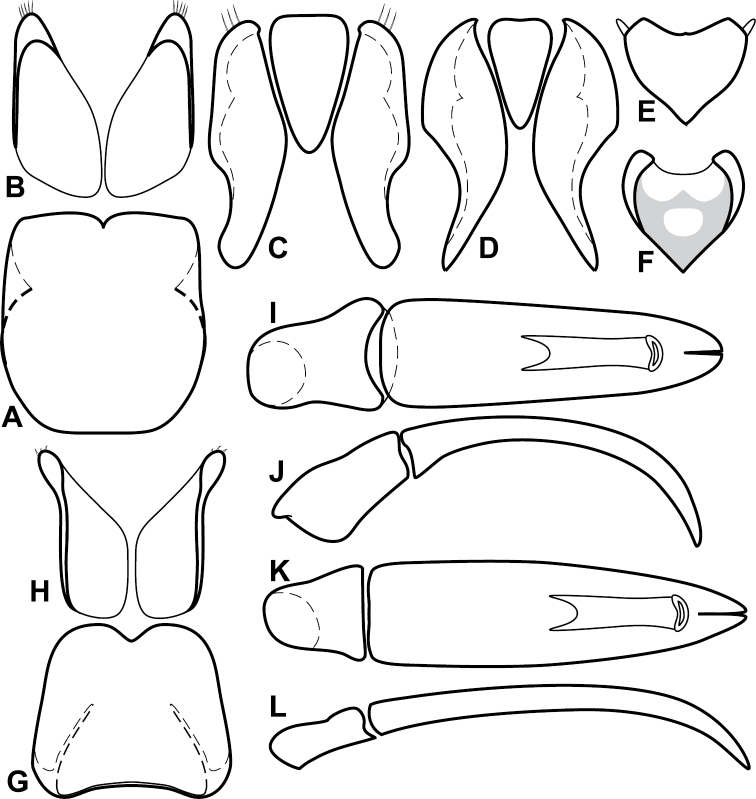
Male genitalia of *Baconia angusta* group. **A** T8 of *Baconia subtilis* **B** S8 of *Baconia subtilis* **C** T9 & T10 of *Baconia subtilis* **D** T9 & T10 of *Baconia rubripennis* **E** S9 of *Baconia subtilis* **F** S9 of *Baconia rubripennis* **G** T8 of *Baconia rubripennis* **H** S8 of *Baconia rubripennis* **I** Aedeagus, dorsal view of *Baconia subtilis* **J** Aedeagus, lateral view of *Baconia subtilis* **K **Aedeagus, dorsal view of *Baconia rubripennis* **L** Aedeagus, lateral view of *Baconia rubripennis*.

Figs 36A–C, E, I–J Map 9

###### Type locality.

FRENCH GUIANA: Belvèdére de Saül [3.01°N, 53.21°W].

###### Type material.

**Holotype male**: “**GUYANE FRANÇAISE**: Bélvédère de Saül, point de vue. 3°1'22"N, 53°12'34"W, Piège vitre 2, 7.ii.2011. SEAG leg.” / “Caterino/Tishechkin Exosternini Voucher EXO-01769” (MNHN). **Paratype** (1): same locality as type, 4.1.2011 (CHND).

###### Other material.

(1): **PERU: Loreto**, 45 km W Iquitos, Rio Nanay, Porvenir 3°54'S, 73°33'W. Terra firme, FIT. 19.vi.2011, G.Lamarre. P10 V5 TF (CHND).

###### Diagnostic description.

Length: 1.7–1.9mm, width: 1.1–1.2mm; body elongate, parallel-sided, weakly depressed, glabrous; color rufescent, shining; frons weakly swollen over antennal bases, slightly depressed at middle, interocular margins convergent dorsad, frontal disk smooth at center, ground punctation moderately conspicuous, few small secondary punctures dorsad, frontal stria absent; antennal scape short, club rounded, slightly wider toward apex; epistoma flat, apex weakly emarginate; labrum about 2.5× wider than long, apical margin weakly arcuate; mandibles short, each with median tooth; pronotum with sides subparallel in basal two-thirds, arcuate to apex, lateral marginal and submarginal striae merging behind anterior corner, submarginal stria very close to marginal along sides, continued anteriorly around anterior margin, slightly removed from margin above head, crenulate; pronotal disk narrowly depressed along anterolateral margin, ground punctation fine, very sparse, with small, sparse secondary punctures present in lateral thirds separated by 3× their widths; elytra with two more or less complete epipleural striae, the outer stria may be fragmented, dorsal stria 1 shortened apically, obsolete or fine and scratchlike in apical half, stria 2 nearly complete, striae 3–4 slightly shorter apically, 4^th^ stria arched to meet base of sutural stria, 5^th^ stria absent, sutural stria obsolete in apical third, elytral disk with small, shallow secondary punctures in apical third, fewer towards sides; prosternal keel weakly convex between striae, emarginate at base, carinal striae subparallel basally, diverging slightly anterad; prosternal lobe about two-thirds keel length, deflexed, apical margin rounded, marginal stria obsolete at sides; mesoventrite produced at middle, marginal stria complete; mesometaventral stria transverse, distinctly crenulate, continued by inner lateral metaventral stria posterad toward inner third of metacoxa, outer lateral metaventral stria present as short, oblique postmesocoxal fragment; metaventral disk impunctate at middle; abdominal ventrite 1 with inner lateral stria complete, outer lateral stria absent, disk impunctate at middle, ventrites 2-5 finely punctate across middle; protibia narrow, with three marginal denticles, the distal pair relatively close together, margin serrulate between; mesofemur with posterior marginal stria weakly impressed around distal margin; mesotibia with two marginal spines; outer metatibial margin smooth; propygidium lacking basal stria, ocellate secondary punctures small, sparse, separated by about 1.5× their diameters, propygidial gland openings inconspicuous; pygidium with fine ground punctation and small, sparse secondary punctures becoming finer but slightly denser toward apex. Male genitalia (Figs 36A–C, E, I–J): T8 slightly longer than broad, sides more or less parallel, base not emarginate, basal rim moderately well sclerotized, apex narrowly, acutely emarginate, ventrolateral apodemes small, inner apices widely separated beneath; S8 divided, slightly longer than T8, inner margins approximate in basal third, divergent apically, outer margins weakly diverging apically, apical guides closed, apical velar membrane absent, apex with several conspicuous setae; T9 with proximal apodemes thick, wide, nearly one-half total length, dorsal lobe large, broad, weakly narrowed apically, apex rounded, with blunt inner corner, ventrolateral apodeme very weakly developed; S9 cordate, stem absent, base acute, apicolateral corners bearing small ventral digitiform process, apical margin with small median denticle; tegmen widest at base, sides weakly convergent, apex narrowly rounded, tegmen in lateral aspect strongly curved from base to apex; median lobe about one-half tegmen length; basal piece about one-third tegmen length.

###### Remarks.

Externally there is little to distinguish this species from *Baconia guartela* (see Figs 32D–F), with a transverse, crenulate mesometaventral stria, relatively impunctate frons and pronotum, and somewhat longer elytral striae than typical for the group. However, the male 8^th^ sternite has the apices somewhat broadly rounded, with conspicuous apical setae, the 9^th^ tergite has very short, broad basal apodemes and apical lobes, also with several conspicuous apical setae, the spiculum gastrale is subtriangular, with small, ventrally directed, apicolateral processes. The aedeagus shows very pronounced dorsoventral curvature, but not the strong deflection of the basal piece seen in *Baconia guartela*. The specimen from Peru, a male, shows some slight genitalic differences, and is therefore excluded from the type series.

###### Etymology.

This species’ name celebrates the subtle differences among species of the *Baconia angusta* group.

##### Baconia dentipes sp. n.

http://zoobank.org/FC504766-3903-4F8B-8353-106E29153ED6

http://species-id.net/wiki/Baconia_dentipes

Fig 35B Map 9

###### Type locality.

PERU: Junín: ~1km N Satipo [11.24°S, 74.65°W].

###### Type material.

**Holotype female**: “**PERU: Depto. Junín**, ~1km N Satipo, Sector San Isidro. 11°14.51'S, 74°38.98'W 730m. Window trap at treefall, 11–12 April 2009. A.V.Petrov” / “Caterino/Tishechkin Exosternini Voucher EXO-00476” (FMNH).

###### Other material.

**BOLIVIA**: 1: **Cochabamba**: Cochabamba, 117 km E, Yungas nr. Rio Carmen Mayu (Cochabamba Villa Tunari Rd.), 17°6'32"S, 65°41'12"W, 1040 m, 6–8.ii.1999, FIT, R. Hanley (CMNC). **ECUADOR**:1: **Orellana**: Res. Ethnica Waorani, 1 km S Onkone Gare Camp, Trans. Ent., 0°39'10"S, 76°26'W, 220 m, 3.vii.1994, fogging, mostly bare green leaves, some with covering of lichenous or brophytic plants in terra firme forest, T. Erwin (USNM).

###### Diagnostic description.

Length: 1.6–1.8mm, width: 1.1–1.2mm; body elongate, parallel-sided, weakly depressed, glabrous; color rufescent, shining; head with frons elevated over antennal bases, slightly depressed at middle, interocular margins convergent dorsad, frontal punctation inconspicuous in anterior half, secondary punctures increasing dorsally, frontal stria absent, supraorbital stria vaguely indicated by serial punctures; antennal scape short, club rounded, slightly expanded apically; epistoma flat, apex truncate; labrum short, flat, about 3× wider than long, apical margin finely carinate, weakly arcuate; mandibles short, each with median tooth; pronotum with sides subparallel in basal half, rounded to apex, lateral marginal and submarginal striae merging near anterior corner, submarginal stria very close to marginal along side, continued anteriorly around anterior margin, slightly removed from margin above head; pronotal disk narrowly depressed along anterolateral margin, ground punctation fine, very sparse, with small, sparse secondary punctures present in lateral third; elytra with two more or less complete epipleural stria, the outermost may be fragmented or abbreviated, outer and inner subhumeral striae absent, dorsal stria 1 nearly complete, fine, scratchlike in apical half, striae 2–4 progressively more abbreviated apically, 4^th^ stria arched to meet base of sutural stria, 5^th^ stria absent, sutural stria obsolete in apical fourth, elytral disk with small, shallow secondary punctures in apical third, diminishing in size and density anteromedially; prosternal keel narrow, convex between striae, emarginate at base, carinal striae subparallel in basal half, diverging slightly anterad; prosternal lobe about two-thirds keel length, apical margin broadly rounded, marginal stria obsolete at sides; mesoventrite produced at middle, marginal stria complete; mesometaventral stria transverse, distinctly crenulate, continued by inner lateral metaventral stria posterad toward inner third of metacoxa, outer lateral metaventral stria short, just an oblique postmesocoxal fragment; metaventral disk impunctate at middle; abdominal ventrite 1 with inner lateral stria complete, curved mediad apically, outer lateral stria absent, disk impunctate at middle, ventrites 2-5 finely punctate across middle; protibia narrow, with four weak marginal denticles more or less evenly spaced, margin serrulate between; mesofemur weakly dentate at posterolateral corner, posterior marginal stria following margin, well impressed along distal margin; mesotibia with moderately distinct basal bend, two distinct marginal spines; outer metatibial margin smooth; propygidium lacking basal stria, with ocellate secondary punctures separated by slightly more than their diameters, propygidial gland openings present nearly one-half behind anterior margin; pygidium with fine ground punctation, small secondary punctures sparser toward apex. Male: not known.

###### Remarks.

Although this species is known from only female specimens, its dentate apices of the mesofemur (Fig. 35B) allow it to be easily separated from related species. In addition its frons is quite convex compared to other species in the *Baconia angusta* group. Given the importance of male genitalia in the group, we refrain from designating females from other localities as paratypes, as males may reveal more significant differences.

###### Etymology.

The name of this species refers to the dentate apices of the mesofemur.

##### Baconia rubripennis sp. n.

http://zoobank.org/13BD2FCF-EBA7-4F6A-8909-B71647AA62E9

http://species-id.net/wiki/Baconia_rubripennis

Figs 35A 36D, F–H, K–L Map 9

###### Type locality.

ECUADOR: Orellana:Res. Ethnica Waorani [0.67°N, 76.43°W].

###### Type material.

**Holotype male**: “**ECUADOR: Depto. Orellana:** Res. Ethnica Waorani, 1km S Onkone Gare Camp, Trans. Ent., 0°39'26"S, 76°27'11"W, 216m, 23 January 2006, T.L. Erwin, M.C.Pimienta et al.” / “Insecticidal fogging of mostly bare green leaves, some with covering of lichenous or bryophytic plants in terra firme forest. Project MAXUS **Lot 3184 Trans. 9 Sta. 5**” / “Caterino/Tishechkin Exosternini Voucher EXO-00474” (USNM). **Paratypes** (2): same locality as type except 0°39'10"S, 76°26'11"W, 220m, 1: 23.vi.1996, fogging, T. Erwin (USNM), 1: 8.x.1995, fogging, T. Erwin (USNM).

###### Diagnostic description.

Length: 1.5–1.7mm, width: 0.9–1.1mm; body elongate, parallel-sided, weakly depressed, glabrous; subtly bicolored, head and pronotum rufobrunneus, elytra and most of venter rufescent, shining; head with frons weakly elevated over antennal bases, more or less flat at middle, interocular margins convergent dorsad, frontal punctation rather sparse, finer and sparser anterad, frontal stria absent, supraorbital stria vaguely indicated by serial punctures; antennal scape short, club rounded, slightly expanded apically; epistoma flat, apex truncate; labrum short, flat, about 4× wider than long, apical margin finely carinate, faintly emarginate; mandibles short, each with median tooth; pronotum with sides subparallel in basal two-thirds, rounded to apex, lateral marginal and submarginal striae merging very close to anterior corner, submarginal stria very close to marginal throughout, continued anteriorly around anterior margin; pronotal disk narrowly depressed along anterolateral margin, ground punctation fine, very sparse, with small secondary punctures nearly throughout separated by 2–3× their widths, with narrow, rather discrete impunctate band along midline; elytra with two more or less complete epipleural striae, the outermost may be faintly abbreviated at apices, outer and inner subhumeral striae absent, dorsal stria 1 nearly complete, striae 2–4 progressively more abbreviated apically, 4^th^ stria arched to meet base of sutural stria, 5^th^ stria absent, sutural stria obsolete in apical fourth, elytral disk with small, shallow secondary punctures in apical third, diminished in size and density anteromedially; prosternal keel narrow, flat, emarginate at base, carinal striae convergent at middle, diverging slightly at apices; prosternal lobe about two-thirds keel length, apical margin rounded, marginal stria obsolete at sides; mesoventrite produced at middle, marginal stria complete; mesometaventral stria transverse, distinctly crenulate, continued by inner lateral metaventral stria posterad toward inner third of metacoxa, outer lateral metaventral stria short, no more than oblique postmesocoxal fragment; metaventral disk impunctate at middle; abdominal ventrite 1 with inner lateral stria abbreviated posteriorly, outer lateral stria represented by short postcoxal fragment, disk impunctate at middle, ventrites 2–5 finely, rather sparsely punctate across middle; protibia narrow, with three distinct marginal denticles plus one small basal marginal tooth, margin serrulate between; mesofemur with posterior marginal stria weakly curving anterad along apical margin; mesotibia with two distinct marginal spines; outer metatibial margin smooth; propygidium lacking basal stria, with moderately dense, ocellate secondary punctures separated by slightly less than their diameters, propygidial gland openings present about one-third behind anterior margin; pygidium with fine ground punctation rather dense, small secondary punctures evenly scattered, slightly smaller but denser toward apex. Male genitalia (Figs 36D, F–H, K–L): T8 slightly longer than broad, sides weakly rounded, converging to apex, base very shallowly emarginate, basal rim moderately well sclerotized, apex narrowly, acutely emarginate, ventrolateral apodemes well developed, inner apices separated by about one-third width beneath; S8 divided, similar in length to T8, inner margins approximate in basal half, divergent apically, outer margins diverging apically, apical guides closed, apices narrow, with a few very fine, inconspicuous setae; T9 with proximal apodemes nearly one-half total length, dorsal lobe large, broad, narrowed apically to narrowly rounded apices, ventrolateral apodeme weakly dentate; S9 cordate, stem absent, base faintly acute, apicolateral corners rounded, apex moderately desclerotized; tegmen widest just basad middle, sides weakly curved, narrowed to apex, tegmen in lateral aspect strongly increasingly curved toward apex; median lobe about one-half tegmen length; basal piece about one-third tegmen length.

###### Remarks.

This species can be fairly readily recognized by the contrasting colors of the darker pronotum and rufescent elytra (Fig. 35C). In addition it is unusual in having secondary punctures over most of pronotum. Male genitalia have the apices of the 8^th^ sternite narrow and strongly divergent, and the spiculum gastrale distinctly cordate in shape, with its apicolateral margins well sclerotized.

###### Etymology.

This species’ name refers to the reddish elytra, which give it a bicolored appearance.

##### Baconia lunatifrons sp. n.

http://zoobank.org/DB7C238A-B765-49AC-BCA1-1F3C66C058DF

http://species-id.net/wiki/Baconia_lunatifrons

Figs 35D 37 Map 9

###### Type locality.

SURINAME: Para: 11km SE Zanderij Airport [5.39°N, 55.27°W].

###### Type material.

**Holotype male**: “**SURINAM**: Para, 11km SE Zanderij Airport, 30m, FIT, 20.VI.1999,Z.Falin” / “Caterino/Tishechkin Exosternini Voucher EXO-00473” (CMNC).

###### Diagnostic description.

**Figure 37.**
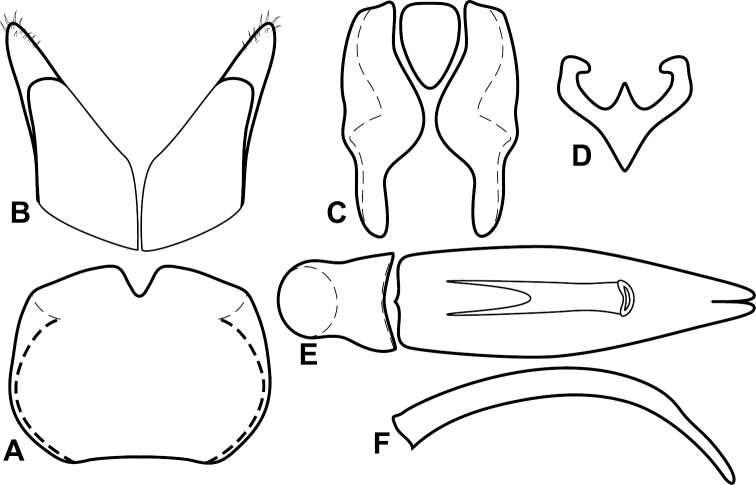
Male genitalia of *Baconia lunatifrons*. **A** T8 **B** S8 **C** T9 & T10 **D** S9 **E** Aedeagus, dorsal view **F** Aedeagus, lateral view.

Length: 1.8mm, width: 1.1mm; body elongate, parallel-sided, weakly depressed, glabrous; color rufescent, shining; head with frons rather broad, weakly elevated over antennal bases, more or less flat across middle, interocular margins convergent dorsad, frontal punctures small, uniform, rather dense above antennal bases, frontal stria absent; antennal scape short, club rounded; epistoma flat, apex truncate; labrum short, flat, about 4× wider than long, apical margin weakly emarginate; mandibles short, each with median tooth; pronotum with sides subparallel in basal half, rounded to apex, lateral marginal and submarginal striae very nearly merging near anterior corner, submarginal stria close to marginal at sides, continued anteriorly around anterior margin, slightly removed from margin anteriorly, coarsely crenulate above head; pronotal disk narrowly depressed along anterolateral margin, ground punctation fine, very sparse, with small secondary punctures sparsely scattered in lateral thirds; elytra with two epipleural striae, the outermost may be fragmented or abbreviated, outer and inner subhumeral striae absent, dorsal stria 1 nearly complete, fine, scratchlike in apical fourth, stria 2 nearly complete, striae 3–4 progressively more abbreviated apically, 4^th^ stria arched to meet base of sutural stria, 5^th^ stria absent, sutural stria obsolete in apical fourth, elytral disk with small, shallow secondary punctures in apical third; prosternal keel narrow, weakly convex between striae, slightly pinched at middle, narrowly emarginate at base, carinal striae convergent to middle; prosternal lobe about two-thirds keel length, apex weakly deflexed, apical margin broadly rounded, marginal stria obsolete at sides; mesoventrite subacutely produced at middle, marginal stria complete; mesometaventral stria weakly arched forward, distinctly crenulate, barely detached from inner lateral metaventral stria, which continues posterad toward inner third of metacoxa, outer lateral metaventral stria short, present as an oblique postmesocoxal fragment; metaventral disk impunctate at middle; abdominal ventrite 1 with inner lateral stria complete, outer lateral stria short, present only behind coxa, disk impunctate at middle, ventrites 2–5 finely punctate across middle; protibia narrow, with four weak marginal denticles, the middle pair more widely separated, margin serrulate between; mesofemur narrowing apically, posterior marginal stria following margin, weakly impressed along distal margin; mesotibia with two distinct marginal spines; outer metatibial margin smooth; propygidium lacking basal stria, with ocellate secondary punctures separated by slightly more than their diameters, smaller and denser anteriorly, propygidial gland openings inconspicuous; pygidium with fine ground punctation and very small, sparse secondary punctures throughout. Male genitalia (Fig. 37): T8 broad, short, sides weakly convergent to apex, basal emargination very shallow, basal rim moderately well sclerotized, apex narrowly, acutely emarginate, ventrolateral apodemes small, inner apices separated by three-fourths T8 width; S8 divided, slightly longer than T8, inner margins approximate in basal third, divergent apically, outer margins diverging apically, apical guides closed, apical velar membrane absent, apex with several conspicuous setae; T9 with proximal apodemes short, rather broad, about one-third total length, dorsal lobe large, subquadrate, only weakly narrowed to broad, truncate apex, ventrolateral apodeme bluntly dentate beneath; S9 wide, shallow, stem absent, basal edge acuminate, distal arms divergent, with apices bent abruptly inward, inner margin with acute median tooth; tegmen moderately broad, sides rounded, widest basad middle, narrowed distally, apices narrowly rounded, tegmen in lateral aspect rather strongly curved ventrad, distinctly and abruptly flatter in apical fourth; median lobe about two-thirds tegmen length; basal piece about one-third tegmen length.

###### Remarks.

There are few external characters to distinguish *Baconia lunatifrons* from either *Baconia guartela* or *Baconia subtilis*, although the broad, evenly and finely punctate frons (Fig. 35D) is weakly distinctive. Like *Baconia subtilis*, the male 9^th^ tergite has short, broad proximal apodemes, and broadly subtruncate apices. However, its spiculum gastrale is much more like that of *Baconia cavei*, being deeply emarginate apically, with a strong median projection. The aedeagus is strongly curved dorsoventrally, and the apical third is distinctly compressed dorsoventrally.

###### Etymology.

This species’ name refers to its large, semicircular frons, lacking marginal striae.

#### *Baconia aeneomicans*group

The *Baconia aeneomicans* group is a group of 27 species that largely exhibit characters associated with the subgenus *Binhister*. Although this name has mainly been applied to the eastern Asian *Baconia chujoi* (Cooman) and *Baconia barbarus* (Cooman) (and the unrelated *Baconia burmeisteri* Marseul), these species share many apomorphies with a large group of American species. The strongest characters uniting this group are found in the male genitalia, in which the halves of the 8^th^ sternite are fused into a single sclerite, which exhibits a rather well-defined apicoventral concavity, and in which the apical lobes bear a distinct setal fringe. Additionally, the proximal apodemes of the 9^th^ tergite are generally very short, the apical lobes of the 9^th^ tergite bear a single subapical seta, and the spiculum gastrale (S9) has a broad head and narrow stem that in many species bears a median keel. Externally the species are also rather distinctive. The body is usually relatively small and elongate, moderately to strongly flattened, usually rufo-piceous to piceous, only rarely metallic in coloration. The lateral submarginal pronotal stria is usually present and complete, elytral striae 4 and 5 arch toward the suture at their bases, and the gland openings of the propygidium in most species are distant from the anterior margin, generally near the midline of the propygidial disk.

These species are closely related to (and perhaps paraphyletic with respect to) a couple of other species groups, including the *Baconia angusta* group, as discussed above, and the *Baconia cylindrica* and *Baconia gibbifer* groups that follow. The *Baconia angusta* group species have the halves of the 8^th^ sternite separate, and are clearly outside the other three groups, but the apical setae of S8 and the short proximal apodemes, as well as the arched 4^th^ and 5^th^ elytral striae, strongly suggest a relationship. Both the subsequent species groups, however, share the fused 8^th^ sternite and most other male genitalic characters, and are mainly recognized with separate species group status for identification convenience – externally they are easily diagnosed. The *Baconia gibbifer* group, in particular, shares a character with most species within the *Baconia aeneomicans* group, eversible subapical denticles on the aedeagus (Fig. 41O), which pretty clearly indicate derivation from within. These possible relationships are discussed in greater detail below.

##### Baconia aeneomicans

(Horn, 1873)

http://species-id.net/wiki/Baconia_aeneomicans

**Figure 38.**
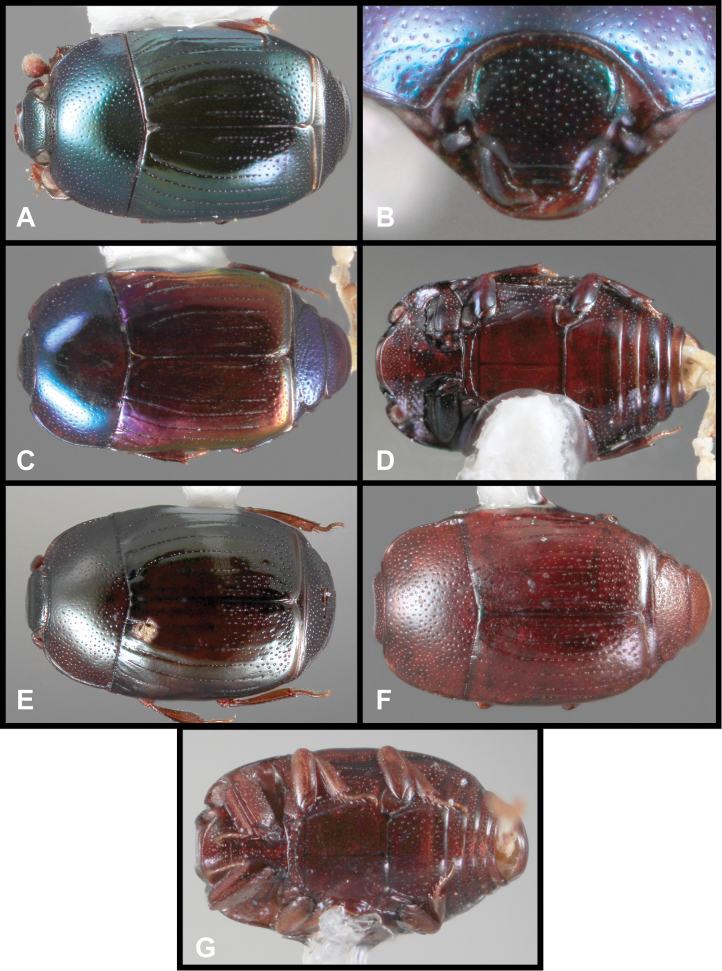
*Baconia aeneomicans* group. **A** Dorsal habitus of *Baconia aeneomicans* **B** Frons of *Baconia pulchella* **C **Dorsal habitus of *Baconia pulchella* **D** Ventral habitus of *Baconia pulchella* **E** Dorsal habitus of *Baconia quercea* **F** Dorsal habitus of *Baconia stephani* **G** Ventral habitus of *Baconia stephani*.

**Map 10.**
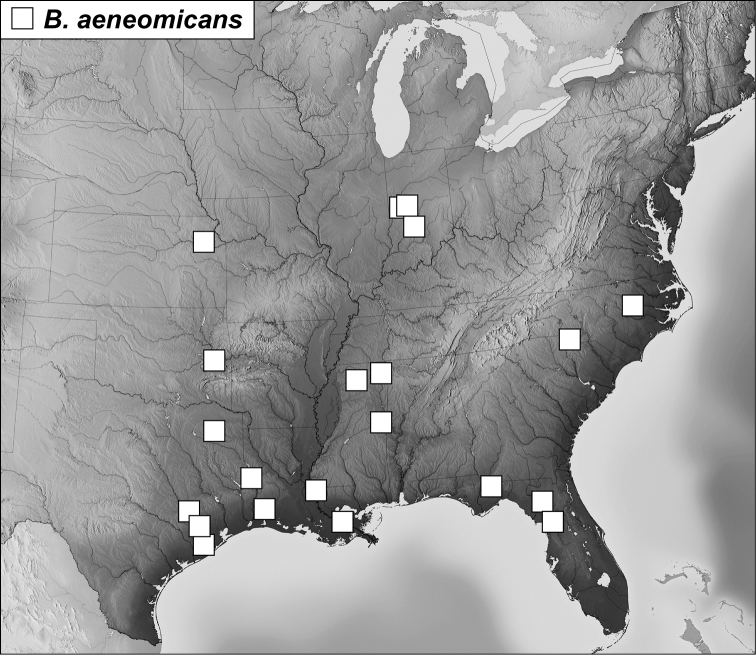
*Baconia aeneomicans* records.

Figs 38A 39A–C, E, I–J Map 10

Hister aeneomicans Horn, 1873: 295; *Phelister aeneomicans*: Schmidt 1884: 149; *Baconia aeneomicans*: Mazur 1984: 280.

###### Type locality.

UNITED STATES: District of Columbia [exact locality uncertain].

###### Type material.

**Neotype**, here designated (CMNH): “D.C.” / “Henry Ulke Beetle Coll. CMNH Acc. No. 1645”/”NEOTYPE *Hister aeneomicans* Horn, 1873, Desg. Caterino & Tishechkin, 2012”. This species was originally described by Horn from “one specimen in the cabinet of Mr. Ulke, collected in the District of Columbia” (Horn, 1873: 295). Therefore there was a unique holotype at one time. The specimen was returned to Ulke, whose collection now resides in the Carnegie Museum, and mixed with other specimens with identical data. Therefore the holotype is technically lost (though probably still in existence), and a Neotype is needed. We select one of three possible specimens with identical data to the original type, the most well preserved and intact. We have not dissected this specimen to confirm sex. However, this species is morphologically uniform throughout its range and there are unlikely to be any ambiguities with its identification in the future.

###### Other material.

**USA**: 2: **Florida**: Leon Co., Tallahassee, 23.v.1986, FIT, H. & A. Howden (MHNG); 1: Columbia Co., O’Leno SP, 10.v.1984, M.C. Thomas (FSCA); Marion Co., Ocala, 18.vii.1977, M.C. Thomas, in fungus on log (FSCA); 1: **Georgia**: Baker Co., Bethany Church, 31°18.5'N, 84°36'W, 28.xii.2003, forest litter, C. W. O’Brien & R. J. Turnbow (LSAM); 1: **Indiana**: Parke Co., 20.vii.1962, N. Downie (FMNH); 1: Tippecanoe Co., 7.ix.1958, N. Downie (FMNH); 1: Monroe Co., Bloomington, 31.v–1.vi.1986, blacklight trap, F.N. Young (FSCA); 2: **Kansas**: Jefferson Co., 1.5 km N jct. 94th St. & Kingman Rd., 39°13.38'N, 95°24.24'W, 10–20.vi.2005, FIT, near lower meadow, Z. Falin (SEMC); 2: 19–24.v.2005, canopy FIT, meadow, Z. Falin (SEMC); 1: 27.vi–3.vii.2005, FIT, meadow, Z. Falin (SEMC); 3: 29.v–7.vi.2005, FIT, meadow, Z. Falin (SEMC); 3: **Louisiana**: Calcasieu Par., Sam Houston Jones State Park, 30°18'N, 93°16'W, 2–31.vii.2002, FIT, A. Cline & A. Tishechkin (LSAM); 5: 31.vii–17.ix.2002, FIT, A. Cline & A. Tishechkin (LSAM); 4: 9.iii–16.v.2003, FIT, A. Cline & A. Tishechkin (AKTC, LSAM); 4: Catahoula Par., Sicily Island W. M. A., 30°49.5'N, 91°26'W, 10.i.2001, under bark, A. Cline & A. Tishechkin (LSAM); 1: East Baton Rouge Par., Baton Rouge, 12.ii.1982, under bark, S. M. Strother (LSAM); 1: Grant Par., 3 km SW Pollock, 15.v.3.vi.1985, FIT, H. & A. Howden & C. Scholtz (LSAM); 1: Jefferson Par., Harahan, 18.iv.1944, on small brown polypore fungus (FMNH); 1: W Feliciana Par., Feliciana Pres., nr. Freeland, 30°47'N, 91°15'W, 25.ii–2.iii.1996, FIT, C. Carlton (LSAM); 3: 14–30.iii.1996, FIT, C. Carlton (AKTC, LSAM); 1: 18.x.1997, pines, under bark, A. Tishechkin (AKTC); 1: 10–21.x.2000, FIT, A. Cline (LSAM); 5: 1–29.iv.2001, FIT, A. Cline (LSAM); 1: 24.v–2.vi.2001, FIT, A. Cline (LSAM); 1: 28.vi-29.vii.2002, FIT, A. Cline & A. Tishechkin (LSAM); 1: 14–24.iv.2003, FIT, A. Tishechkin (LSAM); 2: 24.iv–11.v.2003, FIT, A. Tishechkin (LSAM); 2: 11.v–4.vii.2003, FIT, A. Tishechkin (LSAM); 1: 27.iv–22.v.2005, FIT, A. Tishechkin & S. Gil (AKTC); 29.v-12.vi.2005, FIT, A. Tishechkin & S. Gil (AKTC); 1: 30.iii–9.iv.2005, FIT, A. Tishechkin (AKTC); 1: 12–30.vi.2005, FIT, A. Tishechkin & S. Gil (AKTC); 1: W Feliciana Par., Tunica Hills W. M. A., 30°55'N, 91°30'W, 14–30.x.1998, FIT, C. Carlton & A. Tishechkin (LSAM); 1: 6–28.xi.1998, FIT, C. Carlton & A. Tishechkin (AKTC); 1: **Mississippi**: Tishomingo Co., 5 mi S of Burnsville T4S, R9E, Sec.2SE, 23.iv.1998, 30.iv.1998, Lindgren funnel baited with Frontalin & Loblolly Pine Turpentine, R. Tisdale (TAMU); 1: Lafayette Co., 7 mi. NW Oxford, 13.i.1986, P. Lago (FMNH); 2: Noxubee Co., N.W.R. Big Tree Trail, 33°17'17"N, 88°46'4'W, 50 m, 18–26.vi.2001, FIT, R. Brooks & T. Peterson (SEMC); 1: **North Carolina**: 1: Haywood Co., Great Smoky Mountains National Park, Purchase Knob (top), 1510 m, 35°35'4"N, 83°3'45"W, 20.vii–12.viii.2001, FIT, C. E. Carlton (LSAM); Mecklenburg Co, Charlotte McDowell Nat. Pres., 35.08260°N, 81.013018°W, 650 ft, 11–18.vi.2010, UVL Trap, J. Cornell; 1: Wake Co., Raleigh, 1.xi.1964, Polyporus, J. Cornell (TAMU); 1: **Oklahoma**: Latimer Co., SW of Red Oak, vi.1981, K. Stephan (TAMU), 1: xii.1981, 1: vi.1982, 1: xi.1982, 2: v.1983, 9: v.1983, 1: v.1984, tree hole oak + rodent, 1: v.1984, FIT, 1: v.1984, 1: vii.1984, FIT, 1: iv.1984, 2: ix.1984, 1: iv.1985, 1: v.1985, 1: iii.1986, 2: v.1986, 1: vii.1988, 1: v.1990, 2: iv.1991, 1: v.1991, 3: vi.1992, 4: v.1993, 5: vi.1993, 1: viii.1993, 1: iv.1994, 1: v.1994, 4: v.1995, 4: vi.1995, 1: vii.1995, 4: v.1997, forest litter/FIT, 1: vi.1998 (all K. Stephan, TAMU, AKTC, FSCA); 1: **Texas**: Fort Bend Co., Brazos Bend St. Pk., 29.v–18.vi.1999, FIT, buckeye-sycamore forest, B. Raber & E. Riley (TAMU); 1: Tyler Co., Kirby State Forest, 30°34'30"N, 94°25'03"W, 22.vi–20.vii.2003, Lindgren funnel trap, E. Riley (AKTC); 3: Brazos Co., Lick Ck. Pk., College Station, 13.iv.1996, Berlese forest litter, E. Riley (TAMU, LSAM, AKTC); 1: 13–6.iv.1996, post oak-yaupon, upland forest, E. Riley (AKTC); 1: 13.iv.1996, Berlese forest litter, E. Riley (TAMU); 4: 13.iv.1996, post oak-yaupon, upland forest, E. Riley (TAMU, AKTC); 1: 25.ix–3.i.1998, elevated FIT, bottomland forest, E. Riley (AKTC); 1: 3–11.v.1996, FIT, bottomland forest, E. Riley (AKTC); 1: 30.vi.1996, E. Riley (TAMU); 1: 9–22.iii.1997, elevated FIT, bottomland forest, E. Riley (TAMU); 5: Montgomery Co., 17.2 km N Montgomery, 17.v–17.vi.1987, FIT, R. Anderson; 1: Wood Co., 5 mi. S Hawkins at Jct Hwy 14 & FM 2869, 6.vii–18.viii.1996, FIT, W. Godwin (TAMU); 1: Sabine Co., 9 mi. E Hemphill, 5–17.vi.1989, FIT, beech-magnolia forest, R. Anderson & E. Morris (AKTC); 1: Sabine Co., Mill Creek Cove, 8.8 mi. NE Hemphill, 31.3851°N, 93.7090°W, 16.iii–30.iii.2008, FIT-elevated, Beech Bottom, E. Riley (AKTC); 1: 25.iv–7.v.1989, FIT, R. Anderson & E. Morris (TAMU); 1: 31.iii–13.iv.2008, FIT, Beech Bottom, E. Riley (AKTC); 1: 5–17.vi.1989, FIT, beech-magnolia forest, R. Anderson & E. Morris (TAMU).

**Figure 39.**
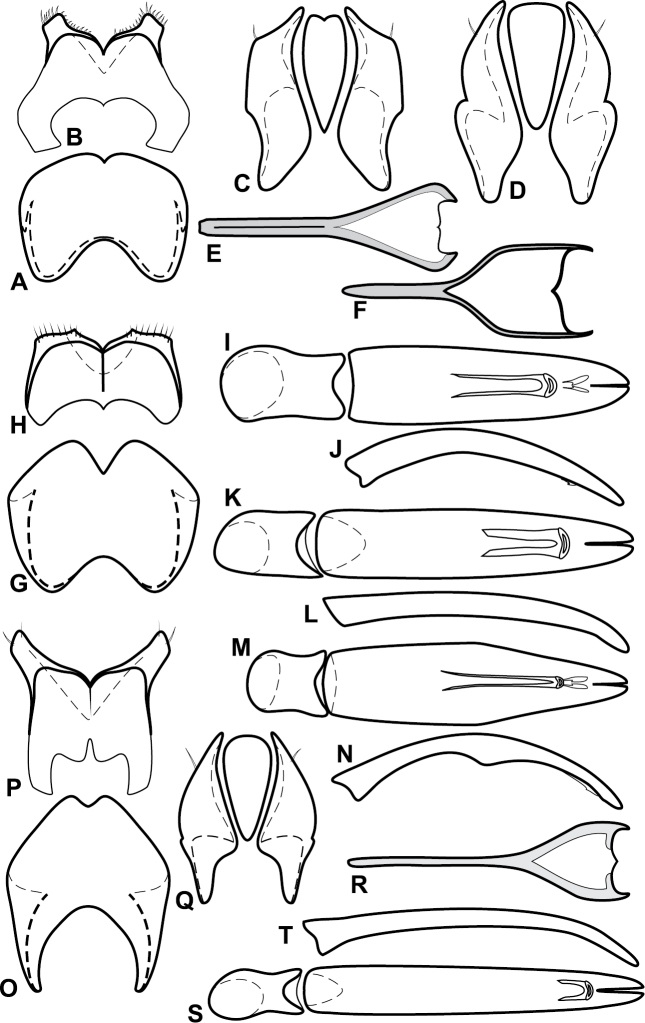
Male genitalia of *Baconia aeneomicans* group. **A** T8 of *Baconia aeneomicans* **B** S8 of *Baconia aeneomicans* **C** T9 & T10 of *Baconia aeneomicans* **D** T9 & T10 of *Baconia quercea* **E** S9 of *Baconia aeneomicans* **F** S9 of *Baconia quercea* **G **T8 of *Baconia quercea* **H** S8 of *Baconia quercea* **I** Aedeagus, dorsal view of *Baconia aeneomicans* **J** Aedeagus, lateral view of *Baconia aeneomicans* **K** Aedeagus, dorsal view of *Baconia quercea* **L** Aedeagus, lateral view of *Baconia quercea* **M** Aedeagus, dorsal view of *Baconia stephani* **N** Aedeagus, lateral view of *Baconia stephani* **O** T8 of *Baconia irinae* **N** S8 of *Baconia irinae* **O** T9 & T10 of *Baconia irinae* **R** S9 of *Baconia irinae* **S** Aedeagus, dorsal view of *Baconia irinae* **T** Aedeagus, lateral view of *Baconia irinae*.

###### Diagnostic description.

Length: 1.4–1.7mm, width: 1.2–1.3mm; body elongate oval, subdepressed, glabrous; color metallic-blue over most of dorsum, elytra grading to green-blue in anterior half, venter rufobrunneus with faint metallic sheen; head with frons flat, ground punctation fine, sparse anteriorly, slightly coarser dorsad, frontal stria present only along inner margin of eye, obsolete across middle, supraorbital stria absent or represented by few fragments; antennal scape short, club nearly circular; epistoma faintly emarginate apically; labrum about 3×wider than long, weakly emarginate apically; both mandibles with strong, acute basal tooth, mesal secretory channel present; pronotum with sides subparallel in basal half, evenly rounded to apex, lateral marginal stria descending to ventral edge in posterior half, continuous anteriorly with complete anterior marginal stria, lateral submarginal stria present in basal three-fourths, pronotal disk with fine ground punctation, conspicuous secondary punctures interspersed more or less throughout, denser toward sides; elytra with one to two complete epipleural striae, outer subhumeral stria absent, inner present in basal one-third or less, dorsal striae 1-4 complete to base, progressively abbreviated apically, 4^th^ stria arched mediad in front, stria 5 slightly abbreviated at base and apex, sutural stria present in middle one-third, abbreviated at base and apex, elytral disk with scattered secondary punctures in apical one-third; prosternum moderately broad, keel weakly emarginate at base, with carinal striae complete, separate, subparallel in basal one-fourth, weakly divergent anterad, with few punctures between; prosternal lobe slightly over one-half keel length, apical margin bluntly rounded, with marginal stria present along middle; mesoventrite weakly produced at middle, with marginal stria absent from anterior margin, present only at anterolateral corners; mesometaventral suture weakly arched forward, mesometaventral stria arched more strongly forward, rounded to subangulate at middle, continuous laterally with oblique lateral metaventral stria; metaventral disk coarsely punctate at sides, impunctate at middle; abdominal ventrite 1 with single, complete lateral stria, disk with coarse secondary punctures only laterad stria, ventrites 2–5 with sparse secondary punctures at sides, the punctures becoming almost obsolete at middle; protibia tridentate, the middle tooth closer to apical than basal, outer margin serrulate between teeth; mesotibia with two very weak marginal spines; outer metatibial margin smooth, edentate; propygidium lacking basal stria, with sparse, fine ground punctation, with coarse secondary punctures evenly interspersed, propygidial gland openings rather conspicuous, located about one-third behind anterior margin, about one-fourth width from each lateral margin, disk impunctate in immediate vicinity; pygidium with sparse ground punctation and only moderately coarser, secondary punctation denser toward base. Male genitalia (Figs 39A–C, E, I–J): T8 narrowly, rather deeply emarginate, ventrolateral apodemes with inner apices widely separated, projecting beyond ventral midpoint, obsolete apically, apical margin shallowly emarginate; S8 with halves fused along midline, basal emargination broad, deep, basal apodemes widely separated, sides sinuately narrowed, apices obliquely truncate, densely setose, widely separated by acute apical emargination; T9 with short basal apodemes, halves nearly meeting dorsobasally, ventrolateral apodemes bluntly produced beneath, nearly to midline, apices narrowly rounded, with single seta borne on subapical tubercle on each side; T10 narrow, finely emarginate apically; S9 with long narrow, medially keeled stem, head rounded to near apex, apices subacute, apical emargination broad, sinuate; tegmen with base rather broad, weakly narrowed to apex, apices narrowly rounded, tegmen strongly curved dorsoventrally, mainly near base, with eversible subapical denticles; median lobe about one-third tegmen length, simple; basal piece about one-third tegmen length.

###### Remarks.

*Baconia aeneomicans* is among the very few metallic species in this species group (Fig. 38A), and this in combination with its distribution makes its identification straightforward. It is sympatric with only two other North American *Baconia* species, *Baconia venusta*, which is much larger, with a larger, more convex, less distinctly punctate body (Figs 11C–F; in addition to lacking all the characters of the *Baconia aeneomicans* group in general), and *Baconia stephani* sp. n., described below. The latter is rather similar, but may be easily separated by its non-metallic coloration, more narrowly elongate body, and more coarsely and completely punctate pronotum.

##### Baconia pulchella sp. n.

http://zoobank.org/3DBB22C5-566E-4BE7-A2D4-51FFDE38FD04

http://species-id.net/wiki/Baconia_pulchella

Figs 38B–D Map 11

###### Type locality.

CUBA: Santiago: Jardin Botanico [20.0°N, 75.8°W].

###### Type material.

**Holotype female**: “CUBA, Santiago Prov. Santiago, Jardin Botanico, 5–17.XII.1995, 5m, disturb for. FITs, S.Peck, 95-74” / “Caterino/Tishechkin Exosternini Voucher EXO-00449” (CMNC).

###### Diagnostic description.

Length: 1.5mm, width: 1.0mm; body elongate, parallel-sided, moderately depressed, glabrous; color metallic, pronotum, head and pygidium blue, contrasting distinctly with violaceous elytra, venter faintly blue; frons faintly depressed at middle, ground punctation conspicuous with few larger punctures interspersed at middle, particularly dorsad; frontal stria broadly interrupted between antennal bases, present along inner edges of eyes; epistoma weakly elevated along apical margin, truncate; labrum about 4×wider than long, apical margin very faintly emarginate; mandibles short, each with conspicuous, acute basal tooth; pronotum with sides subparallel in basal half, narrowed arcuately to apex, lateral and submarginal striae merging behind anterior corner, continued along anterior margin; pronotal disk weakly depressed in anterolateral corners, with ground punctures sparsely impressed throughout, with coarser secondary punctures interspersed in lateral thirds; elytra with two more or less complete epipleural striae, the outer slightly fragmented at middle, outer subhumeral stria absent, short fragment of inner subhumeral present at base, striae 1–4 complete, the 4^th^ bent mediad at base, 5^th^ stria present in apical two-thirds, sutural stria obsolete in basal half, elytral disk with small, sparse punctures restricted to apical sixth, beyond apices of striae; prosternal keel moderately broad, very weakly emarginate at base, carinal striae convergent near basal third, diverging anterad and posterad, slightly abbreviated anteriorly; prosternal lobe about two-thirds keel length, marginal stria obsolete at sides; mesoventrite weakly produced at middle, marginal stria reduced to few lateral punctures; mesometaventral stria broadly arched forward, weakly crenulate, continuous at sides with inner lateral metaventral stria, which extends obliquely posterad toward outer third of metacoxa, outer lateral metaventral stria absent; metaventral disk impunctate at middle; abdominal ventrite 1 with single, complete lateral stria, ventrites 2–5 with sparse punctures at sides, nearly impunctate across middle; protibia unevenly 4-dentate, the middle pair more widely separated, the outer margin finely serrulate between denticles; mesotibia with two marginal spines; outer metatibial margin smooth; propygidium lacking basal stria, ocellate punctures scattered more or less uniformly, larger and denser toward base, propygidial gland openings present, difficult to distinguish from punctures, located about one-third behind anterior margin, one-fourth from lateral corners; pygidium with only fine ground punctation in apical half, secondary punctures becoming evident in basal half. Male: not known.

**Map 11.**
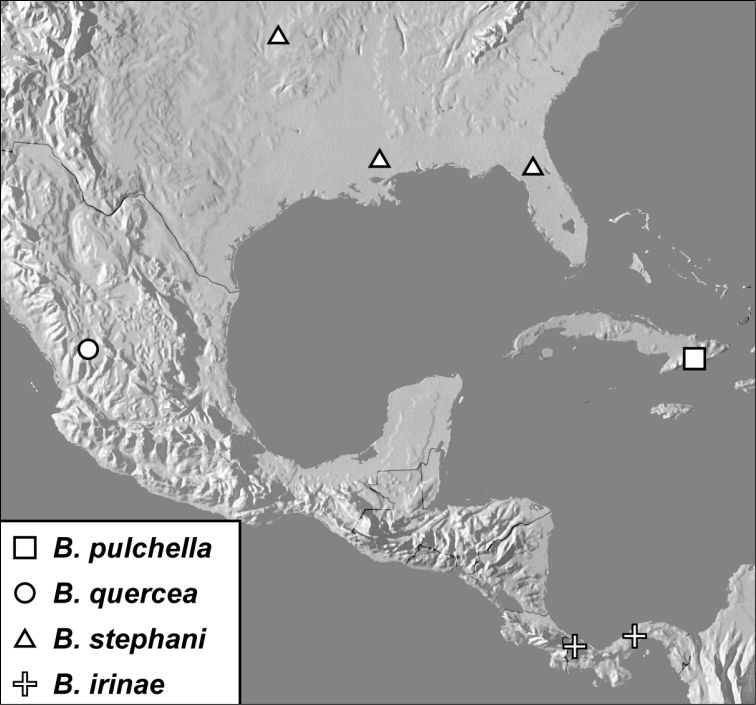
*Baconia aeneomicans* group records.

###### Remarks.

*Baconia pulchella* is highly distinct in both appearance and distribution. It is very similar, and closely related to *Baconia aeneomicans*, the only other metallic-colored *aeneomicans* group species in the Americas. They also share a frontal stria which is well-impressed at the sides and curves slightly inward at front (though obsolete across the front; Fig. 38B) and a mesometaventral stria which is strongly arched forward to almost completely displace the marginal mesoventral stria (Fig. 38D). The coloration (Fig. 38C) of *Baconia pulchella* is distinct, with the elytra violet rather than blue-green. It is known only from Cuba.

###### Etymology.

This species’ name means ‘beauty’, and it is among the most attractively colored *Baconia* species.

##### Baconia quercea sp. n.

http://zoobank.org/01DE6C40-9C6F-4D3F-AF5C-DA439A152B02

http://species-id.net/wiki/Baconia_quercea

Figs 38E 39D, F–H, K–L Map 11

###### Type locality.

MEXICO: Zacatecas: 13 mi W Milpillas [23.06°N, 103.88°W].

**Type material. Holotype male**: “Milpillas (13 mi. W), Zacat., MEXICO 9–12 July 1954 alt. 8400 ft.” / “on oak stump” / “R.H. Brewer leg. Field No. 1352” / “FMNH-INS 0000069304” (FMNH).

###### Diagnostic description.

Length: 1.8mm, width: 1.4mm; body elongate oval, moderately convex, glabrous; color rufopiceous, with blue-bronze metallic tinge, particularly on elytra; head with frons moderately convex, ground punctation fine, sparse, with few coarser punctures near dorsolateral corners, frontal stria present along inner edge of eye, absent across front, supraorbital stria present, connected to sides of frontal stria; antennal scape short, clubs missing from type; epistoma faintly emarginate apically; labrum about 4×wider than long, apical margin shallowly emarginate; mandibles short, each with acute basal tooth; pronotum with sides weakly convergent from base, lateral marginal stria continuous with complete anterior marginal stria, lateral submarginal stria complete, pronotal disk weakly depressed in anterolateral corners, ground punctation fine, very sparse, with coarser secondary punctures sparsely impressed over most of disk, very slightly larger toward prescutellar area; elytra with two epipleural striae, outer subhumeral stria absent, fine fragment of inner subhumeral stria present at base, dorsal striae 1–4 complete to base, weakly bent mediad in anterior one-third, progressively more abbreviated from apex, base of 4^th^ stria weakly arched mediad, 5^th^ stria slightly abbreviated from base, more strongly so from from apex, sutural stria present only as short fragment just behind middle, elytral disk with scattered secondary punctures in apical one-third, extending further anterad toward middle; prosternal keel moderately broad, distinctly emarginate at base, with more or less complete carinal striae converging from base, subparallel in anterior half; prosternal lobe about two-thirds keel length, apical margin bluntly rounded, with marginal stria present only at middle; mesoventrite weakly produced at middle, with marginal stria interrupted for width of prosternal keel; mesometaventral stria strongly and broadly arched forward, continuous laterally with inner lateral metaventral stria, which extends toward inner corner of metacoxa, outer lateral metaventral stria very short, oblique; metaventral disk moderately coarsely punctate at sides, impunctate at middle; abdominal ventrite 1 with single, complete inner lateral stria, lacking secondary punctures on middle portion of disk, ventrites 2–5 with fine punctures at sides, becoming more sparsely punctate across middle; protibiae missing from type; mesotibia with two marginal spines; outer metatibial margin smooth; propygidium lacking basal stria, with fine ground punctation and slightly coarser, ocellate punctures uniformly separated by just more than their diameters, propygidial gland openings inconspicuous; pygidium with sparse ground punctation becoming slightly denser apically, with small secondary punctures only in basal half. Male genitalia (Figs 39D, F–H, K–L): T8 broad, sides rounded in basal two-thirds, obliquely angulate to apex, basal emargination narrowly arcuate, ventrolateral apodemes with inner apices separated by about three-fourths T8 width, projecting beneath beyond ventral midpoint, obsolete apically, apical margin narrowly emarginate; S8 very short, halves fused along midline, basal emargination shallowly sinuate, basal apodemes narrow, corners obliquely subtruncate, sides slightly narrowed toward apex, apical margin nearly truncate, projecting slightly at corners of narrow median emargination, bearing a moderately dense fringe of apical setae; T9 with short, slightly attenuate basal apodemes, halves separated dorsally, ventrolateral apodemes well-developed, acutely recurved proximad beneath, apices of T9 narrowly rounded, with 1–2 subapical setae on each side; T10 entire; S9 with stem narrow, about half total length, with fine median keel, head abruptly widened, sides subparallel to apex, apices acute, apical emargination broad; tegmen with sides weakly convergent in about basal two-thirds, more strongly convergent to apex, apices narrowly rounded, apical half of tegmen weakly curved ventrad; median lobe about one-fourth tegmen length; basal piece nearly one-third tegmen length.

###### Remarks.

This species is most similar and closely related to *Baconia aeneomicans* and *Baconia pulchella*, sharing their relatively well developed frontal stria at the sides, and the arched mesometaventral stria displacing the marginal mesoventral stria. It is also weakly metallic in coloration (Fig. 38E), though under dim light it may not be distinct. Externally it lacks the 1^st^ abdominal ventrite punctures that *Baconia aeneomicans* shows, and has quite distinct male genitalia, with the apices of the 8^th^ sternite broad and subtruncate, rather than narrow and produced. There is a substantial gap between the only known locality for this species, in western Zacatecas, and the nearest localities for *Baconia aeneomicans*, in eastern Texas. Intervening localities may well bridge much of the morphological gap between the two species.

###### Etymology.

This species is named to recognize the association of the only known specimen with oak, or *Quercus*.

##### Baconia stephani sp. n.

http://zoobank.org/1382AAE3-42D2-4F07-A7B3-1559984C4CF4

http://species-id.net/wiki/Baconia_stephani

Figs 38F–G 39M–N Map 11

###### Type locality.

UNITED STATES: Oklahoma: Latimer Co. [exact locality uncertain].

###### Type material.

**Holotype male**: “OKLAHOMA: Latimer Co VIII-1988 Karl Stephan” (FMNH). **Paratypes** (10): same locality as type, 1: viii.1988, 1: ix.1984, 3: viii.1989, 2: viii.1990, 1: x.1990, 2: vii.1992 (TAMU, CHPWK, FMNH, MSCC, AKTC).

###### Other material.

**1: USA**: **Louisiana**: St. Tammany Par., Abita Creek Preserve, 30°31'25"N, 89°58'07"W, 6.vii.2000, FIT, C. Carlton & D. Prowell (LSAM): 1: 29.ix.2000, C. Carlton, D. Prowell (LSAM); 1: **Florida:** Alachua Co., Gainesville, Doyle Connor Bldg, P. Skelley, FIT, 19.iv-2.vii.1987, 1: 2–10.vii.1987, 1: 23.vii–4.viii.1987 (CHPWK).

###### Diagnostic description.

Length: 1.2–1.4mm, width: 0.9–1.1mm; body narrowly elongate oval, subdepressed, glabrous; color rufobrunneous, rather coarsely punctate on most surfaces; head with frons very shallowly depressed at middle, slightly elevated over antennal bases, ground punctation conspicuous, secondary punctures moderately coarse but sparse, frontal stria present along inner margin of eyes, but may be reduced, fragmented, supraorbital stria absent; antennal scape short, club oblong, slightly expanded apically; epistoma weakly convex along apical margin, faintly emarginate; labrum about 3×wider than long, apical margin straight to slightly emarginate; both mandibles with conspicuous, acute basal tooth; pronotum with sides weakly arcuate to apex, lateral marginal stria continuous with complete anterior marginal stria, lateral submarginal stria present in basal four-fifths, very close to marginal, pronotal disk weakly depressed in anterolateral corners, with coarse secondary punctures throughout; elytra with two epipleural striae, outer subhumeral stria absent, short fragment of inner subhumeral stria present at base, dorsal striae 1-4 complete to base, progressively abbreviated apically, 5^th^ dorsal stria slightly abbreviated at base, sutural stria abbreviated basally and apically, present only in middle one-third, elytral disk with coarse punctures in apical one-fourth, a few punctures extending anterad between striae; prosternal keel weakly depressed, very shallowly emarginate at base, carinal striae diverging anteriorly and posteriorly; prosternal lobe about two-thirds keel length, apical margin bluntly rounded, with marginal stria present only at middle; mesoventrite weakly produced at middle, with marginal stria almost entirely obsolete; mesometaventral stria arched forward, crenulate, continuous or weakly interrupted laterally from inner lateral metaventral stria, which extends obliquely posterad toward middle of metacoxa, outer lateral metaventral stria absent; metaventral disk moderately coarsely punctate at sides, with rather conspicuous ground punctation at middle; abdominal ventrite 1 with single inner lateral stria, ventrites 2–5 with sparse punctures at sides, finer across middle; protibia tridentate, the outer margin finely serrulate between denticles; mesotibia with two very weak marginal spines; outer metatibial margin with very fine subbasal denticle; propygidium lacking basal stria, with fine ground punctation and coarser, ocellate secondary punctures dense, larger basally; propygidial gland openings evident, located about one-third behind anterior margin, one-fourth from lateral corner, the immediately surrounding disk devoid of punctures; pygidium with fine, conspicuous ground punctation and a few coarser secondary punctures toward base. Male genitalia (Figs 39M–N): T8 narrowly, rather deeply emarginate; ventrolateral apodemes with inner apices widely separated, projecting beyond ventral midpoint, obsolete apically, apical margin shallowly emarginate; S8 with halves fused along midline, basal emargination broad, deep, basal apodemes widely separated, sides sinuately narrowed, apices obliquely truncate, densely setose, widely separated by acute apical emargination; T9 with short basal apodemes, halves nearly meeting dorsobasally, ventrolateral apodemes bluntly produced beneath, nearly to midline, apices narrowly rounded, with single seta borne on subapical tubercle on each side; T10 narrow, finely emarginate apically; S9 with long narrow, weakly keeled stem, head approximately triangular, apices subacute, apical emargination broad, shallow, sinuate; tegmen with base rather broad, widening slightly to subacute midpoint, corresponding to ventrolateral process, apices narrowed, tegmen strongly curved dorsoventrally, mainly near base, with eversible subapical denticles; median lobe about one-third tegmen length, simple; basal piece about one-third tegmen length.

###### Remarks.

Among species in the *Baconia aeneomicans* group, *Baconia stephani* is sympatric only with *Baconia aeneomicans* itself, and the two are rather similar in general. However, *Baconia stephani* differs consistently in its nonmetallic coloration, more narrowly elongate body (Fig. 38F), and especially its more coarsely punctate pronotum and 1^st^ abdominal ventrite (Fig. 38G). The genitalia of the two species are very similar, although the tegmen can be easily distinguished, with that of *Baconia stephani* strongly angulate at the middle of the outer edge (most distinct in lateral view; Fig. 39N) Although the specimens away from the type locality do not differ in any obvious characters (and male genitalia conform to the differences described here), we limit the type series to those specimens from eastern Oklahoma.

###### Etymology.

We name this species in honor of Karl Stephan, beetle collector extraordinaire.

##### Baconia irinae sp. n.

http://zoobank.org/D408D801-1A7D-4119-BDFF-44268A614255

http://species-id.net/wiki/Baconia_irinae

Figs 39O–T 40A–B Map 11

###### Type locality.

PANAMA: Colón: San Lorenzo Forest [9.28°N, 79.97°W].

###### Type material.

**Holotype male**: “**PANAMA: Colón Pr.**, San Lorenzo Forest, STRI crane site. 9°17'N, 79°58'W, F.I.T., 21m. FL-I1C21n. 5–18 June 2004 M.Rapp. IBISCA’04” / “Caterino/Tishechkin Exosternini Voucher EXO-00453” (FMNH). **Paratypes** (3): same locality as type, 1: FL-I1C28n. 5-18 June 2004, 1: FL-I1C28m, 25.v-5.vi.2004, 1: FL-I1C28o, 25.v-5.vi.2004 (AKTC, GBFM).

###### Other material.

**COSTA RICA**:1: **Heredia**: Est. Biol. La Selva, 10°26'N, 84°01'W, 50–150 m, 14.x.1994, *Pentaclethra macroloba* (INBI), 1: 17.v.2000, *Goethalsia meiantha* (INBI), 1: 24.x.1994, *Virola koschnyi* (MSCC).

###### Diagnostic description.

Length: 1.3–1.5mm, width: 1.0–1.2mm; body elongate oval, weakly depressed, glabrous; color dull blue metallic, principally on elytra, less conspicuously on other parts of dorsum, shining; head with frons very shallowly depressed at middle, slightly elevated over antennal bases, ground punctation fine, sparse, secondary punctures moderately coarser, interspersed dorsad, frontal stria present only at upper inner edge of eye, supraorbital stria present, meeting or nearly meeting frontal stria at sides; antennal scape short, club approximately circular; epistoma faintly emarginate apically; labrum about 3×wider than long, apical margin shallowly emarginate; both mandibles with acute basal tooth; pronotum with sides weakly rounded to apex, lateral marginal stria continuous with complete anterior marginal stria, lateral submarginal stria nearly complete, close to marginal, pronotal disk weakly depressed in anterolateral corners, ground punctation fine, sparse, with coarser secondary punctures uniformly interspersed throughout except for narrow, median impunctate band; elytra with two epipleural striae, outer subhumeral stria absent, fine fragment of inner subhumeral stria present at base, dorsal striae 1–4 complete to base, the inner striae strongly abbreviated from apex, base of 4^th^ stria arched mediad to near scutellum, 5^th^ stria slightly abbreviated basally, sutural stria more strongly so, elytral disk with scattered secondary punctures in apical one-third, extending further anterad toward middle; prosternal keel convex, narrowly but distinctly emarginate at base, carinal striae subparallel to converging slightly anterad; prosternal lobe about two-thirds keel length, apical margin rounded, with marginal stria present only at middle; mesoventrite produced at middle, with marginal stria complete; mesometaventral stria more or less transverse, continuous laterally with inner lateral metaventral stria which extends posterad toward inner corner of metacoxa, curving mediad slightly at apex, outer lateral metaventral stria short, more oblique; metaventral disk moderately coarsely punctate at sides, impunctate at middle; abdominal ventrite 1 with single, complete inner lateral stria, with secondary punctures more or less uniformly distributed on middle portion of disk, ventrites 2–5 with fine but rather deep punctures across entire width; protibia weakly tridentate, the outer margin serrulate between denticles; mesotibia with two very weak marginal spines; outer metatibial margin smooth; propygidium lacking basal stria, with fine ground punctation and slightly coarser, ocellate secondary punctures uniformly interspersed, propygidial gland openings inconspicuous; pygidium with sparse ground punctation becoming slightly denser apically, with secondary punctation denser toward base. Male genitalia (Figs 39O–T): T8 slightly longer than wide, widest just distad middle, basal emargination deep, narrowly arcuate, apical emargination shallow, ventrolateral apodemes with inner apices separated by about two-thirds T8 width, projecting beneath to about ventral midpoint, obsolete apically; S8 with halves fused along midline, basal emargination strongly bisinuate, trident-shaped, basal apodemes bluntly rounded, sides subparallel in basal two-thirds, widening to apices, apices obliquely subtruncate, bisetose, separated by broad, acuminate apical emargination; T9 with very short, bluntly rounded basal apodemes, halves narrowly separated dorsally, apices narrowly rounded, with single subapical seta on each side, ventrolateral apodemes subacute, projecting mediad nearly to midline beneath; T10 elongate, undivided; S9 with long narrow, medially keeled stem, head abruptly widened, rather broad, apices acute, widely separated, apical emargination broad, shallow; tegmen widest just distad base, very weakly, evenly narrowed to apex, apices subacute, tegmen curved ventrad in apical fourth; median lobe simple, very short, about one-eighth tegmen length; basal piece about one-fourth tegmen length.

**Figure 40.**
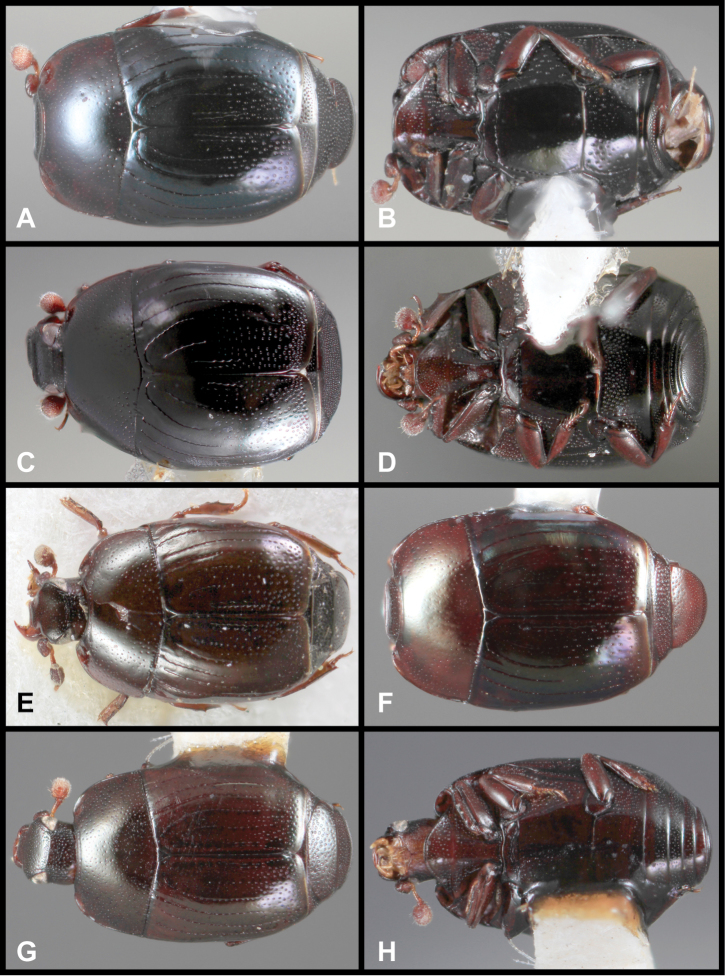
*Baconia aeneomicans* group. **A** Dorsal habitus of *Baconia irinae* **B** Ventral habitus of *Baconia irinae* **C **Dorsal habitus of *Baconia fornix* **D** Ventral habitus of *Baconia fornix* **E** Dorsal habitus of holotype of *Baconia slipinskii* **F** Dorsal habitus of *Baconia submetallica* **G** Dorsal habitus of *Baconia diminua* **H** Ventral habitus of *Baconia diminua*.

###### Remarks.

This species and the next share numerous external characters with those that follow, including generally rufo-piceous coloration, strongly arched dorsal striae, strongly reduced frontal striae, and punctures across the 1^st^ abdominal ventrite. However, both species lack the characteristic subapical aedeagal denticles found in most of the other species in this group. *Baconia irinae* can be distinguished from other species in this group by its faintly metallic blue coloration (Fig. 40A), more or less impunctate pronotal midline, pronotal sides which are only weakly rounded, converging weakly toward the front, subparallel prosternal striae, and the inner lateral metaventral stria being curved mediad apically (Fig. 40B). The series of speimens from Costa Rica is slightly larger in body size, but otherwise matches well (including in male genitalic features). These are excluded from the type series.

###### Etymology.

This species is named for the junior author’s wife, in recognition of her lasting understanding and support.

##### Baconia fornix sp. n.

http://zoobank.org/D2A35F0B-6DAB-4289-BA1E-35CC8D1A115E

http://species-id.net/wiki/Baconia_fornix

**Map 12.**
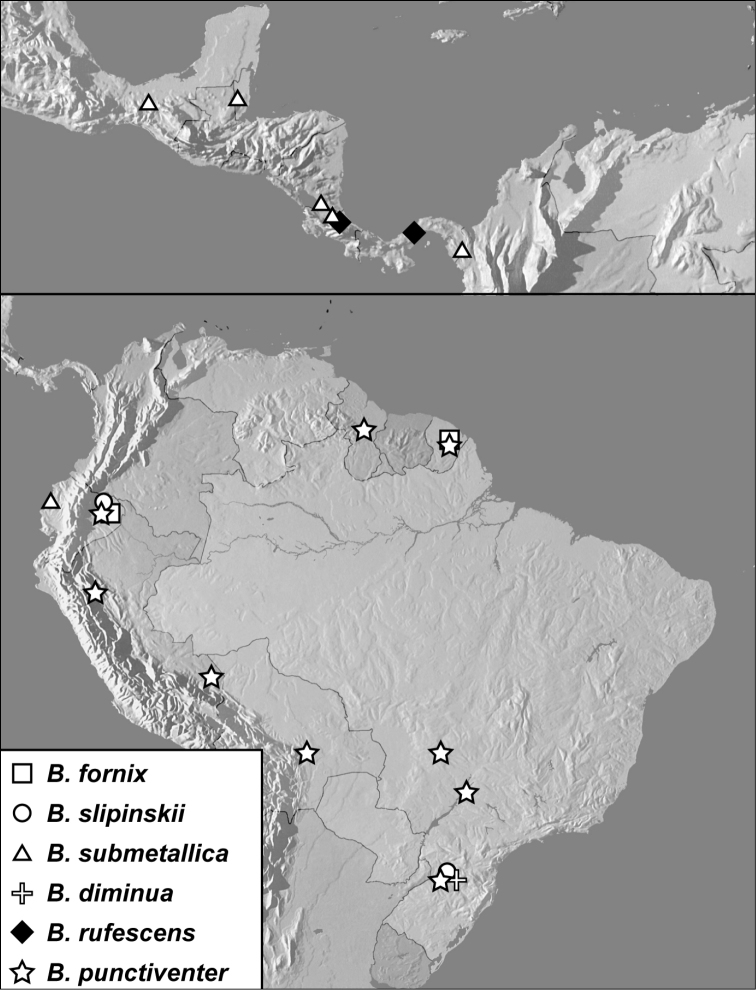
*Baconia aeneomicans* group records.

Figs 40C–D 41A–B, G, I, K–L Map 12

###### Type locality.

ECUADOR: Orellana: Tiputini Biodiversity Station [0.635°S, 76.150°W].

###### Type material.

**Holotype male**: “**ECUADOR: Depto. Orellana,** Tiputini Biodiversity Station 0°37'55"S, 76°08'39"W, 220–250m. 6 February 1999 T.L.Erwin *et al*. collectors” / “insecticidal fogging of mostly bare green leaves, some with covering of lichenous or bryophytic plants **Lot 2076 Trans. 8 Sta. 7**” / “Caterino/Tishechkin Exosternini Voucher EXO-00452” (USNM). **Paratypes** (1): Res. Ethnica Waorani, 1 km S Onkone Gare Camp, Trans. Ent., 0°39'10"S, 76°26'W, 220 m, 3.vii.1995, fogging, T. Erwin (USNM).

###### Other material.

1: **FRENCH GUIANA**:Montagne des Chevaux, 4°43'N, 52°24'W, 4.i.2009, FIT, SEAG (MNHN).

###### Diagnostic description.

**Figure 41.**
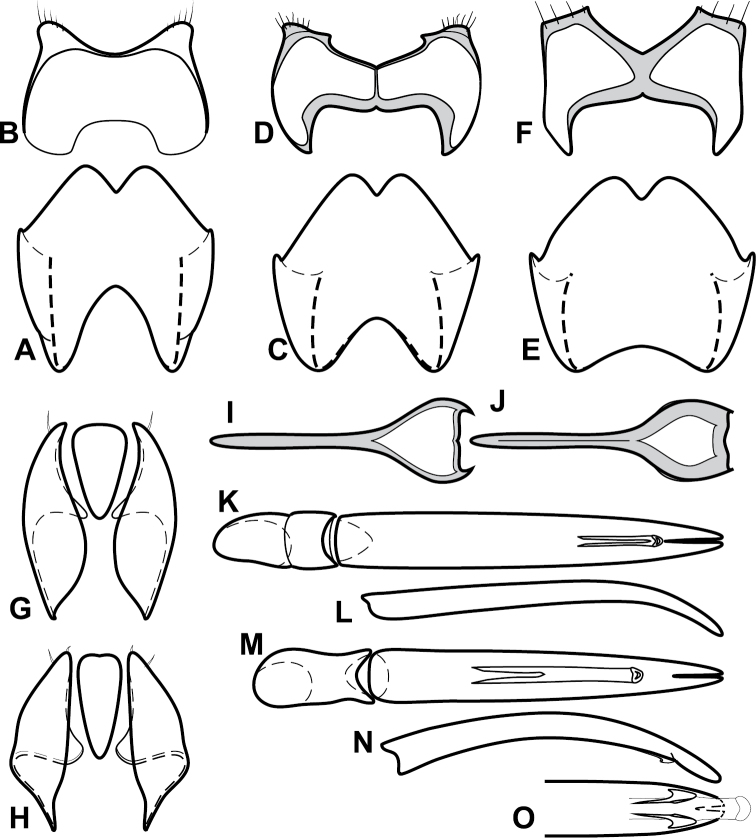
Male genitalia of *Baconia aeneomicans* group. **A** T8 of *Baconia fornix* **B** S8 of *Baconia fornix* **C** T8 of *Baconia slipinskii* **D** S8 of *Baconia slipinskii* **E** T8 of *Baconia submetallica* **F** S8 of *Baconia submetallica* **G** T9 & T10 of *Baconia fornix* **H **T9 & T10 of *Baconia submetallica* **I** S9 of *Baconia fornix* **J** S9 of *Baconia submetallica* **K** Aedeagus, dorsal view of *Baconia fornix* **L** Aedeagus, lateral view of *Baconia fornix* **M** Aedeagus, dorsal view of *Baconia submetallica* **N** Aedeagus, lateral view of *Baconia submetallica* **O** Aedeagus, ventral view of apex, of *Baconia submetallica*.

Length: 1.6–1.7mm, width: 1.3–1.4mm; body broadly elongate oval, slightly wider at humeri, weakly convex, glabrous; color rufopiceous, shining, rarely with slight metallic tinge, particularly on elytra; head with frons moderately narrowed and elongate, more or less flat, slightly elevated over antennal bases, ground punctation fine, denser near eyes, secondary punctures slightly coarser, very sparse, frontal stria barely impressed at upper edge of eye, supraorbital stria variably present at middle, may be detached from sides of frontal stria; antennal scape short, club approximately circular; epistoma slightly swollen along apical margin, faintly emarginate apically; labrum about 3×wider than long, apical margin shallowly emarginate; mandibles short, each with acute basal tooth; pronotum with sides arcuately convergent in basal two-thirds, curvature markedly interrupted where lateral marginal stria descends to lower edge of margin, about one-fourth from apex, continuous with anterior marginal stria in front, lateral submarginal stria complete, close to marginal, thin marginal bead distinctly elevated, pronotal disk weakly depressed in anterolateral corners, ground punctation fine, very sparse, with slightly coarser secondary punctures interspersed laterally, slightly larger toward prescutellar area, with median impunctate area about head-width; elytra with two epipleural striae, outer subhumeral stria absent, fine fragment of inner subhumeral present at base, often also with detached fragment near middle, dorsal striae 1–4 complete to base, bent mediad in anterior one-third, progressively more abbreviated from apex, base of 4^th^ stria arched mediad to near scutellum, recurved slightly along suture, 5^th^ stria strongly abbreviated from apex, nearly meeting basal arc of 4^th^, sutural stria more strongly abbreviated, present only in middle one-third, broadened anterad, elytral disk with scattered secondary punctures in apical one-half, densest along apical margin, smaller and sparser anterad; prosternal keel weakly depressed toward front, shallowly emarginate at base, with more or less complete carinal striae converging slightly to front; prosternal lobe about three-fourths keel length, apical margin rounded, with marginal stria present only at middle, and rather dense ground punctation in anterior half; mesoventrite produced at middle, with complete marginal stria; mesometaventral stria arched slightly forward, continuous laterally with inner lateral metaventral stria, extending posterad toward inner corner of metacoxa, curving mediad slightly at apex, outer lateral metaventral stria present in basal half, subparallel to inner; metaventral disk moderately coarsely punctate at sides, impunctate at middle; abdominal ventrite 1 with single, complete inner lateral stria, with secondary punctures rather densely impressed on anterior half of middle portion of disk, ventrites 2–5 with fine punctures at sides, ventrites 2–3 more finely punctate at middle, 4–5 densely punctate across middle; protibia tridentate, the outer margin serrulate between denticles; mesotibia with two marginal spines; outer metatibial margin smooth; propygidium lacking basal stria, with fine ground punctation and coarser, ocellate secondary punctures uniformly separated by about their diameters, propygidial gland openings inconspicuous; pygidium with sparse ground punctation becoming slightly denser apically, with small secondary punctures denser toward base. Male genitalia (Figs 41A–B, G, I, K–L): T8 slightly longer than wide, widest just distad middle, basal emargination deep, narrowly arcuate, apical emargination shallow, ventrolateral apodemes with inner apices separated by about two-thirds T8 width, projecting beneath to about ventral midpoint, obsolete apically; S8 with halves fused along midline, basal emargination evenly arcuate, basal apodemes obliquely truncate, sides weakly convergent in basal three-fourths, widening slightly to apices, apices bluntly rounded, bearing 4-5 setae, separated by broad, arcuate apical emargination; T9 with very short, broad basal apodemes, apices narrowly rounded, with single subapical seta on each side, ventrolateral apodemes subacute, projecting nearly to midline beneath; T10 short, undivided; S9 with long narrow stem, head abruptly widened, rather broad, apices acute, widely separated, apical emargination broad, sinuate; tegmen narrow, widest just distad base, very weakly narrowed to apex, apices slightly separated, subacute, tegmen curved ventrad in apical third; median lobe simple, about one-third tegmen length; basal piece about one-fourth tegmen length.

**Remarks.** This species is very similar and closely related to the preceding, both of them having the apices of the tegmen slightly divergent. *Baconia fornix* may be distinguished by its slightly broader form (Fig. 40C), rather elongate pronotum and frons, more strongly abbreviated elytral striae 4 and 5, and by the dense punctures on the basal half of abdominal ventrite 1 (Fig. 40D). The singleton from French Guiana exhibits a faint metallic blue coloration shown in neither of the types. It also has the punctures of the 1^st^ abdominal ventrite covering nearly the whole median part of the disk. Therefore we exclude it from the type series.

**Etymology.** This species’ name refers to the ‘arch’ of the 4^th^ elytral stria.

##### Baconia slipinskii

Mazur, 1981

http://species-id.net/wiki/Baconia_slipinskii

Figs 40E 41C–D Map 12

Baconia slipinskii Mazur, 1981: 184.

###### Type locality.

BRAZIL: Santa Catarina: Nova Teutonia [27.18°S, 52.38°W].

###### Type material.

**Holotype** (MNHG): “Brésil: I-78, Santa Catarina, Nova Teutonia, F. Plaumann” / “TYPUS” / “*Baconia slipinskii*”, examined 2012. **Paratypes** (2): same locality as type, xii.1977 (MNHG).

###### Other material.

**ECUADOR: Napo**, Sacha, 7.iii.1983, leg. L. Huggert (MNHG).

###### Diagnostic description.

Length: 1.5–1.6mm, width: 1.3–1.4mm; body elongate oval, parallel-sided, subdepressed, glabrous; color rufobrunneus, shining; head with frons flat, slightly produced over antennal bases, ground punctation fine, with few, sparse coarser punctures dorsad, frontal stria, if present, only at upper corner of eye, absent across front, supraorbital stria present at middle, may be attached to sides of frontal stria; antennal scape short, club slightly expanded apically; epistoma short, slightly convex along apical margin, faintly emarginate; labrum about 4×wider than long, apical margin shallowly emarginate; mandibles short, each with acute basal tooth; pronotum with sides weakly convergent in basal half, more strongly curved to apex, lateral marginal stria descends to ventral edge of pronotum in posterior two-thirds, detached from anterior marginal stria, which diverges slightly from margin behind eye, lateral submarginal stria present in basal two-thirds, pronotal disk weakly depressed in anterolateral corners, ground punctation very fine and sparse, with very small secondary punctures sparsely scattered in lateral thirds; elytra with two epipleural striae, outer subhumeral stria absent, inner subhumeral absent or barely visible near base, dorsal striae 1-2 nearly complete, slightly abbreviated at apex, striae 3–4 present only in basal half, 4^th^ slightly longer than 3^rd^ posteriorly, and arched toward scutellum at base, 5^th^ stria shorter than 4^th^ or sutural, sutural stria present in about middle one-third, elytral disk with very small, very sparse secondary punctures in apical one-third to one-half, extending further anterad toward suture; prosternal keel moderately broad, emarginate at base, with more or less complete carinal striae diverging basally and apically, few punctures between in anterior half; prosternal lobe slightly over half keel length, apical margin bluntly rounded, with marginal stria present only at middle; mesoventrite produced at middle, with marginal stria weak or interrupted medially; mesometaventral stria very weakly arched forward at middle, continuous laterally with inner lateral metaventral stria, which extends obliquely toward middle of metacoxa, outer lateral metaventral stria short, oblique; metaventral disk moderately coarsely punctate at sides, impunctate at middle, but with few small punctures in front of metacoxa; abdominal ventrite 1 with complete inner lateral stria, with a few small secondary punctures in anterior half of middle portion, ventrites 2–5 with fine punctures at sides, those of ventrite 4 dense across middle, the others more sparsely punctate; protibia very weakly tridentate, the middle tooth strongly reduced, margin serrulate between; mesotibia with two marginal spines; outer metatibial margin with very small subbasal denticle; propygidium lacking basal stria, with fine ground punctation and coarser, ocellate punctures uniformly separated by slightly less than their diameters, propygidial gland openings inconspicuous; pygidium with sparse ground punctation becoming slightly denser apically, with small secondary punctures only in basal half. Male genitalia (Figs 41C–D): T8 rather deeply, arcuately emarginate at base, ventrolateral apodemes with inner apices subparallel, separated by about two-thirds T8 width, projecting beneath to about ventral midpoint, obsolete apically, apical margin shallowly, acutely emarginate; S8 short, with halves narrowly fused, slightly more strongly sclerotized along midline and basal and apical margins, basal emargination broad, sinuate, subacute at middle, basal apodemes tapered, blunt, sides slightly narrowed to apex, apices narrowly rounded, deflexed, inner corner slightly produced, with a few apical setae, apical emargination broad, sinuate, subacute at middle; T9 with short, narrow basal apodemes, separated dorsally, ventrolateral apodemes bluntly produced beneath, apices of T9 narrowly rounded, with single subapical seta on each side; T10 with weak apical emargination; S9 with long, narrow, medially keeled stem, head abruptly widened, sides weakly rounded to apex, apices acute, widely separated, apical emargination broad, sinuate; tegmen with sides subparallel from base to about midpoint, narrowed to apex, apices subacute, tegmen weakly but evenly curved in lateral aspect, with eversible subapical denticles ventrally; median lobe about one-third tegmen length; basal piece about one-fourth tegmen length.

###### Remarks.

*Baconia slipinskii* can be recognized by the following combination of characters: a relatively broad, subquadrate body form, the 4^th^ elytral stria extending further posterad than either the 3^rd^ or the 5^th^ (Fig. 40E), relatively fine and sparse secondary punctures on both the frons and pronotum, small punctures on the anterior half of the 1^st^ abdominal ventrite that are separated by about twice their diameters, and a small cluster of very fine metaventral punctures near the metacoxa (similar to Fig. 45C).

##### Baconia submetallica sp. n.

http://zoobank.org/D868E863-28C6-4997-8B04-6BE32DDEA201

http://species-id.net/wiki/Baconia_submetallica

Figs 40F 41E–F, H, J, M–N Map 12

###### Type locality.

BELIZE: Cayo: Las Cuevas Research Station [16.73°N, 88.98°W].

###### Type material.

**Holotype male**: “BELIZE: Cayo; Las Cuevas Research Station, 550m 16°44.33N, 88°59.07W V/30/2000 M.Caterino” / “flight interception trap” / “Caterino/Tishechkin Exosternini Voucher EXO-00459” (BMNH). **Paratypes** (3): 1: **BELIZE**: **Cayo**: Las Cuevas Res. Sta., 8.vi.1997, FIT, D. Inward (BMNH); 1: **Orange Walk**: Rio Bravo Cons. Area, Rd. to Arch. site, 18–25.iv.1996, FIT, C.E. Carlton (CHPWK) 1: **MEXICO**: **Chiapas**: Laguna Belgica, 16 km NW Ocozocoautla, 970 m, 31.v.1990, H. & A. Howden (CMNC).

###### Other material.

**COSTA RICA**:2: **Heredia**: Est. Biol. La Selva, 10°26'N, 84°01'W, 50–150 m, 14.x.1994, *Pentaclethra macroloba* (INBI); 1: **Limón**: Sector Cerro Cocori, Finca de E. Rojas, 150 m, 9–30.xi.1992, E. Rojas (INBI). **NICARAGUA**: 1: **Rio San Juan**: Ref. Bartola, 60 km SE San Carlos, 10°58.40'N, 84°20.30W, 100 m, 28–30.v.2002, FIT, R. Brooks, Z. Falin & S. Chatzimanolis (SEMC). **PANAMA**:1: **Darién**: Cana, Pirre Camp, 7°45.825'N, 77°43.325'W, 1320 m, 6.v.2008, A. Gillogly (AKTC).

###### Diagnostic description.

Length: 1.3–1.4mm, width: 1.1–1.2mm; body elongate oval, subdepressed, glabrous; color rufobrunneus, anterior two-thirds of elytra faintly metallic blue; head with frons produced over antennal bases, very weakly depressed at middle, ground punctation conspicuous, with few, sparse coarser punctures, frontal stria present only at upper corner of eye, absent across front, supraorbital stria present at middle, detached from sides of frontal stria; antennal scape short, clubs missing from type; epistoma faintly emarginate apically; labrum about 3×wider than long, apical margin shallowly emarginate; mandibles short, each with acute basal tooth; pronotum with sides weakly convergent in basal half, rather abruptly convergent to apex, lateral marginal stria descends to ventral edge of pronotum in posterior two-thirds, detached from anterior marginal stria, which diverges slightly from margin behind eye, lateral submarginal stria present in basal three-fourths, pronotal disk weakly depressed in anterolateral corners, ground punctation fine, very sparse, with slightly coarser secondary punctures sparsely scattered in lateral thirds; elytra with two epipleural striae, outer and inner subhumeral striae absent, dorsal striae 1–2 complete, striae 3–4 present only in basal half, stria 5 abbreviated from base and apex, about as long as stria 4 but displaced slightly posterad, sutural stria similar in length and displaced further posterad, elytral disk with very small, very sparse secondary punctures in apical one-third, extending further anterad toward middle; prosternal keel weakly convex, emarginate at base, with more or less complete carinal striae converging from base, few punctures between; prosternal lobe about two-thirds keel length, apical margin rounded, with marginal stria present only at middle; mesoventrite produced at middle, with marginal stria narrowly interrupted; mesometaventral stria broadly arched forward, continuous laterally with inner lateral metaventral stria, extending toward inner third of metacoxa, outer lateral metaventral stria short, oblique; metaventral disk moderately coarsely punctate at sides, impunctate at middle; abdominal ventrite 1 with complete inner lateral stria and short fragment of outer lateral stria, with a few small secondary punctures in anterior half of middle portion, ventrites 2–5 with fine punctures at sides, those of ventrite 4 dense across middle, the others more sparsely punctate across middle; protibia weakly tridentate, margin serrulate between; mesotibia with two marginal spines; outer metatibial margin with very small subbasal denticle; propygidium short, wide, lacking basal stria, with fine ground punctation and slightly coarser, ocellate punctures uniformly separated by about their diameters, propygidial gland openings inconspicuous; pygidium with sparse ground punctation becoming slightly denser apically, with small secondary punctures only in basal half. Male genitalia (Figs 41E–F, H, J, M–N): T8 broadly, shallowly emarginate at base, ventrolateral apodemes with inner apices subparallel, separated by about three-fourths T8 width, projecting beneath to about ventral midpoint, obsolete apically, apical margin shallowly emarginate; S8 with halves narrowly fused, more strongly sclerotized along midline, basal emargination broad, subacute at middle, basal apodemes tapered, blunt, sides slightly narrowed to apex, apices obliquely truncate with inner corner slightly produced, with a few apical setae, apical emargination broad, sinuate, subacute at middle; T9 with short, narrow basal apodemes, separated dorsally, ventrolateral apodemes bluntly produced beneath, apices of T9 narrowly rounded, with apical and subapical setae on each side; T10 with weak apical emargination; S9 with long, narrow, medially keeled stem, head abruptly widened, sides weakly rounded, narrowed to apex, apices acute, apical emargination broad, sinuate; tegmen narrow, with sides subparallel from base to about midpoint, narrowed to apex, apices subacute, tegmen weakly but evenly curved in lateral aspect, with eversible subapical denticles ventrally; median lobe about one-half tegmen length; basal piece almost one-third tegmen length.

###### Remarks.

*Baconia submetallica* is very similar to the preceding species, differing mainly in its narrower, slightly rounded body form (Fig. 40F), generally coarser frontal punctation, and by the faint but distinct metallic coloration in the anterior half of the elytra. Their male genitalia differ in the form of the apices of the male 8^th^ sternite, which in *Baconia submetallica* are wider and more distinctly truncate, and by the very slightly narrower aedeagus of *Baconia submetallica*.

We exclude several specimens as types from more southerly parts of Central America due mainly to variation in the intensity of dorsal coloration, with specimens from Nicaragua and Costa Rica showing more distinctly blue elytral coloration, and a specimen from Panama showing essentially no hints of metallic. Male genitalia are similar throughout the range.

###### Etymology.

This species is named for the faint metallic luster on the anterior half of its elytra.

##### Baconia diminua sp. n.

http://zoobank.org/21BEB6FB-02FF-4691-AD18-979E391042BE

http://species-id.net/wiki/Baconia_diminua

Figs 40G–H 42A–B, G, I, K–L Map 12

###### Type locality.

BRAZIL: Santa Catarina: Nova Teutonia [27.18°S, 52.38°W].

###### Type material.

**Holotype male**: “BRAZIL: Nova Teutonia, Oct. 1978, 27°11' B[sic] 52°23', 300–500m, Plaumann” / “FMNH-INS 0000069306” (FMNH). **Paratypes** (2): same locality as holotype, 1: 27.ix.1957, under bark, F. Plaumann (FMNH), 1: ii.1979, F. Plaumann (FMNH).

###### Other material.

1: **BRAZIL: São Paulo**, Lençóis Paulista Duraflora, Fazenda Rio Claro, 22°49'19.0"S, 48°53'36.5"W, 5 yr-old *Eucalyptus grandis* stand, 8.ix.2006, C.A.H. Flechtmann (UNESP).

###### Diagnostic description.

**Figure 42.**
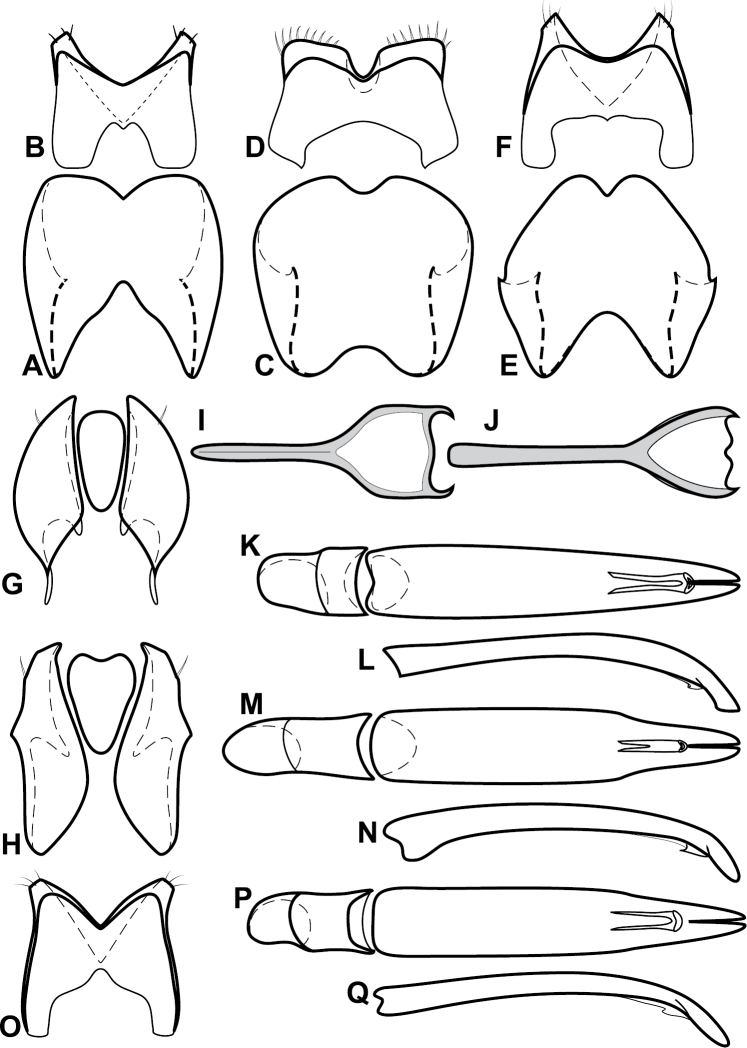
Male genitalia of *Baconia aeneomicans* group. **A** T8 of *Baconia diminua* **B** S8 of *Baconia diminua* **C **T8 of *Baconia rufescens* **D** S8 of *Baconia rufescens* **E** T8 of *Baconia aulaea* **F** S8 of *Baconia aulaea* **G** T9 & T10 of *Baconia diminua* **H **T9 & T10 of *Baconia aulaea* **I** S9 of *Baconia diminua* **J** S9 of *Baconia aulaea* **K** Aedeagus, dorsal view of *Baconia diminua* **L** Aedeagus, lateral view of *Baconia diminua* **M** Aedeagus, dorsal view of *Baconia aulaea* **N** Aedeagus, lateral view of *Baconia aulaea* **O** S8 of *Baconia punctiventer* **P** Aedeagus, dorsal view of *Baconia punctiventer* **Q** Aedeagus, lateral view of *Baconia punctiventer*.

Length: 1.7–1.8mm, width: 1.3–1.4mm; body elongate oval, subparallel-sided, humeri slightly wider, subdepressed, glabrous; color rufopiceous, faintly bronzy-metallic; head with frons more or less flat, ground punctation conspicuous, few sparse coarser punctures dorsad, frontal stria present only at upper corner of eye, absent across front, supraorbital stria variably present, frequently fragmented, usually detached from frontal stria; antennal scape short, club more or less circular; epistoma short, slightly convex along apical margin, faintly emarginate; labrum about 4×wider than long, apical margin shallowly emarginate; mandibles short, each with acute basal tooth; pronotum with sides weakly convergent in basal half, rather abruptly convergent to apex, lateral marginal stria descends to ventral edge of pronotum in posterior two-thirds, usually continuous with anterior marginal stria, but may be narrowly interrupted behind eye, lateral submarginal stria present in basal three-fourths, pronotal disk weakly depressed in anterolateral corners, ground punctation fine, very sparse, with slightly coarser secondary punctures sparsely scattered in lateral thirds, denser toward lateral margin; elytra with two epipleural striae, outer subhumeral stria absent, inner subhumeral stria present at extreme base, dorsal striae 1–4 slightly and progressively abbreviated mediad from apices, 4^th^ stria weakly arched toward scutellum at base, 5^th^ stria distinctly abbreviated from base and apex, shorter than 4^th^ stria, sutural stria shorter than 5^th^, present only at middle third, elytral disk with small, sparse secondary punctures in apical one-fourth, extending further anterad toward middle; prosternal keel weakly emarginate at base, with more or less complete, subparallel carinal striae; prosternal lobe about two-thirds keel length, apical margin rounded, marginal stria present only at middle; mesoventrite weakly produced at middle, with marginal stria interrupted for about width of prosternal keel; mesometaventral stria sinuately arched forward, continuous laterally with inner lateral metaventral stria, which extends obliquely toward middle of metacoxa, outer lateral metaventral stria present as at most a basal fragment; metaventral disk moderately coarsely punctate at sides, impunctate at middle; abdominal ventrite 1 with single, complete inner lateral stria, lacking secondary punctures on middle portion, ventrites 2–5 with fine punctures at sides, more sparsely punctate across middle; protibiae tridentate, margin serrulate between; mesotibia with two marginal spines; outer metatibial margin with very small subbasal denticle; propygidium lacking basal stria, with fine ground punctation and slightly coarser, ocellate punctures uniformly separated by about their diameters, propygidial gland openings inconspicuous; pygidium with sparse ground punctation becoming slightly denser apically, with small secondary punctures very sparse in basal half. Male genitalia (Figs 42A–B, G, I, K–L): T8 narrowly, acutely emarginate at base, ventrolateral apodemes with inner apices separated by about one-half T8 width, projecting beneath to about ventral midpoint, tapered apically, apical margin shallowly emarginate; S8 with halves fused along midline, basal emargination deeply sinuate, basal apodemes truncate, sides subparallel to near apex, expanded slightly apically, apices narrowly, obliquely truncate, with a few apical setae, apical emargination broad, arcuate; T9 with short, narrow, subacute basal apodemes, halves separated dorsally, ventrolateral apodemes acutely produced, slightly recurved proximad beneath, apices of T9 narrowly rounded, with single subapical seta on each side; T10 poorly sclerotized, with weak apical emargination; S9 with long narrow, medially keeled stem, head abruptly widened, sides parallel to apex, apices acute, curving slightly mediad, apical emargination broad, shallow, sinuate; tegmen with sides subparallel from base to about midpoint, narrowed to apex, apices subacute, tegmen very weakly curved in lateral aspect, apex more strongly deflexed, narrowed near base, with eversible subapical denticles ventrally; median lobe about one-fourth tegmen length; basal piece about one-fourth tegmen length.

###### Remarks.

While this species shares a type locality with *Baconia slipinskii*, and is generally quite similar, it does exhibit a number of consistent differences, both externally and in male genitalia. *Baconia diminua* is distinct in its narrower body form (Fig. 40G), in elytral striation, with the 3^rd^-sutural striae longer and progressively shorter mediad, in its lack of dense punctures across the 1^st^ and any subsequent abdominal ventrites (Fig. 40H), and its rather dense lateral pronotal punctation. The 8^th^ sternite of *Baconia diminua* is narrower and more elongate than that of *Baconia slipinskii*, and the apices are more distinctly acute, with a deep, narrowly arcuate median emargination between them. One female from São Paulo state is excluded from the type series since male genitalic characters cannot be confirmed.

###### Etymology.

This species’ name refers to the ‘diminishing’ length of the elytral striae as they approach the sutural.

##### Baconia rufescens sp. n.

http://zoobank.org/D9CFC63D-ADDB-49CE-9113-E7EF28F80961

http://species-id.net/wiki/Baconia_rufescens

Figs 42C–D 43A Map 12

###### Type locality.

PANAMA: Colón: San Lorenzo Forest [9.28°N, 79.97°W].

###### Type material.

**Holotype male**: “**PANAMA: Colón Prov.**, San Lorenzo Forest, STRI crane site. 9°17'N, 79°58'W, FIT B2-18. 26–29 May 2004 A.K.Tishechkin. AT-524” / “Caterino/Tishechkin Exosternini Voucher EXO-00458” (FMNH). **Paratypes** (2): **COSTA RICA**:1: **Heredia**: Est. Biol. La Selva, 3.2 km SE Puerto Viejo, 100 m, 30.i.1992, FIT, W. Bell (SEMC); 1: Est. Biol. La Selva, 10°26'N, 84°01'W, 50–150 m, 1.xi.1993 (INBI).

###### Other material.

1: **ECUADOR**: **Manabí**: Bosque Seco Lalo Loor, 0.0824°S, 80.1503°W, 25.v.2011, fogging understory, M. Caterino & A. Tishechkin, DNA Extract MSC-2168, EXO-00698 (MSCC).

###### Diagnostic description.

Length: 1.2–1.3mm, width: 0.9–1.0mm; body elongate oval, subdepressed, glabrous; color rufobrunneus, shining; head with frons very shallowly depressed at middle, slightly elevated over antennal bases, ground punctation conspicuous, secondary punctures fine and sparse with few coarser punctures dorsad, frontal and supraorbital striae absent; antennal scape short, club oblong, slightly expanded apically; epistoma truncate apically; labrum about 4×wider than long, apical margin straight to slightly emarginate; both mandibles with small, acute basal tooth; pronotum with sides weakly convergent in basal two-thirds, abruptly convergent to apex, lateral marginal stria descending to ventral edge in posterior two-thirds, barely detached anteriorly from anterior marginal stria, free ends of anterior marginal stria may diverge slightly from anterior margin, lateral submarginal stria present in basal three-fourths, pronotal disk weakly depressed in anterolateral corners, ground punctation fine, sparse, with slightly coarser secondary punctures interspersed at sides and along base, disk more or less impuncatate on middle two-thirds; elytra with two epipleural striae, outer subhumeral stria absent, fine fragment of inner subhumeral stria present at base, dorsal striae 1–4 complete to base, vaguely abbreviated apically, 5^th^ and sutural striae slightly abbreviated basally and apically, nearly complete, elytral disk with scattered secondary punctures in apical one-third; prosternum moderately broad, keel very shallowly emarginate at base, carinal striae sinuate, converging slightly anterad; prosternal lobe about two-thirds keel length, apical margin rounded, with marginal stria present only at middle; mesoventrite produced at middle, with marginal stria interrupted for width of prosternal keel; mesometaventral stria arched forward, continuous laterally with inner lateral metaventral stria which curves posterad to middle of metacoxa, outer lateral metaventral stria weak, sinuate behind mesocoxa; metaventral disk moderately coarsely punctate at sides, impunctate at middle; abdominal ventrite 1 with single, complete inner lateral stria, ventrites 2–5 with sparse punctures at sides, finer across middle; protibia weakly tridentate, the outer margin serrulate between denticles; mesotibia with two very weak marginal spines; outer metatibial margin with very fine subbasal denticle; propygidium lacking basal stria, with fine ground punctation and coarser, ocellate secondary punctures uniformly interspersed, propygidial gland openings inconspicuous; pygidium with sparse ground punctation becoming denser apically, with slightly coarser secondary punctation denser toward base. Male genitalia (Fig 42C–D): T8 narrowly, shallowly emarginate at base, ventrolateral apodemes with inner apices separated by about two-thirds T8 width, projecting beneath just beyond ventral midpoint, obsolete apically, apical margin shallowly emarginate; S8 with halves fused along midline, basal emargination broad, shallow, basal apodemes broadly truncate, sides slightly narrowed, weakly arcuate, apices broadly truncate, setose, narrowly separated by apical emargination; T9 with very short, subacute basal apodemes, halves narrowly separated dorsally, ventrolateral apodemes bluntly produced beneath, nearly meeting, apices narrowly rounded, with single subapical seta on each side; T10 elongate, narrowed basally, with weak apical emargination; S9 with long narrow, medially keeled stem, head abruptly widened, sides parallel to apex, apices acute, widely separated, apical emargination broad, shallow; tegmen with sides weakly widened from base, subparallel to just beyond midpoint, narrowed to apex, apices subacute, tegmen evenly but not strongly curved in lateral aspect; median lobe about one-fourth tegmen length, with subapical denticulate plates; basal piece about one-fourth tegmen length.

**Figure 43.**
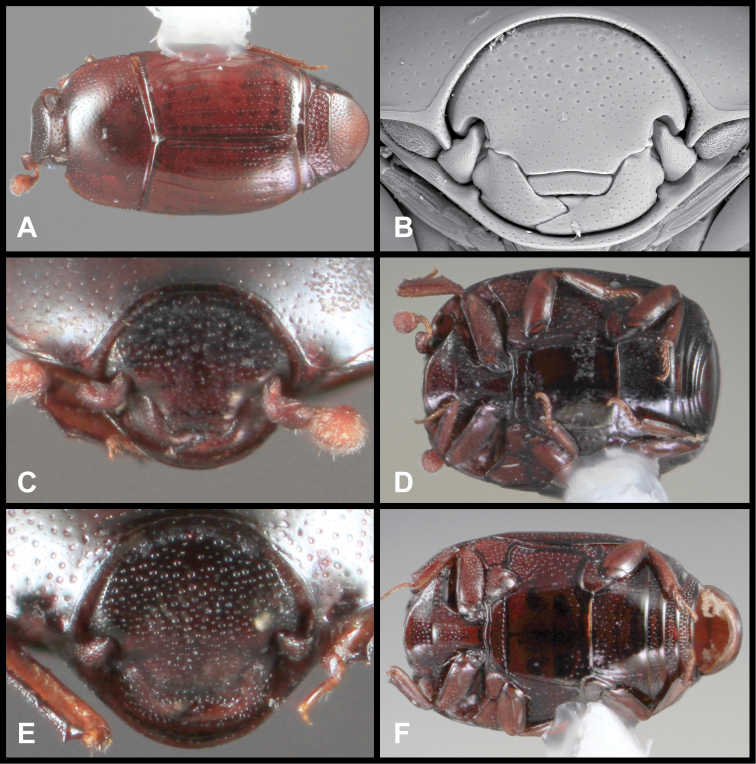
*Baconia aeneomicans* group. **A** Dorsal habitus of *Baconia rufescens* **B** Frons of *Baconia punctiventer* **C **Frons of *Baconia aulaea* **D** Ventral habitus of *Baconia aulaea* **E** Frons of *Baconia mustax* **F** Ventral habitus of *Baconia mustax*.

###### Remarks.

*Baconia rufescens* is distinctive in a number of characters. It exhibits an unusually narrow body form (Fig. 43A), has most of the elytral striae, including the sutural, more or less complete and straight, the 4^th^ stria not arched mediad at the base, and has the mesometaventral stria arched strongly forward, displacing the marginal mesoventral stria. The single specimen from the Pacific slope of Ecuador is very similar in all respects, but is slightly narrower, and has the lateral metaventral striae detached from the mesometaventral stria. Therefore we exclude it from the type series.

###### Etymology.

This species is named for its rufescent coloration, relatively unusual in this more typically rufopiceous species group.

##### Baconia punctiventer sp. n.

http://zoobank.org/73E55971-6BE0-4716-8BDC-7B824EDA9D3E

http://species-id.net/wiki/Baconia_punctiventer

Fig 42O–Q 43B Map 12

###### Type locality.

ECUADOR: Orellana:Res. Ethnica Waorani [0.67°N, 76.43°W].

###### Type material.

**Holotype male**: “**ECUADOR: Depto. Orellana**:Res. Ethnica Waorani, 1km S Onkone Gare Camp, Trans. Ent., 0°39'10"S, 76°26'W, 220m, 2 July 1995, T.L. Erwin et al. collectors” / “Insecticidal fogging of mostly bare green leaves, some with covering of lichenous or bryophytic plants in terra firme forest. Project MAXUS **Lot 1064 Trans. 7 Sta. 4**” / “Caterino/Tishechkin Exosternini Voucher EXO-00505” (USNM). **Paratypes** (13): **ECUADOR**:1: **Orellana**: Est. Biodiv. Tiputini, 0°37'55"S, 76°08'39"W, 220–250 m, 22.x.1998, fogging, mostly bare green leaves, some with covering of lichenous or brophytic plants, T. Erwin (USNM), 1: 23.x.1998, fogging, T. Erwin (USNM), 1: 24.x.1998, fogging, T. Erwin (USNM), 1: P. N. Yasuní, Est. Cient. Yasuní, 0°40.5'S, 76°24'W, 20–24.vii.2008, Malaise/FIT, canopy, A. Tishechkin, DNA Extract MSC-1889, EXO-00451; 1: Res. Ethnica Waorani, 1 km S Onkone Gare Camp, Trans. Ent., 0°39'10"S, 76°26'W, 220 m, 1.vii.1995, fogging, T. Erwin (USNM), 1: 2.vii.1995, fogging, T. Erwin (USNM), 1: 22.vi.1996, fogging, T. Erwin (USNM), 1: 25.vi.1994, fogging, T. Erwin (USNM), 1: 26.vi.1996, fogging, T. Erwin (USNM), 1: 3.vii.1994, fogging, T. Erwin (USNM), 1: 7.vii.2006, fogging, T. Erwin, DNA Extract MSC-2134, EXO-00671, 1: 0°39'26"S, 76°27'11"W, 216 m, 21.i.2006, fogging, T. Erwin (USNM), 1: 22.x.2005, fogging, T. Erwin (USNM).

###### Other material.

**(**33) 1: **ECUADOR**: **Orellana**: P. N. Yasuní, Est. Cient. Yasuní, 0°40.5'S, 76°24'W, 29.vi–17.vii.1999, FIT, mid canopy, A. Tishechkin (LSAM); 1: Res. Ethnica Waorani, 1 km S Onkone Gare Camp, Trans. Ent., 0°39'26"S, 76°27'W, 216 m, 23.i.2006, fogging, T. Erwin (USNM); 2: Est. Biodiv. Tiputini, 0°37'55"S, 76°08'39"W, 220–250 m, 9.ii.1999, fogging, T. Erwin (USNM), 2: 23.x.1998, fogging, T. Erwin (USNM), 1: 4.vii.1998, fogging, T. Erwin (USNM); 1:canopy fogging, T. Erwin, DNA Extract MSC-1903, EXO-02697 (MSCC). **BOLIVIA**: 1: **Santa Cruz**:Hotel Flora y Fauna, 4–5 km SSE Buena Vista, 17°29.92'S, 63°39.13'W, 440 m, 24–31.xii.2003, FIT, S. & J. Peck. **BRAZIL**: 1: **Goias**: Mineiro (BMNH); 1: **Mato Grosso do Sul**: cerradao fragment nr. Selviria, 20°20'10"S, 51°24'36"W, 1.5 m, 11.ii.2011, FIT, C. Flechtmann, 1: 11.xii.2010, FIT, C. Flechtmann, 2: 16.x.2010, FIT, C. Flechtmann, 1: 23.x.2010, FIT, C. Flechtmann, 1: 23.x.2011, FIT, C. Flechtmann, 2: 28.i.2011, FIT, ground level, C. Flechtmann, 1: 30.xii.2010, FIT, C. Flechtmann, 1: 31.xii.2010, FIT, C. Flechtmann, 3: 4.xii.2011, FIT, C. Flechtmann, 1: 7.i.2011, FIT, C. Flechtmann (UNESP, MSCC, AKTC); 1: **Santa Catarina**: Nova Teutonia, xi.1938, F. Plaumann (NHRS). **FRENCH GUIANA**: 1:Montagne des Chevaux, 4°43'N, 52°24'W, 11.vii.2009, FIT, SEAG (CHND). **GUYANA**:1: **Region 8**:Iwokrama Field Stn., Turtle Mt. summit, 4°43'57"N, 58°44'1"W, 290 m, 30.v-1.vi.2001, FIT, R. Brooks & Z. Falin (SEMC). **PERU**:1: **Madre de Dios**: Res. Tambopata, 30 km (air) SW Pto. Maldonado, 12°50'S, 69°20'W, 290 m, 25.ii.1984, canopy fogging, T. Erwin (USNM), 1: 4.v.1984, canopy fogging, T. Erwin (USNM), 1: 9.xi.1983, canopy fogging, T. Erwin (USNM); 1: **San Martín**: Almiranto, 1900 m, 12.xii.1936, subtrop. forest, F. Woytkowski (CHSM).

###### Diagnostic description.

Length: 1.4–1.7mm, width: 1.1–1.2mm; body elongate oval, weakly depressed, glabrous; color rufopiceous, shining, rarely with slight metallic blue tinge, particularly on elytra; head with frons flat, ground punctation fine, sparse, secondary punctures moderately coarser, sparsely interspersed on most of frons, frontal stria barely impressed at upper edge of eye, supraorbital stria present at middle, detached from sides of frontal stria; antennal scape short, club approximately circular; epistoma faintly emarginate apically; labrum about 4×wider than long, apical margin shallowly emarginate; mandibles short, each with acute basal tooth; pronotum with sides slightly convergent in basal two-thirds, rounded to apex, lateral marginal stria continuous with complete anterior marginal stria, lateral submarginal stria nearly complete, pronotal disk weakly depressed in anterolateral corners, ground punctation fine, very sparse, with slightly coarser secondary punctures interspersed laterally, slightly larger toward prescutellar area, with median impunctate area about head-width; elytra with two epipleural striae, outer subhumeral stria absent, fine fragment of inner subhumeral stria present at base, dorsal striae 1–4 complete to base, the inner striae strongly abbreviated from apex, base of 4^th^ stria arched mediad to near scutellum, 5^th^ stria strongly abbreviated from apex, slightly abbreviated basally, sutural stria more strongly abbreviated, present only in middle one-third, stria broadened anterad, elytral disk with scattered secondary punctures in apical one-third, extending further anterad toward middle; prosternal keel weakly convex, shallowly emarginate at base, carinal striae converging slightly at middle; prosternal lobe about two-thirds keel length, apical margin rounded, with marginal stria present only at middle; mesoventrite produced at middle, with complete marginal stria; mesometaventral stria arched weakly forward, continuous laterally with inner lateral metaventral stria, which extends posterad toward inner corner of metacoxa, curving mediad slightly at apex, outer lateral metaventral stria present in basal half to nearly complete, subparallel to inner; metaventral disk moderately coarsely punctate at sides, impunctate at middle; abdominal ventrite 1 with complete inner lateral stria and abbreviated outer stria, with deep secondary punctures rather densely impressed on middle portion of disk, ventrites 2–5 with fine punctures at sides, ventrites 2–3 becoming nearly impunctate at middle, ventrites 4–5 densely but finely punctate across entire width; protibia tridentate, the outer margin serrulate between denticles; mesotibia with two very weak marginal spines; outer metatibial margin smooth; propygidium lacking basal stria, with fine ground punctation and coarser, ocellate secondary punctures uniformly separated by slightly less than their diameters, propygidial gland openings distinct, narrowly elongate, the immediately surrounding disk impunctate; pygidium with sparse ground punctation becoming slightly denser apically, with small secondary punctures denser toward base. Male genitalia (Figs 42O–Q): T8 with basal emargination rather narrow, deep, ventrolateral apodemes with inner apices separated by about two-thirds T8 width, projecting beneath to about ventral midpoint, obsolete apically, apical margin narrowly emarginate; S8 with halves fused along midline, basal emargination deeply sinuate, basal apodemes bluntly rounded, sides narrowed toward apex, weakly expanded near apex, apices narrowly, obliquely truncate, with a few apical setae, apical emargination broad, arcuate; T9 with short, broadly rounded basal apodemes, halves separated dorsally, ventrolateral apodemes tapering, narrowly rounded, nearly meeting beneath, apices of T9 narrowly rounded, with single subapical seta on each side; T10 weakly sclerotized, with shallow apical emargination; S9 with long, narrow, keeled stem, head abruptly widened, sides subparallel to apex, apices acute, apical emargination broad, shallow, sinuate; tegmen with sides subparallel in about basal two-thirds, narrowed to apex, apices narrowly rounded, tegmen weakly curved in lateral aspect, more strongly curved toward apex; median lobe about one-fourth tegmen length, with apical denticulate plates; basal piece about one-fourth tegmen length.

###### Remarks.

*Baconia punctiventer* can be generally distinguished by its coarser frontal punctures (Fig. 43A), pronotal punctures enlarged slightly toward the scutellum, the complete, uninterrupted marginal mesoventral stria, the rather deep punctures of abdominal ventrite 1, and by the curving mediad of the apex of the inner lateral metaventral stria. Many individuals exhibit a very faint metallic blue tinge, but this is not consistent even within localities. Due to considerable variation in several external characters, and the existence of several very similar species, we limit the type series to a subset of specimens from Amazonian Ecuador. Even among Ecuadorian specimens two more or less discrete size classes of individuals are represented, which otherwise exhibit no differences that we can see (including in male genitalia). We conservatively exclude a number of smaller specimens from the type series as well, on the chance that these might represent a cryptic sibling species.

###### Etymology.

This species’ name refers to the rather distinctive punctures of the 1^st^ abdominal ventrite.

##### Baconia aulaea sp. n.

http://zoobank.org/BDD9BD20-83F5-433B-BEEB-AE0A9757F624

http://species-id.net/wiki/Baconia_aulaea

**Map 13.**
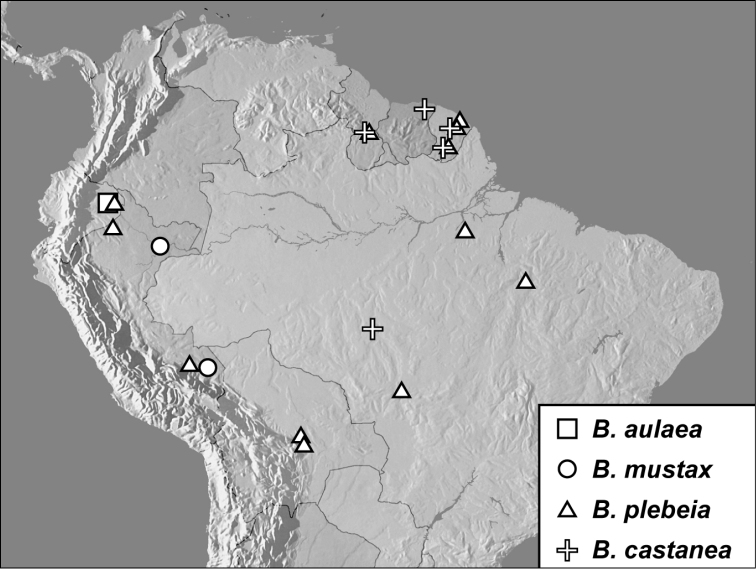
*Baconia aeneomicans* group records.

Figs 42E–F, H, J, M–N 43C–D Map 13

###### Type locality.

ECUADOR: Orellana:Res. Ethnica Waorani [0.67°N, 76.43°W].

###### Type material.

**Holotype male**: “**ECUADOR: Depto. Orellana**:Res. Ethnica Waorani, 1km S Onkone Gare Camp, Trans. Ent., 0°39'10"S, 76°26'W, 220m, 4 October 1995, T.L. Erwin et al. collectors” / “Insecticidal fogging of mostly bare green leaves, some with covering of lichenous or bryophytic plants in terra firme forest. Project MAXUS **Lot 1171 Trans. 1 Sta. 1**” / “Caterino/Tishechkin Exosternini Voucher EXO-00508” (USNM). **Paratype** (1): same locality as type, 26.vi.1996, Lot 1587 Trans. 7 Sta. 7 (USNM).

###### Diagnostic description.

Length: 1.2–1.3mm, width: 1.0–1.1mm; body broadly elongate oval, weakly depressed, glabrous; color rufobrunneus, shining; head with frons flat, ground punctation fine, rather dense, secondary punctures moderately coarser, interspersed dorsad, frontal stria absent; antennal scape short, club approximately circular; epistoma faintly emarginate apically; labrum about 4×wider than long, apical margin shallowly emarginate; mandibles short; pronotum with sides convergent, rounded to apex, lateral marginal stria continuous with complete anterior marginal stria, lateral submarginal stria nearly complete, may join marginal near anterior corner, pronotal disk weakly depressed in anterolateral corners, ground punctation fine, very sparse, with slightly coarser secondary punctures uniformly interspersed, slightly larger toward prescutellar area; elytra with two epipleural striae, outer subhumeral stria absent, fine fragment of inner subhumeral stria present at base, dorsal striae 1-4 complete to base, the inner striae strongly abbreviated from apex, base of 4^th^ stria arched mediad to near scutellum, 5^th^ stria slightly abbreviated basally, sutural stria more strongly abbreviated, stria broadened anterad, elytral disk with scattered secondary punctures in much of apical half, extending further anterad toward middle; prosternal keel weakly convex, shallowly emarginate at base, carinal striae converging slightly anterad; prosternal lobe about two-thirds keel length, apical margin rounded, with marginal stria present only at middle; mesoventrite produced at middle, with marginal stria narrowly interrupted; mesometaventral stria arched slightly forward, detached from inner lateral metaventral stria which extends posterad toward inner corner of metacoxa, curving mediad slightly at apex, outer lateral metaventral stria short, subparallel to inner stria in basal half; metaventral disk moderately coarsely punctate at sides, impunctate at middle; abdominal ventrite 1 with single, complete inner lateral stria, with small secondary punctures sparsely distributed on middle portion of disk, ventrites 2–5 with fine but rather deep punctures across entire width; protibia tridentate, the outer margin serrulate between denticles; mesotibia with two very weak marginal spines; outer metatibial margin smooth; propygidium lacking basal stria, with fine ground punctation and coarser, ocellate secondary punctures uniformly separated by slightly less than their diameters, propygidial gland openings inconspicuous; pygidium with sparse ground punctation becoming slightly denser apically, with secondary punctation denser toward base. Male genitalia (Figs 42E–F, H, J, M–N): T8 with basal emargination rather deep, narrow, ventrolateral apodemes with inner apices separated by about two-thirds T8 width, projecting beneath to about ventral midpoint, obsolete apically, apical margin narrowly emarginate; S8 with halves fused along midline, basal emargination deeply sinuate, basal apodemes obliquely subtruncate, sides narrowed toward apex, apices narrowly, obliquely truncate, with a few apical setae, apical emargination broad, arcuate; T9 with short, broadly rounded basal apodemes, halves separated dorsally, ventrolateral apodemes tapering, narrowly rounded, slightly recurved proximad beneath, apices of T9 obliquely truncate, with single subapical seta on each side; T10 poorly sclerotized, with weak apical emargination; S9 with long, narrow stem, lacking median keel, head abruptly widened, with narrowly desclerotized margins, sides weakly rounded to apex, narrowed slightly, apices acute, apical emargination broad, shallow, sinuate; tegmen with sides subparallel in about basal two-thirds, abruptly narrowed to apex, apices narrowly rounded, tegmen very weakly curved in lateral aspect, apex more strongly deflexed; median lobe about one-fourth tegmen length, with apical denticulate plates; basal piece about one-third tegmen length.

###### Remarks.

This species’ closest relatives, based on a similarly shaped male 8^th^ sternite that has narrow apices and a distinct ‘V’-shaped apicoventral concavity, are *Baconia diminua* and *Baconia punctiventer*, the latter of which is sympatric. *Baconia aulaea* can be distinguished from either of these by the relatively conspicuous ground punctation of the frons and epistoma (Fig. 43C), the anteriorly convergent prosternal striae (Fig. 43D), the long but incomplete outer lateral metaventral stria, and the small, irregularly sparse punctures of abdominal ventrite 1. The abruptly narrowed aedeagal apex is also distinct from these other species.

###### Etymology.

The name of this species refers to the canopy from which it was collected.

##### Baconia mustax sp. n.

http://zoobank.org/D1132A65-DE2E-4F3F-B7EB-4B38D872772A

http://species-id.net/wiki/Baconia_mustax

Figs 43E–F 44A–D, I–J Map 13

###### Type locality.

PERU: Madre de Dios:Rio Los Amigos, CICRA [12.57°S, 70.10°W].

###### Type material.

**Holotype male**: “**PERU Madre de Dios,** Rio Los Amigos, CICRA 18/21.XI.2006, 25/150m leg. Angélico Asenjo Flight intercept trap” / “Collección MUSM Lima-Peru” / “Caterino/Tishechkin Exosternini Voucher EXO-00509” (MUSM). **Paratype** (1): **PERU**: **Loreto**: 68 km SW Iquitos to Nauta, Rio Itaya, 4°11'S, 73°26'W, 110 m, 18-19.i.2008, A. Petrov (AKTC).

###### Diagnostic description.

**Figure 44.**
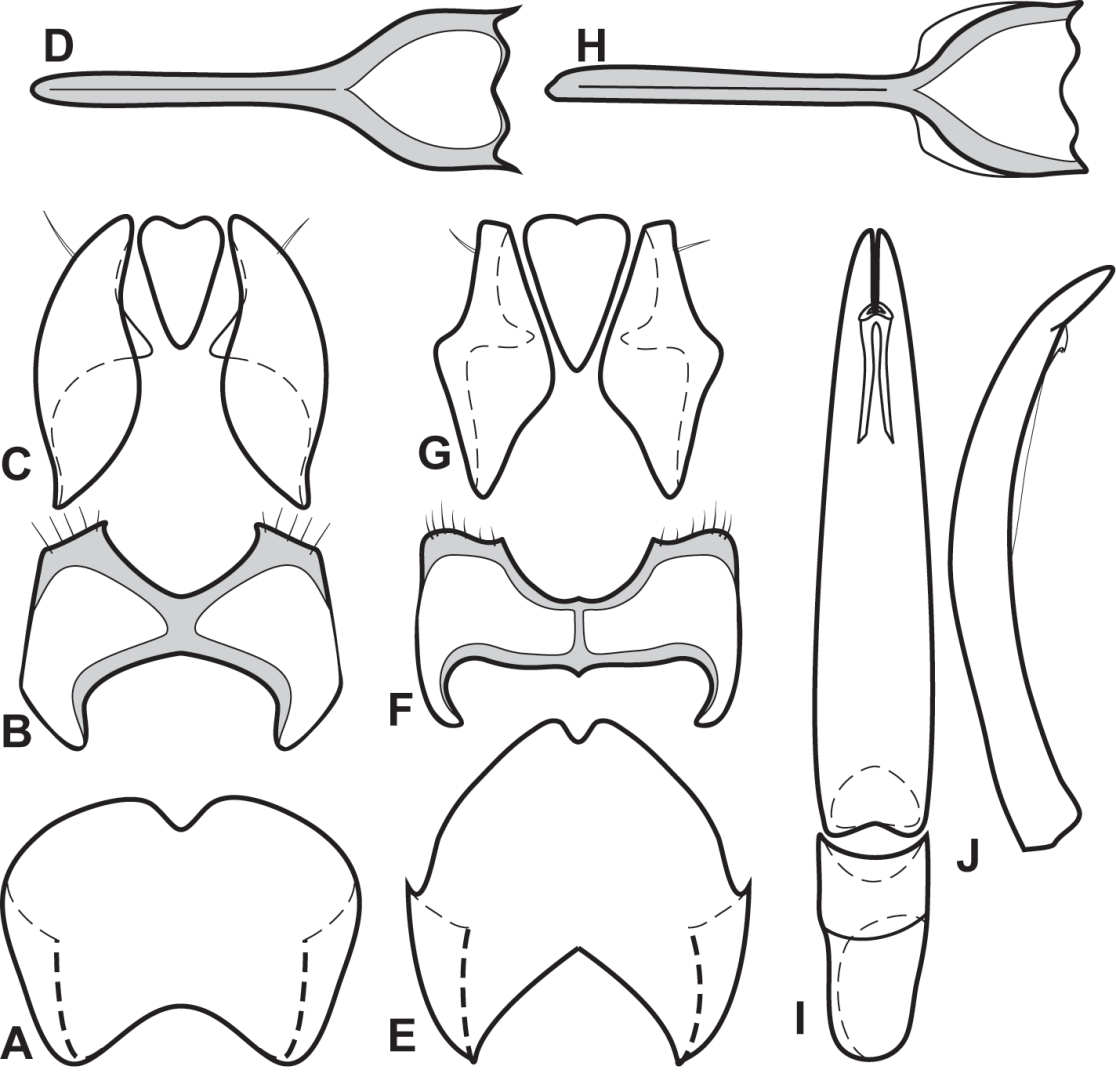
Male genitalia of *Baconia aeneomicans* group. **A** T8 of *Baconia mustax* **B** S8 of *Baconia mustax* **C** T9 & T10 of *Baconia mustax* **D** S9 of *Baconia mustax* **E** T8 of *Baconia plebeia* **F** S8 of *Baconia plebeia* **G** T9 & T10 of *Baconia plebeia* **H** S9 of *Baconia plebeia* **I** Aedeagus, dorsal view of *Baconia mustax* **J** Aedeagus, lateral view of *Baconia mustax*.

Length: 1.3–1.4mm, width: 1.0–1.1mm; body elongate oval, subparallel-sided, humeri slightly wider, subdepressed, glabrous; color rufopiceous, faintly bronzy; head with frons slightly produced over antennal bases, weakly depressed at middle, ground punctation conspicuous, with sparse coarser punctures dorsad, frontal stria present only at upper corner of eye, absent across front, supraorbital stria variably present, frequently fragmented, may be detached from frontal stria; antennal scape short, club more or less circular; epistoma with fine, dense, rugose microsculpture, apical margin faintly emarginate; labrum short, about 4×wider than long, apical margin shallowly emarginate; mandibles short, each with acute basal tooth; pronotum with sides subparallel in basal two-thirds, rather abruptly convergent to apex, lateral marginal stria descending to ventral edge of pronotum in posterior two-thirds, detached from anterior marginal stria, which diverges from anterior behind eye, lateral submarginal stria present in basal two-thirds, diverging slightly from margin toward front, pronotal disk weakly depressed in anterolateral corners, ground punctation fine, very sparse, coarser secondary punctures sparsely scattered in lateral thirds, becoming slightly larger toward prescutellar region; elytra with two epipleural striae, outer subhumeral stria absent, inner subhumeral stria present at extreme base, dorsal striae 1–2 similar in length, only slightly abbreviated apically, 3^rd^ stria present in about basal half, 4^th^ stria slightly longer than 3^rd^, weakly arched toward scutellum at base, 5^th^ stria shorter than 4^th^, more strongly abbreviated apically and basally, sutural stria about equal in length to 5^th^, situated slightly posterad, elytral disk with small, sparse secondary punctures in nearly apical half, extending further anterad toward middle; prosternal keel emarginate at base, with more or less complete carinal striae weakly divergent basally and apically, with few punctures in anterior half; prosternal lobe about two-thirds keel length, apical margin rounded, marginal stria present only at middle; mesoventrite produced at middle, with marginal stria complete, mesoventral disk with a few punctures; mesometaventral stria weakly arched forward, continuous laterally with inner lateral metaventral stria, which extends posterad toward inner third of metacoxa, outer lateral metaventral stria very short, oblique; metaventral disk moderately coarsely punctate at sides, impunctate at middle except for several distinct punctures anteromediad metacoxa; abdominal ventrite 1 with single, complete inner lateral stria, with small secondary punctures in anterior half of middle portion, ventrites 2–5 with fine punctures at sides, those of ventrites 3–4 dense across middle; protibiae with basal and median marginal teeth weak or absent, margin serrulate; mesotibia with two marginal spines; outer metatibial margin with very small subbasal denticle; propygidium lacking basal stria, with fine ground punctation and rather dense, ocellate secondary punctures uniformly separated by about half their diameters, propygidial gland openings inconspicuous; pygidium with sparse ground punctation becoming slightly denser apically, with small secondary punctures conspicuous throughout, larger and denser in basal half. Male genitalia (Figs 44A–D, I–J): T8 broadly, shallowly emarginate at base, ventrolateral apodemes with inner apices subparallel, separated by about three-fourths T8 width, projecting beneath to about ventral midpoint, obsolete apically, apical margin shallowly emarginate; S8 with halves narrowly fused, more strongly sclerotized along midline, basal emargination broad, subacute at middle, basal apodemes tapered, blunt, sides slightly narrowed to apex, apices acutely truncate with inner corner slightly produced, with a few apical setae, apical emargination broad, arcuate; T9 with short, narrow basal apodemes, separated dorsally, ventrolateral apodemes bluntly produced beneath, apices of T9 narrowly rounded, with single subapical seta on each side; T10 with weak apical emargination; S9 with long narrow, medially keeled stem, head abruptly widened, sides weakly rounded to apex, apices acute, widely separated, apical emargination broad, sinuate; tegmen with sides subparallel from base to about midpoint, narrowed to apex, apices subacute, tegmen moderately curved in lateral aspect, with eversible subapical denticles ventrally; median lobe about one-fourth tegmen length; basal piece about one-fourth tegmen length.

###### Remarks.

This species is very similar to *Baconia slipinskii*, exhibiting a few distinct punctures on the metaventrite anteromediad the metacoxae (Fig. 43F). However, it can easily be distinguished by its distinctive epistomal microsculpture (Fig. 43E). In addition *Baconia mustax* has relatively coarse pronotal and frontal punctation, and a more strongly dorsoventrally curved aedeagus.

###### Etymology.

The name of this species means ‘mustache’, and refers to the distinctive microsculpture of the epistoma.

##### Baconia plebeia sp. n.

http://zoobank.org/9B543FE5-A219-458A-A88A-51EF690C727E

http://species-id.net/wiki/Baconia_plebeia

Figs 44E–H 45A–C Map 13

###### Type locality.

FRENCH GUIANA: Réserve Trésor [4.59°N, 52.25°W].

###### Type material.

**Holotype male**: “**GUYANE FR.,** Rés. Trésor (route de Kaw Pk18), 4°36.63'N, 52°16.74'W, 225m Piege d’interception 12 Nov 2009 SEAG leg.” / “Caterino/Tishechkin Exosternini Voucher EXO-02591” (MNHN). **Paratypes** (53): **FRENCH GUIANA**: 1:Rés. des Nouragues, Camp Inselberg, 4°05'N, 52°41'W, 22.ix.2010, FIT, SEAG, 4:25.i.2011, FIT, SEAG, 1:30.ix.2010, FIT, SEAG, 2:8.x.2010, FIT, SEAG, 5:9.xi.2010, FIT, SEAG, 1:16.ix.2010, FIT, SEAG; 2:Rés. des Nouragues, Régina, 4°2.27'N, 52°40.35'W, 28.i.2010, FIT, SEAG (CHND); 2:8.ix.2009, FIT, SEAG (CHND); 2:Res. Trinité, 4°40.11'N, 53°16.99'W, 8.x.2010, FIT, Foret de transition, SEAG; 2:Belvèdére de Saül, 3°1'22"N, 53°12'34"W, 10.xii.2010, FIT, SEAG, 1:14.iii.2011, FIT, SEAG, 3:17.i.2011, FIT, SEAG, 1:2.ix.2011, FIT, SEAG, 2:20.xii.2010, FIT, SEAG, 1:24.i.2011, FIT, SEAG, 5:31.xi.2010, FIT, SEAG, 1:4.i.2011, FIT, SEAG, 6:7.ii.2011, FIT, SEAG, 1:9.ix.2010, FIT, SEAG; 1:Mont tabulaire Itoupé, 3°1.38'N, 53°5.73'W, 570 m, 17.iii.2010, FIT, SEAG (CHND); 1:Montagne des Chevaux, 4°43'N, 52°24'W, 1.viii.2009, FIT, SEAG (CHND), 1:11.iv.2009, FIT, SEAG, 1:11.vii.2009, FIT, SEAG (CHND), 1:22.i.2011, FIT, SEAG, 1:22.v.2010, FIT, SEAG, 2:6.vi.2009, FIT, SEAG (CHND); 2:Savane Matiti, 4.08333°N, 52.6167°W, 30.xi.2011, FIT, SEAG (MNHN).

###### Other material.

(18) **FRENCH GUIANA**: 1: Montagne des Chevaux, 4°43'N, 52°24'W, 6.vi.2009, FIT, SEAG (CHND), 1:8.viii.2010, FIT, SEAG; 1: Belvèdére de Saül, 3°1'22"N, 53°12'34"W, 31.xi.2010, FIT, SEAG. **GUYANA**:1: **Region 8**:Iwokrama Field Stn., 4°40'19"N, 58°41'4"W, 21.v.2001, *Acromyrmex hystrix* refuse pile, R. Brooks & Z. Falin (SEMC). **BOLIVIA**, 1: **Santa Cruz**:Amboro National Park, Los Volcanes, 18°06'S, 63°36'W, 1000 m, 20.xi-12.xii.2004, FIT, H. Mendel & M. Barclay (BMNH); 1:3.7 km SSE Buena Vista, Flora y Fauna Hotel, 17°29.9'S, 63°33.2'W, 400-440 m, 4–9.xi.2003, FIT, R. Leschen (AKTC). **BRAZIL**: 1: **Maranhão**: Estreito, 6°34'S, 47°27'W, v.1993, FIT (CHND); 1: Mirador, Calcarinha, 6°22'S, 44°22'W, 1.v.1993, FIT(CHND); 1: **Mato Grosso do Sul**: Mpio. Cuiaba, Chapada, September (CMNH); 3: **Pará**: Monte Alegre, 3°09'S, 52°03'W, 17.vi–3.vii.1992, FIT (CHND); 2: Tucuruí, 3°45'S, 49°40'W, vi.1985, FIT (CHND). **ECUADOR**:1: **Orellana**: P. N. Yasuní, Est. Cient. Yasuní, 0°40.5'S, 76°24'W, 7-13.vii.1999, C. Carlton & A. Tishechkin (LSAM); 1: Res. Ethnica Waorani, 1 km S Onkone Gare Camp, Trans. Ent., 0°39'10"S, 76°26'W, 220 m, 21.vi.1996, fogging, mostly bare green leaves, some with covering of lichenous or brophytic plants in terra firme forest, T. Erwin (USNM), 1: 4.x.1995, fogging, T. Erwin (USNM). **PERU**:1: **Loreto**: 1.5 km Teniente Lopez, 2°35.66'S, 76°06.92'W, 210–240 m, 17.vii.1993, palmfruit berlese, R. Leschen (SEMC); 1: **Madre de Dios**: Pantiacolla Lodge, Alto Madre de Dios R., 12°39.3'S, 71°13.9'W, 420 m, 14–19.xi.2007, FIT, D. Brzoska (SEMC).

###### Diagnostic description.

Length: 1.2–1.5mm, width: 1.0–1.3mm; body broadly elongate oval, subdepressed, glabrous; color rufescent to rufobrunneus, shining; head with frons slightly elevated over antennal bases, weakly depressed at middle, ground punctation fine, rather dense, with coarser punctures over most of frons, frontal stria present only at upper corner of eye, rarely with fragments further along inner eye margin, absent across front, supraorbital stria represented by few median fragments; antennal scape short, club slightly oblong; epistoma faintly emarginate; labrum about 4×wider than long, apical margin emarginate; mandibles short, each with small, acute basal tooth; pronotum with sides weakly convergent in basal two-thirds, rounded to apex, lateral marginal stria detached from median part of anterior marginal stria, which diverges from margin behind eye, lateral submarginal stria present in basal three-fourths, diverging slightly from margin toward front, pronotal disk weakly depressed in anterolateral corners, ground punctation fine, very sparse, middle of disk impunctate with small secondary punctures in lateral thirds, extending slightly further mediad along basal margin; elytra with two epipleural striae, outer subhumeral stria absent, inner subhumeral stria absent or present only at extreme base, dorsal striae 1-2 similar in length, only slightly abbreviated apically, 3^rd^ stria present in basal one-third, 4^th^ stria slightly longer, curving mediad toward scutellum at base, 5^th^ stria shorter than 4^th^, present only in basal half, sutural stria slightly longer than 5^th^, situated further posterad, elytral disk with small secondary punctures in most of apical one-half; prosternal keel weakly convex, slightly emarginate at base, with more or less complete carinal striae subparallel or converging slightly to front; prosternal lobe about two-thirds keel length, apical margin rounded, marginal stria obsolete at sides; mesoventrite weakly produced at middle, with marginal stria complete; mesometaventral stria broadly but weakly arched forward, continuous laterally with inner lateral metaventral stria, which extends obliquely posterad toward inner third of metacoxa, outer lateral metaventral stria very short, oblique; metaventral disk moderately sparsely punctate at sides, impunctate at middle or with few small punctures in front of metacoxa; abdominal ventrite 1 with two complete lateral striae, with small median discal punctures limited to anterior half, ventrites 2–5 with fine punctures at sides, those of ventrites 3 and 4 moderately dense across middle; protibiae weakly tridentate, with median and basal marginal denticles very small, margin serrulate; mesotibia with two marginal spines; outer metatibial margin with fine subbasal denticle; propygidium lacking basal stria, with fine ground punctation interspersed with ocellate secondary punctures separated by about half their diameters, propygidial gland openings inconspicuous; pygidium with fine ground punctation becoming denser toward apex, with secondary punctures principally in basal half. Male genitalia (Figs 44E–H): T8 rather deeply, arcuately emarginate at base, ventrolateral apodemes with inner apices subparallel, separated by about three-fourths T8 width, projecting beneath to about ventral midpoint, obsolete apically, apical margin shallowly, narrowly emarginate; S8 with halves narrowly fused, more strongly sclerotized along midline and basal and apical margins, basal emargination very broad, sinuate, basal apodemes tapered, curving mediad, sides subparallel to apex, apices obliquely truncate, with inner corner slightly produced, with numerous apical setae, apical emargination broad, rounded; T9 with short, bluntly rounded basal apodemes, separated dorsally, ventrolateral apodemes narrowly but bluntly produced beneath, apices of T9 narrowly rounded to subtruncate, with single subapical seta on each side; T10 with weak apical emargination; S9 with long, narrow, medially keeled stem, head abruptly widened, sides weakly rounded to apex, basolateral margins slightly desclerotized, apices acute, widely separated, apical emargination broad, sinuate; tegmen with sides subparallel from base to about midpoint, narrowed to apex, apices subacute, tegmen moderately curved ventrad, with eversible subapical denticles near apex; median lobe about one-fourth tegmen length; basal piece about one-third tegmen length.

**Figure 45.**
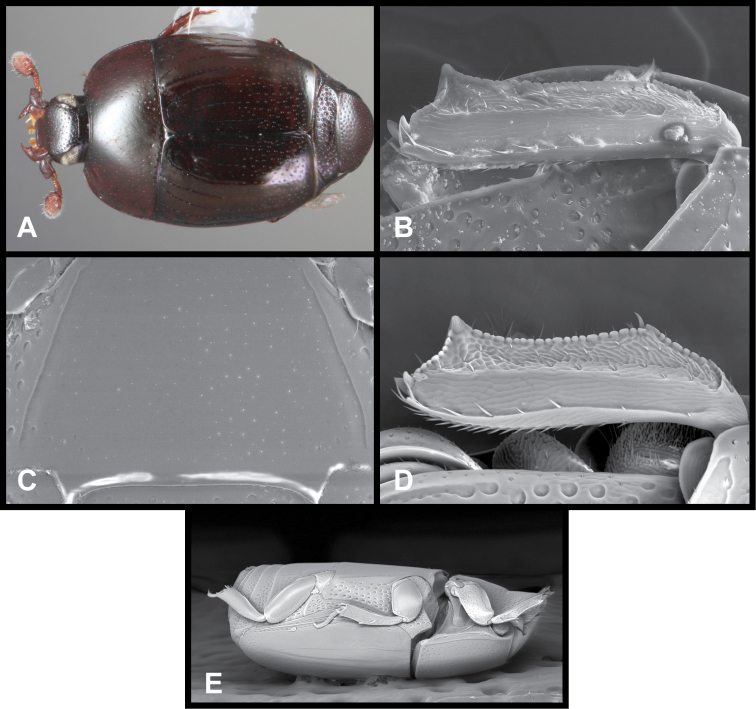
*Baconia aeneomicans* group. **A** Dorsal habitus of *Baconia plebeia* **B** Protibia of *Baconia plebeia* **C **Metaventrite of *Baconia plebeia* **D** Protibia of *Baconia castanea* **E** Ventrolateral view of *Baconia castanea*.

###### Remarks.

This species is very similar to several of the preceding, especially *Baconia slipinskii* and *Baconia mustax*, in possessing sparse punctures on the 1^st^ abdominal ventrite, a 4^th^ elytral stria which is arched mediad basally, dense punctures across the middles of the 3^rd^ and 4^th^ abdominal ventrites, and a small cluster of metaventral punctures anteromediad the metacoxa. This species can be distinguished from all others by its weak middle and basal protibial teeth (Fig. 45B), its complete marginal mesoventral stria which is usually smooth contrasting with its crenulate mesometaventral stria, and its subequal 3^rd^ and 4^th^ elytral striae, with the 5^th^ and sutural striae short and displaced progressively further posterad (Fig. 45A).

The species is somewhat variable over its considerable range, apparently covering most of Amazonia. We restrict the type series to those specimens from French Guiana to avoid the possibility of including cryptic species.

###### Etymology.

This species’ name, ‘*plebeia*’, refers to its being quite commonly collected relative to most *Baconia* species.

##### Baconia castanea sp. n.

http://zoobank.org/B56C7007-64E9-4805-86FD-4F929CE81CCE

http://species-id.net/wiki/Baconia_castanea

Figs 45DE 46A–D, I–J Map 13

###### Type locality.

**Type locality.** FRENCH GUIANA: Montagne des Chevaux [4.72°N, 52.40°W].

###### Type material.

**Holotype male**: “**GUYANE FRANÇAISE**:Montagne des Chevaux 4°43'N, 52°24'W, Piège d’interception 31 Mai 2009. SEAG leg.” / “Caterino/Tishechkin Exosternini Voucher EXO-00461” (MNHN). **Paratypes** (31): **FRENCH GUIANA**: 2:Rés. des Nouragues, Camp Inselberg, 4°05'N, 52°41'W, 25.i.2011, FIT, SEAG; 4:Rés. des Nouragues, Camp Inselberg, 4°05'N, 52°41'W, 8.x.2010, FIT, SEAG, 3:9.ix.2010, FIT, SEAG; 1:Rés. des Nouragues, Régina, 4°2.27'N, 52°40.35'W, 10.x.2009, FIT, SEAG (CHND); 1:Res. Tresor, rte. de Kaw, Pk18, 4°36.63'N, 52°16.74'W, 225 m, 13.x.2009, FIT, SEAG (CHND); 1:Belvèdére de Saül, 3°1'22"N, 53°12'34"W, 31.xi.2010, FIT, SEAG, 4:17.i.2011, FIT, SEAG, 2:20.xii.2010, FIT, SEAG, 1:4.i.2011, FIT, SEAG, 4:7.ii.2011, FIT, SEAG, 1:Montagne des Chevaux, 4°43'N, 52°24'W, 13.vi.2009, FIT, SEAG (CHND), 1:27.vi.2009, FIT, SEAG (CHND). 1: **GUYANA**: **Region 8**:Iwokrama Field Stn., Pakatau hills, 4°44'54"N, 59°1'36"W, 70 m, 25-29.v.2001, FIT, R. Brooks & Z. Falin (SEMC); 1: Kabocalli Field Stn., 4°17'4"N, 58°30'35"W, 60 m, 3-5.vi.2001, FIT, R. Brooks & Z. Falin (SEMC); 1:Kurupukari, 4°40'N, 58°40'W, ix-xi.1992, Malaise/FIT (BMNH). 1: **SURINAME**: **Pará**: nr. Overbridge River Resort, 5°31.8'N, 55°3.5'W, 15-18.ii.2010, FIT, C. Gillet, P. Skelley, W. Warner (FSCA).1: **BRAZIL**: **Mato Grosso**:Mpio. Cotriguaçu, Fazenda São Nicolau, Matinha, 9°50.3'S, 58°15.05'W, x.2009, FIT, F. Vaz-de-Mello (CEMT).

###### Diagnostic description.

**Figure 46.**
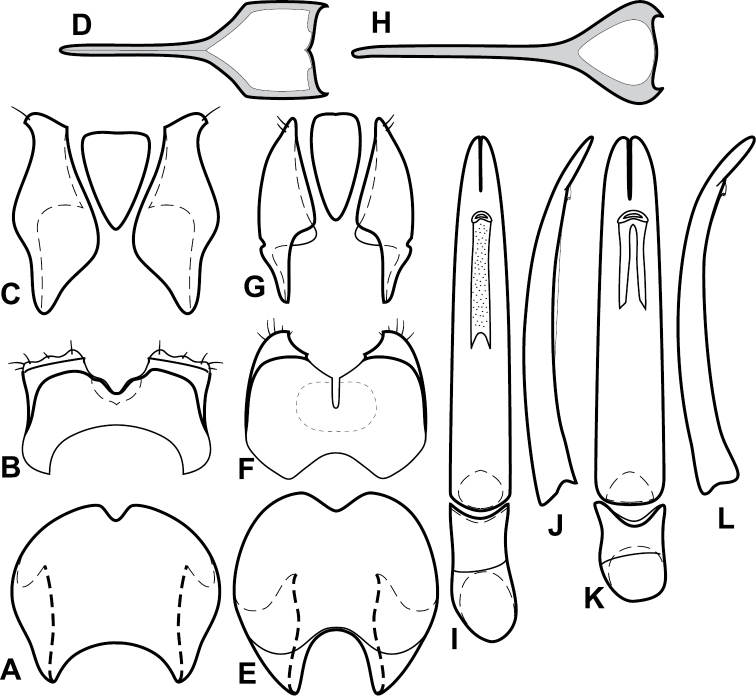
Male genitalia of *Baconia aeneomicans* group. **A** T8 of *Baconia castanea* **B** S8 of *Baconia castanea* **C **T9 & T10 of *Baconia castanea* **D** S9 of *Baconia castanea* **E** T8 of *Baconia lescheni* **F** S8 of *Baconia lescheni* **G** T9 & T10 of *Baconia lescheni* **H** S9 of *Baconia lescheni* **I** Aedeagus, dorsal view of *Baconia castanea* **J** Aedeagus, lateral view of *Baconia castanea* **K **Aedeagus, dorsal view of *Baconia lescheni* **L** Aedeagus, lateral view of *Baconia lescheni*.

Length: 1.2–1.5mm, width: 1.0–1.2mm; body broadly elongate oval, subparallel-sided, subdepressed, glabrous; color rufobrunneus, shining; head with frons more or less flat, ground punctation conspicuous, slightly denser at front and sides, with sparse coarser punctures dorsad, frontal stria absent or present only at upper corner of eye, absent across front, supraorbital stria usually absent, median fragments may be present; antennal scape short, club slightly oblong; epistoma faintly emarginate; labrum about 4×wider than long, apical margin shallowly emarginate; mandibles short, each with acute basal tooth; pronotum with sides weakly convergent in basal two-thirds, rounded to apex, lateral marginal stria descending to ventral edge of pronotum in posterior two-thirds, detached from or merging with lateral submarginal stria, which extends around anterior corners, anterior marginal stria usually detached from lateral marginal, may diverge from anterior margin behind eye, pronotal disk very weakly depressed in anterolateral corners, ground punctation fine, very sparse, coarser secondary punctures sparsely scattered in lateral thirds and further mediad along basal margin; elytra with two epipleural striae, outer subhumeral stria absent, inner subhumeral stria present in basal two-thirds, dorsal striae 1–2 similar in length, only slightly abbreviated apically, 3^rd^ and 4^th^ striae present in about basal two-thirds, 4^th^ stria curving slightly mediad at base, 5^th^ stria shorter than 4^th^, more strongly abbreviated basally, sutural stria similar in length to 5^th^, displaced slightly posterad, elytral disk with small, sparse secondary punctures in nearly apical half, extending slightly further anterad toward middle; prosternal keel weakly convex, shallowly emarginate at base, with more or less complete, carinal striae subparallel; prosternal lobe about two-thirds keel length, apical margin rounded, marginal stria fragmented to sides; mesoventrite produced at middle, with marginal stria interrupted for nearly width of prosternal keel; mesometaventral stria arched forward at middle, continuous laterally with inner lateral metaventral stria, extending posterad toward middle of metacoxa, outer lateral metaventral stria very short, oblique; metaventral disk moderately coarsely punctate at sides, impunctate at middle; abdominal ventrite 1 with single, complete inner lateral stria, lacking median discal punctures, ventrites 2–5 with fine punctures at sides, those of ventrite 4 dense across middle; protibiae bidentate, with median marginal teeth generally absent, margin serrulate; mesotibia usually with only one marginal spine; outer metatibial margin with fine denticle near midpoint; propygidium lacking basal stria, with fine ground punctation and rather dense, ocellate secondary punctures, propygidial gland openings visible about one-fourth from basal and lateral margins; pygidium with sparse ground punctation becoming slightly denser apically, with small secondary punctures conspicuous throughout. Male genitalia (Figs 46A–D, I–J): T8 broadly, shallowly emarginate at base, ventrolateral apodemes with inner apices separated by about three-fourths T8 width, projecting beneath about two-thirds its length, obsolete apically, apical margin shallowly, acutely emarginate; S8 very short, with halves fused along midline, basal emargination broad, evenly arcuate, basal apodemes widely separated, obliquely truncate, sides slightly narrowed to midline, then widened to apex, apices each with subcarinate upper edge, weakly trilobed lower edge, each lobe bearing a seta, apices separated by apical emargination about one-fourth total width; T9 with short, subacute basal apodemes, halves narrowly separated dorsally, ventrolateral apodemes subacutely produced beneath, nearly meeting, sides narrowed to near apex, but apices turned outward, outer edges rather distinctly sclerotized, with apical seta on each side; T10 short, narrowed basally, with weak apical emargination; S9 with long, narrow, medially keeled stem, head abruptly widened, sides parallel to apex, apices acute, widely separated, apical emargination broad, shallow; tegmen with sides weakly narrowed from base, apices subacute, tegmen evenly weakly curved in lateral aspect, with eversible subapical denticles ventrally; median lobe about one-fourth tegmen length; basal piece about one-third tegmen length.

###### Remarks.

The most distinctive character of *Baconia castanea* is its bidentate protibia (Fig. 45D), with the median marginal tooth strongly reduced to absent. In addition, its long inner subhumeral stria, approximately two-thirds the elytral length, is unusual in the *Baconia aeneomicans* group. The male genitalia are quite distinct, with the apical lobes of the 8^th^ sternite being strongly tridentate beneath an apicodorsal ridge, and the apices of the 9^th^ tergite bent laterad, with the typically sub-apical seta displaced to the apex.

###### Etymology.

The name of this species refers to its castaneus coloration, in contrast to the metallic coloration of many species of the genus.

##### Baconia lescheni sp. n.

http://zoobank.org/A1A6D971-FA5A-472C-9D05-5736473694CF

http://species-id.net/wiki/Baconia_lescheni

**Map 14.**
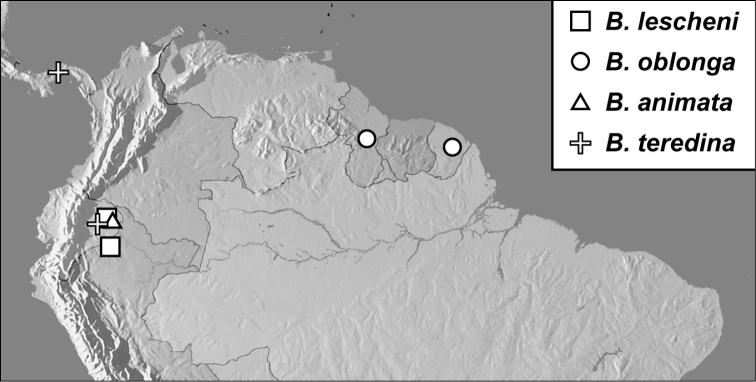
*Baconia aeneomicans* group records.

Figs 46E–H, K–L 47A–C Map 14

###### Type locality.

ECUADOR: Orellana: Yasuní Research Station [0.674°S, 76.398°W].

###### Type material.

**Holotype male**: “**ECUADOR**:Napo, Yasuní Res. Stn. on mid. Rio Tiputini. 0°40.5'S, 76°24'W, FIT#3. 5–12 Jul 1999 AKT#068 A.Tishechkin” / “LSAM0012906” (FMNH). **Paratypes** (3): **ECUADOR**:1: same locality as type, 12–20.vii.1999, FIT, A. Tishechkin (LSAM); 2: Est. Biodiv. Tiputini, 0.6376°S, 76.1499°W, 2–9.vi.2011, FIT, M. Caterino & A. Tishechkin, DNA Extract MSC-2125, EXO-00642 & MSC-2127, EXO-00629 (MSCC, FMNH).

###### Other material.

1: **PERU**: **Loreto**: Teniente Lopez, 2°35.66'S, 76°6.92'W, 210-240 m, 9.vii.1993, FIT, R. Leschen (SEMC).

###### Diagnostic description.

Length: 1.0–1.1mm, width: 0.8–0.9mm; body narrowly elongate oval, sides weakly rounded, subdepressed, glabrous; color rufescent to rufobrunneous; head with frons transversely elevated between antennal bases, weakly depressed medially, ground punctation fine and sparse, with small secondary punctures sparsely and uniformly interspersed, frontal stria at most present as very short fragment near upper edge of eye, supraorbital stria present at middle, detached; antennal scape short, club oblong, slightly expanded apically; epistoma faintly emarginate; labrum about 3×wider than long, apical margin straight; both mandibles with small, acute basal tooth; pronotum with sides weakly convergent in basal two-thirds, arcuate to apex, lateral marginal stria weak to absent, lateral submarginal stria extending around anterior corner, detached from anterior marginal stria which diverges from margin behind eyes; pronotal disk with ground punctation very fine and sparse, with few coarse secondary punctures in lateral fourths; elytra with single epipleural stria, outer and inner subhumeral striae absent, dorsal striae 1–4 complete to base, slightly progressively abbreviated apically, 4^th^ stria not curved mediad at base, 5^th^ dorsal stria present only in middle one-third, sutural stria slightly longer than 5^th^, more broadly impressed anterad, elytral disk with few coarse punctures along apical margin and scattered in posterior one-third of interstriae; prosternal keel flat, moderately broad, weakly convex, shallowly emarginate at base, carinal striae slightly divergent anterad; prosternal lobe about one-half keel length, apical margin bluntly rounded, with marginal stria obsolete at sides; mesoventrite produced at middle, with marginal stria interrupted for width of prosternal keel; mesometaventral stria subangulately arched forward to near mesoventral margin, continuous with inner lateral metaventral stria which extends obliquely posterad toward outer third of metacoxa, outer lateral metaventral stria short, oblique; metaventral disk moderately coarsely punctate at sides, impunctate at middle; abdominal ventrite 1 with single inner lateral stria, lacking coarse punctures on middle part of disk, ventrites 2–5 with sparse punctures at sides, 4^th^ ventrite with a few deep punctures across middle, the others sparsely punctate medially; protibia tridentate, the outer margin finely serrulate; mesotibia with single prominent marginal spine; outer metatibial margin smooth, lacking spines; propygidium lacking basal stria, with fine, sparse ground punctation and few slightly larger, ocellate secondary punctures irregularly scattered, propygidial gland openings inconspicuous; pygidium with fine, sparse ground punctation and few slightly coarser secondary punctures scattered in basal one-third. Male genitalia (Figs 46E–H, K–L): T8 with base narrowly, rather deeply emarginate, ventrolateral apodemes subparallel from base, with inner apices separated by about one-third T8 width, projecting beneath just beyond ventral midpoint, obsolete apically, apical margin shallowly emarginate; S8 with halves fused along midline, basal emargination shallow, basal apodemes broad, obliquely truncate, sides subparallel, weakly rounded to apices, apices narrowed, subacute, setose, moderately widely separated by deep apical emargination, S8 with weakly defined apicoventral velum; T9 with very short, subacute basal apodemes, halves narrowly separated dorsally, ventrolateral apodemes elongate, nearly meeting beneath near base, apices narrowly rounded, with a couple short, subapical setae on each side; T10 elongate, apical emargination weak; S9 with long narrow stem, head subtriangularly widened, apices acute, narrowed slightly, apical emargination broad, sinuate; tegmen with sides subparallel to just beyond midpoint, narrowed to apex, apices narrowly rounded, tegmen evenly but not strongly curved in lateral aspect, with eversible subapical denticles ventrally; median lobe about one-fourth tegmen length; basal piece about one-fourth tegmen length.

**Figure 47.**
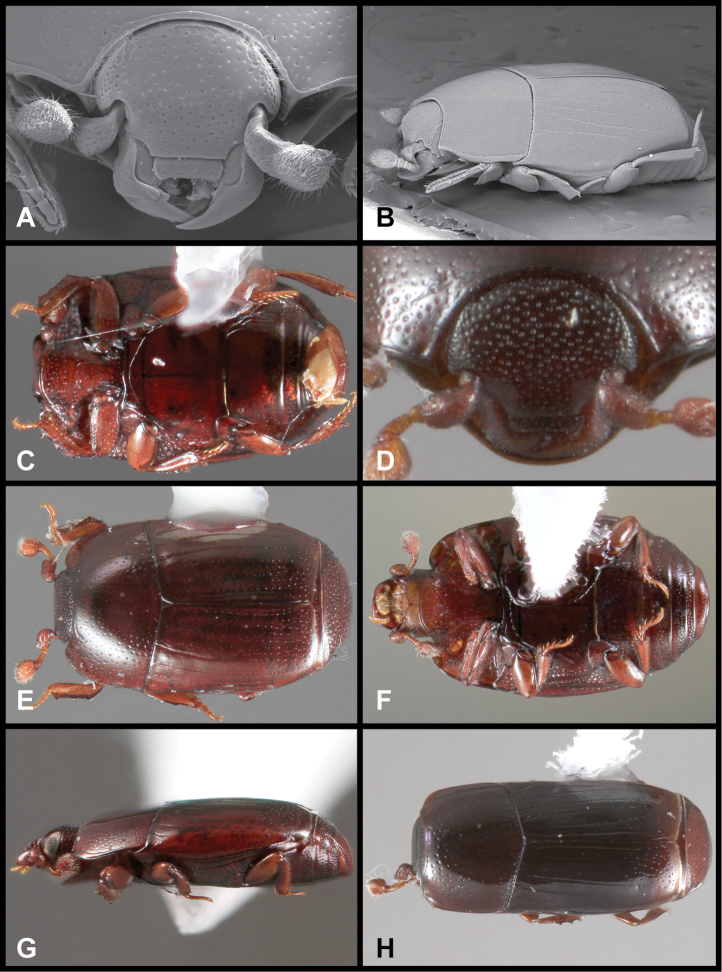
*Baconia aeneomicans* group. **A** Frons of *Baconia lescheni* **B** Lateral habitus of *Baconia lescheni* **C** Ventral habitus of *Baconia lescheni* **D** Frons of *Baconia oblonga* **E** Dorsal habitus of *Baconia oblonga* **F** Ventral habitus of *Baconia animata* **G** Lateral habitus of *Baconia animata* **H** Dorsal habitus of *Baconia teredina*.

###### Remarks.

Recognizing this species among others of the *Baconia aeneomicans* group is relatively straightforward. The most distinctive character is the frons, which is transversely elevated above the antennal bases and uniformly sparsely punctate throughout (Fig. 47A). It is also unusual in having the lateral marginal pronotal stria obsolete, a single epipleural stria (Fig. 47B), an anteriorly subangulate mesometaventral stria (Fig. 47C), and having the base of the 4^th^ elytral stria not curving mediad at the base. A single specimen from Peru agrees in all these characters, but shows some variation in elytral striation otherwise and is therefore excluded from the type series.

###### Etymology.

We have named this species in honor of Dr. Richard Leschen, of New Zealand Landcare Research, to recognize his excellent Neotropical collections that have enhanced this and other histerid studies.

##### Baconia oblonga sp. n.

http://zoobank.org/E18E8857-E2CF-4E56-9888-9D2CC7DEC142

http://species-id.net/wiki/Baconia_oblonga

Figs 47D–E 48A–F Map 14

###### Type locality.

FRENCH GUIANA: Réserve des Nouragues [4.038°N, 52.673°W].

###### Type material.

**Holotype male**: “**GUYANE FR.,** Régina, Réserve des Nouragues 4°2.27'N, 52°40.35'W, Piège d’interception 10 Oct 2009. SEAG leg.” / “Caterino/Tishechkin Exosternini Voucher EXO-00460” (MNHN). **Paratypes** (3): **FRENCH GUIANA**: 1:Rés. des Nouragues, Camp Inselberg, 4°05'N, 52°41'W, 8.x.2010, FIT, SEAG, 1: 9.ix.2010, FIT, SEAG (CHND). **GUYANA**:1: **Region 8**:Iwokrama Field Stn., Pakatau hills, 4°44'54"N, 59°1'36"W, 70 m, 25-29.v.2001, FIT, R. Brooks & Z. Falin (SEMC).

###### Diagnostic description.

**Figure 48.**
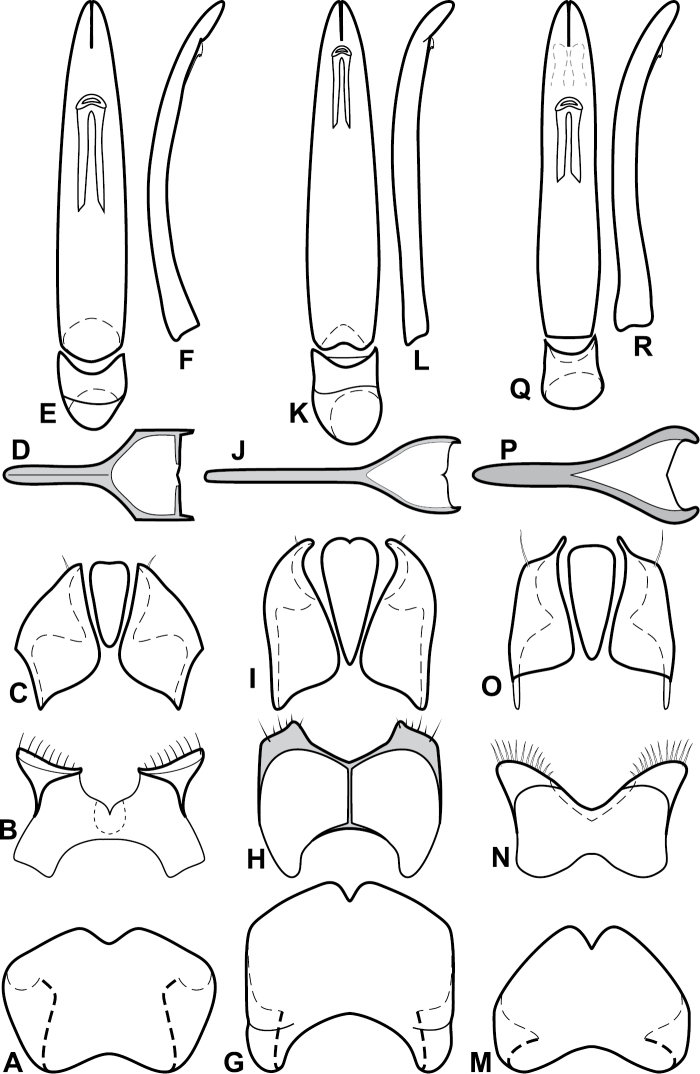
**A–F** Male genitalia of *Baconia oblonga*. **A** T8 **B** S8 **C** T9 & T10 **D** S9 **E** Aedeagus, dorsal view **F** Aedeagus, lateral view **G–L** Male genitalia of *Baconia animata* **G** T8 **H** S8 **I** T9 & T10 **J** S9 **K **Aedeagus, dorsal view **L** Aedeagus, lateral view **M–R** Male genitalia of *Baconia teredina*. **M** T8 **N** S8 **O** T9 & T10 **P** S9 **Q** Aedeagus, dorsal view **R** Aedeagus, lateral view.

Length: 1.1–1.2mm, width: 0.8–0.9mm; body narrowly elongate oval, parallel-sided, weakly depressed, glabrous; color rufescent to rufobrunneous; head with frons very shallowly depressed at middle, slightly elevated over antennal bases, ground punctation conspicuous, moderately dense, not distinct from secondary punctures, frontal stria at most present as very short fragment near upper edge of eye, supraorbital stria present; antennal scape short, club oblong, slightly expanded apically; epistoma weakly convex along apical margin, faintly emarginate, with distinct microsculpture along distal and lateral margins; labrum about 4×wider than long, apical margin slightly emarginate; both mandibles with small, acute basal tooth; pronotum with sides subparallel in basal two-thirds, weakly arcuate to apex, lateral marginal stria may merge with or be displaced by lateral submarginal stria, anterior marginal stria detached from lateral marginal, diverging from margin behind eyes, pronotal disk with coarse secondary punctures in lateral thirds and behind anterior margin, with only fine ground punctation in posterior half of middle; elytra with two epipleural striae, outer subhumeral stria absent, inner subhumeral nearly complete, may be slightly abbreviated apically, dorsal striae 1–4 complete to base, slightly, progressively abbreviated apically, 4^th^ stria not curved mediad at base, 5^th^ dorsal stria abbreviated at base, sutural stria extending further basad than 5^th^, abbreviated apically, elytral disk with few coarse punctures in apical one-third; prosternal keel flat, moderately broad, very shallowly emarginate at base, carinal striae slightly convergent anterad; prosternal lobe about two-thirds keel length, apical margin broadly rounded, with marginal stria present only at middle; mesoventrite very weakly produced at middle, with marginal stria broadly interrupted; mesometaventral stria arched strongly forward to near mesoventral margin, narrowly separated from base of inner lateral metaventral stria which extends obliquely posterad toward middle of metacoxa, outer lateral metaventral stria very short, oblique; metaventral disk moderately coarsely punctate at sides, impunctate at middle; abdominal ventrite 1 with single inner lateral stria, lacking coarse punctures on middle part of disk, ventrites 2–5 with sparse punctures at sides, 4^th^ with dense, deep punctures across middle, the other ventrites sparsely punctate medially; protibia tridentate, outer margin finely serrulate; mesotibia with two marginal spines; outer metatibial margin with fine subbasal denticle; propygidium lacking basal stria, with fine, sparse ground punctation and coarser, ocellate secondary punctures irregularly scattered, propygidial gland openings small, located about one-third behind anterior margin, one-fourth from lateral corner, the immediately surrounding disk devoid of punctures; pygidium with fine, conspicuous ground punctation slightly denser apically, and slightly coarser secondary punctures present in basal two-thirds. Male genitalia (Figs 48A–F): T8 shallowly emarginate at base, ventrolateral apodemes with inner apices separated by about two-thirds T8 width, projecting beneath just beyond ventral midpoint, obsolete apically, apical margin shallowly emarginate; S8 with halves fused along midline, basal emargination broad, shallow, basal apodemes broadly, obliquely truncate, sides strongly narrowed to beyond middle, expanded to apex, apices broadly, obliquely truncate, densely setose, separated by apical emargination about one-third total width; T9 with very short, subacute basal apodemes, halves narrowly separated dorsally, ventrolateral apodemes bluntly produced beneath, nearly meeting, apices narrowly rounded, with single subapical seta on each side; T10 elongate, narrowed basally, with weak apical emargination; S9 with long narrow, medially keeled stem, head abruptly widened, sides parallel to apex, apices acute, widely separated, apical emargination broad, shallow; tegmen with sides weakly widened from base, subparallel to just beyond midpoint, narrowed to apex, apices subacute, tegmen evenly but not strongly curved in lateral aspect, with eversible subapical denticles ventrally; median lobe about one-fourth tegmen length; basal piece about one-fourth tegmen length.

###### Remarks.

This species bears considerable resemblance to *Baconia lescheni*, above, particularly in their relatively small, narrow, subparallel-sided body form (Fig. 47E), and in the straight (not basally curved mediad) 4^th^ elytral stria. However, the nearly complete inner subhumeral stria, detached and slightly recurved anterior marginal pronotal stria, and much more densely punctate frons (Fig. 47D) will distinguish *Baconia oblonga* easily.

###### Etymology.

This species is named for its oblong body form.

##### Baconia animata sp. n.

http://zoobank.org/6F8BE123-6A3F-4EFB-B107-A1B9D2A7873B

http://species-id.net/wiki/Baconia_animata

Figs 47F–G 48G–L Map 14

###### Type locality.

ECUADOR: Orellana: Tiputini Biodiversity Station [0.635°S, 76.150°W].

###### Type material.

**Holotype male**: “**ECUADOR: Depto. Orellana,** Tiputini Biodiversity Station 0°37'55"S, 76°08'39"W 220-250m. 26 October 1998 T.L.Erwin *et al*. collectors” / “insecticidal fogging of mostly bare green leaves, some with covering of lichenous or bryophytic plants **Lot 1957 Trans. 6 Sta. 8**” / “Caterino/Tishechkin Exosternini Voucher EXO-00510” (USNM). **Paratypes** (2): same locality as type, 7.ii.1999, fogging, T. Erwin (USNM).

###### Diagnostic description.

Length: 1.1–1.2mm, width: 0.9–1.0mm; body broadly elongate oval, rather strongly depressed, glabrous; color rufobrunneus, shining; head with frons more or less flat, weakly depressed at middle, ground punctation conspicuous, slightly denser at front and sides, with few coarser punctures dorsad, frontal stria absent or present only at upper corner of eye, absent across front, supraorbital stria absent; antennal scape short, club slightly oblong; epistoma faintly emarginate; labrum about 4×wider than long, apical margin shallowly emarginate; mandibles short, each with acute basal tooth; pronotum with sides subparallel in basal two-thirds, rounded to apex, lateral marginal stria descending to ventral edge of pronotum in posterior two-thirds, detached from median part of anterior marginal stria, which diverges from margin behind eye, lateral submarginal stria present in basal four-fifths, diverging from margin toward front, pronotal disk very weakly depressed in anterolateral corners, ground punctation fine, very sparse, middle of disk impunctate with small secondary punctures in lateral fourths; elytra with two epipleural striae, outer subhumeral stria absent, inner subhumeral present only at extreme base, dorsal striae 1-2 similar in length, only slightly abbreviated apically, 3^rd^ stria present in basal half or less, 4^th^ stria slightly longer, not curving mediad at base, 5^th^ stria similar in length or slightly shorter than 4^th^, displaced slightly posterad, sutural stria slightly shorter than 5^th^, displaced further posterad, elytral disk with small secondary punctures in apical one-third; prosternal keel rather broad, flat, truncate at base, with more or less complete carinal striae subparallel or diverging slightly to front; prosternal lobe about two-thirds keel length, apical margin rounded, marginal stria present only at middle; mesoventrite broadly, shallowly emarginate, with marginal stria interrupted for nearly width of prosternal keel; mesometaventral stria broadly arched forward, continuous laterally with inner lateral metaventral stria, which extends obliquely posterad toward outer third of metacoxa, sinuous apically, outer lateral metaventral stria very short, oblique; metaventral disk moderately sparsely punctate at sides, impunctate at middle; abdominal ventrite 1 with two complete lateral striae, lacking median discal punctures, ventrites 2–5 with fine punctures at sides, those of ventrite 4 moderately dense across middle and at sides; protibiae tridentate, with median marginal denticle rather weak, margin serrulate; mesotibia with one or two small marginal spines; outer metatibial margin with fine subbasal denticle; propygidium short, wide, lacking basal stria, with sparse, fine ground punctation and ocellate secondary punctures, propygidial gland openings inconspicuous; pygidium with very sparse ground punctation, with secondary punctures sparse in basal half. Male genitalia (Figs 48G–L): T8 slightly wider than long, sides subparallel, moderately deeply, broadly emarginate at base, ventrolateral apodemes with inner apices separated by about two-thirds T8 width, projecting beneath about one-third from base, obsolete apically, apical margin shallowly emarginate; S8 almost as long as T8, halves fused and sclerotized along midline, basal emargination broad, basal apodemes bluntly rounded, sides subparallel to apex, apices obliquely truncate, densely setose, separated by apical emargination about one-half total width; T9 with very short, blunt basal apodemes, halves separated dorsally, ventrolateral apodemes bluntly produced beneath, T9 apices narrowly rounded, with single subapical seta on each side; T10 elongate, completely separating halves of T9; S9 with long narrow stem, head abruptly widened, sides rounded to apex, apices subacute, widely separated, apical emargination broad, shallow; tegmen widest in basal fourth, narrowed to apex, apices narrowly rounded, tegmen more or less straight in lateral aspect, just bent at apex, with eversible subapical denticles ventrally; median lobe about one-fourth tegmen length; basal piece about one-fourth tegmen length.

###### Remarks.

This species is among the flattest (Fig. 47G) of those in the *Baconia aeneomicans* group, and in general size and shape might almost be mistaken for a species of *Hypobletus* Schmidt. Once recognized as a *Baconia*, it can almost be recognized on body shape alone. Additionally it has a very broad prosternum, with the carinal striae diverging slightly toward the front (Fig. 47F), and elytral striae 3, 4, 5 and the sutural extend progressively further posterad, while also being progressively more abbreviated from the base, resulting in a stepwise shift posterad in position toward the suture.

###### Etymology.

This species’ name means ‘courageous’ or ‘inspired’, referring to this species’ apparent preference for novel, canopy habitats.

##### Baconia teredina sp. n.

http://zoobank.org/E0644CAE-0FBC-4D2D-B998-DAF45350DBC4

http://species-id.net/wiki/Baconia_teredina

Figs 47H 48M–R Map 14

###### Type locality.

ECUADOR: Orellana:Res. Ethnica Waorani [0.67°N, 76.43°W].

###### Type material.

**Holotype male**: “**ECUADOR: Depto. Orellana:** Res. Ethnica Waorani, 1km S Onkone Gare Camp, Trans. Ent., 0°39'26"S, 76°27'11"W, 216m, 7 July 2006, T.L. Erwin, M.C.Pimienta et al.” / “Insecticidal fogging of mostly bare green leaves, some with covering of lichenous or bryophytic plants in terra firme forest. Project MAXUS **Lot 3215 Trans. 2 Sta. 6**” / “Caterino/Tishechkin Exosternini Voucher EXO-00472” (USNM). **Paratypes** (3): same locality as type, 1: 21.i.2006, fogging, T. Erwin (USNM), 1: same locality as type except 0°39'10"S, 76°26"W, 220m 19.i.1994, fogging, T. Erwin (USNM), 1:fogging canopy, T. Erwin, DNA Extract MSC-1910, EXO-00104.

###### Other material.

**PANAMA**:1: **Colón**: P. N. San Lorenzo, STRI Crane Site, 9°17'N, 79°58'W, 15.v.2004, 17.v.2004, FIT, A. Tishechkin (GBFM).

###### Diagnostic description.

Length: 1.3–1.4mm, width: 0.6–0.7mm; body narrowly elongate, subcylindrical, glabrous; color rufescent, shining; head with frons transversely elevated between antennal bases, weakly convex above, concave below, with punctures becoming larger but sparser dorsad, frontal stria absent, supraorbital stria fragmented if present; antennal scape short, club broadly more or less circular; epistoma faintly emarginate; labrum short, about 3×wider than long, apical margin straight to shallowly emarginate; mandibles short, each with small, acute basal tooth; pronotum with sides subparallel in basal two-thirds, rounded to apex, lateral marginal stria descending to ventral edge of margin behind antennal cavity, faint to obsolete, often merging with lateral submarginal stria when present, lateral submarginal stria continuing around anterior corner to join anterior marginal stria; pronotal disk with ground punctation inconspicuous, but with secondary punctures rather large, shallow, more or less evenly scattered throughout; elytra with single, fine epipleural stria, outer and inner subhumeral striae absent, dorsal stria 1 present in basal half to two-thirds, stria 2–5 longer, nearly complete, 4^th^ stria often slightly shorter, sutural stria more or less complete, arched laterad at base, but not joined to other stria, elytral disk with small secondary punctures in apical one-fourth; prosternal keel narrow, flat, base narrowly emarginate, carinal striae separate basally, subparallel or converging slightly to front; prosternal lobe about two-thirds keel length, apical margin broadly rounded, marginal stria obsolete at sides; mesoventrite narrowly produced at middle, with marginal stria present at middle, interrupted on each side; mesometaventral stria absent from middle, inner lateral metaventral stria extending from mesometaventral suture toward inner third of metacoxa, outer lateral metaventral stria obsolete; metaventral disk sparsely punctate at sides, impunctate at middle; abdominal ventrite 1 with single complete lateral stria, central portion of disk impunctate, ventrites 2–5 with fine punctures at sides, sparser across middle; protibiae distinctly tridentate, margin only very finely serrulate between teeth; mesotibia with two marginal spines, rarely with third small spine at base; outer metatibial margin smooth; propygidium lacking basal stria, with fine, sparse ground punctation interspersed with moderately large, shallow secondary punctures separated by their diameters or slightly less, propygidial gland openings inconspicuous; pygidium with secondary punctures rather deep, sparse but denser toward apex. Male genitalia (Figs 48M–R): T8 wide, short, sides strongly narrowed to apex, basal emargination shallow, arcuate, apical emargination narrow, subacute, ventrolateral apodemes narrow, subbasal, separated by about two-thirds maximum T8 width, obsolete in apical half; S8 short, wide, halves fused, basal emargination shallow, sides widening to broadly outwardly arcuate, densely setose apices, apical guides obsolete; T9 with basal apodemes very short, thin, T9 apices narrow, acuminate, with lateral subapical seta, ventrolateral apodemes moderately well developed beneath; T10 entire; S9 stem narrow, short, widened from midpoint to arcuate apicolateral apices, stem and lateral margins of head sclerotized, apex shallowly emarginate, but not divided; tegmen widest about one-fourth from base, sides unevenly narrowed to apex, weakly constricted at middle, apex narrowly rounded, subapical foramen with accessory denticles, tegmen in lateral aspect weakly curved ventrad in most of apical three-fourths; median lobe short, wide, about one-fourth tegmen length; basal piece very short, about one-sixth tegmen length.

###### Remarks.

Externally this species appears very similar to the two in the *Baconia cylindrica* group, being narrow, elongate (Fig. 47H), and moderately convex. However, its male genitalia, bearing aedeagal denticles, and single subapical setae on each side of the 9^th^ tergite, allies it much more closely with the preceding species. Externally *Baconia teredina* can be distinguished by being slightly more depressed than *Baconia chatzimanolisi* or *Baconia cylindrica*, and it has a unique transverse frontal ridge, larger (though shallow) pronotal punctures which are more distinct toward the scutellum, anterior marginal stria continuous with the lateral marginal stria, not divergent from margin, the 4^th^ elytral stria complete, the 5^th^ and sutural striae narrowly separated at the base, and the prosternal base emarginate. One singleton from Panama is extremely similar in most respects, but has the basal two-thirds of elytra faintly metallic blue and is thus excluded from the type series.

###### Etymology.

This species’ name refers to its possible tunneling habits, given its unusually narrow body form.

##### Baconia chujoi

(Cooman, 1941)

http://species-id.net/wiki/Baconia_chujoi

**Figure 49.**
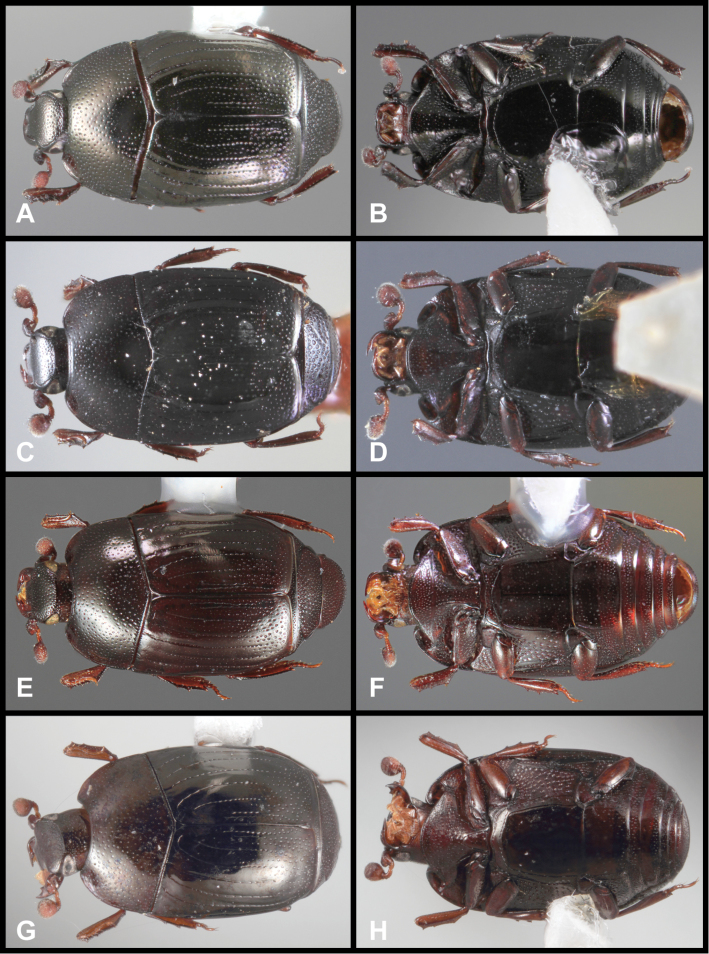
Asian species of the*Baconia aeneomicans* group. **A** Dorsal habitus of *Baconia chujoi* **B** Ventral habitus of *Baconia chujoi* **C** Dorsal habitus of holotype of *Baconia barbarus* **D** Ventral habitus of holotype of *Baconia barbarus* **E** Dorsal habitus of *Baconia reposita* **F** Ventral habitus of *Baconia reposita* **G** Dorsal habitus of *Baconia kubani* **H **Ventral habitus of *Baconia kubani*.

**Map 15.**
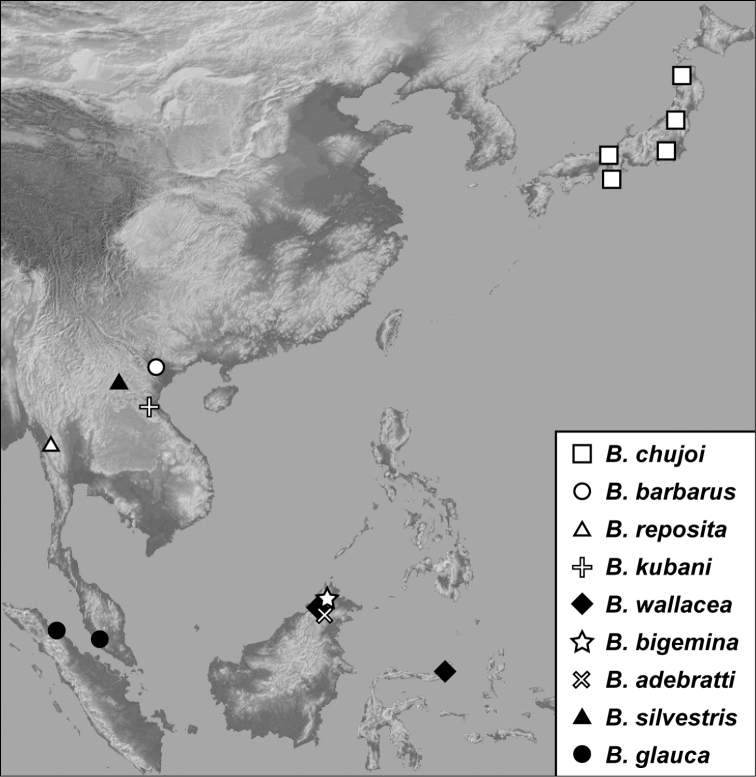
Asian *Baconia aeneomicans* group records, plus *Baconia glauca*.

Figs 49A–B 51A–C, E, G–H Map 15

Binhister chujoi Cooman, 1941: 332; *Baconia (Binhister) chujoi*: Mazur 1984: 26.

###### Type locality.

JAPAN: Tokyo: Inokashira Park [35.70°N, 139.58°E].

###### Type material.

Not found; should be in MNHN.

###### Other material.

**JAPAN**, 1: **Honshu**: Kanagawa, Kawasaki, Masukata-yama, Tama-ku, 13.v.1995, K. Kawada (CHSM); 1:Mt. Daihi, 23.vi.1951 (FMNH).

###### Diagnostic description.

Length: 1.9–2.0mm, width: 1.4–1.7mm; body elongate oval, subdepressed, glabrous; color rufopiceous to piceous, with faint metallic sheen; head with frons flat, wide, ground punctation fine, secondary punctures sparse anteriorly, markedly coarser dorsad, frontal stria present only along inner margin of eye, obsolete across middle, supraorbital stria absent; antennal scape short, club nearly circular; epistoma straight to faintly emarginate apically; labrum about 4×wider than long, weakly emarginate apically; both mandibles with strong, acute basal tooth; pronotum with sides weakly convergent, rounded to apex, lateral marginal stria descending to ventral edge in posterior half, continuous anteriorly with complete anterior marginal stria, lateral submarginal stria present in basal three-fourths, pronotal disk weakly depressed in anterolateral corners, with fine ground punctation, conspicuous secondary punctures interspersed more or less throughout, much denser toward sides; elytra with two complete epipleural striae, outer subhumeral stria absent, inner subhumeral stria present in basal one-fourth or less, occasionally also as apical fragment, dorsal striae 1-4 complete to base, apex progressively shortened mediad, 4^th^ arched mediad in front, stria 5 slightly abbreviated at base and apex, sutural stria present in middle one-third, abbreviated at base and apex, elytral disk with scattered secondary punctures in apical one-third; prosternum moderately broad, keel weakly emarginate at base, with carinal striae complete, separate, weakly convergent in basal one-fourth, weakly divergent anterad; prosternal lobe slightly over half keel length, apical margin bluntly rounded, with marginal stria more or less complete; mesoventrite weakly produced at middle, with marginal stria absent from middle one-third; mesometaventral suture weakly arched forward, mesometaventral stria arched more strongly forward, rounded to subangulate at middle, continuous laterally with oblique lateral metaventral stria, metaventral disk coarsely punctate at sides, impunctate at middle; abdominal ventrite 1 with complete inner lateral stria and fragments of outer lateral behind metacoxa, disk with coarse secondary punctures only laterad stria, ventrites 2–5 with sparse secondary punctures at sides, the punctures becoming almost obsolete at middle; protibia tridentate, the middle tooth closer to the apical than the basal, outer margin serrulate between teeth; mesotibia with two very weak marginal spines; outer metatibial margin smooth, edentate; propygidium lacking basal stria, with sparse, fine ground punctation, with coarse secondary punctures evenly interspersed, propygidial gland openings inconspicuous; pygidium with sparse ground punctation and coarser, secondary punctation denser toward base. Male genitalia (Figs 51A–C, E, G–H): T8 with base narrowly, rather deeply emarginate, ventrolateral apodemes with inner apices separated by about one-half T8 width, projecting beneath nearly to ventral midpoint, obsolete apically, apical margin shallowly emarginate; S8 with halves fused along midline, basal emargination broad, narrowed to subacute, basal apodemes narrowly rounded, subparallel, weakly arcuate, apices narrow, obliquely truncate, setose, widely separated by deep, acute apical emargination; T9 with short basal apodemes, halves well separated dorsally, ventrolateral apodemes bluntly produced beneath, nearly meeting, apices narrowly rounded, with single subapical seta on each side; T10 elongate, apical emargination not well defined; S9 with long narrow, medially keeled stem, head rounded to near apex, apices subacute, apical emargination broad, sinuate; tegmen with sides subparallel in basal half, weakly narrowed to apex, apices narrowly rounded, tegmen evenly curved in lateral aspect; median lobe about one-fourth tegmen length, with eversible subapical denticles; basal piece about one-third tegmen length.

###### Remarks.

This and the following six species are Asian, and no attempt is made to differentiate them from American species. They probably represent a single lineage, although they are quite varied, and clear synapomorphies for such a lineage are not obvious. *Baconia chujoi* is the only species occurring in Japan, and it also differs from all other Asian species by dark bronze metallic coloration (Fig. 49A), the 4^th^ stria being barely arched at base, by the coarsely punctate pronotum, and by the presence of only a single lateral metaventral stria (Fig. 49B).

Ôhara (1989) has provided additional illustrations of the species, as well as more comprehensive documentation of its distribution, apparently only on Honshu, Japan. It is unfortunate that the type specimen cannot be located. However, as the only species of this group in Japan, its identity is not in question. The species has been found in association with fungi, including *Polystictus versicolor* (L.) and *Coriolus hirsutus* (Wulf.) Quel. (Ôhara, 1989).

##### Baconia barbarus

(Cooman, 1934)

http://species-id.net/wiki/Baconia_barbarus

Figs 49C–D Map 15

Binhister barbarus Cooman, 1934: *Baconia (Binhister) barbarus*: Mazur 1984: 26.

###### Type locality.

VIETNAM: Hoa-Binh [20.8°N, 105.3°E].

###### Type material.

**Holotype**, sex undetermined (MNHN): “Hoa-Binh, Tonkin, 1922, P.A. de Cooman”.

###### Diagnostic description.

Length: [not measured, ~1.9mm], width: [not measured, ~1.2mm]; body elongate oval, weakly convex, glabrous; color rufopiceous, with faint metallic sheen; head with frons flat, wide, ground punctation fine, secondary punctures sparse with numerous coarser punctures denser dorsad, frontal stria present only along inner margin of eye, obsolete across middle; antennal scape short, club nearly circular; apical margin of epistoma straight; labrum about 4×wider than long, weakly emarginate apically; both mandibles with strong, acute basal tooth; pronotum with sides weakly convergent, rounded to apex, lateral marginal stria merging with lateral submarginal stria one-fourth from anterior corner, continuous anteriorly with complete anterior marginal stria, pronotal disk weakly depressed in anterolateral corners, fine ground punctation interspersed with rather coarse secondary punctures throughout, only slightly sparser in front of scutellum; elytra with one complete epipleural striae, outer subhumeral stria absent, inner subhumeral stria present as short basal fragment, dorsal striae 1–4 complete to base, progressively shortened apically, 4^th^ stria arched toward sutural in front, stria 5 slightly abbreviated at base and apex, sutural stria present in middle half, abbreviated at base and apex, elytral disk with scattered secondary punctures in apical one-third; prosternum moderately broad, keel emarginate at base, with carinal striae subparallel, separate throughout; prosternal lobe about two-thirds keel length, apical margin rounded, with marginal stria present only at middle; mesoventrite weakly produced at middle, with marginal stria interrupted for width of prosternal keel; mesometaventral stria arched forward, subangulate at middle, continuous laterally with inner lateral metaventral stria which extends straight posterad to inner third of metacoxa, outer lateral metaventral stria barely indicated behind mesocoxa; metaventral disk coarsely punctate at sides, impunctate at middle; abdominal ventrite 1 with two complete lateral striae, disk with only very fine ground punctation; protibia tridentate, the teeth rather evenly spaced, weak, outer margin serrulate between teeth; mesotibia with two weak marginal spines; outer metatibial margin smooth, edentate; propygidium lacking basal stria, with sparse, fine ground punctation, with coarse secondary punctures evenly interspersed, propygidial gland openings evident, located about one-third behind anterior margin, about one-fourth width from each lateral margin; pygidium with sparse ground punctation and slightly coarser, secondary punctation denser toward base. Male genitalia: not known.

###### Remarks.

*Baconia barbarus* is known only from the type specimen (Figs 49C–D), of undetermined sex, but it may be adequately diagnosed on external characters from several close relatives. It shares the marginal mesoventral stria being widely interrupted by the fine, anteriorly subangulate mesometaventral stria (Fig. 49D) only with *Baconia reposita*, below. In that species there is only a single lateral stria on the 1^st^ abdominal ventrite, whereas *Baconia barbarus* has two, and the outer lateral metaventral stria of *Baconia reposita* is distinct and present for about one-third the length of the metaventrite, while being much shorter in *Baconia barbarus*.

##### Baconia reposita sp. n.

http://zoobank.org/7490B30C-881B-46DD-8B9D-25766814D2E2

http://species-id.net/wiki/Baconia_reposita

Figs 49E–F Map 15

###### Type locality.

THAILAND: Thung Yai Wildlife Sanctuary [15.50°N, 98.80°E].

###### Type material.

**Holotype female**: “W.THAILAND: 300m., Thung Yai Wildlife Sanctuary. 15°30'N - 98°48'E” / “Tak Province, Umphang District, Mae Chan/Mae Klong confluence. 27.iv–6.v.1988” / “oak/bamboo forest. M.J.D.Brendell. B.M.1988-183” / “Caterino/Tishechkin Exosternini Voucher EXO-00649” (BMNH). **Paratypes** (4): 1: same data as type; 3: **THAILAND**: **Tak**: Umphang, Thung Yai Wildlife Sanctuary, Song Bae Stream, 15°28'N, 98°48'E, 300 m, 18–27.iv.1988, evergreen rainforest, M. Brendell (BMNH, MSCC).

###### Diagnostic description.

Length: 1.7–1.8mm, width: 1.2–1.3mm; body elongate oval, subdepressed, glabrous; color darkly rufopiceous, with faint bronzy sheen; head with frons flat, wide, sparsely punctate, punctures slightly denser dorsad, frontal stria present only along inner margin of eye, obsolete across middle, supraorbital stria absent; antennal scape short, club slightly oblong; epistoma straight to faintly emarginate apically; labrum about 4×wider than long, weakly emarginate apically; both mandibles with strong, acute basal tooth; pronotum with sides weakly convergent, rounded to apex, lateral marginal stria complete, continuous anteriorly with complete anterior marginal stria, marginal bead moderately wide, lateral submarginal stria present in basal three-fourths, pronotal disk weakly depressed in anterolateral corners, with fine ground punctation and coarse secondary punctures almost uniformly interspersed, slightly sparser toward antescutellar region; elytra with two complete epipleural striae, outer subhumeral stria absent, inner absent or weakly impressed at extreme base, dorsal striae 1–4 complete to base, progressively shortened apically, 4^th^ stria distinctly arched toward base of 5^th^ in front, stria 5 slightly abbreviated at base and apex, sutural stria present in middle one-third, abbreviated at base and apex, elytral disk with scattered secondary punctures in apical one-third, extending slightly further basad toward suture; prosternum moderately broad, keel weakly emarginate at base, with carinal striae extending nearly to anterior margin, separate; prosternal lobe about two-thirds keel length, apical margin broadly rounded, with marginal stria well impressed only at middle; mesoventrite somewhat broadly emarginate, but weakly produced at middle, with marginal stria interrupted for width of prosternal keel; mesometaventral stria arched forward, subangulate at middle, continuous laterally with inner lateral metaventral stria which extends straight posterad to inner corner of metacoxa, outer lateral metaventral stria short, slightly oblique; metaventral disk coarsely punctate at sides, impunctate at middle; abdominal ventrite 1 with single lateral stria, disk with coarse secondary punctures at sides and fine secondary punctures across posterior margin of middle portion, ventrites 2–5 with sparse secondary punctures at sides, the punctures becoming sparser and finer at middle; protibia tridentate, the middle tooth closer to apical than basal, outer margin serrulate between teeth; mesotibia with two very weak marginal spines; outer metatibial margin smooth, edentate; propygidium lacking basal stria, with sparse, fine ground punctation, with secondary punctures larger and denser toward base, propygidial gland openings thin, elongate, located about one-third behind anterior margin, about one-fourth width from each lateral margin, propygidium impunctate in immediate vicinity; pygidium with sparse ground punctation and slightly coarser, secondary punctation denser toward base. Male: not known.

###### Remarks.

This species can be distinguished from the other Asian species by the fine, anteriorly angulate mesometaventral stria displacing the middle part of the marginal mesoventral stria (Fig. 49F), the short outer lateral metaventral stria, and the almost uniformly punctate pronotum (Fig. 49E). It is very closely related to *Baconia barbarus*, as discussed above, but is consistent in the differences noted. Discovery of males of both species would greatly help confirm their distinctness.

###### Etymology.

This species name means ‘far away’ or ‘remote’, referring to this Asian outpost of *Baconia* diversity.

##### Baconia kubani sp. n.

http://zoobank.org/9DC3C6A9-D9CC-474D-A167-F2A78EA152BE

http://species-id.net/wiki/Baconia_kubani

Figs 49G–H Map 15

###### Type locality.

LAOS: Bolikhamxai: 8 km NE Ban Nape [18.35°N, 105.13°E].

###### Type material.

**Holotype female**: “LAOS, 1–18.v.2001, Bolikhamxai prov., 18°21'N, 105°08'E, Ban Nape (8 km NE) ~600m, V. Kubáň leg.” / “*Binhister barbarus*, Det. S. Mazur” (MNHG).

###### Diagnostic description.

Length: 2.0mm, width: 1.5mm; body elongate oval, weakly convex, glabrous; color rufopiceous to piceous, with faint metallic sheen; head with frons flat, wide, ground punctation fine, secondary punctures sparse with only few coarser punctures dorsad, frontal stria present only along inner margin of eye, obsolete across middle, supraorbital stria present, meeting frontal stria at sides; antennal scape short, club nearly circular; epistoma straight to faintly emarginate apically; labrum about 4×wider than long, weakly emarginate apically; both mandibles with strong, acute basal tooth; pronotum with sides weakly convergent, rounded to apex, lateral marginal stria descending to ventral edge in posterior half, continuous anteriorly with complete anterior marginal stria, lateral submarginal stria present in basal three-fourths, pronotal disk weakly depressed in anterolateral corners, with fine ground punctation, conspicuous secondary punctures interspersed more or less throughout, slightly denser toward sides; elytra with two complete epipleural striae, outer subhumeral stria absent, inner present in basal one-fourth or less, dorsal striae 1-4 complete to base, progressively shortened apically, 4^th^ stria distinctly arched to sutural in front, stria 5 slightly abbreviated at base and apex, sutural stria present in middle one-fourth, abbreviated at base and apex, elytral disk with scattered secondary punctures in apical one-third; prosternum moderately broad, keel weakly emarginate at base, with carinal striae shortened, joined in anterior arch near midpoint; prosternal lobe about two-thirds keel length, apical margin bluntly rounded, with marginal stria present only at middle; mesoventrite weakly produced at middle, with marginal stria complete or weakly interrupted at middle; mesometaventral stria arched forward, subangulate at middle, continuous laterally with inner lateral metaventral stria, which extends straight posterad to inner corner of metacoxa, outer lateral metaventral stria present, parallel to inner stria, complete; metaventral disk coarsely punctate at sides, impunctate at middle; abdominal ventrite 1 with two complete lateral striae, disk with coarse secondary punctures at sides and in posterior corners of middle portion, ventrites 2–5 with sparse secondary punctures at sides, the punctures becoming sparser and finer at middle; protibia tridentate, the middle tooth closer to apical than basal, outer margin serrulate between teeth; mesotibia with two very weak marginal spines; outer metatibial margin smooth, edentate; propygidium lacking basal stria, with sparse, fine ground punctation, with coarse secondary punctures evenly interspersed, propygidial gland openings narrow, obliquely elongate, located about one-third behind anterior margin, about one-fourth width from each lateral margin; pygidium with sparse ground punctation and slightly coarser, secondary punctation denser toward base. Male: not known.

###### Remarks.

The holotype of *Baconia kubani* was initially identified as a specimen of *Baconia barbarus*, and they are very similar. However, a combination of characters distinguish it quite clearly: more uniform pronotal punctation (Fig. 49G), slightly more abbreviated elytral striae, presence of two complete lateral metaventral striae, and shortened prosternal carinal striae that are united at the middle of the prosternal keel (Fig. 49H). In fact the united prosternal keel striae are shared only with *Baconia adebratti*, which is easily distinguished by the inner metaventral stria curving strongly mediad posteriorly.

###### Etymology.

We name this species in honor of its collector (as well as one other new species treated here), the Czech buprestid specialist Vítězslav Kubáň.

##### Baconia wallacea sp. n.

http://zoobank.org/21302B13-98E0-48E7-8934-7918E493AB0B

http://species-id.net/wiki/Baconia_wallacea

Figs 50A–B Map 15

###### Type locality.

INDONESIA: Sulawesi Utara: Dumoga-Bone National Park [exact locality uncertain].

###### Type material.

**Holotype female**: “**INDONESIA**: **Sulawesi Utara**, Dumoga-BoneNP, Plot A, lowland forest, Oct. 1985, FIT, ca. 200m” / “R.Ent.Soc.Lond. PROJECT WALLACE, B.M. 1985-10” / “Caterino/Tishechkin Exosternini Voucher EXO-02560” (BMNH). **Paratype** (1): same locality as holotype, 400 m, iii.1985, FIT (BMNH).

###### Other material.

**MALAYSIA: Sabah**: Sipitang, Mendolong, 11.v.1988, S. Adebratt (MNHG).

###### Diagnostic description.

Length: 1.8–1.9mm, width: 1.3–1.4mm; body elongate oval, weakly convex, glabrous; color rufobrunneus; head with frons flat, wide, ground punctation fine, secondary punctures sparse with coarser punctures dorsad, frontal stria present only along inner margin of eye, obsolete across middle, supraorbital stria present; antennal scape short, club nearly circular; epistoma straight to faintly emarginate apically; labrum about 4×wider than long, weakly emarginate apically; both mandibles with strong, acute basal tooth; pronotum with sides weakly convergent, rounded to apex, lateral marginal stria descending to ventral edge in posterior half, continuous anteriorly with complete anterior marginal stria, lateral submarginal stria present in basal four-fifths, pronotal disk weakly depressed in anterolateral corners, with fine ground punctation, conspicuous secondary punctures interspersed at sides, becoming larger (though very shallow) toward prescutellar area; elytra with two complete epipleural striae, outer subhumeral stria absent, inner present in basal one-fifth or less, dorsal striae 1–4 complete to base, vaguely abbreviated apically, 4^th^ stria arched to sutural in front, recurved slightly posterad along extreme anterior part of suture, stria 5 slightly abbreviated at base and apex, curving inward at front, sutural stria present in middle one-half, abbreviated at base and apex, elytral disk with scattered secondary punctures in apical one-fourth; prosternum moderately broad, keel weakly emarginate at base, with carinal striae complete, subparallel; prosternal lobe about two-thirds keel length, apical margin bluntly rounded, with marginal stria present only at middle; mesoventrite weakly produced at middle, with marginal stria complete; mesometaventral stria transverse, continuous laterally with inner lateral metaventral stria which extends straight posterad to inner corner of metacoxa, recurved mediad at apex, outer lateral metaventral stria present, parallel to inner stria, complete; metaventral disk coarsely punctate at sides, impunctate at middle; abdominal ventrite 1 with single, abbreviated lateral stria, small fragment of outer stria may be present, disk with few coarse secondary punctures along anterior margin, ventrites 2–5 with sparse punctures at sides, impunctate at middle; protibia tridentate, the middle tooth weak, closer to apical than basal, outer margin serrulate between teeth; mesotibia with two very weak marginal spines; outer metatibial margin smooth, edentate; propygidium lacking basal stria, with sparse, fine ground punctation, with moderately coarser secondary punctures evenly interspersed, propygidial gland openings narrow, obliquely elongate, located about one-third behind anterior margin, about one-fourth width from each lateral margin; pygidium with sparse ground punctation and slightly coarser, secondary punctation. Male: not known.

**Figure 50.**
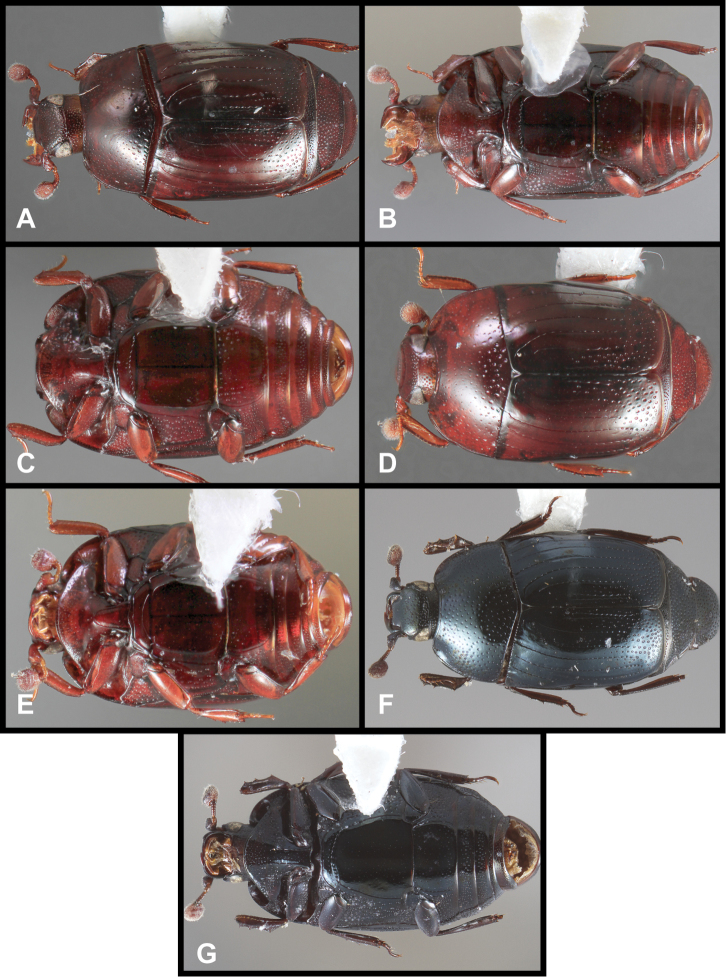
Asian species of the*Baconia aeneomicans* group. **A** Dorsal habitus of *Baconia wallacea* **B** Ventral habitus of *Baconia wallacea* **C** Ventral habitus of *Baconia bigemina* **D** Dorsal habitus of *Baconia adebratti* **E** Ventral habitus of *Baconia adebratti* **F** Dorsal habitus of *Baconia silvestris* **G** Ventral habitus of *Baconia silvestris*.

###### Remarks.

This species can be distinguished from the others in the Asian group by its shortened, separate prosternal striae (Fig. 50B), the presence of two complete lateral metaventral striae which are curved mediad apically, and by the pattern of pronotal punctation in which a median band of the pronotal disk is impunctate, but large, shallow prescutellar punctures are present on each side (Fig. 50A). The single specimen from Sabah, Malaysia has a more thoroughly punctate pronotum, and differs slightly in ventral striation, with the inner metaventral stria less distinctly curved inward at the apex, and we therefore exclude it from the type series.

###### Etymology.

This species name refers to both the region of Wallacea and to the Natural History Museum’s 1985 Project Wallace expedition, which produced so many interesting Histeridae.

##### Baconia bigemina sp. n.

http://zoobank.org/9DAEB925-D0B3-433F-829E-FAC104D67AE4

http://species-id.net/wiki/Baconia_bigemina

Figs 50C Map 15

###### Type locality.

MALAYSIA: Sabah: Poring Hot Springs [6.04°N, 116.70°E].

###### Type material.

**Holotype male**: “SABAH: Poring Hot Springs, 500 m 6.V.1987 Burckhardt – Löbl” / “Caterino/Tishechkin Exosternini Voucher EXO-01772” (MHNG).

###### Diagnostic description.

**Figure 51.**
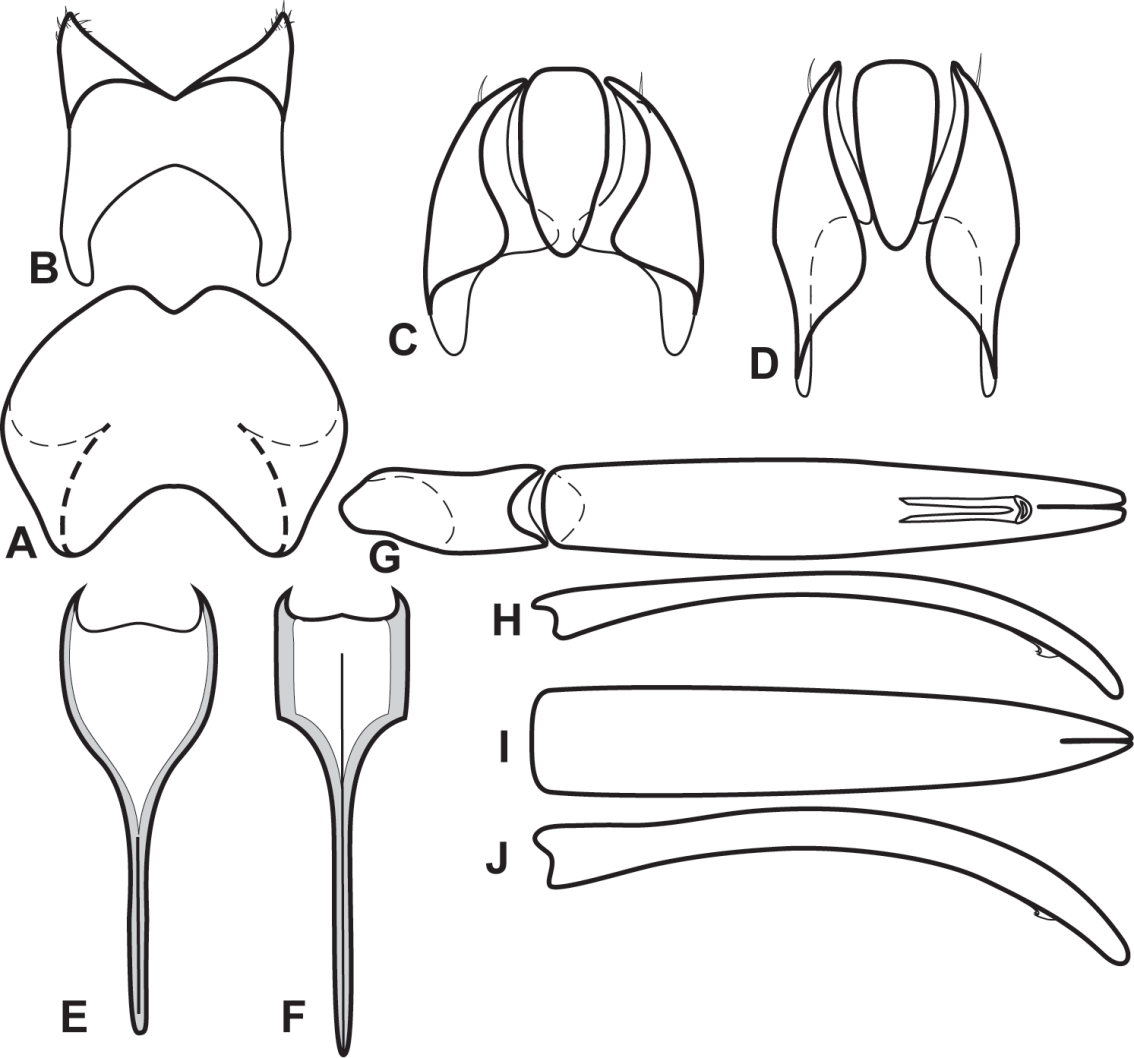
Male genitalia of *Baconia aeneomicans* group. **A** T8 of *Baconia chujoi* **B** S8 of *Baconia chujoi* **C** T9 & T10 of *Baconia chujoi* **D** T9 & T10 of *Baconia adebratti* **E** S9 of *Baconia chujoi* **F** S9 of *Baconia adebratti* **G** Aedeagus, dorsal view of *Baconia chujoi* **H** Aedeagus, lateral view of *Baconia chujoi* **I** Aedeagus, dorsal view of *Baconia adebratti* **J** Aedeagus, lateral view of *Baconia adebratti*.

Length: 1.8mm, width: 1.3mm; body elongate oval, weakly convex, glabrous; color rufobrunneus; head with frons flat, wide, ground punctation fine, secondary punctures sparse with coarser punctures dorsad, frontal stria present only along inner margin of eye, obsolete across middle, supraorbital stria present; antennal scape short, club nearly circular; epistoma straight to faintly emarginate apically; labrum about 4×wider than long, weakly emarginate apically; both mandibles with strong, acute basal tooth; pronotum with sides weakly convergent, rounded to apex, lateral marginal stria descending to ventral edge in posterior half, continuous anteriorly with complete anterior marginal stria, lateral submarginal stria present in basal three-fourths, pronotal disk weakly depressed in anterolateral corners, with fine ground punctation, conspicuous secondary punctures interspersed throughout, becoming vaguely larger toward prescutellar area; elytra with two complete epipleural striae, outer subhumeral stria absent, inner subhumeral stria present in basal one-fifth or less, dorsal striae 1–4 complete to base, vaguely abbreviated apically, 4^th^ stria arched to sutural in front, recurved slightly posterad along extreme anterior part of suture, stria 5 extending anteriorly into basal arch of 4^th^, sutural stria present in middle one-half, abbreviated at base and apex, elytral disk with scattered secondary punctures in apical one-fourth; prosternum moderately broad, keel weakly emarginate at base, convex, smooth between fine, complete carinal striae, carinal striae divergent basally and apically; prosternal lobe about one-half keel length, apical margin rounded, slightly deflexed, with marginal stria present only at middle; mesoventrite weakly produced at middle, with marginal stria complete; mesometaventral stria transverse, continuous laterally with inner lateral metaventral stria which extends straight posterad to inner corner of metacoxa, outer lateral metaventral stria present, parallel to inner stria, complete; metaventral disk coarsely punctate at sides, impunctate at middle; abdominal ventrite 1 with complete inner lateral stria, small fragment of outer stria may be present, disk with few coarse secondary punctures along anterior margin, ventrites 2–5 with sparse punctures at sides, finer but conspicuous across middle; protibia tridentate, the middle tooth very reduced, closer to apical tooth than basal, outer margin serrulate between teeth; mesotibia with two very weak marginal spines; outer metatibial margin smooth, edentate; propygidium lacking basal stria, with sparse, fine ground punctation, with moderately coarser secondary punctures evenly interspersed, propygidial gland openings narrow, obliquely elongate, located about one-half behind anterior margin, about one-fourth pygidial width from each lateral margin; pygidium with sparse ground punctation becoming denser apically, with slightly coarser secondary punctation denser toward base. Male genitalia essentially indistinguishable from that of *Baconia chujoi* (see Fig. 51).

###### Remarks.

This species is very similar to the preceding, *Baconia wallacea*, but differs in having the inner lateral metasternal stria straight to the apex (Fig. 50C), not curved mediad, the outer lateral metaventral stria incomplete, and the prosternal striae complete and separate anteriorly.

###### Etymology.

The name of this species means ‘doubled’, referring to the metaventral striae, though this is not unique to the species.

##### Baconia adebratti sp. n.

http://zoobank.org/0F05BD69-4B2A-4E14-A3C3-FAD439E69085

http://species-id.net/wiki/Baconia_adebratti

Figs 50D–E 51D, F, I–J Map 15

###### Type locality.

Malaysia: Sabah: Sipitang: Mendolong [exact locality uncertain].

###### Type material.

**Holotype male**: “Malaysia: Sabah,Sipitang, Mendolong. T6/R, 31.III.1989, leg. S. Adebratt” / “Caterino/Tishechkin Exosternini Voucher EXO-01771” (LUND).

###### Diagnostic description.

Length: 1.5mm, width: 1.2mm; body elongate oval, moderately convex, glabrous; color rufobrunneus; head with frons flat, wide, ground punctation fine, secondary punctures sparse with coarser punctures dorsad, frontal stria present only along inner margin of eye, obsolete across middle, supraorbital stria absent; antennal scape short, club nearly circular; epistoma faintly emarginate apically; labrum about 3×wider than long, weakly emarginate apically; both mandibles with strong, acute basal tooth; pronotum with sides weakly convergent, rounded to apex, lateral marginal stria descending to ventral edge in posterior half, continuous anteriorly with complete anterior marginal stria, lateral submarginal stria present in basal four-fifths, pronotal disk weakly depressed in anterolateral corners, ground punctation fine, with conspicuous secondary punctures interspersed throughout, becoming larger and denser toward sides and prescutellar area; elytra with two epipleural striae, outer subhumeral stria absent, inner present in basal one-fifth or less, dorsal striae 1-4 complete to base, vaguely abbreviated apically, 4^th^ stria arched to sutural in front, recurved slightly posterad along extreme anterior part of suture, stria 5 slightly extending anteriorly into basal arch of 4^th^, sutural stria present in middle one-third, abbreviated at base and apex, elytral disk with scattered secondary punctures in apical one-fourth, extending anterad between 5^th^ and sutural striae; prosternum moderately broad, keel shallowly emarginate at base, convex, smooth, carinal striae convergent anterad, joined in broad anterior arc just beyond midpoint; prosternal lobe about two-thirds keel length, apical margin rounded, slightly deflexed, with marginal stria present only at middle; mesoventrite produced at middle, with marginal stria complete; mesometaventral stria arched forward, detached laterally from inner lateral metaventral stria which extends straight posterad to inner corner of metacoxa, curving mediad at posterior apex, outer lateral metaventral stria present, parallel to inner stria, complete; metaventral disk moderately coarsely punctate at sides, impunctate at middle; abdominal ventrite 1 with complete inner lateral stria, outer stria absent, ventrites 2–5 with sparse punctures at sides, much finer across middle; protibia with very weak marginal spines, more or less linear, its outer margin serrulate; mesotibia with two very weak marginal spines; outer metatibial margin smooth, edentate; propygidium lacking basal stria, with sparse, fine ground punctation, with moderately coarser secondary punctures uniformly interspersed, propygidial gland openings inconspicuous; pygidium with sparse ground punctation becoming denser apically, with slightly coarser secondary punctation denser toward base. Male genitalia (Figs 51D, F, I–J): T8 with base narrowly, rather deeply emarginate, ventrolateral apodemes with inner apices separated by about one-half T8 width, projecting beneath nearly to ventral midpoint, obsolete apically, apical margin shallowly emarginate; S8 with halves fused along midline, basal emargination broad, narrowed to subacute, basal apodemes narrowly rounded, sides subparallel, weakly arcuate, apices narrow, obliquely truncate, setose, widely separated by deep, acute apical emargination; T9 with short basal apodemes, halves well separated dorsally, ventrolateral apodemes bluntly produced beneath, nearly meeting, apices narrowly rounded, with single subapical seta on each side; T10 elongate; S9 with long narrow, medially keeled stem, head abruptly widened near apical one-fourth, sides parallel to subacute apices, apical emargination broad, shallow, sinuate; tegmen with sides slightly widened from base, subparallel in most of two-thirds, weakly narrowed to apex, apices narrowly rounded, tegmen evenly curved in lateral aspect; median lobe about one-fourth tegmen length, with eversible subapical denticles; basal piece about one-third tegmen length.

###### Remarks.

This species is very similar to *Baconia wallacea*, in that the inner lateral metaventral stria is strongly curved mediad at the apex, and it has large, shallow punctures in antescutellar area. The united prosternal striae (Fig. 50E) of *Baconia adebratti* will distinguish it, and it also differs in body shape, being wider toward the front (Fig. 50D), and in protibial dentation, with the protibia of *Baconia adebratti* more or less lacking marginal denticles.

###### Etymology.

This species is named in honor of Swedish entomologist Stig Adebratt (1927–2002), whose voluminous collections in Borneo resulted in numerous new histerid species.

##### Baconia silvestris sp. n.

http://zoobank.org/DDA152A3-9904-40CC-9B9D-366CC5E07A81

http://species-id.net/wiki/Baconia_silvestris

Figs 50F–G Map 15

###### Type locality.

LAOS: Xieng Khouang: Ban Na Lam-Phou Sane Mt [19.63°N, 103.34°E].

###### Type material.

**Holotype female**: “LAOS-NE, Xieng Khouang prov., 19°37.8'N, 103°20.1'E, 30km NE Phonsavan : Ban Na Lam-Phou Sane Mt., 1300–1700 m 10–30.v.2009, M.Geiser leg.” / “NHMB Basel, NMPC Prague Laos 2009 Expedition: M. Brancucci, M. Geiser, Z. Kraus, D. Hauck, V. Kuban” / “Caterino/Tishechkin Exosternini Voucher EXO-02564” (NHMB).

###### Diagnostic description.

Length: 2.0mm, width: 1.4mm; body elongate oval, weakly convex, glabrous; color dark metallic blue dorsally, with venter only faintly metallic; head with frons wide, weakly convex across middle, ground punctation fine, with sparse secondary punctures slightly denser dorsad, frontal stria present only along inner margin of eye, obsolete across middle, supraorbital stria present, detached from frontal stria at sides; antennal scape short, club nearly circular; epistoma faintly emarginate apically; labrum about 4×wider than long, weakly emarginate apically; both mandibles short with strong, acute basal tooth; pronotum with sides weakly rounded to apex, lateral marginal stria descending to ventral edge in posterior half, anterior marginal stria complete, lateral submarginal stria present, more or less complete, may be joined to anterior marginal stria in front, pronotal disk weakly depressed in anterolateral corners, with fine ground punctation, conspicuous secondary punctures interspersed more or less throughout, slightly larger toward antescutellar region; elytra with two complete epipleural striae, outer subhumeral stria absent, inner present in basal one-fourth or less, dorsal striae 1–4 complete to base, progressively shortened apically, 4^th^ stria distinctly arched to sutural in front, stria 5 slightly abbreviated at base and apex, sutural stria present in middle one-fourth, abbreviated at base and apex, elytral disk with scattered secondary punctures in apical one-third, extending further basad toward suture; prosternal keel weakly emarginate at base, with carinal striae parallel, separate; prosternal lobe about two-thirds keel length, apical margin broadly rounded, with marginal stria present only at middle; mesoventrite weakly produced at middle, with marginal stria complete or weakly interrupted at middle; mesometaventral stria arched forward, continuous laterally with inner lateral metaventral stria which extends slightly obliquely posterad to inner third of metacoxa, outer lateral metaventral stria short, oblique; metaventral disk coarsely punctate at sides, impunctate at middle; abdominal ventrite 1 with inner lateral stria complete, outer abbreviated, disk with coarse secondary punctures at sides and fine punctures in posterior corners of middle portion, ventrites 2–5 with sparse secondary punctures at sides, punctures becoming sparser and finer at middle; protibia tridentate, middle tooth closer to apical than basal, outer margin serrulate between teeth; mesotibia with two very weak marginal spines; outer metatibial margin smooth, edentate; propygidium lacking basal stria, with sparse, fine ground punctation and secondary punctures evenly interspersed, propygidial gland slightly elongate, located about one-third behind anterior margin, and about one-third from each lateral margin; pygidium with sparse ground punctation and coarser, secondary punctation. Male: not known.

###### Remarks.

This is the only one of the Asian *Baconia aeneomicans* group species to exhibit distinctly blue metallic coloration (Fig. 50F; but see *Baconia glauca*, under incertae sedis, below), and it can easily be recognized by this alone. In addition it has uniformly coarse pronotal punctation, complete, separate and subparallel prosternal striae (Fig. 50G), and a short, oblique outer lateral metaventral stria.

###### Etymology.

This species’ name refers to the ‘forests’ from which it came.

#### *Baconia cylindrica* group

**Figure 52.**
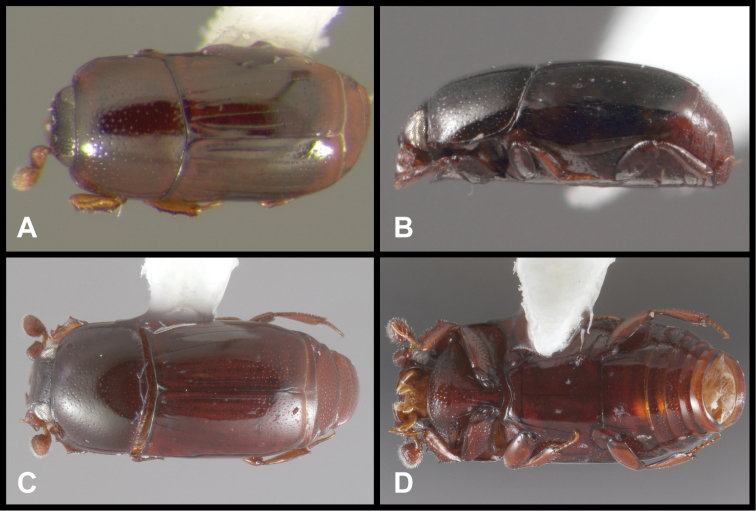
*Baconia cylindrica* group. **A** Dorsal habitus of *Baconia cylindrica* **B** Lateral habitus of *Baconia cylindrica* **C** Dorsal habitus of *Baconia chatzimanolisi* **D** Ventral habitus of *Baconia chatzimanolisi*.

This species group contains only two quite similar, distinctive species. As the name of the nominate species suggests, they have a strongly cylindrical body form, contrasting with the typical flattened *Baconia* form. Externally, they can be separated from the few other subcylindrical species (like *Baconia teredina*, above) by their emarginate anterior mesoventral margin (Fig. 52D), lack of mesometaventral stria, and basally united 5^th^ and sutural striae. The two species have very similar male genitalia, with an undivided, apically setose 8^th^ sternite that would appear to be a synapomorphy with the preceding group. However, their 9^th^ sternite is of a more generalized type, lacking the large quadrate head, and their aedeagus lacks eversible denticles as found in most *Baconia aeneomicans* group species, including the only cylindrical species, *Baconia teredina*.

##### Baconia cylindrica sp. n.

http://zoobank.org/E4F83D28-DE40-4ED7-8FFC-5B133A5AFB66

http://species-id.net/wiki/Baconia_cylindrica

**Map 16.**
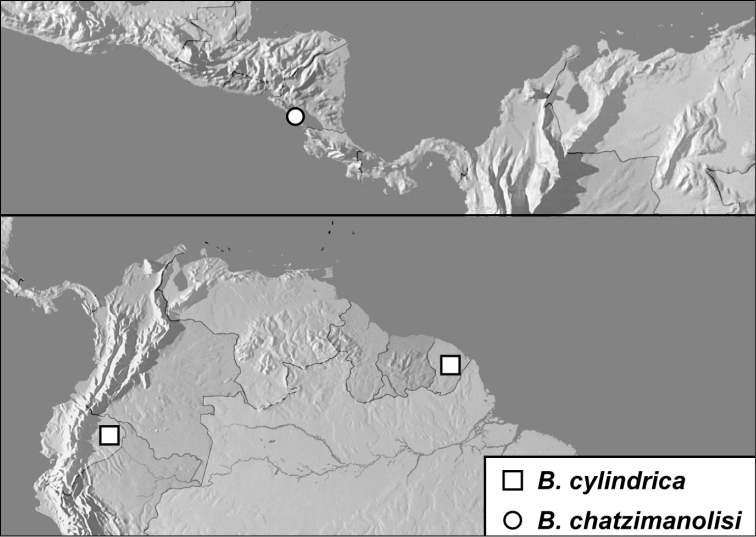
*Baconia cylindrica* group records.

Figs 52A–B 53A–C, H–I Map 16

###### Type locality.

**FRENCH GUIANA**: Rés. des Nouragues [4.0834°N, 52.6833°W].

###### Type material.

**Holotype male:** **“GUYANE FRANCAISE:** Rés. Natur. de Nouragues, Camp Inselberg. 4°05'N, 52°41'W, Piege vitre 8.x.2010. SEAG leg.” / “Caterino Tishechkin Exosternini Voucher EXO-0249” (MNHN); **Paratypes** (5):2: same data as type, 1: same locality as type, 25.i.2011 (CHND), 1: Rés. des Nouragues, Saut Parare, 4°02'N, 52°41'W, 15.vi.2010, FIT, SEAG, 1:20.iv.2010 (MNHN, CHND, MSCC, FMNH).

###### Other material.

1: **ECUADOR: Orellana,** Tiputini Biodiversity Station, 0°37'55"S, 76°08'39"W, 220–250m. 8.ii.1999, T.L.Erwin, fogging of mostly bare green leaves, some with covering of lichenous or bryophytic plants [specimen lost, male genitalia present] (USNM). 1: P.N. Yasuní, Est. Cient. Yasuní, 0°40.5'S, 76°24'W, 28.vi–5.vii.1999, FIT, C. Carlton & A. Tishechkin (LSAM).

###### Diagnostic description.

**Figure 53.**
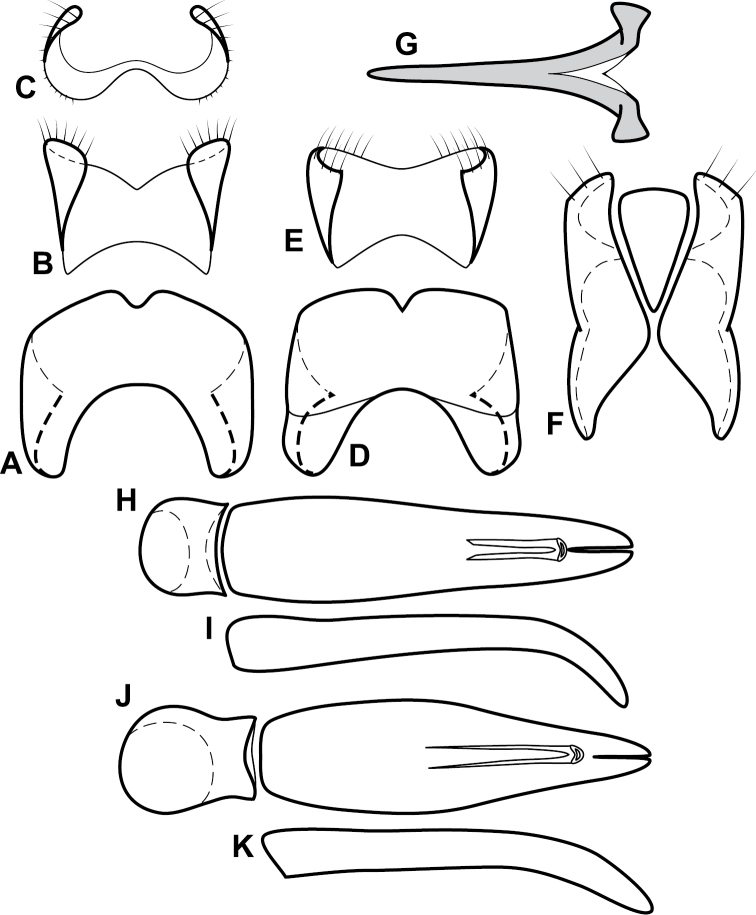
Male genitalia of *Baconia cylindrica* group. **A** T8 of *Baconia cylindrica* **B** S8 of *Baconia cylindrica* **C **S8 (apical view) of *Baconia cylindrica* **D** T8 of *Baconia chatzimanolisi* **E** S8 of *Baconia chatzimanolisi* **F** T9 & T10 of *Baconia chatzimanolisi* **G** S9 of *Baconia chatzimanolisi* **H** Aedeagus, dorsal view of *Baconia cylindrica* **I** Aedeagus, lateral view of *Baconia cylindrica* **J** Aedeagus, dorsal view of *Baconia chatzimanolisi* **K** Aedeagus, lateral view of *Baconia chatzimanolisi*.

Length: 1.1–1.3mm, width: 0.6–0.7mm; body narrowly elongate, cylindrical, glabrous; color rufescent, shining; head with frons convex, weakly elevated between antennal bases, very weakly depressed along midline, with small, coarse punctures evenly scattered throughout, frontal stria present only along upper edge of eye, absent across front, supraorbital stria present, may be fragmented; antennal scape short, club more or less circular; epistoma faintly emarginate; labrum about 2×wider than long, apical margin shallowly emarginate; mandibles short, each with small, acute basal tooth; pronotum with sides subparallel in basal two-thirds, rounded to apex, lateral marginal stria descending to ventral edge of margin behind antennal cavity, faint or merging with lateral submarginal stria, continued around anterior corner but detached from median part of anterior marginal stria, which diverges slightly from margin behind eye; pronotal disk with ground punctation fine, very sparse, with small secondary punctures densest across anterior half; elytra with single, fine epipleural stria, outer subhumeral stria absent, inner subhumeral stria barely impressed at extreme base, dorsal stria 1 present in basal half, striae 2–3 slightly longer, 4^th^ stria present basally and with separate median fragment, 5^th^ and sutural striae present in basal two-thirds, joined in narrow basal arch, elytral disk with small secondary punctures in apical one-fourth; prosternal keel narrow, flat, base produced, with carinal striae converging slightly to front, may be united basally and/or apically; prosternal lobe about two-thirds keel length, apical margin broadly rounded, slightly deflexed, marginal stria obsolete at sides; mesoventrite emarginate at middle, with complete marginal stria; mesometaventral stria absent from middle, inner lateral metaventral stria continuing from marginal mesoventral stria, extending posterad toward inner corner of metacoxa, outer lateral metaventral stria very short, curving behind mesocoxa; metaventral disk sparsely punctate at sides, impunctate at middle; abdominal ventrite 1 with complete inner lateral stria and abbreviated outer stria, central portion of disk impunctate, ventrites 2–5 with fine punctures at sides, sparser across middle; protibiae distinctly tridentate, margin only very finely serrulate between teeth; mesotibia with two marginal and one subapical spines; outer metatibial margin smooth; propygidium lacking basal stria, with fine, sparse ground punctation interspersed with small secondary punctures separated by slightly less than their diameters, propygidial gland openings inconspicuous; pygidium similarly punctate to propygidium, slightly sparser. Male genitalia (Figs 53 A–C, H–I): T8 slightly wider than long, deeply, arcuately emarginate at base, sides weakly converging apicad, ventrolateral apodemes with inner apices opposing, separated by about two-thirds T8 width, projecting beneath to about ventral midpoint, rapidly narrowed apically, apical margin shallowly, narrowly emarginate; S8 with halves fused, basal emargination broad, shallow, sides diverging to apex, apical guides well developed, widest apically, bearing a dense fringe of setae along most of outer margin; T9 with dorsal plates broad to base, proximal apodemes strongly reduced, sides weakly diverging to obliquely truncate apices bearing several conspicuous setae, ventrolateral apodemes strong, nearly meeting at midline about one-third from apex; S9 stem more or less evenly tapered to very narrow base, head bluntly subquadrate, apices divergent, apical emargination narrow; tegmen widest near base, sides undulate, narrowed just distad middle, curved ventrad in apical half, apex rounded; median lobe simple, about one-third tegmen length; basal piece very short, about one-fifth tegmen length.

###### Remarks.

While this species and the following are easily diagnosed together, they are very similar in their elongate, cylindrical form (Figs 52A, C), and rather difficult to separate from each other. *Baconia cylindrica* is smaller in body size and has the 4^th^ elytral stria merely interrupted, whereas in *Baconia chatzimanolisi*, it is largely absent. We exclude specimens from Ecuador from the type series due to slight differences in pronotal and propygidial punctation.

###### Etymology.

This species is named for its cylindrical body form.

##### Baconia chatzimanolisi sp. n.

http://zoobank.org/5DCA0360-4458-4F5E-82BE-5D465A6C277C

http://species-id.net/wiki/Baconia_chatzimanolisi

Figs 52C–D 53D–G, J–K Map 16

###### Type locality.

NICARAGUA: Granada: Volcan Mombacho [11.83°N, 85.98°W].

###### Type material.

**Holotype male**: “**NICARAGUA: Granada** Dept. Res. Nat. Volcan Mombacho, entrance rd, 910m 11°50.05'N, 85°58.83'W 1-50-VI-2002, R.Brooks, Z.Falin, S.Chatzimanolis ex. flight intercept trap, NIC1BFC02 189” / “SM0537395 KUNHM-ENT” (SEMC). **Paratypes** (1): same data as type (SEMC).

###### Diagnostic description.

Length: 1.4–1.5mm, width: 0.8–0.9mm; body narrowly elongate, cylindrical, glabrous; color rufescent, shining; head with frons slightly elevated between antennal bases, weakly convex, with distinct small punctures sparsely but evenly scattered throughout, frontal stria present only along upper half of eye, absent across front, supraorbital stria present but fragmented; antennal scape short, club broadly oval, subtruncate apically; epistoma faintly emarginate; labrum about 2×wider than long, apical margin shallowly emarginate on upper edge, slightly produced beneath; mandibles short, each with small, acute basal tooth; pronotum with sides subparallel in basal two-thirds, rounded to apex, lateral marginal stria descending to ventral edge of margin behind antennal cavity, merging with lateral submarginal stria, continued around anterior corner but detached from median part of anterior marginal stria, which diverges from margin behind eye, pronotal disk with ground punctation fine, very sparse, with small secondary punctures more or less evenly scattered throughout, somewhat sparser in prescutellar area; elytra with single, fine epipleural stria, outer subhumeral stria absent, inner subhumeral stria barely impressed at extreme base, dorsal stria 1 present in basal half, striae 2–3 progressively slightly longer, 4^th^ stria absent, 5^th^ and sutural striae present in basal two-thirds, joined in narrow basal arch; elytral disk with small secondary punctures in apical one-fourth; prosternal keel narrow, flat, base produced, with carinal striae joined along basal margin, subparallel or converging slightly to front; prosternal lobe about two-thirds keel length, apical margin broadly rounded, slightly deflexed, marginal stria obsolete at sides; mesoventrite emarginate at middle, with complete marginal stria; mesometaventral stria absent from middle, inner lateral metaventral stria continuing from marginal mesoventral stria, extending posterad toward inner corner of metacoxa, outer lateral metaventral stria very short, curving behind mesocoxa; metaventral disk sparsely punctate at sides, impunctate at middle; abdominal ventrite 1 with single complete lateral stria, central portion of disk impunctate, ventrites 2–5 with fine punctures at sides, sparser across middle; protibiae distinctly tridentate, margin only very finely serrulate between teeth; mesotibia with two marginal and one subapical spines; outer metatibial margin smooth; propygidium lacking basal stria, with fine, sparse ground punctation interspersed with small secondary punctures separated by slightly more than their diameters, propygidial gland openings inconspicuous; pygidium similarly punctate to propygidium, slightly sparser. Male genitalia (Figs 53D–G, J–K): T8 slightly wider than long, deeply, arcuately emarginate at base, sides weakly converging apicad, ventrolateral apodemes with inner apices opposing, separated by about two-thirds T8 width, projecting beneath to about ventral midpoint, strongly narrowed apically, apical margin shallowly, narrowly emarginate; S8 with halves fused, basal emargination broad, shallow, sides diverging to apex, apical guides well developed, widest apically, bearing a dense fringe of setae along outer margin; T9 with dorsal plates broad to base, proximal apodemes strongly reduced, sides weakly diverging to obliquely truncate apices bearing several conspicuous setae, ventrolateral apodemes strong, nearly meeting at midline about one-third from apex; S9 stem more or less evenly tapered to very narrow base, head bluntly subquadrate, apices divergent, apical emargination narrow; tegmen widest in basal two-thirds, sides sinuate, narrowed in apical third, apex narrowly rounded, tegmen weakly curved ventrad in apical half; median lobe simple, about one-half tegmen length; basal piece basally bulbous, about one-third tegmen length.

###### Remarks.

As discussed above, the two species in this group are very similar. *Baconia chatzimanolisi* can be recognized by its larger body size and strongly reduced 4^th^ elytral stria (Fig. 52C).

###### Etymology.

We name this species for staphylinid specialist Dr. Stylianos Chatzimanolis, a former SBMNH postdoctoral fellow, now at University of Tennessee Chattanooga, and co-collector of the types of this species.

#### *Baconia gibbifer* group

**Figure 54.**
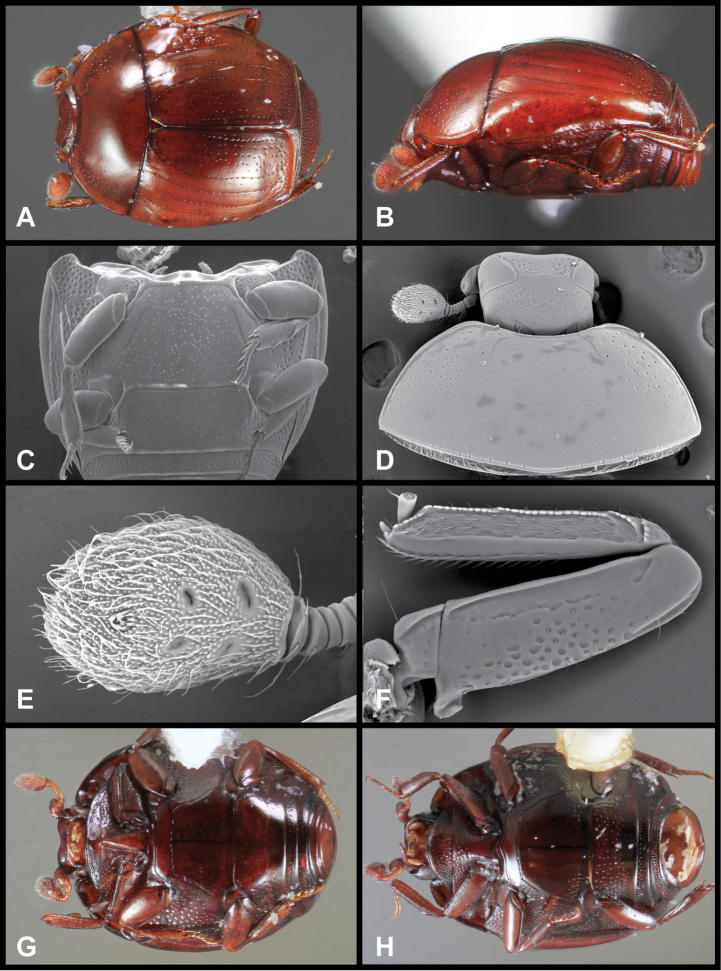
*Baconia gibbifer* group. **A** Dorsal habitus of *Baconia gibbifer* **B** Lateral habitus of *Baconia gibbifer* **C** Meso- and metaventrites of *Baconia gibbifer* **D** Pronotum of *Baconia gibbifer* **E** Antennal club of *Baconia gibbifer* **F **Protibia of *Baconia gibbifer* **G** Ventral habitus of *Baconia piluliformis* **H** Ventral habitus of *Baconia maquipucunae*.

As with the *Baconia cylindrica* group, above, members of the *Baconia gibbifer* grouphave genitalic characters (fused S8) which ally them clearly with the *Baconia aeneomicans* group, but they exhibit another highly distinctive body form, in which the species are strongly convex (Fig. 54B) and broadly rounded to subquadrate (Fig. 54A). All have very narrow protibiae which lack discrete marginal spines (Fig. 54F), except at the apical corner. Most also have the frons protuberant over the antennal bases (e.g. Fig. 56B), and the central portion of the anterior marginal pronotal stria detached with the ends curved posterad. Interestingly this last character is associated with posterior movement of the median pair of anterior pronotal glands, just as in *Operclipygus* (Caterino & Tishechkin 2013a), although this undoubtedly represents convergence. Male gentialia in the group show numerous similarities with members of the *Baconia aeneomicans* group, from within which they are probably derived. Most notably they share an undivided 8^th^ sternite, broad-headed spiculum gastrale, and presence of eversible subapical aedeagal denticles.

##### Baconia gibbifer sp. n.

http://zoobank.org/CCA7742B-87F7-41E9-92F3-9EC5261F0C4C

http://species-id.net/wiki/Baconia_gibbifer

**Map 17.**
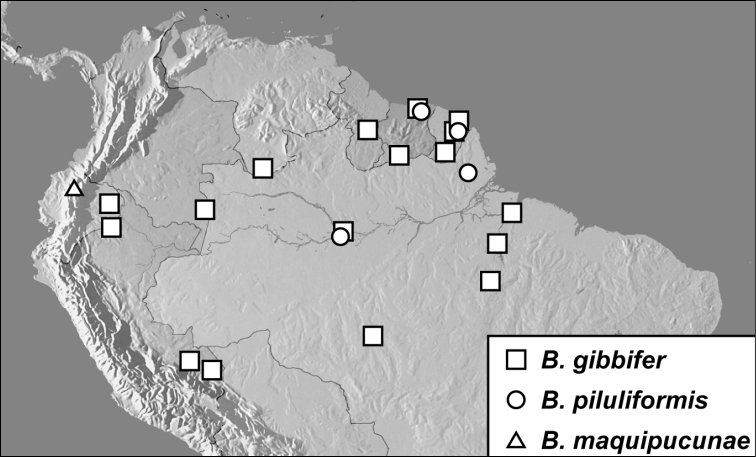
*Baconia gibbifer* group records.

Figs 1C 54A–F 55A–B, D, F, H–I Map 17

###### Type locality.

ECUADOR: Orellana, Yasuní Research Station [0.674°S, 76.398°W].

###### Type material.

**Holotype male**: “**ECUADOR:** Napo, mid. Rio Tiputini, Yasuní Res. Stn. 0°40.5'S, 76°24'W FIT#M1, 7-13 Jul 1999 AKT#088 C.Carlton & A.Tishechkin” / “LSAM0012899” (FMNH). **Paratypes** (10): **ECUADOR**:1: **Orellana**: Est. Biodiv. Tiputini, 0.6376°S, 76.1499°W, 2–9.vi.2011, FIT, M. Caterino & A. Tishechkin, DNA Extract MSC-2126, EXO-00628, 1: 30.vii.2008, Day FIT, A. Tishechkin, DNA Extract MSC-1898, EXO-02850, 2: 220 m, 5–25.ix.2000, D. Inward & K. Jackson; 1: P. N. Yasuní, Est. Cient. Yasuní, 0°40.5'S, 76°24'W, 16–17.vii.2008, FIT, A. Tishechkin, 1: 18–23.vi.1999, FIT, A. Tishechkin (LSAM), 2: 23–30.vi.1999, FIT, C. Carlton & A. Tishechkin (LSAM), 1: 28.vi–5.vii.1999, FIT, A. Tishechkin (LSAM), 1: 5–11.vii.1999, FIT, C. Carlton & A. Tishechkin (LSAM).

###### Other material.

(60) **BRAZIL**: 2: **Manaus**: INPA, 2°25'S, 59°50'W, iii.1994, Winklered leaf litter, terra firme forest, R. Didham; 1: **Mato Grosso**:Mpio. Cotriguaçu, Fazenda São Nicolau, Matinha, 9°50.3'S, 58°15.05'W, 3.iv.2009, FIT, F. Vaz-de-Mello (CEMT); 1: **Pará**: Belem, Utinga, IPEAN, 1°27'S, 48°26'W, viii.1985, FIT (CHND), 1: xi.1984, FIT (AKTC), 1: xii.1984, FIT (MSCC); 2: Carajas (Serra Norte), 6°04'S, 50°12'W, v.1985, FIT; 2: Tucuruí, 3°45'S, 49°40'W, 10-29.vii.1985, FIT, 1: 19.vi–7.vii.1986, FIT, 1: v.1986, FIT (CHND). **COLOMBIA**: 3: **Vaupés**: P. N. Mosiro-Itajura, Centro Ambiental, 1°04'S, 69°31'W, 60 m, 20-30.i.2003, FIT, D. Arias & M. Sharkey (IAVH, FMNH). **FRENCH GUIANA**: 1:Rés. des Nouragues, Camp Inselberg, 4°05'N, 52°41'W, 20.vii.2009, FIT, SEAG (MNHN), 6:25.i.2011, FIT, SEAG (CHND), 1:30.ix.2010, FIT, SEAG (AKTC); 2:16.ix.2010, FIT, S. Brule (UFPR); 1:Rés. des Nouragues, Régina, 4°2.27'N, 52°40.35'W, 10.x.2009, FIT, SEAG (CHND), 2:8.ix.2009, FIT, SEAG (CHND, AKTC); 1:Res. Tresor, rte. de Kaw, Pk18, 4°36.63'N, 52°16.74'W, 225 m, 13.x.2009, FIT, SEAG (CHND), 1: 26.ix.2009, FIT, SEAG (CHND); 1:18.4 km SSE Roura, 4°36'38"N, 52°13'25"W, 240 m, 29.v–10.vi.1997, FIT, J. Ashe & R. Brooks (SEMC); 1:8.4 km SSE Roura, 4°40'41"N, 52°13'25"W, 200 m, 29.v–10.vi.1997, FIT, J. Ashe & R. Brooks (SEMC); 3:Belvèdére de Saül, 3°1'22"N, 53°12'34"W, 14.ii.2011, FIT, SEAG (CHND), 1:17.i.2011, FIT, SEAG (CHND), 1:17.ix.2010, FIT, S. Brule (UFPR), 1:20.xii.2010, FIT, SEAG (CHND), 1:30.xi.2010, FIT, SEAG (CHND), 1:31.i.2011, FIT, SEAG (CHND), 4:7.ii.2011, FIT, SEAG (CHND, MSCC, AKTC), 1:Cayenne, 33.5 km S and 8.4 km NW of Hwy N2 on Hwy D5, 4°48'18"N, 52°28'41"W, 30 m, 29.v-9.vi.1997, FIT, J. Ashe & R. Brooks (SEMC); 1:Mont tabulaire Itoupé, 3°1.82'N, 53°6.40'W, 400 m, 17.iii.2010, FIT, SEAG (CHND); 1:Mont tabulaire Itoupé, 3°1.38'N, 53°5.73'W, 570 m, 31.iii.2010, FIT, SEAG (CHND); 1:Montagne des Chevaux, 4°43'N, 52°24'W, 23.ii.2009, FIT, SEAG (CHND), 1:4.vii.2009, FIT, SEAG (CHND); 1:20.vi.2009, FIT, SEAG (CHND). **GUYANA**:1: **Region 8**:Iwokrama Field Stn., 4°40'19"N, 58°41'4"W, 60 m, 30.v–2.vi.2001, FIT, R. Brooks & Z. Falin (SEMC). **PERU**:1: **Loreto**: Teniente Lopez, 2°35.66'S, 76°06.92'W, 210-240 m, 24.vii.1993, FIT, R. Leschen (SEMC); 2: **Madre de Dios**: CICRA, Rio Los Amigos, 25–150 m, 18–21.xi.2006, FIT, A. Asenjo (MUSM), 1: 24–26.xi.2006, FIT, A. Asenjo (MUSM); 1: P. N. Manu, Est. Biol. Cocha Cashu, 11°53'45"S, 71°24'24"W, 350 m, 17–19.x.2000, FIT, R. Brooks (SEMC); 1: Pantiacolla Lodge, Alto Madre de Dios R., 12°39.3'S, 71°13.9'W, 420 m, 14–19.xi.2007, FIT, D. Brzoska (SEMC). **SURINAME**: 1: **Pará**: nr. Overbridge River Resort, 5°31.8'N, 55°3.5'W, 15–18.ii.2010, FIT, C. Gillet, P. Skelley, W. Warner (FSCA); 2: **Sipaliwini**: CI-RAP Surv. Camp 1: on Kutari River, 2°10.521'N, 56°47.244'W, 228 m, 19–24.viii.2010, FIT, T. Larsen & A. Short (SEMC, MSCC); **VENEZUELA**: 1: **Amazonas**: Cerro de la Neblina basecamp, 0°50'N, 66°10'W, 140 m, 10–20.ii.1985, FIT, rainforest, P. Spangler, R.A. Faitoute & W. Steiner (USNM).

**Figure 55.**
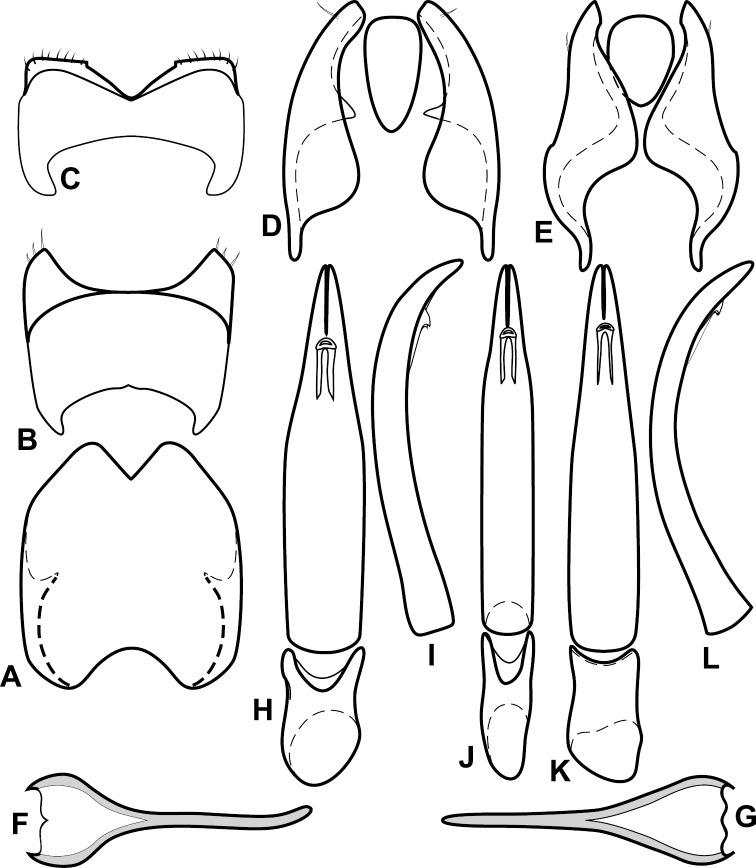
Male genitalia of *Baconia gibbifer* group. **A** T8 of *Baconia gibbifer* **B** S8 of *Baconia gibbifer* **C** S8 of *Baconia maquipucunae* **D** T9 & T10 of *Baconia gibbifer* **E** T9 & T10 of *Baconia piluliformis* **F** S9 of *Baconia gibbifer* **G** S9 of *Baconia piluliformis* **H** Aedeagus, dorsal view of *Baconia gibbifer* **I** Aedeagus, lateral view of *Baconia gibbifer* **J** Aedeagus, dorsal view of *Baconia piluliformis* **K** Aedeagus, dorsal view of *Baconia maquipucunae* **L** Aedeagus, lateral view of *Baconia maquipucunae*.

###### Diagnostic description.

Length: 1.4–1.5mm, width: 1.2–1.5mm; body elongate oval, strongly convex, glabrous; color rufescent, shining; head with frons elevated over and between antennal bases, narrowly depressed at middle, interocular margins convergent dorsad, frontal disk mostly impunctate, with few punctures at sides and along dorsal margin, frontal stria represented by short fragment along upper margin of eyes, supraorbital stria short, irregular; antennal scape very short, club large, elongate oval, with small, round median sensorium on upper surface distad the typical four sensoria (Fig. 54E); epistoma narrowly, transversely depressed beneath antennal bosses, apical margin truncate; labrum about 3×wider than long, apical margin deeply emarginate; mandibles short, each with minute, acute basal tooth; pronotum transverse, short, with sides strongly arcuate to apex, lateral marginal stria descending to ventral edge about one-third behind anterior corner, extending around corner to meet anterior marginal stria, which is recurved posterad for almost one-third pronotal length behind each eye (median pronotal gland opening displaced posterad to its apex), lateral submarginal stria nearly complete, merging with marginal stria or ending freely, pronotal disk with ground punctation fine, very sparse, with small secondary punctures mainly in anterolateral corners; elytra with upper epipleural stria complete, the lower somewhat fragmented, outer and inner subhumeral striae absent, dorsal stria 1 strongly shortened, present only in basal one-half, striae 2–3 nearly complete, 4^th^ stria present only as short basal arch, 5^th^ stria absent, sutural stria abbreviated slightly from base and apex, elytral disk with small secondary punctures in most of apical one-fourth, extending further anteriorly mediad 3^rd^ stria; prosternal keel narrowly convex, narrowing anteriorly, emarginate at base, with more or less complete carinal striae converging to front; prosternal lobe very short, slightly deflexed, apical margin broadly arcuate, marginal stria obsolete at sides; mesoventrite weakly produced at middle, with marginal stria complete; mesometaventral stria absent from middle; inner lateral metaventral stria present as isolated oblique stria from mesometaventral suture to near inner corner of metacoxa, outer lateral metaventral stria very short, present only behind mesocoxa; metaventral disk moderately coarsely punctate at sides, impunctate at middle; abdominal ventrite 1 with lateral striae abbreviated posteriorly, usually continuous with complete anterior marginal stria, disk impunctate at middle, ventrites 2–5 with fine punctures across width; protibia narrow, lacking median and basal marginal denticles, margin straight, serrulate; mesofemur with posterior marginal stria curving anterad along apical margin, with transverse apical setal series; mesotibia with one fine marginal spine and short, oblique basal submarginal ridge; outer metatibial margin smooth; propygidium lacking basal stria, with fine ground punctation interspersed with dense, ocellate secondary punctures, propygidial gland openings evident, situated about one-fourth behind anterior margin and nearly one-third from lateral margin; pygidium with fine ground punctation, with small secondary punctures sparsely interspersed, principally in basal half. Male genitalia (Figs 55A–B, D, F, H–I): T8 slightly longer than wide, rather narrowly, arcuately emarginate at base, sides rounded, not markedly narrowing apically, ventrolateral apodemes with inner apices widely separated, projecting beneath to about ventral midpoint, obsolete apically, apical margin subacutely emarginate; S8 more or less flat, sides slightly upturned, diverging to apex, halves fused along midline, basal emargination broad, shallow, apical guides absent, apical lobes with few very fine, inconspicuous setae; T9 with dorsal plates rather broad, proximal apodemes reduced, short, sides curving, converging to obliquely truncate apices bearing single subapical seta on each side, ventrolateral apodemes nearly meeting at midline about one-third from apex; S9 stem narrow, weakly dorsoventrally keeled, head rounded, apical emargination shallow and sinuate; tegmen sides subparallel in basal half, strongly narrowed to apex, thick, strongly curved ventrad in apical half, with eversible subapical denticles ventrally; median lobe simple, about one-fifth tegmen length; basal piece one-third tegmen length.

###### Remarks.

This, the most common and widespread species in this group, may be separated by the others by its strongly recurved anterior marginal pronotal stria (Fig. 54D), short 4^th^, and absent 5^th^ elytral striae (Fig. 54A), the basal arch of the 4^th^ stria narrowly separated from the sutural stria, absence of mesometaventral stria (Fig. 54C), and usually complete stria across the anterior margin of abdominal ventrite 1 (Fig. 54C). The species shows considerable variability in minor characters of striation and punctation, and we limit the type series to those specimens from Amazonian Ecuador.

###### Etymology.

This species’ name refers to its broadly rounded, humpbacked appearance.

##### Baconia piluliformis sp. n.

http://zoobank.org/07B19606-2369-4788-9790-D15270C4297D

http://species-id.net/wiki/Baconia_piluliformis

Figs 54G 55E, G, J Map 17

###### Type locality.

BRAZIL: Manaus: INPA [2.41°N, 59.83°W].

###### Type material.

**Holotype male**: “BRAZIL:Manaus,AM. INPA/Smithsonian Res. 2°25'S, 59°50'W R.Didham.iv.1994” / “Leaf litter. Winkler method. Terra firme fst.” / “536 | 3” / “0463” / “Caterino Tishechkin Exosternini Voucher EXO-00495” (BMNH). **Paratypes** (6): **BRAZIL**: 1: **Amapá**: Serra do Navio, 0°59'N, 52°00'W, 1–14.v.1991, FIT (CHND). **FRENCH GUIANA**: 2:Rés. des Nouragues, Camp Inselberg, 4°05'N, 52°41'W, 25.i.2011, FIT, SEAG (MNHN, AKTC); 2:Belvèdére de Saül, 3°1'22"N, 53°12'34"W, 7.ii.2011, FIT, SEAG (CHND, MSCC). 1: **SURINAME**: **Commewijne**: Akintosoela, 32 km SE SURINAME River Bridge, road to Redi Doti, 5°16'17"N, 54°55'15"W, 40 m, 29.vi-3.vii.1999, FIT, Z. Falin, B. DeDijn, A. Gangadin (SEMC).

###### Diagnostic description.

Length: 1.4–1.5mm, width: 1.2–1.3mm; body elongate oval, strongly convex, glabrous; color rufescent, shining; head with frons elevated over and between antennal bases, narrowly depressed at middle, interocular margins convergent dorsad, frontal disk mostly impunctate, with few punctures at sides and along dorsal margin, frontal stria represented by short fragment along upper margin of eyes, supraorbital stria short, irregular; antennal scape very short, club large, elongate oval; epistoma narrowly transversely depressed beneath antennal bosses, apical margin truncate; labrum about 3×wider than long, apical margin deeply emarginate; mandibles short, each with minute, acute basal tooth; pronotum transverse, short, with sides strongly arcuate to apex, lateral marginal stria descending to ventral edge about one-third behind anterior corner, extending around corner to meet anterior marginal stria, which is recurved posterad about one-third pronotal length behind each eye, the median pronotal gland opening displaced posterad to near its apex, lateral submarginal stria nearly complete, merging with marginal stria or ending freely, pronotal disk with ground punctation fine, very sparse, with small secondary punctures mainly in anterolateral corners; elytra with upper epipleural stria complete, the lower somewhat fragmented, outer and inner subhumeral striae absent, dorsal stria 1 strongly shortened, present only in basal half, stria 2 nearly complete, 3^rd^ stria present in basal half or slightly more, 4^th^ stria present only as short basal arch, connected to base of sutural stria, 5^th^ stria absent, sutural stria abbreviated slightly from apex, elytral disk with small secondary punctures in most of apical one-fourth, extending further anteriorly mediad 3^rd^ stria; prosternal keel narrowly convex, strongly narrowed to subcarinate anteriorly, truncate at base, with more or less complete carinal striae weakly diverging to front; prosternal lobe very short, slightly deflexed, apical margin broadly arcuate, marginal stria more or less complete; mesoventrite truncate to very weakly produced at middle, with marginal stria complete; mesometaventral stria present, crenulate across middle, continued by inner lateral metaventral stria, extending obliquely posterad to near inner corner of metacoxa, outer lateral metaventral stria very short, present only behind mesocoxa; metaventral disk moderately coarsely punctate at sides, impunctate at middle; abdominal ventrite 1 with lateral stria strongly abbreviated posteriorly, ventrite lacking transverse anterior marginal stria, disk impunctate at middle, ventrites 2-5 with fine punctures across width; protibia narrow, lacking median and basal marginal denticles, margin straight, serrulate; mesofemur with posterior marginal stria curving anterad along apical margin; mesotibia with oblique, basal submarginal ridge; outer metatibial margin smooth; propygidium lacking basal stria, with fine ground punctation interspersed with ocellate secondary punctures mainly in basal half, propygidial gland openings evident, situated about one-fourth behind anterior margin and nearly one-third from lateral margin; pygidium with fine ground punctation, with few or no secondary punctures interspersed. Male genitalia (Figs 55E, G, J): T8 and S8 as in *Baconia gibbifer* (Figs 55A–B); T9 with dorsal plates rather broad near base, proximal apodemes reduced, short, sides uneven, narrowed strongly to apex, bearing single, inconspicuous, subapical seta on each side, ventrolateral apodemes nearly meeting at midline about one-third from apex, bluntly dentate; S9 stem narrow, strongly dorsoventrally keeled, head gradually widened to apex, apical emargination shallow and sinuate; tegmen narrower than that of *Baconia gibbifer*, sides subparallel in basal half, strongly narrowed to apex, thick, strongly curved ventrad in apical half, with eversible subapical denticles ventrally; median lobe simple, about one-fifth tegmen length; basal piece one-third tegmen length.

###### Remarks.

*Baconia piluliformis* is very similar to *Baconia gibbifer*, above, but has its mesometaventral stria present at middle (Fig. 54G), the prosternal keel strongly narrowed to a nearly knife-like carina anteriorly, the 4^th^ dorsal and sutural striae joined basally, the pygidium largely devoid of secondary punctures, and the anterior stria of 1^st^ abdominal ventrite absent.

###### Etymology.

This species’ name refers to its pill-shaped form.

##### Baconia maquipucunae sp. n.

http://zoobank.org/026F8151-FE51-47F0-9E4D-9D67E3D1274D

http://species-id.net/wiki/Baconia_maquipucunae

Figs 54H 55C, K–L Map 17

###### Type locality.

ECUADOR: Pichincha: Maquipucuna Forest Reserve [0.05°N, 78.68°W].

###### Type material.

**Holotype male**: “ECUADOR:Pichincha Maquipucuna For. Res. 50 km NW Quito, 1660 m 21 Dec. 1991, C.Carlton R.Leschen #22, ex:FIT” / “SEMC0903647 KUNHM-ENT” (SEMC).

###### Diagnostic description.

Length: 1.7mm, width: 1.6mm; body slightly elongate, rounded, convex, especially elytra, glabrous; color rufescent, shining; head with frons slightly elevated over antennal bases, weakly depressed at middle, ground punctation fine, inconspicuous, with coarser punctures at middle and along dorsal margin, frontal and supraorbital striae absent; antennal scape short, club slightly oblong; epistoma truncate; labrum about 4×wider than long, apical margin emarginate; mandibles short, right mandible with small, acute basal tooth; pronotum with sides convergent in basal two-thirds, rounded to apex, lateral marginal stria descending to ventral edge of pronotal margin one-third from apex, extending around anterior corner, detached from median part of anterior marginal stria, which diverges from margin behind eye, lateral submarginal stria present in basal three-fourths, very close to marginal stria, pronotal disk narrowly but strongly depressed in anterolateral corners, ground punctation inconspicuous, middle of disk impunctate, with small secondary punctures only in anterolateral corners; elytral humeri prominent, elytra more strongly convex than pronotum, with two epipleural striae, outer and inner subhumeral striae absent, dorsal striae 1–5 restricted to basal half of elytra, 4^th^ stria strongly arched toward scutellum at base, 5^th^ stria very short, sutural stria impressed along about middle one-fourth of suture, elytral disk with small secondary punctures sparsely scattered in apical one-third; venter markedly convex, prosternal keel ascending anterad, distinctly emarginate at base, with carinal striae weakly converging anterad, obsolete in anterior one-third; prosternal lobe about two-thirds keel length, apical margin rounded, deflexed, marginal stria obsolete at sides, both prosternal lobe and keel densely punctate at sides; mesoventrite produced at middle, with marginal stria narrowly interrupted; mesometaventral stria transverse, weakly crenulate, continuous laterally with inner lateral metaventral stria, which extends obliquely posterad toward inner third of metacoxa, outer lateral metaventral stria absent; metaventral disk densely punctate at sides, with numerous small punctures in front of metacoxa; abdominal ventrite 1 with single abbreviated lateral stria, with small median discal punctures sparsely scattered in anterior two-thirds, ventrites 2–5 with fine punctures, those of ventrite 4 moderately dense across middle; protibia narrow, lacking denticles along margin, with apical denticle only, margin very finely serrulate; meso- and metatibial margins smooth, lacking marginal spines; propygidium lacking basal stria, with fine ground punctation uniformly interspersed with small, ocellate secondary punctures, propygidial gland openings inconspicuous; pygidium with fine ground punctation only in apical two-thirds, with small secondary punctures principally in basal one-third. Male genitalia (Figs 55C, K–L): T8 as in *Baconia gibbifer* (Fig. 55A); S8 short, basal emargination broad and deep, sides rounded, apices subtruncate, lateral thirds well-sclerotized and bearing short fringe of setae, inner corners weakly produced, middle third subacutely emarginate; T9 as in *Baconia piluliformis* (Fig. 55E), but bearing 2–3 inconspicuous subapical setae; S9 stem narrow, strongly dorsoventrally keeled, head gradually widened to apex, apical emargination shallow and sinuate; tegmen rather narrow, sides subparallel in basal half, strongly narrowed to apex, thick, more or less evenly curving ventrad over entire length, with eversible subapical denticles ventrally; median lobe simple, about one-third tegmen length; basal piece one-third tegmen length.

###### Remarks.

This species may be separated from others in the group by the presence of a short fragment of the 5^th^ elytral stria, the rather strongly narrowed pronotum which is strongly depressed in the anterior corners, the weakly produced frontal corners, the broad prosternal keel (Fig. 54H), and the strongly convex metaventrite.

###### Etymology.

This species named for its type locality, the Maquipucuna Forest Reserve, on the high western slopes of the Ecuadorian Andes.

##### Baconia tenuipes sp. n.

http://zoobank.org/117E71FA-AE4B-4DD6-976D-DA25BA0155E6

http://species-id.net/wiki/Baconia_tenuipes

**Figure 56.**
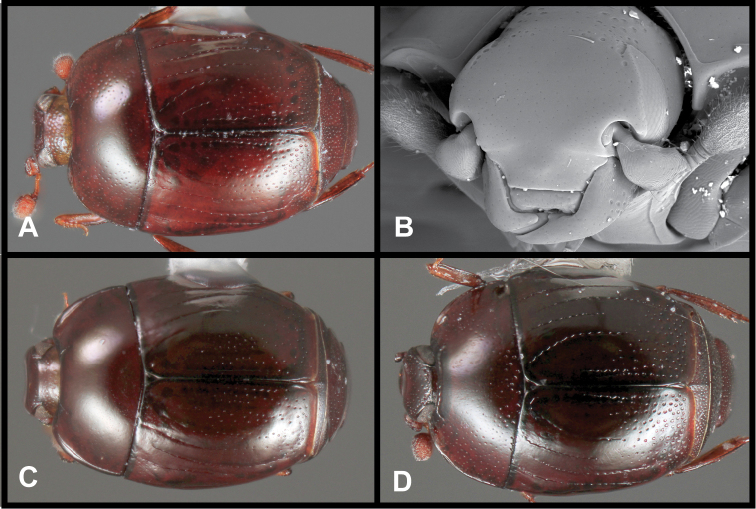
*Baconia gibbifer* group. **A** Dorsal habitus of *Baconia tenuipes* **B** Frons of *Baconia tuberculifer* **C** Dorsal habitus of *Baconia tuberculifer* **D** Dorsal habitus of *Baconia globosa*.

**Map 18.**
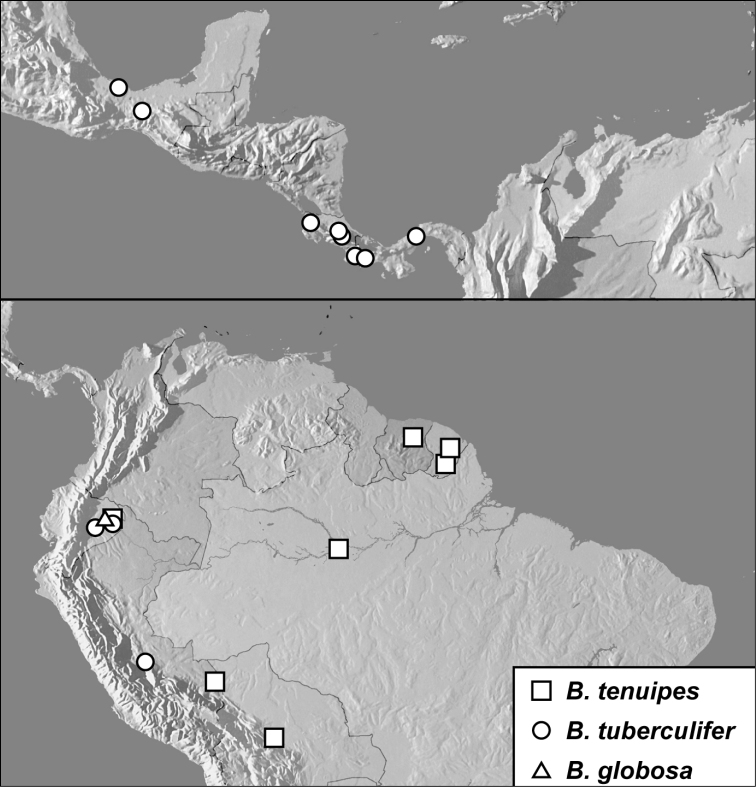
*Baconia gibbifer* group records.

Figs 56A 57A–B, J–K Map 18

###### Type locality.

ECUADOR: Orellana: Tiputini Biodiversity Station [0.635°S, 76.150°W].

###### Type material.

**Holotype male**: “**ECUADOR: Depto. Orellana,** Tiputini Biodiversity Station 0°37'55"S, 76°08'39"W, 220–250m. 1 July 1998 T.L.Erwin et al. collectors” / “insecticidal fogging of mostly bare green leaves, some with covering of lichenous or bryophytic plants **Lot 1841 Trans. 5 Sta. 2**” / “Caterino/Tishechkin Exosternini Voucher EXO-02868”” (USNM). **Paratypes** (8): **ECUADOR**: **Orellana**: 1: P. N. Yasuní, Est. Cient. Yasuní, 0°40.5'S, 76°24'W, 17-23.vi.1999, FIT, C. Carlton & A. Tishechkin (LSAM); 1: Res. Ethnica Waorani, 1 km S Onkone Gare Camp, Trans. Ent., 0°39'10"S, 76°26'W, 220 m, 2.vii.1995, fogging, mostly bare green leaves, some with covering of lichenous or brophytic plants in terra firme forest, T. Erwin (USNM). 1: **BOLIVIA**: **Cochabamba**: 117 km E Cochabamba, at Lagunitas, 17°06'22"S, 65°40'57"W, 1000 m, 6–8.ii.1999, FIT, mountain evergreen forest, F. Genier (CMNC). **BRAZIL**: **Manaus**: 1: INPA, 2°25'S, 59°50'W, ii.1994, Winklered leaf litter, terra firme forest, R. Didham (BMNH). **FRENCH GUIANA**: 1:Belvèdére de Saül, 3°1'22"N, 53°12'34"W, 30.xi.2010, FIT, SEAG. 1:Montagne des Chevaux, 4°43'N, 52°24'W, 4.vii.2009, FIT, SEAG (CHND). 1: **PERU**: **Madre de Dios**: Res. Cuzco Amazonica, 15 km NE Pto. Maldonado, 12°33'S, 69°03'W, 200 m, 17.vi.1989, FIT, J. Ashe, R. Leschen (SEMC). 1: **SURINAME**: **Brokopondo**: Brownsberg Nat. Pre., 4°56'55"N, 55°10'53"W, 480 m, 23.vi.1999, FIT, Z. Falin (CMNC).

###### Diagnostic description.

**Figure 57.**
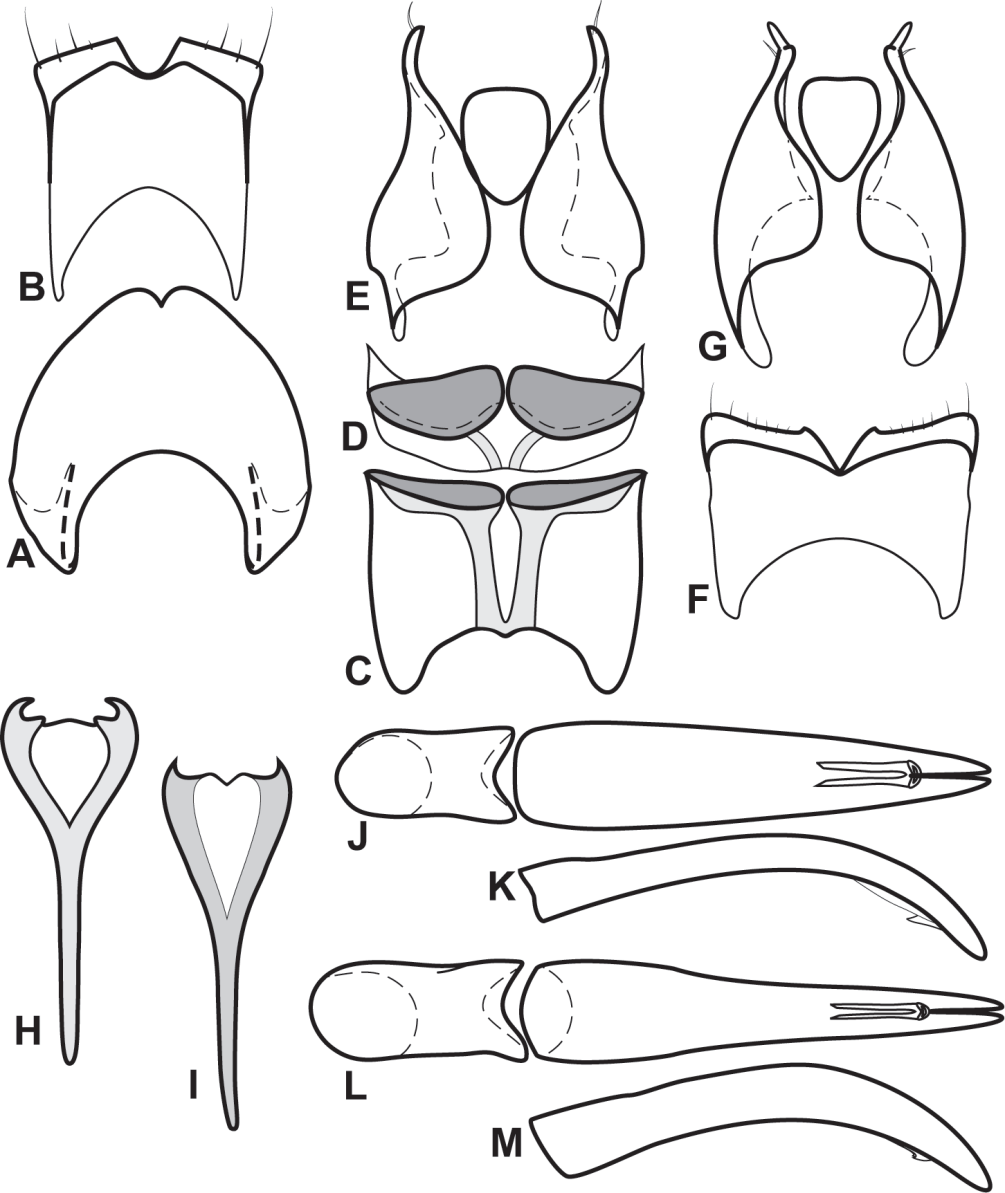
Male genitalia of *Baconia gibbifer* group. **A** T8 of *Baconia tenuipes* **B** S8 of *Baconia tenuipes* **C** S8 of *Baconia tuberculifer* **D** S8 (apical view) of *Baconia tuberculifer* **E** T9 & T10 of *Baconia tuberculifer* **F** S8 of *Baconia globosa* **G **T9 & T10 of *Baconia globosa* **H** S9 of *Baconia tenuipes* **I** S9 of *Baconia tuberculifer* **J** Aedeagus, dorsal view of *Baconia tenuipes* **K **Aedeagus, lateral view of *Baconia tenuipes* **L** Aedeagus, dorsal view of *Baconia tuberculifer* **M** Aedeagus, lateral view of *Baconia tuberculifer*.

Length: 1.3–1.7mm, width: 1.1–1.4mm; body subquadrate, weakly convex, glabrous; color rufescent, shining; head with frons weakly elevated over and between antennal bases, shallowly depressed at middle, interocular margins convergent dorsad, frontal disk mostly impunctate, with few punctures at sides and along dorsal margin, frontal stria absent or represented by short fragment along upper margin of eyes, supraorbital stria present; antennal scape very short, club large, elongate oval; epistoma weakly depressed beneath antennal bosses, apical margin truncate; labrum about 3×wider than long, apical margin emarginate; mandibles short, each with minute, acute basal tooth; pronotum transverse, short, with sides subparallel in basal half, arcuate to apex, lateral marginal stria descending to ventral edge about one-third behind anterior corner, extending around corner nearly meeting anterior marginal stria, which is recurved posterad for about one-sixth pronotal length behind each eye, the median pronotal gland opening displaced posterad to near its apex, lateral submarginal stria nearly complete, ending freely, pronotal disk with ground punctation fine, very sparse, with small secondary punctures in lateral thirds of disk; elytra with two complete epipleural striae, the lower somewhat fragmented, outer subhumeral stria absent, inner subhumeral stria present as short basal fragment, dorsal stria 1 obsolete in apical third, stria 2 nearly complete, 3^rd^ stria present in basal half or slightly more, 4^th^ stria present in basal half, narrowly arched to connect to base of sutural, 5^th^ stria present as short basal fragment, sutural stria obsolete in apical one-third, elytral disk with small secondary punctures in most of apical half; prosternal keel narrowly convex, more strongly narrowed anteriorly, emarginate at base, with carinal striae fragmented to obsolete; prosternal lobe short, deflexed, apical margin broadly arcuate, marginal stria more or less complete; mesoventrite narrowly produced at middle, with marginal stria complete; mesometaventral stria present, crenulate across middle, continued by inner lateral metaventral stria, extending obliquely posterad to near inner corner of metacoxa, outer lateral metaventral stria very short, present only behind mesocoxa; metaventral disk moderately coarsely punctate at sides, impunctate at middle; abdominal ventrite 1 with single, complete lateral stria, ventrite lacking transverse anterior marginal stria, disk impunctate at middle, ventrites 2–5 with fine, moderately dense punctures across width; protibia narrow, lacking median and basal marginal denticles, margin straight, serrulate; mesofemur with posterior marginal stria curving anterad along apical margin; mesotibia with subapical marginal spine and oblique, basal submarginal ridge; outer metatibial margin smooth; propygidium lacking basal stria, with fine ground punctation interspersed with small, ocellate secondary punctures, propygidial gland openings inconspicuous; pygidium with fine ground punctation, with coarser secondary punctures more or less uniformly interspersed. Male genitalia (Figs 57A–B, J–K): T8 as in *Baconia gibbifer* (Fig. 55A); S8 slightly longer than wide, weakly widened to apex, basal emargination broad and deep, apices subtruncate, very slightly oblique, laterally well-sclerotized and bearing short fringe of inconspicuous setae, inner corners weakly produced, with narrow, subacute median emargination; T9 as in *Baconia piluliformis* (Fig. 55E); S9 stem narrow, strongly dorsoventrally keeled, head gradually widened to apex, apical emargination shallow and sinuate; tegmen widest just distad base, sides evenly narrowed to apex, thick, curving ventrad in apical half, with eversible subapical denticles ventrally; median lobe simple, about one-third tegmen length; basal piece one-third tegmen length.

###### Remarks.

The body shape in *Baconia tenuipes* is more distinctly subdepressed and broad, nearly subquadrate, than in other species in this group (Fig. 56A). The antennal bosses are weak, pronotal punctures coarser and more widespread, recurved anterior marginal stria short, basal fragment of the 5^th^ elytral stria present, and the 4^th^ stria more narrowly arched anteriorly, connected to the sutural stria basally.

###### Etymology.

This species’ name refers to its very narrow protibiae.

##### Baconia tuberculifer sp. n.

http://zoobank.org/4AD78173-B549-4E68-A7B1-FB5D07DE4C0A

http://species-id.net/wiki/Baconia_tuberculifer

Figs 56B–C 57C–E, L–M Map 18

###### Type locality.

PANAMA: Colón: San Lorenzo Forest [9.28°N, 79.97°W].

###### Type material.

**Holotype male**: “**PANAMA: Colón Prov.**, San Lorenzo Forest, STRI crane site. 9°17'N, 79°58'W, FIT-Z-3. 14–15 May 2004 A.Tishechkin. AT-440” / “Caterino/Tishechkin Exosternini Voucher EXO-00515” (FMNH). **Paratypes** (17): **COSTA RICA**:2: **Cartago**: P. N. Tapanti, Quebrada Segunda, 1250 m, vii.1992, G. Mora (INBI); 1: **Heredia**: 16 km SSE La Virgen, 10°16'N, 84°05'W, 1050–1150 m, 9–14.iii.2001, FIT, primary forest, E. Riley (TAMU); 2: **Puntarenas**: Est. Biol. Las Cruces, Coto Brus, 8°17'N, 82°57'W, 1100 m, 18-20.vi.2005, FIT, M. Ferro (AKTC, LSAM), 1: 1100 m, 22–23.iii.2002, FIT, A. Tishechkin (AKTC), 2: 1000 m, 22-23.iii.2002, FIT, A. Cline & A. Tishechkin (LSAM), 1: 1330 m, 28–31.v.2004, FIT, J. Ashe, Z. Falin, I. Hinojosa (SEMC); 1: P. N. Amistad, Est. Las Mellizas, Fca. Cafrosa, 1300 m, iii.1990, M. Ramirez & G. Mora (INBI); 1: San Vito, Las Cruces, 17.viii–12.ix.1982, FIT, B. Gill (BDGC), 1: 1200 m, vii.1982, B. Gill (CHSM). **PANAMA**:1: **Chiriquí**: Res. La Fortuna Estacion Biologica, 08°43'18"N, 82°14'17"W, 3900 ft, 4–10.viii.1999, FIT, Schaffner & J. Woolley (TAMU); 1: La Fortuna Dam, 1200 m, 14.vi–16.vii.1982, B. Gill (BDGC); 1: La Fortuna, “Hydro Trail”, 08°42'N, 82°14'W, 1150 m, 23.v–9.vi.1995, FIT, J. Ashe & R. Brooks (SEMC); 1: **Colón**: P. N. San Lorenzo, STRI Crane Site, 9°17'N, 79°58'W, 20–21.v.2004, FIT, A. Tishechkin (GBFM); 1: **Panamá**:Chepo-Cartí Rd., 400 m, vi.1982, FIT, B. Gill (BDGC).

###### Other material.

(14) **COSTA RICA**:1: **Guanacaste**: P. N. Guanacaste, 9 km S Santa Cecilia, Est. Pitilla, 700 m, 27.vii–14.viii.1992, P. Rios (INBI), 2: x.1994, C. Moraga (INBI). **MEXICO**: 1: **Chiapas**: Parque Laguna Belgica, 19.3 km N Ocozocoautla, 970 m, 12.vi.1991, FIT, J. Ashe (SEMC), 1: 8.vi.1991, FIT, J. Ashe (CHSM); 1: 16 km NW Ocozocoautla, 970 m, 31.v.1990, H. & A. Howden (CMNC); 1: **Veracruz**: Est. Biol. Los Tuxtlas, 33 km N Catemaco, 22–29.vi.1984, D. Lindeman (SEMC). **ECUADOR**:1: **Napo**: Est. Biol. Jatun Sacha, 21 km E Puerto Napo, 400 m, 18.vii.1994, FIT, lowland rainforest, Levy & F. Genier (CMNC); 1: **Orellana**: Yasuní Res. Stn., 0°40.5'S, 76°24'W, 23–30.vi.1999, FIT, C. Carlton & A. Tishechkin (LSAM), 2: 5–11.vii.1999, FIT, C. Carlton & A. Tishechkin (LSAM). **PERU**:1: **Junín**: 11 km NE Puerto Ocopa, Los Olivos, 11°3.00'S, 74°15.52'W, 1200 m, 26–27.iii.2009, FIT, A. Tishechkin, DNA Extract MSC-2152, EXO-00680, 1: 30–31.iii.2009, A. Tishechkin, DNA Extract MSC-2161, EXO-00684, 1: 30–31.iii.2009, A. Tishechkin, DNA Extract MSC-2414, EXO-00643 (AKTC, MSCC, FMNH).

###### Diagnostic description.

Length: 1.3–1.6mm, width: 1.2–1.4mm; body subquadrate, weakly convex, glabrous; color rufescent, shining; head with frons elevated over and between antennal bases, narrowly depressed at middle, interocular margins weakly convergent dorsad, frontal disk with only very fine ground punctation, frontal stria absent or represented by short fragment along upper margin of eyes, supraorbital stria weakly impressed to absent; antennal scape very short, club large, elongate oval; epistoma weakly depressed beneath antennal bosses, with weak tubercle medially, apical margin truncate; labrum 2.5–3×wider than long, apical margin shallowly emarginate; mandibles short, each with small, acute basal tooth; pronotum transverse, with sides weakly convergent in basal half, arcuate to apex, lateral marginal stria descending to ventral edge about one-third behind anterior corner, extending around corner nearly meeting anterior marginal stria, which is recurved posterad for about one-sixth pronotal length behind each eye, the median pronotal gland opening displaced posterad to near its apex, lateral submarginal stria nearly complete, diverging from margin to front, marginal bead somewhat swollen in anterior half, disk depressed along submarginal stria, with ground punctation very fine, inconspicuous, with few faint, or no secondary punctures laterally; elytra with complete upper epipleural striae, lower epipleural stria may be weak or fragmented, outer and inner subhumeral striae absent, dorsal stria 1 obsolete in apical half to one-third, stria 2 more nearly complete, 3^rd^ stria present in basal half or slightly more, 4^th^ stria present in basal one-fourth or less, basally arched toward scutellum, 5^th^ stria absent, sutural stria usually abbreviated basally, but may be connected to arch of 4^th^ stria, elytral disk with small secondary punctures in most of apical half, denser toward midline; prosternal keel moderately narrow, convex, emarginate at base, with carinal striae divergent anterad; prosternal lobe short, deflexed, apical margin broadly arcuate, marginal stria more or less complete; mesoventrite produced at middle, with marginal stria complete; mesometaventral stria present, sinuate, weakly crenulate across middle, continued by inner lateral metaventral stria, extending obliquely posterad toward middle of metacoxa, abbreviated at apex, outer lateral metaventral stria short, curved behind mesocoxa; metaventral disk moderately coarsely punctate at sides, impunctate at middle; abdominal ventrite 1 with single, complete lateral stria, ventrite lacking transverse anterior marginal stria, disk impunctate at middle, ventrites 2–5 with fine, moderately dense punctures across width; protibia narrow, lacking median marginal denticle, margin more or less straight, serrulate; mesofemur with posterior marginal stria not curving around apical margin; mesotibia with subapical marginal spine and oblique, basal submarginal ridge; outer metatibial margin smooth; propygidium lacking basal stria, with fine ground punctation very sparsely interspersed with small, ocellate secondary punctures, propygidial gland openings inconspicuous; pygidium with fine ground punctation, with few coarser secondary punctures in basal half of disk. Male genitalia (Figs 57C–E, L–M): T8 as in *Baconia gibbifer* (Fig. 55A); S8 subquadrate, slightly wider than long, basal emargination shallow, arcuate, apices with two transverse, well-sclerotized disks bearing faint fringe of apical setae, oblique strengthening ridges extending from disks to basal midpoint; T9 with dorsal lobes broad, proximal apodemes obsolete, apices prolonged, divergent, sinuate, deflexed, bearing single subapical seta; S9 stem narrow, strongly dorsoventrally keeled, head gradually widened to apex, apical emargination shallow and sinuate, desclerotized medially; tegmen with sides subparallel in basal fourth, slightly concavely narrowed to very narrow apices, rather thick in lateral aspect, curving ventrad in apical half, with eversible subapical denticles ventrally; median lobe simple, about one-fourth tegmen length; basal piece one-third tegmen length.

###### Remarks.

Most individuals of this species can be recognized by the presence of a distinct epistomal tubercle (Fig. 56B). However, this is more conspicuous in the ‘typical’ populations from Costa Rica and Panama. Where this is not as distinct, the species also exhibits a relatively impunctate frons and pronotum and a markedly swollen pronotal marginal bead (Fig. 56C). The species’ fundamental character is a highly distinctive male 8^th^ sternite in which the apices form strongly sclerotized disk-like plates. The elongate and downturned apices of the 9^th^ tergite are also unique (though somewhat similar to those in *Baconia globosa*, below.) We restrict the type series to localities in Costa Rica and Panama, where the epistomal tubercle is relatively distinct.

###### Etymology.

This species is named for the epistomal tubercle that characterizes typical examples.

##### Baconia globosa sp. n.

http://zoobank.org/0AC05062-3162-4523-BE77-0555941240DA

http://species-id.net/wiki/Baconia_globosa

Figs 56D 57F–G Map 18

###### Type locality.

ECUADOR: Orellana:Res. Ethnica Waorani [0.67°N, 76.43°W].

###### Type material.

**Holotype male**: “**ECUADOR: Depto. Orellana:** Res. Ethnica Waorani, 1km S Onkone Gare Camp, Trans. Ent., 0°39'10"S, 76°26'W, 220m, 22 June 1996, T.L. Erwin et al. collectors” / “Insecticidal fogging of mostly bare green leaves, some with covering of lichenous or bryophytic plants in terra firme forest. Project MAXUS **Lot 1562 Trans. 5 Sta. 2**” / “Caterino/Tishechkin Exosternini Voucher EXO-00817” (USNM).

###### Diagnostic description.

Length: 1.5mm, width: 1.4mm; body broadly oval, widest anterad, convex, glabrous; color rufescent, shining; head with frons slightly elevated over antennal bases, weakly depressed at middle, interocular margins strongly convergent dorsad, ground punctation fine, inconspicuous, with coarser punctures mainly at sides and along dorsal margin, frontal stria fragmented to absent, supraorbital stria present as several confluent punctures; antennal scape short, club rather large, round; epistoma truncate; labrum about 4×wider than long, apical margin emarginate; mandibles short; pronotum with sides weakly convergent in basal half, rounded to apex, lateral marginal and lateral submarginal striae merging behind anterior corner, detached from median part of anterior marginal stria, the sides of which recurve posterad about one-eighth behind anterior pronotal margin; pronotal disk narrowly depressed along anterolateral margin, ground punctation fine, very sparse, with small secondary punctures in lateral thirds, few larger punctures extending mediad along basal margin; elytra with upper epipleural stria complete, lower epipleural stria somewhat fragmented, outer subhumeral stria absent, inner subhumeral stria present only at extreme base, dorsal striae 1–2 obsolete from posterior one-fourth, 3^rd^ stria present in basal one-third, 4^th^ stria slightly longer, curving mediad toward scutellum at base, 5^th^ stria absent, sutural stria very short, present only along middle one-fourth of suture, elytral disk with small secondary punctures in most of apical third; prosternal keel narrow, convex, weakly emarginate at base, with more or less complete carinal striae subparallel or diverging slightly to front; prosternal lobe about one-half keel length, apical margin rounded, marginal stria obsolete at sides; mesoventrite weakly produced at middle, with marginal stria complete; mesometaventral stria transverse, crenulate, continuous laterally with inner lateral metaventral stria, which extends obliquely posterad toward inner corner of metacoxa, outer lateral metaventral stria about half as long as inner, subparallel, continuing marginal mesoventral stria; metaventral disk moderately coarsely punctate at sides, impunctate at middle; abdominal ventrite 1 with single, complete lateral stria, curved inward posterad, disk impunctate at middle, ventrites 2-5 with fine punctures across width; protibia narrow, lacking median and basal marginal denticles, margin straight, serrulate; mesofemur with posterior marginal stria curving anterad along apical margin; mesotibia with one fine marginal spine and short, oblique basal submarginal ridge; outer metatibial margin smooth; propygidium lacking basal stria, with fine ground punctation interspersed with dense, ocellate secondary punctures, propygidial gland openings inconspicuous; pygidium with fine ground punctation, with secondary punctures sparsely scattered, principally in basal half. Male genitalia (Figs 57F–G): T8 as in *Baconia gibbifer* (Fig. 55A); S8 short, broad, sides slightly divergent to apex, apieces obliquely subtruncate, bearing fine apical setae, inner corners slightly produced, median emargination narrowly acute; T9 with dorsal plates rather broad near base, proximal apodemes short, sides uneven, narrowed strongly distally, apices prolonged, sinuate, somewhat digitiform, strongly bent ventrad, bearing single, inconspicuous, subapical seta on each side, ventrolateral apodemes nearly meeting at midline about one-third from apex, bluntly dentate; S9 stem narrow [broken off in type, probably dorsoventrally keeled], head gradually widened to apex, apical emargination shallow and sinuate; tegmen indistinguishable from that of *Baconia tenuipes*.

###### Remarks.

This species may be considered somewhat generalized in the group. Its body is weakly depressed and broad (Fig. 56D), with the sides only weakly rounded. It is similar to *Baconia piluliforme* in the absence of the 5^th^ elytral stria and the presence of a crenulate mesometaventral stria. However, it does not have the strongly narrowed prosternal keel, has the lateral stria of the 1^st^ abdominal ventrite longer, and has a relatively long 4^th^ elytral stria which is detached from the base of the sutural stria. Confident identification requires examination of male genitalia to confirm the unusual shape of the 9^th^ tergite, with ventrally pointed, digitiform apices.

###### Etymology.

This species is named in reference to its rather broadly oval body form.

#### *Baconia insolita* group

The *Baconia insolita* group is primarily characterized by a small, elongate, parallel-sided body form, and the presence of very finely setigerous punctures on most body surfaces (see e.g. Fig. 60C). All the species have the frons swollen to at least some degree, and have most of the elytral striae complete, straight and slightly convergent toward the front. The male genitalia, where known, span a surprisingly broad range of variability and are impossible to collectively characterize, primarily due to the highly divergent genitalia of *Baconia insolita*. The one character that they have in common, though to different degrees, is the well-developed and strongly hook-like ventrolateral apodemes of the 9^th^ tergite.

Previously, *Baconia burmeisteri* was assigned to the subgenus *Binhister* Cooman, along with the Asian species *Baconia barbarus* and *Baconia chujoi*. However, this grouping is clearly artificial, with the latter species clearly more closely related to *Baconia aeneomicans*, and *Baconia burmeisteri* very close to *Baconia insolita*. Male genitalic characters separate these groups widely.

##### Baconia insolita

(Schmidt, 1893) comb. n.

http://species-id.net/wiki/Baconia_insolita

**Map 19.**
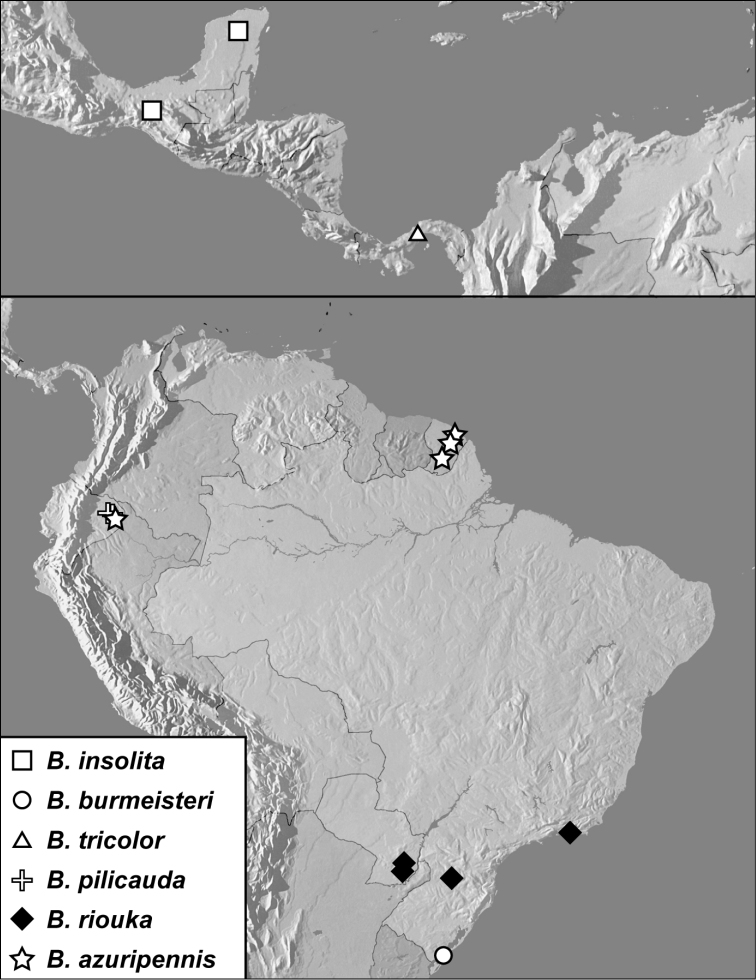
*Baconia insolita* and *Baconia riouka* group records.

Figs 2D 58A–C 59 Map 19

Phelister insolitus Schmidt, 1893a: 12.

###### Type locality.

MEXICO [exact locality unknown].

###### Type material.

**Lectotype**, sex undetermined, here designated (ZMHB): “Mexique” / “Type” / “*insolitus*” / “*insolitus* Schm.” / “coll. J.Schmidt” / “LECTOTYPE *Phelister insolitus* Schmidt, 1893, M.S.Caterino & A.K.Tishechkin des. 2010”. **Paralectotypes** (8): same data as type (ZMHB, MNHN, BMNH). This species was described from an unspecified number of specimens, which were evidently distributed widely, and the lectotype designation fixes primary type status on one of the original specimens.

###### Other material.

**MEXICO**: 1: **Chiapas**: P. N. Sumidero, 1000 m, 29.v.1990, FIT, H. & A. Howden (CMNC); 1: **Yucatan**, 2 km E Chichen Itza, 20 m, 20.vii.1983, S.&J. Peck, seas. forest litter (CMNC) 9: [country record only] (BMNH, FMNH, MHNG).

###### Diagnostic description.

Length: 1.7–1.8mm, width: 1.2–1.3mm; body elongate, sides subparallel, narrowing slightly to front, weakly depressed, conspicuously punctate on most surfaces, most punctures bearing single short, fine seta (usually abraded on dorsal and ventral surfaces, more persistent on pygidia); color rufopiceous, shining; head with frons produced, elevated over antennal bases, epistoma strongly convex, weakly depressed at middle by high density of punctures, frontal stria broadly interrupted between antennal bases, present along inner edge of eyes, supraorbital stria absent; antennal scape short, subpyramidal, club short, sides rounded, apex subtruncate; epistoma straight across distal margin; labrum about 2.5× wider than long, rounded at sides, apical margin entire; mandibles strong, rather bulky, each with median tooth; apical maxillary palpomeres slightly widened, markedly setose; pronotum with sides subparallel in basal three-fourths, rather abruptly narrowed to apex, lateral marginal stria continuous around sides and front, lateral submarginal stria absent; pronotal disk weakly depressed in anterior corners, punctation of disk coarse, more or less uniform, just slightly denser toward sides; elytra with inner epipleural stria complete, subhumeral striae absent, dorsal striae 1-3 complete, striae 4-5 varied from nearly complete to highly fragmented, sutural stria more or less complete, elytral disk with sparse secondary punctures throughout, denser in apical fourth; prosternal keel narrow, depressed between coxae, very shallowly emarginate at base, carinal striae convergent at basal third, diverging posterad and anterad, often united at base; prosternal lobe short, less than half keel length, apically shallowly emarginate, marginal stria more or less complete but often slightly fragmented; mesoventrite not produced at middle, marginal stria usually complete, may be fragmented; mesometaventral stria absent from middle, inner lateral metaventral stria extending from inner corner of mesocoxa to middle of metacoxa, outer lateral metaventral stria present or absent, when present paralleling inner stria for brief anterior distance; mesometaventral and abdominal ventral disks with rather sparse secondary punctures throughout; abdominal ventrite 1 with complete inner lateral stria and outer lateral stria in apical half; protibia rather broad, curving, with 6-7 marginal denticles, margin not serrulate between; mesotibia with 3-4 marginal spines, the basal-most spines weak; outer metatibial margin spineless, setose; propygidium lacking basal stria, propygidial gland openings evident about one-third from anterior margin and one-fourth from lateral margins; propygidium and pygidium with coarse punctation throughout. Male genitalia (Fig. 59): T8 strongly reduced, halves separated along midline, inner lobes rounded, ventrolateral apodemes sclerotized, projecting beneath; S8 about as long as broad, halves approximate along inner middle third, sides strongly curved dorsomediad, forming apicolateral rim, apical velar membrane bearing sclerotized median disk; T9 with proximal apodemes narrow, about one-third total length, dorsal lobes evenly narrowed to apices, ventrolateral apodemes very prominent, recurved acutely nearly to base; T10 entire; S9 broader than aedeagus along entire length, base rounded, apex shallowly emarginate; tegmen elongate, narrow, sides sinuate, only very weakly curved ventrad near apex; median lobe simple, about one-fourth tegmen length; basal piece narrower than tegmen, about one-third its length.

**Figure 58.**
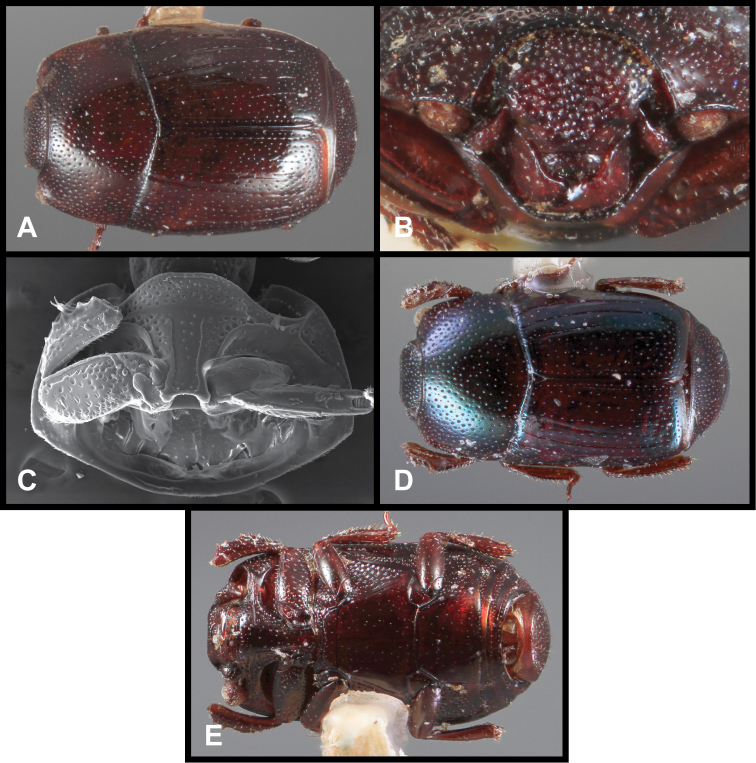
*Baconia insolita* group. **A** Dorsal habitus of *Baconia insolita* **B** Frons of *Baconia insolita* **C** Prosternum of *Baconia insolita* **D** Dorsal habitus of *Baconia burmeisteri* **E** Ventral habitus of *Baconia burmeisteri*.

**Figure 59.**
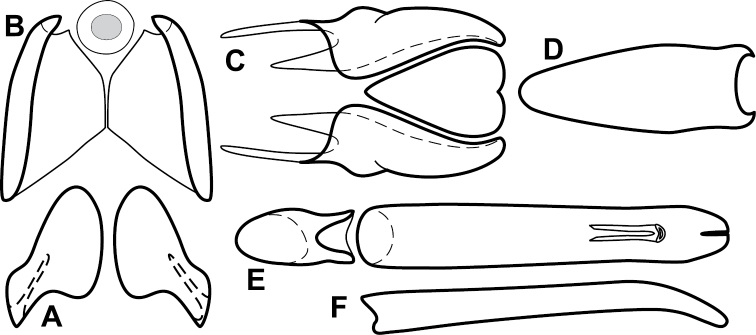
Male genitalia of *Baconia insolita*. **A** T8 **B** S8 **C** T9 & T10 **D** S9 **E** Aedeagus, dorsal view **F** Aedeagus, lateral view.

###### Remarks.

This species and the following are unmistakable in their small, punctate, slightly pubescent, parallel-sided form, convex, densely punctate frons (Fig. 58B) and epistoma, weakly emarginate prosternal keel (Fig. 58C), and strongly dentate mandibles. *Baconia insolita* can be further distinguished by its non-metallic, rufobrunneus coloration. Many typically diagnostic characters are highly varied including striation of the elytra, prosternum, and meso-metaventrites. The male genitalia of *Baconia insolita* is remarkably autapomorphic, with the divided T8, velar sclerite on the apex of S8, the strongly recurved ventrolateral apodemes of T9, and the wide spiculum gastrale resembling nothing else in the genus. Although the male of *Baconia burmeisteri* is unknown, its external similarity to *Baconia insolita* suggests the male’s genitalia would be similar. However, the male genitalia of other species we assign to this group on the basis of seemingly reliable external characters are quite different, so it remains to be seen.

##### Baconia burmeisteri

(Marseul, 1870)

http://species-id.net/wiki/Baconia_burmeisteri

Figs 58D–E Map 19

Pachycraerus burmeisteri Marseul, 1870: 76; *Baconia (Binhister) burmeisteri*: Mazur 1997: 26.

###### Type locality.

BRAZIL [exact locality unknown].

###### Type material.

**Lectotype**, sex undetermined, here designated (MNHN): “*Carcinops* [sic] *burmeisteri*, Brésil, Hag, 67” / “Bresil” / “G.H.62” / “Museum Paris Coll. de Marseul 2842-90” / “LECTOTYPE *Pachycraerus burmeisteri* Marseul, 1870, M.S.Caterino & A.K.Tishechkin des. 2010”. This species was described from an unspecified number of specimens, and the lectotype designation fixes primary type status on the only known original specimen.

###### Other material.

**BRAZIL**: 1: Rio Grande [do Sul]: 22.ii.1884, tabac, A. Grouvelle (BMNH).

###### Diagnostic description.

Length: 1.6mm, width: 1.1mm; body elongate, sides subparallel, weakly depressed, conspicuously punctate on most surfaces, most punctures bearing single short, fine seta (usually abraded on dorsal and ventral surfaces, more persistent on pygidia); color weakly metallic blue-green, shining; head with frons produced in front, elevated over antennal bases, epistoma strongly convex, weakly depressed at middle by high density of punctures, frontal stria broadly interrupted between antennal bases, present along inner edge of eyes, supraorbital stria absent; antennal scape short, subpyramidal, club short, sides rounded, apex subtruncate; epistoma straight across distal margin; labrum about 2× wider than long, rounded at sides, apical margin entire; mandibles strong, rather bulky, each with median tooth; pronotum with sides subparallel in basal three-fourths, rather abruptly narrowed to apex, lateral marginal stria continuous around sides and front, lateral submarginal stria very close to marginal stria, merging near anterior corner; pronotal disk weakly depressed in anterior corners, punctation of disk coarse, more or less uniform, slightly denser toward sides; elytra with inner epipleural stria complete, outer subhumeral stria absent, inner subhumeral stria very short, basal, dorsal striae 1-4 complete, 5^th^ stria absent, sutural stria more or less complete, though slightly abbreviated at apices; elytral disk with sparse secondary punctures throughout, denser in apical fifth; prosternal keel narrow, depressed between coxae, very weakly produced, carinal striae subparallel in basal third, united at base, diverging anterad; prosternal lobe short, less than half keel length, truncate to shallowly emarginate apically, marginal stria obsolete at sides; mesoventrite shallowly emarginate at middle, marginal stria complete; mesometaventral stria absent from middle, inner lateral metaventral stria continuing from mesoventral marginal stria to middle of metacoxa, outer lateral metaventral stria paralleling inner stria for short anterior distance; mesometaventral and abdominal ventral disks with sparse secondary punctures throughout; abdominal ventrite 1 with complete inner lateral stria and outer lateral stria in apical half; protibia rather broad, curving, with 5–6 marginal denticles, margin not serrulate between; mesotibia with 3–4 marginal spines, the basal-most weak; outer metatibial margin with 1 or 2 weak submarginal spines, setose; propygidium lacking basal stria, propygidial gland openings evident about one-fourth from anterior and lateral margins; propygidium and pygidium with coarse punctation throughout. Male genitalia: not known.

###### Remarks.

This species is very similar externally to *Baconia insolita* in most characters. Aside from their widely disjunct distributions, *Baconia burmeisteri* can easily be recognized by its metallic coloration (Fig. 58D), weakly produced prosternal keel (Fig. 58E) and slightly more convex form.

The localities for this species are poorly documented, and the type locality cannot be specified beyond the country of Brazil. Grouvelle’s collection from ‘Rio Grande’ is most likely from Rio Grande do Sul, as many of the species he collected and described originated in this state.

##### Baconia tricolor sp. n.

http://zoobank.org/A9C84D79-CC14-477C-9EAF-73AD1D9AA4E1

http://species-id.net/wiki/Baconia_tricolor

Figs 1B, F 2A–B 60A–C 61A–D, F–G Map 19

###### Type locality.

PANAMA: Colón: San Lorenzo Forest [9.28°N, 79.97°W].

###### Type material.

**Holotype male**: “**PANAMA**: **Colón Pr.**, San Lorenzo Forest. 9°17'N, 79°58'W. F.I.T., 21m, 10 d.FL-I1-C21a. 18.x.2003 R.Didham, L.Fagan. IBISCA” / “Caterino/Tishechkin Exosternini Voucher EXO-00471” (FMNH). **Paratypes** (16): **PANAMA**:1: **Colón**: P. N. San Lorenzo, STRI Crane Site, 9°17'N, 79°58'W, 14 m, 12–23.ix.2004, FIT, M. Rapp, 5: 28 m, 14-26.vii.2004, FIT, M. Rapp, 1: 28 m, 18.x.2003, FIT, R. Didham & L. Fagan, 1: 28 m, 19–25.v.2004, FIT, R. Didham, 1: 14 m, 23.viii–2.ix.2004, FIT, M. Rapp, 1: 14 m, 23.x.2003, FIT, R. Didham & L. Fagan, 1: 28 m, 23.x.2003, FIT, R. Didham & L. Fagan, 1: 21 m, 26.iii–5.iv.2004, FIT, M. Gonzales, 1: 14 m, 29.x.2003, FIT, L. Fagan & R. Didham, 1: 7 m, 29.x.2003, FIT, L. Fagan & R. Didham, 1: 28 m, 3-13.viii.2004, FIT, M. Rapp, 1: 21 m, 3–13.x.2004, FIT, M. Rapp (AKTC, GBFM, MSCC).

###### Diagnostic description.

Length: 1.4–1.5mm, width: 1.0–1.1mm; body elongate, sides subparallel, weakly depressed, conspicuously punctate on most surfaces, most punctures bearing single short, fine seta (may be abraded on dorsal and ventral surfaces, more persistent on pygidia); elytra faintly metallic blue, head and pronotum metallic blue-green, apical margin of elytra, pygidia and venter rufescent; frons produced in front, elevated above and between antennal bases, weakly depressed dorsad, uniformly punctate, frontal and supraorbital striae absent; antennal scape short, strongly bent at base, club rounded, slightly elongate; epistoma convex, straight across distal margin; labrum about 3× wider than long, apical margin deeply and distinctly emarginate; mandibles short, each with median tooth; apical maxillary palpomeres slightly widened; pronotum with sides subparallel to slightly narrowing in basal three-fourths, abruptly narrowed to apex, lateral marginal stria continuous around sides and front, lateral submarginal stria very close to marginal, subcarinate, merging near anterior corner; pronotal disk weakly depressed along inner edge of anterior half of lateral submarginal stria; punctation of pronotal disk coarse, more or less uniform; elytra with two complete epipleural striae, outer subhumeral stria absent, inner subhumeral stria usually represented by short basal fragment, dorsal striae 1–5 present to base, variably abbreviated in apical fourth, sutural stria present in apical two-thirds, may be abbreviated apically, elytral disk with sparse secondary punctures throughout, slightly denser in apical fourth; prosternal keel narrow, weakly convex, narrowly, shallowly emarginate at base, carinal striae convergent in basal half, united by arch just distad middle; prosternal lobe about half keel length, apically truncate, marginal stria obsolete at sides; mesoventrite narrowly produced at middle, marginal stria usually complete, may be narrowly interrupted medially; mesometaventral stria arched forward at middle, continuous with inner lateral metaventral stria from inner corner of mesocoxa to middle of metacoxa, short fragment of outer lateral metaventral stria present near base; mesometaventral and abdominal ventral disks very finely and sparsely punctate; abdominal ventrite 1 with complete inner lateral stria and median fragments of outer lateral stria; protibia with 3-4 marginal denticles, the basal-most usually weak, margin finely serrulate between; mesotibia with 2–3 marginal spines; outer metatibial margin smooth; propygidium lacking basal stria, propygidial gland openings evident about one-third distance from anterior and lateral margins; propygidium and pygidium with coarse punctation throughout. Male genitalia (Figs 61A–D, F–G): T8 short, basal emargination deep, subacute, sides rounded, apical emargination narrowly acute, ventrolateral apodemes transversely convergent basally, barely projecting distad; S8 about as long as broad, halves approximate at inner bases only, rapidly narrowed apically, apical guides widening to apex, apical membrane lightly sclerotized to form bilateral velar disks which nearly meet along the midline; T9 with proximal apodemes narrow, about one-half total length, dorsal lobes evenly narrowed to apices, ventrolateral apodemes very prominent, acuminate, strongly recurved proximad; T10 entire; S9 parallel-sided, apices short, divergent, quadrate; tegmen parallel-sided in basal third, strongly narrowed to apex, thick in lateral aspect, weakly curving ventrad over much of length; median lobe nearly one-half tegmen length, with very short proximal apodemes; basal piece broad, about one-third tegmen length.

**Figure 60.**
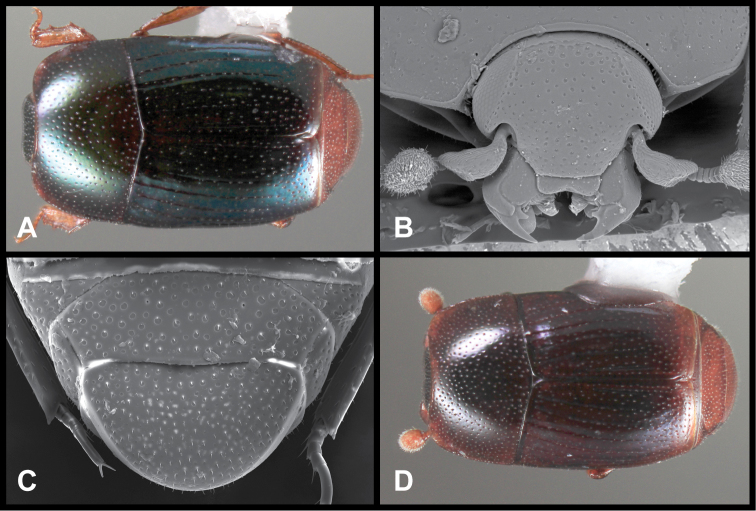
*Baconia insolita* group. **A** Dorsal habitus of *Baconia tricolor* **B** Frons of *Baconia tricolor* **C** Pygidia of *Baconia tricolor* **D** Dorsal habitus of *Baconia pilicauda*.

**Figure 61.**
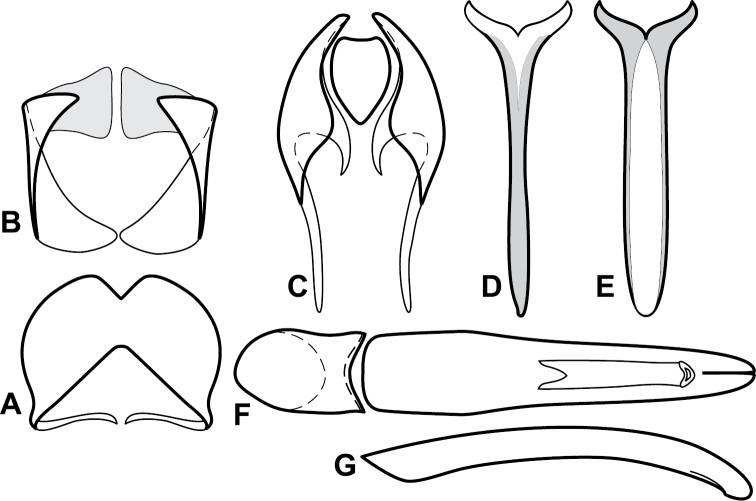
Male genitalia of *Baconia insolita* group. **A** T8 of *Baconia tricolor* **B** S8 of *Baconia tricolor* **C** T9 & T10 of *Baconia tricolor* **D** S9 of *Baconia tricolor* **E** S9 of *Baconia pilicauda* **F** Aedeagus, dorsal view of *Baconia tricolor* **G** Aedeagus, lateral view of *Baconia tricolor*.

###### Remarks.

This species is fairly distinct in its coloration alone (Fig. 60A). However, there are a number of more or less similar (i.e., narrow and subcylindrical) species in this group and others. The best characters for separating it besides its coloration are the fine setae, preserved almost invariably on the pygidia if not elsewhere (Figs 60A, C), the convex frons, and the narrowly emarginate labrum (Fig. 60B).

###### Etymology.

The greenish pronotum, blue elytra, and rufescent pygidia of this species make for a distinctly tricolored dorsal appearance.

##### Baconia pilicauda sp. n.

http://zoobank.org/72E580F2-BA87-485E-A944-E1824D0A4849

http://species-id.net/wiki/Baconia_pilicauda

Figs 60D 61E Map 19

###### Type locality.

ECUADOR: Orellana:Res. Ethnica Waorani [0.67°N, 76.43°W].

###### Type material.

**Holotype male**: **ECUADOR: Depto. Orellana**:Res. Ethnica Waorani, 1km S Onkone Gare Camp, Trans. Ent., 0°39'10"S, 76°26'W, 220m, 12 February 1995, T.L. Erwin et al. collectors” / “Insecticidal fogging of mostly bare green leaves, some with covering of lichenous or bryophytic plants in terra firme forest. Project MAXUS **Lot 1047 Trans. 5 Sta. 8**” / “Caterino/Tishechkin Exosternini Voucher EXO-02733” (USNM). **Paratypes** (4): **ECUADOR**: **Orellana**: 1: Res. Ethnica Waorani, 1 km S Onkone Gare Camp, Trans. Ent., 0°39'10"S, 76°26'W, 220 m, 1: 25.vi.1996, fogging, T. Erwin (USNM), 1: 3.x.1996, fogging, T. Erwin (USNM); 1: Est. Biodiv. Tiputini, 0°37'55"S, 76°08'39"W, 220–250 m, 21.x.1998, fogging, mostly bare green leaves, some with covering of lichenous or brophytic plants, T. Erwin (USNM), 1: 9.ii.1999, fogging, T. Erwin (USNM).

###### Diagnostic description.

Length: 1.4–1.7mm, width: 0.9–1.2mm; body elongate, sides subparallel, weakly depressed, conspicuously punctate on most surfaces, most punctures bearing single short, fine seta (may be abraded on dorsal and ventral surfaces, more persistent on pygidia); elytra faintly metallic blue, head and pronotum rufobrunneus, apical margin of elytra, pygidia and venter rufescent; frons produced in front, elevated above and between antennal bases, weakly depressed dorsad, uniformly punctate, frontal and supraorbital striae absent; antennal scape short, strongly bent at base, club rounded, slightly elongate; epistoma convex, straight across distal margin; labrum about 3× wider than long, apical margin deeply and distinctly emarginate; mandibles short, each with median tooth; apical maxillary palpomeres slightly widened; pronotum with sides subparallel to slightly narrowing in basal three-fourths, abruptly narrowed to apex, lateral marginal stria continuous around sides and front, lateral submarginal stria very close to marginal stria, subcarinate, merging near anterior corner; pronotal disk weakly depressed along inner edge of anterior half of lateral submarginal stria; punctation of pronotal disk coarse, more or less uniform; elytra with two complete epipleural striae, outer subhumeral stria absent, inner subhumeral stria varied, present as basal fragments to absent, dorsal striae 1–5 present to base, variably abbreviated in apical fourth, sutural stria present only in apical half, elytral disk with sparse secondary punctures throughout, slightly denser in apical fourth; prosternal keel narrow, weakly convex, narrowly, shallowly emarginate at base, carinal striae subparallel in basal half, united near middle; prosternal lobe nearly half keel length, apically truncate, marginal stria obsolete at sides; mesoventrite narrowly produced at middle, marginal stria usually complete to interrupted medially; mesometaventral stria arched forward at middle, continuous with inner lateral metaventral stria from inner corner of mesocoxa to middle of metacoxa, very short fragment of outer lateral metaventral stria present near base; mesometaventral and abdominal ventral disks very finely and sparsely punctate; abdominal ventrite 1 with complete inner lateral stria and median fragments of outer lateral stria; protibia rather broad, with 4 marginal denticles, basal-most weak, margin finely serrulate between; mesotibia with 4 marginal spines, basal-most weak; outer metatibial margin smooth; propygidium lacking basal stria, propygidial gland openings evident about one-third distance from anterior and lateral margins; propygidium and pygidium with coarse punctation throughout. Male genitalia essentially as in *Baconia tricolor*, but stem of S9 tending to be wider (Fig. 61E).

###### Remarks.

*Baconia pilicauda* and *Baconia tricolor* are extremely closely related, and could almost be lumped into a single polymorphic species. However, there are consistent differences in body shape and general appearance, with *Baconia pilicauda* less brightly colored and slightly broader (Fig. 60D), as well as slightly larger. Genitalic differences are very minor.

All of the specimens of *Baconia pilicauda* were collected in canopy samples in the Napo region of Ecuador. Given the large amount of more general (i.e., subcanopy) collecting that has been done in the same vicinity, it seems likely that this species is a canopy specialist.

###### Etymology.

This species’ name refers to its distinctly setose pygidium.

#### *Baconia riouka* group

**Figure 62.**
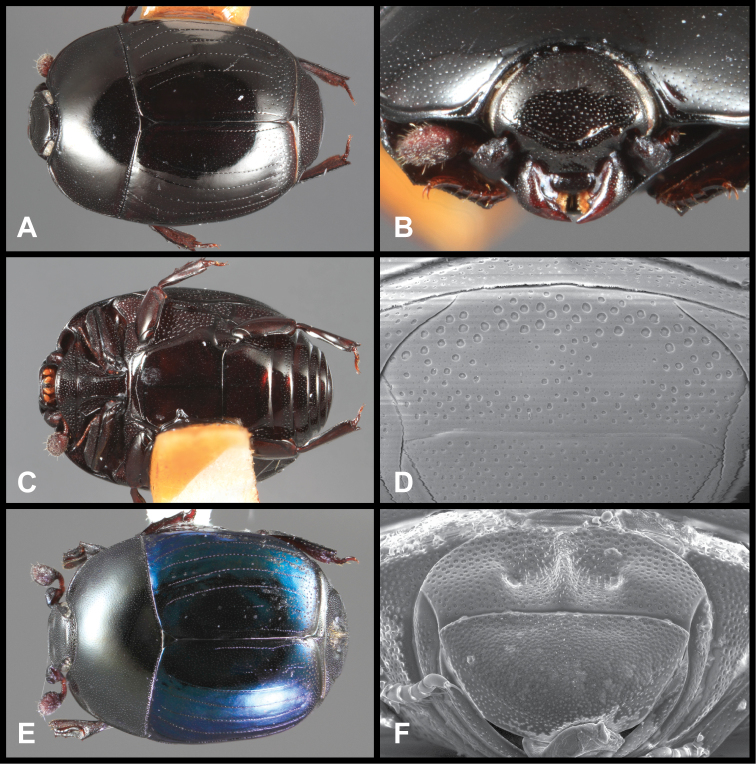
*Baconia riouka* group. **A** Dorsal habitus of *Baconia riouka* **B** Frons of *Baconia riouka* **C** Ventral habitus of *Baconia riouka* **D** Propygidium of male *Baconia riouka* **E** Dorsal habitus of *Baconia azuripennis* **F** Pygidia of male *Baconia azuripennis*.

We place two species in the *Baconia riouka* group, *Baconia riouka* (Marseul) and *Baconia azuripennis*, sp. n. They are rather different from each other, but clearly related and highly divergent from other *Baconia* species. The most significant character they share is a sexually dimorphic propygidium, although the manifestation of this differs greatly between them. In *Baconia riouka* the male propygidium exhibits a pair of simple micropunctate depressions on either side (Fig 62D). In *Baconia azuripennis* these depressions are much deeper, but are separated by a remarkable setose median ridge (Fig. 62F), and the central part of the propygidium and the entire pygidium are densely punctate and bearing alutaceous microsculpture. Beyond this strong synapomorphy, the two species share a transversely, arcuately produced frons bearing a complete frontal stria (Fig. 62B), frontal disk with weak bilateral depressions, subdepressed and rather broadly rounded body form (Figs 62A, E), complete inner subhumeral elytral stria, and a nearly complete outer subhumeral stria. Male genitalia share a short, rather broad spiculum gastrale (S9).

##### Baconia riouka

(Marseul, 1861)

http://species-id.net/wiki/Baconia_riouka

Figs 62A–D 63A–C, G, I–J Map 19

Phelister riouka Marseul, 1861: 158; *Pseudister riouka*: Bickhardt 1917: 165; *Baconia riouka*: Mazur 1997: 25.

###### Type locality.

BRAZIL: Rio de Janeiro [22.9°S, 43.2°W].

###### Type material.

**Lectotype**, sex undetermined, here designated (NHRS): “Rio. Jan” / “F. Sahlb.” / “Typus” / “Type” / “6910 E91 +” / “LECTOTYPE *Phelister riouka* Marseul M.S.Caterino & A.K.Tishechkin des. 2010”. This species was described from an unspecified number of specimens, and the lectotype designation fixes primary type status on the only known original specimen.

###### Other material.

**PARAGUAY:** 1: **Itapua**, 17 km W Karonay, San Rafael Reserve, 26°45'53"S, 55°50'37"W, 90–110m, 18-21.xi.2000, FIT, Z.H.Falin (SEMC); 1: **Cazaapa**, Hermosta, prop. Sosa family, San Rafael Reserve, 26°19'15"S, 55°44'55"W, 90m, 3-6.xii.2000, FIT, Z.H.Falin (SEMC). 2: **BRAZIL: Santa Catarina**, Nova Teutonia, i.1973, F. Plaumann (FMNH).

###### Diagnostic description.

**Figure 63.**
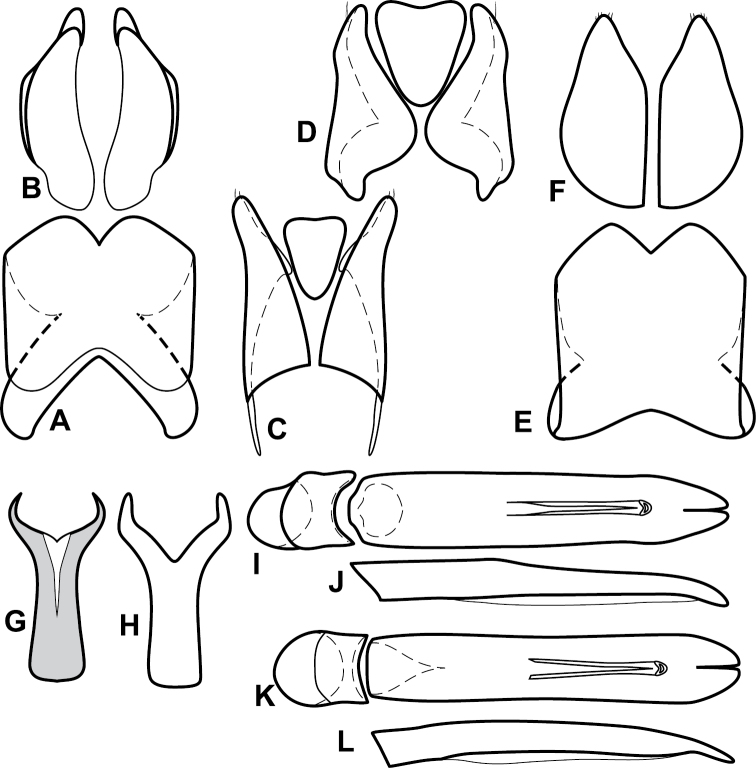
Male genitalia of *Baconia riouka* group. **A** T8 of *Baconia riouka* **B** S8 of *Baconia riouka* **C** T9 & T10 of *Baconia riouka* **D** T9 & T10 of *Baconia azuripennis* **E** T8 of *Baconia azuripennis* **F** S8 of *Baconia azuripennis* **G** S9 of *Baconia riouka* **H** S9 of *Baconia azuripennis* **I** Aedeagus, dorsal view of *Baconia riouka* **J** Aedeagus, lateral view of *Baconia riouka* **K **Aedeagus, dorsal view of *Baconia azuripennis* **L** Aedeagus, lateral view of *Baconia azuripennis*.

Length: 2.4–2.5mm, width: 2.2–2.3mm; body broadly oval, moderately depressed, glabrous; color piceous, shining; frons broad, interocular margins convergent dorsad, frontal disk transversely, arcuately elevated above and between antennal bases, frontal stria absent along margin of eyes but complete across front, finely carinate, frontal disk broadly depressed behind, ground punctation conspicuous, rather dense; supraorbital stria absent; antennal scape short, thick, club slightly asymmetrically oblong; epistoma more or less convex, straight across apex; labrum about 2×wider than long, apical margin outwardly arcuate; mandibles narrow, convex, left mandible with small basal tooth, right mandible edentate; pronotum wide, sides evenly arcuate from base to apices, lateral marginal and submarginal striae close, parallel, separate to anterior corner, marginal stria usually rounding corner, ending free, submarginal stria joining or detached from anterior marginal stria, pronotal disk narrowly depressed along submarginal stria, largely impunctate at middle, with very small, sparse punctures becoming evident in lateral thirds; elytra with three epipleural striae, outer subhumeral stria present in basal half, weak, inner subhumeral stria complete, dorsal striae 1-3 complete, 4^th^ stria complete or interrupted medially, 5^th^ stria absent, sutural stria present in apical half to two-thirds, elytral disk punctate along apical margin; prosternal keel moderately broad, weakly convex, truncate or very shallowly emarginate at base, with more or less complete, subparallel carinal striae, separate throughout; prosternal lobe about one-half keel length, apical margin rounded, marginal stria present only at middle; mesoventrite broadly, shallowly emarginate, may be very weakly produced at middle, marginal stria interrupted for nearly width of prosternal keel; mesometaventral stria broadly arched forward, detached laterally from inner lateral metaventral stria, which curves obliquely posterad toward outer third of metacoxa, outer lateral metaventral stria very short; metaventral and abdominal disks impunctate at middle; abdominal ventrite 1 with single complete lateral stria; protibia tridentate, with median marginal denticle rather weak, margin serrulate; mesotibia with two small marginal spines; outer metatibial margin with fine subbasal denticle; propygidium short, wide, lacking basal stria, male propygidium biimpressed, each side with small area of dense ground punctation, devoid of secondary punctures, female propygidium only very weakly depressed, with secondary punctures more uniformly scattered; pygidium with fine ground punctation interspersed with small secondary punctures, separated by 2-3× their diameters. Male genitalia (Figs 63A–C, G, I–J): T8 slightly longer than wide, basal emargination moderately deep and narrow, sides subparallel, apical emargination subacute, ventrolateral apodemes well sclerotized, separated by half T8 width, projecting distad to about midpoint; S8 longer than broad, halves approximate at inner bases only, narrowing apically, apical guides weakly developed at sides, apices narrowly rounded, moderately well-sclerotized; T9 with proximal apodemes narrow, about one-third total length, apices weakly divergent, bearing a few fine setae, ventrolateral apodemes bluntly rounded beneath; T10 cordate, with distinct desclerotization along middle part of anteroposterior midline; S9 short, broadly subtruncate at base, narrowed to near apex, apical arms thin, divergent, apical emargination broadly arcuate; tegmen nearly parallel-sided throughout, subangulately narrowed to apex, in lateral view showing a pronounced dorsal hump near midpoint, becoming thinner apically, and with a distinct ventral longitudinal keel; median lobe about one-half tegmen length; basal piece short, about one-fifth tegmen length.

###### Remarks.

This species is highly distinctive, given the characters of the group, in addition to its black, non-metallic coloration of the elytra (Fig. 62A), and its male’s relatively simple propygidium with only shallow micropunctate depressions on each side (Fig. 62D). Apparently the shallow depressions of the male are associated with a median displacement of the propygidial glands, which are, in the female, clearly visible rather close to the sides, but not obviously visible in males.

##### Baconia azuripennis sp. n.

http://zoobank.org/D1B9139F-6ADB-4EB8-B76E-F4A7E7BF9357

http://species-id.net/wiki/Baconia_azuripennis

Figs 62E–F 63D–F, H, K–L Map 19

###### Type locality.

ECUADOR: Orellana:Res. Ethnica Waorani [0.67°N, 76.43°W].

###### Type material.

**Holotype male**: “**ECUADOR**: **Orellana**: Res. Ethnica Waorani, 1 km S Onkone Gare Camp, Trans. Ent., 0°39'10"S, 76°26'W, 220 m, 12 Febr 1995, T.L.Erwin et al., collectors” / “fogging, bare green leaves, some with covering of lichenous or bryophytic plants in terra firme forest. Project MAXUS **Lot 1041 Trans. 5 Sta. 2**” / “Caterino/Tishechkin Exosternini Voucher EXO-00409” (USNM). **Paratypes** (5): **FRENCH GUIANA**: 1:Montagne des Chevaux, 4°43'N, 52°24'W, 11.vii.2009, FIT, SEAG (CHND), 1:27.vi.2009, FIT, SEAG (MNHN), 1: 3.iv.2011, FIT, SEAG (MNHN); Savanne Matiti 4.0833°N, 52.6167°W, FIT, SEAG (FMNH); 1: Belvèdére de Saül, 3°1'22"N, 53°12'34"W, 31.xi.2010, FIT, SEAG (MSCC).

###### Diagnostic description.

Length: 2.5–2.7mm, width: 2.2–2.5mm; body broadly oval, moderately depressed, glabrous; elytra metallic blue, rest of body piceous, shining; frons broad, interocular margins convergent dorsad, frontal disk transversely, arcuately elevated above and between antennal bases, frontal stria absent along margin of eyes but complete across front, finely carinate, frontal disk bilaterally depressed behind, moreso in male, ground punctation conspicuous, rather dense; supraorbital stria absent; antennal scape short, thick, club slightly asymmetrically oblong; epistoma strongly recessed below frontal ridge, straight across apex; labrum about 2×wider than long, apical margin outwardly arcuate; mandibles narrow, convex, left mandible with small basal tooth, right mandible edentate; pronotum wide, sides evenly arcuate from base to apices, lateral marginal and submarginal striae close, parallel, separate to anterior corner, marginal stria usually rounding corner, ending free, submarginal stria usually detached from anterior marginal stria, disk largely impunctate at middle, with very small, sparse punctures becoming evident in lateral thirds; elytra with three epipleural striae, outer subhumeral stria present in basal two-thirds, inner subhumeral stria complete, dorsal striae 1-4 complete, 5^th^ stria generally present in apical half, may be absent, sutural stria present in apical half to two-thirds, elytral disk punctate along apical margin; prosternal keel moderately broad, weakly convex, truncate or very shallowly emarginate at base, carinal striae subparallel, separate throughout, usually obsolete in apical fifth; prosternal lobe about one-half keel length, apical margin rounded, marginal stria present only at middle; mesoventrite straight to weakly produced at middle, marginal stria interrupted for nearly width of prosternal keel; mesometaventral stria broadly arched forward, detached laterally from inner lateral metaventral stria, which curves obliquely posterad toward middle of metacoxa, outer lateral metaventral stria very short; metaventral and abdominal disks impunctate at middle; abdominal ventrite 1 with single complete lateral stria; protibia tridentate, outer margin serrulate; mesotibia with two small marginal spines; outer metatibial margin with fine subbasal denticle; propygidium lacking basal stria, that of female simply biimpressed, with uniformly scattered secondary punctures, that of male deeply impressed on either side of distinct median keel, entire middle of propygidium alutaceous, setose; pygidium of female with moderately dense small punctures, that of male densely alutaceous. Male genitalia (Figs 63D–F, H, K–L): T8 slightly longer than wide, basal emargination very shallow, basal rim slightly explanate, sides subparallel, apical emargination subacute, ventrolateral apodemes well sclerotized, separated by two-thirds T8 width, projecting distad about one-third from base; S8 longer than broad, halves approximate at inner bases, narrowing apically, apical guides very weakly developed at sides, apices narrowly rounded, moderately well-sclerotized; T9 broad, proximal apodemes very short, apices divergent, bluntly rounded, bearing a single conspicuous seta, ventrolateral apodemes bluntly rounded beneath; T10 cordate; S9 short, broadly subtruncate at base, narrowed to near apex, apical arms thin, divergent, apical emargination deeply subacute; tegmen sides weakly sinuate, narrowed near middle, in lateral view revealing a distinct ventral longitudinal keel; median lobe about one-half tegmen length; basal piece short, about one-fifth tegmen length.

###### Remarks.

While the similarities of this species to *Baconia riouka* are obvious, and a close relationship is supported by numerous characters, external and genitalic, males are easily separated by the extreme sexual dimorphism of the propygidium, and both sexes are distinct in the metallic blue coloration of the elytra of *Baconia azuripennis* (Fig. 62D). As above, the male modifications appear to be elaborations associated with the propygidial glands, which in the male are not otherwise visible, while being simple and apparent in the female.

###### Etymology.

This species is named for the strongly metallic blue elytra.

#### *Baconia famelica* group

**Figure 64.**
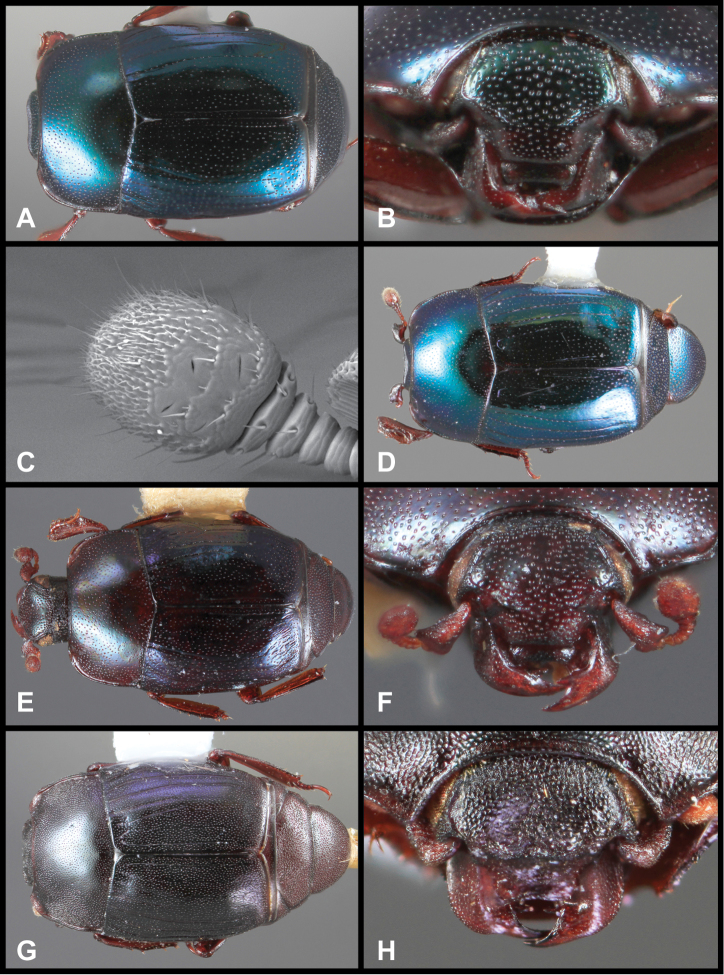
*Baconia famelica* group. **A** Dorsal habitus of *Baconia famelica* **B** Frons of *Baconia famelica* **C** Antennal club of *Baconia famelica* **D** Dorsal habitus of *Baconia grossii* **E** Dorsal habitus of *Baconia redemptor* **F** Frons of *Baconia redemptor* **G** Dorsal habitus of *Baconia fortis* **H** Frons of *Baconia fortis*.

This group contains eight quite divergent species, which are somewhat loosely united by several apparently reliable characters. The most consistent thing they share (except *Baconia cavifrons*) is an antennal club that is largely glabrous in the basal half (Fig. 64C), as opposed to the typically tomentose antennal club of other groups. In addition they are mostly rather parallel-sided, subdepressed, and usually distinctly punctate on most of the dorsum (e.g., Figs 64A, E, G). Most have a prosternal keel that is basally narrow, with the carinal striae subparallel at the base and frequently united along the basal margin. Several species have a complete and usually fine, transverse frontal carina. They tend to have very strong mandibles with large subbasal teeth. The lateral submarginal pronotal stria is usually present and strongly impressed close to the margin, with the marginal bead elevated or carinate. Also, the legs are frequently elongated, and are also broad and flat in a few species. There are no obvious characters of the male genitalia that unite the species, though they are all rather similar in a generalized way.

##### Baconia famelica sp. n.

http://zoobank.org/91087367-1D71-4A70-90CB-197147730CE4

http://species-id.net/wiki/Baconia_famelica

**Map 20.**
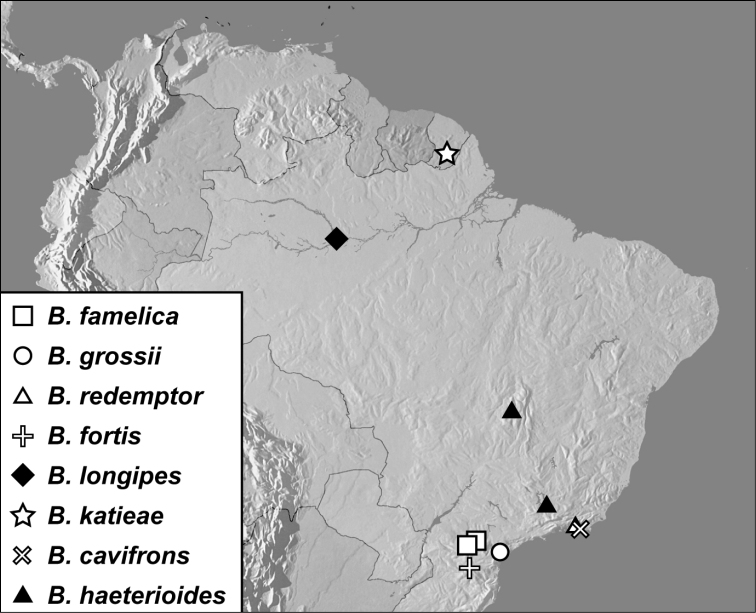
*Baconia famelica* group records.

Figs 64A–C 65 Map 20

###### Type locality.

BRAZIL: Paraná: Telêmaco Borba [24.31°S, 50.51°W].

###### Type material.

**Holotype male** : “BR-PR-Telêmaco Borba, Klabin Papel e Celulose, ESALQ-84 ethanol-baited FIT *Pinus taeda* stand, Flechtmann, C.A.H. col., 09/XI/1999” / “NKL34” / “Caterino/Tishechkin Exosternini Voucher EXO-00593” (UNESP). **Paratypes** (12): **BRAZIL**: 11: **Paraná**:same locality as type [24°18.9'S, 50°30.6'W] various dates and FIT lures as follows: 2: 17.xii.1999, ethanol-baited FIT, 2: 9.xi.1999, ESALQ-84 ethanol-baited FIT, 1: 24.xi.2000, multiple funnel ethanol-baited FIT, 1: 31.x.2003, ethanol baited multiple funnel FIT, 1: 28.xi.2003, sulcatol-baited multiple funnel FIT, 1: 28.xii.2003, ethanol+α-pinene+sulcatol baited multiple funnel FIT, 1: 6.i.2004, sulcatol+α-pinene-baited multiple funnel FIT, 1: 16.i.2004, sulcatol+α-pinene-baited multiple funnel FIT, 1: 22.ix.2000, baited FIT; 1: Candido de Abreu Klabin S.A., Guarda Florestal Perau, 24°29'S, 51°16'W, 10.xi.2003, ethanol+α-pinene-baited multiple funnel FIT, *Pinus taeda* stand, C. Flechtmann (UNESP, CHND, FMNH, MSCC, AKTC).

###### Diagnostic description.

**Figure 65.**
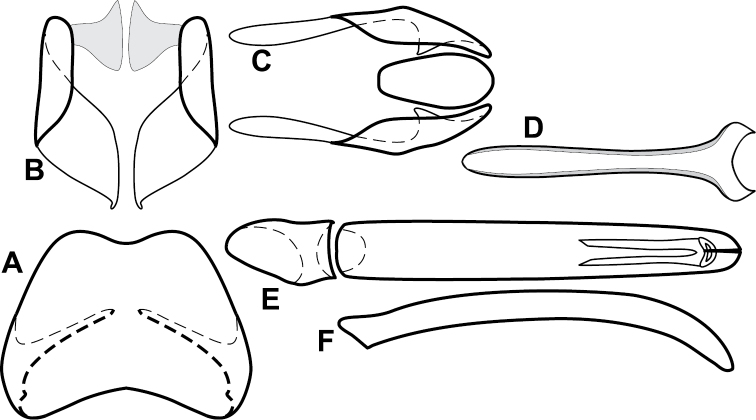
Male genitalia of *Baconia famelica*. **A** T8 **B** S8 **C** T9 & T10 **D** S9 **E** Aedeagus, dorsal view **F** Aedeagus, lateral view.

Length: 2.3-2.5mm, width: 1.6-2.0mm; body elongate, subparallel-sided, moderately depressed; head, pronotum and pygidia greenish blue, elytra distinctly more bluish, venter rufobrunneus; frons weakly carinate above antennal bases, shallowly depressed at middle, punctation of frontal disk moderately coarse, frontal stria present along eyes, curving inward at sides, weakly indicated in middle by faintly serial punctures, supraorbital stria absent; epistoma with apical margin convex, weakly elevated, depressed at middle; labrum about 4×wider than long, apical margin emarginate; each mandible with elongate apex and strong, acute median tooth; antennal scape rather short, apex obliquely truncate, club small, elongate oval, basal half mostly glabrous; pronotal sides weakly convergent in basal three-fourths, abruptly rounded to apices, marginal stria complete along lateral and anterior margins; lateral submarginal pronotal stria present along sides, weakly carinate, approaching marginal stria near anterior corner, pronotal disk narrowly, weakly depressed along its inner edge, depression slightly broadening in anterior corner; pronotal discal punctation fine and sparse medially, becoming rather dense at sides; elytra with epipleural striae slightly confused, two striae more or less complete, with a third variably abbreviated, outer subhumeral stria absent, inner subhumeral stria present in about basal three-fourths, 1^st^ dorsal stria complete, 2^nd^-3^rd^ striae very slightly abbreviated apically, 4^th^ stria very fine, usually fragmented in basal half, 5^th^ stria usually indicated by very short basal striole, occasionally absent, sutural stria present in apical two-thirds to nearly complete, elytral punctation fine, sparse, but conspicuous throughout, slightly denser posterad; prosternum narrow basally, weakly convex, keel narrowly and weakly emarginate at base, carinal striae closest near base, weakly divergent posterad and anterad, often slightly abbreviated anteriorly; prosternal lobe about one-half keel length, apical margin sinuate, weakly emarginate at middle, weakly deflexed, marginal stria fine, present only at middle; mesoventrite with anterior margin finely produced, marginal stria complete, broadly arcuate, diverging from margin laterally, mesometaventral stria absent; lateral metaventral stria extending posterolaterad toward middle of metacoxa, outer lateral stria short, disrupted by lateral punctures, metaventral disk with only very fine, sparse ground punctation medially; abdominal ventrite 1 with single lateral stria abbreviated apically, middle portion of disk mostly very finely, sparsely punctate, with slightly coarser punctures along posterior margin; protibia with 3 weak teeth and few weaker basal marginal denticles, outer margin very finely, weakly serrulate; mesotibia with 2 fine marginal spines; outer metatibial margin smooth; propygidium without basal stria, coarsely and uniformly punctate; propygidial gland openings very fine but evident, about one-third from basal and one-fourth from lateral margins; pygidium more finely, more or less uniformly punctate. Male genitalia (Fig. 65): T8 slightly wider than long, basal emargination shallow, sides convergent, apical emargination shallow, ventrolateral apodemes well sclerotized, nearly meeting at midline beneath; S8 halves approximate in basal half, narrowing apically, apical guides similar in width along apical half, apices bluntly rounded, apical velum with weakly sclerotized subtriangular plates; T9 with long, thick proximal apodemes about one-half entire length, apices narrowly rounded, convergent, ventrolateral apodemes subacute beneath; T10 elongate; S9 with stem weakly widened near base, apical arms divergent, apical emargination shallow; tegmen sides subparallel in basal two-thirds, tegmen in lateral view weakly curving ventrad over most of length; median lobe about one-third tegmen length; basal piece short, about one-fourth tegmen length.

###### Remarks.

This species and following two are very similar, and rather distinctive in this species group by being relatively impunctate and ‘normal’ looking (the subsequent species are more obviously autaomorphic in general body form). *Baconia famelica* differs in its relatively uniformly punctate frons (Fig. 64B) and epistoma (the epistoma in particular is nearly impunctate in *Baconia grossii*), lack of fine oblique rugosity on the elytral apices (finely, obliquely rugose in *Baconia redemptor*), and nearly complete inner subhumeral stria (only fine basal fragment present in *Baconia grossii*; irregularly interrupted in *Baconia redemptor*).

###### Etymology.

This species’ name means ‘hungry’, alluding to the large number of specimens attracted by bark beetle lures, undoubtedly hoping to find their prey.

##### Baconia grossii sp. n.

http://zoobank.org/07BAA5C6-0370-4B92-AEF3-33795300629B

http://species-id.net/wiki/Baconia_grossii

Fig 64D Map 20

###### Type locality.

BRAZIL: Paraná: Piraquara [25.49˚S, 48.98˚W]

###### Type material.

**Holotype female**: “BRASIL, PR, Piraquara, 15-VII-06, 1000 m P. Grossi col.” / “Coletado em masugo arboricula” / “DZUP272577” (UFPR).

###### Diagnostic description.

Length: 2.5mm, width: 1.8mm; body elongate, subparallel-sided, moderately depressed; dorsum metallic blue, pronotum faintly more greenish-blue than elytra, venter rufobrunneus with faint metallic sheen; much of dorsum very finely, inconspicuously pubescent; frons weakly carinate above antennal bases, shallowly depressed at middle, punctation of frontal disk rather sparse, slightly denser in dorsolateral corners, frontal stria present along eyes, more or less obsolete in middle, but indicated faintly by serial punctures; epistoma more or less uniformly, weakly convex; labrum about 4×wider than long, apical margin weakly emarginate; each mandible with strong, acute median tooth; antennal scape rather short, apex expanded, obliquely truncate, club small, elongate oval, basal half nearly glabrous; pronotal sides subparallel in basal half, increasingly rounded to apices, marginal stria complete along lateral and anterior margins; lateral submarginal pronotal stria present along sides, weakly carinate, approaching marginal stria near anterior corner, pronotal disk narrowly, weakly depressed along its inner edge, depression slightly broadening in anterior corner; pronotal discal punctation fine and sparse medially, only very slightly denser to sides, particularly in posterior corners; elytra with two more or less complete epipleural striae, with a third variably abbreviated, outer subhumeral stria absent, inner subhumeral stria present as very short basal fragment, 1^st^-3^rd^ dorsal striae nearly complete, subequal, slightly abbreviated apically, 4^th^ stria weak, fragmented, present in about basal two-thirds, connected by fine basal arch to base of 5^th^ stria, which is similarly fine, indicated by only few fragments, sutural stria present only in middle third, elytral punctation fine, sparse, but present throughout, slightly denser posterad; prosternum narrow basally, weakly convex, keel narrowly and weakly emarginate at base, carinal striae united along basal margin, subparallel in basal third, weakly divergent anterad, more or less complete; prosternal lobe about two-thirds keel length, apical margin bluntly rounded, weakly deflexed, marginal stria fine, complete to sides; mesoventrite with anterior margin weakly produced, marginal stria complete, mesometaventral stria absent; lateral metaventral stria extending posterolaterad toward middle of metacoxa, outer lateral stria close to inner, almost two-thirds its length, disrupted by lateral punctures, metaventral disk with only very fine, sparse ground punctation medially; abdominal ventrite 1 with single lateral stria abbreviated apically, middle portion of disk mostly very finely sparsely punctate, with slightly coarser punctures along posterior margin; protibia with 3 weak marginal teeth and few weaker basal marginal denticles, outer margin very finely serrulate; mesotibia with 2 marginal spines; outer metatibial margin smooth; propygidium without basal stria, with coarse, shallow punctures uniformly separated by about their diameters, punctures microsculptured within; propygidial gland openings fine but evident, about one-third from basal and lateral margins, surrounding disk impunctate; pygidium more finely punctate, coarse punctures mostly confined to basal half. Male: not known.

###### Remarks.

Among the species with the frons variably transversely carinate, the mandibles strongly dentate, and the antennal club at least somewhat glabrous basally, this species may be distinguished by the faint metallic coloration of the venter and the basally arched 4^th^ dorsal elytral stria (Fig. 64D), in addition to characters distinguishing it from *Baconia famelica*, above.

The labels indicate that the sole specimen of this species was collected in arboreal moss, a unique record for the genus, but perhaps a worthwhile place to search for species otherwise collected only in flight traps.

###### Etymology.

We name this species for our friend Paschoal Grossi, collector of the unique type of this species, and provider of many other interesting specimens over the course of our studies.

##### Baconia redemptor sp. n.

http://zoobank.org/BD03D759-9809-4067-8D22-675D5BED795C

http://species-id.net/wiki/Baconia_redemptor

Figs 64E–F Map 20

###### Type locality.

BRAZIL: Rio de Janeiro [22.9°S, 43.2°W].

###### Type material.

**Holotype female**: “Rio Janeiro” / “G. Lewis Coll. B.M. 1926-369” (BMNH).

###### Diagnostic description.

Length: 2.2mm, width: 1.7mm; body elongate, subparallel-sided, moderately depressed, most surfaces finely but conspicuously punctate; dorsum dully metallic, head, pronotum and pygidia greenish blue, elytra distinctly more bluish, venter rufobrunneus; frons weakly carinate above antennal bases, very shallowly depressed at middle, punctation of frontal disk coarse, rather dense, frontal stria present along eyes, curving inward at sides, broadly obsolete in middle, supraorbital stria absent; epistoma with apical margin convex, elevated above labrum; labrum about 4×wider than long, apical margin weakly emarginate; mandibles with elongate apices and strong, acute median teeth; antennal scape narrowly subtriangular, club elongate oval, basal two-thirds mostly glabrous; pronotal sides subparallel in basal three-fourths, abruptly rounded to apices, marginal stria complete along lateral and anterior margins; lateral submarginal pronotal stria present along sides, carinate, approaching marginal stria near anterior corner, pronotal disk depressed along its inner edge; pronotal discal punctation conspicuous throughout, slightly denser at sides; elytra with one complete epipleural stria, outer subhumeral stria absent, homologies of other striae slightly unclear, all striae disrupted by punctation, especially apically, and fragmented, probable inner subhumeral stria close to 1^st^ dorsal, sinuous, nearly complete, dorsal striae 1-2 complete, 3^rd^ stria abbreviated in apical fourth, 4^th^ and 5^th^ striae absent, sutural stria present in apical two-thirds, elytral punctation becoming subrugose in posterolateral corners; prosternum narrow basally, weakly convex, keel narrowly and weakly emarginate at base, carinal striae united along basal margin, subparallel in basal third, diverging, becoming obsolete anterad; prosternal lobe about one-half keel length, apical margin truncate, strongly deflexed, marginal stria well-impressed at middle, obsolete to sides; mesoventrite with anterior margin finely produced, marginal stria complete, mesometaventral stria absent; lateral metaventral stria curving posterolaterad toward middle of metacoxa, outer lateral stria absent, metaventral disk with only relatively sparse ground punctation medially; abdominal ventrite 1 with single complete lateral stria, middle portion of disk with slightly coarser punctures nearer posterior margin; all tibiae slightly broadened and flattened; protibia with 4 weak teeth and few weaker basal marginal denticles, outer margin not serrulate; mesotibia with 3 weak marginal spines; outer metatibial margin smooth; propygidium without basal stria, coarsely and uniformly punctate; propygidial gland openings very fine but evident, about one-fourth from basal and lateral margins; pygidium finely, more or less uniformly punctate, more densely near basal margin. Male: not known.

###### Remarks.

The faint metallic coloration (Fig. 64E), the frons with weak, incomplete transverse carina (Fig. 64F), conspicuous ground punctation, and the elytra with slightly irregular striae and fine, oblique apical rugosity will separate this species from all other *Baconia*.

###### Etymology.

This species’ name alludes to the famous Cristo Redentor (Christ the Redeemer) statue which now stands near its type locality.

##### Baconia fortis sp. n.

http://zoobank.org/74609543-DBE3-463E-AAA2-8DAF460CBA52

http://species-id.net/wiki/Baconia_fortis

Figs 64G–H Map 20

###### Type locality.

BRAZIL: Santa Catarina: Porto União [26.2810°S, 51.0276°W].

###### Type material.

**Holotype female**: “BR-SC-Porto União, Swedish Match 3-methyl-1-butanol baited flight intercept trap, *Populus deltoides* stand,Flechtmann, C.A.H. col., 03/XI/1998” / “PRED8” / “Caterino/Tishechkin Exosternini Voucher EXO-00750” (UNESP).

###### Diagnostic description.

Length: 2.8mm, width: 2.1mm; body narrowly elongate oval, parallel-sided, moderately depressed, most surfaces densely punctate and bearing extremely short, fine pubescence; rufopiceous throughout; frons with fine anterior transverse carina, depressed above and on epistoma below, punctation of frontal disk very dense, frontal stria apparent along eyes, obscured by punctures across middle; labrum about 4×wider than long, apical margin distinctly emarginate; mandibles with elongate apices and strong, acute median teeth; antennal scape rather elongate, curved, club small, basal two-thirds mostly glabrous; pronotal sides weakly arcuate in basal three-fourths, abruptly narrowed to apices, marginal stria complete along lateral and anterior margins, somewhat obscured by dense punctation; lateral submarginal pronotal stria present along sides, carinate, merging with marginal stria near anterior corner, pronotal disk depressed along submarginal stria, slightly more broadly depressed behind anterior corners; pronotal discal punctation dense throughout, becoming contiguously rugose at sides; elytra with two complete epipleural striae, outer and inner subhumeral striae nearly complete, dorsal striae 1–2 complete, 3^rd^ and 4^th^ striae present in basal half only, 5^th^ stria barely represented by fine basal fragment, sutural stria complete, fragmented to apex, elytral punctation becoming subrugose in posterolateral corners; prosternum narrow basally, weakly convex, keel truncate at base, carinal striae united along basal margin, subparallel in basal third, diverging anterad; prosternal lobe about one-half keel length, apical margin truncate to weakly emarginate, strongly deflexed, marginal stria obscured by punctures; mesoventrite with anterior margin truncate, marginal stria complete, mesometaventral stria present at sides, but broadly obsolete across middle; lateral metaventral stria extending posterolaterad toward middle of metacoxa, outer lateral stria absent, metaventral disk with only relatively sparse ground punctation medially; abdominal ventrite 1 with single complete lateral stria, middle portion of disk punctate as metaventrite, and with coarser punctures along posterior margin; protibia with 3 prominent teeth, and few weaker basal denticles, outer margin uneven but not distinctly serrulate; mesotibia with four marginal spines; outer metatibial margin smooth; propygidium without basal stria, propygidium and pygidium with dense intermingled ground and secondary punctation; propygidial gland openings conspicuous, very close to basal corners. Male: not known.

###### Remarks.

This species is unmistakeable in its elongate body form, non-metallic coloration, and unbiquitous dense punctation (Fig. 64G). It appears to be related to *Baconia katieae* (described below) sharing the finely carinate frontal ridge (Fig. 64H), very fine pubescence, general body form, strong mandibular teeth, and carinate lateral submarginal pronotal stria.

###### Etymology.

This species name means ‘strong’ or ‘robust’, and refers primarily to its powerful mandibles.

##### Baconia longipes sp. n.

http://zoobank.org/804D0376-7A8F-4511-B0C8-5549D856AE5E

http://species-id.net/wiki/Baconia_longipes

Figs 66 67 Map 20

###### Type locality.

BRAZIL: Amazonas: Rio Tarumã Mirim [3.03°S, 60.28°W].

###### Type material.

**Holotype male**: “BRASIL: Amazonas, Rio Tarumã Mirim, 2km from Rio Negro, 3°02'S-060°17'W, 27July1979 – Igapo” / “Black water innundation forest canopy fogged with Pyrethrum” /”CANOPY FOGGING PROJECT TRS#01 Tray #041, Adis,Erwin,Montgomery et.al. collectors” / “Histeridae sp.#2” / “Caterino/Tishechkin Exosternini Voucher EXO-00478” (USNM).

###### Diagnostic description.

Length: 2.7mm, width: 2.2mm; body elongate, weakly subquadrate, elytral sides weakly rounded, pronotum narrower, sides convergent, moderately depressed, most surfaces conspicuously, but not too deeply or densely punctate, with very short, inconspicuous, fine pubescence on at least pygidia and pronotum (probably more conspicuous on most of dorsum in fresh specimens); head, pronotum, and pygidia rufopiceous, shining, with very faint metallic tinge, elytra dully metallic blue, venter rufobrunneus; frons with fine anterior transverse carina, deeply depressed above and on epistoma below; frons with distinct alutaceous microsculpture, otherwise densely but very shallowly punctate, frontal stria apparent along eyes, obscured by punctures across middle; labrum about 4×wider than long, apical margin distinctly emarginate; each mandible with strong, acute median tooth; antennal scape rather elongate, curved, expanded apically, truncate, club small, oval, basal two-thirds mostly glabrous; pronotal sides more or less straight, weakly convergent in basal three-fourths, subangulate to apices, marginal stria complete along lateral and anterior margins, somewhat obscured by dense punctation; lateral submarginal pronotal stria present along sides, deeply impressed, merging with marginal stria near anterior corner, marginal bead narrowly, strongly convex, punctatorugose; pronotal discal punctation dense throughout; elytra with single complete, broadly impressed epipleural stria, outer subhumeral stria present in basal half, inner subhumeral stria present in basal two-thirds, dorsal striae 1–3 complete, 4^th^ and 5^th^ striae barely indicated by basal strioles, sutural stria weakly impressed but present in most of apical three-fourths, elytral punctation dense throughout, becoming weakly subrugose near apices of 1^st^–3^rd^ dorsal striae; prosternum narrow basally, flat, keel narrowly, weakly emarginate at base, carinal striae subparallel in basal third, weakly diverging anterad; prosternal lobe about one-half keel length, apical margin distinctly emarginate, strongly deflexed, marginal stria obsolete; mesoventrite with anterior margin very weakly produced, marginal stria complete, mesometaventral stria weak, present at sides, interrupted medially; lateral metaventral stria extending posterolaterad toward middle of metacoxa, outer lateral stria absent, metaventral disk sparsely, finely punctate medially; abdominal ventrite 1 with single lateral stria abbreviated apically, middle portion of disk punctate as metaventrite; all tibiae rather elongate, narrow, protibia bluntly, weakly tridentate, outer margin very weakly serrulate; mesotibia with two weak marginal spines; outer metatibial margin with single fine, subapical marginal spine; propygidium without basal stria, densely punctate, individual punctures microsculptured within; propygidial gland openings conspicuous, very close to basal corners; pygidial punctation similar to that of propygidium, punctures somewhat finer around all edges, larger in middle of disk. Male genitalia (Fig. 67): T8 slightly wider than long, basal emargination shallow, sides rounded, converging apically, apical emargination narrow, ventrolateral apodemes well sclerotized, nearly meeting at ventral midline; S8 halves rather narrow, approximate at bases, diverging and curving upward apically, apices bluntly rounded; T9 with long, thick proximal apodemes about one-half entire length, apices narrowly rounded, convergent, ventrolateral apodemes short, recurving proximad, subacute; T10 very weakly sclerotized; S9 stem widely rounded toward base, apical arms divergent, obliquely subquadrate, apical emargination shallow, desclerotized along midline; tegmen narrow, long, sides subparallel more or less throughout, weakly curved ventrad in apical third; median lobe about one-third tegmen length; basal piece about one-third tegmen length.

**Figure 66.**
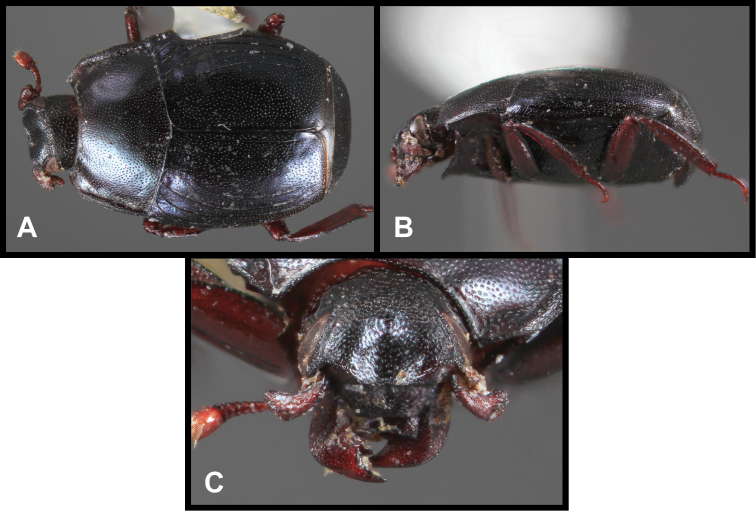
*Baconia longipes*. **A** Dorsal habitus of *Baconia longipes* **B** Lateral habitus of *Baconia longipes* **C** Frons of *Baconia longipes*.

**Figure 67.**
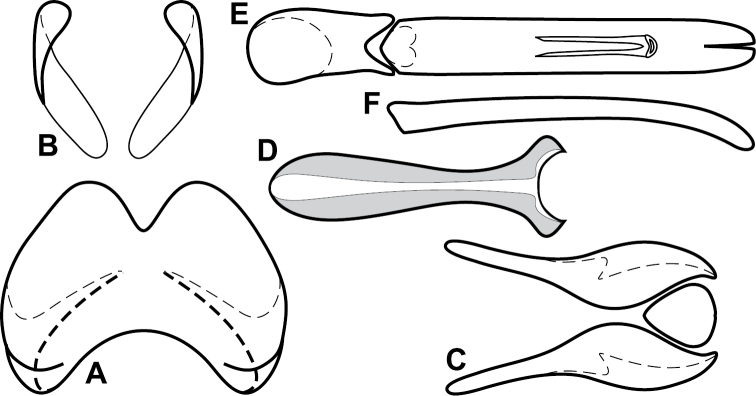
Male genitalia of *Baconia longipes*. **A** T8 **B** S8 **C** T9 & T10 **D** S9 **E** Aedeagus, dorsal view **F** Aedeagus, lateral view.

###### Remarks.

This species is very distinctive, with metallic coloration only on the elytra (Fig. 66A), conspicuous punctation throughout, its transverse frontal carina dividing a deeply depressed frons and epistoma (Fig. 66C), the deeply impressed lateral submarginal pronotal stria, and elongate legs (Fig. 66B).

###### Etymology.

This species’ name refers to its elongate femora and tibiae.

##### Baconia katieae sp. n.

http://zoobank.org/F6D97217-2D5F-4798-9D11-FB50F3F62B51

http://species-id.net/wiki/Baconia_katieae

Fig 68 Map 20

###### Type locality.

FRENCH GUIANA: Belvèdére de Saül [3.01°N, 53.21°W].

###### Type material.

**Holotype female**: “**GUYANE FRANÇAISE**: Bélvédère de Saül, point de vue. 3°1'22"N, 53°12'34"W, Polytrap 2, 30.xi.2010. SEAG leg.” / “Caterino/Tishechkin Exosternini Voucher EXO-01291” (MNHN).

###### Diagnostic description.

Length: 2.9mm, width: 2.3mm; body elongate oval, almost parallel-sided, subdepressed; head, pronotum, and pygidia metallic greenish-blue, elytra metallic violet-blue along sutural and apical margins, but with large, rufescent maculae on most of basal two-thirds of each elytron, venter bronzy-piceous, faintly metallic; head and pronotum finely but rather conspicuously pubescent; frons with prominent anterolateral carinae over antennal bases, weakened toward middle, frontal stria present only at sides, frontal disk broadly, shallowly depressed, appearing matte, with ubiquitous dense reticulate microsculpture, ground punctation conspicuous and dense, but lacking coarser secondary punctures; supraorbital stria absent; epistoma strongly transversely produced along apical margin, labrum about 3×wider than long, weakly emarginate apically; both mandibles with strong, acute basal tooth; distal palpomeres, particularly those of maxilla, short and wide; antennal scape short, obliquely truncate apically, club rather small, elongate oval, pubescence more or less restricted to apical half; pronotal sides weakly convergent in basal three-fourths, abruptly narrowed to apices, marginal stria present along basal two-thirds of lateral margin, displaced anteriorly by complete submarginal stria which is finely carinate, close to marginal, continuing around anterior margin; pronotal disk rather broadly depressed in anterior corners; ground punctation of pronotal disk conspicuous, evenly distributed, lacking coarse secondary punctures; elytra with two complete epipleural striae, outer subhumeral stria absent, inner subhumeral stria present in basal fifth and as few apical fragments, 1^st^ dorsal stria more or less complete, 2^nd^ stria present in basal two-thirds, 3^rd^ stria obsolete in apical half and basal fourth, 4^th^ stria represented by very short basal fragment, 5^th^ stria absent, sutural stria present in most of apical two-thirds, but slightly abbreviated apically, elytral disk with coarse punctures in apical third; prosternum moderately broad, weakly convex, keel emarginate at base, carinal striae subparallel, slightly abbreviated anteriorly; prosternal lobe about two-thirds keel length, apical margin subtruncate, deflexed, marginal stria fine, complete; mesoventrite produced at middle, marginal stria narrowly interrupted, mesometaventral stria weakly arched forward, crenulate, fragmented at middle, meeting lateral metaventral stria at sides, extending posterolaterad toward middle of metacoxa, outer lateral metaventral stria absent, metaventral disk impunctate at middle; abdominal ventrite 1 with single lateral stria curving mediad apically, middle portion of disk impunctate; protibia 4–5 dentate, with basal denticles weak, outer margin very finely serrulate between; mesotibia with two marginal spines; outer metatibial margin smooth; propygidium without basal stria, ground punctation conspicuous and dense, especially along basal and lateral margins, discal punctures slightly larger, removed from margins, separated by their diameters or slightly more; propygidial gland openings conspicuous, present one-fourth from basal margin and almost one-third mediad lateral corners; pygidium weakly depressed along apicolateral margins, with ground punctation conspicuous, rather dense, without coarser secondary punctures. Male: not known.

**Figure 68.**
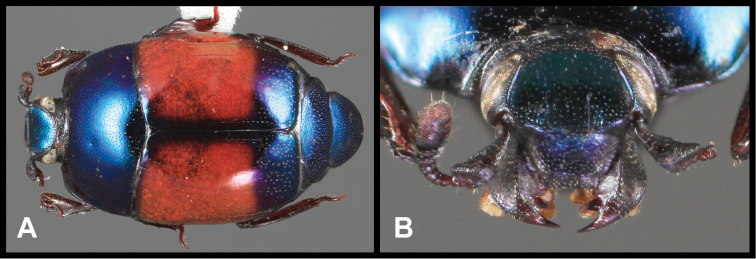
*Baconia katieae*. **A** Dorsal habitus **B** Frons.

###### Remarks.

This species is immediately identifiable by the unique color pattern (Fig. 68A), with large red maculae on the elytra and the rest of the dorsum brilliant metallic violet-blue. It has a number of other distinctive characters as well, including fine pubescence on the frons, the oblique frontal carinae over the antennal bases (Fig. 68B), the apically, transversely produced epistoma, and the apically obsolete elytral striae.

###### Etymology.

This lovely species is named for the senior author’s wife, in grateful recognition of her companionship and support.

##### Baconia cavifrons

(Lewis, 1893) comb. n.

http://species-id.net/wiki/Baconia_cavifrons

**Figure 69.**
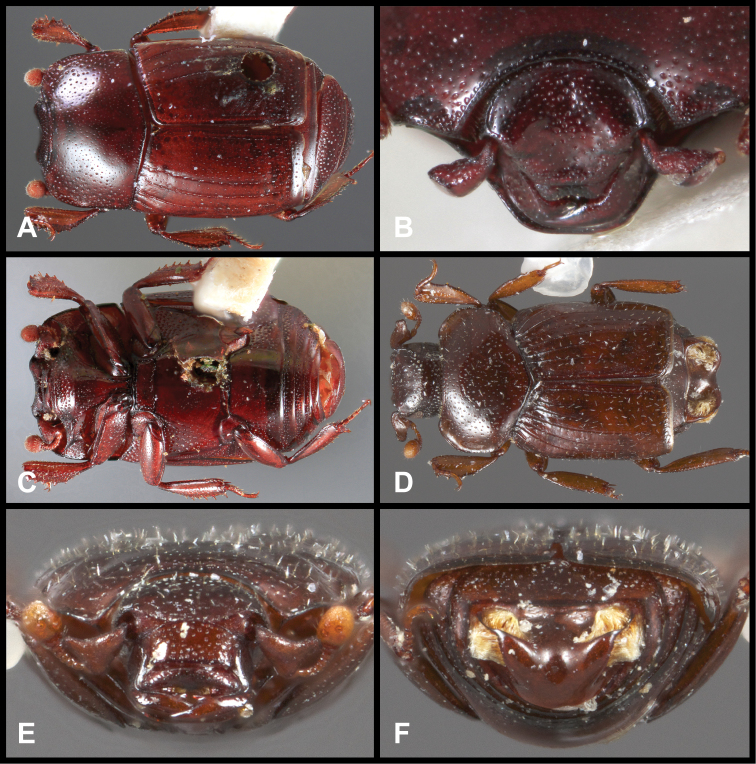
*Baconia famelica* group. **A** Dorsal habitus of *Baconia cavifrons* **B** Frons of *Baconia cavifrons* **C** Ventral habitus of *Baconia cavifrons* **D** Dorsal habitus of *Baconia haeterioides* **E** Frons of *Baconia haeterioides* **F** Pygidia of *Baconia haeterioides*.

Figs 69A–C 70A–C, G–H, K Map 20

Homalopygus cavifrons Lewis, 1893: 421.

###### Type locality.

BRAZIL: Rio de Janeiro: [22.9°S, 43.2°W].

###### Type material.

**Lectotype**, sex undetermined, here designated (BMNH): “Fry, Rio Jan^o^.” / “*Homalopygus cavifrons* Lewis Type” / “G.Lewis Coll. B.M.1926-369” / “LECTOTYPE *Homalopygus cavifrons* Lewis, 1893 M.S.Caterino & A.K.Tishechkin des. 2010”. **Paralectotypes** (3): same data as type (BMNH, FMNH). This species was explicitly described from ‘several specimens’, and the lectotype designation fixes primary type status on one of the original specimens.

###### Diagnostic description.

**Figure 70.**
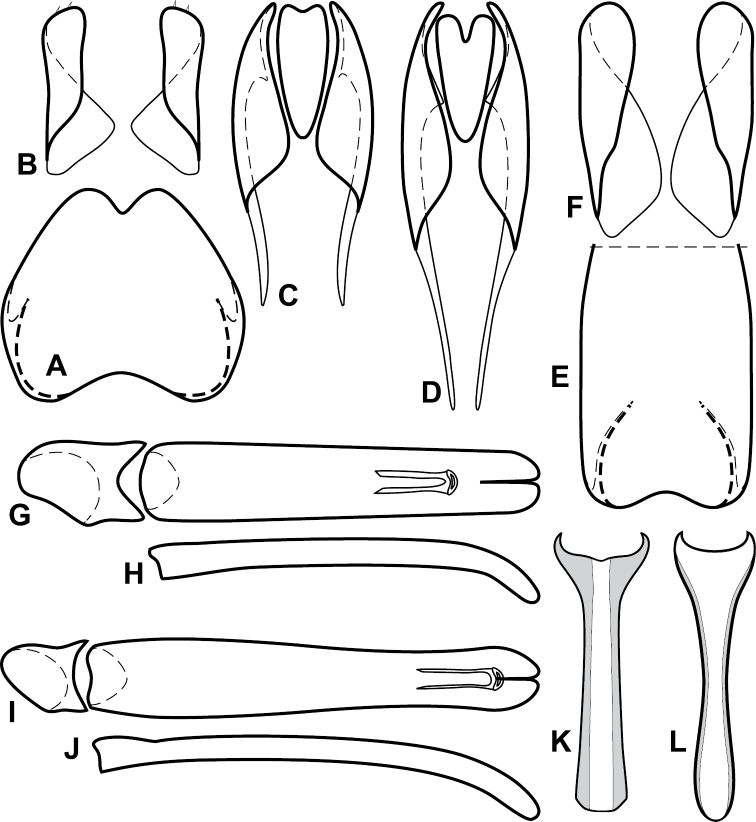
Male genitalia of *Baconia famelica* group. **A** T8 of *Baconia cavifrons* **B** S8 of *Baconia cavifrons* **C** T9 & T10 of *Baconia cavifrons* **D** T9 & T10 of *Baconia haeterioides* **E** T8 (apex damaged, not drawn) of *Baconia haeterioides* **F** S8 of *Baconia haeterioides* **G** Aedeagus, dorsal view of *Baconia cavifrons* **H** Aedeagus, lateral view of *Baconia cavifrons* **I **Aedeagus, dorsal view of *Baconia haeterioides* **J** Aedeagus, lateral view of *Baconia haeterioides* **K** S9 of *Baconia cavifrons* **L **S9 of *Baconia haeterioides*.

Length: 2.4–2.5mm, width: 1.7–1.8mm; body narrowly elongate, parallel-sided, moderately depressed, ground punctation rather conspicuous throughout; rufobrunneus; frons elevated over antennal bases, ridges more or less continued anterad onto epistoma and meeting across a distinct apical epistomal ridge, frons deeply depressed above this ridge, punctation of frontal disk coarse along raised ridges, finer between, with few deeper punctures in median area; frontal stria present along eyes, absent across middle; labrum large, about 4×wider than long, apical margin distinctly emarginate; mandibles with elongate apices and strong, acute median teeth; antennal scape short, obliquely truncate apically, club small, subspherical, completely tomentose; pronotal sides parallel in basal four-fifths, abruptly narrowed to apices, marginal stria complete along lateral and anterior margins; lateral submarginal pronotal stria absent, pronotal disk not depressed in anterior corners; pronotal ground punctation conspicuous throughout, with secondary punctures becoming larger and denser laterally and, to a lesser degree, anteriorly, relatively impunctate basomedially; elytra with two complete epipleural striae, outer subhumeral stria absent, inner subhumeral stria present as very short basal fragment, dorsal striae 1–3 complete, 4^th^ and 5^th^ striae largely absent, but a short basal arch present probably representing 5^th^ dorsal stria, sutural stria present in apical two-thirds, elytral disk with scattered secondary punctures in apical fourth and extending further forward between 3^rd^ and sutural striae; prosternum narrow basally, weakly convex, keel very weakly emarginate at base, carinal striae approximate basally, diverging anterad; prosternal lobe about one-half keel length, apical margin bluntly rounded, deflexed, marginal stria present at middle, obscured by punctures at sides; mesoventrite quadrate, weakly produced at middle, marginal stria complete, mesometaventral stria weakly arched forward, crenulate; lateral metaventral stria extending obliquely posterolaterad toward middle of metacoxa, obsolete in apical half, outer lateral stria nearly as long, metaventral disk impunctate at middle; abdominal ventrite 1 with complete inner lateral stria and short fragments of outer stria, middle portion of disk impunctate; protibia with outer margin arcuate, bearing 7–8 distinct teeth, outer margin not distinctly serrulate between; meso- and metatibiae (and femora) rather elongate, mesotibia parallel-sided with series of about 7 marginal spines; outer metatibial margin with only 2–3 marginal spines; propygidium without basal stria, discal punctures rather large, ocellate, densest along basal margin, sparser at middle; propygidial gland openings conspicuous, about one-third from anterior and lateral margins; pygidium with secondary punctures moderately large, separated by their diameters in basal two-thirds, abruptly sparser in apical third. Male genitalia (Figs 70A–C, G–H, K): T8 slightly wider than long, basal emargination shallow, sides rounded, converging apically, apical emargination narrow, ventrolateral apodemes well sclerotized, only weakly projecting beneath; S8 short, halves rather narrow, approximate at bases, inner margins diverging apically, sides subparallel, apical guides well developed, apices bluntly rounded, bearing a few inconspicuous setae; T9 with proximal apodemes about one-third entire length, apices narrowly rounded, convergent, ventrolateral apodemes short, barely recurved proximad; T10 elongate; S9 stem desclerotized along midline, weakly widened toward subtruncate base, apical arms divergent, curving distad, apical emargination broad, shallow; tegmen narrow, long, sides only very weakly narrowed near apex, weakly curved ventrad in apical third; median lobe about one-fourth tegmen length; basal piece about one-fourth tegmen length.

###### Remarks.

This species may be most easily distinguished by its elongate, subdepressed body form (Fig. 69A), in combination with the deeply depressed frons and rather long, relatively spinose legs (Fig. 69B, C). Its original description in *Homalopygus* is difficult to understand, as its similarity to members of that Haeteriine genus seems obviously superficial. However, it is certainly not very similar to any other *Baconia* species, so its persistent assignment to *Homalopygus* may simply be due to difficulty in determining a better placement. Its antennal club’s sensory foveae make its assignment to *Baconia* unambiguous, despite its numerous unusual characters.

##### Baconia haeterioides sp. n.

http://zoobank.org/1ED3207D-84E8-407C-9BAD-5631FECCB0D9

http://species-id.net/wiki/Baconia_haeterioides

Figs 69D–F 70D–F, I–J, L Map 20

###### Type locality.

BRAZIL: Minas Gerais: Ingaí Municipality [21.3°S, 44.9°W].

###### Type material.

**Holotype male**: “**BRASIL: Minas Gerais**, Ingaí Municip., Boqueirão, Res. Nr. Lavras. Rocky field flight intercept trap December 2002. R.J.Silva” / “LSAM0047469” (CEMT). **Paratype** (1): **BRAZIL**: **Goias**: Paraiso, 8–14.ii.1962, J. Běchyně (CHND).

###### Diagnostic description.

Length: 1.8–2.0mm, width: 1.2–1.3mm; body elongate, subparallel-sided, constricted at humeri, rather strongly depressed, most dorsal surfaces distinctly, sparsely punctate, bearing short, narrowly acute scale-like setae; rufobrunneus throughout; frons transversely carinate between antennal bases, flattened above, epistoma very broad, flat to weakly concave below, delimited above by distinct, complete frontoclypeal suture; frontal disk sparsely punctate, frontal stria only vaguely indicated by punctures along inner edge of eyes; labrum extremely wide, about 8×wider than median length, extending laterad beyond sides of epistoma, broadly and deeply arcuate; mandibles strongly bent at anterolateral corners, inner apices long, both with strong, acute median tooth; antennal scape strongly expanded apically, irregularly triangular, club small, elongate oval, basal two-thirds glabrous and shining, with sensory openings distinct; pronotal sides strongly, unevenly rounded, narrowed basally, with small, rather inconspicuous trichome on opposing surfaces of hypomeron and mesepisternum, barely visible from above; pronotal sides depressed to explanate; marginal pronotal stria obsolete in basal half, otherwise more or less continuous along lateral and anterior margins, lateral submarginal stria absent; pronotal discal punctation sparse throughout, dual, with larger punctures setigerous; elytral sides subparallel but strongly narrowed to humeri, shallowly depressed along basal margin, with one complete epipleural stria, outer subhumeral stria very briefly indicated at base, inner subhumeral stria present in basal half to two-thirds, dorsal striae 1–2 more or less complete, 3^rd^–5^th^ striae progressively abbreviated from apex, sutural stria longer, more or less complete, setigerous punctures of elytra smaller and more uniform than those of pronotum; prosternum narrow basally, weakly convex, keel truncate at base, carinal striae united along basal margin, subparallel in basal third, diverging anterad; prosternal lobe about one-half keel length, apical margin truncate to weakly emarginate, weakly deflexed, marginal stria fine, obsolete at sides; mesoventrite with anterior margin finely and weakly produced, marginal stria very close to margin, fragmented at middle, mesometaventral stria present as detached fragments on each side; lateral metaventral stria extending posterolaterad toward outer third of metacoxa, outer lateral stria short, vaguely indicated, metaventral disk with only conspicuous, sparse ground punctation medially; abdominal ventrite 1 with single complete lateral stria, middle portion of disk punctate as metaventrite; protibia broad, somewhat swollen, with 3-4 weak teeth, outer margin not serrulate between teeth; meso- and metatibiae weakly expanded, flattened, mesotibia with 2 very weak marginal spines; outer metatibial margin smooth; propygidium without basal stria, with only sparse setigerous punctures; pygidium strongly produced at middle, with large, expanded, setose depressions on each side, above and below, the margin between upper and lower trichomes finely carinate, glabrous, pygidial disk otherwise impunctate. Male genitalia (Figs 70D–F, I–J, L): T8 slightly elongate, basal emargination shallow, sides subparallel, ventrolateral apodemes only projecting beneath basal half, their apices separated by half T8 width; S8 halves rather narrow, approximate at bases, inner margins diverging apically, sides subparallel, apical guides well developed, apices bluntly rounded; T9 with proximal apodemes nearly half entire length, apices narrowly rounded, convergent, ventrolateral apodemes short, blunt; T10 apically emarginate; S9 stem desclerotized along midline, weakly widened toward base, apical arms divergent, curving distad, apical emargination broad, shallow; tegmen narrow, long, sides sinuate, widest near base, narrowed about one-third from apex, apex slightly widened, weakly curved ventrad in apical half; median lobe very short, about one-fifth tegmen length; basal piece about one-fifth tegmen length.

###### Remarks.

A diagnosis is almost superfluous for this remarkable species. The very conspicuous pygidial trichomes immediately distinguish it from any other Neotropical histerid (Figs 69D, F). The presence of trichomes and the unusual expansion of the antennal scapes first led us to think this might be an aberrant member of Haeteriinae. However, the distinctive sensoria of the antennal club make its inclusion in *Baconia* unambiguous, and it in fact shares a number of characters with several other species in the *Baconia famelica* group, especially the setigerous, punctate cuticle, the truncate/emarginate prosternal lobe, the basally glabrous antennal club, and the transversely carinate frontal margin (Fig. 69E). It shares its broadened tibiae with *Baconia cavifrons* (also first considered an Haeteriine), but is probably more closely related to *Baconia fortis*, *Baconia longipes*, and *Baconia redemptor*.

The trichomes of this species clearly suggest a myrmecophilous habit. Unfortunately both known specimens appear to have been collected in passive traps, and no such data exists. The ‘Paraiso’ locality in Goias is somewhat ambiguous. We suggest this is from Alto Paraiso in the northeastern part of the state.

###### Etymology.

This species’ name alludes to its similarity to some Haeteriinae, particularly in its prominent trichomes and probable inquilinous habits.

#### *Baconia micans* group

**Figure 71.**
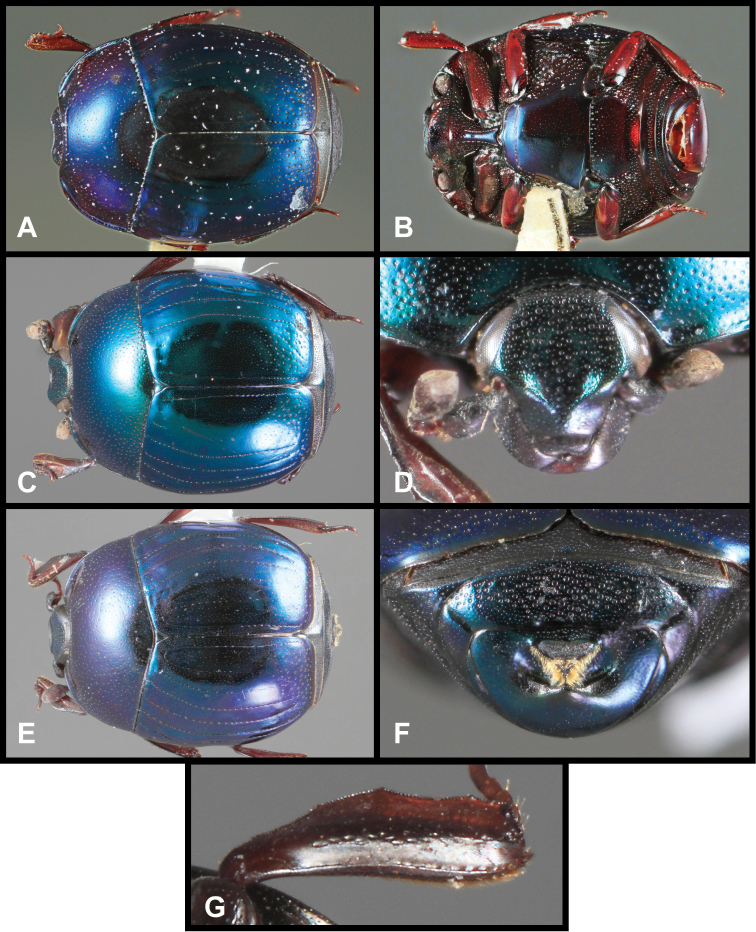
*Baconia micans* group. **A** Dorsal habitus of *Baconia micans* **B** Ventral habitus of *Baconia micans* **C** Dorsal habitus of *Baconia carinifrons* **D** Frons of *Baconia carinifrons* **E** Dorsal habitus of *Baconia fulgida* **F** Pygidia of male *Baconia fulgida* **G** Protibia of *Baconia fulgida*.

The three species of the *Baconia micans* group share several obvious characters, including a relatively large, convex body form, bright metallic blue coloration which extends at least faintly onto the venter (Fig. 71A), relatively weakly dentate protibiae (Fig. 71G), and a large and asymmetrical antennal club, widened predominantly on its inner edge. They also share an anterior pronotal stria that is detached from the marginal (Fig. 71D). Two of the species are known from both males and females, and only one of these, *Baconia fulgida*, shows a remarkable pygidial dimorphism, with the male pygidium bearing a setose median carina (Fig. 71F). This species also has highly autapomorphic male genitalia, relative to the other species in the group and to the genus as a whole. Discovery of the male of *Baconia carinifrons* would be very interesting as it relates to both of these character systems.

##### Baconia micans

(Schmidt, 1889)

http://species-id.net/wiki/Baconia_micans

**Map 21.**
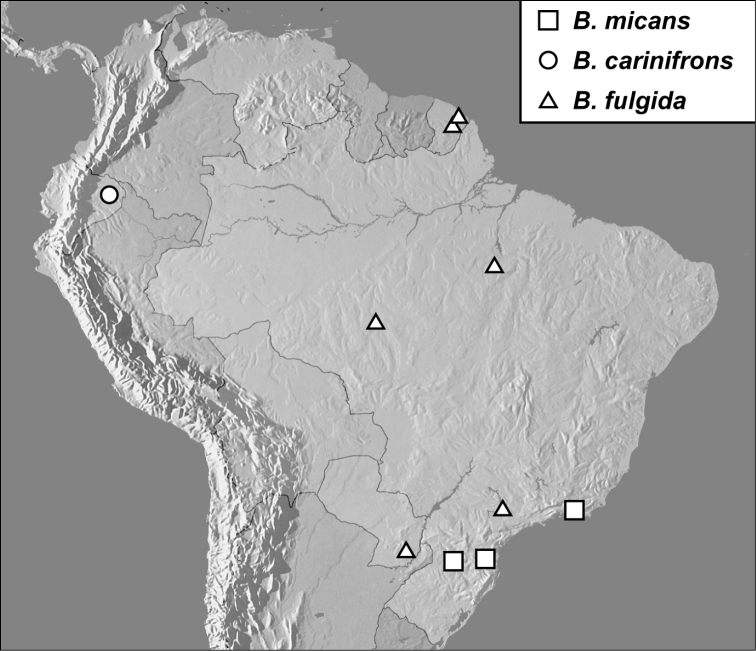
*Baconia micans* group records.

Figs 71A–B 72A–F Map 21

Phelister micans Schmidt, 1889a: 336; *Baconia micans*: Mazur 1984: 281.

###### Type locality.

BRAZIL: Santa Catarina: Blumenau [26.9°S, 49.0°W].

###### Type material.

**Lectotype male**, here designated (ZMHB): “Blumenau, Bras.” / “micans Schmidt.” / “coll. J.Schmidt” / “*Phelister micans* Schmidt, 1889 ex. Coll. Schmidt-Bickhardt” / “Caterino/Tishechkin Exosternini Voucher EXO-00419” / “LECTOTYPE *Phelister micans* Schmidt, 1889, M.S.Caterino & A.K.Tishechkin des. 2010”. This species was described from an unspecified number of specimens, and the lectotype designation fixes primary type status on the only known original specimen.

###### Other material.

**BRAZIL**: 1: **Santa Catarina**: Nova Teutonia, x.1965 F. Plaumann (FMNH); 1: **Rio de Janeiro**:Rio de Janeiro, x.[year?] (MHNG).

###### Diagnostic description.

**Figure 72.**
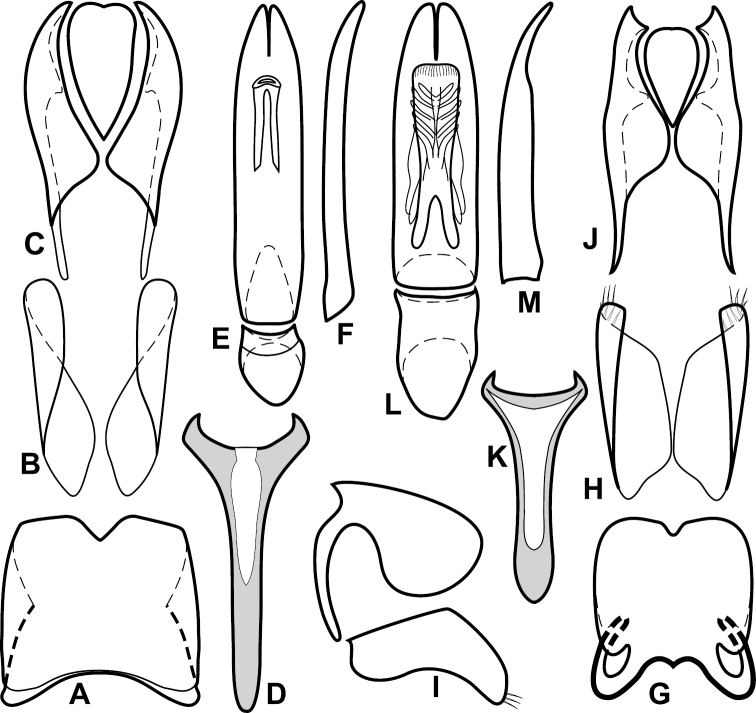
Male genitalia of *Baconia micans* group. **A–F** *Baconia micans*. **A** T8 **B** S8 **C** T9 & T10**D** S9 **E **Aedeagus, dorsal view **F** Aedeagus, lateral view **G–M** *Baconia fulgida* **G** T8 **H** S8 **I** T8 & S8, lateral view **J **T9 & T10 **K** S9 **L** Aedeagus, dorsal view **M** Aedeagus, lateral view.

Length: 2.8–2.9mm, width: 2.5–2.6mm; body elongate oval, convex, glabrous; entire body metallic blue to greenish-blue, venter more faintly so; frons weakly, obliquely elevated anterolaterally, depressed at middle, epistoma convex anteriorly, frontal disk with ground punctation rather conspicuous, with few coarser secondary punctures within frontal depression and at dorsal margin, frontal stria present along inner margin of eye, curving mediad but otherwise absent across front, supraorbital stria absent; labrum about 3×wider than long, weakly emarginate apically; both mandibles with blunt basal tooth; antennal scape short, club oblong, weakly widened to apex; pronotal sides increasingly arcuate to apex, marginal stria complete along lateral margin, anterior portion transverse, detached behind eyes; lateral submarginal pronotal stria complete, pronotal disk narrowly depressed along its inner edge, more deeply so anteriorly, ground punctation of pronotal disk rather conspicuous, sparsely interspersed with small secondary punctures almost throughout, more densely to sides; elytra with two complete epipleural striae, outer subhumeral stria absent, inner subhumeral stria present in basal two-thirds, may be weakened at middle, dorsal striae 1–3 more or less complete, 4^th^ stria variably abbreviated from apex, may be largely absent, 5^th^ stria mostly present as short basal arch, sutural stria nearly complete, but weak to obsolete basally, elytral disk with coarse punctures in apical third; prosternum narrow, convex, keel emarginate at base, carinal striae convergent, abbreviated anteriorly; prosternal lobe short, about one-half keel length, apical margin broadly rounded, marginal stria obsolete at sides; mesoventrite produced at middle, marginal stria complete, mesometaventral stria arched forward, crenulate, interrupted at middle; lateral metaventral stria extending posterolaterad toward inner third of metacoxa, outer lateral metaventral stria short, present anteriorly, metaventral disk impunctate at middle; abdominal ventrite 1 with single lateral stria abbreviated apically, middle portion of disk with small punctures along basal and apical margins; protibia very weakly 3–4 dentate, marginal spines very small, outer margin finely serrulate; mesotibia with few weak submarginal spines; outer metatibial margin smooth; propygidium without basal stria, discal punctures generally rather small, separated by about their diameters basally, sparser apically; propygidial gland openings generally evident close to basal margin; pygidium with ground punctation very fine, interspersed with small secondary punctures mainly in basal third. Male genitalia (Figs 72A–F): T8 broad, basal emargination shallow, sides weakly rounded, subparallel, apical emargination shallow, ventrolateral apodemes well sclerotized, reaching longitudinal midpoint beneath; S8 halves rather narrow, approximate near bases, inner margins diverging apically, sides subparallel, apical guides well developed toward apex, apices broadly rounded, setigerous; T9 with proximal apodemes about one-third entire length, apices narrowly rounded, convergent, ventrolateral apodemes short, barely recurved proximad; S9 stem desclerotized along midline, narrow, sides convergent to base, apical arms divergent, obliquely subquadrate, apical emargination broad, shallow; tegmen rather short, sides weakly narrowed at middle, weakly curved ventrad in apical fourth; median lobe about one-third tegmen length; basal piece about one-fourth tegmen length.

**Remarks.** Species in the *Baconia micans* group are among the few *Baconia* that are distinctly metallic-colored ventrally (Fig. 71B) as well as dorsally. In addition, the complete lateral submarginal pronotal stria, the basally arched 5^th^ dorsal stria, and generally round and convex body form (Fig. 71A) will distinguish *Baconia micans* from its close relatives. The specimen from Nova Teutonia has much sparser propygidial punctation than the lectotype or the Rio de Janeiro specimen, but is otherwise consistent in all characters.

##### Baconia carinifrons sp. n.

http://zoobank.org/5B4E07AB-05EA-45B5-AFAB-50C8F68EC831

http://species-id.net/wiki/Baconia_carinifrons

Figs 71C–D Map 21

###### Type locality.

ECUADOR: Orellana:Res. Ethnica Waorani [0.67°N, 76.43°W].

###### Type material.

**Holotype female**: “**ECUADOR: Depto. Orellana**: Res. Ethnica Waorani, 1km S Onkone Gare Camp, Trans. Ent., 0°39'26"S, 76°27'11"W, 216m, 21 June 2006, T.L. Erwin, M.C.Oimienta et al.” / “Insecticidal fogging of mostly bare green leaves, some with covering of lichenous or bryophytic plants in terra firme forest. Project MAXUS **Lot 3115 Trans. 2 Sta. 6**” / “Caterino/Tishechkin Exosternini Voucher EXO-00413” (USNM).

###### Diagnostic description.

Length: 2.9mm, width: 2.7mm; body broadly oval, convex, glabrous; head and pronotum metallic greenish-blue, elytra slightly bluer, venter rufopiceous with faint metallic blue coloration; frons with strong, oblique anterolateral carinae extending from above antennal bases onto middle of epistoma, deeply depressed between, frontal disk with dense ground punctation and numerous coarser secondary punctures nearly throughout, frontal and supraorbital striae absent; labrum about 3×wider than long, weakly emarginate apically; antennal scape short, club asymmetrically oblong; pronotal sides increasingly arcuate to apex, marginal stria complete along lateral margin, anterior portion narrowly detached behind eyes; lateral submarginal pronotal stria complete, pronotal disk narrowly depressed along its inner edge, more broadly so anteriorly, ground punctation of pronotal disk rather conspicuous, sparsely interspersed with small secondary punctures almost throughout, sparser mediad, denser to sides; elytra with two complete epipleural striae, outer subhumeral stria represented by basal and median fragments, inner subhumeral stria nearly complete, slightly abbreviated apically, dorsal striae 1-3 complete, 4^th^ stria barely visible at base, 5^th^ stria represented only by basal puncture, sutural stria complete, elytral disk with coarse punctures in apical third; prosternum narrow, convex, keel emarginate at base, carinal striae convergent, abbreviated anteriorly; prosternal lobe about two-thirds keel length, apical margin rather narrowly rounded, marginal stria fine, obsolete at sides; mesoventrite very weakly produced at middle, marginal stria complete, mesometaventral stria arched forward, crenulate, broadly interrupted at middle; lateral metaventral stria extending posterolaterad toward inner corner of metacoxa, outer lateral stria parallel to basal two-thirds of inner stria, metaventral disk impunctate at middle; abdominal ventrite 1 with single lateral stria curved mediad, abbreviated apically, middle portion of disk with few small punctures along basal margin; protibia very weakly 2-3 dentate, marginal spines very small, outer margin serrulate; mesotibia with few weak submarginal spines; outer metatibial margin smooth; propygidium without basal stria, discal punctures generally small and dense along basal margin, larger but sparser toward apical margin; propygidial gland openings small, close to basal margin, about one-fourth from lateral corner; pygidium with ground punctation very fine, denser toward apex, interspersed with small secondary punctures mainly in basal two-thirds. Male: not known.

###### Remarks.

This species is related to *Baconia micans* and *Baconia fulgida*, sharing the oblique frontal ridges, which are most strongly developed and distinctly carinate in this species (Fig. 71D). In addition to the frontal carinae, *Baconia carinifrons* can easily be distinguished by its lack of a basal arch of the 5^th^ stria (Fig. 71C), complete lack of frontal stria, very weakly metallic ventral coloration, and more conspicuous frontal punctures. It would be very desireable to find a male of this species to see if it shared the very distinctive pygidial and genitalic characters of *Baconia fulgida*.

###### Etymology.

This species is named in reference to its distinct, oblique frontal carinae.

##### Baconia fulgida

(Schimdt, 1889)

http://species-id.net/wiki/Baconia_fulgida

Figs 71E–G 72G–M Map 21

Phelister fulgidus Schmidt, 1889c: 324; *Baconia fulgida*: Mazur 1984: 280.

###### Type locality.

PARAGUAY [exact locality unknown].

###### Type material.

No type material of this species can be located. The published type locality is simply Paraguay, collected by a ‘Herr Drake’. We have no reason to believe that the type should be anywhere other than with the rest of Schmidt’s collection at ZMHB. However, several other types from Drake’s collections are also unaccounted for, and some unknown arrangement may have seen them deposited elsewhere. Due to this uncertainty we refrain from designating a neotype at present. The species is sufficiently distinctive, and a perfect match to Schmidt’s description, to preclude any confusion about its identity.

###### Other material.

**BRAZIL**: 3: **Mato Grosso**:Mpio. Cotriguaçu, Fazenda São Nicolau, Matinha, 9°50.3'S, 58°15.05'W, xii.2010, FIT, F. Vaz-de-Mello, 4: x.2009, FIT, F. Vaz-de-Mello (CEMT, MSCC, AKTC); 1: Mpio. Cotriguaçu, Fazenda São Nicolau, Prainha, 9°51.6'S, 58°12.9'W, x.2009, FIT, F. Vaz-de-Mello, 4: ix.2009, FIT, R. Nunes (FMNH); 1: **Pará**: Carajas (Serra Norte), 6°4'S, 50°12'W, x.1984, FIT (CHND); 1: **São Paulo**: Lençóis, Faz. Rio Pardo-Duratex, 22°47'33"S, 49°1'5"W, cerradão fragment, α-pinene baited FIT, 10.xi.2006, C. Flechtmann (UNESP). **FRENCH GUIANA**: 1:Rés. des Nouragues, Régina, 4°2.27'N, 52°40.35'W, 22.ix.2009, FIT, SEAG (CHND); 1:3.xi.2009, FIT, SEAG, 1:30.ix.2009, FIT, SEAG (CHND); 1:Montagne des Chevaux, 4°43'N, 52°24'W, 27.vii.2009, FIT, SEAG (CHND). **PARAGUAY**: 1: **Itapúa**: Res. San Rafael, 17 km W Karonay, 26°45'53"S, 55°50'37"W, 90–110 m, 19.xi.2000, fogging fungusy logs, Z. Falin (SEMC).

###### Diagnostic description.

Length: 2.4–3.1mm, width: 2.2–2.8mm; body elongate oval, broadly rounded, moderately depressed, glabrous; body coloration varied, dorsum metallic blue, greenish-blue or violet, head and/or pronotum may be slightly more greenish than elytra, venter rufobrunneus to rufopiceous; frons somewhat elongate, obliquely elevated from antennal bases onto epistoma, weakly depressed behind, epistoma strongly convex anterad, interocular margins strongly convergent dorsad, sides of frontal stria angulate, following oblique frontal ridges, complete or interrupted at middle and/or sides, ground punctation conspicuous, with few secondary punctures at middle and near vertex, supraorbital stria absent, but often apparently represented by dense series of punctures; antennal scape short, thick, club quite elongate; labrum about 3×wider than long, apical margin straight to weakly emarginate; both mandibles with rather weak basal tooth; pronotal sides more or less evenly arcuate to apex, weakly depressed in anterior corners, marginal stria complete along lateral margins, anterior portion generally transverse, detached from anterior portion behind eyes, lateral submarginal stria absent, ground punctation of pronotal disk fine, sparsely interspersed with small secondary punctures almost throughout, relatively impunctate posteromedially; elytra with 2–3 epipleural striae, the outermost variably complete, outer subhumeral stria absent, inner subhumeral stria more or less complete, may be slightly abbreviated apically, dorsal striae 1-3 complete, 4^th^ stria variably abbreviated from apex, may be complete, 5^th^ stria usually present only as short basal arch connected to complete sutural stria, elytral disk with few coarse punctures in apical fifth; prosternum narrow, convex between complete carinal striae, keel weakly emarginate at base; prosternal lobe about one-half keel length, apical margin rounded, marginal stria obsolete at sides; mesoventrite weakly produced at middle, marginal stria complete, mesometaventral stria weakly arched forward, crenulate, continuous at sides with lateral metaventral stria from near mesocoxa posterolaterad toward inner third of metacoxa, sinuate apically, outer lateral metaventral stria parallel to basal half of inner stria, metaventral disk impunctate at middle; abdominal ventrite 1 with single lateral stria, middle portion of disk impunctate; protibia weakly 4 dentate, marginal denticles minute, outer margin serrulate; mesotibia with few very weak submarginal spines; outer metatibial margin smooth; propygidium wide and short, with complete transverse basal stria, discal punctures rather small, ocellate, uniformly dispersed; propygidial gland openings evident behind basal stria, about one-third from anterior and lateral margins; pygidium strongly sexually dimorphic, female with fine ground punctation more or less uniformly interspersed with small secondary punctures, male pygidium dominated by elevated, pyramidal, setose median process (Fig. 71F), with deep depression on each side. Male genitalia (Figs 72G–M): T8 about as long as broad, sides subparallel, narrowing to very strong, linear ventrolateral apodemes, basal emargination bisinuate, acute medially, apical emargination shallow; S8 longer than T8, distinctly hinged to T8 at base, divided, inner margins weakly divergent toward apex, outer margins subparallel to weakly divergent, apical guides narrow, gradually widening to narrowly rounded apices, bearing few apical setae; T9 with basal apodemes moderately short, about one-third total length, T9 apices wide, obliquely emarginate, glabrous, ventrolateral apodemes moderately strongly projecting beneath; T10 entire; S9 broad, sides of stem subparallel, head abruptly widened, sides curved to apicolateral points, sclerotized along lateral, apical, and basal edges, undivided apically; tegmen broad with sides subparallel in basal three fourths, weakly narrowed to apex, dorsobasal edge straight, tegmen in lateral aspect flattening, slightly curved ventrad at apex; median lobe prominent, about two-thirds tegmen length and half tegmen width, with complex encompassing armature; basal piece about one-half tegmen length, dorsal and ventral apical emarginations shallow.

###### Remarks.

This species is unmistakeable, particularly in the male, with its unique pygidial process (Fig. 71F). The obliquely elevated frontal ridges, broadly oval body form, and basally connected 5^th^ and sutural striae (Fig. 71E) will easily identify either sex.

The so-called armature of the aedeagus is unique and difficult to resolve as to function. Near the subapical foramen of the tegmen, where the median lobe extrudes, parts of the interlocking digitiform processes of the armature do project. It appears that it would move in concert with the median lobe, and that lateral series of these processes are everted and project laterally as a novel grasping mechanism during mating.

####### *Baconia* incertae sedis

The species below are unplaced as to group. In most cases this is due to combinations of characters incompatible with any other recognized groups. However, in a few it is simply indicative of limited material and particularly unavailability of males with which to confirm genitalic characters.

##### Baconia chilense

(Redtenbacher, 1867)

http://species-id.net/wiki/Baconia_chilense

**Figure 73.**
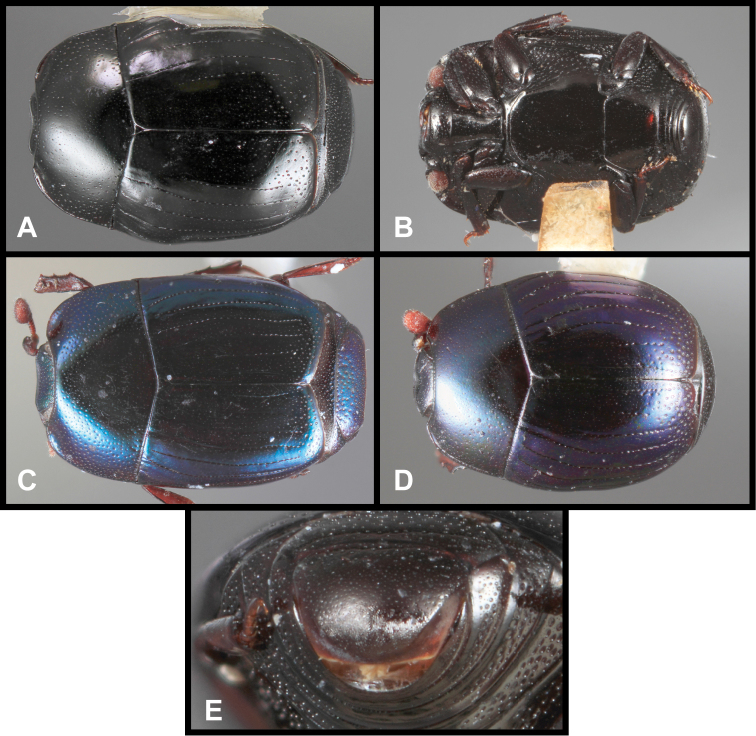
*Baconia* incertae sedis spp. **A** Dorsal habitus of *Baconia chilense* **B** Ventral habitus of *Baconia chilense* **C **Dorsal habitus of *Baconia glauca* **D** Dorsal habitus of *Baconia coerulea* **E** Pygidia of *Baconia coerulea*.

**Map 22.**
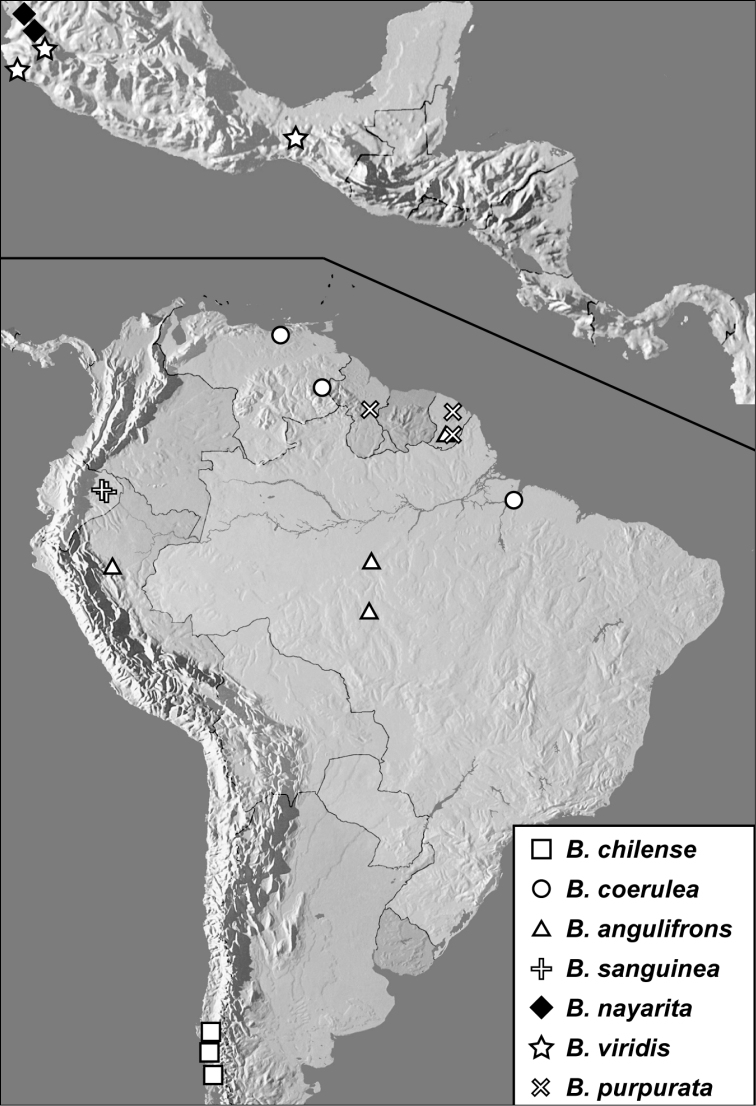
*Baconia* incertae sedis records. Record for *Baconia coerulea* in Pará, Brazil is a state record only.

Figs 73A–B 74 Map 22

Platysoma chilense Redtenbacher, 1867: 32; *Pseudister chilensis*: Johnson in Johnson et al. 1991: 12; *Baconia chilensis*: Mazur 1997: 25.
Hister impressifrons Solier, 1849: 379 (not Blanchard 1843); *Phelister impressifrons*: Marseul 1861: 159; *Phelister impressifrons*: Lewis 1905: 48; *Pseudister impressifrons*: Bickhardt 1917: 165; junior primary homonym.

###### Type locality.

CHILE [exact locality unknown]

###### Type material.

We have been unable to locate type material for either the junior or senior synonym of this species. However, because only a single *Baconia* species is known from Chile, because Marseul identified some available specimens as Solier’s species (with which he would have been familiar), and because all previous authors have agreed on its identity, we feel very confident in this species’ identity, and do not feel that the designation of a neotype is necessary.

###### Other material.

**CHILE**: 39: **Cautín**: Cherquenco, W. of Volc. Laima, 38°21'S, 72°00'W, i.1954, L. Pena (FMNH); 2: Fdo Las Selvas, N.W. Nueva Imperial W. Temuco, 600–700 m, 18.ii.1981, L. Pena (USNM, MHNG); 1: **Llanquihue**: N.W. Shore, Lago Chapo, 41°27'S, 72°30'W, 250 m, 13.xi.1966, M. Irwin & E. Schlinger (CASC), 1, 14.ii.1982, G. Arriagada (MHNG); 1: **Valdivia**: Rincon de la Piedra, turnoff 14.8 km SE Valdivia, 39°55.32'S, 73°06.27'W, 50 m, 2.ii.1997, under bark, disturbed Valdivian rainforest w/ *Nothofagus dombeyi*, *Podocarpus saligna*, *Persea lingue* log, A. Newton & M. Thayer (FMNH); 2: [country record only] (BMNH).

###### Diagnostic description.

**Figure 74.**
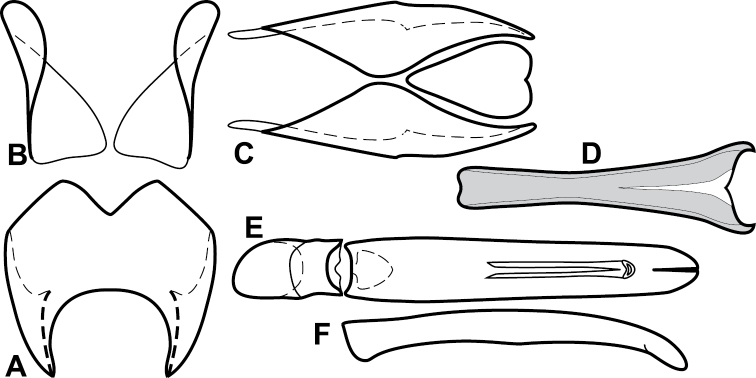
Male genitalia of *Baconia chilense*. **A** T8 **B** S8 **C** T9 & T10 **D** S9 **E** Aedeagus, dorsal view **F** Aedeagus, lateral view.

Length: 2.2–2.4mm, width: 1.8–1.9mm; body elongate oval, humeri prominent, moderately depressed, glabrous; piceous, shining, not metallic; frons elongate, weakly elevated over antennal bases, depressed along midline, interocular margins rounded, convergent dorsad, frontal stria nearly complete, narrowly interrupted or disrupted by punctures at middle, frontal disk with few coarse punctures in median depression and toward vertex, supraorbital stria absent; epistoma flat to weakly depressed in middle, apical margin straight; labrum broadly emarginate to weakly bisinuate; mandibles each with basal tooth; antennal scape short, club broadly rounded; pronotal sides weakly convergent in basal two-thirds, more strongly arcuate to apices, lateral marginal striae continuous around sides and front, submarginal stria present, merging with marginal stria about one-fourth behind anterior corner, pronotal disk with anterior corners depressed, coarsely punctate along most of posterior margin, with shallow discal punctures present in anterior half and lateral third, only fine ground punctures present at middle; elytra with two complete epipleural striae, outer subhumeral stria absent, inner subhumeral stria nearly complete, slightly abbreviated at apex, dorsal striae 1–3 complete, 4^th^ stria abbreviated from base and apex, 5^th^ and sutural striae absent, elytral disk with small, sparse punctures in apical third; prosternal keel broad, weakly convex, truncate to weakly emarginate at base, carinal striae subparallel basally, diverging anterad, complete, may be united along basal margin; prosternal lobe short, about one-half keel length, apical margin deflexed, subtruncate, marginal stria absent; mesoventrite sinuate to weakly produced at middle, marginal stria broadly interrupted; mesometaventral stria arched strongly forward displacing marginal stria, not crenulate, narrowly detached from inner lateral metaventral stria, which curves posterolaterad toward outer third of hind coxa, slightly abbreviated apically, outer lateral metaventral stria short, oblique; metaventral disk impunctate at middle; abdominal ventrite 1 with inner lateral stria complete, outer stria short, apical, median portion of disk with few punctures near apices of lateral stria; protibia 4–5 dentate, basal denticles weak, outer margin finely serrulate between spines; mesotibia with two weak marginal spines; outer metatibial margin smooth; propygidium lacking basal stria, with rather large, shallow secondary punctures separated by slightly less than their diameters in middle, smaller and sparser to sides, propygidial glands conspicuous, about midway back from anterior margin, about one-fifth from lateral corners; pygidium short, with fine ground punctation interspersed with secondary punctures, mainly in basal half. Male genitalia (Fig. 74): T8 broad, sides rounded, basal emargination deeply arcuate, apical emargination shallow, ventrolateral apodemes reaching one-third from base beneath; S8 halves approximate near bases, inner margins rapidly diverging apically, sides divergent, apical guides developed toward apex, apices broadly rounded; T9 with proximal apodemes short, dorsal lobes broad at middle apices narrowly rounded, convergent, ventrolateral apodemes very weakly developed; S9 stem desclerotized along midline, sides narrowed, one-third from base, apical arms divergent, curved, apical emargination broad, shallow; tegmen narrow, sides weakly narrowed one-third from apex, weakly curved ventrad at extreme apex; median lobe about one-half tegmen length; basal piece about one-fourth tegmen length.

###### Remarks.

While the distribution of this species alone is diagnostic, it has a number of other distinguishing characters in the genus, including the prominent, swollen humeri (Fig. 73A), the relatively broad prosternal keel (unusual among less obviously flattened species), and displaced mesoventral marginal stria (Fig. 73B). In many respects it resembles members of the *Baconia godmani* group, and it may be close to that group, although the male S8 is neither elongate nor setose.

##### Baconia glauca

(Marseul, 1884)

http://species-id.net/wiki/Baconia_glauca

Figs 73C 75 Map 15

Phelister glaucus Marseul, 1884: 162; *Pseudister glaucus*: Bickhardt 1917: 165 *Baconia glauca*: Mazur 1990: 755.

###### Type locality.

INDONESIA: Sumatra: Serdang [3.51°N, 98.81°E].

###### Type material.

**Holotype**, (MNHN): not located. This species was explicitly described from a single specimen. The published type data is eastern Sumatra, Serdang, Tandjong Morawa, collected by B. Hagen.

###### Other material.

**MALAYSIA**, 1: **Selangor**: Penchala, Kuala Lumpur, 8.ix.1969, R. Pilet (CHSM).

###### Diagnostic description.

**Figure 75.**
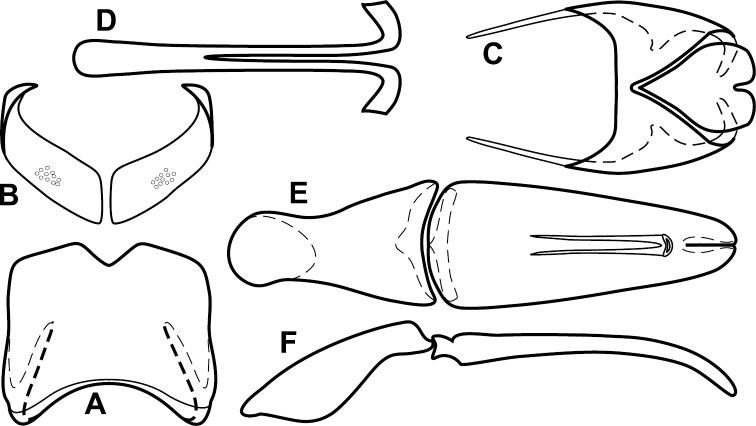
Male genitalia of *Baconia glauca*. **A** T8 **B** S8 **C** T9 & T10 **D** S9 **E** Aedeagus, dorsal view **F **Aedeagus, lateral view.

Length: 2.1mm, width: 1.7mm; body elongate oval, moderately strongly depressed, glabrous; color metallic blue dorsally (pygidia, elytra, pronotum, head), rufobrunneus ventrally; head with frons depressed at middle, ground punctation rather coarse, with few secondary punctures at middle and at upper margin, frontal stria complete across front, weakly sinuate at middle; antennal scape short, club nearly circular; epistoma convex, truncate apically; labrum about twice as wide as long, weakly depressed at middle, apical margin arcuate; mandibles short, each with strong, acute basal tooth; pronotum with sides weakly convergent, rounded to apex, lateral marginal stria descending to ventral edge in posterior half, continuous anteriorly with complete anterior marginal stria, lateral submarginal stria absent, pronotal disk weakly depressed in anterolateral corners, with fine ground punctation, conspicuous secondary punctures denser toward sides; elytra with two complete epipleural striae, outer subhumeral stria absent, inner subhumeral stria complete, dorsal striae 1–5 complete to base, weakly abbreviated apically, sutural stria present in apical half or more, fading to base, elytral disk with scattered secondary punctures in apical one-eighth; prosternum broad, keel truncate at base, with carinal striae complete, sinuate, separate; prosternal lobe slightly less than one-half keel length, apical margin bluntly rounded, with marginal stria well impressed only at middle; mesoventrite broadly emarginate, with marginal stria absent from middle two-thirds; mesometaventral suture arched forward, mesometaventral stria arched more strongly forward, transverse at middle, continuous laterally with oblique lateral metaventral stria, which extends posterad toward middle of metacoxa, metaventral disk coarsely punctate at sides, impunctate at middle; abdominal ventrite 1 with complete inner lateral stria and abbreviated outer lateral stria behind metacoxa, disk with coarse secondary punctures only at sides, ventrites 2–5 with sparse secondary punctures at sides, impunctate at middle; protibia tridentate, the middle tooth closer to apical than basal, outer margin serrulate between teeth; mesotibia with two very weak marginal spines; outer metatibial margin smooth; propygidium lacking basal stria, with sparse, fine ground punctation, with coarse secondary punctures sparsely but evenly interspersed, propygidial gland openings slightly elongate, about midway between anterior and posterior margins, about one-fourth from lateral corners; pygidium strongly convex, with dense, fine ground punctation and coarser, secondary punctation mainly along basal margin. Male genitalia (Fig. 75): T8 broad, sides subparallel, basal emargination very shallow, basal rim well sclerotized, ventrolateral apodemes reaching longitudinal midpoint beneath, separated by about two-thirds T8 width; S8 halves approximate near bases, with conspicuous basal pore clusters on each side, inner margins rapidly diverging apically, sides divergent, apical guides poorly developed, apices connected by ventral velar membrane with vague sclerotizations; T9 with proximal apodemes thin, about one-half total length, dorsal lobes fused at middle, apices narrow, with convergent digitiform apical processes, ventrolateral apodemes well developed, dentate beneath; S9 stem strongly desclerotized along midline, nearly divided to base, sides narrowed to base, apical arms divergent, slightly recurved; tegmen broad at base, evenly narrowed to apex, thin in lateral aspect, curving ventrad in apical half; median lobe simple, about two-thirds tegmen length; basal piece widening apically to width of tegmen, almost two-thirds tegmen length.

###### Remarks.

Surprisingly, this species is not closely related to the other Asian *Baconia*, thoseof the *Baconia aeneomicans* group. Its male genitalia are in fact highly divergent from anything else in the genus. Externally, its metallic blue coloration (Fig. 73C), strongly depressed body form, and 4^th^ dorsal stria ending free at base, not arched mediad toward the suture, will distinguish it easily from all other known SE Asian species.

Despite the fact that we have not been able to study the type specimen, Marseul’s description is perfect compatible with the specimen we attribute to this species, and is inconsistent with any of the members of the *Baconia aeneomicans* group known from SE Asia.

##### Baconia coerulea

(Bickhardt, 1917)

http://species-id.net/wiki/Baconia_coerulea

Figs 73D–E 76 Map 22

Phelister coeruleus Bickhardt, 1917: 215; *Baconia coerulea*: Mazur 1984: 280.

###### Type locality.

BRAZIL: Pará [exact locality unknown].

###### Type material.

**Lectotype**, here designated (ZMHB): “Pará, 10/2 93” / “*coeruleus* n. sp. Bickh.” / “*coeruleus* Bickh.” / “*Phelister coeruleus* Bickhardt, 1917 ex. Coll. Schmidt-Bickhardt / “Type” / “LECTOTYPE *Phelister coeruleus* Bickhardt, M.S.Caterino & A.K.Tishechkin des. 2010”. This species was described from an unspecified number of specimens, and the lectotype designation fixes primary type status on the only known original specimen.

###### Other material.

**VENEZUELA**, 2: **Anzoátegui**: Los Naranjos, Rio Neveri (+ or -) 900 m, 25.viii.1966, L. Joly (FMNH, MHNLS); 1: **Bolívar**: Carret. El Dorado-S. Elena, 6°6'N, 61°23'W, 88 m, 29.ix.1967, L. Joly (MHNLS).

###### Diagnostic description.

**Figure 76.**
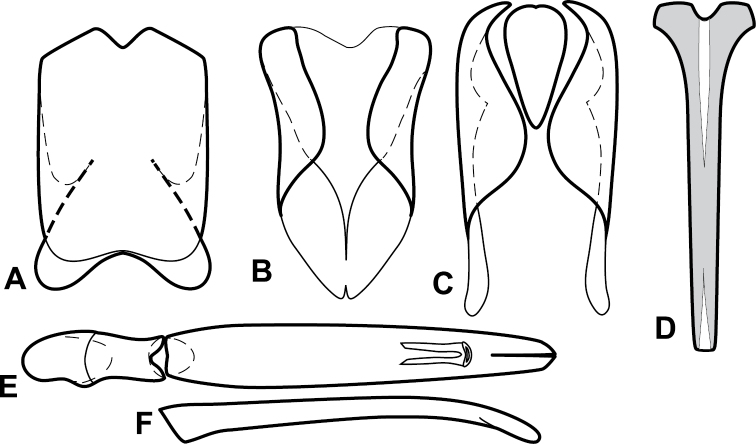
Male genitalia of *Baconia coerulea*. **A** T8 **B** S8 **C** T9 & T10 **D** S9 **E** Aedeagus, dorsal view **F** Aedeagus, lateral view.

Length: 2.0–2.1mm, width: 1.8–1.9mm; body elongate oval, narrowing anterad, weakly convex, glabrous; head and pronotum weakly metallic blue to greenish-blue, elytra more distinctly metallic blue, pygidia and venter rufobrunneus to rufopiceous; frons elevated more or less transversely above antennal bases, frontal stria complete, obtusely subangulate at middle, frontal disk depressed behind, with very conspicuous ground punctation and few coarser punctures on epistoma and middle of frons, supraorbital stria absent, epistoma convex anteriorly, labrum about 4×wider than long, broadly and weakly emarginate apically; both mandibles with small, acute basal tooth; antennal scape short, club slightly asymmetrically elongate oval; pronotal sides rather evenly, weakly convergent in basal two-thirds, abruptly arcuate to apex, marginal stria complete along lateral and anterior margins; lateral submarginal pronotal stria present along basal three-fourths of margin, pronotal disk not depressed mediad, only very narrowly in anterior corners, ground punctation of pronotal disk rather conspicuous, sparsely interspersed with small secondary punctures almost throughout except along midline; elytra with three complete epipleural striae, outer subhumeral stria absent, inner subhumeral stria present in basal two-thirds, may be weakened or interrupted at middle, dorsal striae 1–5 more or less complete but with inner striae increasingly abbreviated apically, sutural stria present in apical three-fourths, may extend faintly to base, elytral disk with coarse punctures in apical third; prosternum narrow, weakly convex, keel emarginate at base, carinal striae convergent between coxae, divergent anterad and posterad; prosternal lobe just over one-half keel length, apical margin rounded, marginal stria obsolete at sides; mesoventrite produced at middle, marginal stria complete, mesometaventral stria arched forward, crenulate, complete, meeting lateral metaventral stria at sides, which curves posterolaterad toward middle of metacoxa, outer lateral metaventral stria about half length of inner stria, oblique, metaventral disk impunctate at middle; abdominal ventrite 1 with single, complete lateral stria, middle portion of disk impunctate; protibia with 4 marginal denticles, outer margin serrulate between; mesotibia with one marginal and 1-2 weak submarginal spines; outer metatibial margin smooth; propygidium without basal stria, discal punctures moderately large, separated by about their diameters in basal half, smaller and sparser apically; propygidial gland openings evident one-third from basal and lateral margins; pygidium with ground punctation fine, rather dense, interspersed with small secondary punctures mainly in basal half, apex of pygidium with distinctive, whorled microsculpture, this possibly more conspicuous in male. Male genitalia (Fig. 76): T8 slightly longer than broad, sides subparallel, basal emargination shallow, basal rim slightly explanate, ventrolateral apodemes reaching longitudinal midpoint beneath, separated by about one-half T8 width; S8 halves fused near bases, inner margins diverging apically, sides curved outward slightly to apex, apical guides well developed, similar in width from midpoint to apex, apices connected by ventral velar membrane; T9 with proximal apodemes about one-third total length, dorsal lobes narrowed apically, with convergent, subacute apices, ventrolateral apodemes weakly developed; S9 desclerotized along midline in apical half, sides narrowed to base, apical arms divergent, obliquely subquadrate; tegmen elongate, narrow at base, widened one-fourth from base, weakly curved to apex, weakly curved ventrad near apex; median lobe simple, very short; basal piece about one-third tegmen length.

###### Remarks.

*Baconia coerulea* may be distinguished by its elongate ovoid body form (Fig. 73D), its nearly complete lateral submarginal pronotal stria, 5 more or less complete elytral striae, and distinctive (possibly sexually dimorphic) apical pygidial microsculpture (Fig. 73E).

This species is generally similar to members of the *Baconia godmani* group, but differences in a number of characters make this ambiguous: more convex body, non-depressed frons, complete lateral submarginal pronotal stria, transversely carinate labrum, and lack of distinct setal fringe along the male’s 8^th^ sternite. However, the brief fusion of the bases of the halves of the 8^th^ sternite bear some resemblance to the completely fused sternite of *Baconia dives*, *Baconia eximia*, and *Baconia varicolor*, and this may be a significant indicator of their relationship.

##### Baconia angulifrons sp. n.

http://zoobank.org/A67D3BFA-EB55-44B2-87C9-A9959646ECDF

http://species-id.net/wiki/Baconia_angulifrons

Figs 77A–B Map 22

###### Type locality.

FRENCH GUIANA: Belvèdére de Saül [3.01°N, 53.21°W].

###### Type material.

**Holotype female**: “**GUYANE FRANÇAISE**:Belvèdére de Saül, point de vue. 3°1'22"N, 53°12'34"W, Piège vitre, 10.xii.2010, SEAG leg.” / “Caterino/Tishechkin Exosternini Voucher EXO-01319” (MNHN). **Paratype** (1): **BRAZIL: Pará**: Jacareacanga, x.1959, M. Alvarenga (UFPR).

###### Other material.

1: **BRAZIL**: **Mato Grosso**:Mpio. Cotriguaçu, Fazenda São Nicolau, Mata Norte, 9°49.15'S, 58°15.6'W, 8–14.xii.2010, FIT, F. Vaz-de-Mello (CEMT). 1: **PERU**: **San Martín**: Tarapoto, v–viii.1886, M. de Mathan (MNHN).

###### Diagnostic description.

Length: 2.3–2.5mm, width: 1.9–2.1mm; body broadly elongate oval, sides subparallel, weakly depressed, glabrous; body color varied, dorsum metallic, head and pronotum metallic greenish-blue to weakly violet bronze, elytra may be similar in color to pronotum or slightly contrasting metallic blue, pygidia and venter rufo-piceous; frons elevated, convex over antennal bases and along sides of frontal stria, slightly depressed behind, ground punctation rather conspicuous, slightly but markedly both finer and denser anterad frontal stria, epistoma and frons lacking secondary punctures, frontal stria present along inner margin of eye, usually interrupted over antennal bases but complete across middle, rather strongly descending toward epistoma, angulate medially; supraorbital stria absent; epistoma weakly convex throughout, apical margin weakly emarginate; labrum about 3×wider than long, weakly emarginate apically; antennal scape short, club broadly rounded; pronotal sides subparallel in basal half, abruptly arcuate to apex, only very weakly depressed in anterior corners, marginal stria complete along lateral and anterior margins, slightly crenulate in front, lateral submarginal stria absent, ground punctation of pronotal disk conspicuous, secondary punctures limited to about lateral fourth; elytra with three complete epipleural striae, outer subhumeral stria absent, inner subhumeral stria present in basal fourth, dorsal striae 1–4 more or less complete, the inner striae faintly abbreviated apically, 5^th^ stria generally present in most of apical two-thirds, but often weak or fragmented, sutural stria present in apical two-thirds, slightly longer than 5^th^, elytral disk with few coarse punctures in apical fourth; prosternum rather narrow, weakly convex, keel very shallowly emarginate at base, carinal striae complete, convergent anterad, separate throughout; prosternal lobe about two-thirds keel length, apical margin narrowly rounded, marginal stria well impressed at middle, obsolete at sides; mesoventrite sinuate to weakly produced, marginal stria interrupted, mesometaventral stria arched forward, subangulate at middle, crenulate, continuous at sides with lateral metaventral stria, which curves posterolaterad toward outer third of metacoxa, outer lateral metaventral stria present, short, divergent from base of inner stria, metaventral disk impunctate at middle; abdominal ventrite 1 with single lateral stria slightly abbreviated apically, middle portion of disk impunctate; protibia 4–5 dentate, basal denticles weak, outer margin serrulate between teeth; mesotibia with one marginal spine and few submarginal denticles basally; outer metatibial margin smooth; propygidium without basal stria, with ground punctation fine, conspicuous, discal punctures rather small, densest in basal third, separated by about their diameters, smaller and sparser apically; propygidial gland openings small but evident about one-third from anterior and one-fourth from lateral margins; pygidium with ground punctation conspicuous, secondary punctures small, restricted to narrow band along basal margin. Male: not known.

**Figure 77.**
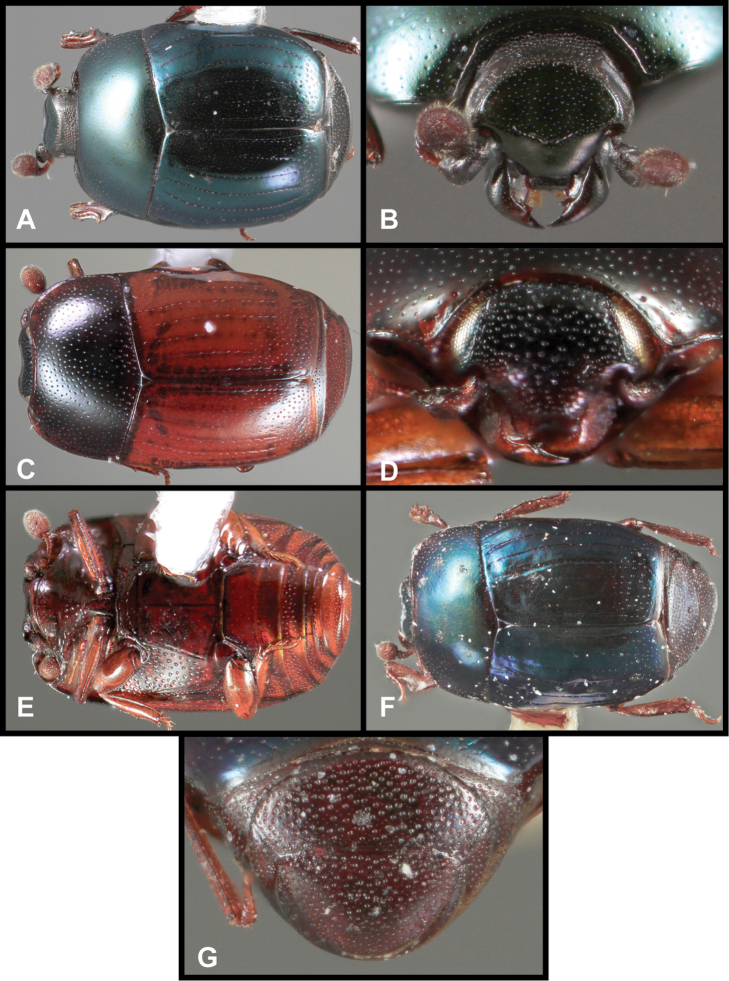
*Baconia* incertae sedis spp. **A** Dorsal habitus of *Baconia angulifrons* **B** Frons of *Baconia angulifrons* **C **Dorsal habitus of *Baconia sanguinea* **D** Frons of *Baconia sanguinea* **E** Ventral habitus of *Baconia sanguinea* **F** Dorsal habitus of male *Baconia viridimicans* **G** Pygidia of *Baconia viridimicans*.

###### Remarks.

The four female specimens representing this species are all from fairly disparate localities, and show moderate variations in coloration and elytral striation. We limit the type ‘locality’ to northeastern Brazil and French Guiana, as these are the most proximate and similar. The species is fairly distinctive, with its broadly elongate body form (Fig. 77A), rather complete elytral striation, and especially by frontal characters, with the frontal stria curving anterad toward the epistoma and subangulate at the middle (Fig. 77B), and the general lack of secondary punctures on the epistoma and frons. Apart from these frontal characters this species shows some similarity to members of the *Baconia godmani* group. At the same time, the frontal stria is rather similar to that found in the preceding species, *Baconia coerulea*, although they are otherwise dissimilar.

###### Etymology.

This species’ name refers to its subangulate frontal stria.

##### Baconia sanguinea sp. n.

http://zoobank.org/962C2522-FE50-47F3-86FF-C6F9EF910306

http://species-id.net/wiki/Baconia_sanguinea

Figs 77C–E 78 Map 22

###### Type locality.

ECUADOR: Orellana: Tiputini Biodiversity Station [0.635°S, 76.150°W].

###### Type material.

**Holotype male**: “**ECUADOR: Depto. Orellana,** Tiputini Biodiversity Station 0°37'55"S, 76°08'39"W 220–250m. 21 October 1998 T.L.Erwin et al. collectors” / “insecticidal fogging of mostly bare green leaves, some with covering of lichenous or bryophytic plants **Lot 1998 Trans. 10 Sta. 9**” / “Caterino/Tishechkin Exosternini Voucher EXO-00462” (USNM). **Paratypes** (3): 1: same locality as type, 29.vi.1998, fogging, T. Erwin (USNM, USFQ); 1: Res. Ethnica Waorani, 1 km S Onkone Gare Camp, Trans. Ent., 0°39'10"S, 76°26'W, 220 m, 29.vi.1994, fogging, T. Erwin (USNM), 1: 8.x.1995, fogging, T. Erwin (USNM).

###### Diagnostic description.

**Figure 78.**
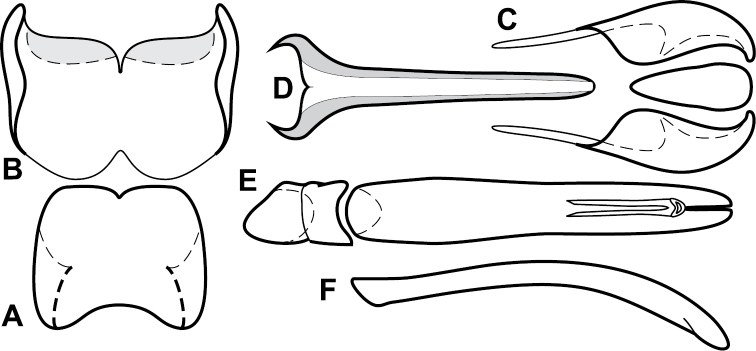
Male genitalia of *Baconia sanguinea*. **A** T8 **B** S8 **C** T9 & T10 **D** S9 **E** Aedeagus, dorsal view **F** Aedeagus, lateral view.

Length: 1.6–1.8mm, width: 1.1–1.3mm; body narrowly elongate oval, subparallel-sided, moderately depressed, glabrous; subtly bicolored, with pronotum and head rufobrunneous, elytra, pygidia and venter rufescent; frons distinctly swollen and faintly microsculptured over antennal bases, depressed at middle, with small, rather deep punctures separated by about 2× their diameters at middle, denser toward eyes and vertex; frontal and supraorbital striae absent; antennal scape short, subtriangular, club oblong; epistoma weakly elevated along apical margin, faintly emarginate; labrum about 4×wider than long, apical margin distinctly emarginate; mandibles short, each with conspicuous, acute basal tooth; pronotum with sides subparallel in basal half, weakly arcuate to apex, lateral and submarginal striae merging behind anterior corner, continued along anterior margin; pronotal disk weakly depressed in anterolateral corners, with very small punctures sparsely, uniformly scattered throughout; elytra with two complete epipleural striae, outer subhumeral stria absent, short fragment of inner subhumeral stria present at base, stria 1 nearly complete, striae 2–5 present to base, progressively abbreviated apically, sutural stria obsolete in basal half and apical fourth, elytral disk with small, sparse punctures in apical third; prosternal keel moderately narrow, shallowly emarginate at base, carinal striae subparallel in basal half, united just anterad midpoint; prosternal lobe about two-thirds keel length, apical margin deflexed, bluntly rounded, marginal stria obsolete at sides; mesoventrite weakly produced at middle, marginal stria complete; mesometaventral stria broadly arched forward, crenulate, continuous at sides with inner lateral metaventral stria, which extends obliquely posterad toward middle of metacoxa, outer lateral metaventral stria absent; metaventral disk impunctate at middle; abdominal ventrite 1 with single lateral stria, slightly abbreviated apically, ventrites 2–5 with sparse punctures at sides, finely and sparsely punctate across middle; protibia tridentate, widest basad midpoint, the outer margin finely serrulate between denticles; mesotibia with two marginal spines; outer metatibial margin smooth; propygidium lacking basal stria, ocellate punctures separated more or less uniformly by slightly less than their widths, denser toward base, propygidial gland openings evident, located about one-third behind anterior margin, one-fourth from lateral corners, the immediately surrounding disk devoid of punctures; pygidium more or less uniformly punctate, the punctures slightly smaller and denser toward apex. Male genitalia (Fig. 78): T8 small and weakly sclerotized, basal emargination shallow, ventrolateral apodemes with inner apices widely separated, projecting short of ventral midpoint, obsolete apically, apical margin shallowly emarginate; S8 about 1.5× longer and broader than T8, halves weakly sclerotized but apparently fused along midline, sides subparallel, diverging slightly, apices projecting at corners, broadly emarginate between, with apical pair of weakly sclerotized, tomentose velar disks; T9 with basal apodemes thin, nearly half entire length, T9 sides rounded, convergent apically, without apical setae, ventrolateral apodemes bluntly produced beneath; T10 narrow, weakly sclerotized; S9 desclerotized along midline, with stem narrow, weakly expanded to base, head abruptly widened near apex, apices subacute, apical emargination broad; tegmen widest just distad base, sides sinuate, narrowed to middle, weakly bulbous apically, apices narrowly rounded, tegmen weakly curved ventrad over much of its length; median lobe simple, about one-third tegmen length; basal piece about one-fourth tegmen length.

###### Remarks.

This species is rather isolated, although it has some general similarity with members of the *Baconia aeneomicans* group, including presence of complete lateral submarginal pronotal stria, inwardly bent bases of 4^th^ and 5^th^ elytral striae, and generally subdepressed, elongate body form (Fig. 77C). The male genitalia are difficult to interpret, as the 8^th^ sternite appears fused into a single sclerite, but its apical velae are unique, and the general form of the 9^th^ tergite and sternite (Figs 78C–D) are quite different from any in the *Baconia aeneomicans* group. It is generally easy to recognize, with its pronotum distinctly darker than its rufescent elytra, prosternal carinal striae united near middle (Fig. 77E), and the epistoma elevated along apical margin and rather coarsely microsculptured (Fig. 77D).

###### Etymology.

This species is named for the red color of the elytra.

##### Baconia viridimicans

(Schmidt, 1893)

http://species-id.net/wiki/Baconia_viridimicans

Figs 77F–G 79

Phelister viridimicans Schmidt, 1893b: 83; *Baconia viridimicans*: Mazur 1984: 281.

###### Type locality.

BRAZIL [exact locality unknown].

###### Type material.

**Lectotype male**, here designated (ZMHB): “Brasil” / “*viridimicans* Schm.” / “*viridimicans*” / “Type” / “coll. J.Schmidt” / “*Phelister viridimicans* Schmidt, 1893, ex. Coll. Schmidt-Bickhardt” / “Caterino/Tishechkin Exosternini Voucher EXO-00423” / “LECTOTYPE *Phelister viridimicans* Schmidt, M.S.Caterino & A.K.Tishechkin des. 2010”. This species was described from an unspecified number of specimens, and the lectotype designation fixes primary type status on the only known original specimen.

###### Diagnostic description.

**Figure 79.**
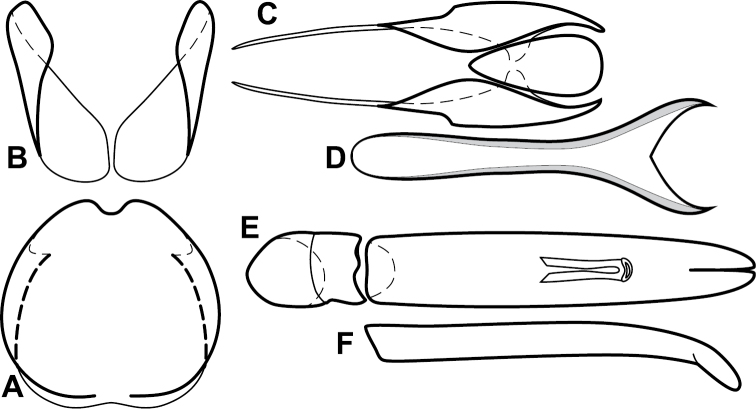
Male genitalia of *Baconia viridimicans*. **A** T8 **B** S8 **C** T9 & T10 **D** S9 **E** Aedeagus, dorsal view **F** Aedeagus, lateral view.

Length: 2.2mm, width: 1.7mm; body rather narrowly elongate oval, subparallel-sided, only weakly depressed, glabrous; head and pronotum metallic greenish-blue, elytra slightly contrasting metallic blue, pygidia and venter rufobrunneus; frons rather strongly convex over antennal bases, shallowly depressed along midline, ground punctation rather conspicuous, especially on epistoma, with few secondary punctures within frontal depression, frontal stria present along inner margin of eye, absent across middle; epistoma convex in apical half; labrum about 3×wider than long, apical margin straight; both mandibles with acute basal tooth; antennal scape short, apex obliquely truncate, club small, nearly circular; pronotal sides subparallel in basal half, arcuate to apex, disk not depressed in anterior corners, marginal stria complete along lateral and anterior margins, slightly crenulate in front, lateral submarginal stria absent, ground punctation of pronotal disk fine, with conspicuous secondary punctures in lateral thirds, slightly denser toward sides; elytra with two complete epipleural striae, outer subhumeral stria absent, inner subhumeral stria present in basal fourth, dorsal striae –4 complete, 4^th^ stria slightly abbreviated from apex, 5^th^ stria present as short fragment behind middle, sutural stria present in apical half, elytral disk with few coarse punctures in apical fifth; prosternal keel narrow, convex, basal margin truncate, carinal striae complete, united along basal margin, weakly divergent anterad; prosternal lobe about one-half keel length, broadly rounded, marginal stria obsolete at sides; mesoventrite weakly sinuate, marginal stria complete, mesometaventral stria arched forward, weakly crenulate, continued by sinuate lateral metaventral stria posterolaterad toward outer third of metacoxa, more or less complete, outer lateral metaventral stria represented by only fine basal fragment, metaventral disk impunctate at middle; abdominal ventrite 1 with inner lateral stria complete, outer stria present only behind metacoxa, middle portion of disk impunctate; protibia 4-5 dentate, basal denticles weak, outer margin finely serrulate between teeth; mesotibia with two distinct marginal spines and few weak submarginal spines toward base; outer metatibial margin smooth; propygidium without basal stria, propygidium and pygidium almost uniformly covered with small, ocellate punctures separated by slightly less than their diameters, only slightly smaller toward pygidial apex; propygidial gland openings evident midway from anterior margin and almost one-third from lateral margins. Male genitalia (Fig. 79): T8 short, broad, sides rounded, basal emargination very shallow, basal rim well sclerotized, ventrolateral apodemes reaching distad midpoint beneath, widely separated; S8 halves approximate near bases, inner margins diverging apically, sides weakly divergent, apical guides well developed toward apex; T9 with proximal apodemes thin, about one-half total length, dorsal lobes separated, narrow, apices narrowly subacute, ventrolateral apodemes well developed, narrowly, bluntly rounded beneath; S9 stem desclerotized along midline, sides weakly widened to base, apical arms divergent, curving to apices, apical emargination deep; tegmen sides subparallel in basal two-thirds, narrowed to apex, weakly curving ventrad in apical fourth; median lobe simple, one-fourth tegmen length; basal piece one-fourth tegmen length.

###### Remarks.

*Baconia viridimicans* is recognizeable by its narrowly elongate body form (Fig. 77F), swollen frontal sides and epistoma, nearly complete elytral striation, and almost uniformly punctate pygidia (Fig. 77G). The species is only known from the type specimen. It is possible that this species belongs near or within the *Baconia famelica* group, and it has similarities to *Baconia cavifrons* in particular, especially the short, nearly spherical antennal club. Male genitalia are similar in the two, but they are very generalized and not particularly informative.

##### Baconia nayarita sp. n.

http://zoobank.org/DB63342A-13F1-4393-9FDC-EBFC57AE8771

http://species-id.net/wiki/Baconia_nayarita

**Figure 80.**
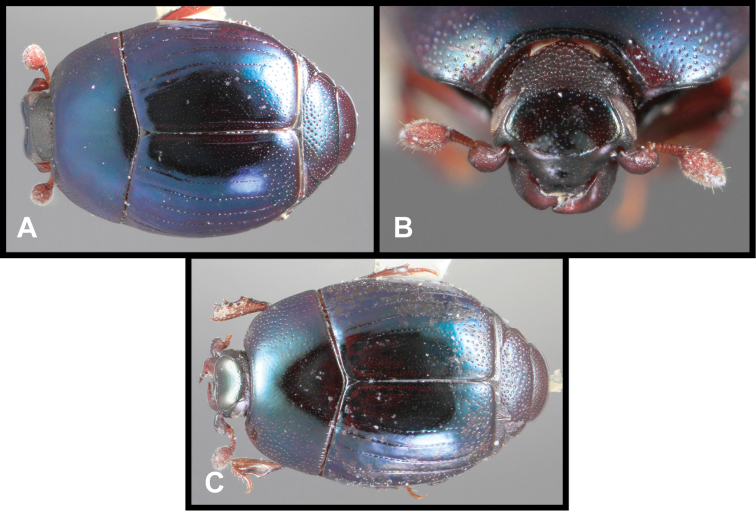
*Baconia* incertae sedis spp. **A** Dorsal habitus of *Baconia nayarita* **B** Frons of *Baconia nayarita* **C** Dorsal habitus of *Baconia viridis*.

Figs 80A–B 81A–F Map 22

###### Type locality.

MEXICO: Nayarit: Tepic [exact locality unknown]

###### Type material.

**Holotype male**: “El Cora, Tepic, Ad.Lüdecke” / “Zool. Mus. Berlin” / “Caterino/Tishechkin Exosternini Voucher EXO-00494” (ZMHB). **Paratypes** (3): 3: **MEXICO**: **Nayarit**: 48 km SE Tepic, 1188 m, 19.viii.1976, E. Ross (CASC, MSCC).

###### Diagnostic description.

**Figure 81.**
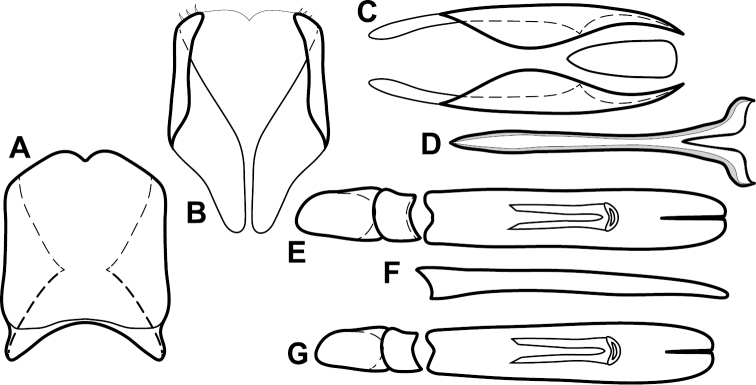
Male genitalia of *Baconia* spp. **A–F** *Baconia nayarita* **A** T8 **B** S8 **C** T9 & T10 **D** S9 **E** Aedeagus, dorsal view **F** Aedeagus, lateral view **G** Aedeagus of *Baconia viridis*, dorsal view.

Length: 2.5–2.6mm, width: 2.2–2.3mm; body elongate oval, only weakly depressed, glabrous; body coloration somewhat variable, dorsum metallic blue to faintly greenish-blue to near violet, more or less uniform, but head and/or pronotum may appear slightly greener than elytra, venter rufopiceous; frons elevated over antennal bases, depressed along antero-posterior midline, ground punctation rather conspicuous, with few secondary punctures on epistoma, within frontal depression and near vertex, frontal stria present along inner margin of eye, curving mediad, but usually somewhat fragmented across middle, supraorbital stria may be absent or vaguely indicated; antennal scape short, club rounded; epistoma truncate to weakly emarginate; labrum about 3×wider than long, weakly emarginate along apical margin; both mandibles with acute basal tooth; pronotal sides weakly, evenly arcuate to apex, not depressed in anterior corners, marginal stria complete along lateral and anterior margins, slightly crenulate in front, lateral submarginal stria absent, ground punctation of pronotal disk fine, with conspicuous secondary punctures in lateral thirds, denser toward sides; elytra with three complete epipleural striae, outer subhumeral stria absent, inner subhumeral stria present at base and as median fragment (continuous in type), dorsal striae 1–3 complete, 4^th^ stria slightly abbreviated from apex, 5^th^ stria absent or only very weakly indicated near apex, sutural stria present in apical half to two-thirds, elytral disk with few coarse punctures in apical fifth; prosternum weakly convex, keel weakly emarginate at base, carinal striae complete, subparallel, separate throughout; prosternal lobe about one-half keel length, apical margin bluntly rounded, marginal stria obsolete at sides; mesoventrite weakly produced, marginal stria complete, mesometaventral stria absent; lateral metaventral stria extending from near mesocoxa posterolaterad toward middle of metacoxa, more or less complete, outer lateral metaventral stria parallel to inner stria for about two-thirds its length, metaventral disk impunctate at middle; abdominal ventrite 1 with inner lateral stria abbreviated, outer stria absent, middle portion of disk impunctate; protibia 4–5 dentate, basal denticles weak, outer margin serrulate between teeth; mesotibia with one marginal spine and short series of very weak submarginal spines toward base; outer metatibial margin smooth; propygidium without basal stria, discal punctures ocellate, largest basomedially, smaller and sparser posterad and laterad; propygidial gland openings evident about one-third from anterior margin and one-fourth from lateral margins; pygidium with ground punctation conspicuous, secondary punctures very small and restricted to near basal margin. Male genitalia (Fig. 81): T8 slightly longer than wide, sides subparallel, basal emargination shallow, broadly arcuate, basal rim slightly explanate, subbasal membrane attachment line evident, ventrolateral apodemes reaching longitudinal midpoint beneath, separated by about one-half T8 width, evenly tapered to apex; S8 halves approximate in basal half, inner margins rapidly diverging to apex, sides subparallel to converging slightly, apical guides narrowly developed, apices bearing a few inconspicuous setae, connected by thin ventral velar membrane; T9 with proximal apodemes short, about one-fourth total length, dorsal lobes elongate, narrowed apically, ventrolateral apodemes weakly dentate beneath; S9 desclerotized along midline, divided in apical third, stem narrow, constricted slightly at middle, apical arms divergent, apices acute; tegmen with sides very weakly widening to apex, slightly sinuate, thin, straight in lateral aspect; median lobe simple, one-third tegmen length; basal piece one-third tegmen length.

###### Remarks.

This species is very similar to *Baconia viridis*, below, even in male genitalic characters, and can best be distinguished by its larger body size, more blue-to-violet dorsal coloration (Fig. 80A), presence of the sutural elytral stria, and nearly complete frontal stria (Fig. 80B). Externally both of these species look much like members of the *Baconia godmani* group, but their male genitalia are quite different, lacking the conspicuous apical fringe of the 8^th^ sternite.

###### Etymology.

The species name reflects the state of Mexico from which it is known.

##### Baconia viridis sp. n.

http://zoobank.org/FB705304-84E4-40EB-898D-03CF0907FB72

http://species-id.net/wiki/Baconia_viridis

Figs 80C 81G Map 22

###### Type locality.

MEXICO: Jalisco: Chamela Biological Station [19.49°N, 105.04°W].

###### Type material.

**Holotype male**: “MEXICO:Jalisco: Chamela Biol. Stn., 14 July 1989, Robert W. Brooks #035 ex. flight intercept trap” / “SEMC0903649 KUNHM-ENT” (SEMC). **Paratypes** (2): **MEXICO: Jalisco:** 1: San Cristobal de la Barranca, Rancho el Tablon, 21°3'36"N, 103°25'50"W, 850 m, 31.vii.2004, FIT, F. Vaz-de-Mello (AKTC); 1: **Guerrero**: 2 mi. N. Cacahuamilpa, 5300 ft., 4.vii.1987, P. Kovarik (CHPWK).

###### Other material.

1: **MEXICO: Chiapas**: El Aguacero, 16 km W Ocozocoautla, 680 m, 5–13.vi.1990, FIT, H. & A. Howden (CMNC).

###### Diagnostic description.

Length: 2.1–2.2mm, width: 1.8–1.9mm; body elongate oval, only weakly depressed, glabrous; dorsum metallic blue to faintly greenish-blue, head and pronotum appearing very slightly greener than elytra, pygidia rather faintly metallic, venter rufobrunneus to rufopiceous; frons weakly elevated over antennal bases, depressed along antero-posterior midline, ground punctation rather conspicuous, with few, very small secondary punctures at middle of frontal disk and near vertex, frontal stria present along inner margin of eye, curving mediad, interrupted over antennal bases, continuous across middle, supraorbital stria absent or very faintly indicated; epistoma slightly convex anterad frontal stria, but shallowly depressed at middle, with irregular series of small, very shallow depressions along anterior margin; labrum about 3×wider than long, emarginate along apical margin, coarsely microsculptured; basal tooth of right mandible blunt, tooth of left mandible acute; antennal scape short, club rounded; pronotal sides weakly, evenly arcuate to apex, not depressed in anterior corners, marginal stria complete along lateral and anterior margins, slightly crenulate in front, lateral submarginal stria absent, ground punctation of pronotal disk fine, with conspicuous secondary punctures in lateral thirds, denser toward sides; elytra with two to three epipleural striae, the outermost may be abbreviated apically, outer subhumeral stria absent, inner subhumeral stria present in basal fifth, dorsal striae 1-3 more or less complete, the 3^rd^ stria often abbreviated from apex, 4^th^ stria more distinctly abbreviated from apex, present in about basal two-thirds, 5^th^ and sutural striae absent, elytral disk with few coarse punctures in apical fourth; prosternum rather narrow, weakly convex, keel weakly emarginate at base, carinal striae complete, convergent between coxae, otherwise subparallel, separate throughout; prosternal lobe about one-half keel length, apical margin bluntly rounded, marginal stria obsolete at sides; mesoventrite weakly produced, marginal stria complete, mesometaventral stria absent; lateral metaventral stria extending from near mesocoxa posterolaterad toward middle of metacoxa, more or less complete, outer lateral metaventral stria parallel to inner for half to two-thirds its length, metaventral disk impunctate at middle; abdominal ventrite 1 with inner lateral stria slightly abbreviated, outer stria absent, middle portion of disk impunctate; protibia 4–5 dentate, basal denticles weak, outer margin serrulate between teeth; mesotibia with one marginal spine and short series of very weak submarginal spines toward base; outer metatibial margin smooth; propygidium without basal stria, discal punctures ocellate, rather large at middle, smaller and sparser posterad and laterad; propygidial gland openings evident about one-third from anterior margin and one-fourth from lateral margins, immediately surrounding disk impunctate; pygidium with ground punctation fairly fine, secondary punctures very small and restricted to near basal margin. Male genitalia very similar in most respects to that of *Baconia nayarita*, above (Fig. 81), but with the tegmen slightly shorter, and the sides more evenly, weakly convergent to the base, less sinuate (Fig. 81G).

###### Remarks.

This species is extremely similar to *Baconia nayarita*, above, but can be distinguished by its slightly smaller size, complete lack of sutural stria, and generally more greenish-blue coloration (Fig. 80C). The faint sculpturing of the apical region of the epistoma and microsculptured labrum are also distinctive. The single specimen from Chiapas is a female, and lacking confirming male genitalic characters we exclude it from the type series.

###### Etymology.

This species is named for its greenish-blue dorsal coloration.

##### Baconia purpurata sp. n.

http://zoobank.org/CCCD485F-B56A-488A-AD91-F19258DEC441

http://species-id.net/wiki/Baconia_purpurata

Figs 82A–B Map 22

###### Type locality.

FRENCH GUIANA: Montagne des Chevaux [4.72°N, 52.40°W].

###### Type material.

**Holotype female**: “**GUYANE FRANÇAISE**: Montagne des Chevaux, 4°43'N, 52°24'W, Piège d’interception 22.i.2011, SEAG leg.”/ “Caterino/Tishechkin Exosternini Voucher EXO-02501” (MNHN). **Paratypes** (2): **FRENCH GUIANA:** 1: Bélvédère de Saül, 3°1'22"N, 53°12'34"W,14.ii.2011, FIT, SEAG (CHND). 1: **GUYANA: Region 8**, Iwokrama Forest, Kabocalli Field Stn., 60m, 4°17'4"N, 58°30'35"W, 3-5.vi.2001, R. Brooks, Z. Falin (SEMC).

###### Diagnostic description.

Length: 2.0–2.2mm, width: 1.7–1.9mm; body elongate oval, sides subparallel, weakly depressed, glabrous; pronotum and elytra uniformly metallic violet-bronze, head, pygidia and venter rufo-piceous; frons weakly elevated above antennal bases, slightly depressed at middle, interocular margins strongly convergent dorsad, ground punctation fine, secondary punctures small, sparse, slightly denser toward vertex, frontal stria present along inner margin of eye, curving mediad but absent across front; supraorbital stria absent; epistoma weakly convex along apical margin; labrum about 4×wider than long, weakly bisinuate apically; each mandible with acute basal tooth; antennal scape short, club asymmetrically oblong; pronotal sides subparallel in basal half, abruptly arcuate to apex, depressed in anterior corners, marginal stria complete along lateral and anterior margins, slightly crenulate in front, lateral submarginal stria absent, ground punctation of pronotal disk very fine, secondary punctures uniformly interspersed in lateral thirds; elytra with 2–3 complete epipleural striae, the outer variably abbreviated apically, outer subhumeral stria absent, inner subhumeral stria present as short basal fragment, dorsal striae 1–2 more or less complete, 3^rd^ stria nearly complete to significantly abbreviated apically, 4^th^ stria abbreviated from apex, but generally more nearly complete than 3^rd^ stria, 5^th^ stria absent, sutural stria present in apical two-thirds, elytral disk with few coarse punctures in apical fourth; prosternum rather narrow, distinctly convex, keel shallowly emarginate at base, carinal striae complete, convergent at middle but separate throughout; prosternal lobe about half keel length, apical margin broadly rounded, marginal stria nearly complete, faintly obsolete at sides; mesoventrite sinuate to weakly produced, marginal stria complete, mesometaventral stria weakly arched forward, crenulate, continuous at sides with lateral metaventral stria, which curves posterolaterad toward middle of metacoxa, outer lateral metaventral stria present, short, divergent from base of inner stria, metaventral disk impunctate at middle; abdominal ventrite 1 with single lateral stria more or less complete, middle portion of disk impunctate; protibia 4–5 dentate, basal denticles weak, outer margin serrulate between teeth; mesotibia with two marginal spines; outer metatibial margin smooth; propygidium without basal stria, with ground punctation very fine, inconspicuous, discal punctures rather small, ocellate, separated by their diameters or slightly less in basal half, smaller and sparser apically; propygidial gland openings conspicuous nearly one-half from anterior and one-fourth from lateral margins; pygidium with ground punctation fine, secondary punctures small, very uniformly interspersed throughout. Male: not known.

**Figure 82.**
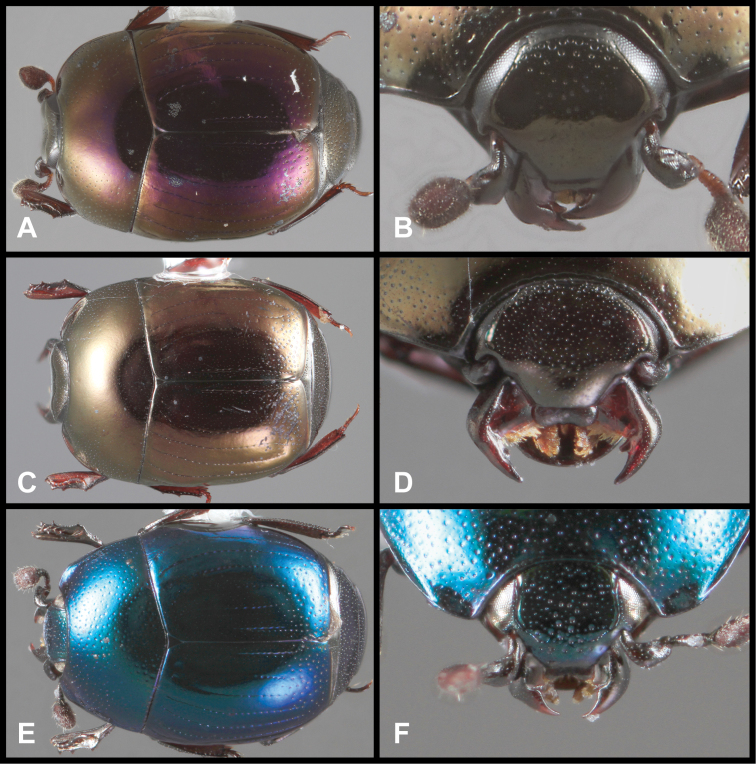
*Baconia* incertae sedis spp. **A** Dorsal habitus of *Baconia purpurata* **B** Frons of *Baconia purpurata* **C **Dorsal habitus of *Baconia aenea* **D** Frons of *Baconia aenea* **E** Dorsal habitus of *Baconia clemens* **F** Frons of male *Baconia clemens*.

###### Remarks.

This species’ color pattern is distinctive, with strongly violet metallic pronotum and elytra (Fig. 82A), but with the head and pygidium similarly colored to non-metallic venter. It is similar and apparently closely related to *Baconia aenea*, which is much more commonly collected in French Guiana – both lack the frontal stria, and may be generally violet in color. However, *Baconia purpurata* is more richly violet in color, lacks the transverse basal propygidial stria, has a narrower frons, a rather small labrum (Fig. 82B), and lacks a basal arch representing the 5^th^ elytral stria.

###### Etymology.

This species’ name refers to its metallic violet coloration.

##### Baconia aenea sp. n.

http://zoobank.org/A484C116-5DF3-46C3-B99C-4B361CCA1B55

http://species-id.net/wiki/Baconia_aenea

**Map 23.**
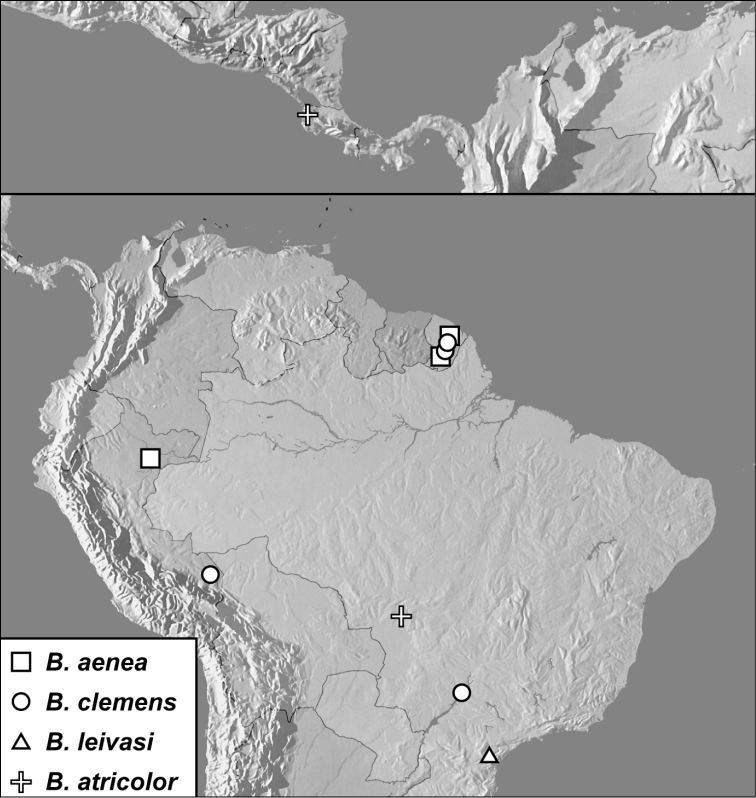
*Baconia* incertae sedis records.

Figs 82C–D 83A–C, G, I–J Map 23

###### Type locality.

FRENCH GUIANA: Montagne des Chevaux [4.72°N, 52.40°W].

###### Type material.

**Holotype male**: “**GUYANE FRANÇAISE**: Montagne des Chevaux, 4°43'N, 52°24'W, Piège d’interception 13 Jun 2009. SEAG leg.” / “Caterino/Tishechkin Exosternini Voucher EXO-00425” (MNHN). **Paratypes** (65): **FRENCH GUIANA**: 4: Montagne des Chevaux, 4°43'N, 52°24'W, 26.xii.2008, 3: 4.i.2009, 1: 9.i.2009, 2: 10.i.2009, 1: 19.i.2009, 4: 25.i.2009, 2: 8.ii.2009, 3: 23.ii.2009, 1: 8.iii.2009, 1: 15.iii.2009, 2: 18.iv.2009, 1: 20.iv.2010, 1: 25.iv.2009, 1: 2.v.2009, 1: 16.v.2009, 1: 13.vi.2009, 1: 20.vi.2009, 2: 27.vi.2009, 4: 4.vii.2009, 7: 11.vii.2009, 3: 19.vii.2009, 4: 27.vii.2009, 1: 1.viii.2009, 2: 9.viii.2009, 1: 19.ix.2009, 1: 10.i.2010, 1: 10.ix.2010, 1: 9.i.2011, 1: 13.ii.2011; 1: Bélvédère de Saül, 3°1'22"N, 53°12'34"W, 7.ii.2011, 1: 14.ii.2011, 1: 17.i.2011; 1: Rés. des Nouragues, Régina, 4°2.27'N, 52°40.35'W, 28.i.2010, 1: 19.ii.2010; 2: Mont tabulaire Itoupé, 3°1.82'N, 53°6.40'W, 400 m, 31.iii.2010 (all FIT, coll. by SEAG; MNHN, CHND, FMNH, MSCC, AKTC).

###### Other material.

1: **PERU: Loreto**, 45 km SW Iquitos, Rio Ucayali, Genaro Herrera, 4°54'S, 73°40'W, FIT, 31.vii.2011, G.Lamarre (CHND).

###### Diagnostic description.

**Figure 83.**
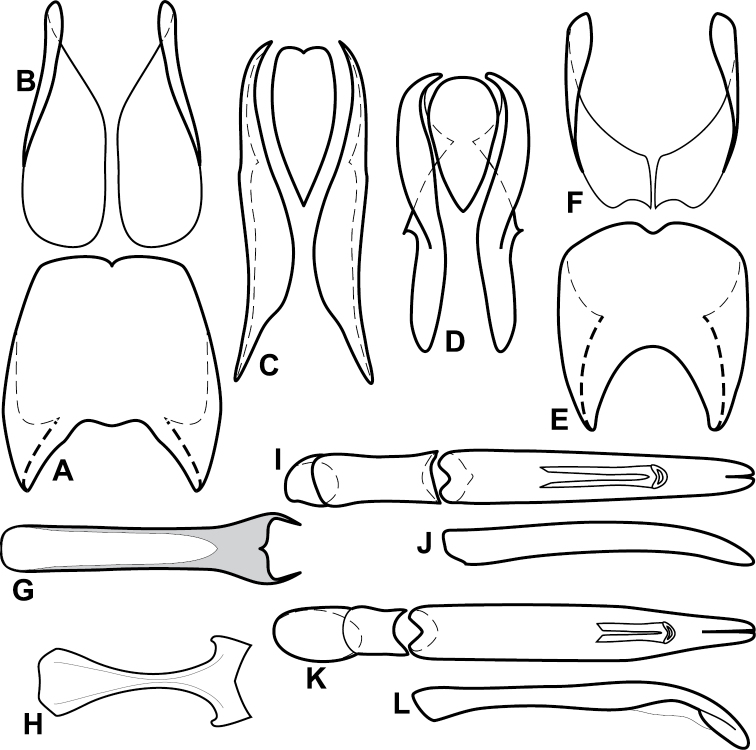
Male genitalia of *Baconia* spp. **A** T8 of *Baconia aenea* **B** S8 of *Baconia aenea* **C** T9 & T10 of *Baconia aenea* **D** T9 & T10 of *Baconia clemens* **E** T8 of *Baconia clemens* **F** S8 of *Baconia clemens* **G** S9 of *Baconia aenea* **H** S9 of *Baconia clemens* **I** Aedeagus, dorsal view of *Baconia aenea* **J** Aedeagus, lateral view of *Baconia aenea* **K** Aedeagus, dorsal view of *Baconia clemens* **L** Aedeagus, lateral view of *Baconia clemens*.

Length: 2.0–2.3mm, width: 1.7–2.1mm; body broadly elongate oval, sides subparallel, moderately depressed, glabrous; head, pronotum and elytra uniformly metallic dark bronze, many individuals with hints of violet, pygidia and venter rufo-piceous; frons broad, rounded above, with interocular margins strongly convergent dorsad, weakly elevated above antennal bases, depressed at middle, ground punctation conspicuous, secondary punctures small, sparse, few at center and near vertex, frontal stria fine, present along inner margin of eye, absent across front; supraorbital stria absent though may be vaguely indicated by series of punctures; epistoma with apical margin weakly emarginate; labrum only about 2.5×wider than long, distinctly bilobed, emarginate in middle; apices of mandibles prolonged, each mandible with acute basal tooth; antennal scape short, club nearly circular; pronotal sides subparallel in basal third, evenly arcuate to apex, only weakly depressed in anterior corners, marginal stria complete along lateral and anterior margins, slightly crenulate in front, lateral submarginal stria absent, ground punctation of pronotal disk very fine at middle, secondary punctures uniformly interspersed in lateral thirds; elytra with 2 complete epipleural striae, outer subhumeral stria absent, inner subhumeral stria present as basal and medial fragments, dorsal striae 1–3 more or less complete, 4^th^ stria variably abbreviated from apex, less commonly complete, 5^th^ stria generally present as very short basal fragment, may arch basally toward suture, sutural stria present in apical two-thirds to three-fourths, elytral disk with coarse punctures in apical third; prosternum rather narrow, distinctly convex, keel shallowly emarginate at base, carinal striae nearly complete, may be abbreviated anteriorly, slightly convergent toward front; prosternal lobe about two-thirds keel length, apical margin rather narrowly rounded, marginal stria well impressed, obsolete at extreme sides; mesoventrite sinuate to weakly produced at middle, marginal stria complete, mesometaventral stria weakly arched forward, crenulate, continuous at sides with lateral metaventral stria, which curves posterolaterad toward middle of metacoxa, outer lateral metaventral stria present, short, divergent from base of inner stria, metaventral disk impunctate at middle; abdominal ventrite 1 with single lateral stria more or less complete, disk with few secondary punctures along apical margin; protibia 4-dentate, basal denticle weak, outer margin serrulate between teeth; mesotibia with two marginal spines; outer metatibial margin smooth; propygidium with transverse basal stria complete, discal punctures moderately large at middle, ocellate, separated by about their diameters, smaller and sparser toward sides; propygidial gland openings small but evident behind ends of transverse stria, one-fourth from lateral margins; pygidial sculpturing sexually dimorphic, that of female uniformly, rather densely covered with secondary puntation, that of the male less densely punctate throughout and with two weakly depressed areas on either side of midline in basal half devoid of secondary punctures but with very dense, fine ground punctures. Male genitalia (Figs 83A–C, G, I–J): T8 slightly longer than wide, sides weakly convergent to apex, basal emargination rather deep, sinuate, apical emargination very shallow, ventrolateral apodemes extending only about one-third distad from base, separated by about one-half T8 width; S8 halves approximate in basal half, narrowing to apex, sides converging apically, apical guides narrow; T9 with proximal apodemes obsolete, dorsal lobes narrowing gradually to base, apices narrowly subacute, ventrolateral apodemes weakly dentate beneath; S9 desclerotized along basal two-thirds of midline, stem moderately broad, widening slightly toward base, apical arms slightly divergent, apices acute; tegmen narrow, sides weakly converging to apex, rather thick, very weakly curved in lateral aspect; median lobe simple, one-half tegmen length; basal piece long, one-half tegmen length.

###### Remarks.

This distinctive species is closely related to *Baconia purpurata*, but the broader frons (Fig. 82D), large and distinctly bilobed labrum, complete basal propygidial stria, presence of a short basal fragment of the 5^th^ elytral stria, bronzy rather than distinctly violet coloration (Fig. 82C), and unique pygidial dimorphism will easily distinguish it. It has been the most abundantly collected species of *Baconia* in French Guiana. The single specimen (female) from Peru is a somewhat surprising geographic outlier, but it does differ in a few characters, most notably having more distinctly violet colored elytra than most members of *Baconia aenea*, as well as having the sutural stria complete and meeting the basal arch of the 5^th^ stria. Therefore we exclude it from the type series. Discovery of a male from near its locality would help evaluate its status.

###### Etymology.

This species’ name refers to its distinct bronzy coloration.

##### Baconia clemens sp. n.

http://zoobank.org/2C0F48BC-4089-49F5-ADFF-FDFE5BB2EC77

http://species-id.net/wiki/Baconia_clemens

Figs 82E–F 83D–F, K–L Map 23

###### Type locality.

BRAZIL: Mato Grosso do Sul: Três Lagoas [20.8°S, 51.7°W]

###### Type material.

**Holotype male**: “BR-MS-Três Lagoas Champion Papel e Celulose Horto Rio Verde, black light trap – *Eucalyptus grandis* stand, Flechtmann, C.A.H. col., 16/XI/1994” / “C-1579” / “Caterino/Tishechkin Exosternini Voucher EXO-00433” (UNESP). **Paratypes** (3): **FRENCH GUIANA**:1: Belvèdére de Saül, 3°1'22"N, 53°12'34"W, 30.xi.2010, FIT, SEAG (CHND); 1: Montagne des Chevaux, 4°43'N, 52°24'W, FIT, 10.iii.2012, SEAG (MNHN). **PERU**:1: **Madre de Dios**: Res. Tambopata, 30 km (air) SW Pto. Maldonado, 12°50'S, 069°20'W, 290 m, 1.ii.2018, canopy fogging, T. Erwin (USNM).

###### Diagnostic description.

Length: 1.8–2.0mm, width: 1.6–1.7mm; body elongate oval, weakly depressed, glabrous; head, pronotum and pygidia metallic greenish-blue to blue, elytra contrasting slightly, more blue than greenish blue, venter rufo-piceous; frons appearing slightly elongate, interocular margins approximately parallel, frontal disk flat, ground punctation conspicuous, secondary punctures numerous and dense, frontal stria extending along inner edge of eye, curving mediad in front but broadly interrupted in middle; supraorbital stria absent; epistoma weakly convex along apical margin; labrum about 3×wider than long, apical margin deeply, subacutely emarginate; mandibles lacking inner marginal teeth; antennal scape short, club elongate oval; pronotum somewhat elongate, sides evenly convergent in basal three-fourths, abruptly arcuate to apex, distinctly depressed in anterior corners, marginal stria complete along lateral and anterior margins, slightly crenulate in front, lateral submarginal stria absent, ground punctation of pronotal disk fine, interspersed with coarse secondary punctures throughout, those anterolaterad scutellum markedly larger; elytra with 2 complete epipleural striae, outer subhumeral stria absent, inner subhumeral stria present in about basal three-fourths, medially interrupted or complete, dorsal striae 1–5 more or less complete, though the inner striae may be variably abbreviated apically, sutural stria present in apical half to two-thirds, elytral disk with coarse punctures in apical third; prosternum moderately broad, weakly convex, keel shallowly emarginate at base, carinal striae complete, divergent anterad, separate throughout; prosternal lobe about two-thirds keel length, rather broad and flat, apical margin bluntly rounded, marginal stria fine across middle, obsolete at sides; mesoventrite weakly produced at middle, marginal stria complete, mesometaventral stria arched forward, crenulate, continuous at sides with lateral metaventral stria, which extends posterolaterad toward middle of metacoxa; outer lateral metaventral stria may be indicated by few connected punctures; metaventral and abdominal disks with ground punctation sparse but rather conspicuous; abdominal ventrite 1 with single lateral stria slightly abbreviated; protibia 4–5 dentate, basal denticles weak, outer margin very finely serrulate; mesotibia with two weak marginal spines; outer metatibial margin smooth; propygidium without basal stria, ground punctation distinct, secondary punctures small but numerous, uniformly scattered, separated by about their diameters; propygidial gland openings evident one-fourth from anterior and lateral margins; pygidium with ground punctation fine, rather dense, secondary punctures small, mostly restricted to basal third. Male genitalia (Figs 83D–F, K–L): T8 with deep basal emargination, sides weakly rounded at base, subparallel to apex, apical emargination very shallow, ventrolateral apodemes extending about one-half distad from base, separated by about two-thirds T8 width; S8 halves approximate in basal fourth, diverging strongly, narrowing slightly toward apex, apices narrowly rounded; T9 with proximal apodemes very thick, not differentiated from dorsal lobes, dorsal lobes narrow, converging, narrowly subacute at apices, ventrolateral apodemes strongly dentate beneath; S9 stem narrow at middle but strongly widened basally and apically, apical arms divergent, with weakly recurved basolateral teeth; tegmen narrow, sides subparallel in basal two-thirds, lateral profile uneven, depressed in basal half, rather thick at middle, then distinctly excavate beneath at subapical foramen; median lobe one-fourth tegmen length; basal piece one-third tegmen length.

###### Remarks.

Despite the widespread localities for the type specimens, this species is quite distinctive and uniform (except in coloration, with the Peruvian specimen’s pronotum distinctly greener), and we choose to unite them all unambiguously as types. The head characters distinguish it most easily, with the frontal disk flat (Fig. 82F), somewhat reclined, subquadrate and punctate, the labrum deeply emarginate, and the mandibles lacking basal teeth. The presence of mostly complete striae 1-5 and the sutural stria is also rather unusual for *Baconia*, as is the almost uniformly punctate pronotum (Fig. 82E).

###### Etymology.

The Latin name *clemens* means tranquil, merciful, or reclined, and alludes both to the lack of mandibular teeth and the ‘reclined’ position of the frons in repose.

##### Baconia leivasi sp. n.

http://zoobank.org/4DDB50CE-7084-46F2-BFBA-074201143A32

http://species-id.net/wiki/Baconia_leivasi

**Figure 84.**
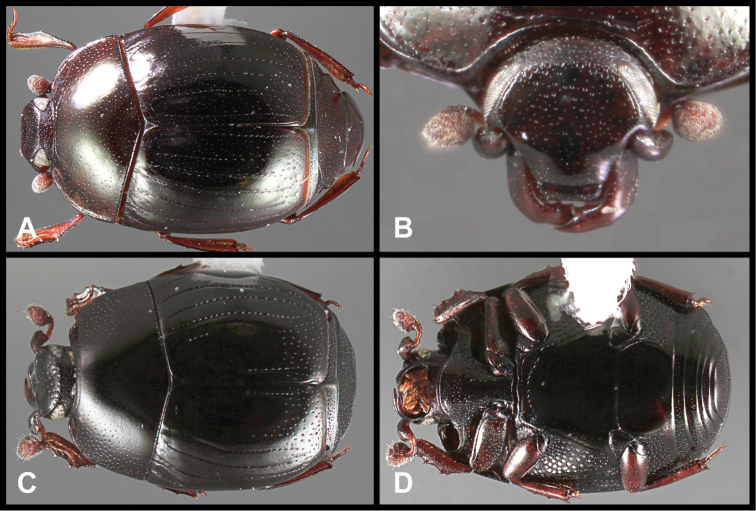
*Baconia* incertae sedis spp. **A** Dorsal habitus of *Baconia leivasi* **B** Frons of *Baconia leivasi* **C** Dorsal habitus of *Baconia atricolor* **D** Ventral habitus of *Baconia atricolor*.

Figs 84A–B 85 Map 23

###### Type locality.

BRAZIL: Paraná: Campina Grande do Sul [25.2965˚S, 49.0381˚W].

###### Type material.

**Holotype male**: “Brasil, Paraná: Campina Grande do Sul, Estrada da Mandaçaia – 1800. 26/XII/2008 F.W.T.Leivas (*leg*.)” / “Interceptação de vôo (FIT)” / “DZUP272498” (UFPR).

###### Diagnostic description.

**Figure 85.**
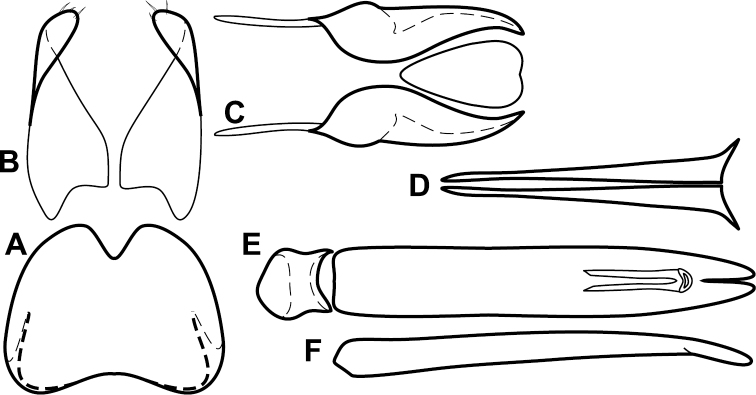
Male genitalia of *Baconia leivasi*. **A** T8 **B** S8 **C** T9 & T10 **D** S9 **E** Aedeagus, dorsal view **F **Aedeagus, lateral view.

Length: 2.6mm, width: 1.6mm; body elongate oval, nearly parallel-sided, subdepressed, glabrous; entire body rufobrunneus to rufopiceous, elytral very faintly bronzy; frons wide, with short, weak anterolateral carinae over antennal bases, frontal stria present only along upper two-thirds of ocular margin, frontal disk weakly depressed at middle, ground punctation fine but conspicuous, few coarser punctures at middle and dorsad, supraorbital stria absent, epistoma wide, convex, labrum about 3×wider than long, very weakly emarginate apically; both mandibles with small, acute basal tooth; antennal scape short, club elongate oval; pronotal sides rather evenly arcuate to apices, marginal stria complete along lateral and anterior margins, with additional transverse submarginal anterior stria detached at sides, lateral submarginal pronotal stria present along middle three-fourths of sides, on inner edge of marginal ridge, pronotal disk depressed alongside; ground punctation of pronotal disk fine, sparse, with very small secondary punctures sparsely interspersed throughout, larger laterally; elytra with two complete epipleural striae, outer subhumeral stria absent, inner subhumeral stria present in basal two-thirds, dorsal striae 1–4 more or less complete, though inner striae progressively abbreviated apically, 5^th^ stria present in basal half, connected by basal arch to complete sutural stria, elytral disk with coarse punctures in apical fourth; prosternum narrow, convex, keel weakly emarginate at base, carinal striae convergent between coxae, divergent and obsolete anterad; prosternal lobe short, about one-half keel length, apical margin broadly rounded, marginal stria obsolete at sides; mesoventrite produced at middle, marginal stria complete, mesometaventral stria weakly arched forward, crenulate, complete, meeting lateral metaventral stria at sides, curving posterolaterad toward inner third of metacoxa, outer lateral metaventral stria about one-third length of inner stria, metaventral disk impunctate at middle; abdominal ventrite 1 with single lateral stria abbreviated apically, middle portion of disk with small secondary punctures scattered in basal half, smaller but more densely impressed along apical margin; protibia with 4 very weak marginal denticles, outer margin finely serrulate between; mesotibia with one marginal and 2–3 weak submarginal spines; outer metatibial margin smooth; propygidium without basal stria, discal punctures small, separated by slightly less than their diameters in basal half, smaller and much sparser apically; propygidial gland openings very small, present one-fourth from basal and lateral margins; pygidium with ground punctation fine, sparse, with few small secondary punctures along basal margin. Male genitalia (Fig. 85): T8 short, sides rounded, converging apically, basal emargination very shallow, apical emargination narrow, acute, ventrolateral apodemes extending about one-half distad from base, widely separated beneath; S8 halves approximate in basal third, inner margins diverging toward apex, lateral margins subparallel, apical guides widened only near apex, bearing a few conspicuous apical setae; T9 with proximal apodemes thin, about one-third total length, apices narrowly subacute, ventrolateral apodemes weakly dentate beneath; S9 apparently completely divided along midline, stem very narrow to base, head abruptly widened; tegmen with sides slightly sinuate, widest just beyond midpoint, very weakly bent ventrad near apex; median lobe simple, one-fourth tegmen length; basal piece very short, one-fifth tegmen length.

###### Remarks.

While the weakly dentate protibiae and poorly developed frontal carinae (Fig. 84B) of this species suggest relationships with the *Baconia micans* group, it could not be confused with any of them, as it is much more elongate, subdepressed, and non-metallic (Fig. 84A). The shallow but broad and distinctly crenulate elytral striae are quite distinctive, as is the basal connection of the 5^th^ elytral and sutural striae

###### Etymology.

This species is named in honor of our friend and colleague, Dr. Fernando Leivas, whose family’s hospitality during our 2011 visit was very generous. The lone specimen was collected on the family’s small ranch near Curitiba, Paraná.

##### Baconia atricolor sp. n.

http://zoobank.org/00BE6BFD-CB61-4A2B-9E14-6ADF2D8E2A95

http://species-id.net/wiki/Baconia_atricolor

Figs 84C–D 86 Map 23

###### Type locality.

COSTA RICA: Guanacaste: Sta. Rosa National Park [10.3°N, 85.62°W].

###### Type material.

**Holotype male**: “Est. Sta. Rosa, 300m, P.N. Sta. Rosa, Guanacaste Costa Rica, 13 a 28 jun 1992, III curso Parataxon. L-N 313000, 359800” / “INBIO CRI000492752 (INBIO). **Paratypes** (2): **COSTA RICA**:1: **Guanacaste**: P. N. Guanacaste, Los Almendros, 1-22.vii.1992, E. Lopez (INBI); 1: x.1983, D. Janzen & W. Hallwachs (INBI).

###### Other material.

**BRAZIL**: 1: **Mato Grosso**:Mpio. Cuiaba, Fazenda Mutuca, 15.3145°S, 55.9703°W, 6.iii.2009, FIT, J. Rocha (CEMT), 1: 6.xii.2008, FIT, F. Gava & J. Rocha (FMNH).

###### Diagnostic description.

**Figure 86.**
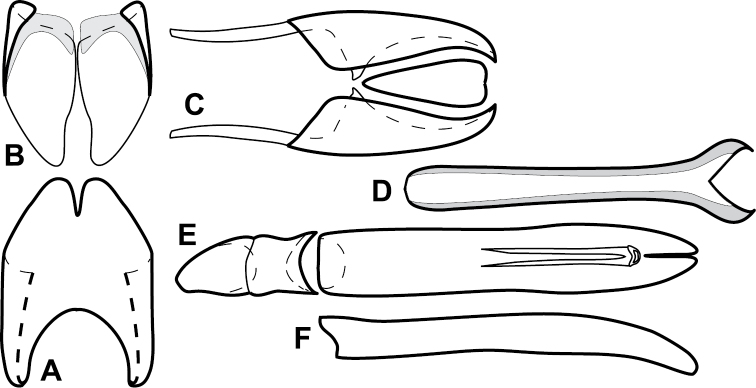
Male genitalia of *Baconia atricolor*. **A** T8 **B** S8 **C** T9 & T10 **D** S9 **E** Aedeagus, dorsal view **F** Aedeagus, lateral view.

Length: 1.7–2.0mm, width: 1.2–1.8mm; body broadly ovoid, strongly depressed, glabrous; rufopiceous throughout; frons weakly elevated over antennal bases, depressed in middle, ground punctation of frontal disk very fine, inconspicuous, with few irregularly scattered coarse punctures on epistoma, middle of frons and near vertex, frontal stria present along inner margin of eye and across front but narrowly interrupted over antennae, and more broadly interrupted at middle; supraorbital stria absent; epistoma broadly depressed, weakly emarginate apically; labrum about 3×wider than long, apical margin emarginate; antennal scape short, club rounded, expanded to apex; pronotal sides evenly arcuate to apex, marginal stria complete along lateral and anterior margins, weakly crenulate in front; lateral submarginal pronotal stria absent, pronotal disk not depressed in anterior corners, ground punctation of pronotal disk very fine, inconspicuous, sparsely interspersed with small secondary punctures in lateral thirds; elytra with two complete epipleural striae, outer subhumeral stria absent, inner subhumeral stria present in basal fifth, dorsal striae 1–2 complete, 3^rd^ stria slightly abbreviated apically, sinuate in apical half, 4^th^ stria present in basal third, weakly arched toward suture at base, 5^th^ stria absent, sutural stria present in apical half, elytral disk with sparse punctures in apical third; prosternum moderately broad, weakly convex, keel shallowly emarginate at base, carinal striae subparallel, nearly complete, may be united along extreme basal margin; prosternal lobe slightly over one-half keel length, apical margin rather rounded, marginal stria obsolete at sides; mesoventrite weakly produced at middle, marginal stria complete, mesometaventral stria strongly arched forward, crenulate, detached at sides; lateral metaventral stria extending posterolaterad toward middle of metacoxa, outer lateral stria present along basal two-thirds of inner stria, metaventral disk impunctate at middle; abdominal ventrite 1 with inner lateral stria curved mediad, abbreviated apically, outer lateral stria present as short postcoxal fragment, middle portion of disk impunctate; protibia very weakly 4-dentate, outer margin serrulate; mesotibia with two marginal spines; outer metatibial margin smooth; propygidium without basal stria, discal punctures separated by about their diameters in basal half, smaller and sparser apically; propygidial gland openings conspicuous, about one-third from basal and lateral margins; pygidium with ground punctation fine, uniformly interspersed with small, sparse secondary punctures. Male genitalia (Fig. 86): T8 slightly longer than wide, sides subparallel in basal two-thirds, narrowing to apex, basal emargination rather deeply arcuate, apical emargination narrow, ventrolateral apodemes extending to about midpoint beneath, separated by two-thirds T8 width; S8 halves approximate along most of midline, base rounded, sides subparallel, apical guides narrow, widened slightly toward apex, connected by fine dorsal membraneous velum; T9 with proximal apodemes thin, almost one-half total length, apices of dorsal lobes convergent, acute, ventrolateral apodemes strongly dentate, meeting at midline beneath; S9 desclerotized along midline, stem moderately broad, widening slightly toward base, apical arms slightly divergent, curved, apices acute; tegmen with sides subparallel in basal half, widened slightly near apex, rather thick, very weakly curved in lateral aspect; median lobe one-half tegmen length; basal piece one-third tegmen length.

###### Remarks.

This species is among the few strongly flattened, piceous species. Its pattern of elytral striation is also unique, with the 4^th^ stria represented by a basal arch, the 3^rd^ stria abbreviated and sinuate apically, and the sutural stria very short and apical (Fig. 84C). Preliminary phylogenetic analyses place this species among those of the *Baconia salobrus* group, and given its coloration this is conceivable. However, the body form is very different, being strongly flattened in *Baconia atricolor* as opposed to the strongly convex body form of all *salobrus* group species, and we prefer not to include it at this point. Male genitalia are similar, but without exhibiting any outstanding synapomorphies.

This species is known from a couple of broadly disjunct areas. The Brazilian specimens, from Mato Grosso, are in nearly all respects very similar to those from Costa Rica, but they have slightly denser lateral pronotal punctation, and have the S8 of the male genitalia more broadly expanded apically. Given the limited material available, we prefer to consider these unusual representatives of the same species, but we exclude them as types.

###### Etymology.

This species is named in reference to its black coloration, unusual among flattened *Baconia* spp.

## Discussion

*Baconia* does not appear to be closely related to any other New World Exosternini. Our unpublished preliminary analyses place the genus closest to the Middle Eastern *Spathochus* Marseul. Together these are nested within a clade of mainly Afrotropical Exosternini including *Cylistosoma* Lewis, *Pachycraerus* Marseul, and several others. In fact there is some indication that Platysomatini and Hololeptini might be related to these, and that assignment of *Baconia* to Exosternini might need reassessment. However, at present, there is inadequate support or taxon sampling at these deeper levels of the Histerinae tree to support any major reclassification. It certainly represents an important future question.

Interestingly, given the Old World relationships of *Baconia*, the Old World *Baconia* species are decidedly not, in any sense, early-branching sisters to the New World species. In fact the Old World species appear to represent two different New World lineages that have dispersed westward into Southeast Asia. All but one of the Asian species are relatively deeply nested members of the *Baconia aeneomicans* group. This appears more or less consistent with the presence of two species of this group (*Baconia aeneomicans* and *Baconia stephani*) occurring in the US, the most northern extension of the group, perhaps pointing to a Beringian connection. The relationships of the Malaysian *Baconia glauca* are very unclear, although preliminary analyses place it well inside the genus, weakly supported as close to *Baconia maculata*, *Baconia viridimicans*, and *Baconia ruficauda*. Even regarding any of these as possible relatives, *Baconia glauca* would be highly divergent. Resolving its relationships and biogeographical history will require additional analysis.

The phylogenetic analyses conducted to date have focused primarily on higher level relationships and on delineating meaningful genera within the Exosternini. As such there have been relatively few characters informative only within genera included, and relationships among species of *Baconia* remain inadequately resolved at this point. Nonetheless, a few relationships of interest are worth highlighting. The most well-supported relationships are those of the *Baconia aeneomicans* group and relatives, and the paraphyly of the more generalized *aeneomicans* group species with respect to the *cylindrica* and *gibbifer* groups (both of which are supported as monophyletic) is indicated as described above. The *Baconia angusta* group is basically recovered as monophyletic, although with the inclusion of *Baconia purpurata*, which we do not place in this group. The latter species is represented only by females, and cannot be considered well-placed given the characters available. Similarly, the *Baconia salobrus* group is monophyletic only with the inclusion of *Baconia atricolor*, which we provisionally exclude from it given the strong differences in body form. If more detailed analyses support this relationship, we would revise this judgement. The generally larger, flatter *Baconia loricata* group is recovered as monophyletic, without close relatives. The *Baconia riouka* group is resolved to also include *Baconia fulgida* (but not other species of the *Baconia micans* group). Other than these, the remaining species groups appear variably non-monophyletic. The *Baconia insolita* group is split into a basal lineage (*insolita* + *burmeisteri*), sister to the remainder of the genus, and a more deeply nested *tricolor* + *pilicauda* clade as sister to the *aeneomicans* group. The *Baconia famelica* group shows a similar result, with *Baconia cavifrons*, *Baconia famelica*, *Baconia grossii*, and *Baconia redemptor* forming a basal grade (immediately within the basal *insolita* group bifurcation), and the remaining species forming a more deeply nested clade. Beyond this, relationships among *Baconia* species deserve much greater scrutiny. Many more characters are available for resolving relationships within *Baconia*, and we plan future studies to examine these in more detail.

Life history details for species of *Baconia* are disappointingly few. The most frequent records point to under-bark associations, as the flattened body form of many species obviously suggests. A few records, and our own few observations suggest that these species are usually associated with relatively recently killed trees, rather than older, rotten logs. However, some exceptions may occur, with *Baconia isthmia*, for example, having been collected in a ‘fungusy log’. Supporting this basic observation are the records for several species having been attracted to various bark beetle pheromone lures (primarily in the work of Carlos Flechtmann). An interesting variant is the observation of numerous *Baconia patula* in association with bamboo, presumably (although not known for certain) under the leaf sheaths along the major stems. Only a few other specific associations have been recorded, including rotting fruit (*Baconia eximia*), orchid (*Baconia dives*), leaf cutter ant refuse (*Baconia plebeia*), arboreal moss (*Baconia grossii*), and tobacco (*Baconia burmeisteri*), but most of these represent unique observations for species generally collected by other means. So while they may help guide further searching for associations, we consider none of them definitive for the various species. A fairly large number of species has been taken in canopy fogging samples, with at least a small proportion of these likely to be canopy specialists. However, the microhabitat preferences of these are unknown and likely varied. By far the majority of specimens and species are known only from passive interception traps, and their true habits remain a mystery.

While this study has expanded the diversity and concept of the genus *Baconia* considerably, there is undoubtedly very much still to learn, even at the alpha diversity level. It is remarkable that such an attractive group of beetles should have been completely ignored for so long, and we hope that this work makes them much more tractable and appealing to work on in the future.
